# Child Mortality Estimation 2013: An Overview of Updates in Estimation Methods by the United Nations Inter-Agency Group for Child Mortality Estimation

**DOI:** 10.1371/journal.pone.0101112

**Published:** 2014-07-11

**Authors:** Leontine Alkema, Jin Rou New, Jon Pedersen, Danzhen You, all members of the UN Inter-agency Group for Child Mortality Estimation and its Technical Advisory Group

**Affiliations:** 1 Department of Statistics and Applied Probability, National University of Singapore, Singapore, Singapore; 2 Saw Swee Hock School of Public Health, National University of Singapore, Singapore, Singapore; 3 Fafo Institute for Applied International Studies, Oslo, Norway; 4 United Nations Children’s Fund, New York, New York, United States of America; Université Catholique de Louvain, Belgium

## Abstract

**Background:**

In September 2013, the United Nations Inter-agency Group for Child Mortality Estimation (UN IGME) published an update of the estimates of the under-five mortality rate (U5MR) and under-five deaths for all countries. Compared to the UN IGME estimates published in 2012, updated data inputs and a new method for estimating the U5MR were used.

**Methods:**

We summarize the new U5MR estimation method, which is a Bayesian B-spline Bias-reduction model, and highlight differences with the previously used method. Differences in UN IGME U5MR estimates as published in 2012 and those published in 2013 are presented and decomposed into differences due to the updated database and differences due to the new estimation method to explain and motivate changes in estimates.

**Findings:**

Compared to the previously used method, the new UN IGME estimation method is based on a different trend fitting method that can track (recent) changes in U5MR more closely. The new method provides U5MR estimates that account for data quality issues. Resulting differences in U5MR point estimates between the UN IGME 2012 and 2013 publications are small for the majority of countries but greater than 10 deaths per 1,000 live births for 33 countries in 2011 and 19 countries in 1990. These differences can be explained by the updated database used, the curve fitting method as well as accounting for data quality issues. Changes in the number of deaths were less than 10% on the global level and for the majority of MDG regions.

**Conclusions:**

The 2013 UN IGME estimates provide the most recent assessment of levels and trends in U5MR based on all available data and an improved estimation method that allows for closer-to-real-time monitoring of changes in the U5MR and takes account of data quality issues.

## Introduction

Millennium Development Goal 4 (MDG 4) calls for a reduction of the under-five mortality rate (U5MR) by two-thirds between 1990 and 2015 [1]. With only two years remaining before the deadline of the goal, the global spotlight has never been more strongly focused on the child mortality estimates on which progress towards MDG 4 at the global and country level is assessed. This underscores the need for better communication of the estimates and greater transparency in the estimation process.

Every year, the United Nations Inter-agency Group for Child Mortality Estimation (UN IGME, comprising the United Nations Children’s Fund, the World Health Organization, the United Nations Population Division and the World Bank) updates its estimates on child mortality including neonatal, infant and under-five mortality rates (U5MR) [2], [3]. The UN IGME estimates are widely used by the UN and its agencies, non-governmental organizations, donors, governments and researchers to monitor progress towards MDG 4. Yearly updates are provided to take account of newly available data and for a review of estimation methods used, to update methods where deemed necessary. As a result, country-specific estimates from various rounds may differ slightly (in most cases) or significantly and may not be comparable due to differences in database or methods used to derive the estimates. Understanding the differences between estimates from different rounds and the reasons for them is critical for improved transparency of estimates of child mortality indicators.

In the most recent UN IGME publication in 2013 [2], estimates of the U5MR were based on an updated database as well as a revised estimation method as compared to the 2012 publication [3]. The new method, the Bayesian B-spline Bias-reduction (B3) method, was chosen to replace the previously used method because it better accounts for data errors (including biases and sampling and non-sampling errors in the data), provides a more flexible trend fitting method and resulted in improved model validation [4]. The objectives of this paper are to communicate the reasons for the change in estimation method and to summarize and explain differences between the U5MR estimates published in September 2013 by the UN IGME (referred to as the UN IGME 2013 estimates) and those published by the UN IGME in 2012 [5]–[12].

The paper is organized as follows: we first summarize the database and modelling approach used for the construction of the UN IGME 2013 U5MR estimates, highlight differences with the UN IGME 2012 database and estimation method, and explain the rationale for the change in estimation methods. We then give an overview of differences and perform a decomposition exercise of these differences between the UN IGME 2013 and UN IGME 2012 U5MR estimates to identify and explain the key drivers of those differences. Finally, we analyse differences in the number of global and regional deaths and decompose differences in those due to updated U5MR estimates and those due to updated population numbers.

## Methods

### Overview of the estimation process for the UN IGME 2013 estimates

The UN IGME compiles data on U5MR annually from various sources, typically vital registration (VR) systems, surveys and censuses. These data sources either record recent births and deaths on an ongoing basis or collect retrospective information on child mortality in the form of full or summary birth histories of women. From such data, it is possible to construct observations of U5MR directly from the reported births and deaths below age 5 or indirectly via models applied to the information from summary birth histories [5]. However, different data sources may yield different estimates of U5MR for a given time period because of differences in data errors, for example random errors in sample surveys or systematic errors due to misreporting. Additionally, data may not be available for all (recent) years of interest. To obtain country-specific U5MR estimates which are comparable across time within countries, as well as across countries, trend fitting procedures are used.

While UN IGME 2012 and 2013 U5MR estimates are based on similar input data sources, the three key differences are that for the UN IGME 2013 estimates (1) the database was extended, (2) a new trend fitting method was used, and (3) data quality issues were taken into account. The UN IGME 2013 database and methods are explained below in more detail and differences with the UN IGME 2012 database and methods are highlighted. An overview of differences in UN IGME 2012 and 2013 methods is given in Table 1.

**Table 1:** Overview of differences in modelling approach and datasets used by UN IGME 2012 and UN IGME 2013, for estimating the U5MR and the number of under-five deaths.

	UN IGME 2012	UN IGME 2013
**1. Estimation method**		
1a. Default smoothing method	Loess smoother [5].	Penalized B-spline regression [4].
1b. Uncertainty assessment	Bootstrap method [12].	Uncertainty assessed through estimation in the Bayesian framework whereby uncertainty in all model parameters is accounted for.
1c. Countries with conflicts,natural disasters orlimited numberof observationswith dubious quality	Modified estimation method (changing thesmoothing parameter alpha to bettercapture trends or using an adjusted methodwith crisis mortality subtracted from dataand later added to the crisis-free fit) basedon expert opinion and evidence from othersources such as health interventionand coverage indicators.	Same as UN IGME 2012: Crisis mortality subtracted from data and later added to the crisis-free fit.
	A piecewise straight line was usedfor Democratic Republic of Congo.A straight line was used for Nauru and Somalia.	Constant fit used for conflict years in Democratic Republic of Congo and Somalia.
	Estimates from WPP 2010 were usedfor the People’s Democratic Republic of Korea.	Estimates from the WPP 2012 were used for the People’s Democratic Republic of Korea.
**2. VR data**	VR data were adjusted for 12European countries [5].	Same as UN IGME 2012: VR data were adjusted for 12 European countries.
	VR data from the World HealthOrganization were calculated forsingle-year periods.	VR data from the World Health Organization were recalculated for longer periods for smaller countries where the coefficient of variation of the observation was larger than 10% due to small numbers of births and deaths (when available).
	Incomplete VR data were not used.	In 10 selected CEE/CIS countries, incomplete VR data in recent years were set as the minimum and observations in the early 1990s were used to inform the trend in estimates. An assumed level of completeness of the VR data was assumed in some cases.
	All included observations were treatedequally (error variance follows fromoverall error variance in country).	Stochastic error variance for VR observations accounted for in the model fitting.
**3. Data from surveys and censuses**		
3a. Exclusion of datasources/observations	Surveys were excluded if they areconsistently below other data sources,or if data quality issues had been reported.	Same as UN IGME 2012.
3b. Indirect estimates from surveysand censuses	Methods: UN Manual X methodologywas applied to aggregatedata (excludes recent points basedon reports of women 15–19, 20–24).	Methods: Same as UN IGME 2012.
	All included observations were treatedequally (error variance follows fromoverall error variance in country) andassumed to be unbiased.	Sampling errors were calculated (where micro-data is available, else a relative standard error is assumed) and accounted for; non-sampling error variance parameters were estimated by source type. Slope and level biases were estimated for each data series except for series with source dates before 1975, where the level bias was assumed to be equal to the mean bias for all surveys from the respective source type.
3c. Direct estimates(e.g. from DHSs)	Methods: Pederson & Liu 2012.	Methods: Same as UN IGME 2012.
	All included observations were treated equally (error variance follows from overall error variance in country) and assumed to be unbiased.	Sampling errors were calculated and accounted for; non-sampling error variance parameters were estimated by source type. Slope and level biases were estimated for each data series except for series with source dates before 1975, where the level bias was assumed to be equal to the mean bias for all surveys from the respective source type.
**4. HIV countries**	Methods: Observations and estimation procedures wereadjusted to account for selectionbias resulting from HIV.	Methods: Same as UN IGME 2012.
	Information used from UNAIDS 2011.	Information used from UNAIDS 2012.
**5. Estimation of infant** **mortality rate**	Methods: Loess smoother mostlyfor countries with high-qualityVR data, model life table otherwise.	Methods: B3 model for countries with high-quality VR data, model life table otherwise.
**6. Estimation of** **under-five deaths**	Method: Central mortality rates appliedto estimated populations (probability ofdying converted to central mortality rate).	Method: Same as UN IGME 2012.
	Estimated populations taken from WPP 2010.	Estimated populations taken from WPP 2012.

#### Database

The UN IGME 2013 database, which contains the underlying data used for estimation, is publicly available on the website http://www.childmortality.org (referred to as Child Mortality Estimates (CME) Info). For the 2013 estimates, a substantial amount of newly available data was incorporated: data from about 50 surveys and censuses conducted since 2009 for almost 50 countries, data from more than 30 surveys and censuses conducted before 2009 for more than 10 countries and new data from VR systems for about 90 countries.

In 2013, the methods for constructing U5MR observations from micro-data on full birth histories were identical to those used for the 2012 estimates [5], [7]. For summary birth histories, all surveys with available micro-data were recalculated in 2013, and standard errors were estimated using a Jackknife variance estimation method similar to the one used for full birth histories [7]. In general, the point estimates from surveys with summary birth histories were identical to those in the 2012 database, except with a few corrections. Resulting observations, standard errors and the model life tables used to construct the observations are given in the database on CME Info. For surveys without available micro-data, standard errors of 10% were assumed (in line with the mean/median standard errors observed in other data sources), while 2.5% was used for censuses (this value was used for any missing standard or stochastic error for population-based outcomes) [4].

For VR data with available data on the number of deaths and mid-year populations, annual observations were constructed if the coefficient of variation was less than 10%. For country-years in which the coefficient of variation exceeded 10%, deaths and mid-year populations were pooled over longer periods to reduce spurious fluctuations in countries where small numbers of birth and deaths were observed [4].

#### Trend fitting method and construction of uncertainty intervals

For the UN IGME 2012 estimates, Loess (locally weighted least squares) regression was used, whereas for UN IGME 2013 estimates, a penalized B-spline regression model was used to derive trend estimates. In the Loess regression approach used in 2012, the estimates of U5MR were obtained by local fits of a weighted linear regression model to the data. A smoothing parameter alpha (*α*) determined the range of points included in each local fit and their weights (the flexibility of the fitted trend line decreases with *α*). Default settings for *α* were based on the number of surveys and data points from VR in each country [5]. Expert-based adjustments of *α* were used for countries where the Loess smoother did not capture the recent trend in the data. A bootstrap method was used for constructing uncertainty intervals [12].

**Figure 1:**
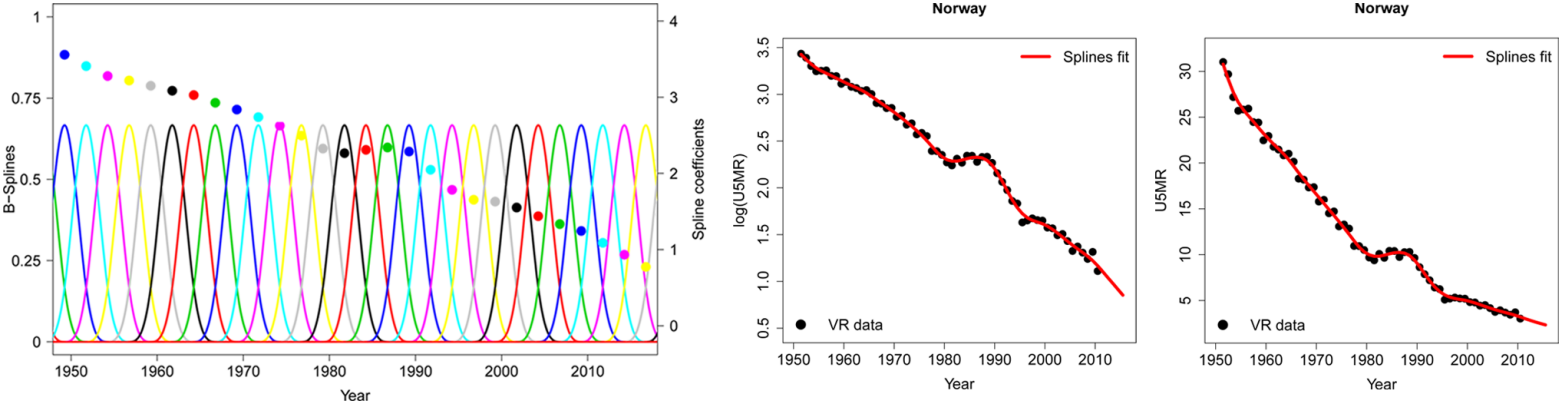
Illustration of the B-spline regression model for Norway. From left to right: B-splines and their corresponding spline coefficients (plotted in the same color), observed log(U5MR) and U5MR (black dots) plotted against time, together with the spline estimates (red line). The spline estimate for log(U5MR) in each year is the sum of the non-zero B-splines in that year weighted by their respective spline coefficients.

For the UN IGME 2013 estimates, a penalized B-spline regression model was used for U5MR trend fitting as a flexible alternative to the Loess smoother to better capture recent trends in data [13]–[16]. The B-spline method is illustrated in Figure 1 for Norway. The B-splines used in this application are smooth curves that add up to unity at any point in time. All B-splines were placed 2.5 years apart. This setting was determined through validation exercises [4] that showed that fits were similar up to a spacing of 3 years, but short-term fluctuations were not captured with a spacing of more than 3 years; a spacing of 2.5 years was hence chosen for ease of interpretation. For any year, the estimated U5MR (on the natural logarithmic scale) is the sum of the (non-zero) spline functions evaluated in that year multiplied by their corresponding spline coefficients. When estimating the spline coefficients, a flexible yet reasonably smooth U5MR curve was obtained by including a penalization of changes in spline coefficients. In the resulting spline fit, the difference between two adjacent coefficients is given by the difference between the previous two coefficients with an estimated data-driven “distortion term” added to it. For example, in Norway during the early 1980s, these distortion terms are estimated to be around zero when U5MR did not change much, but they are negative in the late 1980s when the U5MR started to decline again. The variance of the distortion terms plays the role of the smoothing parameter in the splines fit: larger variance allows for greater fluctuations in the distortion terms, thus greater variations in the trend from one period to the next. The country-specific smoothing parameter was estimated through a hierarchical model. Global smoothing settings were used for a subset of 57 countries [17], which were countries with both VR and non-VR data, countries with VR data but with gaps of more than 5 years in the VR data, as well as small countries with less than 10,000 live births in 2012 [18].

U5MR projections, after the end of the most recent observation period, were obtained by projecting forward the differences between adjacent spline coefficients. These projected differences were based on a combination of country-specific projected differences in spline coefficients, which are based on the country’s recent past differences, and a global distribution of observed past differences. Uncertainty in U5MR was assessed through estimation in the Bayesian framework whereby uncertainty in all model parameters is accounted for.

#### Data quality issues

An observed value for U5MR can be considered as the true value for U5MR multiplied by an error factor, i.e. observed U5MR  =  true U5MR×error, or on the log-scale, log(observed U5MR) = log(true U5MR)+log(error), where error refers to the relative difference between an observation and the truth. In the UN IGME 2012 approach, all observations in a country were treated equally, or in other words, no distinction was made between errors in observations. In the UN IGME 2013 approach, differences in the expected values of the errors (or biases) as well as the differences in variance of (or uncertainty in) errors were incorporated through the inclusion of a data quality model [4].

In the data quality model, for each data series (e.g., all observations from a particular Demographic and Health Survey (DHS) in a given country), biases in log-transformed observations were modeled as a linear function of time to capture any level and trend bias in that particular data series. These unknown level and trend biases were estimated for all data series through the inclusion of a multilevel model, which was used to exchange information about mean biases and their variance across data series of the same source type (e.g., to exchange information about biases across all data series from DHSs, or across all data series from censuses). Differences in the variance of observations were also accounted for in the data quality model. Such differences arise from differences in sampling or stochastic variance, as well as differences in non-sampling variance, which refers to the occurrence of random errors at any point of the data collection and pre-processing phase. Sampling/stochastic variance was calculated before fitting the model, while non-sampling variance was unknown and estimated by source type. The estimation of the spline parameters and data model parameters occurs simultaneously in the Bayesian model.

#### Additional adjustments

In the UN IGME 2012 and 2013 estimation approach, additional adjustments were carried out, as explained in Table 1. Below we summarize the UN IGME 2013 adjustments and highlight differences with the UN IGME 2012 adjustments.

#### Countries in Central and Eastern Europe/Commonwealth of Independent States (CEE/CIS)

For a subset of 10 countries in the regional grouping of CEE/CIS, VR data were considered to be incomplete and mostly excluded in the trend fitting in earlier rounds of estimation. These countries were: Armenia, Azerbaijan, Georgia, Kazakhstan, Kyrgyzstan, Moldova, Tajikistan, Turkmenistan, Ukraine and Uzbekistan. However, the VR data showed a plateau or increase in U5MR in the early 1990s, which was considered to be indicative of the true trend in U5MR in these countries. For this round of estimation, two observations in the period 1990–1995 were included per country to inform the trend during that period but not the level estimates. Moreover, for some of these countries where the U5MR extrapolations were below the incomplete VR observations in the recent period from 2005 onwards, these VR observations were included in the model as a minimum bound (while accounting for stochastic errors). Furthermore, there were cases where the U5MR extrapolations were too far above VR observations for which there was an assumed minimum level of completeness; hence an upper bound was also included [4].

#### Countries with crises or limited observations of dubious quality

For the Democratic Republic of Congo and Somalia, the U5MR data available were not considered to be representative of the country’s past trend. A constant fit was estimated for the conflict period in each country. This was done by combining the B-splines lying within the conflict period such that only one spline coefficient is estimated for each conflict period [4].

For other countries that have experienced crises, the same crisis adjustment method was applied for both UN IGME 2013 and UN IGME 2012 estimates. However, updated estimates of the number of deaths for major natural disasters from the Centre for Research on the Epidemiology of Disasters International Disaster Database [19] and other sources (e.g. [20]) were used. The under-five proportion of deaths was estimated as described elsewhere [21]. Population numbers from the 2012 revision of the World Population Prospects (WPP 2012) [18] instead of the 2010 revision (WPP 2010) [22] were used to translate estimated numbers of under-five crisis deaths to under-five crisis mortality rates.

#### Countries with high HIV prevalence

There was no change in the adjustment method for 17 countries with high HIV prevalence [5], [6]. However, the HIV-related U5MR adjustments for data series based on full birth histories have been updated; the latest estimates from UNAIDS, referred to as the UNAIDS 2012 estimates [23], were used instead of the UNAIDS 2011 estimates [24].

#### Update in the estimation of the infant mortality rate

For countries with high-quality VR data (covering a sufficient period of time and deemed to have high levels of completeness and coverage), a model similar to the B3 model for U5MR was developed to construct IMR estimates. In the model for the IMR, a penalized spline regression model was used to estimate the trend in the ratio of the IMR to the median B3 estimate of U5MR in the corresponding country-year (on the logit scale, to restrict the IMR to be lower than the U5MR). In UN IGME 2012, the Loess smoother was used for this set of countries. For the remaining countries, the IMR was derived from the U5MR through the use of model life tables that contain known regularities in age patterns of child mortality [9], as in UN IGME 2012, but with revisions to the choice of the model life table for some countries [17].

#### Update in the inputs for calculating the number of under-five deaths

For UN IGME 2012 and 2013 under-five death estimates, the number of estimated under-five deaths was estimated through a life table approach, using estimates of the population below age 1 and between ages 1 to 5. For UN IGME 2013 estimates, the population estimates were taken from WPP 2012 [18], while for UN IGME 2012 estimates, WPP 2010 was used [22].

All computations were carried out using open-source software packages R [25] and JAGS [26]. Code is available from the authors upon request.

### Decomposition of differences in U5MR and under-five deaths

We examined how much of the difference in U5MR estimates between the UN IGME 2013 and UN IGME 2012 estimates were caused by the use of the updated database versus a new modelling approach, by decomposing the differences in U5MR estimates for countries where in 2012 the default Loess approach was used (180 countries in total). This decomposition was carried out for the U5MR in 1990 and 2011.

If Δ*U* represents the total difference in U5MR between the UN IGME 2013 estimates (denoted by *U_B3_*) and the UN IGME 2012 estimates (denoted by *U_Loess_*), and if the Loess fit to the 2013 UN IGME database is denoted by U*_Loess_ in a particular year, the decomposition of differences in U5MR is given by:
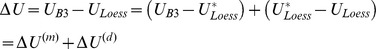
where Δ*U^(m)^* represents the difference “due to the use of the B3 modelling approach” (the difference between the B3 and Loess fits to the 2013 database) and Δ*U^(d)^* represents the difference “due to different databases” (the difference between the Loess fits to the 2013 and 2012 databases).

Subsequently, for countries where the difference due to the change in estimation method, Δ*U^(m)^*, is larger than 10 deaths per 1,000 live births, we further decomposed the differences into differences that are due to the change from Loess to B-splines and differences that are due to the change from treating all observations equally to the approach where the data quality model is included:
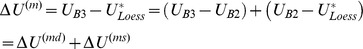
where Δ*U^(m)^* is the difference between the B3 and Loess fits to the 2013 database, Δ*U^(md)^* represents the difference due to the inclusion of the data quality model” and Δ*U^(ms)^* represents the difference “due to the use of a different smoothing method” (the use of B-spline regression over the Loess smoother). To construct this decomposition, we fitted the B-splines to the 2013 database without including a data quality model. This model is referred to as the Bayesian B-spline model (B2 model), and the resulting estimates are denoted by *U_B2_*.

Lastly, we decomposed differences in the number of deaths in a similar matter, to obtain differences “due to the update in population estimates from WPP 2010 to WPP 2012” and differences “due to the updates in U5MR estimates”. This decomposition was carried out for MDG regions and the world.

## Results

### Comparison of UN IGME 2013 and UN IGME 2012 estimates

The reference years 1990 and 2011 were selected for comparison purposes, as 1990 is the start year for MDG 4, while 2011 is the last common published year for the UN IGME 2013 and UN IGME 2012 estimates.

**Figure 2:**
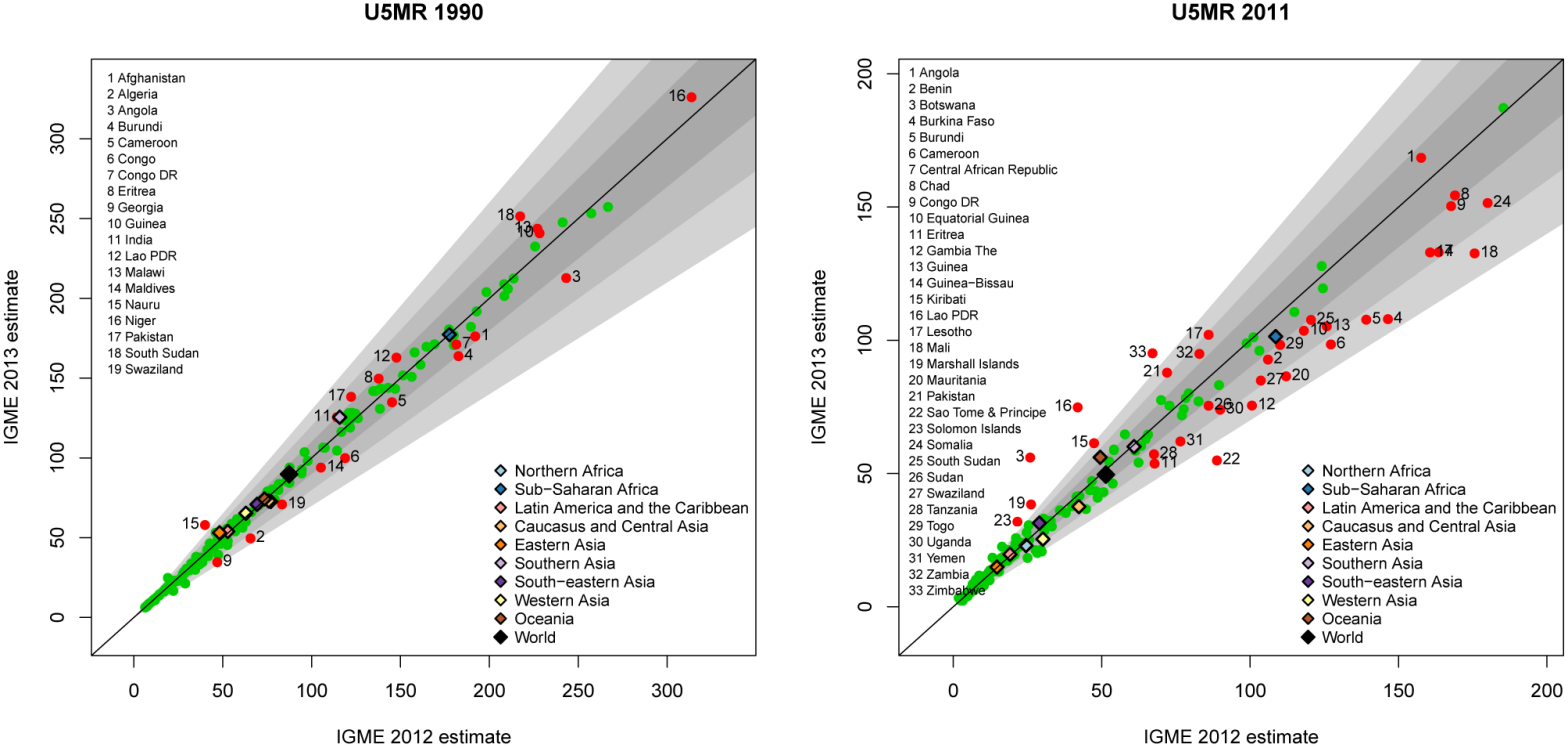
UN IGME 2013 and UN IGME 2012 estimates of the U5MR for the years 1990 (left) and 2011 (right). UN IGME 2013 estimates are plotted against UN IGME 2012 estimates. Gray areas represent relative differences of up to 10%, 20% and 30% respectively. Country-specific U5MR estimates are displayed as green points, or highlighted in red if the estimates differ by more than ten deaths per 1,000 live births. Regions are colored according to the given legend.

At the global level, differences between the updated UN IGME 2013 estimates and the UN IGME 2012 estimates were small. For the U5MR in 1990, the updated UN IGME 2013 estimate was 2.9% higher than the UN IGME 2012 estimate, an increase from 87.3 to 89.8 deaths per 1,000 live births. Conversely, for the U5MR in 2011, there was a 3.5% decrease from 51.4 to 49.6 deaths per 1,000 live births for 2013 and 2012, respectively. On the regional level, differences between the UN IGME 2013 and UN IGME 2012 estimates for the U5MR in both years were also small, with relative differences not exceeding 10% (see Figure 2). For individual countries, differences did exist as shown in Figure 2, and there are more countries with larger differences for the U5MR in 2011 (33 countries with a difference of more than 10 deaths per 1,000 live births) as compared to the U5MR in 1990 (19 countries with a difference of more than 10 deaths per 1,000 live births). The main reasons for the differences in these countries will be discussed in the next section. UN IGME 2012 and 2013 estimates for all countries are given in Figure S1.

Likewise, the differences between the UN IGME 2013 and UN IGME 2012 estimated numbers of under-five deaths were small. The UN IGME 2013 estimate of the global number of under-five deaths in 1990 was 5.5% higher than the UN IGME 2012 estimate (an increase from nearly 12 million to 12.6 million). For 2011, the UN IGME 2013 estimate of 6.8 million was 2.3% lower than the UN IGME 2012 estimate of 6.9 million.

**Figure 3:**
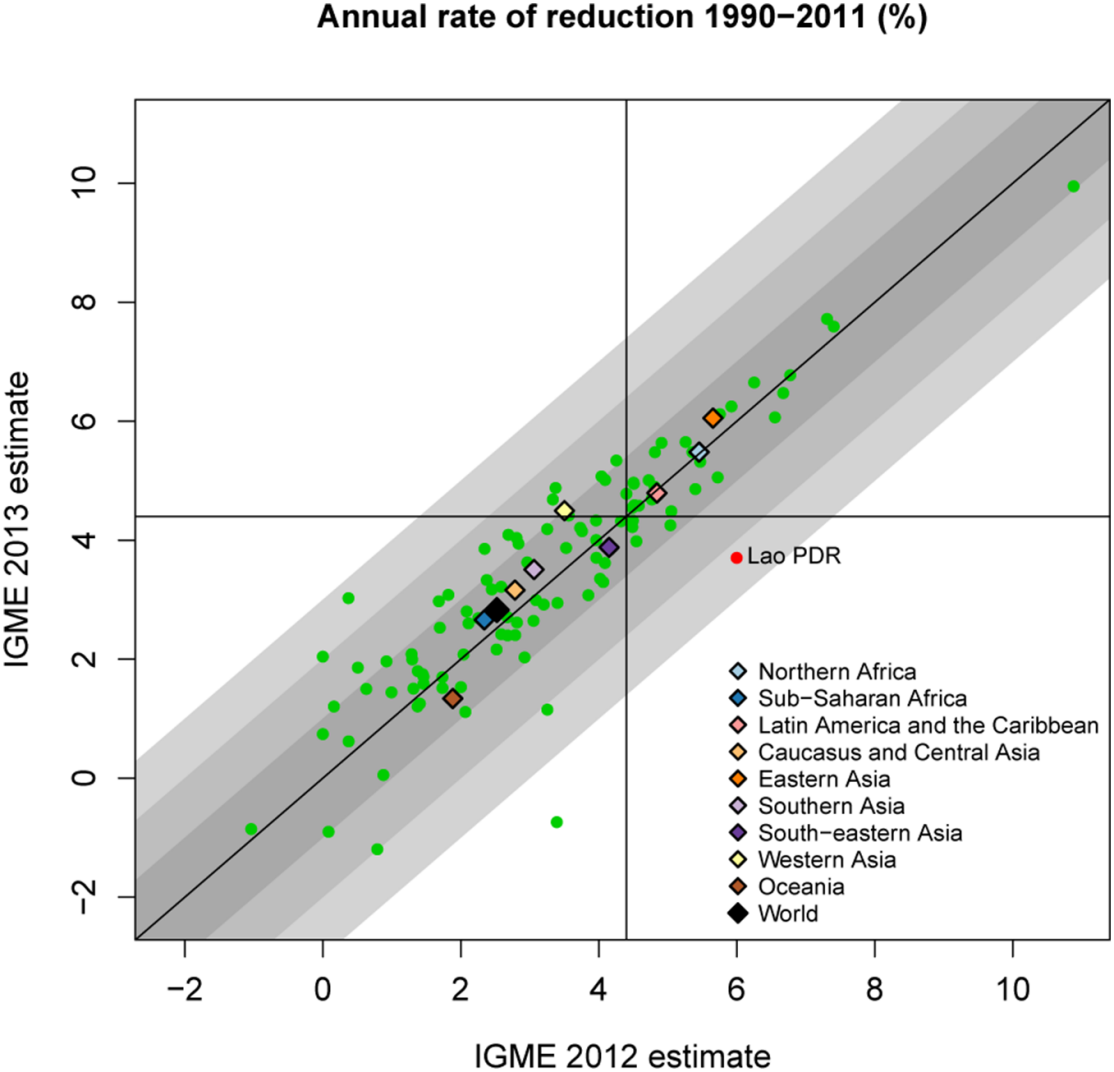
UN IGME 2013 and UN IGME 2012 estimates of the annual rate of reduction for 1990–2011. UN IGME 2013 estimates are plotted against UN IGME 2012 estimates. Gray areas represent absolute differences of up to 1%, 2% and 3% respectively (absolute difference). Country-specific ARR estimates are plotted in green for high mortality countries (with U5MR in 1990 of at least 40 deaths per 1,000 live births), or in red for a subset of these if the difference is at least 2% and the UN IGME 2013 and UN IGME 2012 estimates disagree with respect to whether the country is on track to meet MDG 4 (4.4% annual rate of reduction). Regions are colored according to the given legend.

Generally, differences between the UN IGME 2013 and UN IGME 2012 estimates in the annual rate of reduction (ARR) in U5MR from 1990 to 2011 were also small (see Figure 3). Among the high mortality countries, defined here as the countries with a U5MR of at least 40 deaths per 1,000 live births in 1990, there was only one country, the Lao People's Democratic Republic (Lao PDR), for which the UN IGME 2012 ARR point estimate suggested that the country was on track with respect to MDG 4, while based on the UN IGME 2013 estimates, it was not (see Discussion).

### Decomposition of differences in U5MR

#### Differences due to the updated database versus new B3 estimation method

**Figure 4:**
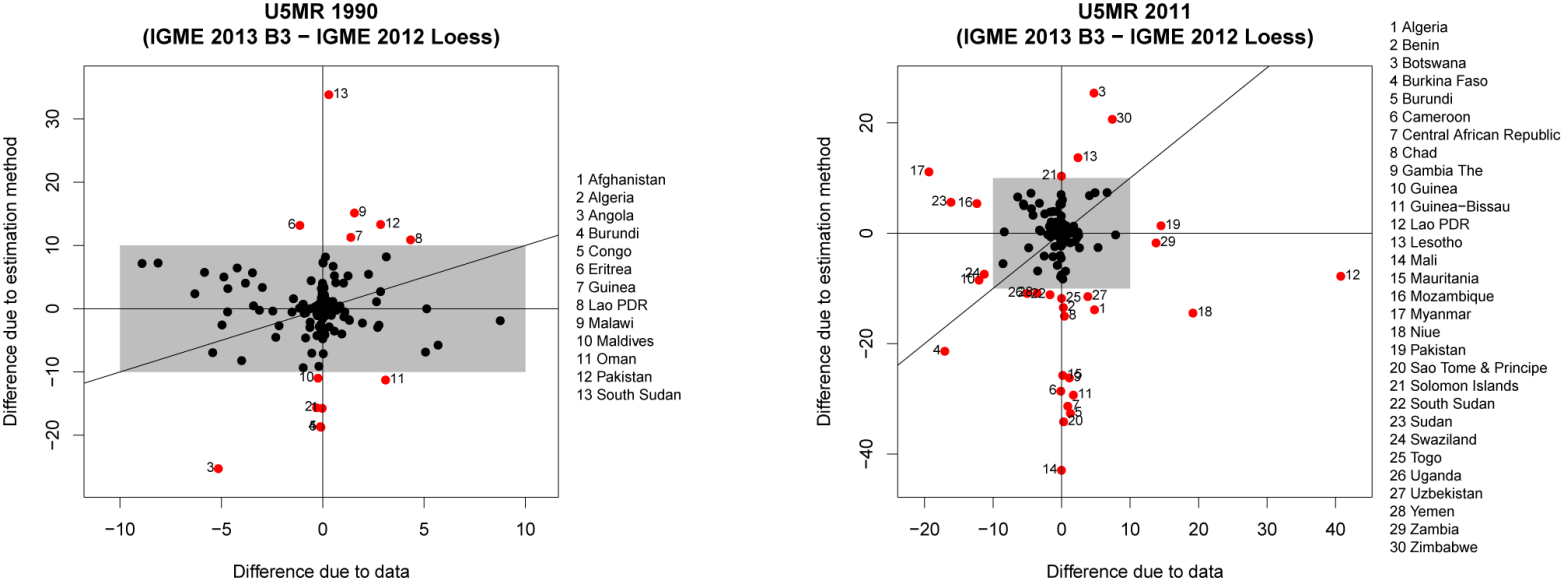
Decomposition of differences in U5MR for 1990 and 2011 into differences due to estimation method and differences due to data. The gray box represents differences up to 10 deaths per 1,000 live births. Countries with differences of more than 10 deaths per 1,000 deaths due to either factor are highlighted in red.

The decomposition of the difference in the U5MR for a country in a particular year into differences that are due to differences in databases and due to using the B3 model instead of the Loess smoother is summarized in Figure 4 for 1990 and 2011. In the figure, differences due to the new estimation method Δ*U^(m)^* are plotted against the differences due to the data Δ*U^(d)^* for all countries where in 2012 the default Loess smoother was used. Focusing on countries where differences are greater than 10 deaths per 1,000 live births (countries outside the gray box), the figure illustrates that in 1990, such changes were due to the change in estimation method while for 2011, both changes in estimation method as well as changes in database explained the differences.

**Figure 5:**
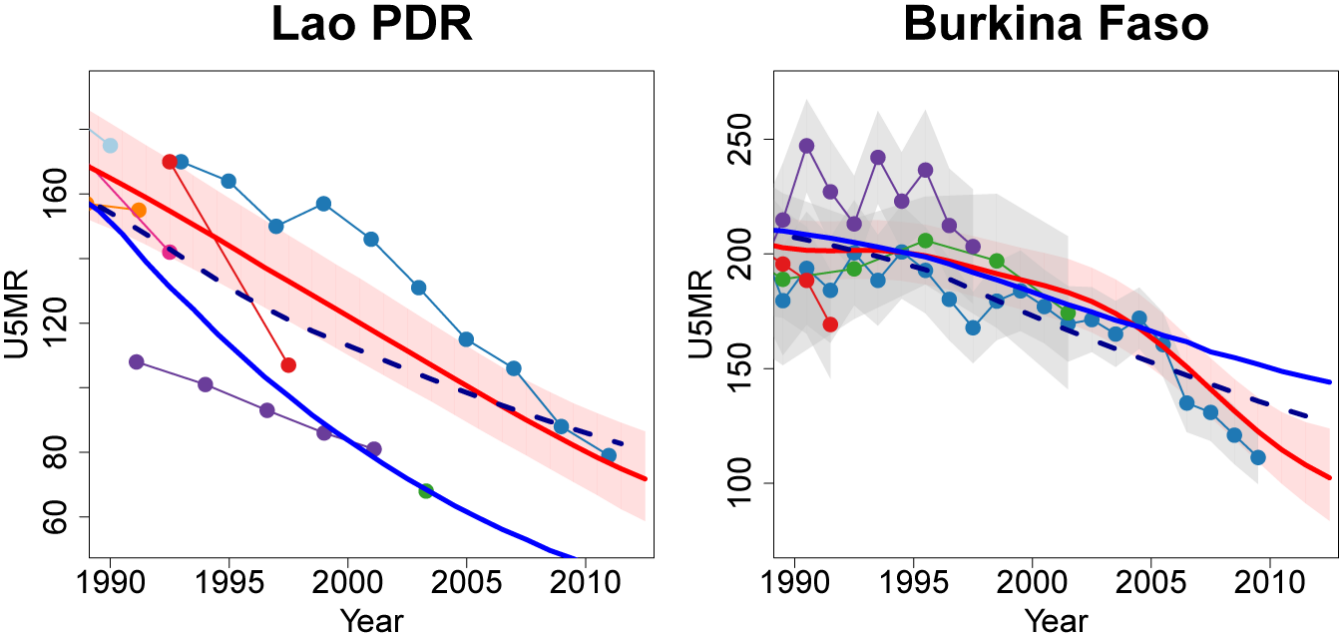
Comparison of U5MR estimates for Lao PDR and Burkina Faso where the change in database changed the estimate by more than 10 deaths per 1,000 live births. Estimates compared are from UN IGME 2013 (solid red line with 90% credible intervals given by the shaded regions), default Loess fit to 2013 database (dashed dark blue line) and UN IGME 2012 (solid dark blue line). Connected dots denote data from the UN IGME 2013 database and gray shaded areas around series of observations represent the sampling variability in the series (quantified by two times of the sampling standard errors). The newly-added/updated series for Lao PDR and Burkina Faso are those shown in dark blue. Excluded data series and detailed information on all data series are displayed in Figure S1.

Countries where the change in database changed the estimate by more than 10 deaths per 1,000 live births in 2011 are Burkina Faso, Guinea, Lao PDR, Myanmar, Niue, Pakistan, Sudan, Mozambique, Swaziland and Zambia. In general, the differences are due to newly-added/updated data series that showed higher (e.g. for Lao PDR, Pakistan and Zambia) or lower (e.g. for Burkina Faso, Guinea, Myanmar, Sudan, Mozambique) levels of U5MR than previously expected based on older data. Estimates for example countries are shown in Figure 5.

#### Differences due to new trend fitting method versus new data quality model

**Figure 6:**
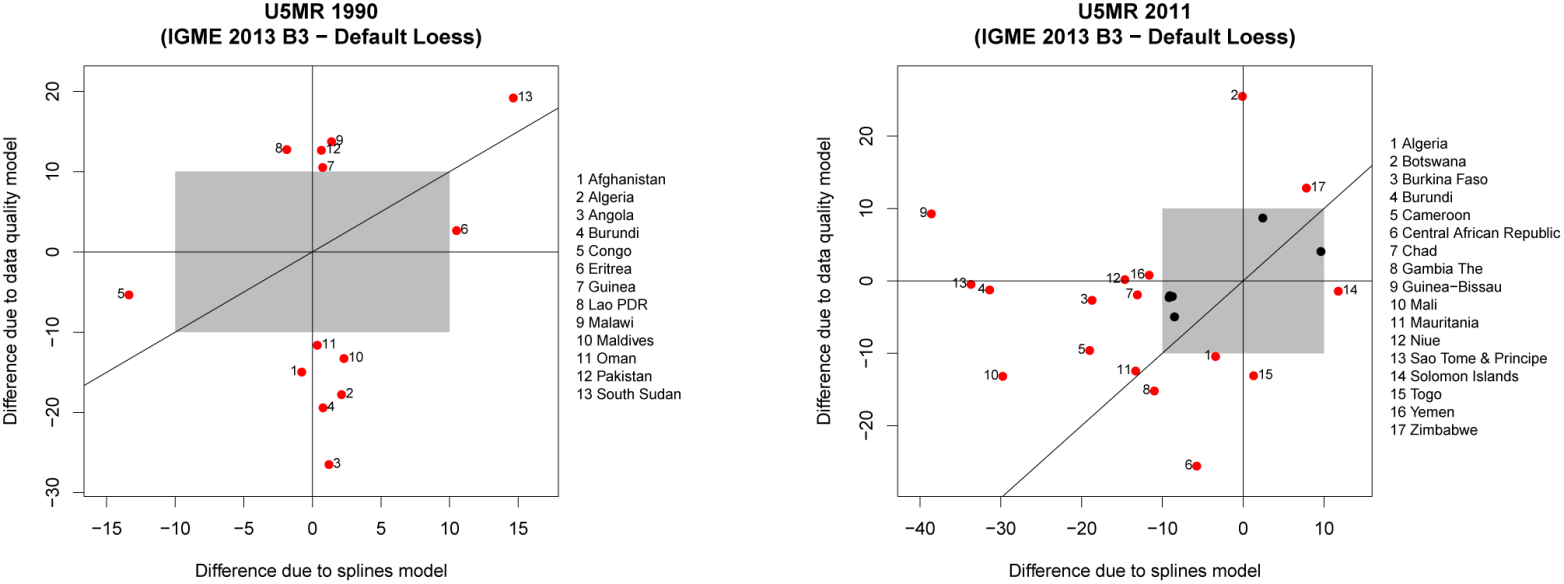
Decomposition of differences in U5MR for 1990 and 2011 into differences due to data quality model and differences due to splines model. The gray box represents differences up to 10 deaths per 1,000 live births. Countries with differences of more than 10 deaths per 1,000 deaths due to either factor are highlighted in red.

The decomposition of the difference in the U5MR for a country in a particular year into differences that are due to the inclusion of the data quality model versus differences that are due to the curve fitting (using the B3 model instead of the Loess smoother) are summarized in Figure 6 for 1990 and 2011, where the differences due to the data quality model (Δ*U^(md)^*) are plotted against the differences due to the splines model (Δ*U^(ms)^*). This exercise was performed only for countries where in 2012 the default Loess smoother was used and the difference in U5MR in the year of interest due to the change in estimation method is more than 10 deaths per 1,000 live births, i.e. 13 countries for the year 1990 and 23 countries for the year 2011 (the estimates for all countries are shown in Figure S2). The figure illustrates that in 1990, for the majority of such countries, differences were due mainly to the inclusion of the data quality model, while for the 2011 estimates, both the data quality model as well as the curve fitting approach gave rise to differences, resulting in mostly decreases in the estimated U5MR. A subset of highlighted countries will be discussed to explain what properties of the new data and curve fitting model gave rise to the differences.

**Figure 7:**
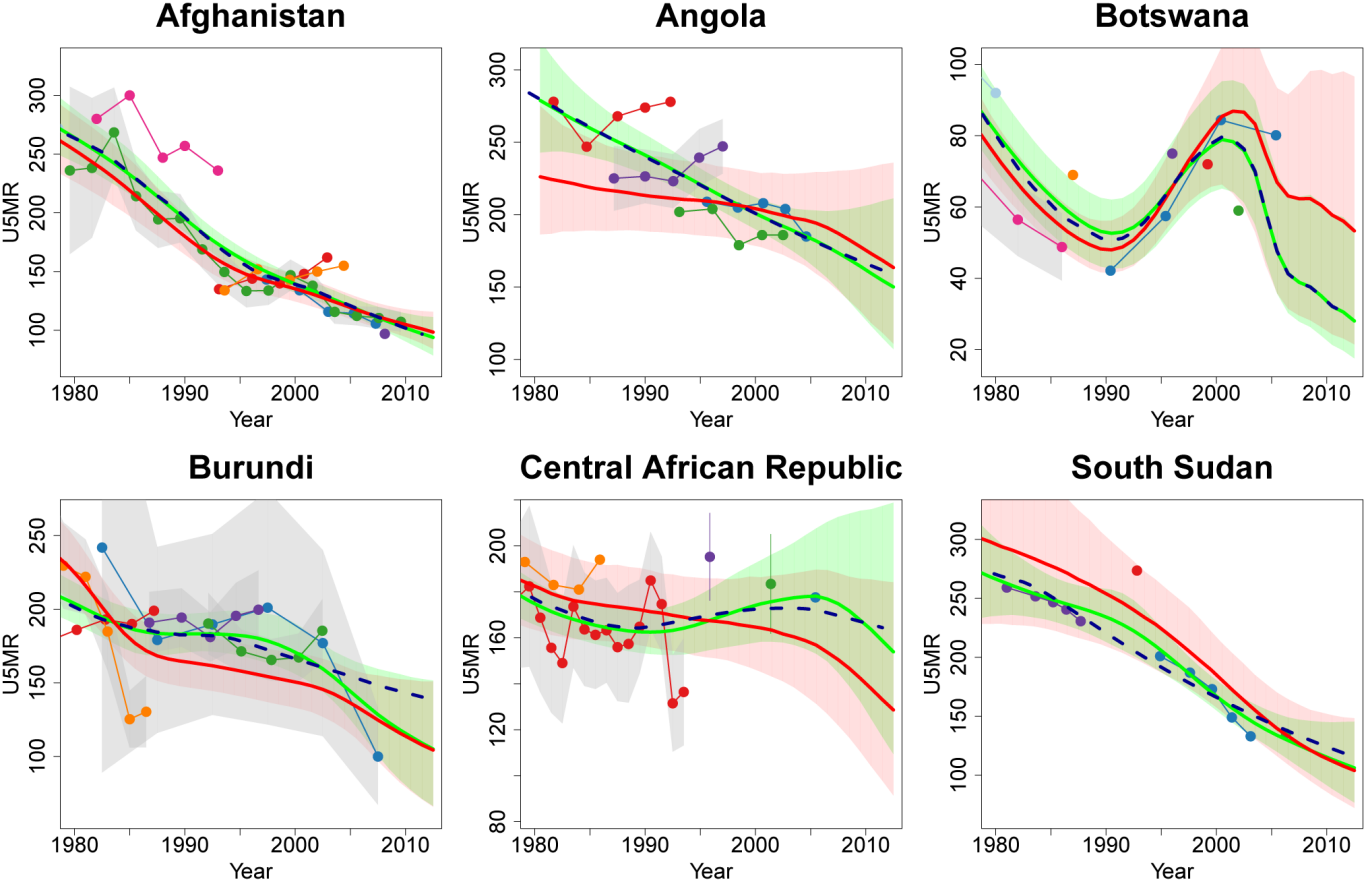
Comparison of U5MR estimates for Afghanistan, Angola, Botswana, Burundi, Central African Republic and South Sudan, where the inclusion of the data quality model changed the estimate by more than 10 deaths per 1,000 live births. Estimates compared are from UN IGME 2013 (solid red line with 90% credible intervals given by the shaded regions), B2 fit to 2013 database (solid light green line with 90% credible intervals given by the shaded regions), default Loess fit to 2013 database (dashed dark blue line). Connected dots denote data from the UN IGME 2013 database and gray shaded areas around series of observations represent the sampling variability in the series (quantified by two times of the sampling standard errors). Excluded data series and detailed information on all data series are displayed in Figure S1.

The inclusion of the data quality model in the B3 model has led to changes in various countries without VR data because of the estimation of biases for data series, while simultaneously “weighting” observations to account for the uncertainty associated with the observation related to biases as well as sampling and non-sampling errors, as illustrated for selected countries in Figure 7. Angola is the country with the largest difference between UN IGME 2012 and 2013 estimates for 1990 caused by the difference in the data quality model. In Angola, data series are quite spread out and each individual series does not suggest much decline in U5MR. In the B3 model, generally slope biases are estimated to be less variable than level biases, which can result in B3 estimates that follow more closely the within-series trend as opposed to the series level. This is illustrated in Angola, where considerable positive level biases are estimated for the earliest two series (shown in red and purple), resulting in U5MR estimates around 1990 that are below the two series. These estimates contrast with the Loess estimates which pass through both series and are hence higher.

In 2011, Central African Republic is one of the countries with the largest difference due to the inclusion of the data quality model. The main reason is the contradictory information between the data from the DHS 1994–1995 (displayed in red) and the data from Multiple Indicator Cluster Survey (MICS) 2000 (the purple data point). Because of the low likelihood of large changes in U5MR in a short period, the most recent data points of the DHS 1994–1995 are estimated to be biased downwards, while the data point from MICS 2000 is estimated to be biased upwards, resulting in lower U5MR estimates.

The simultaneous accounting for biases and weighting of observations in B3, as opposed to having all observations treated equally, accounts for the increase in the 1990 estimate for South Sudan; the single observation (in red) based on household deaths in 1992, which is much higher than the other available data series, now carries more weight. Similarly, in Afghanistan in the 1980s, the B3 estimates are closer to the DHS series (in green) as compared to the much higher MICS series (in pink), resulting in a lower U5MR estimate for 1990.

The lower estimates for Burundi are due to a greater estimated decline in the 1980s (the trend in the B3 estimates is informed by the declines observed within the two DHS series (in blue and orange), but may also be caused by the most recent observations in both (DHS) series (see Discussion). Finally, for Botswana, recent estimates have changed because of the observed increase in the most recent data series (in blue), as well as changes in the projected AIDS trend in the U5MR projections.

**Figure 8:**
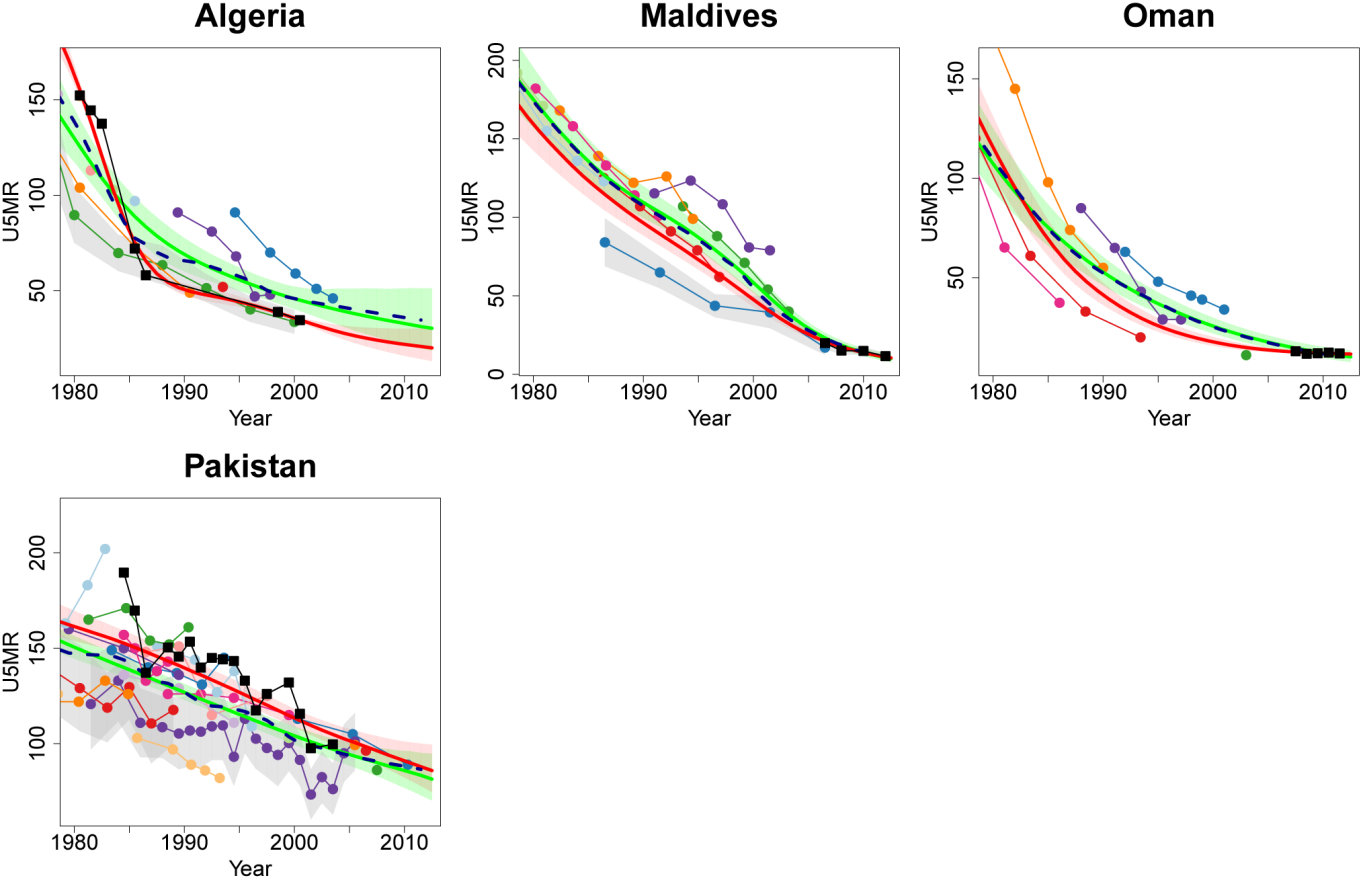
Comparison of U5MR estimates for Algeria, Maldives, Oman and Pakistan, where the inclusion of the data quality model resulted in estimates that are closer to VR data. Estimates compared are from UN IGME 2013 (solid red line with 90% credible intervals given by the shaded regions), B2 fit to 2013 database (solid light green line with 90% credible intervals given by the shaded regions), default Loess fit to 2013 database (dashed dark blue line). Connected dots denote data from the UN IGME 2013 database and gray shaded areas around series of observations represent the sampling variability in the series (quantified by two times of the sampling standard errors). VR data is denoted by connected black squares. Excluded data series and detailed information on all data series are displayed in Figure S1.

For countries with VR data (including VR, sample VR, and other registration data), the data quality model assumes that such data are unbiased, and associated stochastic errors are generally small. Consequently, in countries with VR data, the level and trend of the B3 estimates tend to be dictated by such VR data, as in the case of Algeria, Maldives, Oman and Pakistan (see Figure 8). Resulting UN IGME 2013 estimates differ from the UN IGME 2012 estimates, where VR and non-VR observations were weighted equally. The new data quality model induced the added advantage of U5MR estimates not crossing through a VR series (as observed in the UN IGME 2012 estimates for Oman) – VR observations are more likely to be underreported than not, so estimates that fall under observed values from a VR system are deemed unrealistic. Note that for Oman, the inclusion of the data quality model for non-VR data also resulted in a different trend estimate for earlier years: the U5MR estimates follow the within-series trends more closely.

**Figure 9:**
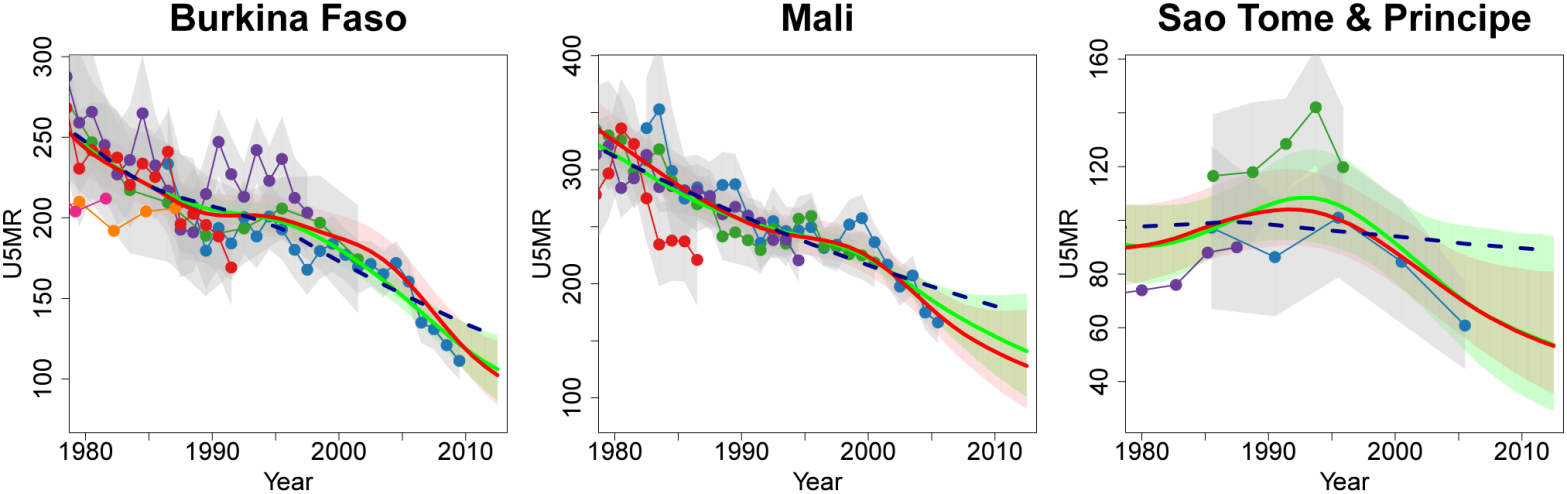
Comparison of U5MR estimates for Burkina Faso, Mali and Sao Tome and Principe where the change in curve fitting method changed the estimate by more than 10 deaths per 1,000 live births. Estimates compared are from UN IGME 2013 (solid red line with 90% credible intervals given by the shaded regions), B2 fit to 2013 database (solid light green line with 90% credible intervals given by the shaded regions), default Loess fit to 2013 database (dashed dark blue line). Connected dots denote data from the UN IGME 2013 database and gray shaded areas around series of observations represent the sampling variability in the series (quantified by two times of the sampling standard errors). Excluded data series and detailed information on all data series are displayed in Figure S1.

The comparison between the default Loess curve fitting approach and the B-spline method shows that the B-spline method results in fits that can follow recent trends in the data more closely. This results in lower estimates for countries where more recent data series show a decline in U5MR and explains the differences due to the curve fitting method for the majority of countries in 2011, e.g., in Burkina Faso, Burundi, Guinea-Bissau, Mali and Sao Tome & Principe; see Figure 9 for examples.

### Decomposition of differences in under-five deaths

**Figure 10:**
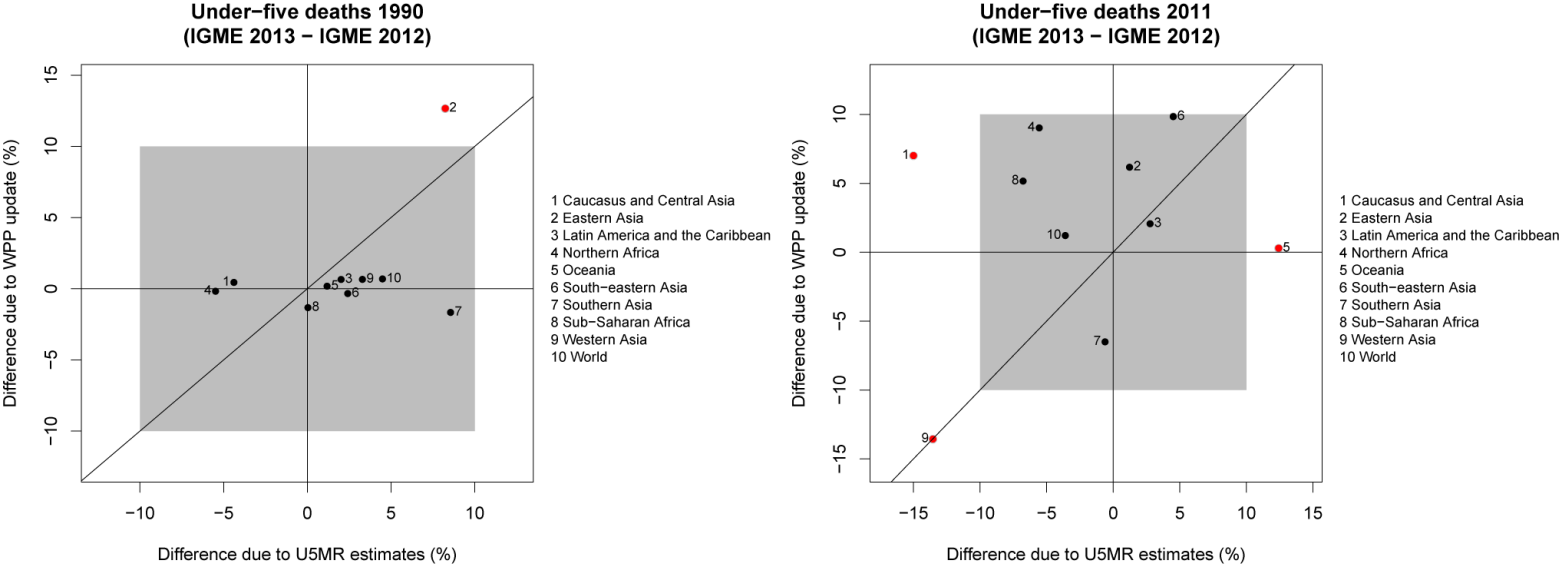
Decomposition of differences in under-five deaths in 1990 and 2011 into differences due to the WPP update and differences due to updates in U5MR estimates. The gray box represents differences up to 10%. Regions with differences of more than 10% due to either factor are highlighted in red.

For the number of under-five deaths in 1990, there was a more than 10% difference due to the WPP update for Eastern Asia and less than 10% for the world and other regions (see Figure 10). Differences due to the change in U5MR estimates were less than 10% for the world and all regions. For the U5MR in 2011, there was a more than 10% difference due to the WPP update only for Western Asia. The difference due to the change in U5MR estimates was more than 10% for Western Asia as well, and also for Caucasus and Central Asia and Oceania.

## Discussion

The estimation of child mortality is challenging for the great majority of developing countries without well-functioning VR systems due to issues with data quality (and for some countries, data quantity), and models are required to construct U5MR estimates for years of interest. In 2013, the UN IGME published new estimates of the U5MR for all countries based on a new estimation method, referred to as the B3 method [4]. In this paper, we summarized differences between the databases and modelling approaches used for the UN IGME 2013 and UN IGME 2012 estimates and analysed the effect of the change in methods on the resulting U5MR estimates through a decomposition exercise.

Global and regional estimates of the U5MR in 1990 and 2011 changed little (less than 10%) from the UN IGME 2012 version to the UN IGME 2013 version. Point estimates of the U5MR changed by more than 10% for 19 countries in 1990 and for 33 countries in 2011. For 1990, the decomposition exercise showed that differences were mainly due to the inclusion of the extended data quality model in the B3 estimation method. For the year 2011, both new data as well the extended data quality model in the B3 estimation method and the B-spline fitting method explained the differences between UN IGME 2012 and 2013 estimates.

The inclusion of the B3 data quality model, which accounts for various properties of the errors that provide information about the quality of the observation, resulted in changes in U5MR estimates in 1990 and 2011 for various reasons. Firstly, the new data quality model allowed for level and trend biases in data series. If, in a country, U5MR data series partially overlap in time but suggest very different U5MR levels and/or trends, such series are deemed to be subject to level and/or trend biases, which are accounted for when estimating the U5MR and can result in different estimates compared to treating all observations equally. Slope biases were estimated to be less variable than level biases and as a result, B3 estimates were found to follow more closely the within-series trend as opposed to the series level. In addition to the estimation of biases for data series, “weighting” of observations to account for the uncertainty associated with the observation related to biases as well as sampling and non-sampling errors is simultaneously performed, and can result in changes in estimates, as discussed for Angola, Central African Republic, South Sudan, Afghanistan, Burundi and Botswana. Lastly, UN IGME 2013 estimates tend to be closer to VR data, if available, such as in Oman, Algeria, Maldives and Pakistan. The B-spline method resulted in lower estimates for countries where more recent data series show a decline in U5MR (e.g., in Burkina Faso, Burundi, Guinea-Bissau, Mali and Sao Tome & Principe) because it can follow recent trends in the data more closely as compared to the UN IGME 2012 default Loess method.

With this paper, our aim was to motivate and explain changes in modeling assumptions and their effect on U5MR estimates and to be transparent about the construction of estimates to avoid confusion about changes in point estimates. We focused the decomposition on high mortality countries with absolute differences in estimates of more than 10 deaths per 1,000 live births, because of the great attention for the point estimates for these countries to assess progress towards MDG 4. For a subset of these countries, the change in point estimate is illustrative of the uncertainty in levels and trends of U5MR within a country, and the paucity of reliable data. It is therefore not surprising that we found more countries with differences in estimates for the year 2011 as compared to 1990, given the limited availability of data for more recent years for many countries. Quantification of uncertainty helps to explain differences in estimates. For example, we identified one country (Lao PDR) that was originally deemed to be on track for MDG 4 in UN IGME 2012 [2] but no longer considered so in UN IGME 2013 based on the point estimates for the ARR in U5MR [3]. However, when accounting for uncertainty and categorizing countries’ progress based on the upper and lower bounds of the uncertainty intervals for the ARR, we find that the classification for Lao PDR has not changed from UN IGME 2012 [27] to 2013 [28]: based on either set of uncertainty intervals it is unclear whether it has achieved progress at an ARR of 4.4% or above. Therefore, to avoid inaccurate conclusions based on point estimates alone, we repeat our call for the inclusion of the uncertainty assessment into measurement of progress towards MDG 4 and progress in reducing the U5MR in general [4], [12], [29].

Going forward, child mortality estimates will still be subject to continuous updating because of extensions of databases and refinement of estimation methods. While the B3 approach provided important advantages to the previously used UN IGME approach, room for improvement remains. One particular area for future research is related to data quality issues; currently differences in data quality across data series are accounted for but rely on the assumption that biases are comparable across data series of the same source type. Thus far, a residual analysis in which (absolute) residuals were plotted against a number of data quality predictors (region that country belongs to, series source type, series year, observation year, retrospective period, level of U5MR in observation year, total fertility rate in the series year, and change in the total fertility rate in the last 15 years before the series) has not revealed substantial differences in the biases for different values of the data quality predictors, except for a possible downwards bias in recent observations from DHS data series which may be due to birth transference [30]. In future research we plan to explore whether these effects can be modelled more explicitly. This may help to identify for countries such as Burundi (Figure 7) whether recent observations from DHS series are biased downwards. Additionally, ideally external information on data quality would be incorporated to make a distinction between series of differing levels of reliability. This may help to assess whether the default bias adjustments in countries such as Central African Republic (Figure 7) are justified or need to be re-assessed. Ultimately, we hope that greater transparency of estimation methods, better communication of these methods and the challenges involved in constructing estimates for all countries that are comparable across countries, as well as revisions of estimates where necessary, will lead to increased engagement of and dialogue among all stakeholders, from those responsible for data collection and processing to those in charge of making policies and allocating funding. While the real-time monitoring of child mortality remains unattainable in the near future, every effort made brings us one step closer to that goal.

### Statement

The views expressed in this paper are those of the authors and do not necessarily reflect the views of the United Nations Children’s Fund nor those of other members of the UN Inter-agency Group for Child Mortality Estimation.

## Supporting Information

Figure S1 **Comparison of UN IGME 2013 U5MR estimates to UN IGME 2012 U5MR estimates for all countries.** UN IGME 2013 estimates are given by the solid red line (with 90% uncertainty intervals denoted by the shaded regions) while UN IGME 2012 estimates are given by the solid dark blue line and the default Loess fit to the 2013 UN IGME database is given by the dashed dark blue line. Connected dots denote data from the UN IGME 2013 database and gray shaded areas around series of observations represent the sampling variability in the series (quantified by two times of the sampling standard errors).
(PDF)

Figure S2 **U5MR estimates for 33 countries where the difference in estimation method between UN IGME 2012 and UN IGME 2013 led to an absolute difference in U5MR estimate for the year 1990 or 2011 of more than 10 deaths per 1,000 births.** The default loess fit to the 2013 UN IGME database is given by the dashed dark blue line, the B2 method (B3 method for curve fitting but treating all observations equally) fit is shown by the solid light green line (with 90% credible intervals given by the shaded regions) and UN IGME 2013 estimates are given in red by the solid red line (with 90% uncertainty intervals denoted by the shaded regions). Connected dots denote data from the UN IGME 2013 database and gray shaded areas around series of observations represent the sampling variability in the series (quantified by two times of the sampling standard errors).
(PDF)
