# Supplementary material for: Child Mortality Estimation 2013: An Overview of Updates in Estimation Methods by the United Nations Inter-Agency Group for Child Mortality Estimation
Source: PLoS One. 2014 Jul 11;9(7):e101112. doi: 10.1371/journal.pone.0101112 (PMC4094389; doi:10.1371/journal.pone.0101112)
Supplement: Figure S1 — Comparison of UN IGME 2013 U5MR estimates to UN IGME 2012 U5MR estimates for all countries. UN IGME 2013 estimates are given by the solid red line (with 90% uncertainty intervals denoted by the shaded regions) while UN IGME 2012 estimates are given by the solid dark blue line and the default Loess fit to the 2013 UN IGME database is given by the dashed dark blue line. Connected dots denote data from the UN IGME 2013 database and gray shaded areas around series of observations represent the sampling variability in the series (quantified by two times of the sampling standard errors). (PDF) [file pone.0101112.s001.pdf]

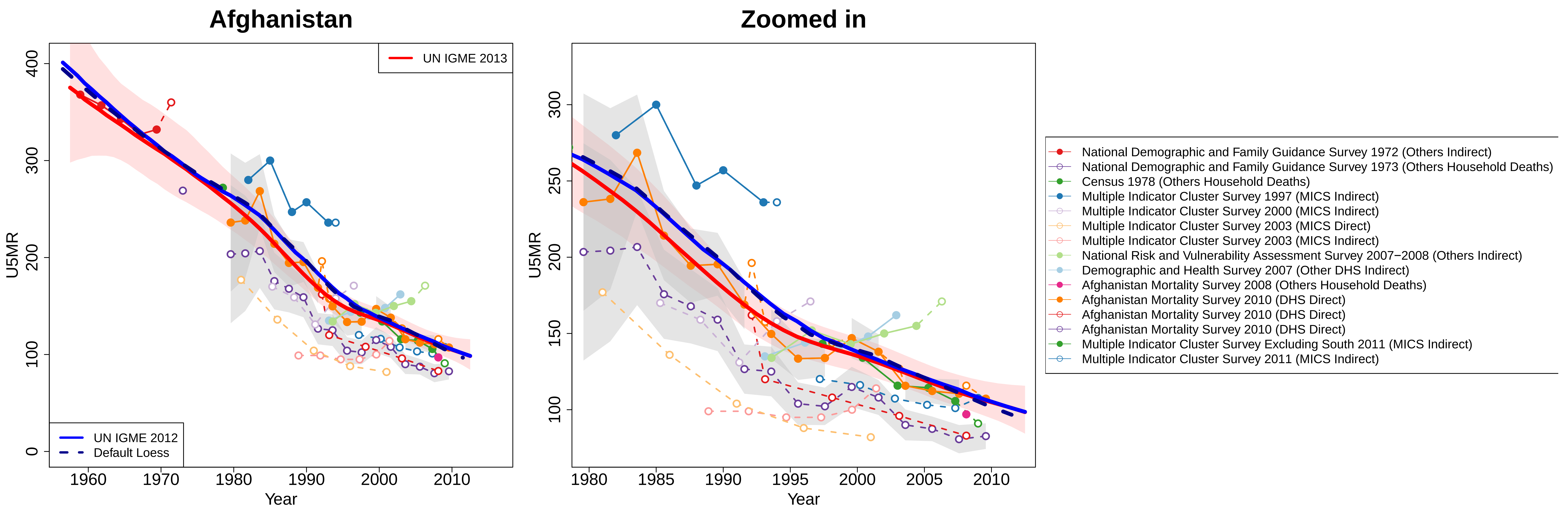

Afghanistan
Zoomed in
U5MR
Year
UN IGME 2013
UN IGME 2012
Default Loess
0
100
200
300
400
150
250
1960
1970
1980
1985
1990
1995
2000
2005
2010
National Demographic and Family Guidance Survey 1972 (Others Indirect)
National Demographic and Family Guidance Survey 1973 (Others Household Deaths)
Census 1978 (Others Household Deaths)
Multiple Indicator Cluster Survey 1997 (MICS Indirect)
Multiple Indicator Cluster Survey 2000 (MICS Indirect)
Multiple Indicator Cluster Survey 2003 (MICS Direct)
Multiple Indicator Cluster Survey 2003 (MICS Indirect)
National Risk and Vulnerability Assessment Survey 2007−2008 (Others Indirect)
Demographic and Health Survey 2007 (Other DHS Indirect)
Afghanistan Mortality Survey 2008 (Others Household Deaths)
Afghanistan Mortality Survey 2010 (DHS Direct)
Afghanistan Mortality Survey 2010 (DHS Direct)
Afghanistan Mortality Survey 2010 (DHS Direct)
Multiple Indicator Cluster Survey Excluding South 2011 (MICS Indirect)
Multiple Indicator Cluster Survey 2011 (MICS Indirect)

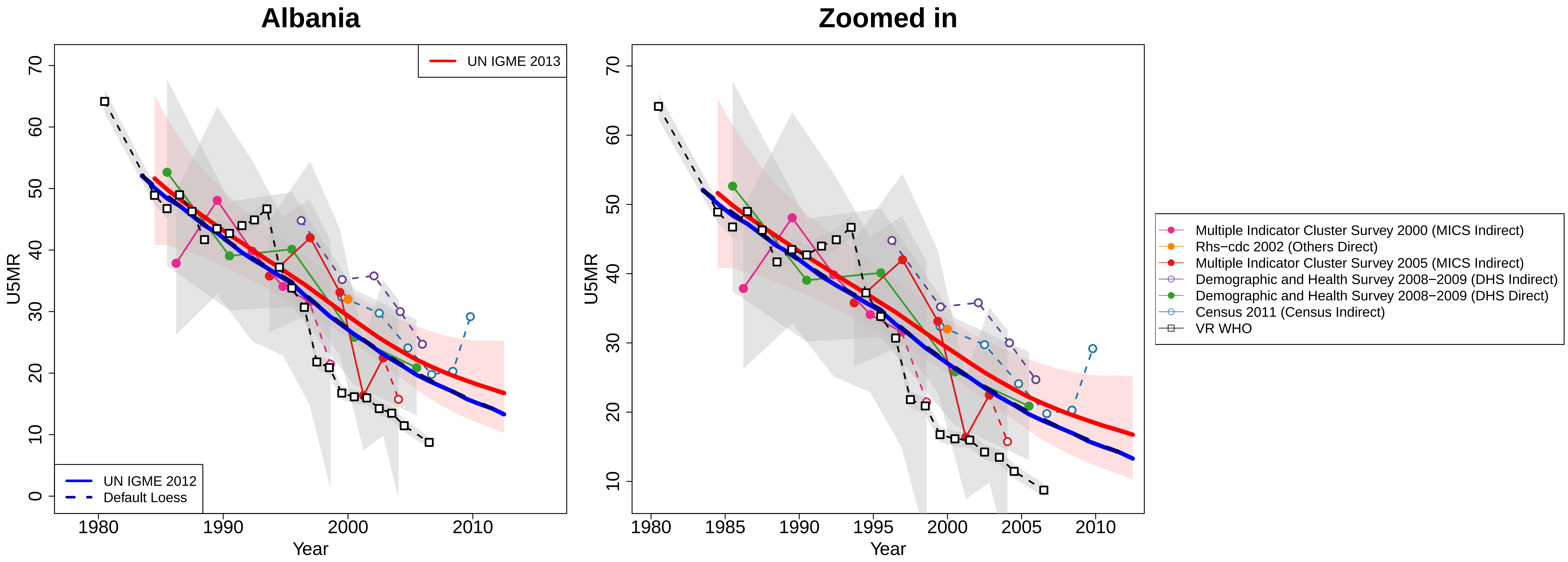
Albania
Zoomed in
UN IGME 2013
UN IGME 2012
Default Loess
U5MR
Year
Multiple Indicator Cluster Survey 2000 (MICS Indirect)
Rhs−cdc 2002 (Others Direct)
Multiple Indicator Cluster Survey 2005 (MICS Indirect)
Demographic and Health Survey 2008−2009 (DHS Indirect)
Demographic and Health Survey 2008−2009 (DHS Direct)
Census 2011 (Census Indirect)
VR WHO

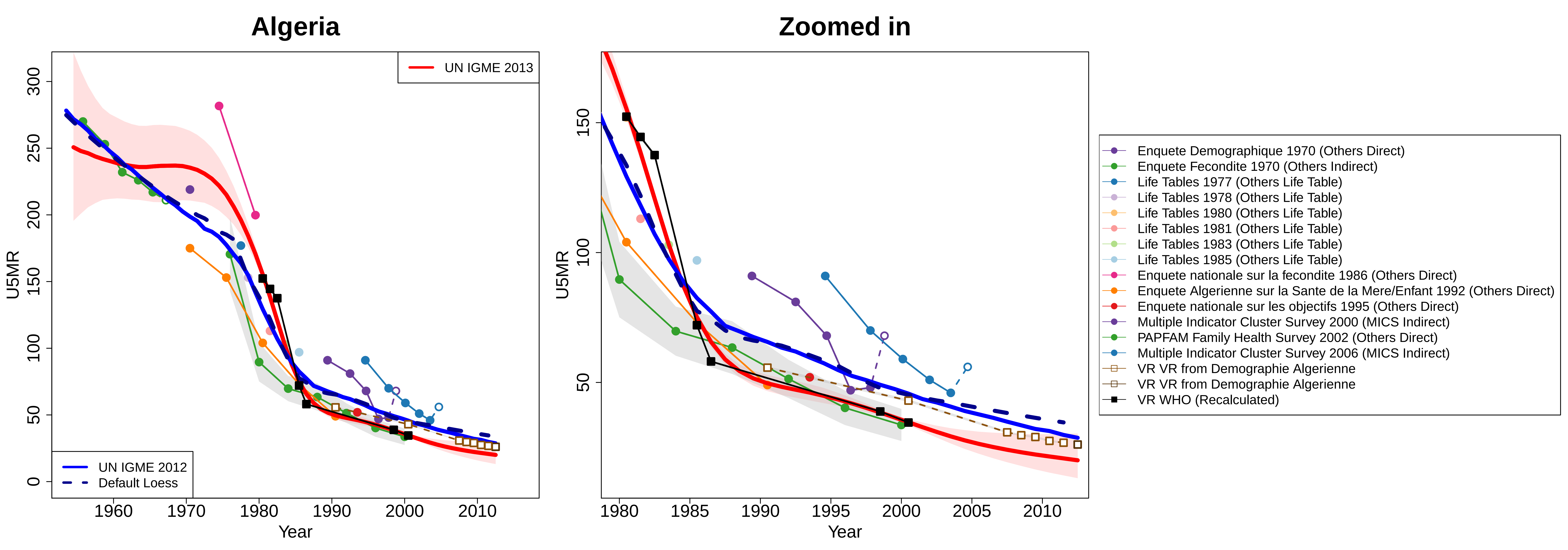

Algeria
Zoomed in
UN IGME 2013
UN IGME 2012
Default Loess
U5MR
Year
Enquete Demographique 1970 (Others Direct)
Enquete Fecondite 1970 (Others Indirect)
Life Tables 1977 (Others Life Table)
Life Tables 1978 (Others Life Table)
Life Tables 1980 (Others Life Table)
Life Tables 1981 (Others Life Table)
Life Tables 1983 (Others Life Table)
Life Tables 1985 (Others Life Table)
Enquete nationale sur la fecondite 1986 (Others Direct)
Enquete Algerienne sur la Sante de la Mere/Enfant 1992 (Others Direct)
Enquete nationale sur les objectifs 1995 (Others Direct)
Multiple Indicator Cluster Survey 2000 (MICS Indirect)
PAPFAM Family Health Survey 2002 (Others Direct)
Multiple Indicator Cluster Survey 2006 (MICS Indirect)
VR VR from Demographie Algerienne
VR VR from Demographie Algerienne
VR WHO (Recalculated)

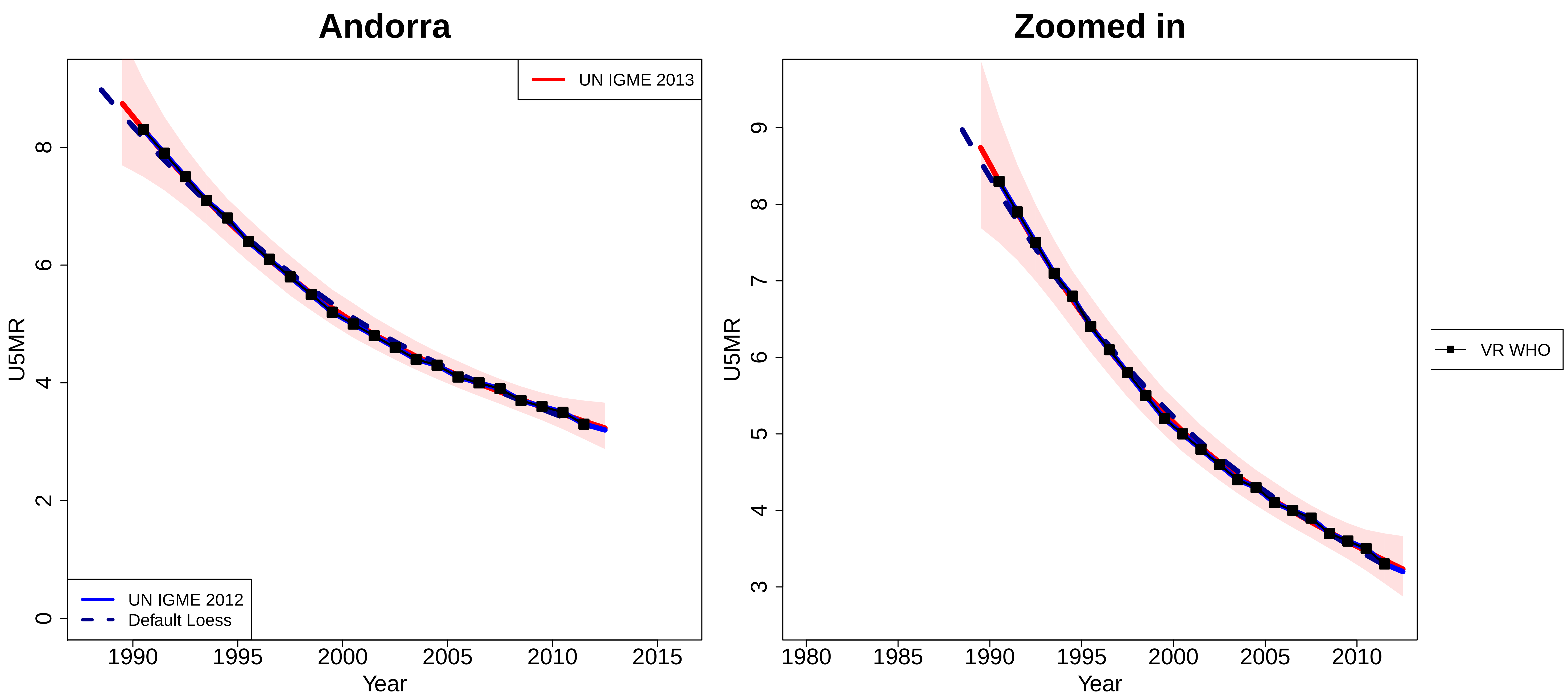

Andorra
UN IGME 2013
UN IGME 2012
Default Loess
U5MR
Year
0
2
4
6
8
1990
1995
2000
2005
2010
2015
Zoomed in
VR WHO
3
4
5
6
7
8
9
1980
1985

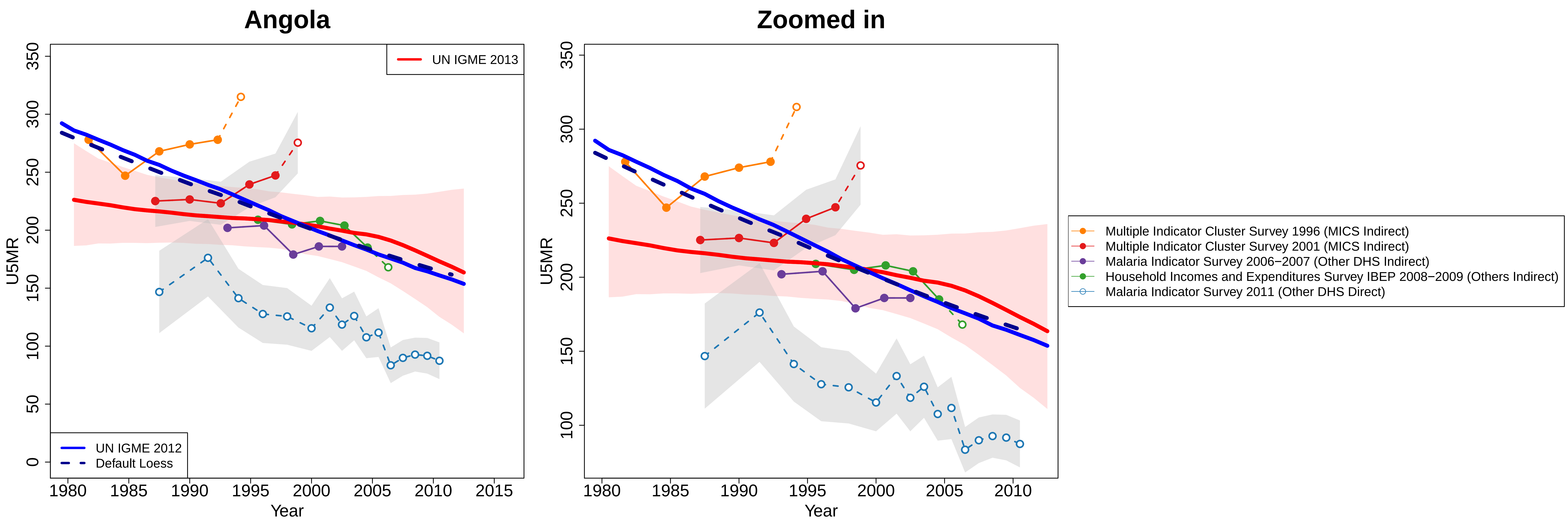

Angola
Zoomed in
U5MR
Year
UN IGME 2013
UN IGME 2012
Default Loess
Multiple Indicator Cluster Survey 1996 (MICS Indirect)
Multiple Indicator Cluster Survey 2001 (MICS Indirect)
Malaria Indicator Survey 2006−2007 (Other DHS Indirect)
Household Incomes and Expenditures Survey IBEP 2008−2009 (Others Indirect)
Malaria Indicator Survey 2011 (Other DHS Direct)

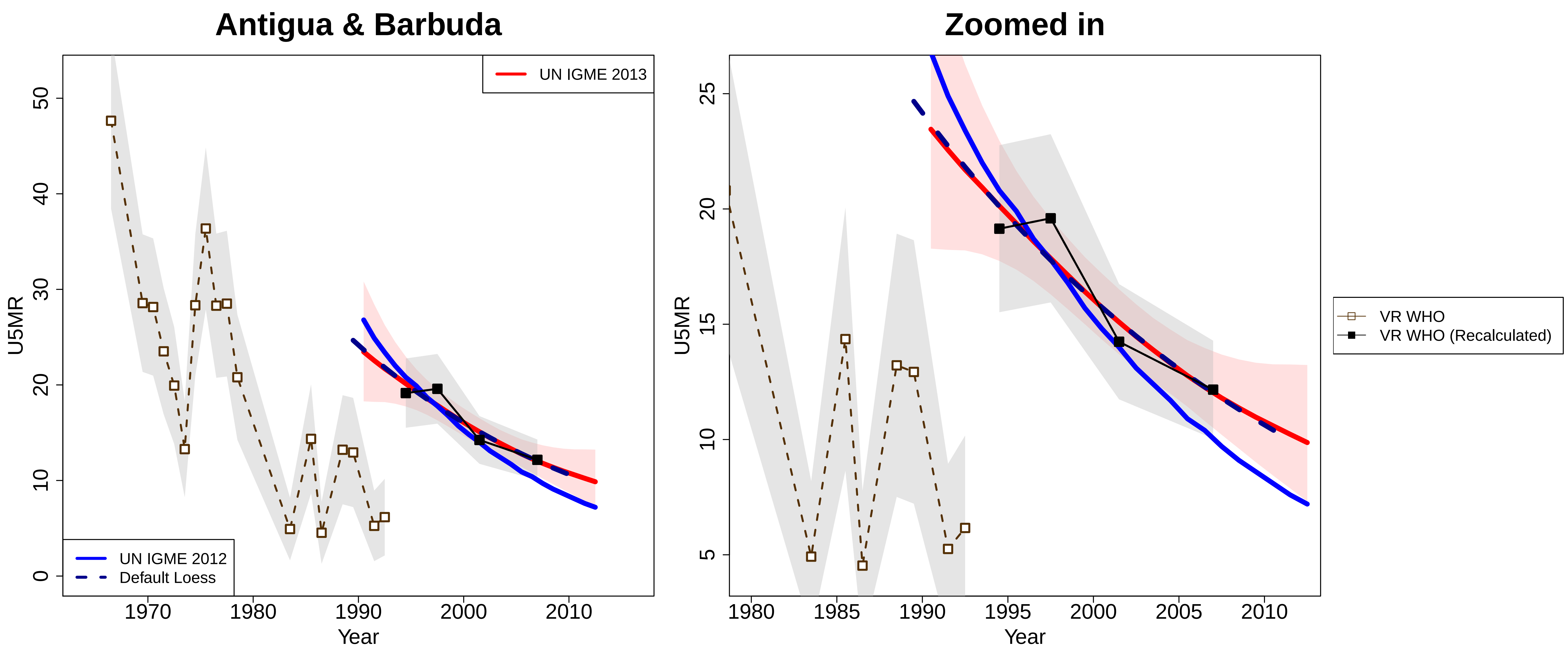

Antigua & Barbuda
UN IGME 2013
UN IGME 2012
Default Loess
U5MR
Year
Zoomed in
VR WHO
VR WHO (Recalculated)

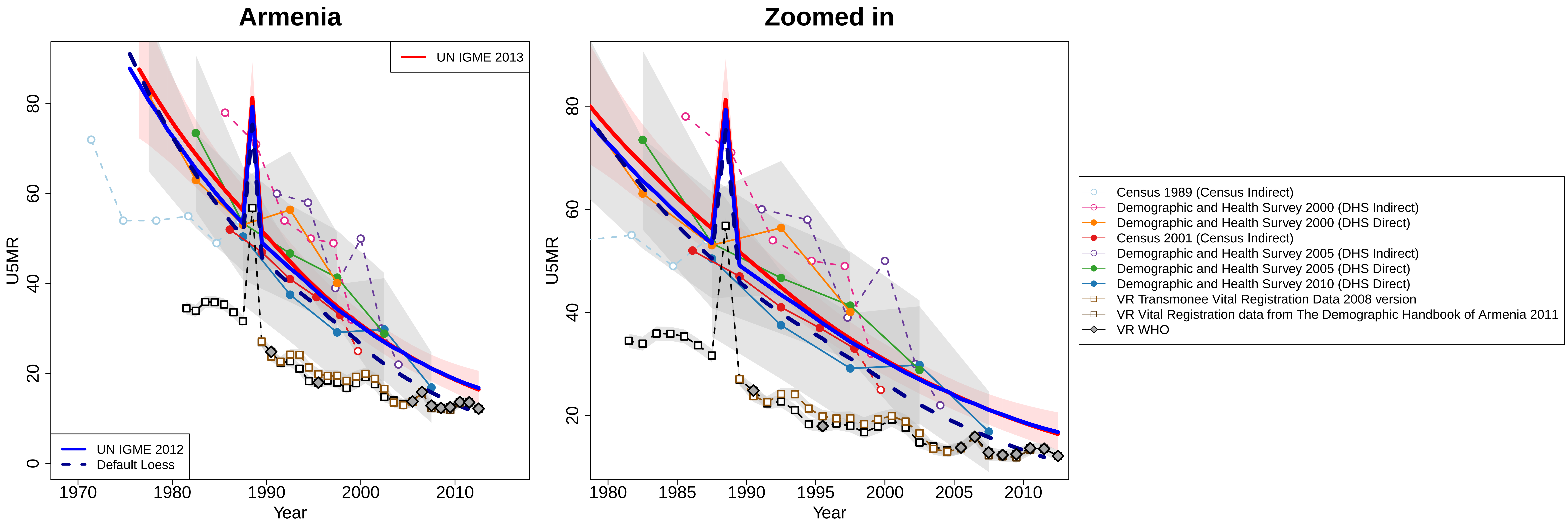

Armenia
UN IGME 2013
UN IGME 2012
Default Loess
U5MR
Year
Zoomed in
U5MR
Year
Census 1989 (Census Indirect)
Demographic and Health Survey 2000 (DHS Indirect)
Demographic and Health Survey 2000 (DHS Direct)
Census 2001 (Census Indirect)
Demographic and Health Survey 2005 (DHS Indirect)
Demographic and Health Survey 2005 (DHS Direct)
Demographic and Health Survey 2010 (DHS Direct)
VR Transmonee Vital Registration Data 2008 version
VR Vital Registration data from The Demographic Handbook of Armenia 2011
VR WHO

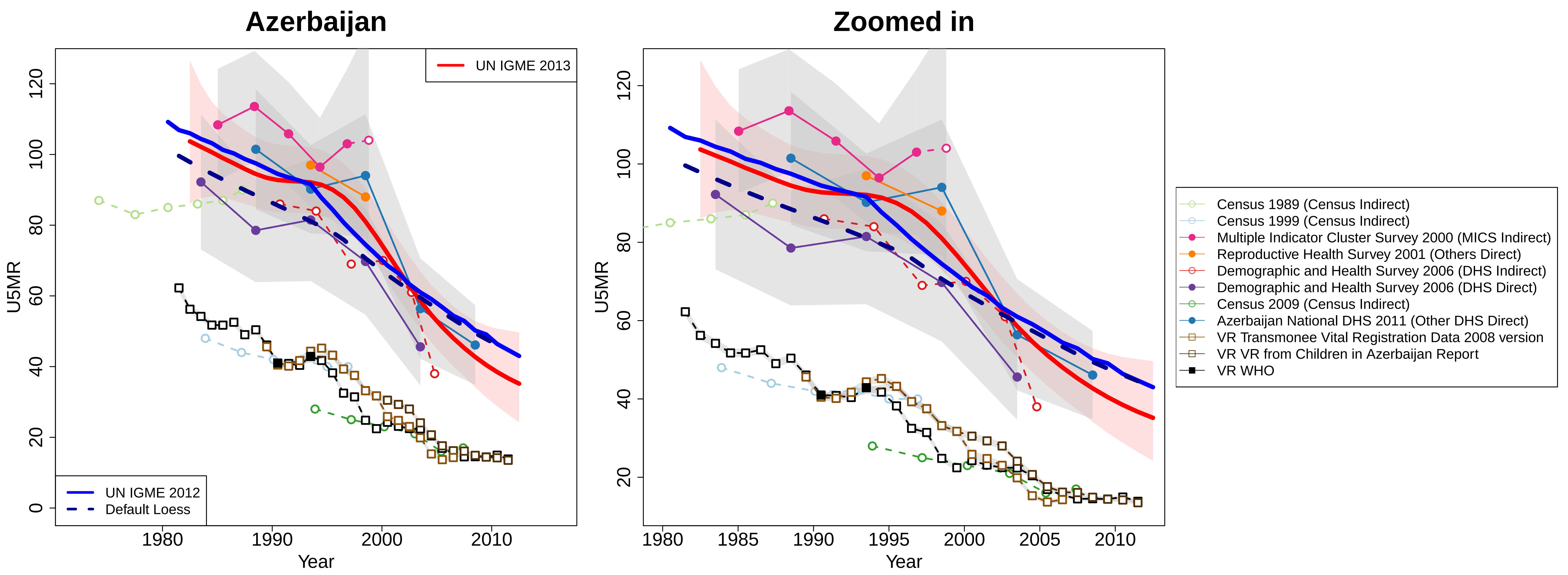
Azerbaijan
Zoomed in
UN IGME 2013
UN IGME 2012
Default Loess
U5MR
Year
Census 1989 (Census Indirect)
Census 1999 (Census Indirect)
Multiple Indicator Cluster Survey 2000 (MICS Indirect)
Reproductive Health Survey 2001 (Others Direct)
Demographic and Health Survey 2006 (DHS Indirect)
Demographic and Health Survey 2006 (DHS Direct)
Census 2009 (Census Indirect)
Azerbaijan National DHS 2011 (Other DHS Direct)
VR Transmonee Vital Registration Data 2008 version
VR VR from Children in Azerbaijan Report
VR WHO

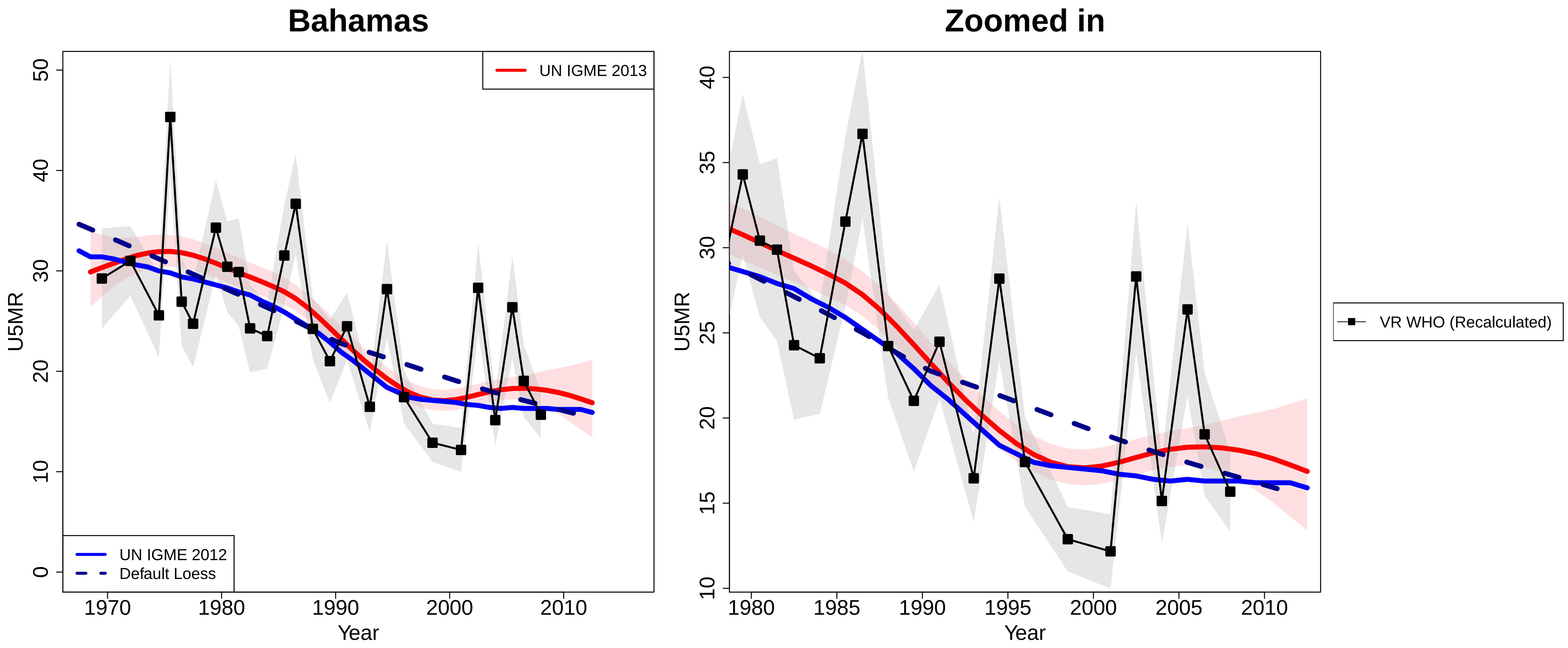

Bahamas
Zoomed in
UN IGME 2013
UN IGME 2012
Default Loess
VR WHO (Recalculated)
U5MR
Year

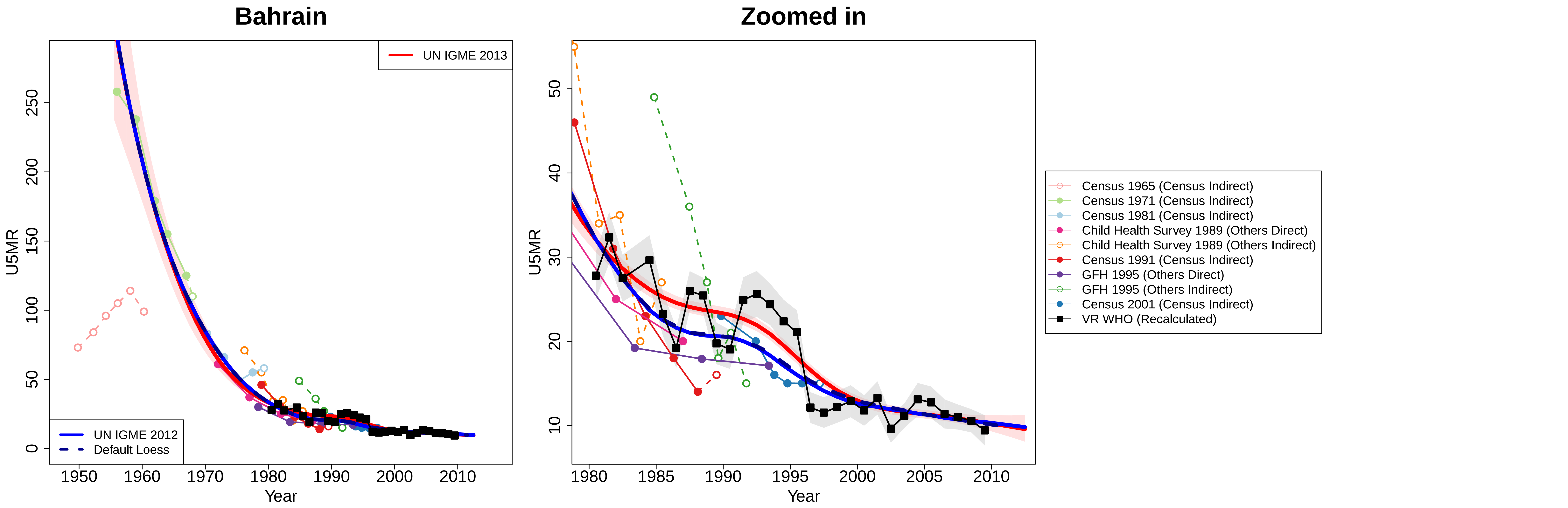

Bahrain
Zoomed in
U5MR
Year
UN IGME 2013
UN IGME 2012
Default Loess
Census 1965 (Census Indirect)
Census 1971 (Census Indirect)
Census 1981 (Census Indirect)
Child Health Survey 1989 (Others Direct)
Child Health Survey 1989 (Others Indirect)
Census 1991 (Census Indirect)
GFH 1995 (Others Direct)
GFH 1995 (Others Indirect)
Census 2001 (Census Indirect)
VR WHO (Recalculated)

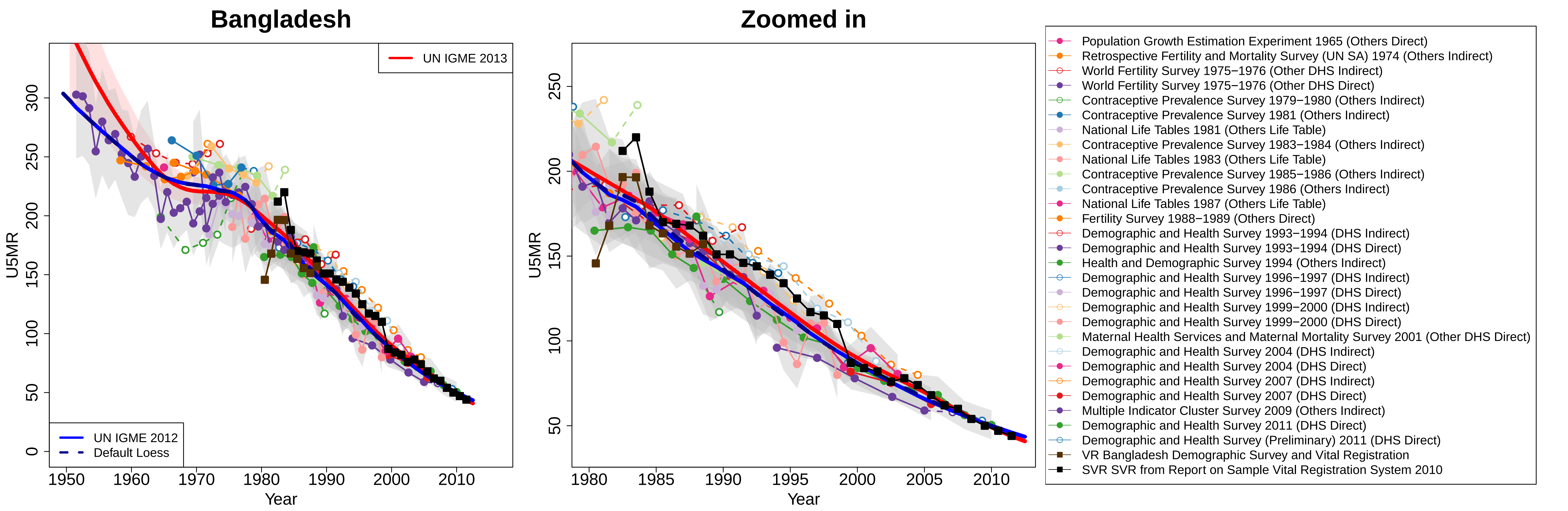

Bangladesh
Zoomed in
UN IGME 2013
UN IGME 2012
Default Loess
U5MR
Year
Population Growth Estimation Experiment 1965 (Others Direct)
Retrospective Fertility and Mortality Survey (UN SA) 1974 (Others Indirect)
World Fertility Survey 1975−1976 (Other DHS Indirect)
World Fertility Survey 1975−1976 (Other DHS Direct)
Contraceptive Prevalence Survey 1979−1980 (Others Indirect)
Contraceptive Prevalence Survey 1981 (Others Indirect)
National Life Tables 1981 (Others Life Table)
Contraceptive Prevalence Survey 1983−1984 (Others Indirect)
National Life Tables 1983 (Others Life Table)
Contraceptive Prevalence Survey 1985−1986 (Others Indirect)
Contraceptive Prevalence Survey 1986 (Others Indirect)
National Life Tables 1987 (Others Life Table)
Fertility Survey 1988−1989 (Others Direct)
Demographic and Health Survey 1993−1994 (DHS Indirect)
Demographic and Health Survey 1993−1994 (DHS Direct)
Health and Demographic Survey 1994 (Others Indirect)
Demographic and Health Survey 1996−1997 (DHS Indirect)
Demographic and Health Survey 1996−1997 (DHS Direct)
Demographic and Health Survey 1999−2000 (DHS Indirect)
Demographic and Health Survey 1999−2000 (DHS Direct)
Maternal Health Services and Maternal Mortality Survey 2001 (Other DHS Direct)
Demographic and Health Survey 2004 (DHS Indirect)
Demographic and Health Survey 2004 (DHS Direct)
Demographic and Health Survey 2007 (DHS Indirect)
Demographic and Health Survey 2007 (DHS Direct)
Multiple Indicator Cluster Survey 2009 (Others Indirect)
Demographic and Health Survey 2011 (DHS Direct)
Demographic and Health Survey (Preliminary) 2011 (DHS Direct)
VR Bangladesh Demographic Survey and Vital Registration
SVR SVR from Report on Sample Vital Registration System 2010

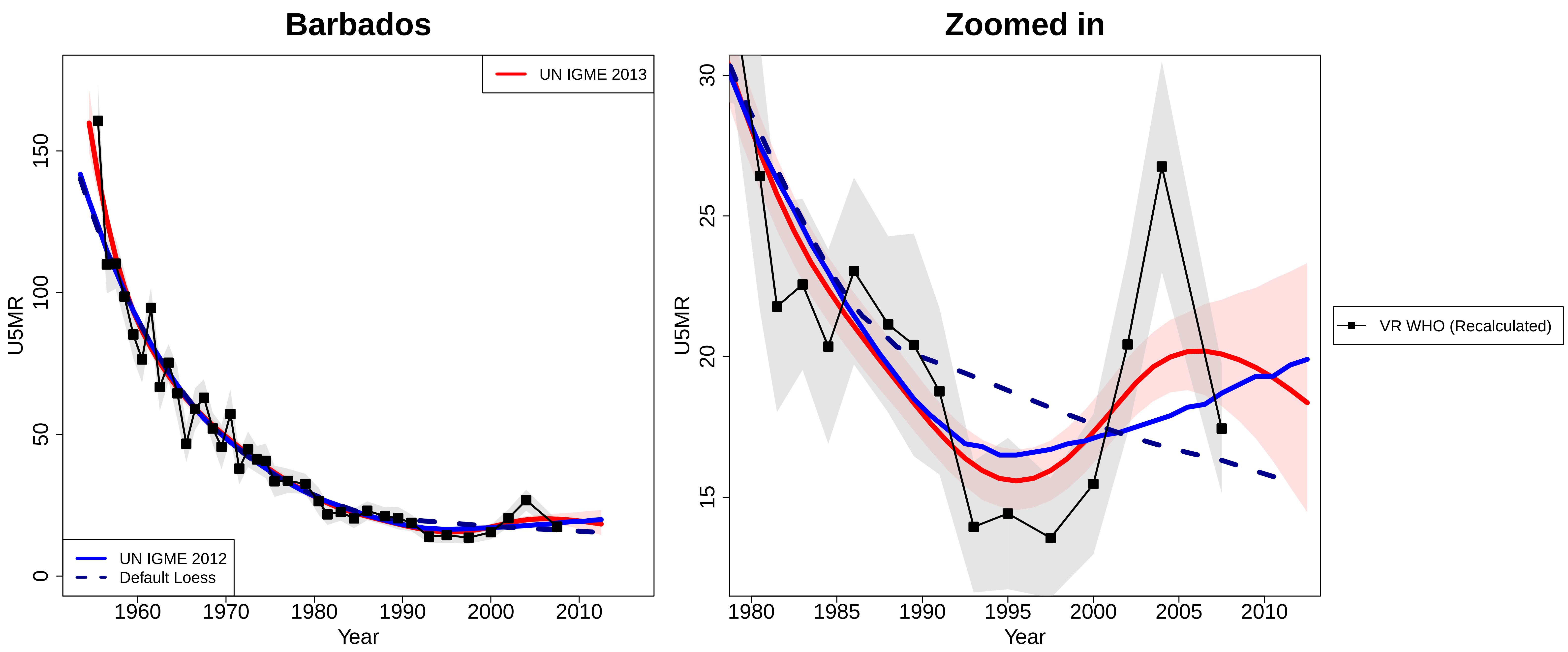

Barbados
Zoomed in
UN IGME 2013
UN IGME 2012
Default Loess
VR WHO (Recalculated)
U5MR
Year
0
50
100
150
1960
1970
1980
1990
2000
2010
15
20
25
30
1980
1985
1990
1995
2000
2005
2010

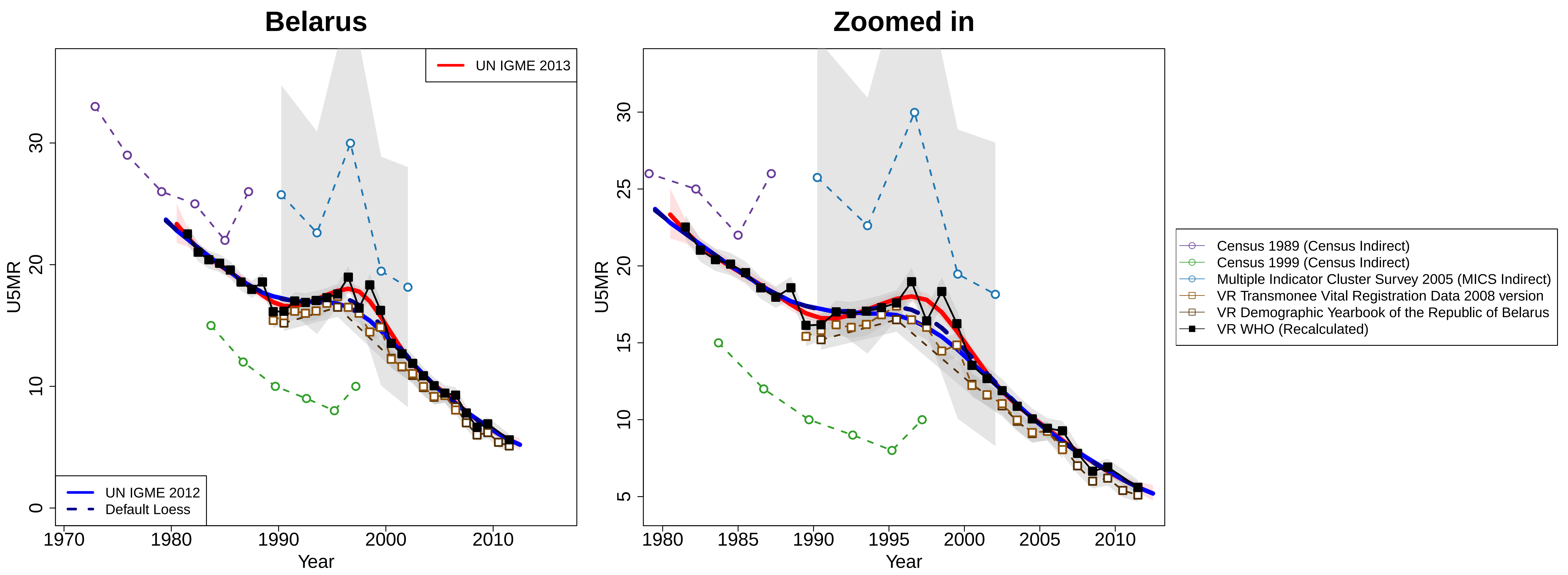

Belarus
Zoomed in
UN IGME 2013
UN IGME 2012
Default Loess
U5MR
Year
Census 1989 (Census Indirect)
Census 1999 (Census Indirect)
Multiple Indicator Cluster Survey 2005 (MICS Indirect)
VR Transmonee Vital Registration Data 2008 version
VR Demographic Yearbook of the Republic of Belarus
VR WHO (Recalculated)

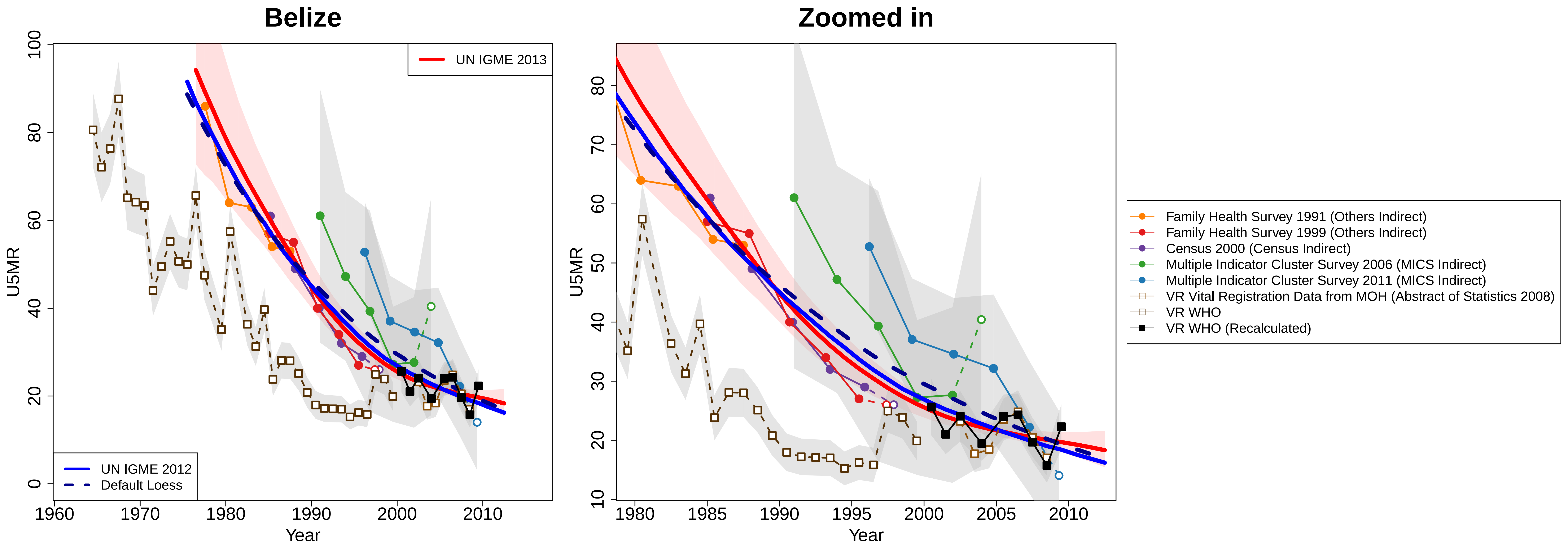

Belize
Zoomed in
UN IGME 2013
UN IGME 2012
Default Loess
U5MR
Year
Family Health Survey 1991 (Others Indirect)
Family Health Survey 1999 (Others Indirect)
Census 2000 (Census Indirect)
Multiple Indicator Cluster Survey 2006 (MICS Indirect)
Multiple Indicator Cluster Survey 2011 (MICS Indirect)
VR Vital Registration Data from MOH (Abstract of Statistics 2008)
VR WHO
VR WHO (Recalculated)

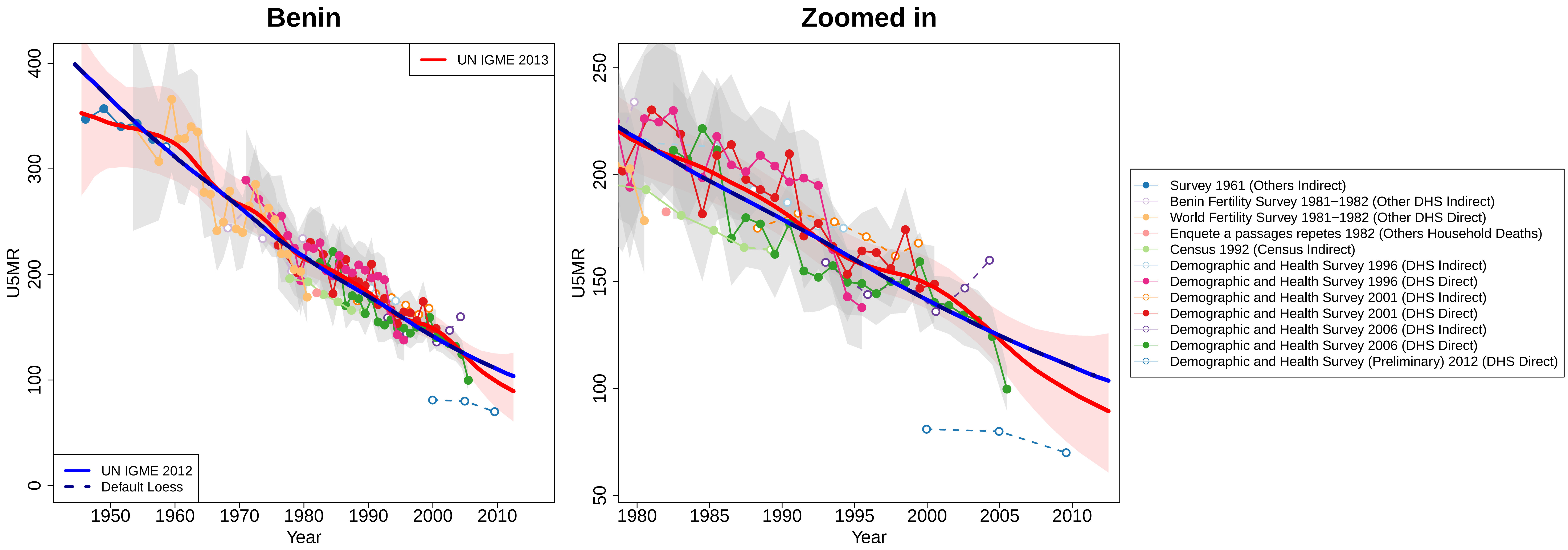
Benin
Zoomed in
UN IGME 2013
UN IGME 2012
Default Loess
U5MR
Year
Survey 1961 (Others Indirect)
Benin Fertility Survey 1981−1982 (Other DHS Indirect)
World Fertility Survey 1981−1982 (Other DHS Direct)
Enquete a passages repetes 1982 (Others Household Deaths)
Census 1992 (Census Indirect)
Demographic and Health Survey 1996 (DHS Indirect)
Demographic and Health Survey 1996 (DHS Direct)
Demographic and Health Survey 2001 (DHS Indirect)
Demographic and Health Survey 2001 (DHS Direct)
Demographic and Health Survey 2006 (DHS Indirect)
Demographic and Health Survey 2006 (DHS Direct)
Demographic and Health Survey (Preliminary) 2012 (DHS Direct)

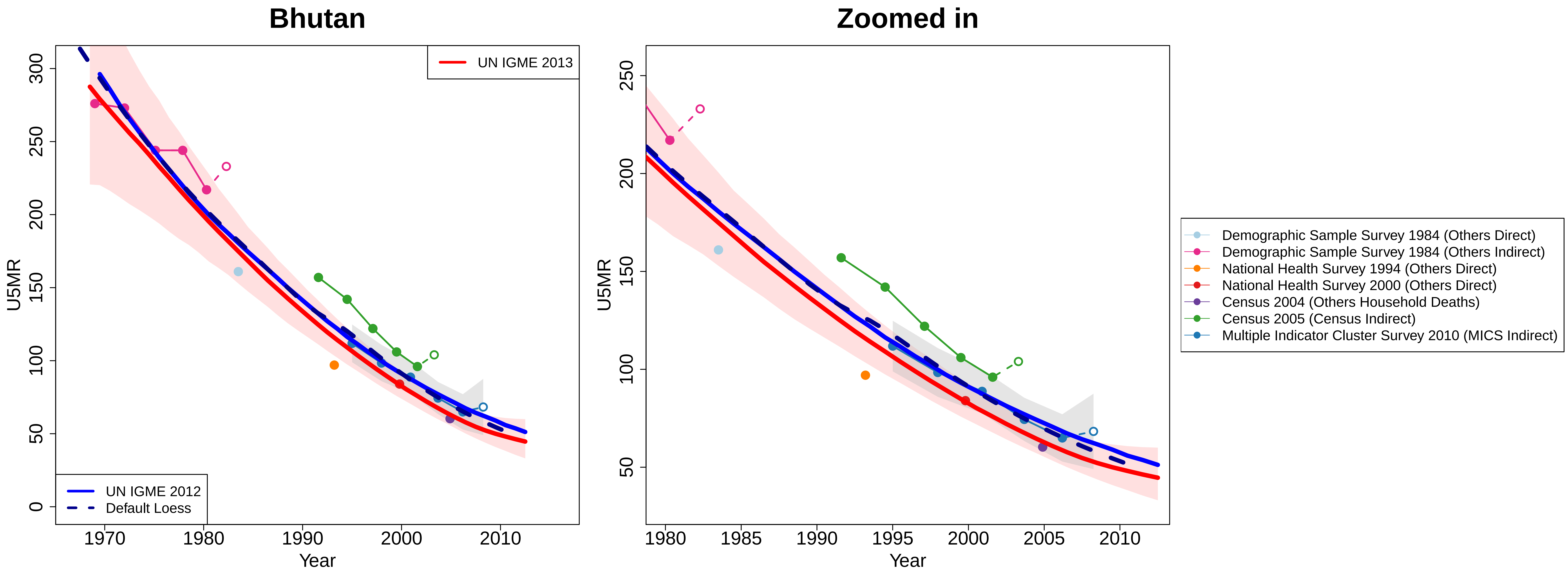
Bhutan
Zoomed in
UN IGME 2013
UN IGME 2012
Default Loess
U5MR
Year
Demographic Sample Survey 1984 (Others Direct)
Demographic Sample Survey 1984 (Others Indirect)
National Health Survey 1994 (Others Direct)
National Health Survey 2000 (Others Direct)
Census 2004 (Others Household Deaths)
Census 2005 (Census Indirect)
Multiple Indicator Cluster Survey 2010 (MICS Indirect)

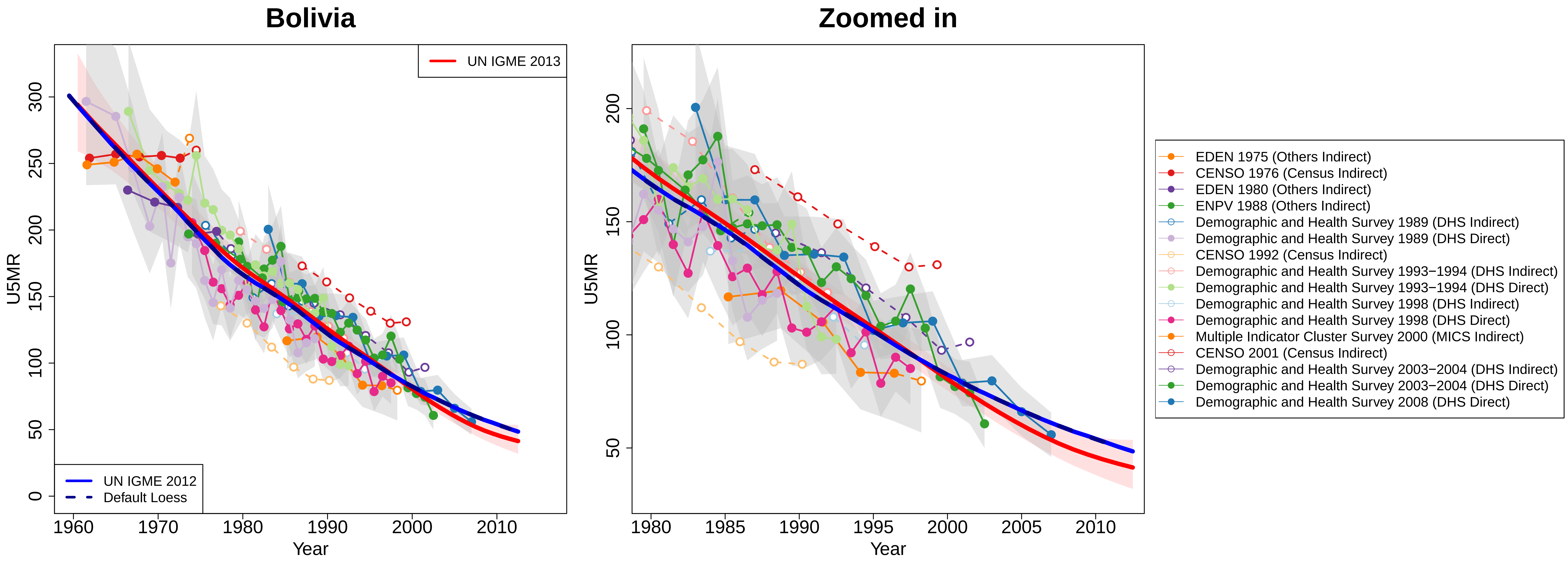

Bolivia
Zoomed in
U5MR
Year
UN IGME 2013
UN IGME 2012
Default Loess
EDEN 1975 (Others Indirect)
CENSO 1976 (Census Indirect)
EDEN 1980 (Others Indirect)
ENPV 1988 (Others Indirect)
Demographic and Health Survey 1989 (DHS Indirect)
Demographic and Health Survey 1989 (DHS Direct)
CENSO 1992 (Census Indirect)
Demographic and Health Survey 1993−1994 (DHS Indirect)
Demographic and Health Survey 1993−1994 (DHS Direct)
Demographic and Health Survey 1998 (DHS Indirect)
Demographic and Health Survey 1998 (DHS Direct)
Multiple Indicator Cluster Survey 2000 (MICS Indirect)
CENSO 2001 (Census Indirect)
Demographic and Health Survey 2003−2004 (DHS Indirect)
Demographic and Health Survey 2003−2004 (DHS Direct)
Demographic and Health Survey 2008 (DHS Direct)

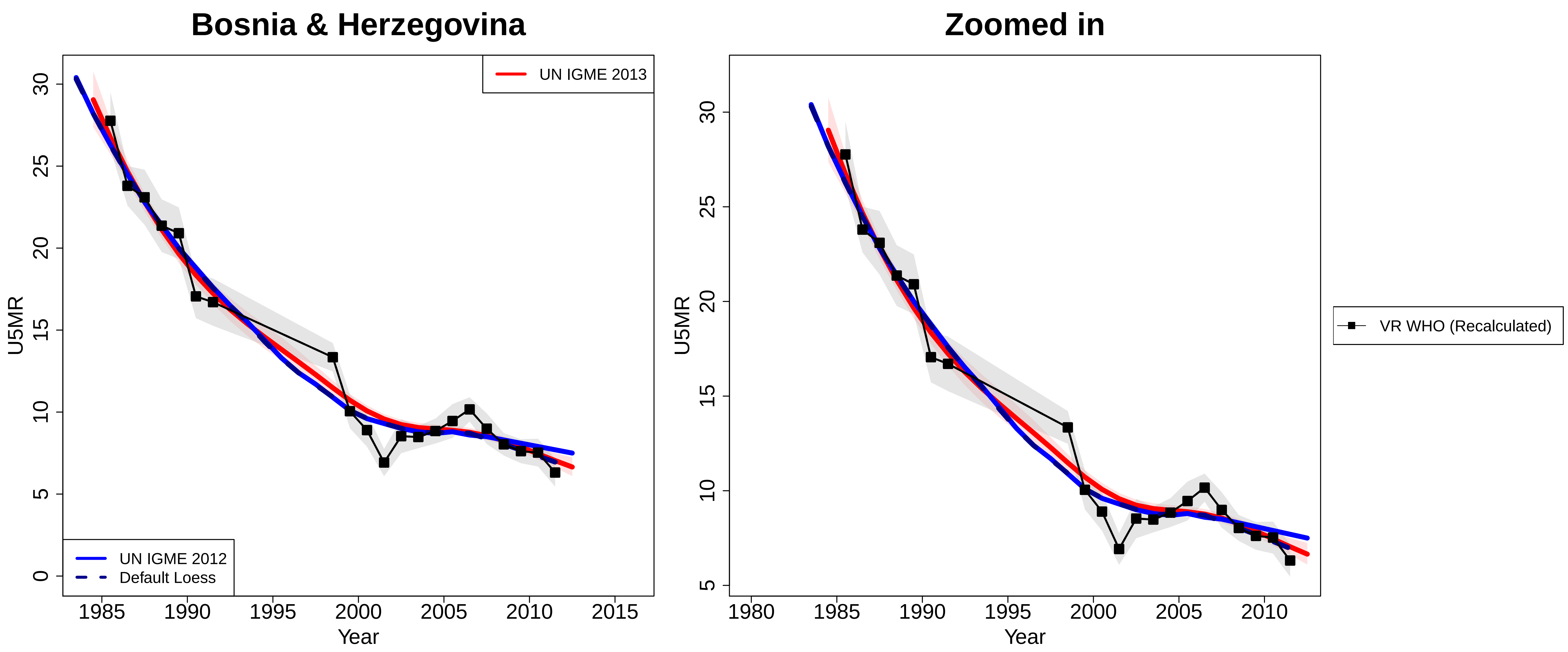

Bosnia & Herzegovina
Zoomed in
UN IGME 2013
UN IGME 2012
Default Loess
VR WHO (Recalculated)
U5MR
Year
0
5
10
15
20
25
30
1980
1985
1990
1995
2000
2005
2010
2015

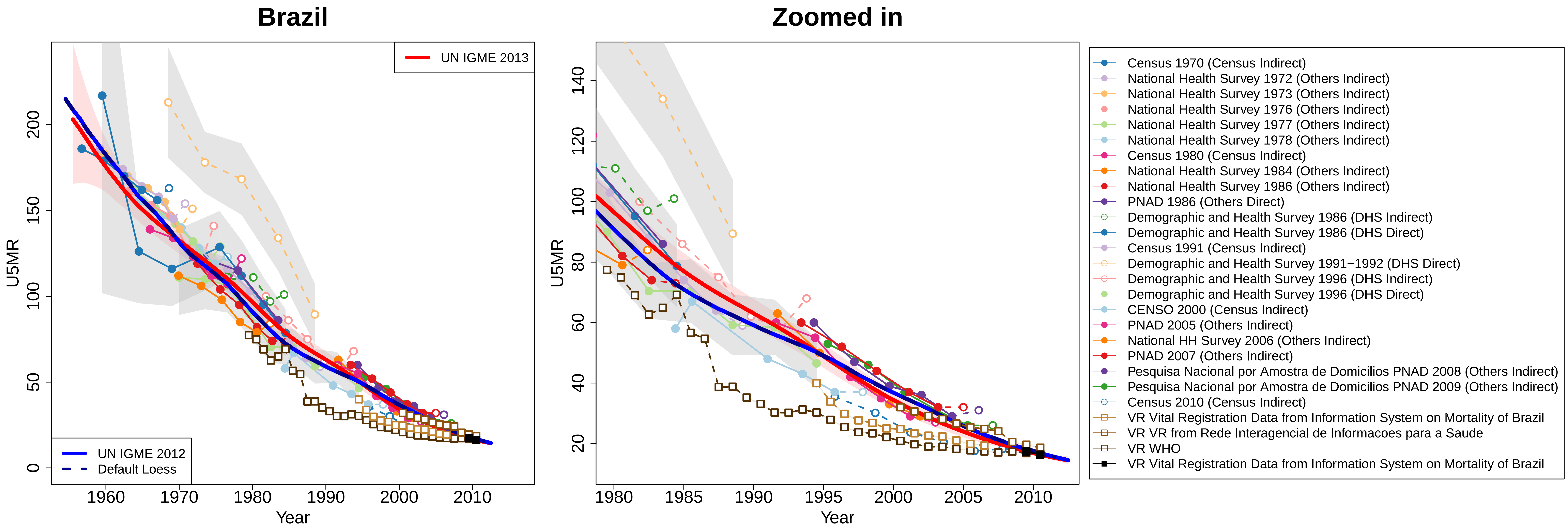

Brazil
Zoomed in
U5MR
Year
UN IGME 2013
UN IGME 2012
Default Loess
Census 1970 (Census Indirect)
National Health Survey 1972 (Others Indirect)
National Health Survey 1973 (Others Indirect)
National Health Survey 1976 (Others Indirect)
National Health Survey 1977 (Others Indirect)
National Health Survey 1978 (Others Indirect)
Census 1980 (Census Indirect)
National Health Survey 1984 (Others Indirect)
National Health Survey 1986 (Others Indirect)
PNAD 1986 (Others Direct)
Demographic and Health Survey 1986 (DHS Indirect)
Demographic and Health Survey 1986 (DHS Direct)
Census 1991 (Census Indirect)
Demographic and Health Survey 1991−1992 (DHS Direct)
Demographic and Health Survey 1996 (DHS Indirect)
Demographic and Health Survey 1996 (DHS Direct)
CENSO 2000 (Census Indirect)
PNAD 2005 (Others Indirect)
National HH Survey 2006 (Others Indirect)
PNAD 2007 (Others Indirect)
Pesquisa Nacional por Amostra de Domicilios PNAD 2008 (Others Indirect)
Pesquisa Nacional por Amostra de Domicilios PNAD 2009 (Others Indirect)
Census 2010 (Census Indirect)
VR Vital Registration Data from Information System on Mortality of Brazil
VR VR from Rede Interagencial de Informacoes para a Saude
VR WHO
VR Vital Registration Data from Information System on Mortality of Brazil

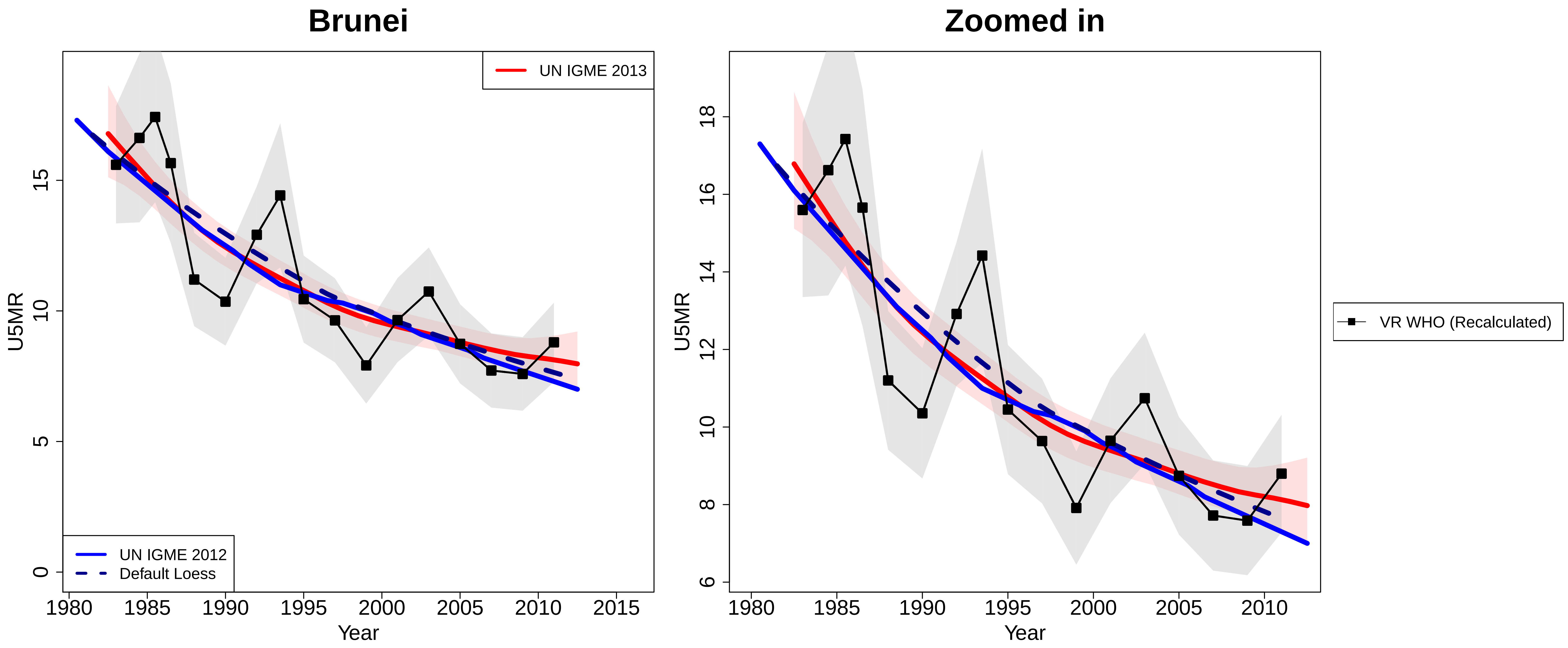

Brunei
Zoomed in
UN IGME 2013
UN IGME 2012
Default Loess
VR WHO (Recalculated)
U5MR
Year
0
5
10
15
6
8
10
12
14
16
18
1980
1985
1990
1995
2000
2005
2010
2015

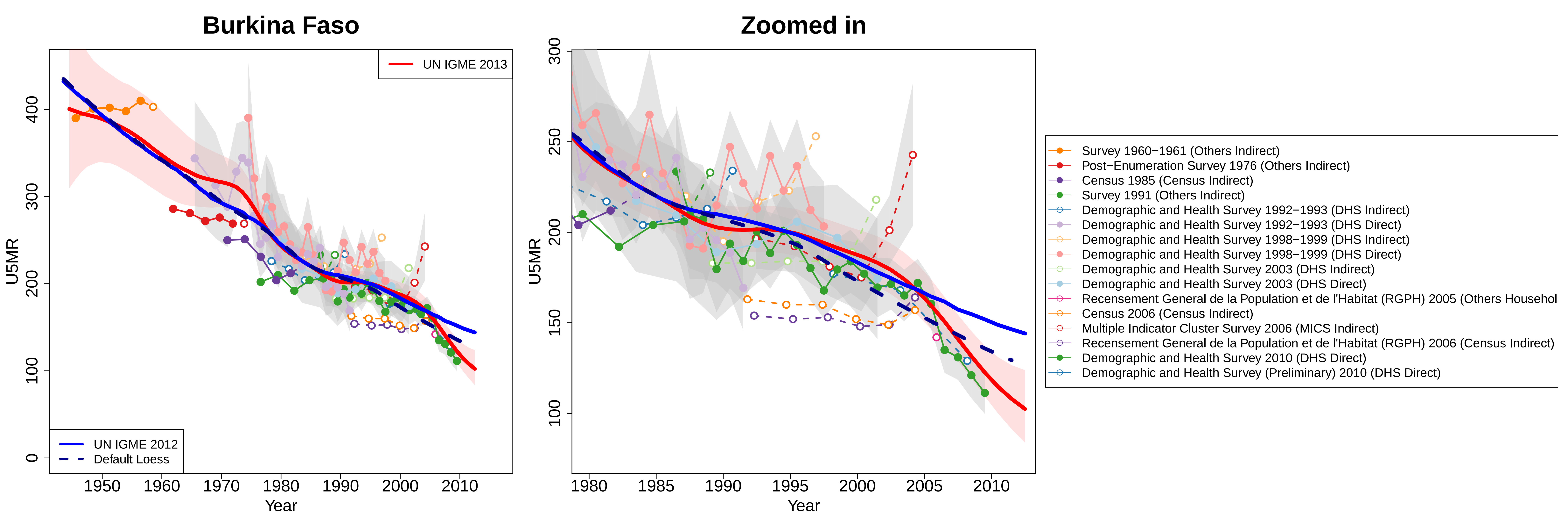
Burkina Faso
Zoomed in
U5MR
Year
UN IGME 2013
UN IGME 2012
Default Loess
Survey 1960−1961 (Others Indirect)
Post−Enumeration Survey 1976 (Others Indirect)
Census 1985 (Census Indirect)
Survey 1991 (Others Indirect)
Demographic and Health Survey 1992−1993 (DHS Indirect)
Demographic and Health Survey 1992−1993 (DHS Direct)
Demographic and Health Survey 1998−1999 (DHS Indirect)
Demographic and Health Survey 1998−1999 (DHS Direct)
Demographic and Health Survey 2003 (DHS Indirect)
Demographic and Health Survey 2003 (DHS Direct)
Recensement General de la Population et de l'Habitat (RGPH) 2005 (Others Househol
Census 2006 (Census Indirect)
Multiple Indicator Cluster Survey 2006 (MICS Indirect)
Recensement General de la Population et de l'Habitat (RGPH) 2006 (Census Indirect)
Demographic and Health Survey 2010 (DHS Direct)
Demographic and Health Survey (Preliminary) 2010 (DHS Direct)

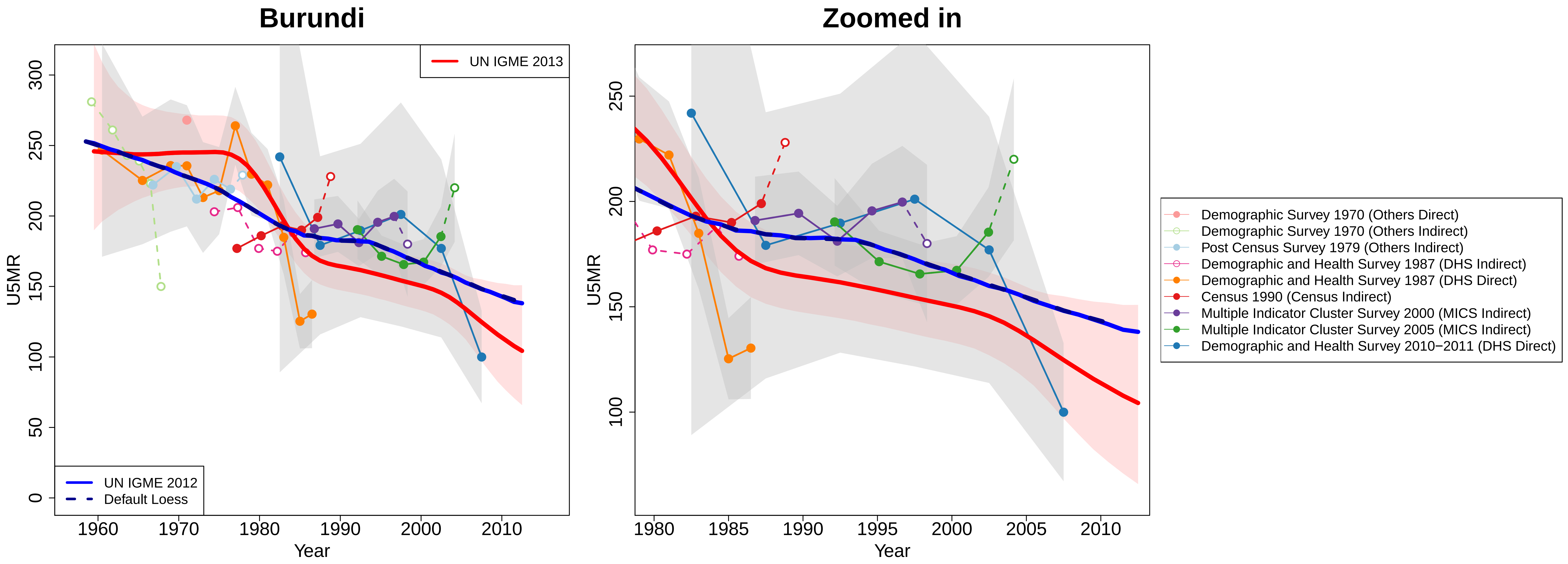

Burundi
Zoomed in
UN IGME 2013
UN IGME 2012
Default Loess
U5MR
Year
Demographic Survey 1970 (Others Direct)
Demographic Survey 1970 (Others Indirect)
Post Census Survey 1979 (Others Indirect)
Demographic and Health Survey 1987 (DHS Indirect)
Demographic and Health Survey 1987 (DHS Direct)
Census 1990 (Census Indirect)
Multiple Indicator Cluster Survey 2000 (MICS Indirect)
Multiple Indicator Cluster Survey 2005 (MICS Indirect)
Demographic and Health Survey 2010−2011 (DHS Direct)

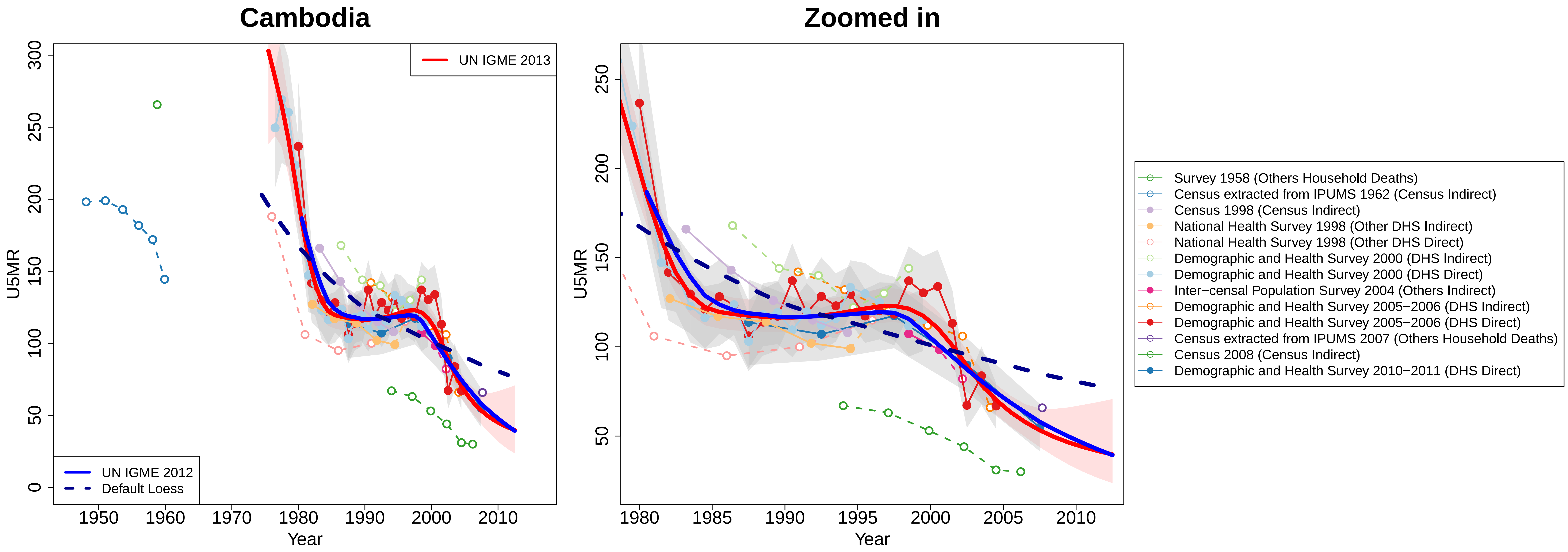

Cambodia
Zoomed in
U5MR
Year
UN IGME 2013
UN IGME 2012
Default Loess
0
50
100
150
200
250
300
1950
1960
1970
1980
1985
1990
1995
2000
2005
2010
Survey 1958 (Others Household Deaths)
Census extracted from IPUMS 1962 (Census Indirect)
Census 1998 (Census Indirect)
National Health Survey 1998 (Other DHS Indirect)
National Health Survey 1998 (Other DHS Direct)
Demographic and Health Survey 2000 (DHS Indirect)
Demographic and Health Survey 2000 (DHS Direct)
Inter−censal Population Survey 2004 (Others Indirect)
Demographic and Health Survey 2005−2006 (DHS Indirect)
Demographic and Health Survey 2005−2006 (DHS Direct)
Census extracted from IPUMS 2007 (Others Household Deaths)
Census 2008 (Census Indirect)
Demographic and Health Survey 2010−2011 (DHS Direct)

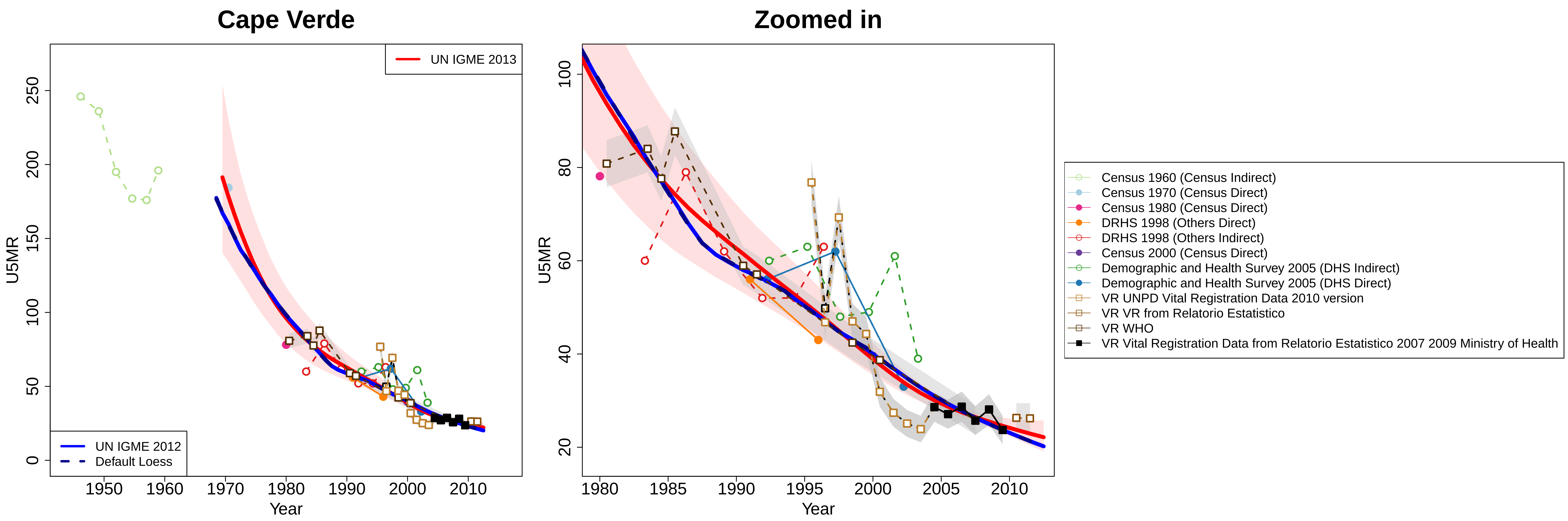
Cape Verde
Zoomed in
U5MR
Year
UN IGME 2013
UN IGME 2012
Default Loess
0
50
100
150
200
250
1950
1960
1970
1980
1990
2000
2010
20
40
60
80
100
1985
1995
2005
Census 1960 (Census Indirect)
Census 1970 (Census Direct)
Census 1980 (Census Direct)
DRHS 1998 (Others Direct)
DRHS 1998 (Others Indirect)
Census 2000 (Census Direct)
Demographic and Health Survey 2005 (DHS Indirect)
Demographic and Health Survey 2005 (DHS Direct)
VR UNPD Vital Registration Data 2010 version
VR VR from Relatorio Estatistico
VR WHO
VR Vital Registration Data from Relatorio Estatistico 2007 2009 Ministry of Health

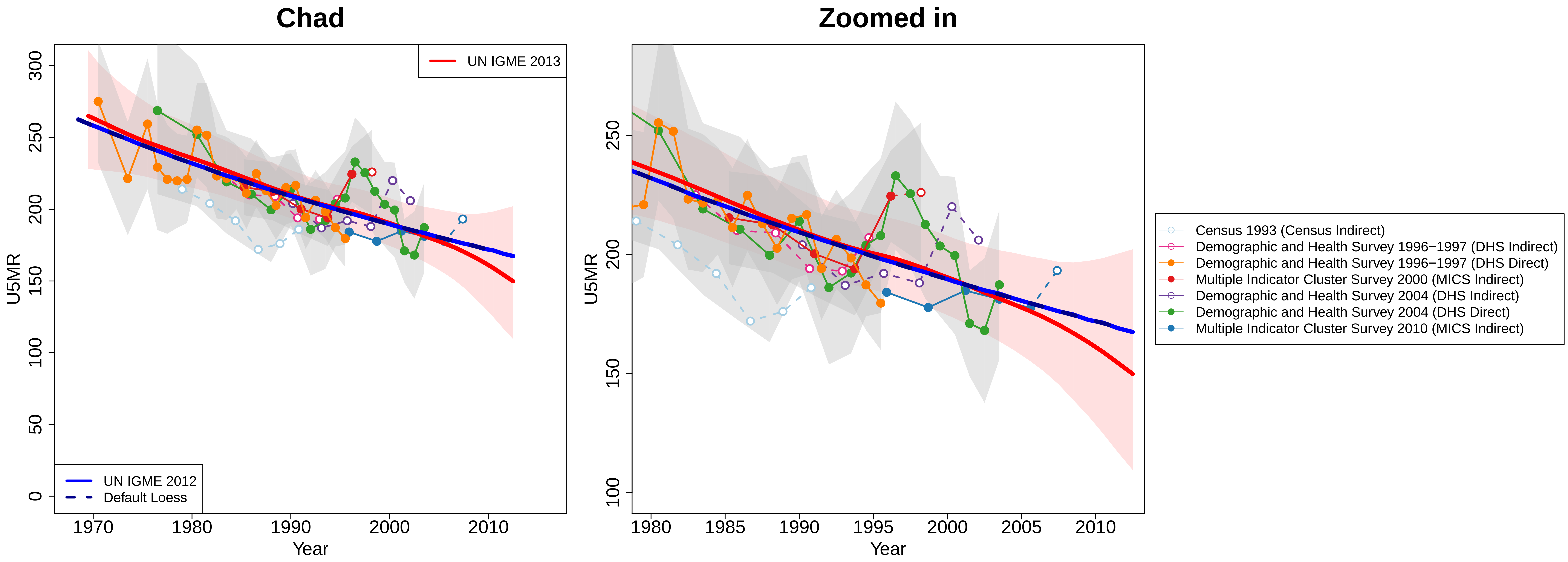
Chad
Zoomed in
U5MR
Year
UN IGME 2013
UN IGME 2012
Default Loess
Census 1993 (Census Indirect)
Demographic and Health Survey 1996−1997 (DHS Indirect)
Demographic and Health Survey 1996−1997 (DHS Direct)
Multiple Indicator Cluster Survey 2000 (MICS Indirect)
Demographic and Health Survey 2004 (DHS Indirect)
Demographic and Health Survey 2004 (DHS Direct)
Multiple Indicator Cluster Survey 2010 (MICS Indirect)

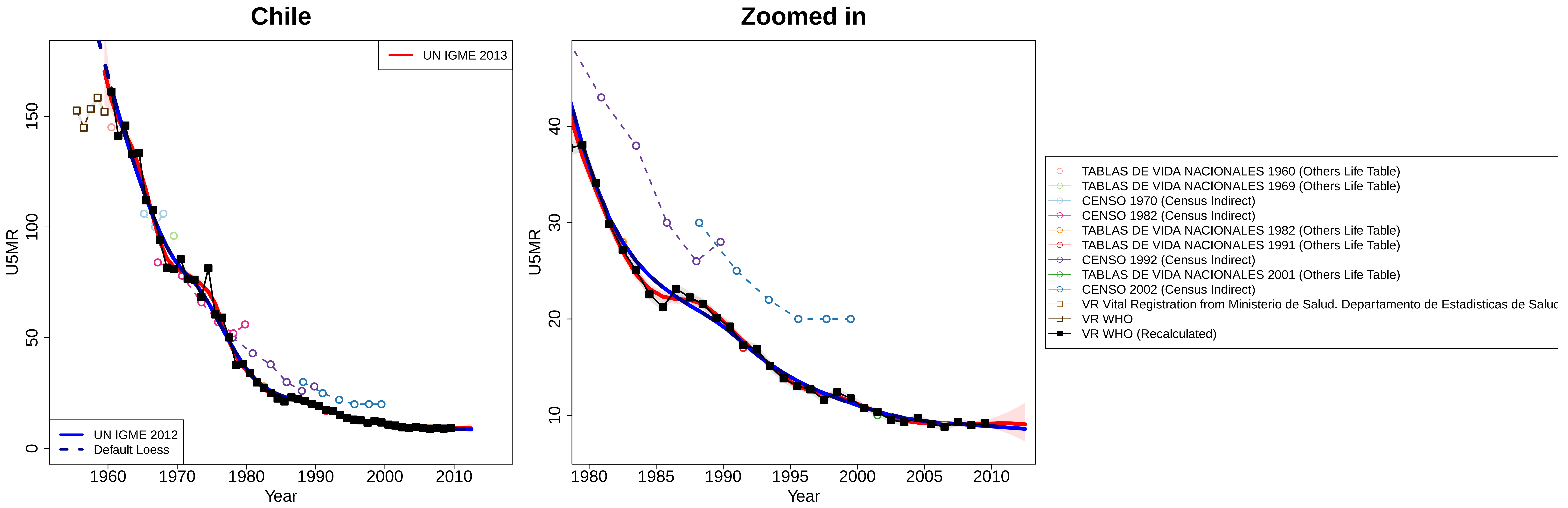

Chile
Zoomed in
UN IGME 2013
UN IGME 2012
Default Loess
U5MR
Year
TABLAS DE VIDA NACIONALES 1960 (Others Life Table)
TABLAS DE VIDA NACIONALES 1969 (Others Life Table)
CENSO 1970 (Census Indirect)
CENSO 1982 (Census Indirect)
TABLAS DE VIDA NACIONALES 1982 (Others Life Table)
TABLAS DE VIDA NACIONALES 1991 (Others Life Table)
CENSO 1992 (Census Indirect)
TABLAS DE VIDA NACIONALES 2001 (Others Life Table)
CENSO 2002 (Census Indirect)
VR Vital Registration from Ministerio de Salud. Departamento de Estadisticas de Saluc
VR WHO
VR WHO (Recalculated)

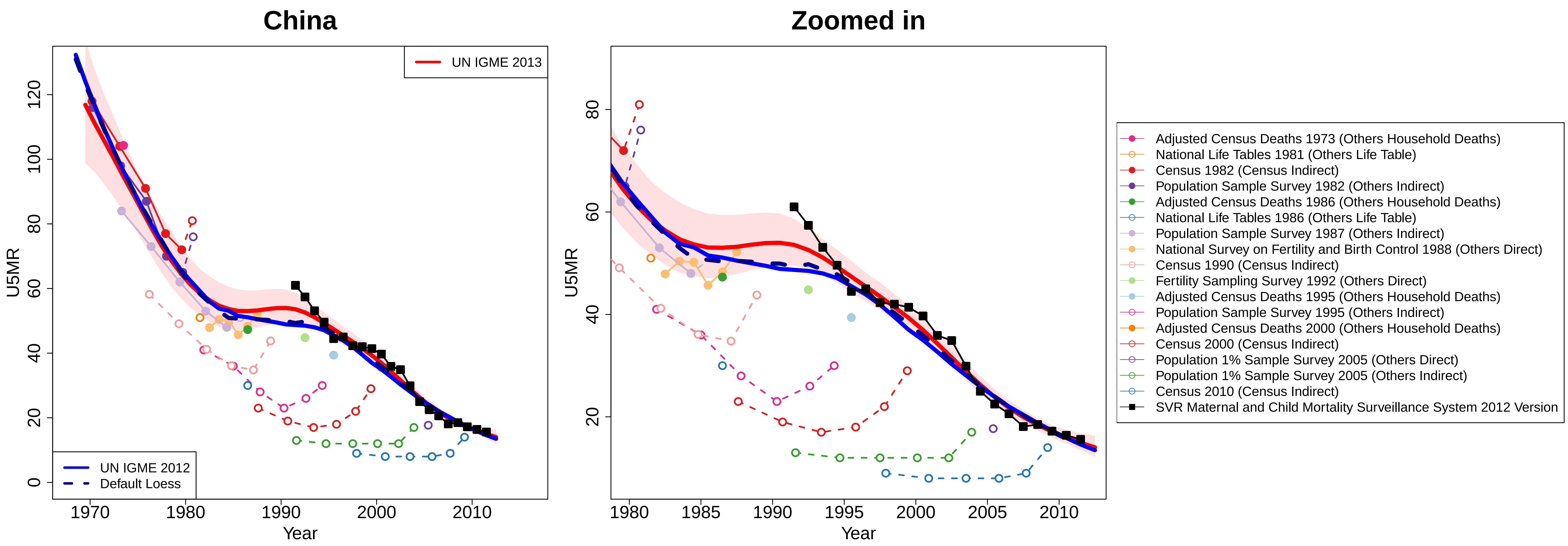
China
Zoomed in
UN IGME 2013
UN IGME 2012
Default Loess
U5MR
Year
Adjusted Census Deaths 1973 (Others Household Deaths)
National Life Tables 1981 (Others Life Table)
Census 1982 (Census Indirect)
Population Sample Survey 1982 (Others Indirect)
Adjusted Census Deaths 1986 (Others Household Deaths)
National Life Tables 1986 (Others Life Table)
Population Sample Survey 1987 (Others Indirect)
National Survey on Fertility and Birth Control 1988 (Others Direct)
Census 1990 (Census Indirect)
Fertility Sampling Survey 1992 (Others Direct)
Adjusted Census Deaths 1995 (Others Household Deaths)
Population Sample Survey 1995 (Others Indirect)
Adjusted Census Deaths 2000 (Others Household Deaths)
Census 2000 (Census Indirect)
Population 1% Sample Survey 2005 (Others Direct)
Population 1% Sample Survey 2005 (Others Indirect)
Census 2010 (Census Indirect)
SVR Maternal and Child Mortality Surveillance System 2012 Version

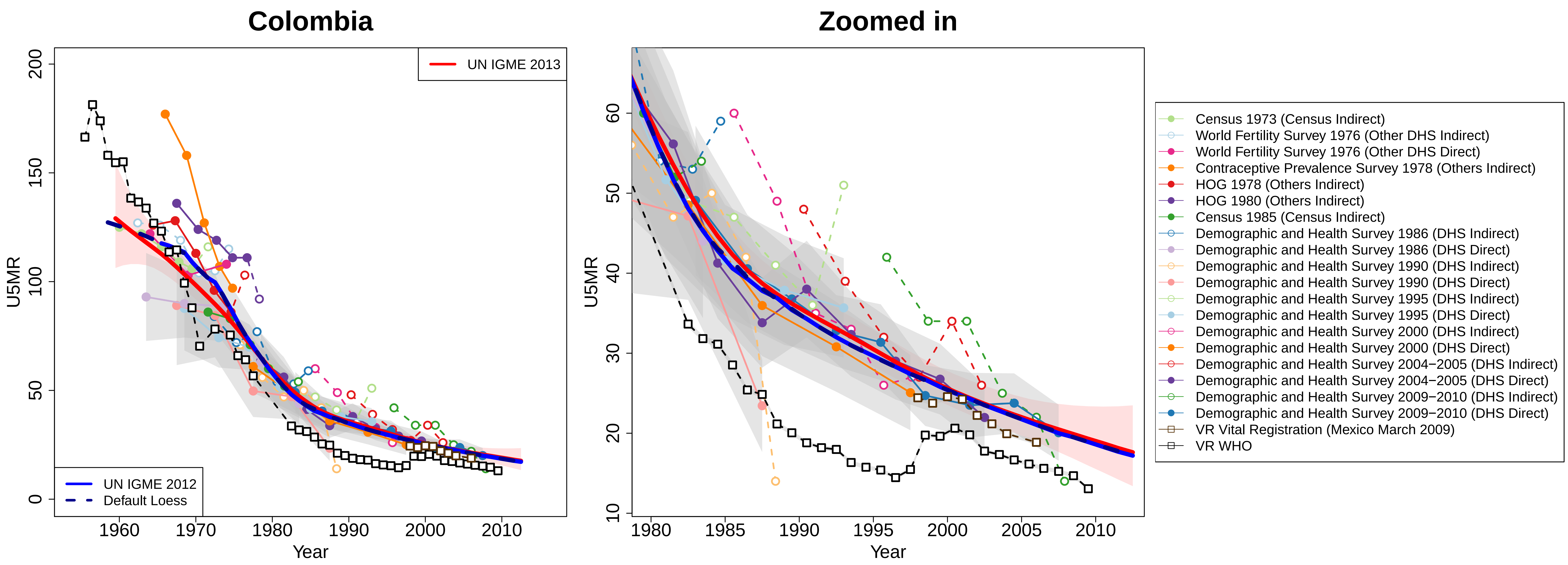

Colombia
Zoomed in
U5MR
Year
UN IGME 2013
UN IGME 2012
Default Loess
Census 1973 (Census Indirect)
World Fertility Survey 1976 (Other DHS Indirect)
World Fertility Survey 1976 (Other DHS Direct)
Contraceptive Prevalence Survey 1978 (Others Indirect)
HOG 1978 (Others Indirect)
HOG 1980 (Others Indirect)
Census 1985 (Census Indirect)
Demographic and Health Survey 1986 (DHS Indirect)
Demographic and Health Survey 1986 (DHS Direct)
Demographic and Health Survey 1990 (DHS Indirect)
Demographic and Health Survey 1990 (DHS Direct)
Demographic and Health Survey 1995 (DHS Indirect)
Demographic and Health Survey 1995 (DHS Direct)
Demographic and Health Survey 2000 (DHS Indirect)
Demographic and Health Survey 2000 (DHS Direct)
Demographic and Health Survey 2004−2005 (DHS Indirect)
Demographic and Health Survey 2004−2005 (DHS Direct)
Demographic and Health Survey 2009−2010 (DHS Indirect)
Demographic and Health Survey 2009−2010 (DHS Direct)
VR Vital Registration (Mexico March 2009)
VR WHO

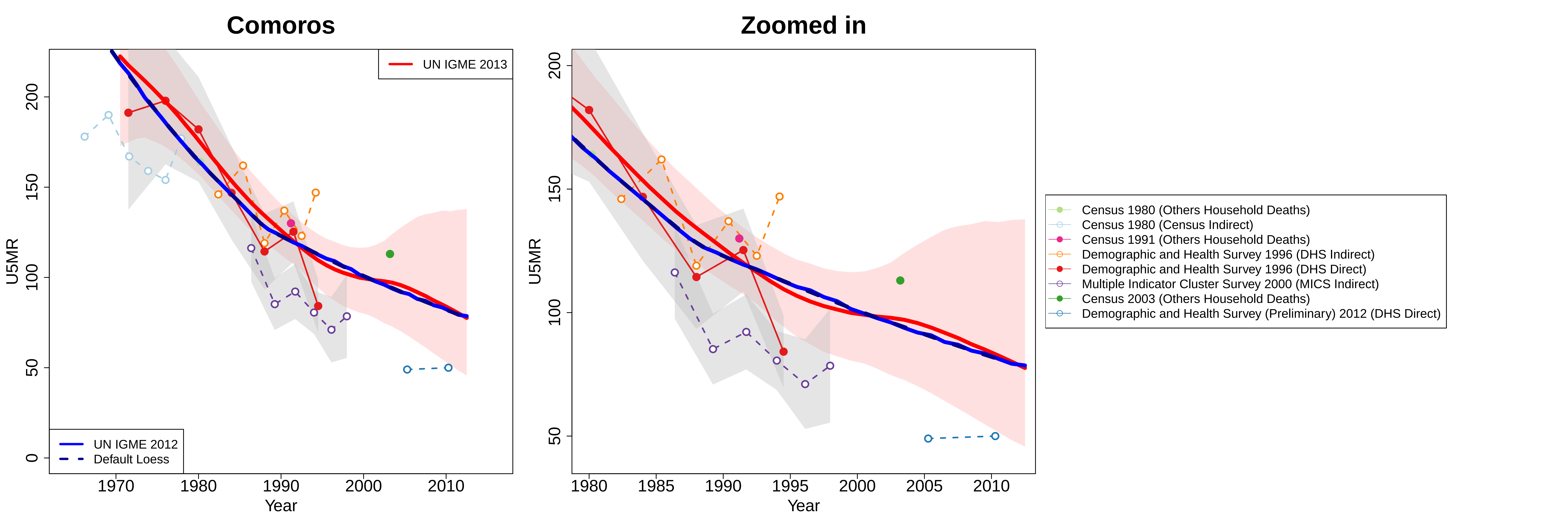

Comoros
Zoomed in
UN IGME 2013
UN IGME 2012
Default Loess
U5MR
Year
Census 1980 (Others Household Deaths)
Census 1980 (Census Indirect)
Census 1991 (Others Household Deaths)
Demographic and Health Survey 1996 (DHS Indirect)
Demographic and Health Survey 1996 (DHS Direct)
Multiple Indicator Cluster Survey 2000 (MICS Indirect)
Census 2003 (Others Household Deaths)
Demographic and Health Survey (Preliminary) 2012 (DHS Direct)

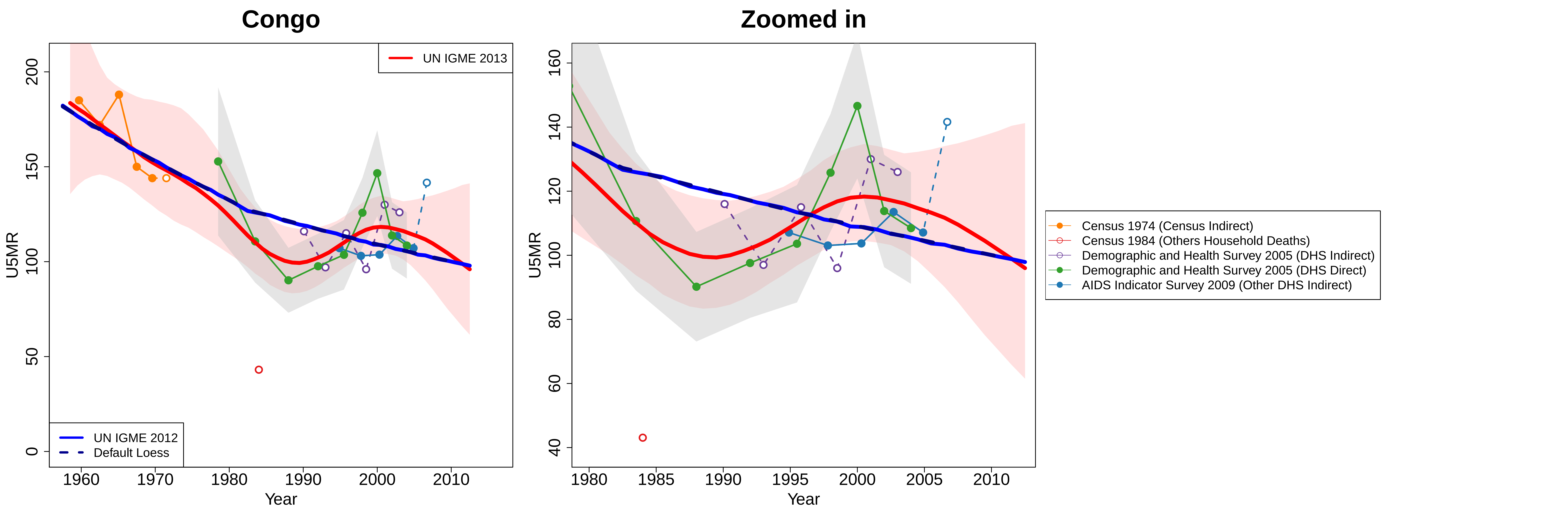

Congo
Zoomed in
UN IGME 2013
UN IGME 2012
Default Loess
U5MR
Year
Census 1974 (Census Indirect)
Census 1984 (Others Household Deaths)
Demographic and Health Survey 2005 (DHS Indirect)
Demographic and Health Survey 2005 (DHS Direct)
AIDS Indicator Survey 2009 (Other DHS Indirect)

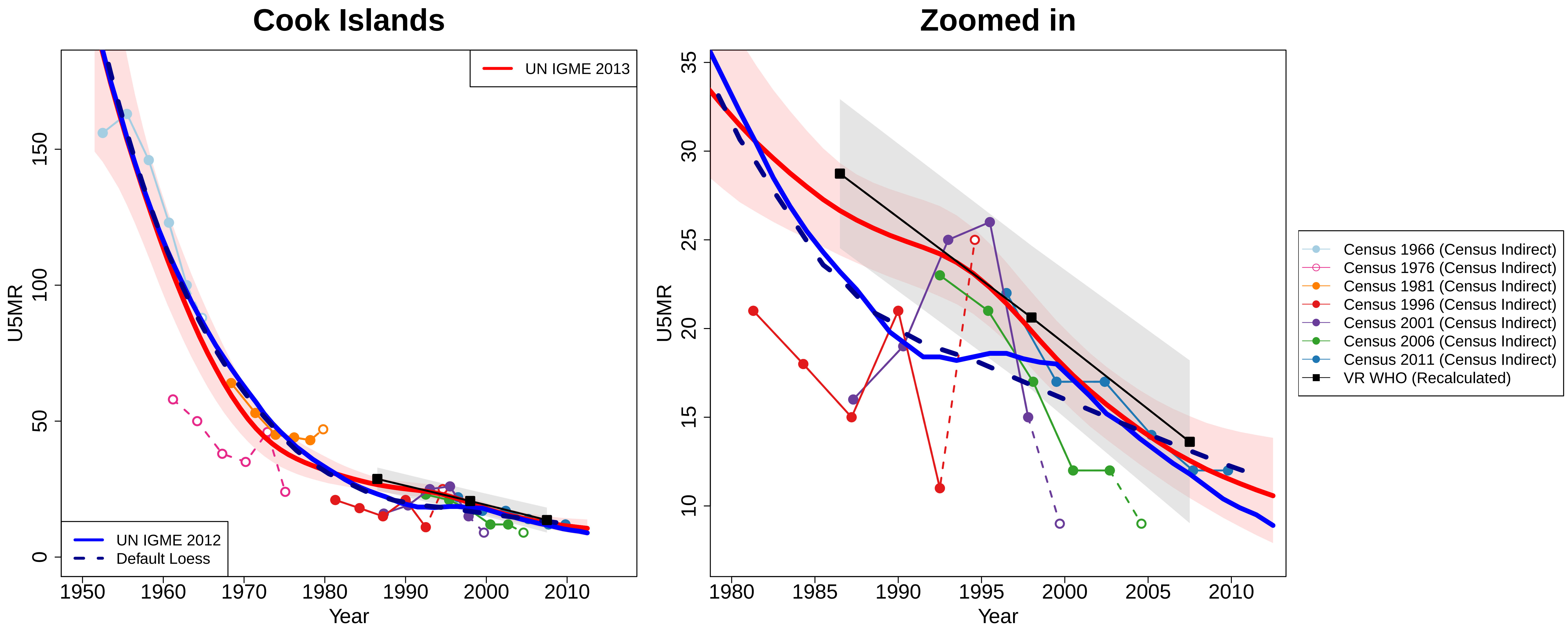

Cook Islands
UN IGME 2013
UN IGME 2012
Default Loess
U5MR
Year
Zoomed in
Census 1966 (Census Indirect)
Census 1976 (Census Indirect)
Census 1981 (Census Indirect)
Census 1996 (Census Indirect)
Census 2001 (Census Indirect)
Census 2006 (Census Indirect)
Census 2011 (Census Indirect)
VR WHO (Recalculated)

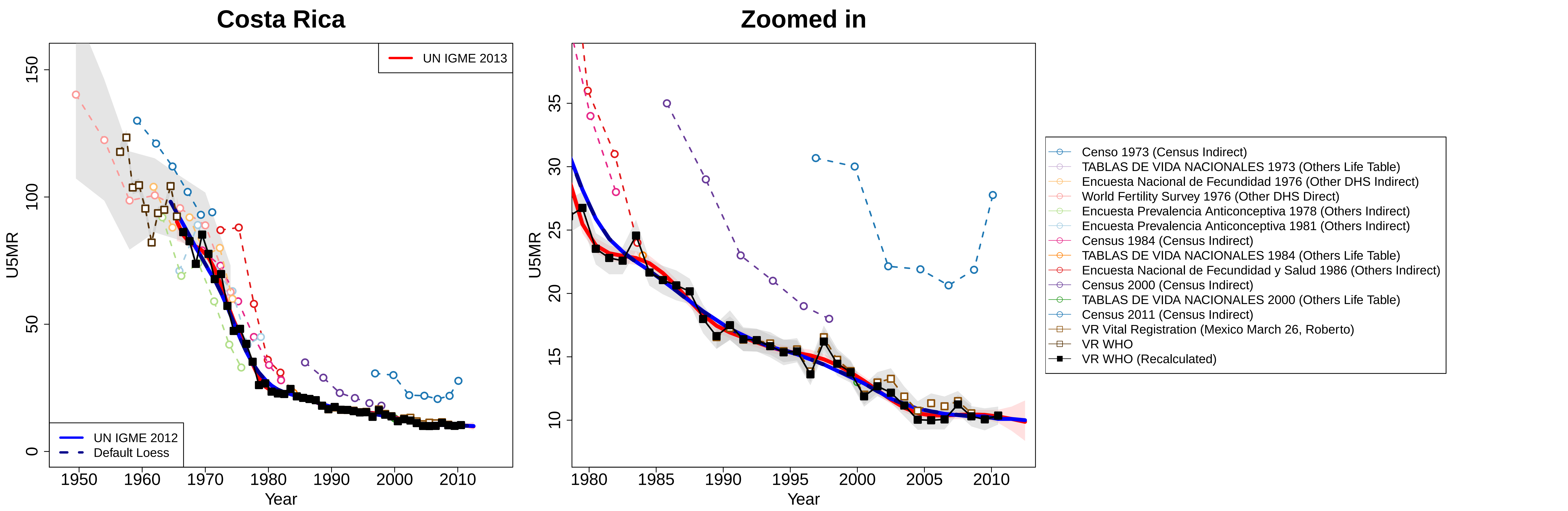
Costa Rica
Zoomed in
UN IGME 2013
UN IGME 2012
Default Loess
U5MR
Year
Censo 1973 (Census Indirect)
TABLAS DE VIDA NACIONALES 1973 (Others Life Table)
Encuesta Nacional de Fecundidad 1976 (Other DHS Indirect)
World Fertility Survey 1976 (Other DHS Direct)
Encuesta Prevalencia Anticonceptiva 1978 (Others Indirect)
Encuesta Prevalencia Anticonceptiva 1981 (Others Indirect)
Census 1984 (Census Indirect)
TABLAS DE VIDA NACIONALES 1984 (Others Life Table)
Encuesta Nacional de Fecundidad y Salud 1986 (Others Indirect)
Census 2000 (Census Indirect)
TABLAS DE VIDA NACIONALES 2000 (Others Life Table)
Census 2011 (Census Indirect)
VR Vital Registration (Mexico March 26, Roberto)
VR WHO
VR WHO (Recalculated)

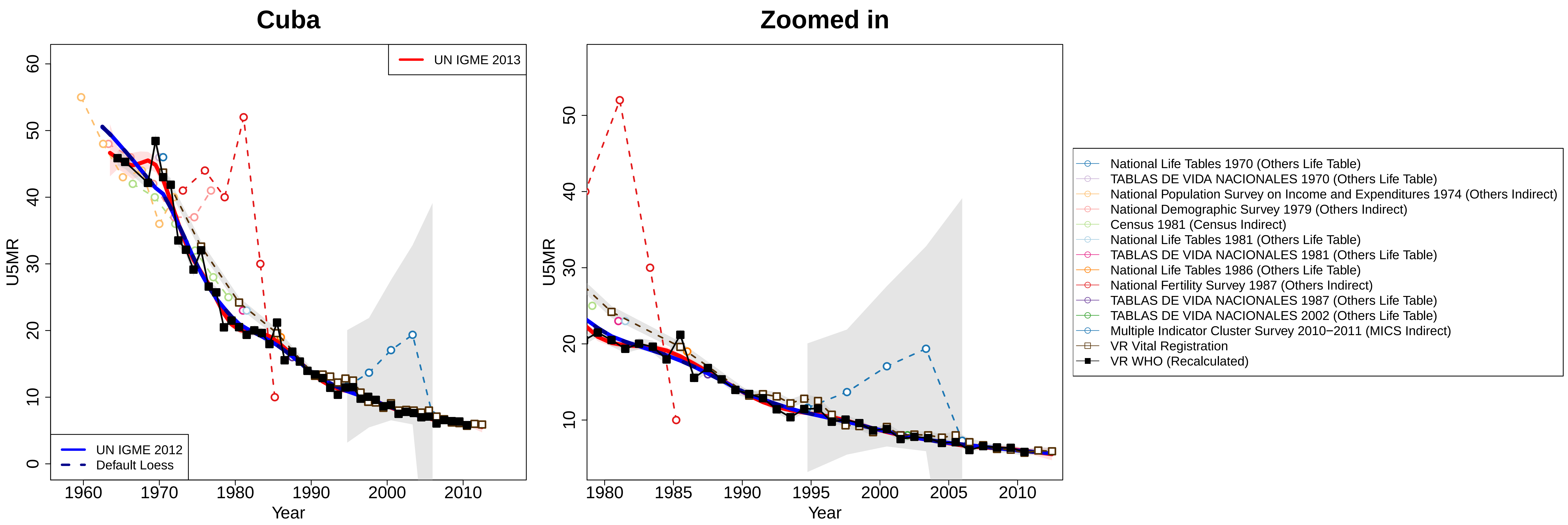
Cuba
Zoomed in
U5MR
Year
UN IGME 2013
UN IGME 2012
Default Loess
National Life Tables 1970 (Others Life Table)
TABLAS DE VIDA NACIONALES 1970 (Others Life Table)
National Population Survey on Income and Expenditures 1974 (Others Indirect)
National Demographic Survey 1979 (Others Indirect)
Census 1981 (Census Indirect)
National Life Tables 1981 (Others Life Table)
TABLAS DE VIDA NACIONALES 1981 (Others Life Table)
National Life Tables 1986 (Others Life Table)
National Fertility Survey 1987 (Others Indirect)
TABLAS DE VIDA NACIONALES 1987 (Others Life Table)
TABLAS DE VIDA NACIONALES 2002 (Others Life Table)
Multiple Indicator Cluster Survey 2010−2011 (MICS Indirect)
VR Vital Registration
VR WHO (Recalculated)

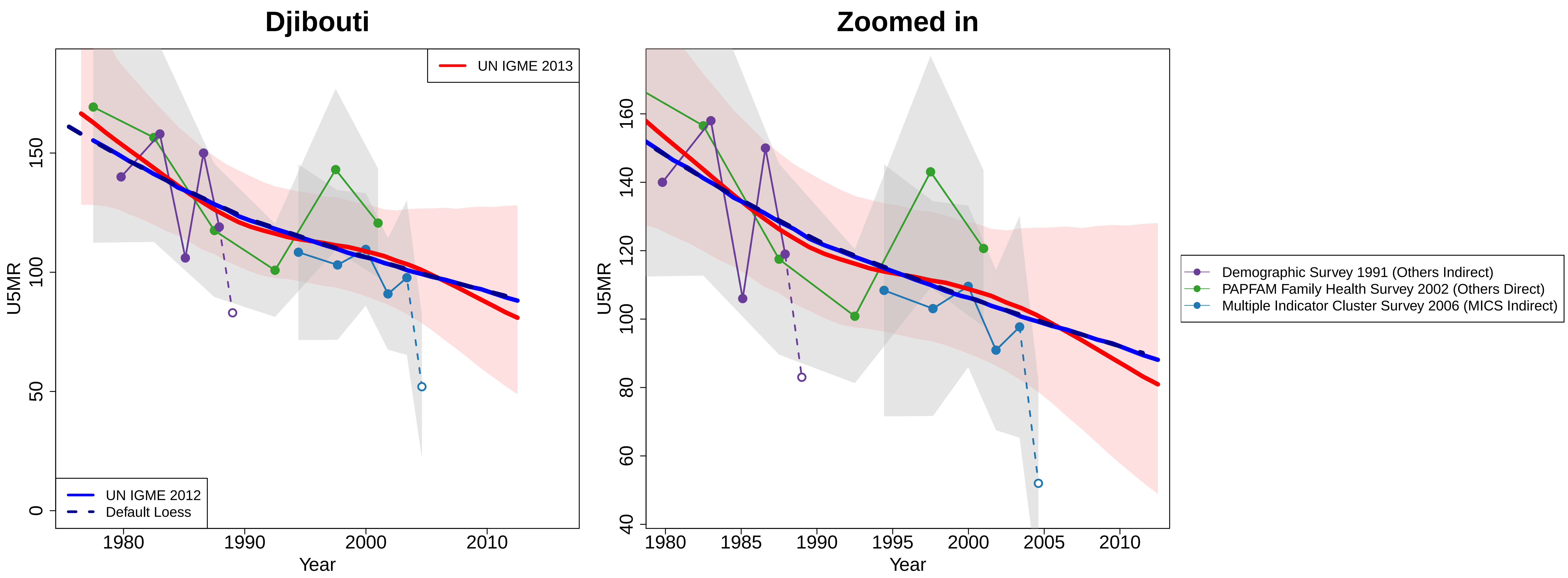
Djibouti
UN IGME 2013
UN IGME 2012
Default Loess
U5MR
Year
0
50
100
150
1980
1990
2000
2010
Zoomed in
U5MR
Year
40
60
80
100
120
140
160
1980
1985
1990
1995
2000
2005
2010
Demographic Survey 1991 (Others Indirect)
PAPFAM Family Health Survey 2002 (Others Direct)
Multiple Indicator Cluster Survey 2006 (MICS Indirect)

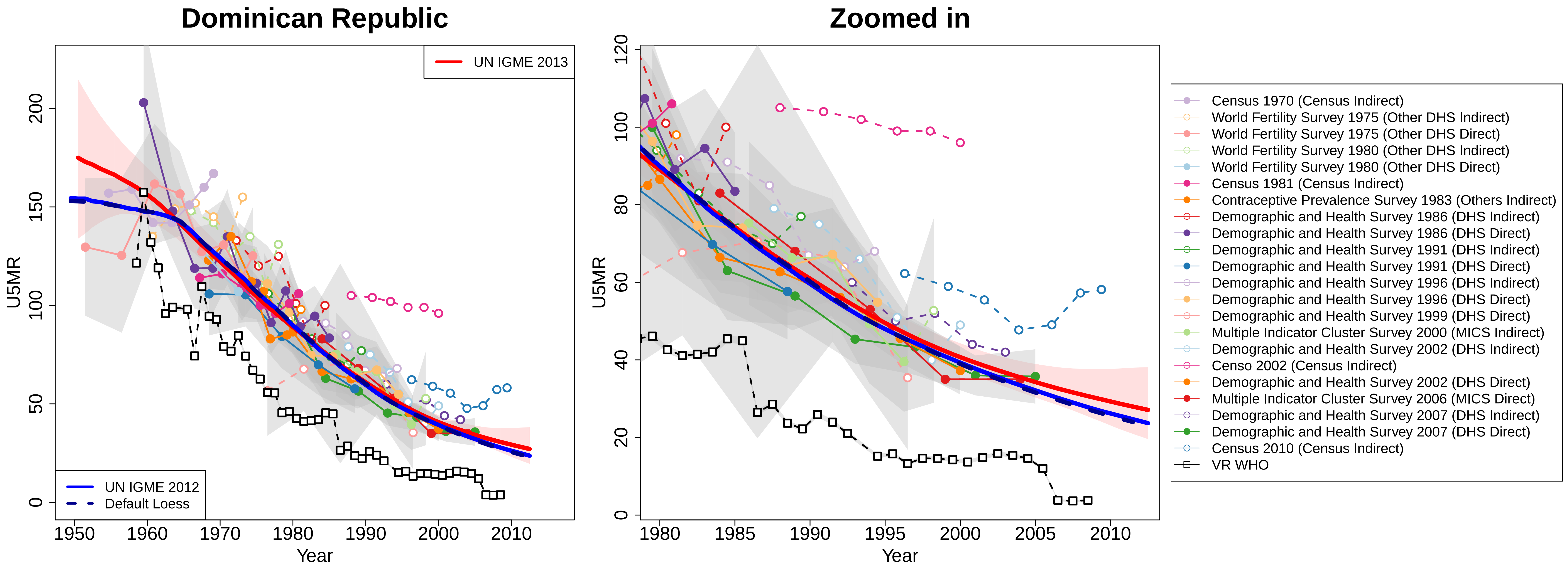

Dominican Republic
Zoomed in
U5MR
Year
UN IGME 2013
UN IGME 2012
Default Loess
Census 1970 (Census Indirect)
World Fertility Survey 1975 (Other DHS Indirect)
World Fertility Survey 1975 (Other DHS Direct)
World Fertility Survey 1980 (Other DHS Indirect)
World Fertility Survey 1980 (Other DHS Direct)
Census 1981 (Census Indirect)
Contraceptive Prevalence Survey 1983 (Others Indirect)
Demographic and Health Survey 1986 (DHS Indirect)
Demographic and Health Survey 1986 (DHS Direct)
Demographic and Health Survey 1991 (DHS Indirect)
Demographic and Health Survey 1991 (DHS Direct)
Demographic and Health Survey 1996 (DHS Indirect)
Demographic and Health Survey 1996 (DHS Direct)
Demographic and Health Survey 1999 (DHS Direct)
Multiple Indicator Cluster Survey 2000 (MICS Indirect)
Demographic and Health Survey 2002 (DHS Indirect)
Censo 2002 (Census Indirect)
Demographic and Health Survey 2002 (DHS Direct)
Multiple Indicator Cluster Survey 2006 (MICS Direct)
Demographic and Health Survey 2007 (DHS Indirect)
Demographic and Health Survey 2007 (DHS Direct)
Census 2010 (Census Indirect)
VR WHO

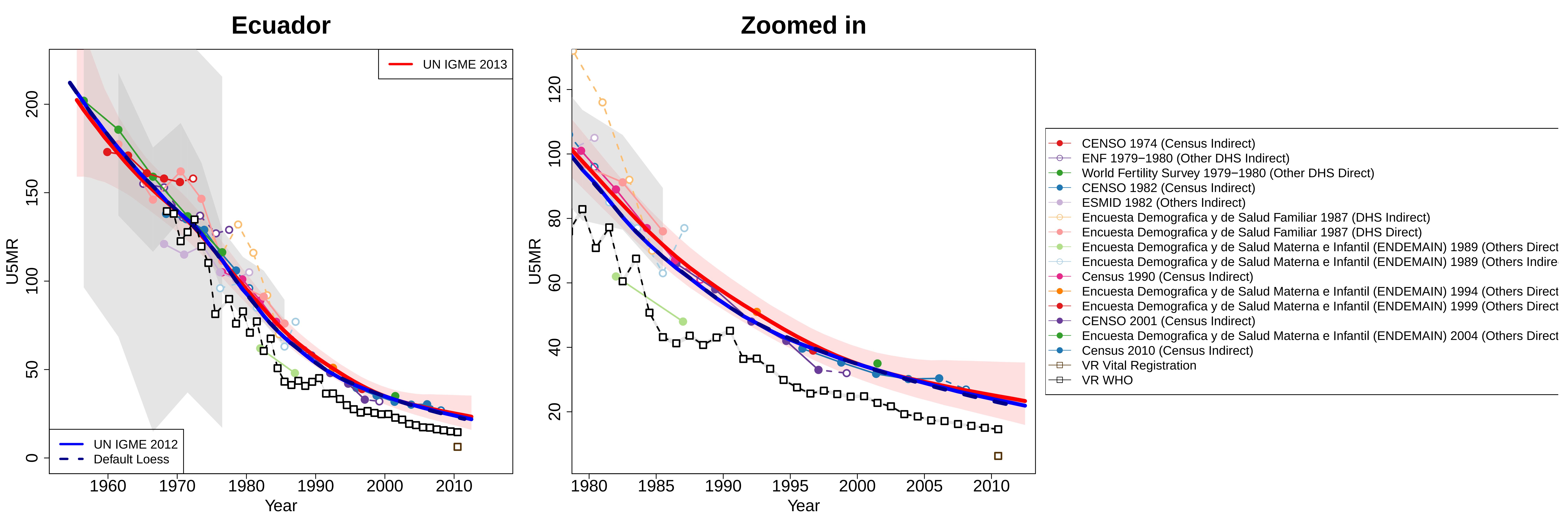

Ecuador
Zoomed in
UN IGME 2013
UN IGME 2012
Default Loess
U5MR
Year
CENSO 1974 (Census Indirect)
ENF 1979−1980 (Other DHS Indirect)
World Fertility Survey 1979−1980 (Other DHS Direct)
CENSO 1982 (Census Indirect)
ESMID 1982 (Others Indirect)
Encuesta Demografica y de Salud Familiar 1987 (DHS Indirect)
Encuesta Demografica y de Salud Familiar 1987 (DHS Direct)
Encuesta Demografica y de Salud Materna e Infantil (ENDEMAIN) 1989 (Others Direct
Encuesta Demografica y de Salud Materna e Infantil (ENDEMAIN) 1989 (Others Indire
Census 1990 (Census Indirect)
Encuesta Demografica y de Salud Materna e Infantil (ENDEMAIN) 1994 (Others Direct
Encuesta Demografica y de Salud Materna e Infantil (ENDEMAIN) 1999 (Others Direct
CENSO 2001 (Census Indirect)
Encuesta Demografica y de Salud Materna e Infantil (ENDEMAIN) 2004 (Others Direct
Census 2010 (Census Indirect)
VR Vital Registration
VR WHO

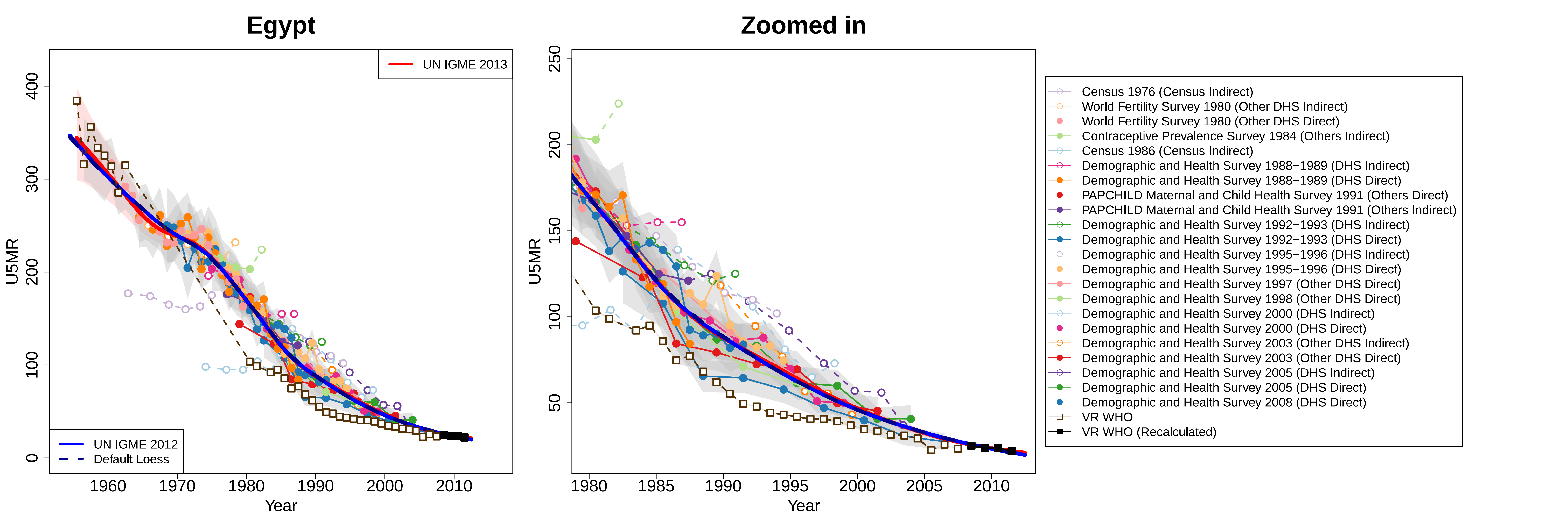

Egypt
Zoomed in
U5MR
Year
UN IGME 2013
UN IGME 2012
Default Loess
Census 1976 (Census Indirect)
World Fertility Survey 1980 (Other DHS Indirect)
World Fertility Survey 1980 (Other DHS Direct)
Contraceptive Prevalence Survey 1984 (Others Indirect)
Census 1986 (Census Indirect)
Demographic and Health Survey 1988−1989 (DHS Indirect)
Demographic and Health Survey 1988−1989 (DHS Direct)
PAPCHILD Maternal and Child Health Survey 1991 (Others Direct)
PAPCHILD Maternal and Child Health Survey 1991 (Others Indirect)
Demographic and Health Survey 1992−1993 (DHS Indirect)
Demographic and Health Survey 1992−1993 (DHS Direct)
Demographic and Health Survey 1995−1996 (DHS Indirect)
Demographic and Health Survey 1995−1996 (DHS Direct)
Demographic and Health Survey 1997 (Other DHS Direct)
Demographic and Health Survey 1998 (Other DHS Direct)
Demographic and Health Survey 2000 (DHS Indirect)
Demographic and Health Survey 2000 (DHS Direct)
Demographic and Health Survey 2003 (Other DHS Indirect)
Demographic and Health Survey 2003 (Other DHS Direct)
Demographic and Health Survey 2005 (DHS Indirect)
Demographic and Health Survey 2005 (DHS Direct)
Demographic and Health Survey 2008 (DHS Direct)
VR WHO
VR WHO (Recalculated)

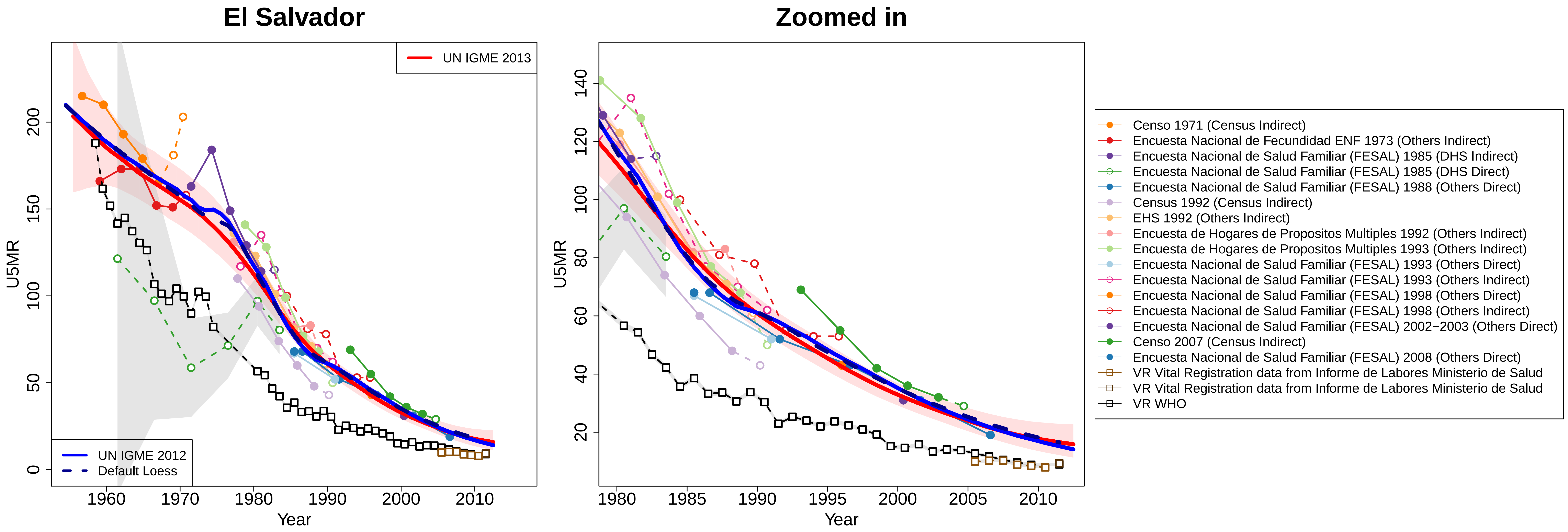
El Salvador
Zoomed in
UN IGME 2013
UN IGME 2012
Default Loess
U5MR
Year
Censo 1971 (Census Indirect)
Encuesta Nacional de Fecundidad ENF 1973 (Others Indirect)
Encuesta Nacional de Salud Familiar (FESAL) 1985 (DHS Indirect)
Encuesta Nacional de Salud Familiar (FESAL) 1985 (DHS Direct)
Encuesta Nacional de Salud Familiar (FESAL) 1988 (Others Direct)
Census 1992 (Census Indirect)
EHS 1992 (Others Indirect)
Encuesta de Hogares de Propositos Multiples 1992 (Others Indirect)
Encuesta de Hogares de Propositos Multiples 1993 (Others Indirect)
Encuesta Nacional de Salud Familiar (FESAL) 1993 (Others Direct)
Encuesta Nacional de Salud Familiar (FESAL) 1993 (Others Indirect)
Encuesta Nacional de Salud Familiar (FESAL) 1998 (Others Direct)
Encuesta Nacional de Salud Familiar (FESAL) 1998 (Others Indirect)
Encuesta Nacional de Salud Familiar (FESAL) 2002−2003 (Others Direct)
Censo 2007 (Census Indirect)
Encuesta Nacional de Salud Familiar (FESAL) 2008 (Others Direct)
VR Vital Registration data from Informe de Labores Ministerio de Salud
VR Vital Registration data from Informe de Labores Ministerio de Salud
VR WHO

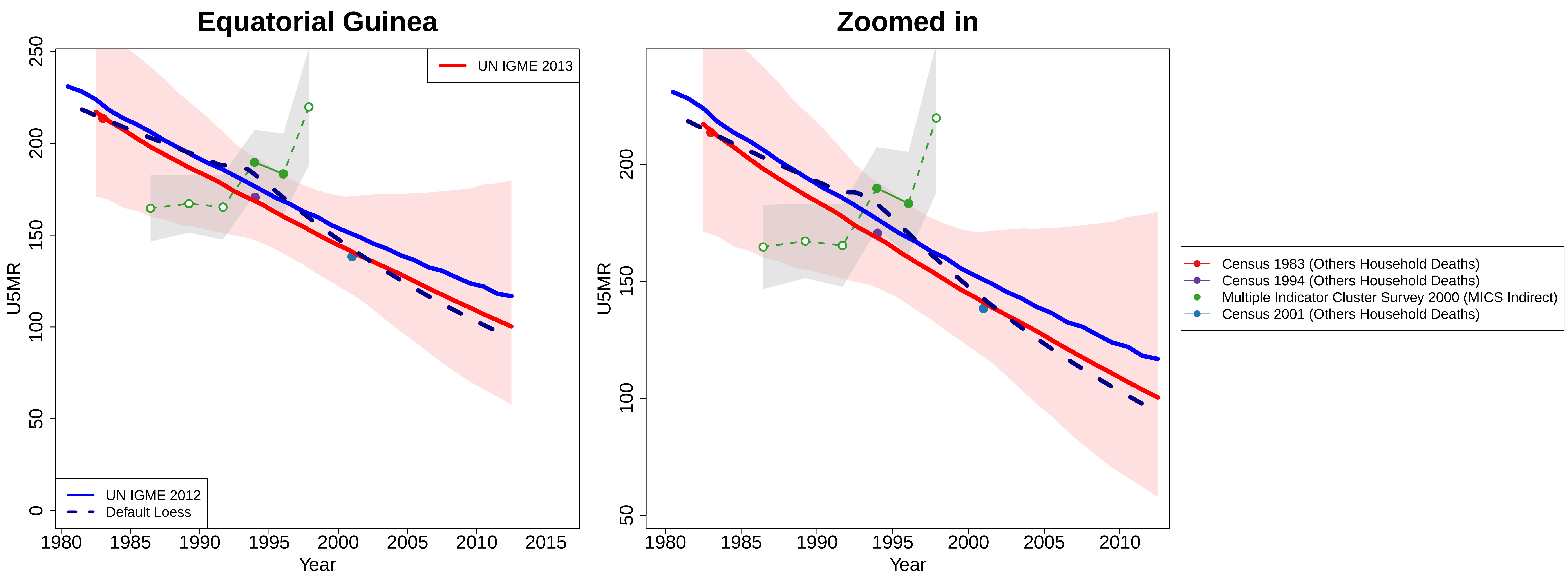

Equatorial Guinea
Zoomed in
UN IGME 2013
UN IGME 2012
Default Loess
Census 1983 (Others Household Deaths)
Census 1994 (Others Household Deaths)
Multiple Indicator Cluster Survey 2000 (MICS Indirect)
Census 2001 (Others Household Deaths)
U5MR
Year
0
50
100
150
200
250
1980
1985
1990
1995
2000
2005
2010
2015

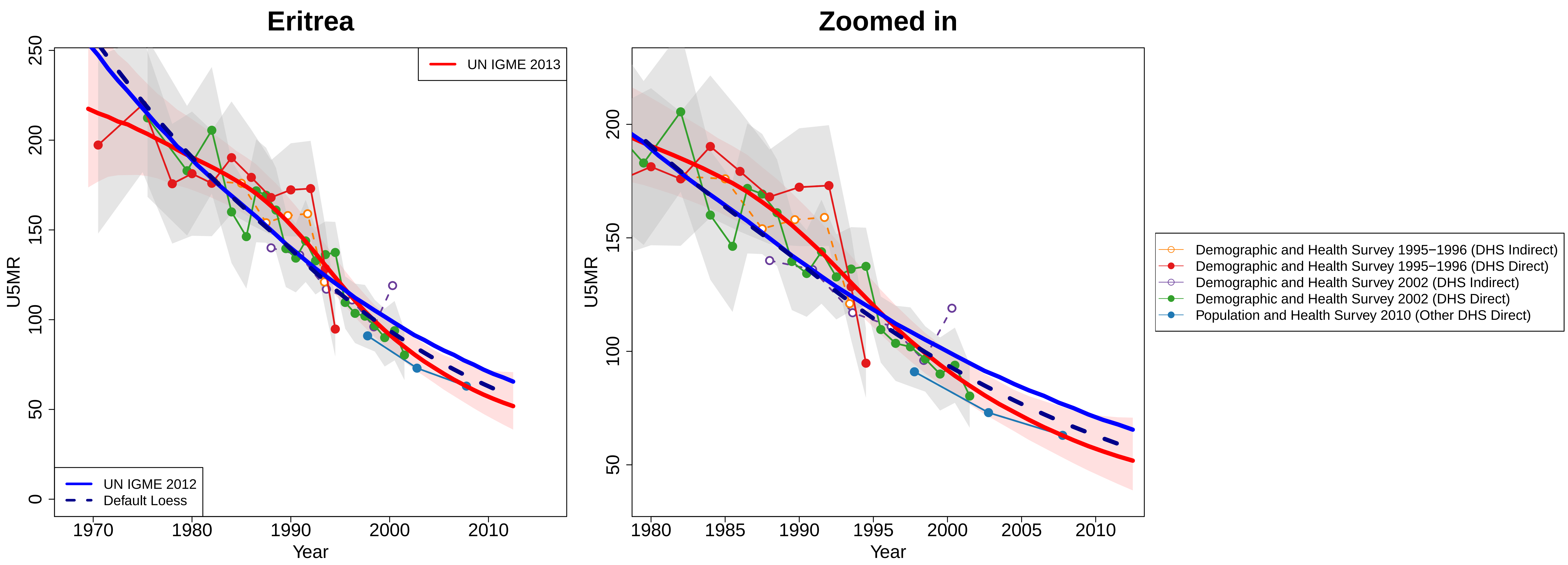

Eritrea
Zoomed in
U5MR
Year
UN IGME 2013
UN IGME 2012
Default Loess
Demographic and Health Survey 1995−1996 (DHS Indirect)
Demographic and Health Survey 1995−1996 (DHS Direct)
Demographic and Health Survey 2002 (DHS Indirect)
Demographic and Health Survey 2002 (DHS Direct)
Population and Health Survey 2010 (Other DHS Direct)
0
50
100
150
200
250
1970
1980
1990
2000
2010
1985
1995
2005

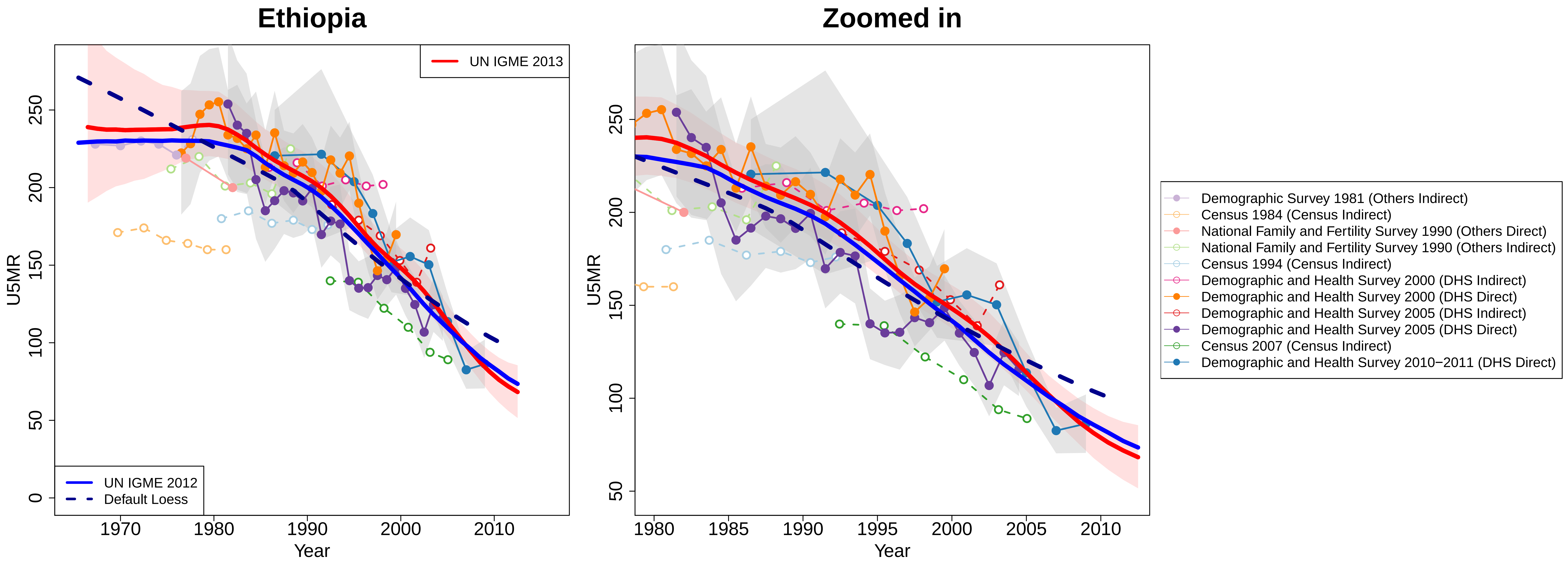

Ethiopia
Zoomed in
U5MR
Year
UN IGME 2013
UN IGME 2012
Default Loess
Demographic Survey 1981 (Others Indirect)
Census 1984 (Census Indirect)
National Family and Fertility Survey 1990 (Others Direct)
National Family and Fertility Survey 1990 (Others Indirect)
Census 1994 (Census Indirect)
Demographic and Health Survey 2000 (DHS Indirect)
Demographic and Health Survey 2000 (DHS Direct)
Demographic and Health Survey 2005 (DHS Indirect)
Demographic and Health Survey 2005 (DHS Direct)
Census 2007 (Census Indirect)
Demographic and Health Survey 2010−2011 (DHS Direct)

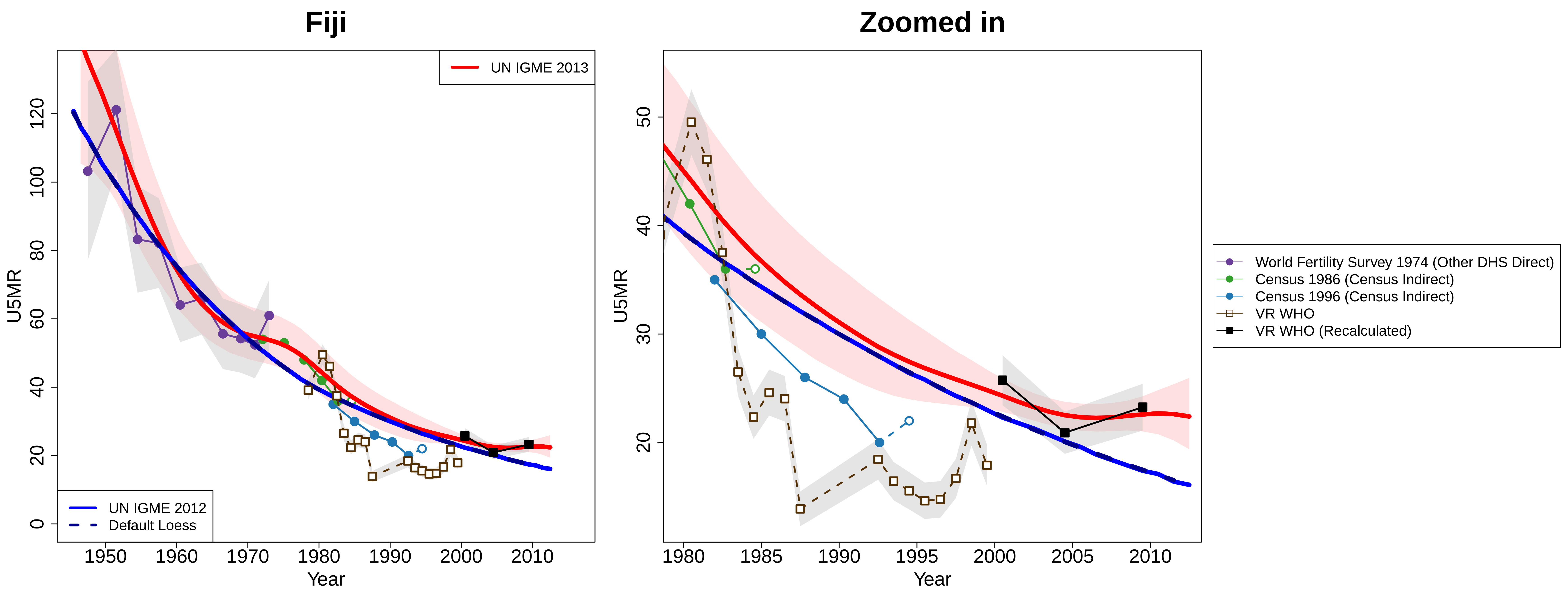

Fiji
Zoomed in
UN IGME 2013
UN IGME 2012
Default Loess
U5MR
Year
World Fertility Survey 1974 (Other DHS Direct)
Census 1986 (Census Indirect)
Census 1996 (Census Indirect)
VR WHO
VR WHO (Recalculated)

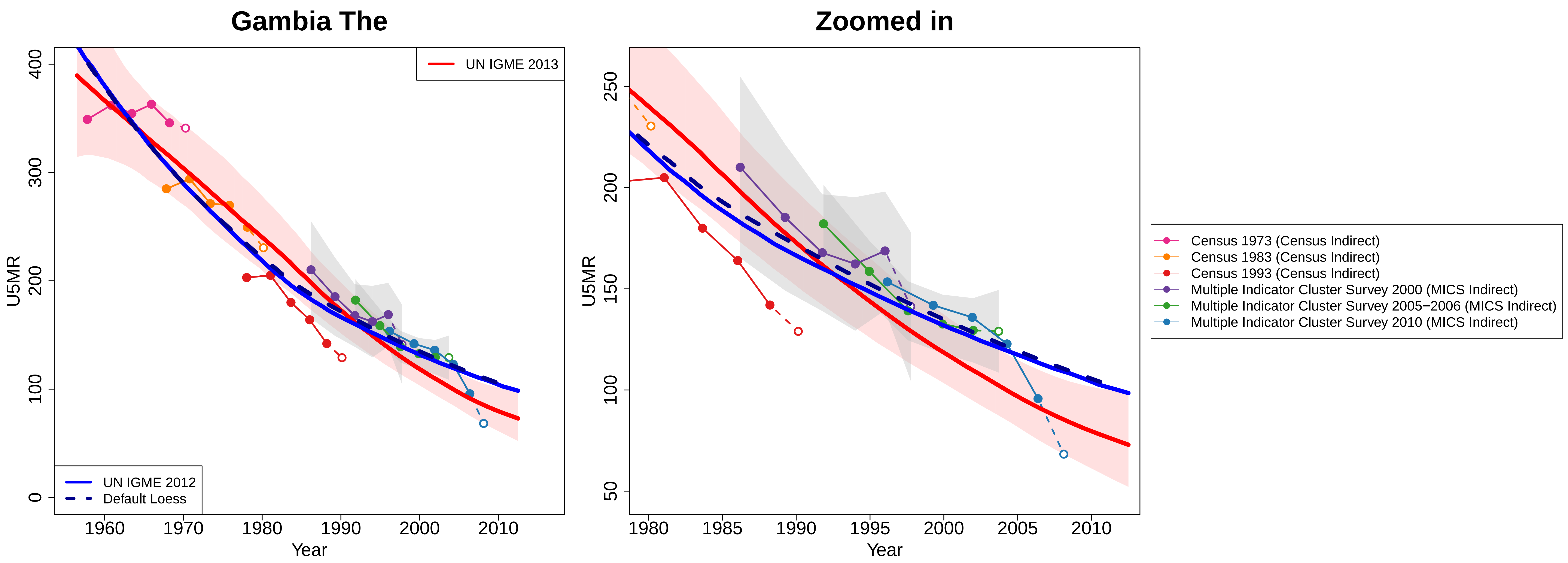

Gambia The
Zoomed in
UN IGME 2013
UN IGME 2012
Default Loess
U5MR
Year
Census 1973 (Census Indirect)
Census 1983 (Census Indirect)
Census 1993 (Census Indirect)
Multiple Indicator Cluster Survey 2000 (MICS Indirect)
Multiple Indicator Cluster Survey 2005−2006 (MICS Indirect)
Multiple Indicator Cluster Survey 2010 (MICS Indirect)

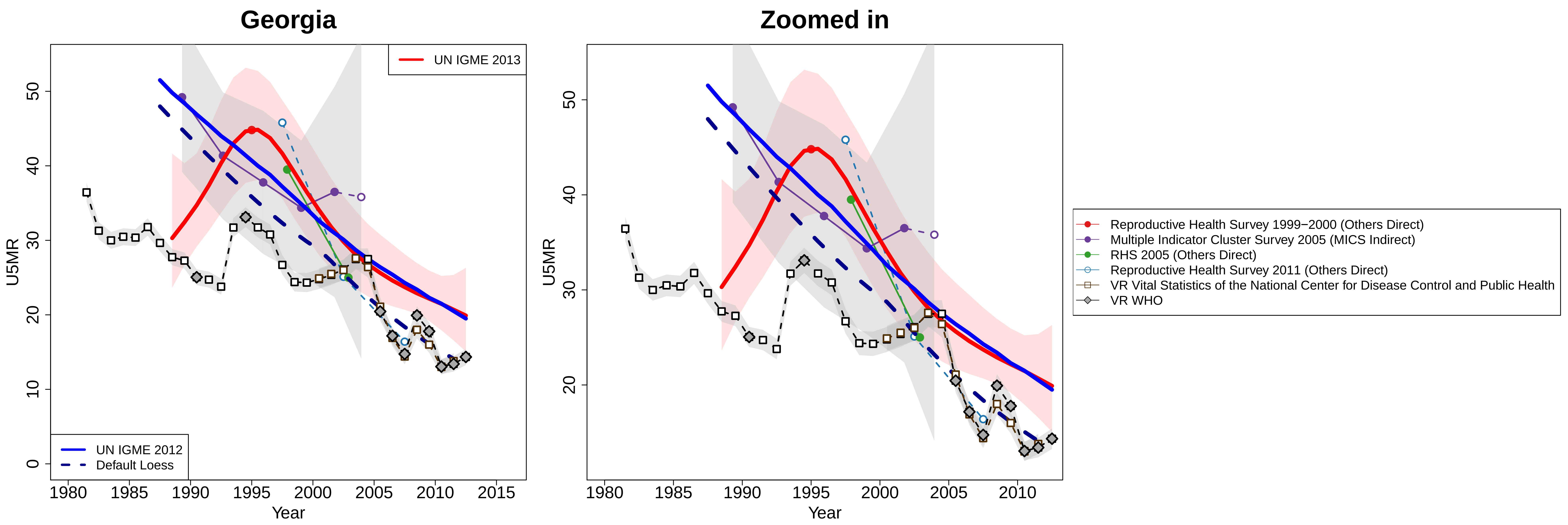

Georgia
Zoomed in
UN IGME 2013
UN IGME 2012
Default Loess
U5MR
Year
Reproductive Health Survey 1999−2000 (Others Direct)
Multiple Indicator Cluster Survey 2005 (MICS Indirect)
RHS 2005 (Others Direct)
Reproductive Health Survey 2011 (Others Direct)
VR Vital Statistics of the National Center for Disease Control and Public Health
VR WHO

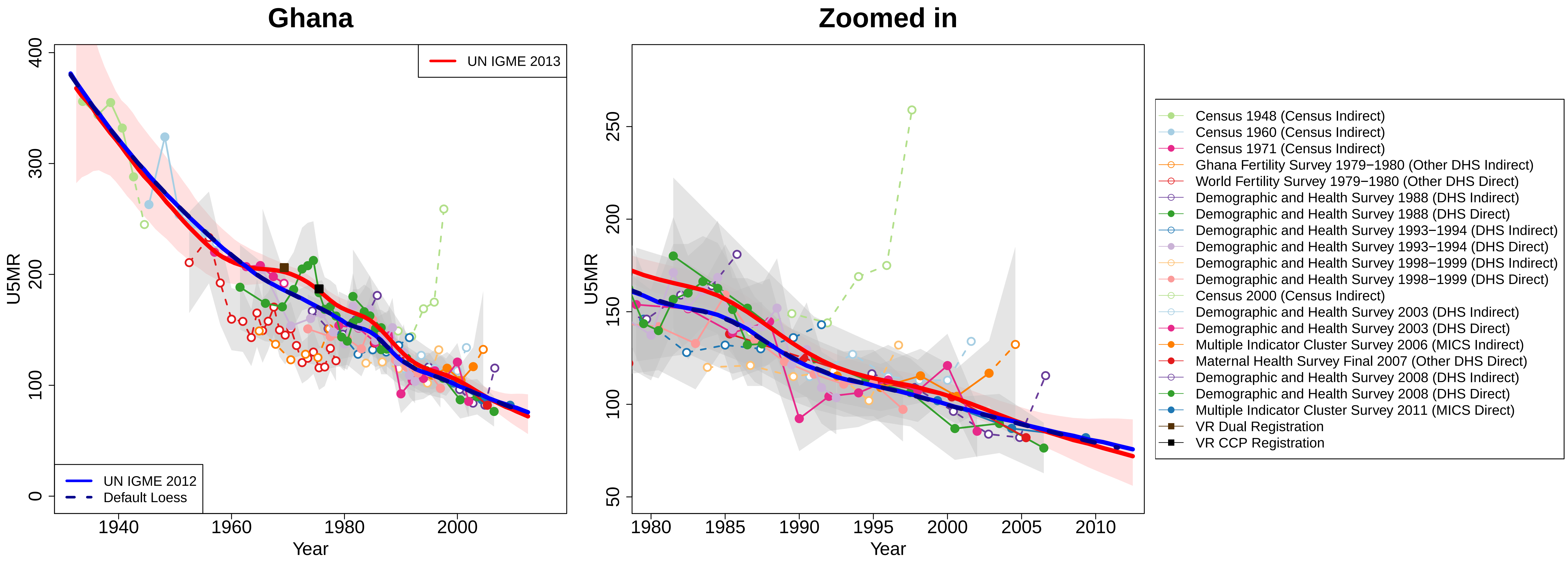
Ghana
Zoomed in
U5MR
Year
0
100
200
300
400
1940
1960
1980
2000
50
150
250
1980
1985
1990
1995
2005
2010
UN IGME 2013
UN IGME 2012
Default Loess
Census 1948 (Census Indirect)
Census 1960 (Census Indirect)
Census 1971 (Census Indirect)
Ghana Fertility Survey 1979−1980 (Other DHS Indirect)
World Fertility Survey 1979−1980 (Other DHS Direct)
Demographic and Health Survey 1988 (DHS Indirect)
Demographic and Health Survey 1988 (DHS Direct)
Demographic and Health Survey 1993−1994 (DHS Indirect)
Demographic and Health Survey 1993−1994 (DHS Direct)
Demographic and Health Survey 1998−1999 (DHS Indirect)
Demographic and Health Survey 1998−1999 (DHS Direct)
Census 2000 (Census Indirect)
Demographic and Health Survey 2003 (DHS Indirect)
Demographic and Health Survey 2003 (DHS Direct)
Multiple Indicator Cluster Survey 2006 (MICS Indirect)
Maternal Health Survey Final 2007 (Other DHS Direct)
Demographic and Health Survey 2008 (DHS Indirect)
Demographic and Health Survey 2008 (DHS Direct)
Multiple Indicator Cluster Survey 2011 (MICS Direct)
VR Dual Registration
VR CCP Registration

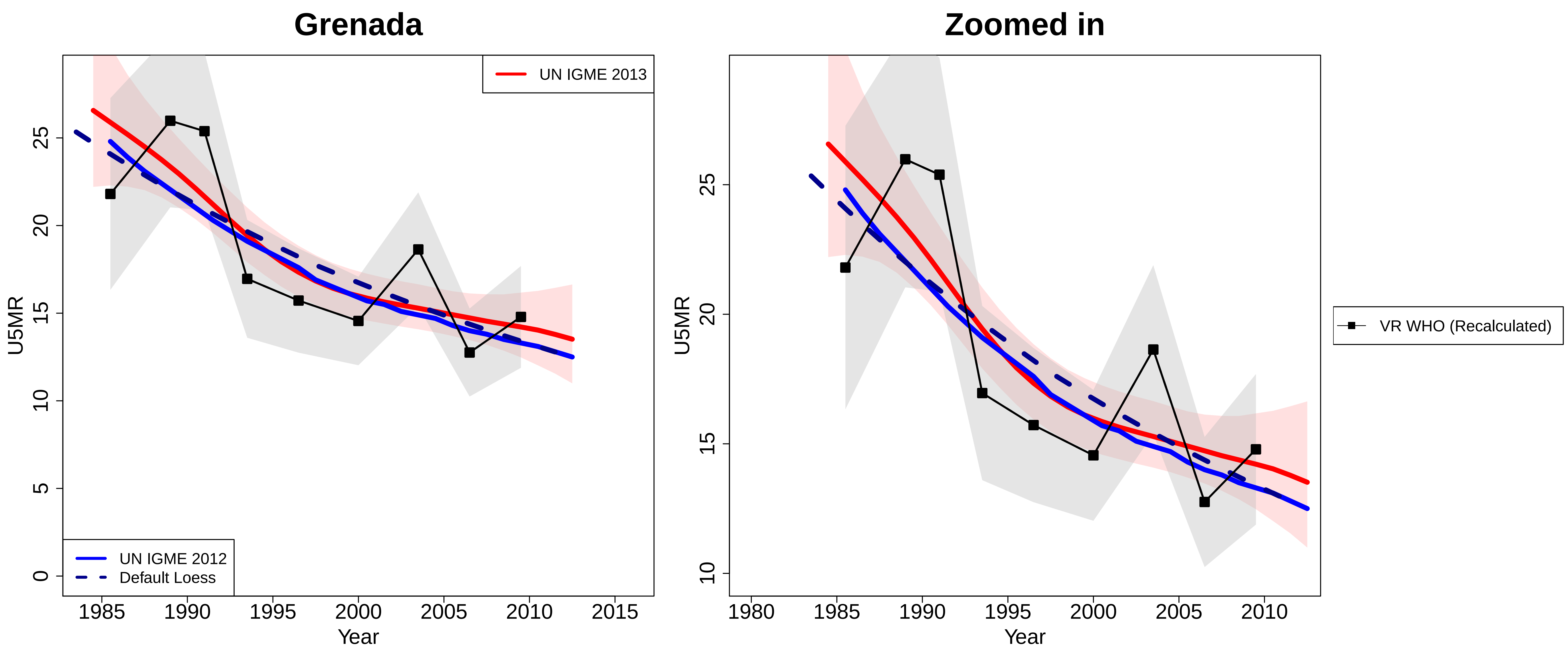

Grenada
Zoomed in
UN IGME 2013
UN IGME 2012
Default Loess
VR WHO (Recalculated)
U5MR
Year

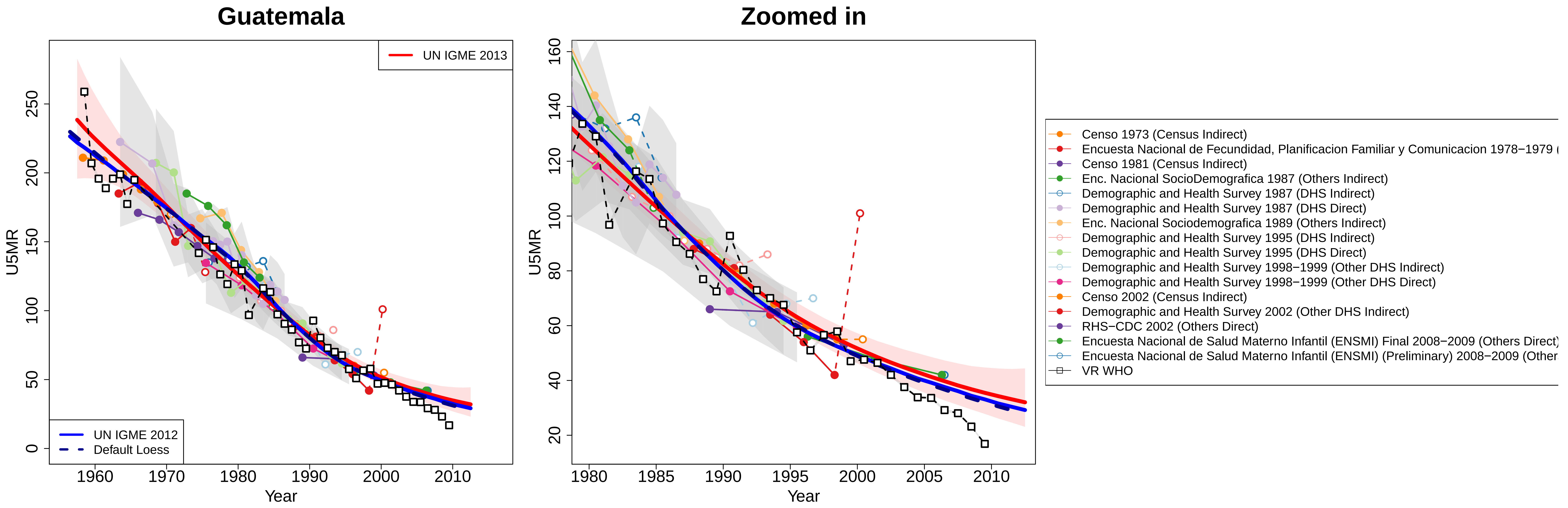
Guatemala
Zoomed in
U5MR
Year
UN IGME 2013
UN IGME 2012
Default Loess
Censo 1973 (Census Indirect)
Encuesta Nacional de Fecundidad, Planificacion Familiar y Comunicacion 1978−1979 (
Censo 1981 (Census Indirect)
Enc. Nacional SocioDemografica 1987 (Others Indirect)
Demographic and Health Survey 1987 (DHS Indirect)
Demographic and Health Survey 1987 (DHS Direct)
Enc. Nacional Sociodemografica 1989 (Others Indirect)
Demographic and Health Survey 1995 (DHS Indirect)
Demographic and Health Survey 1995 (DHS Direct)
Demographic and Health Survey 1998−1999 (Other DHS Indirect)
Demographic and Health Survey 1998−1999 (Other DHS Direct)
Censo 2002 (Census Indirect)
Demographic and Health Survey 2002 (Other DHS Indirect)
RHS−CDC 2002 (Others Direct)
Encuesta Nacional de Salud Materno Infantil (ENSMI) Final 2008−2009 (Others Direct)
Encuesta Nacional de Salud Materno Infantil (ENSMI) (Preliminary) 2008−2009 (Other
VR WHO

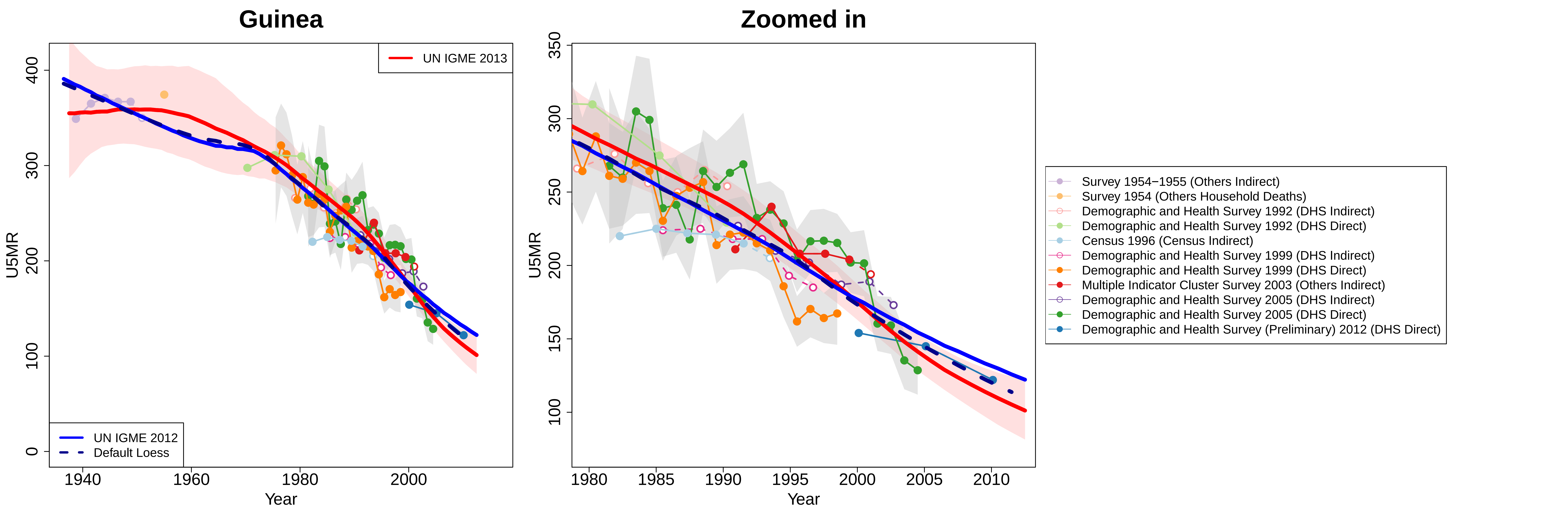
Guinea
Zoomed in
UN IGME 2013
UN IGME 2012
Default Loess
U5MR
Year
Survey 1954−1955 (Others Indirect)
Survey 1954 (Others Household Deaths)
Demographic and Health Survey 1992 (DHS Indirect)
Demographic and Health Survey 1992 (DHS Direct)
Census 1996 (Census Indirect)
Demographic and Health Survey 1999 (DHS Indirect)
Demographic and Health Survey 1999 (DHS Direct)
Multiple Indicator Cluster Survey 2003 (Others Indirect)
Demographic and Health Survey 2005 (DHS Indirect)
Demographic and Health Survey 2005 (DHS Direct)
Demographic and Health Survey (Preliminary) 2012 (DHS Direct)

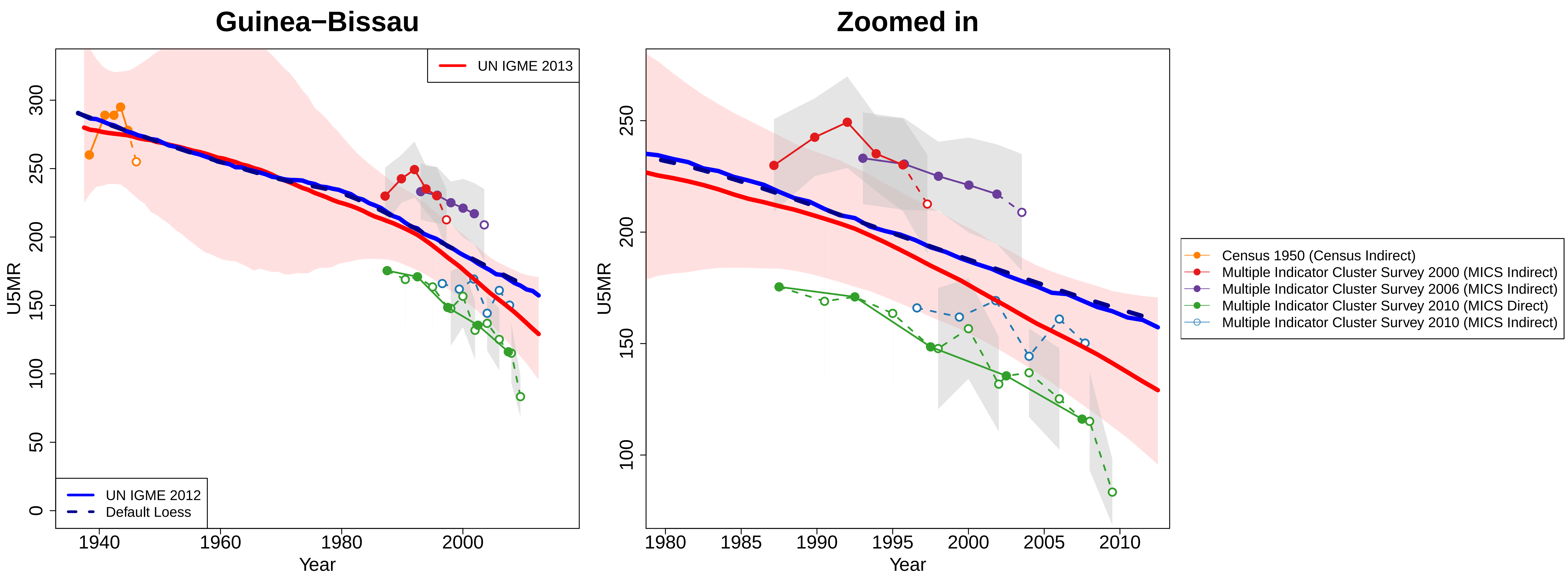

Guinea−Bissau
UN IGME 2013
UN IGME 2012
Default Loess
U5MR
Year
0
50
100
150
200
250
300
1940
1960
1980
2000
Zoomed in
U5MR
Year
100
150
200
250
1980
1985
1990
1995
2000
2005
2010
Census 1950 (Census Indirect)
Multiple Indicator Cluster Survey 2000 (MICS Indirect)
Multiple Indicator Cluster Survey 2006 (MICS Indirect)
Multiple Indicator Cluster Survey 2010 (MICS Direct)
Multiple Indicator Cluster Survey 2010 (MICS Indirect)

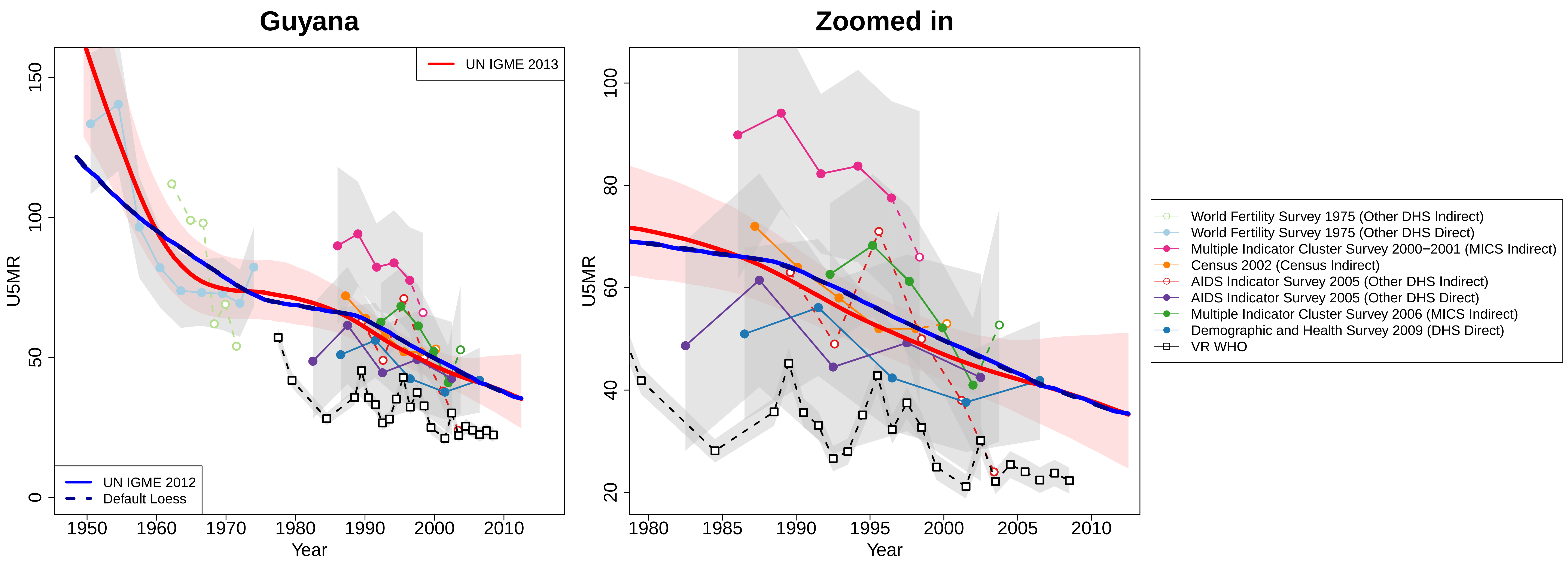

Guyana
Zoomed in
U5MR
Year
UN IGME 2013
UN IGME 2012
Default Loess
World Fertility Survey 1975 (Other DHS Indirect)
World Fertility Survey 1975 (Other DHS Direct)
Multiple Indicator Cluster Survey 2000−2001 (MICS Indirect)
Census 2002 (Census Indirect)
AIDS Indicator Survey 2005 (Other DHS Indirect)
AIDS Indicator Survey 2005 (Other DHS Direct)
Multiple Indicator Cluster Survey 2006 (MICS Indirect)
Demographic and Health Survey 2009 (DHS Direct)
VR WHO

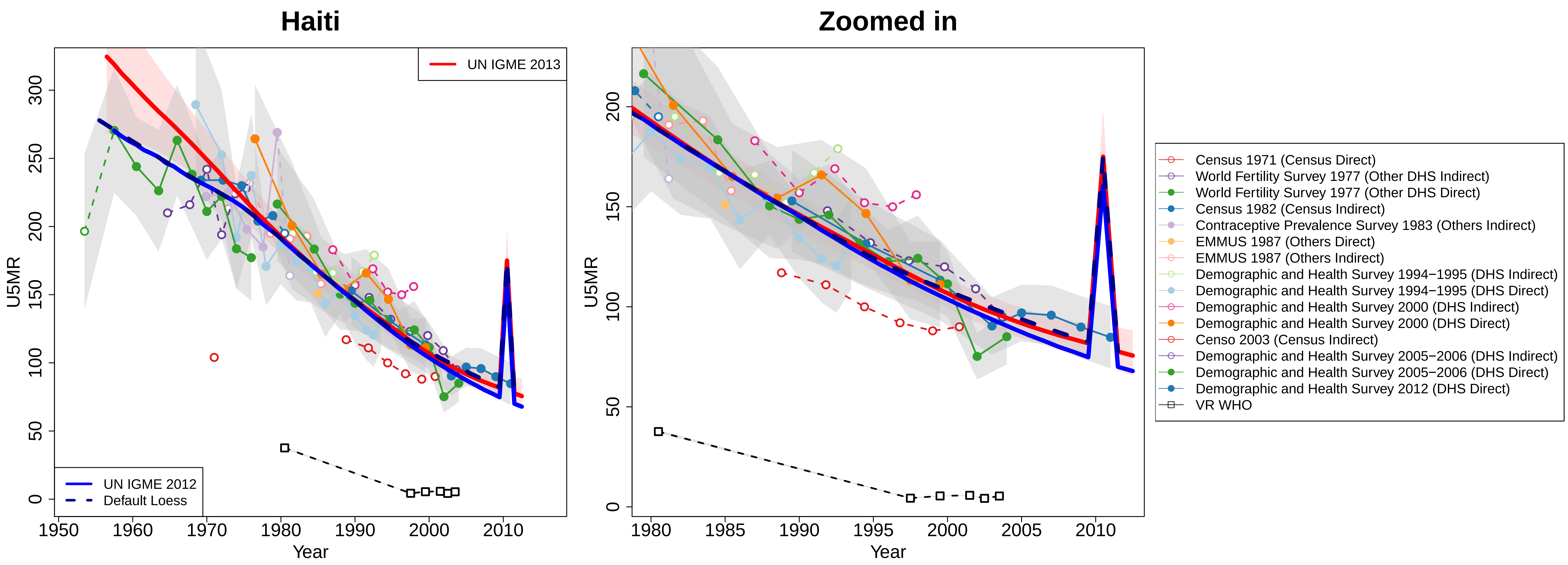

Haiti
Zoomed in
UN IGME 2013
UN IGME 2012
Default Loess
U5MR
Year
Census 1971 (Census Direct)
World Fertility Survey 1977 (Other DHS Indirect)
World Fertility Survey 1977 (Other DHS Direct)
Census 1982 (Census Indirect)
Contraceptive Prevalence Survey 1983 (Others Indirect)
EMMUS 1987 (Others Direct)
EMMUS 1987 (Others Indirect)
Demographic and Health Survey 1994−1995 (DHS Indirect)
Demographic and Health Survey 1994−1995 (DHS Direct)
Demographic and Health Survey 2000 (DHS Indirect)
Demographic and Health Survey 2000 (DHS Direct)
Censo 2003 (Census Indirect)
Demographic and Health Survey 2005−2006 (DHS Indirect)
Demographic and Health Survey 2005−2006 (DHS Direct)
Demographic and Health Survey 2012 (DHS Direct)
VR WHO

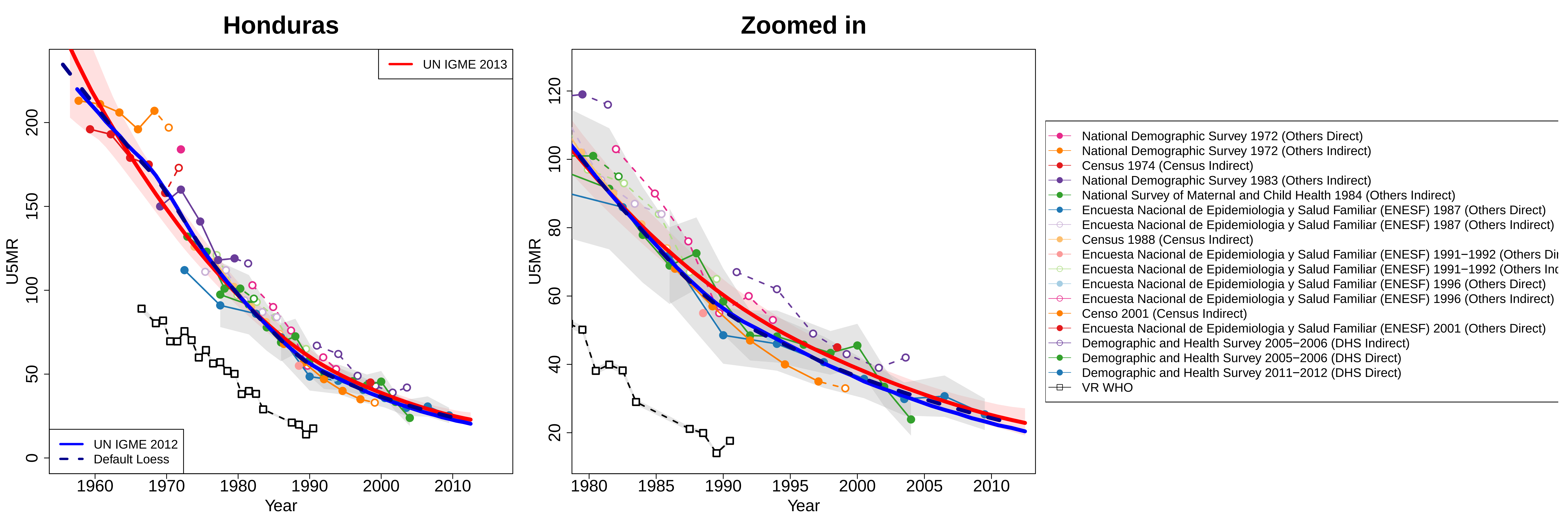

Honduras
Zoomed in
UN IGME 2013
UN IGME 2012
Default Loess
U5MR
Year
0
50
100
150
200
1960
1970
1980
1990
2000
2010
20
40
60
80
100
120
1980
1985
1990
1995
2000
2005
2010
National Demographic Survey 1972 (Others Direct)
National Demographic Survey 1972 (Others Indirect)
Census 1974 (Census Indirect)
National Demographic Survey 1983 (Others Indirect)
National Survey of Maternal and Child Health 1984 (Others Indirect)
Encuesta Nacional de Epidemiologia y Salud Familiar (ENESF) 1987 (Others Direct)
Encuesta Nacional de Epidemiologia y Salud Familiar (ENESF) 1987 (Others Indirect)
Census 1988 (Census Indirect)
Encuesta Nacional de Epidemiologia y Salud Familiar (ENESF) 1991−1992 (Others Di
Encuesta Nacional de Epidemiologia y Salud Familiar (ENESF) 1991−1992 (Others Inc
Encuesta Nacional de Epidemiologia y Salud Familiar (ENESF) 1996 (Others Direct)
Encuesta Nacional de Epidemiologia y Salud Familiar (ENESF) 1996 (Others Indirect)
Censo 2001 (Census Indirect)
Encuesta Nacional de Epidemiologia y Salud Familiar (ENESF) 2001 (Others Direct)
Demographic and Health Survey 2005−2006 (DHS Indirect)
Demographic and Health Survey 2005−2006 (DHS Direct)
Demographic and Health Survey 2011−2012 (DHS Direct)
VR WHO

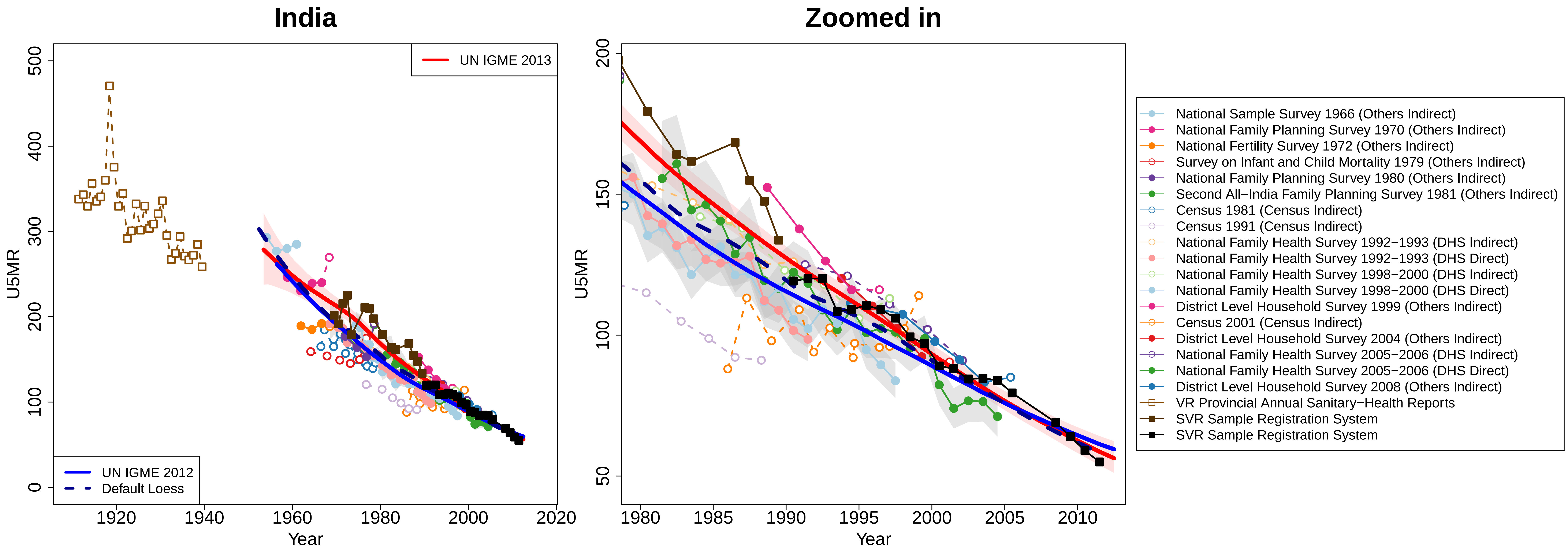

India
Zoomed in
UN IGME 2013
UN IGME 2012
Default Loess
U5MR
Year
National Sample Survey 1966 (Others Indirect)
National Family Planning Survey 1970 (Others Indirect)
National Fertility Survey 1972 (Others Indirect)
Survey on Infant and Child Mortality 1979 (Others Indirect)
National Family Planning Survey 1980 (Others Indirect)
Second All−India Family Planning Survey 1981 (Others Indirect)
Census 1981 (Census Indirect)
Census 1991 (Census Indirect)
National Family Health Survey 1992−1993 (DHS Indirect)
National Family Health Survey 1992−1993 (DHS Direct)
National Family Health Survey 1998−2000 (DHS Indirect)
National Family Health Survey 1998−2000 (DHS Direct)
District Level Household Survey 1999 (Others Indirect)
Census 2001 (Census Indirect)
District Level Household Survey 2004 (Others Indirect)
National Family Health Survey 2005−2006 (DHS Indirect)
National Family Health Survey 2005−2006 (DHS Direct)
District Level Household Survey 2008 (Others Indirect)
VR Provincial Annual Sanitary−Health Reports
SVR Sample Registration System
SVR Sample Registration System

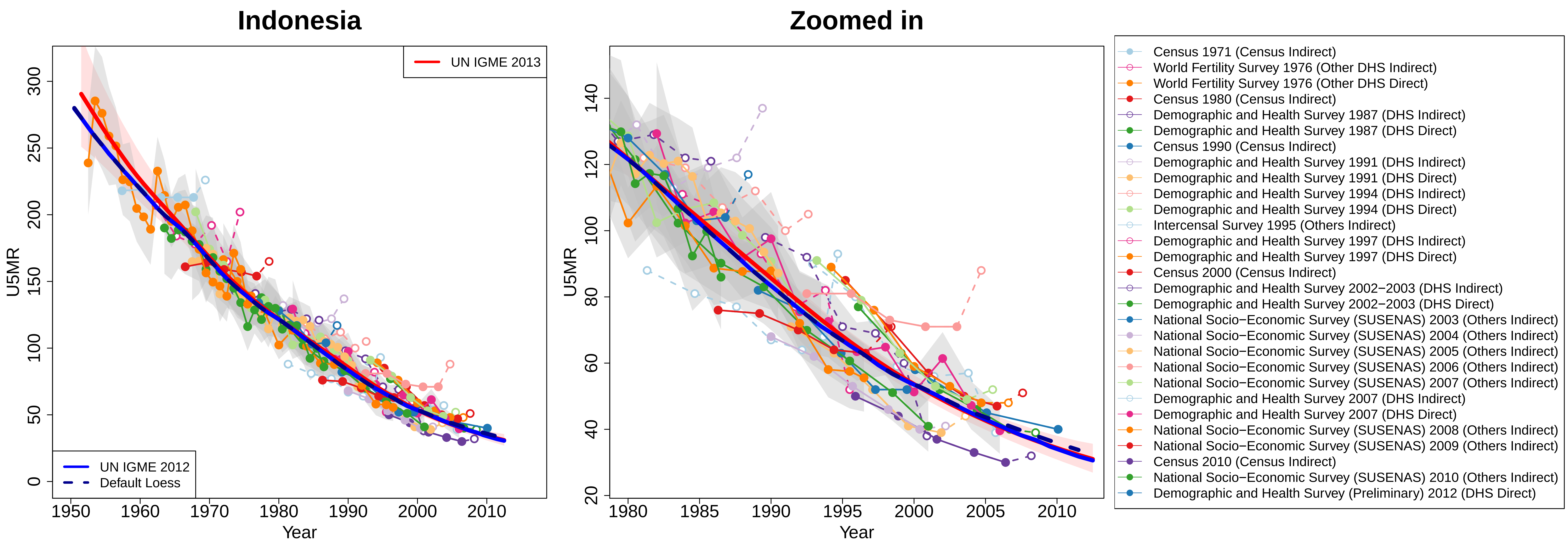
Indonesia
Zoomed in
UN IGME 2013
UN IGME 2012
Default Loess
U5MR
Year
Census 1971 (Census Indirect)
World Fertility Survey 1976 (Other DHS Indirect)
World Fertility Survey 1976 (Other DHS Direct)
Census 1980 (Census Indirect)
Demographic and Health Survey 1987 (DHS Indirect)
Demographic and Health Survey 1987 (DHS Direct)
Census 1990 (Census Indirect)
Demographic and Health Survey 1991 (DHS Indirect)
Demographic and Health Survey 1991 (DHS Direct)
Demographic and Health Survey 1994 (DHS Indirect)
Demographic and Health Survey 1994 (DHS Direct)
Intercensal Survey 1995 (Others Indirect)
Demographic and Health Survey 1997 (DHS Indirect)
Demographic and Health Survey 1997 (DHS Direct)
Census 2000 (Census Indirect)
Demographic and Health Survey 2002−2003 (DHS Indirect)
Demographic and Health Survey 2002−2003 (DHS Direct)
National Socio−Economic Survey (SUSENAS) 2003 (Others Indirect)
National Socio−Economic Survey (SUSENAS) 2004 (Others Indirect)
National Socio−Economic Survey (SUSENAS) 2005 (Others Indirect)
National Socio−Economic Survey (SUSENAS) 2006 (Others Indirect)
National Socio−Economic Survey (SUSENAS) 2007 (Others Indirect)
Demographic and Health Survey 2007 (DHS Indirect)
Demographic and Health Survey 2007 (DHS Direct)
National Socio−Economic Survey (SUSENAS) 2008 (Others Indirect)
National Socio−Economic Survey (SUSENAS) 2009 (Others Indirect)
Census 2010 (Census Indirect)
National Socio−Economic Survey (SUSENAS) 2010 (Others Indirect)
Demographic and Health Survey (Preliminary) 2012 (DHS Direct)

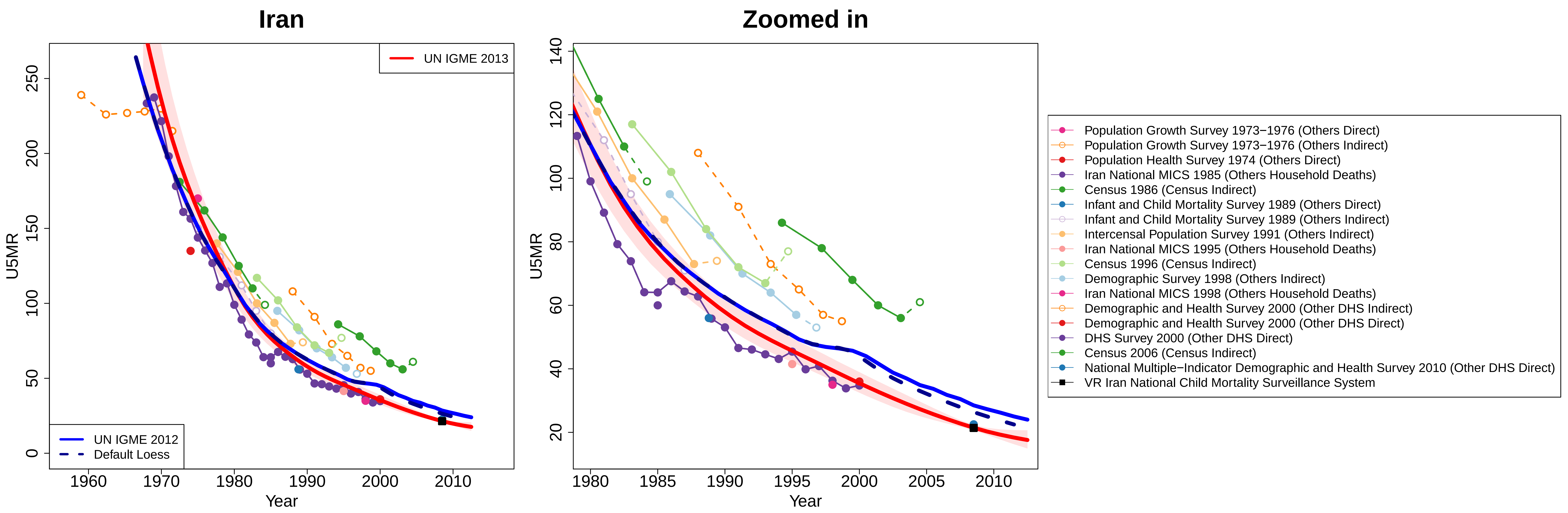

Iran
Zoomed in
U5MR
Year
UN IGME 2013
UN IGME 2012
Default Loess
Population Growth Survey 1973−1976 (Others Direct)
Population Growth Survey 1973−1976 (Others Indirect)
Population Health Survey 1974 (Others Direct)
Iran National MICS 1985 (Others Household Deaths)
Census 1986 (Census Indirect)
Infant and Child Mortality Survey 1989 (Others Direct)
Infant and Child Mortality Survey 1989 (Others Indirect)
Intercensal Population Survey 1991 (Others Indirect)
Iran National MICS 1995 (Others Household Deaths)
Census 1996 (Census Indirect)
Demographic Survey 1998 (Others Indirect)
Iran National MICS 1998 (Others Household Deaths)
Demographic and Health Survey 2000 (Other DHS Indirect)
Demographic and Health Survey 2000 (Other DHS Direct)
DHS Survey 2000 (Other DHS Direct)
Census 2006 (Census Indirect)
National Multiple−Indicator Demographic and Health Survey 2010 (Other DHS Direct)
VR Iran National Child Mortality Surveillance System

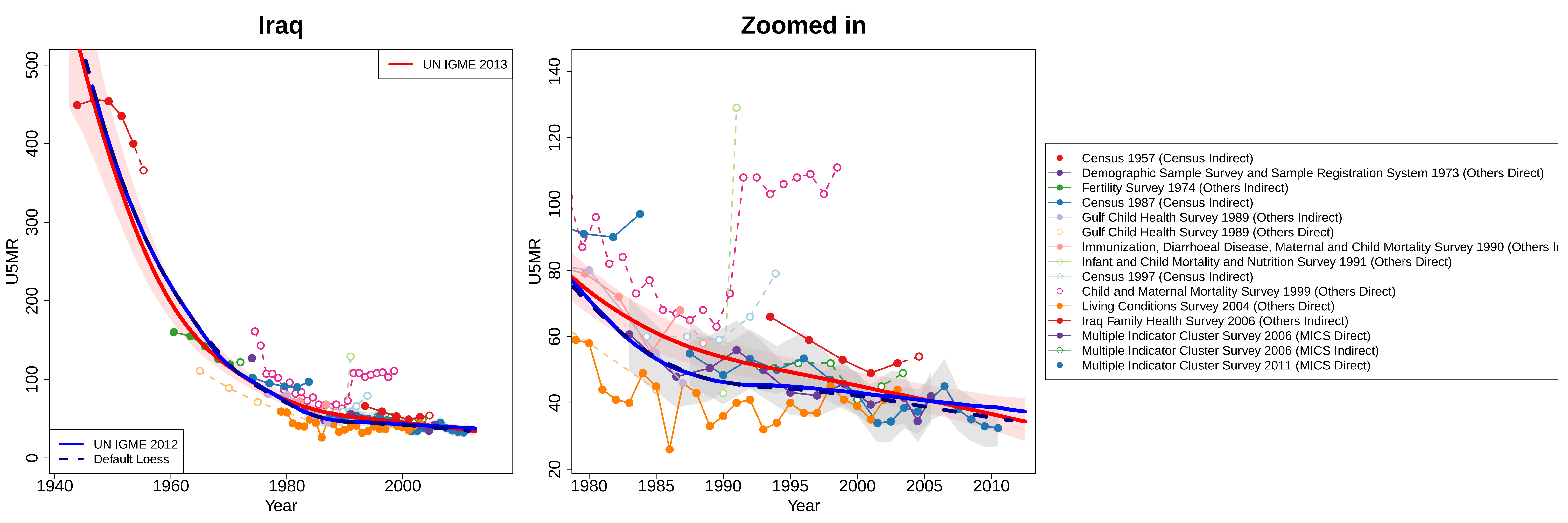

Iraq
Zoomed in
UN IGME 2013
UN IGME 2012
Default Loess
U5MR
Year
Census 1957 (Census Indirect)
Demographic Sample Survey and Sample Registration System 1973 (Others Direct)
Fertility Survey 1974 (Others Indirect)
Census 1987 (Census Indirect)
Gulf Child Health Survey 1989 (Others Indirect)
Gulf Child Health Survey 1989 (Others Direct)
Immunization, Diarrhoeal Disease, Maternal and Child Mortality Survey 1990 (Others I
Infant and Child Mortality and Nutrition Survey 1991 (Others Direct)
Census 1997 (Census Indirect)
Child and Maternal Mortality Survey 1999 (Others Direct)
Living Conditions Survey 2004 (Others Direct)
Iraq Family Health Survey 2006 (Others Indirect)
Multiple Indicator Cluster Survey 2006 (MICS Direct)
Multiple Indicator Cluster Survey 2006 (MICS Indirect)
Multiple Indicator Cluster Survey 2011 (MICS Direct)

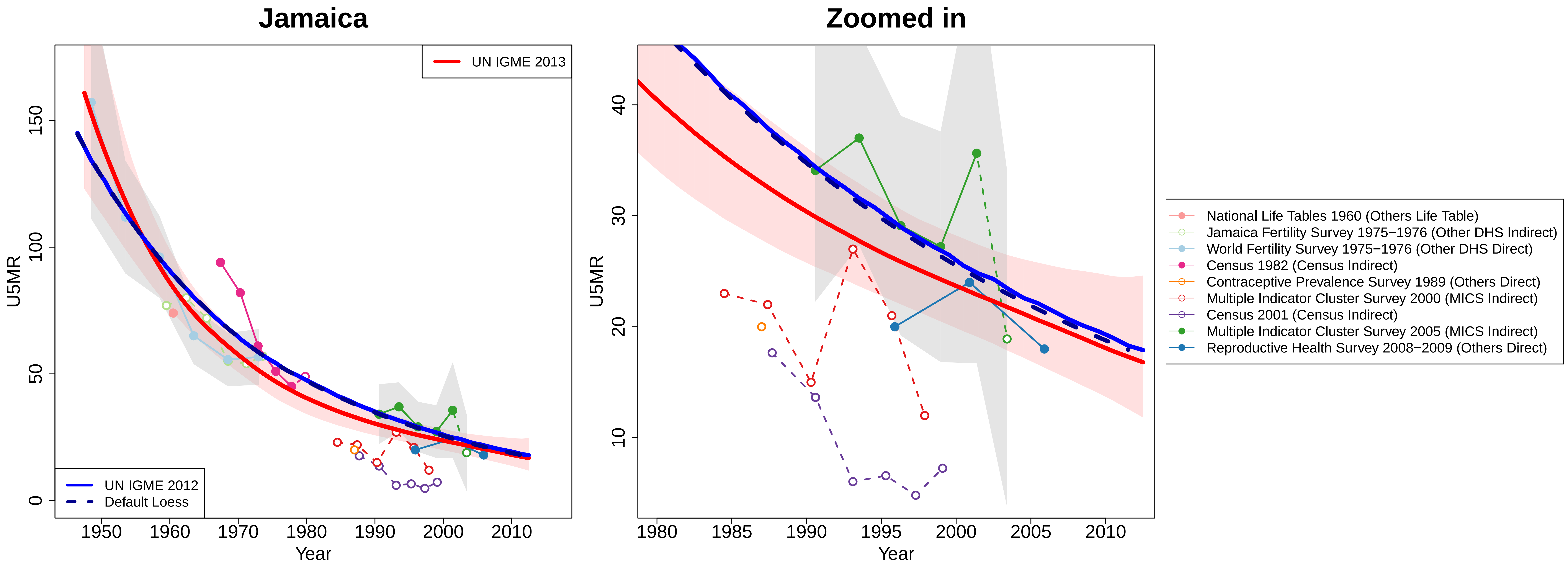
Jamaica
Zoomed in
UN IGME 2013
UN IGME 2012
Default Loess
U5MR
Year
National Life Tables 1960 (Others Life Table)
Jamaica Fertility Survey 1975−1976 (Other DHS Indirect)
World Fertility Survey 1975−1976 (Other DHS Direct)
Census 1982 (Census Indirect)
Contraceptive Prevalence Survey 1989 (Others Direct)
Multiple Indicator Cluster Survey 2000 (MICS Indirect)
Census 2001 (Census Indirect)
Multiple Indicator Cluster Survey 2005 (MICS Indirect)
Reproductive Health Survey 2008−2009 (Others Direct)

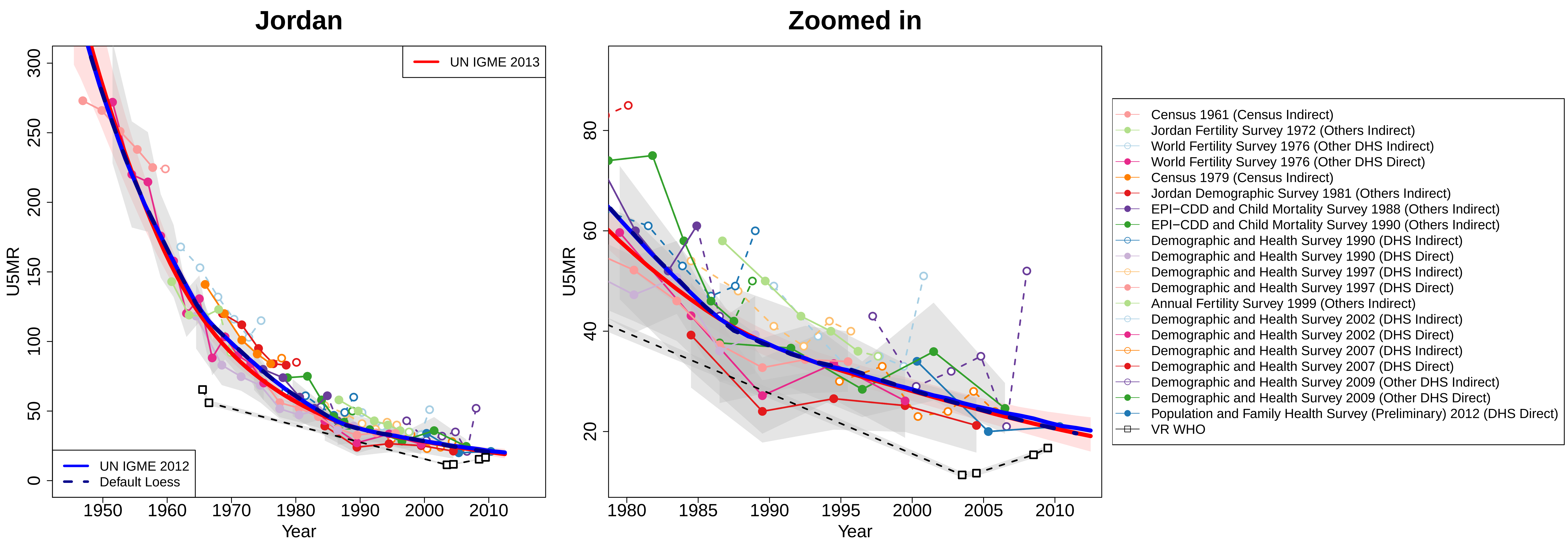

Jordan
Zoomed in
U5MR
Year
UN IGME 2013
UN IGME 2012
Default Loess
Census 1961 (Census Indirect)
Jordan Fertility Survey 1972 (Others Indirect)
World Fertility Survey 1976 (Other DHS Indirect)
World Fertility Survey 1976 (Other DHS Direct)
Census 1979 (Census Indirect)
Jordan Demographic Survey 1981 (Others Indirect)
EPI−CDD and Child Mortality Survey 1988 (Others Indirect)
EPI−CDD and Child Mortality Survey 1990 (Others Indirect)
Demographic and Health Survey 1990 (DHS Indirect)
Demographic and Health Survey 1990 (DHS Direct)
Demographic and Health Survey 1997 (DHS Indirect)
Demographic and Health Survey 1997 (DHS Direct)
Annual Fertility Survey 1999 (Others Indirect)
Demographic and Health Survey 2002 (DHS Indirect)
Demographic and Health Survey 2002 (DHS Direct)
Demographic and Health Survey 2007 (DHS Indirect)
Demographic and Health Survey 2007 (DHS Direct)
Demographic and Health Survey 2009 (Other DHS Indirect)
Demographic and Health Survey 2009 (Other DHS Direct)
Population and Family Health Survey (Preliminary) 2012 (DHS Direct)
VR WHO

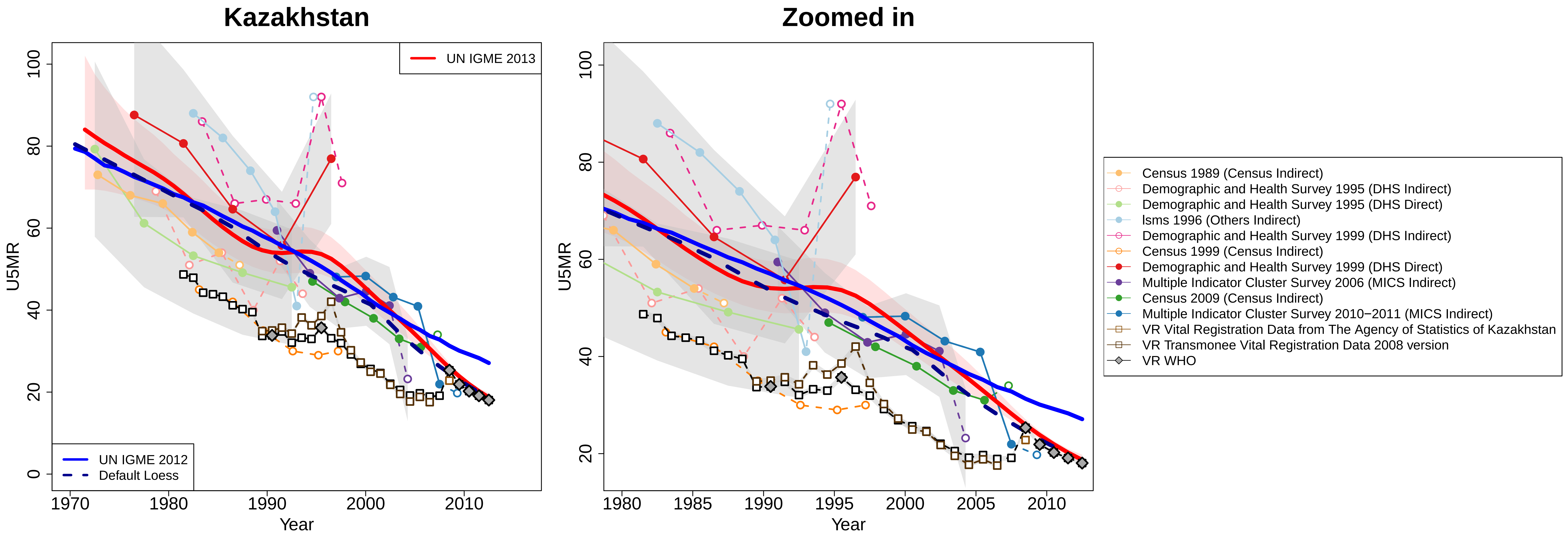
Kazakhstan
Zoomed in
UN IGME 2013
UN IGME 2012
Default Loess
U5MR
Year
Census 1989 (Census Indirect)
Demographic and Health Survey 1995 (DHS Indirect)
Demographic and Health Survey 1995 (DHS Direct)
Isms 1996 (Others Indirect)
Demographic and Health Survey 1999 (DHS Indirect)
Census 1999 (Census Indirect)
Demographic and Health Survey 1999 (DHS Direct)
Multiple Indicator Cluster Survey 2006 (MICS Indirect)
Census 2009 (Census Indirect)
Multiple Indicator Cluster Survey 2010−2011 (MICS Indirect)
VR Vital Registration Data from The Agency of Statistics of Kazakhstan
VR Transmonee Vital Registration Data 2008 version
VR WHO

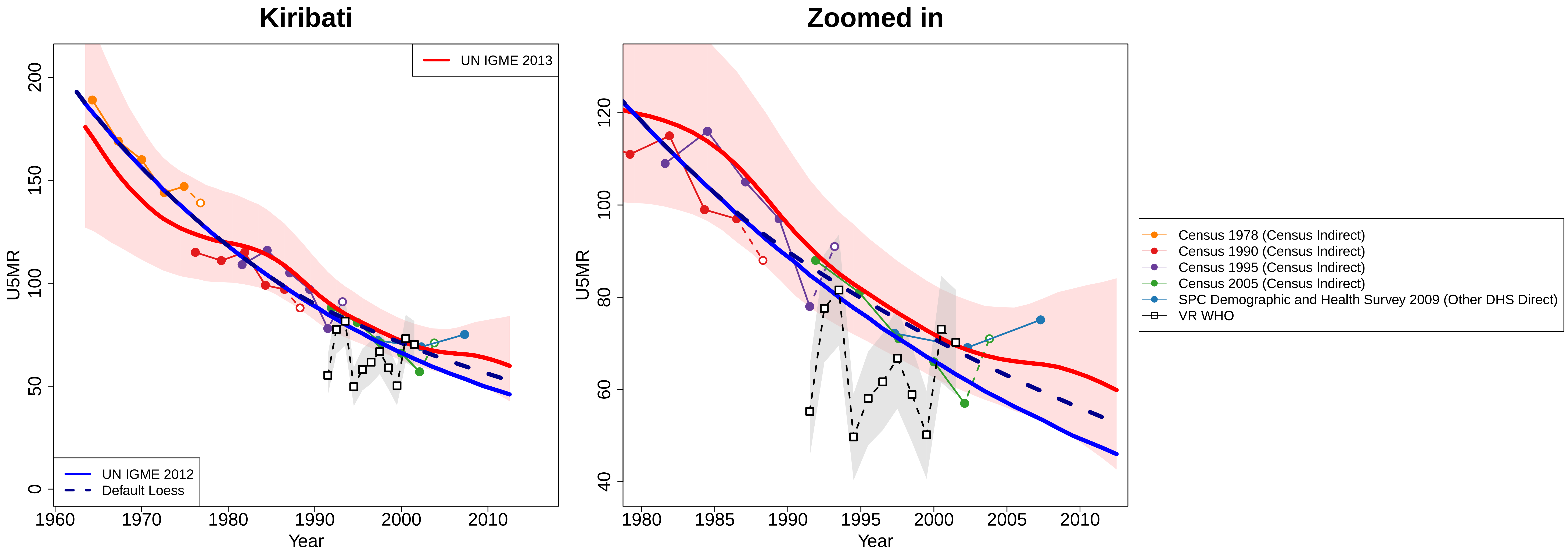

Kiribati
Zoomed in
UN IGME 2013
UN IGME 2012
Default Loess
U5MR
Year
Census 1978 (Census Indirect)
Census 1990 (Census Indirect)
Census 1995 (Census Indirect)
Census 2005 (Census Indirect)
SPC Demographic and Health Survey 2009 (Other DHS Direct)
VR WHO

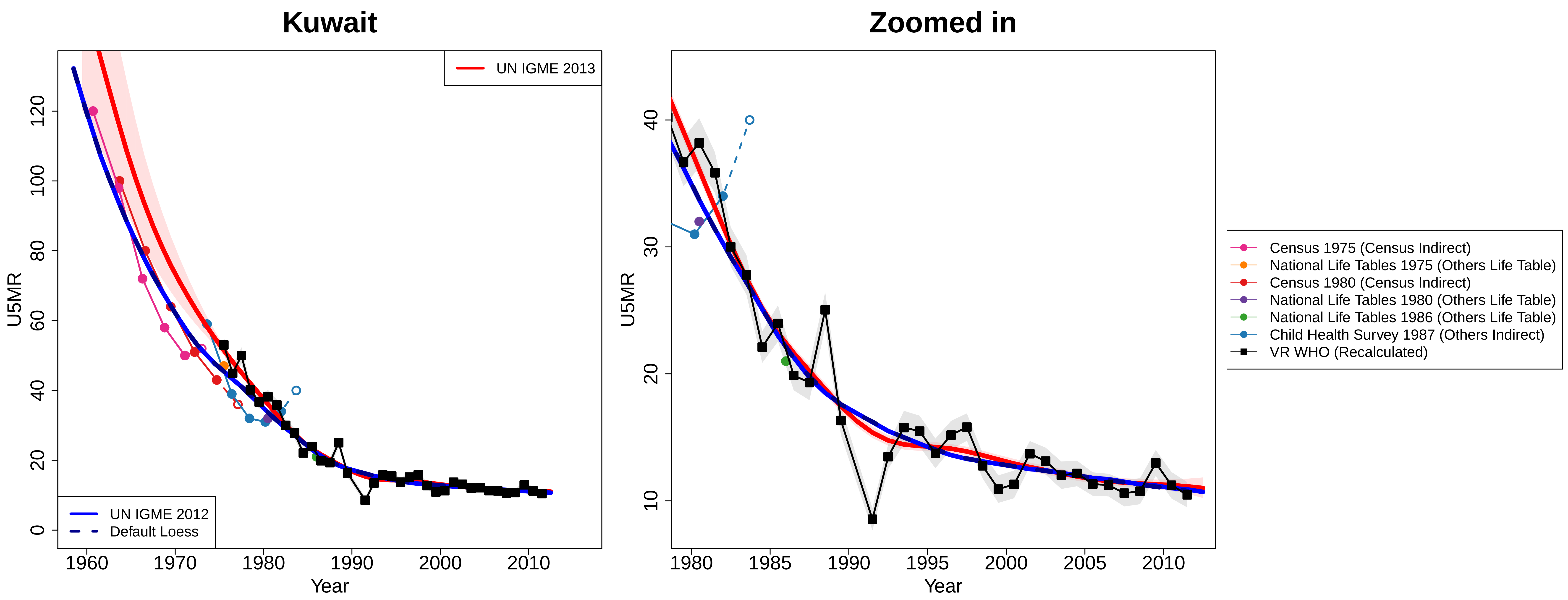

Kuwait
UN IGME 2013
UN IGME 2012
Default Loess
U5MR
Year
0
20
40
60
80
100
120
1960
1970
1980
1990
2000
2010
Zoomed in
U5MR
Year
10
20
30
40
1980
1985
1990
1995
2000
2005
2010
Census 1975 (Census Indirect)
National Life Tables 1975 (Others Life Table)
Census 1980 (Census Indirect)
National Life Tables 1980 (Others Life Table)
National Life Tables 1986 (Others Life Table)
Child Health Survey 1987 (Others Indirect)
VR WHO (Recalculated)

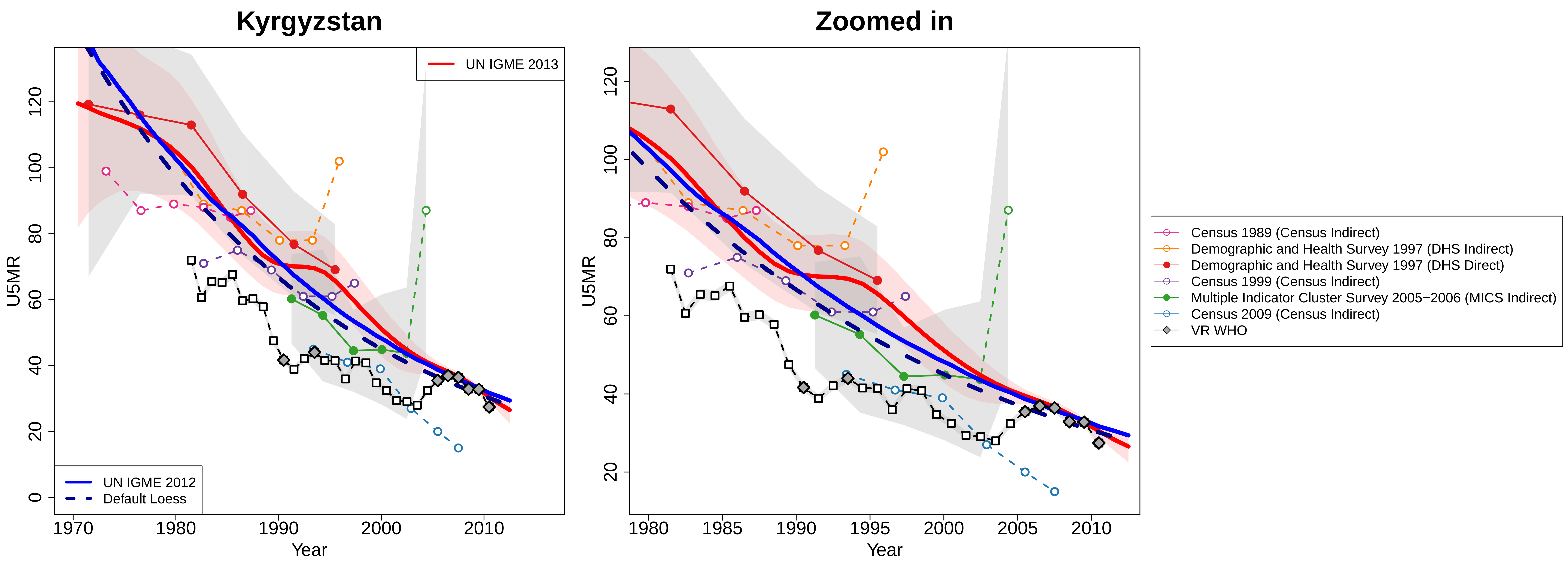
Kyrgyzstan
Zoomed in
UN IGME 2013
UN IGME 2012
Default Loess
U5MR
Year
Census 1989 (Census Indirect)
Demographic and Health Survey 1997 (DHS Indirect)
Demographic and Health Survey 1997 (DHS Direct)
Census 1999 (Census Indirect)
Multiple Indicator Cluster Survey 2005−2006 (MICS Indirect)
Census 2009 (Census Indirect)
VR WHO

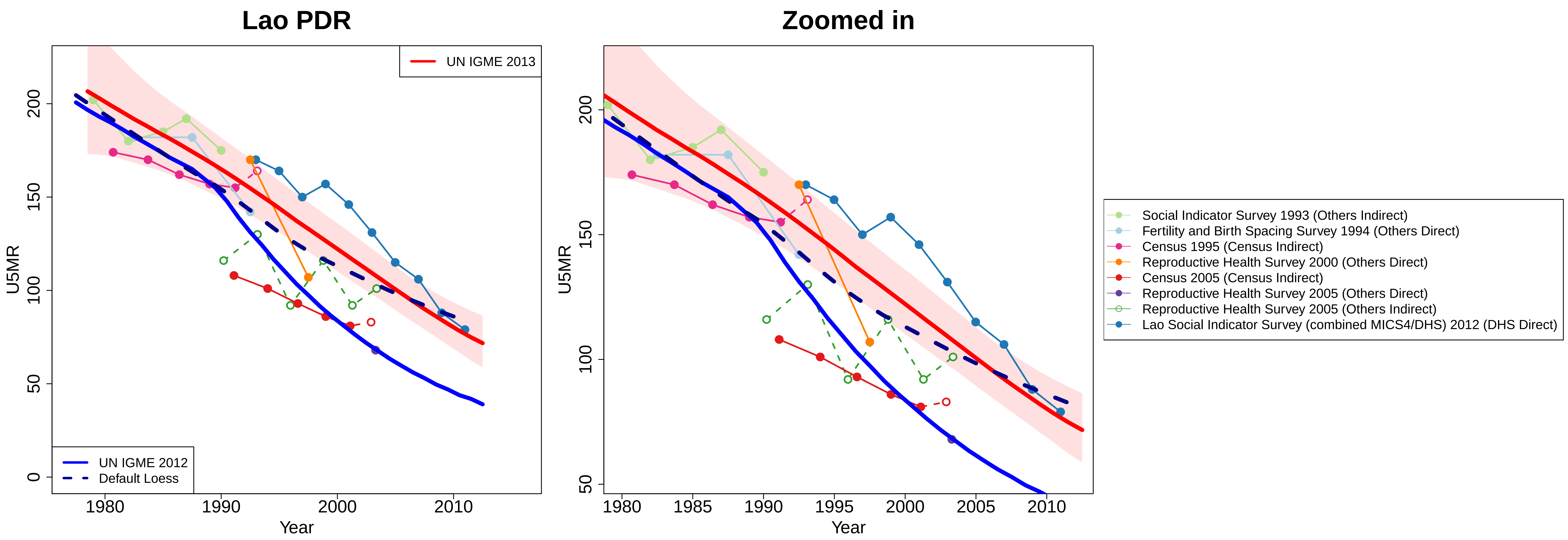

Lao PDR
Zoomed in
UN IGME 2013
UN IGME 2012
Default Loess
U5MR
Year
Social Indicator Survey 1993 (Others Indirect)
Fertility and Birth Spacing Survey 1994 (Others Direct)
Census 1995 (Census Indirect)
Reproductive Health Survey 2000 (Others Direct)
Census 2005 (Census Indirect)
Reproductive Health Survey 2005 (Others Direct)
Reproductive Health Survey 2005 (Others Indirect)
Lao Social Indicator Survey (combined MICS4/DHS) 2012 (DHS Direct)

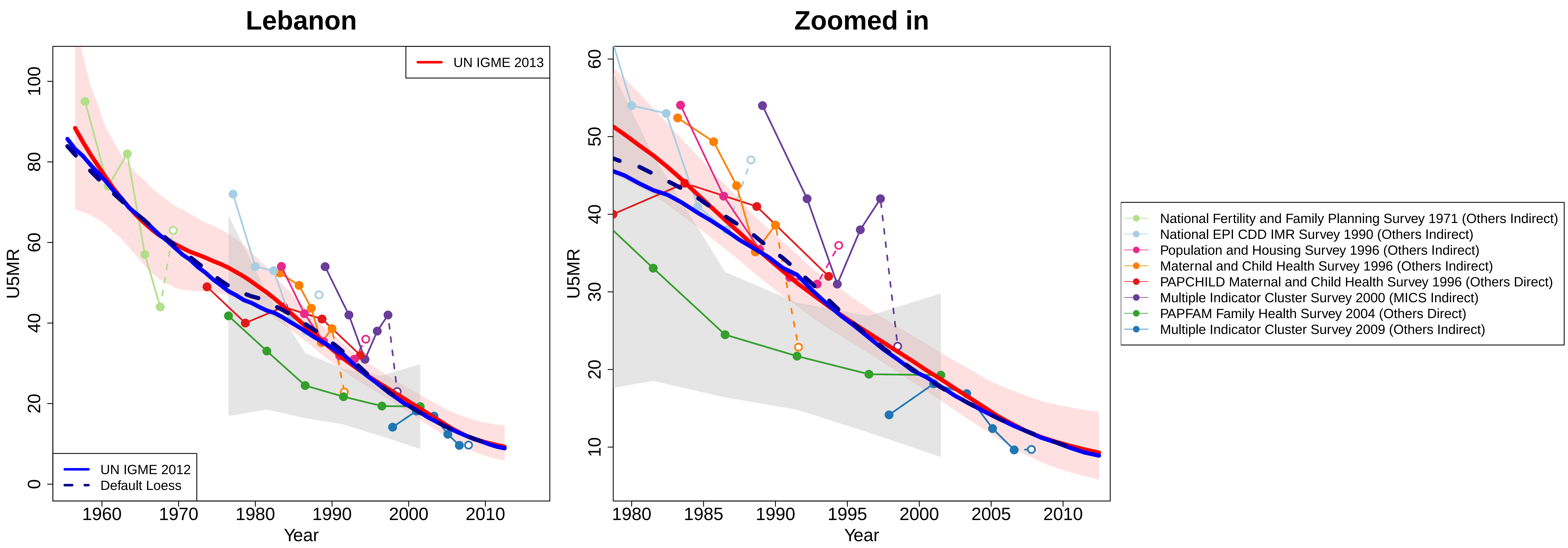
Lebanon
Zoomed in
UN IGME 2013
UN IGME 2012
Default Loess
U5MR
Year
National Fertility and Family Planning Survey 1971 (Others Indirect)
National EPI CDD IMR Survey 1990 (Others Indirect)
Population and Housing Survey 1996 (Others Indirect)
Maternal and Child Health Survey 1996 (Others Indirect)
PAPCHILD Maternal and Child Health Survey 1996 (Others Direct)
Multiple Indicator Cluster Survey 2000 (MICS Indirect)
PAPFAM Family Health Survey 2004 (Others Direct)
Multiple Indicator Cluster Survey 2009 (Others Indirect)

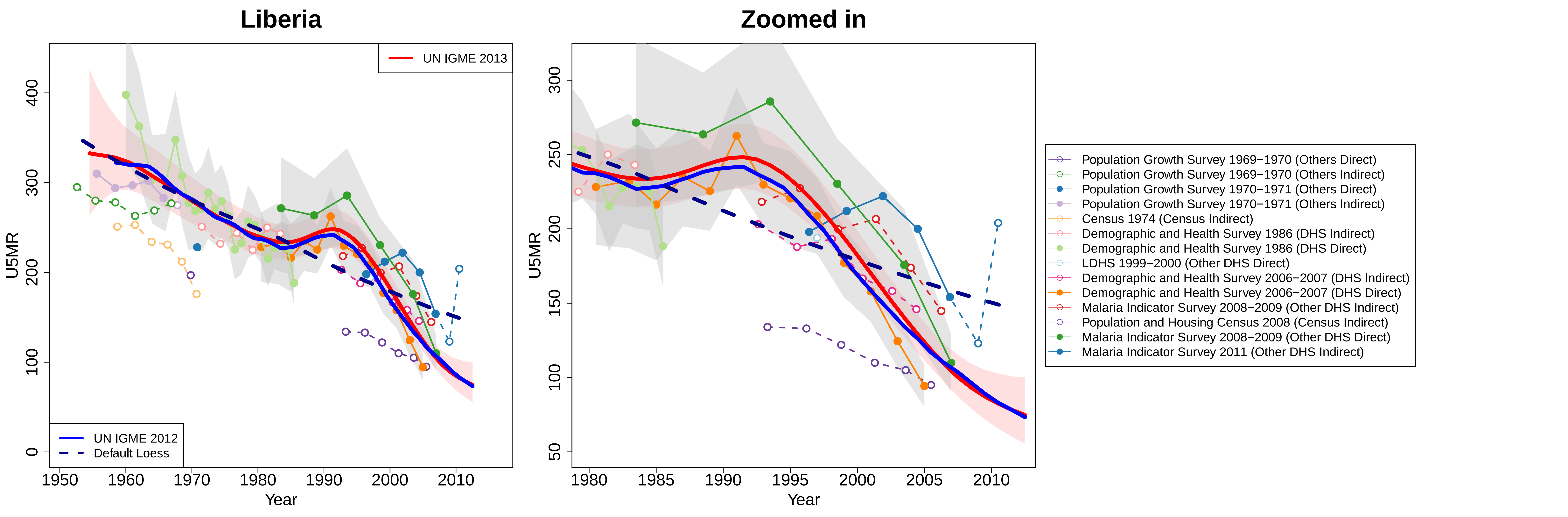

Liberia
Zoomed in
UN IGME 2013
UN IGME 2012
Default Loess
U5MR
Year
Population Growth Survey 1969−1970 (Others Direct)
Population Growth Survey 1969−1970 (Others Indirect)
Population Growth Survey 1970−1971 (Others Direct)
Population Growth Survey 1970−1971 (Others Indirect)
Census 1974 (Census Indirect)
Demographic and Health Survey 1986 (DHS Indirect)
Demographic and Health Survey 1986 (DHS Direct)
LDHS 1999−2000 (Other DHS Direct)
Demographic and Health Survey 2006−2007 (DHS Indirect)
Demographic and Health Survey 2006−2007 (DHS Direct)
Malaria Indicator Survey 2008−2009 (Other DHS Indirect)
Population and Housing Census 2008 (Census Indirect)
Malaria Indicator Survey 2008−2009 (Other DHS Direct)
Malaria Indicator Survey 2011 (Other DHS Indirect)

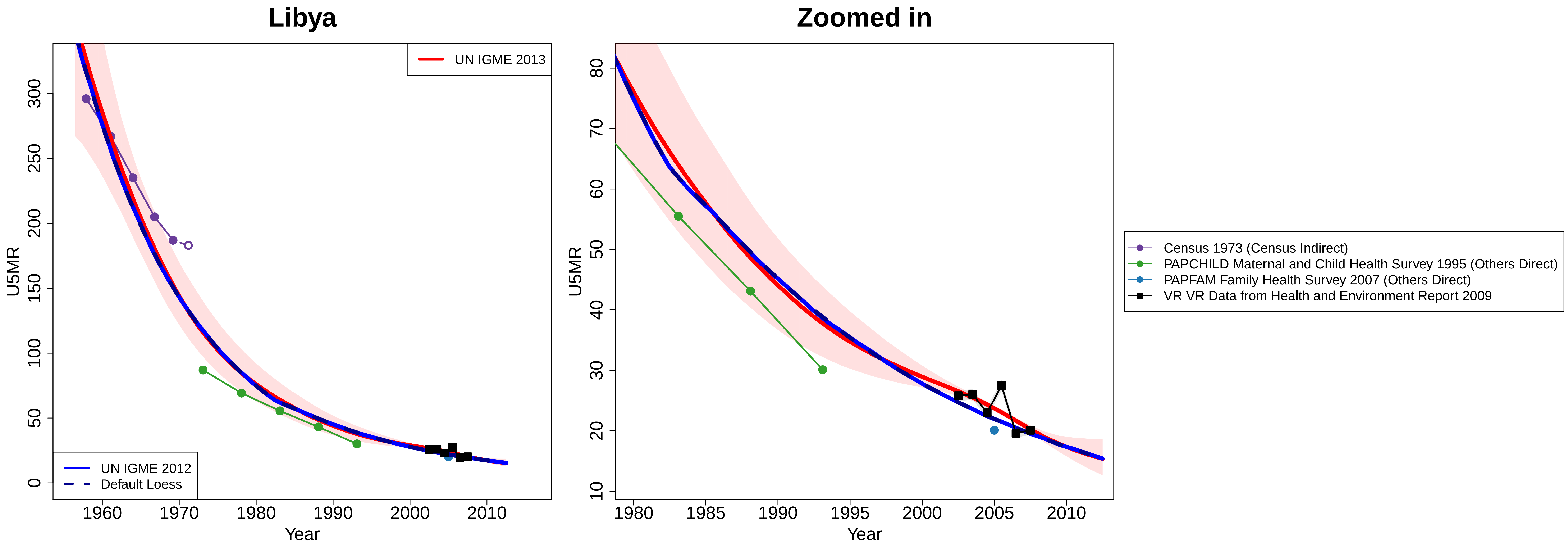

Libya
Zoomed in
UN IGME 2013
UN IGME 2012
Default Loess
U5MR
Year
Census 1973 (Census Indirect)
PAPCHILD Maternal and Child Health Survey 1995 (Others Direct)
PAPFAM Family Health Survey 2007 (Others Direct)
VR VR Data from Health and Environment Report 2009

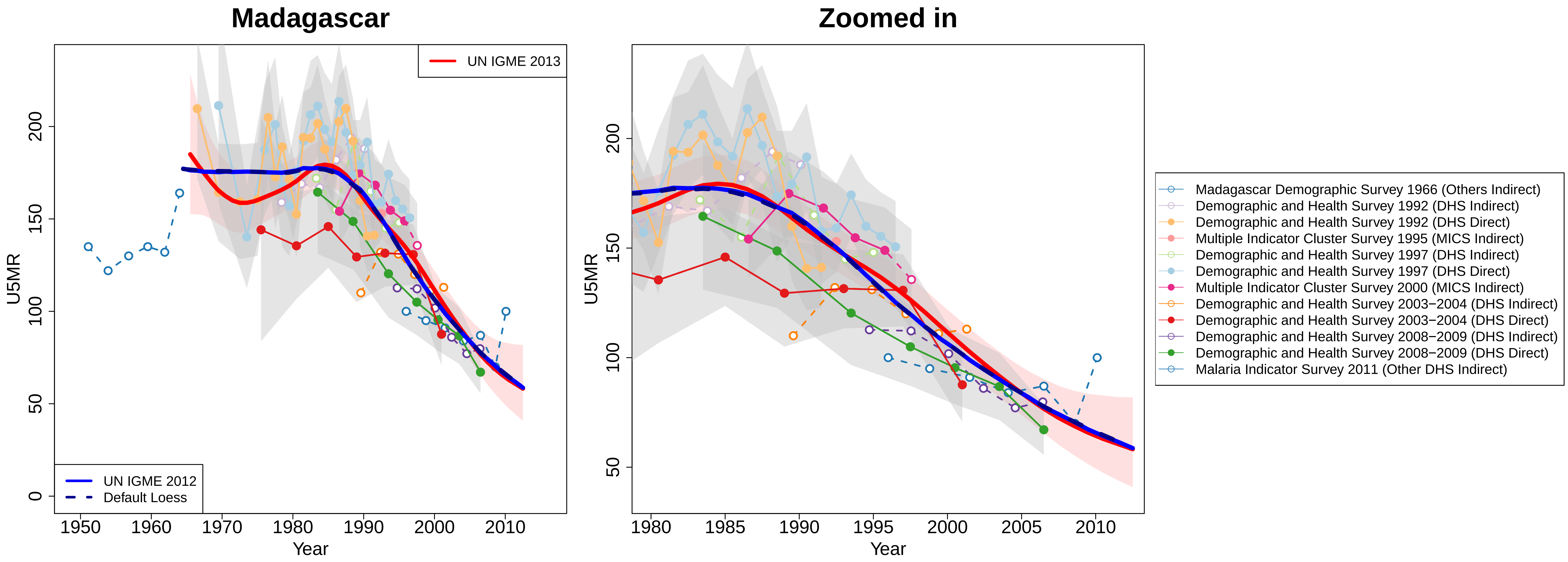

Madagascar
Zoomed in
UN IGME 2013
UN IGME 2012
Default Loess
U5MR
Year
0
50
100
150
200
1950
1960
1970
1980
1990
2000
2010
1985
1995
2005
Madagascar Demographic Survey 1966 (Others Indirect)
Demographic and Health Survey 1992 (DHS Indirect)
Demographic and Health Survey 1992 (DHS Direct)
Multiple Indicator Cluster Survey 1995 (MICS Indirect)
Demographic and Health Survey 1997 (DHS Indirect)
Demographic and Health Survey 1997 (DHS Direct)
Multiple Indicator Cluster Survey 2000 (MICS Indirect)
Demographic and Health Survey 2003−2004 (DHS Indirect)
Demographic and Health Survey 2003−2004 (DHS Direct)
Demographic and Health Survey 2008−2009 (DHS Indirect)
Demographic and Health Survey 2008−2009 (DHS Direct)
Malaria Indicator Survey 2011 (Other DHS Indirect)

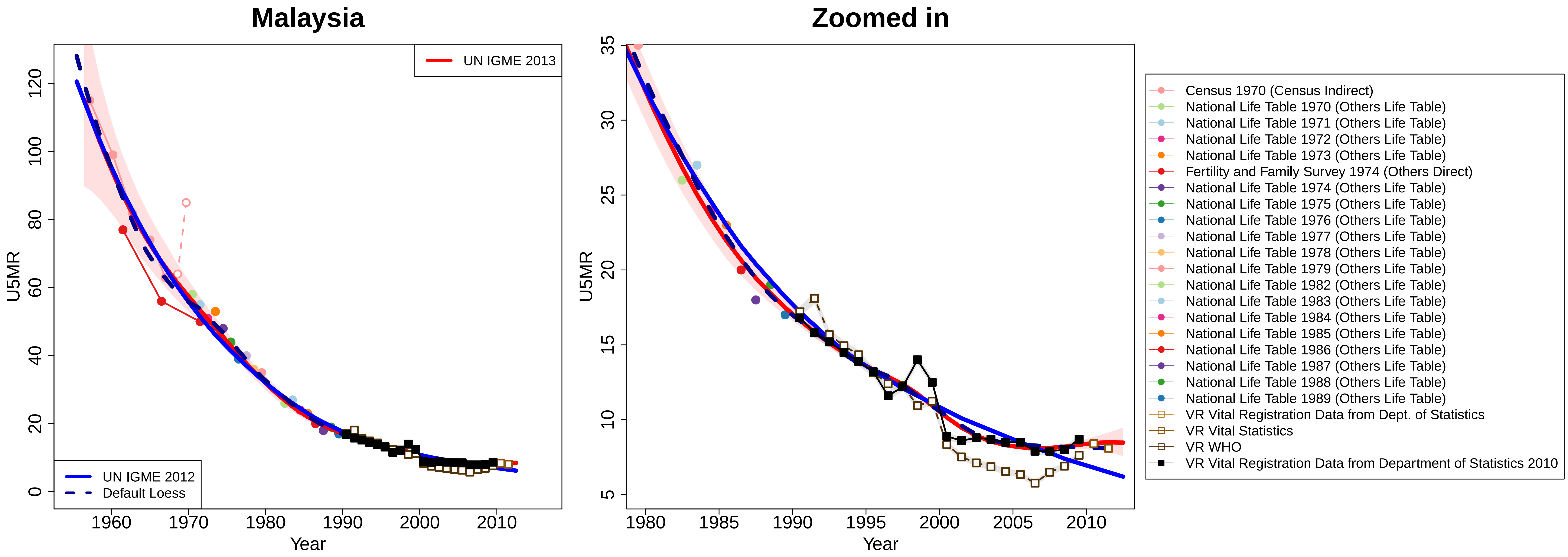
Malaysia
UN IGME 2013
UN IGME 2012
Default Loess
U5MR
Year
Zoomed in
Census 1970 (Census Indirect)
National Life Table 1970 (Others Life Table)
National Life Table 1971 (Others Life Table)
National Life Table 1972 (Others Life Table)
National Life Table 1973 (Others Life Table)
Fertility and Family Survey 1974 (Others Direct)
National Life Table 1974 (Others Life Table)
National Life Table 1975 (Others Life Table)
National Life Table 1976 (Others Life Table)
National Life Table 1977 (Others Life Table)
National Life Table 1978 (Others Life Table)
National Life Table 1979 (Others Life Table)
National Life Table 1982 (Others Life Table)
National Life Table 1983 (Others Life Table)
National Life Table 1984 (Others Life Table)
National Life Table 1985 (Others Life Table)
National Life Table 1986 (Others Life Table)
National Life Table 1987 (Others Life Table)
National Life Table 1988 (Others Life Table)
National Life Table 1989 (Others Life Table)
VR Vital Registration Data from Dept. of Statistics
VR Vital Statistics
VR WHO
VR Vital Registration Data from Department of Statistics 2010

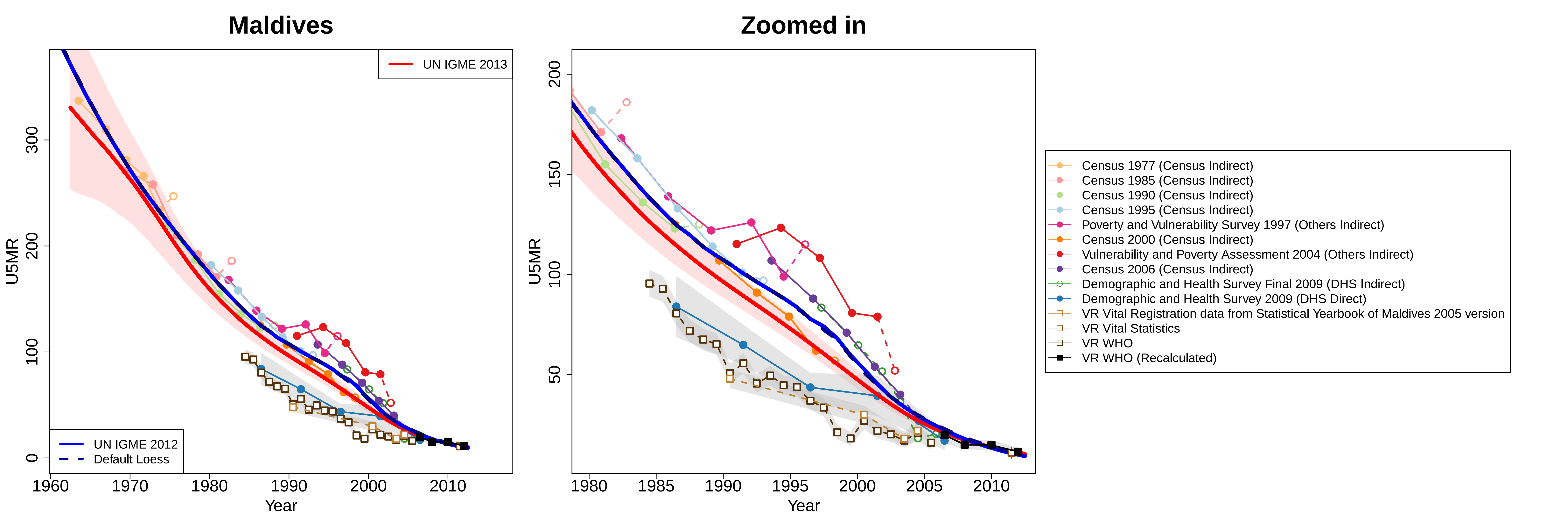

Maldives
Zoomed in
UN IGME 2013
UN IGME 2012
Default Loess
U5MR
Year
Census 1977 (Census Indirect)
Census 1985 (Census Indirect)
Census 1990 (Census Indirect)
Census 1995 (Census Indirect)
Poverty and Vulnerability Survey 1997 (Others Indirect)
Census 2000 (Census Indirect)
Vulnerability and Poverty Assessment 2004 (Others Indirect)
Census 2006 (Census Indirect)
Demographic and Health Survey Final 2009 (DHS Indirect)
Demographic and Health Survey 2009 (DHS Direct)
VR Vital Registration data from Statistical Yearbook of Maldives 2005 version
VR Vital Statistics
VR WHO
VR WHO (Recalculated)

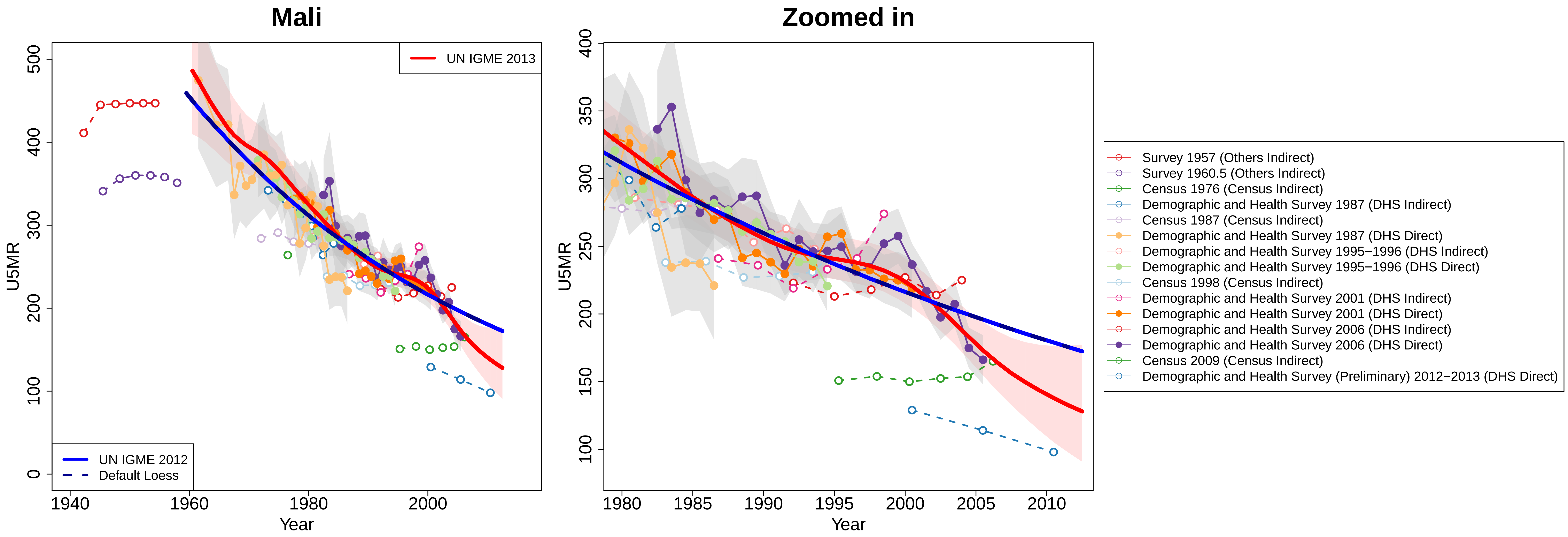
Mali
Zoomed in
UN IGME 2013
UN IGME 2012
Default Loess
U5MR
Year
Survey 1957 (Others Indirect)
Survey 1960.5 (Others Indirect)
Census 1976 (Census Indirect)
Demographic and Health Survey 1987 (DHS Indirect)
Census 1987 (Census Indirect)
Demographic and Health Survey 1987 (DHS Direct)
Demographic and Health Survey 1995−1996 (DHS Indirect)
Demographic and Health Survey 1995−1996 (DHS Direct)
Census 1998 (Census Indirect)
Demographic and Health Survey 2001 (DHS Indirect)
Demographic and Health Survey 2001 (DHS Direct)
Demographic and Health Survey 2006 (DHS Indirect)
Demographic and Health Survey 2006 (DHS Direct)
Census 2009 (Census Indirect)
Demographic and Health Survey (Preliminary) 2012−2013 (DHS Direct)

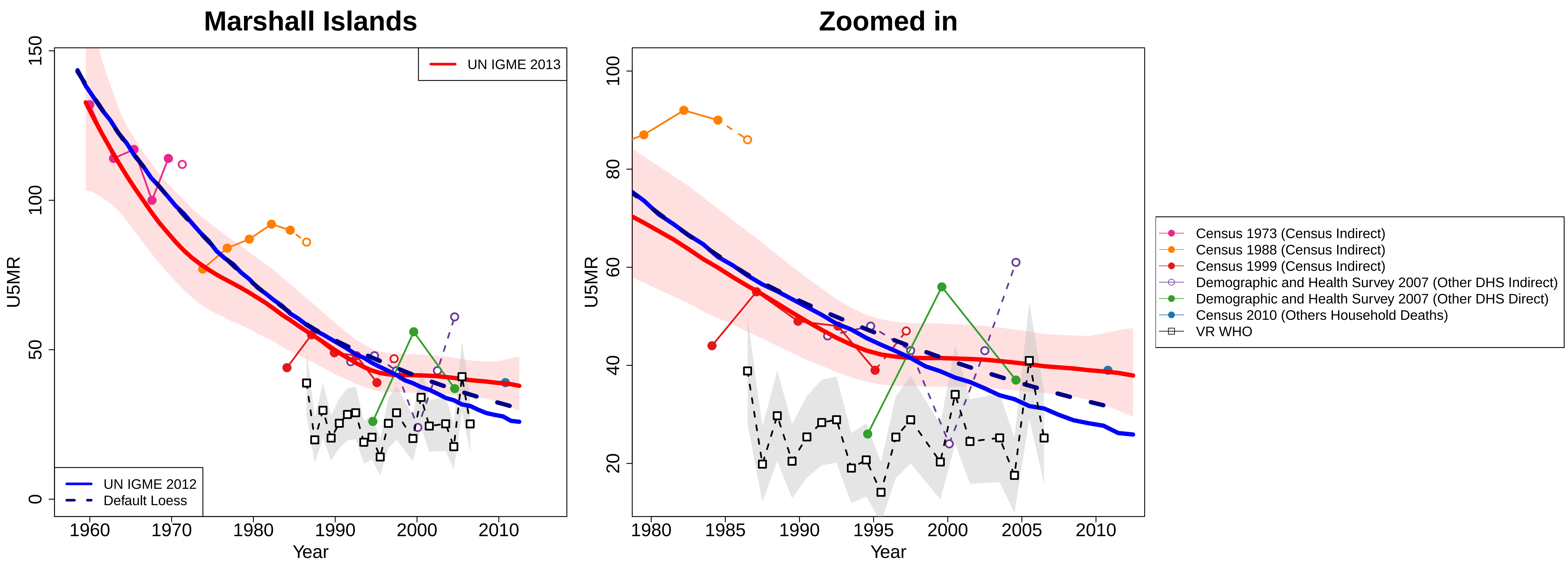

Marshall Islands
Zoomed in
U5MR
Year
UN IGME 2013
UN IGME 2012
Default Loess
Census 1973 (Census Indirect)
Census 1988 (Census Indirect)
Census 1999 (Census Indirect)
Demographic and Health Survey 2007 (Other DHS Indirect)
Demographic and Health Survey 2007 (Other DHS Direct)
Census 2010 (Others Household Deaths)
VR WHO

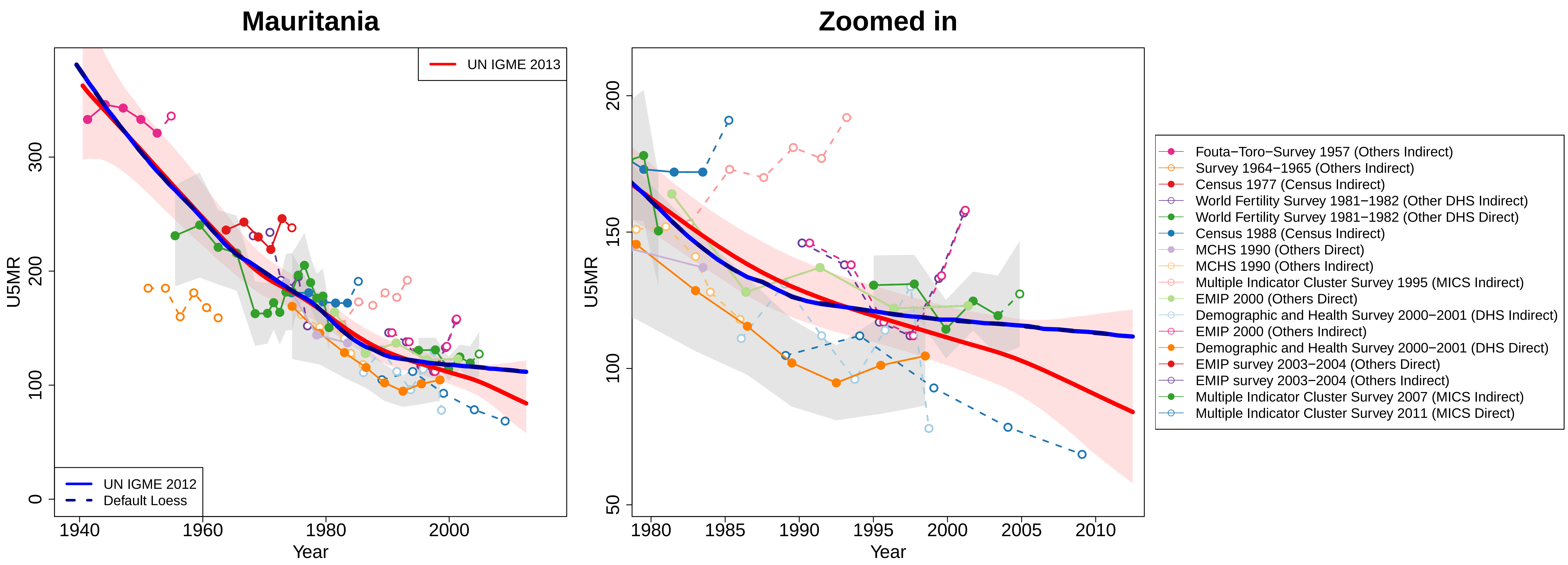
Mauritania
Zoomed in
UN IGME 2013
UN IGME 2012
Default Loess
U5MR
Year
0
100
200
300
1940
1960
1980
2000
50
150
1985
1990
1995
2005
2010
Fouta−Toro−Survey 1957 (Others Indirect)
Survey 1964−1965 (Others Indirect)
Census 1977 (Census Indirect)
World Fertility Survey 1981−1982 (Other DHS Indirect)
World Fertility Survey 1981−1982 (Other DHS Direct)
Census 1988 (Census Indirect)
MCHS 1990 (Others Direct)
MCHS 1990 (Others Indirect)
Multiple Indicator Cluster Survey 1995 (MICS Indirect)
EMIP 2000 (Others Direct)
Demographic and Health Survey 2000−2001 (DHS Indirect)
EMIP 2000 (Others Indirect)
Demographic and Health Survey 2000−2001 (DHS Direct)
EMIP survey 2003−2004 (Others Direct)
EMIP survey 2003−2004 (Others Indirect)
Multiple Indicator Cluster Survey 2007 (MICS Indirect)
Multiple Indicator Cluster Survey 2011 (MICS Direct)

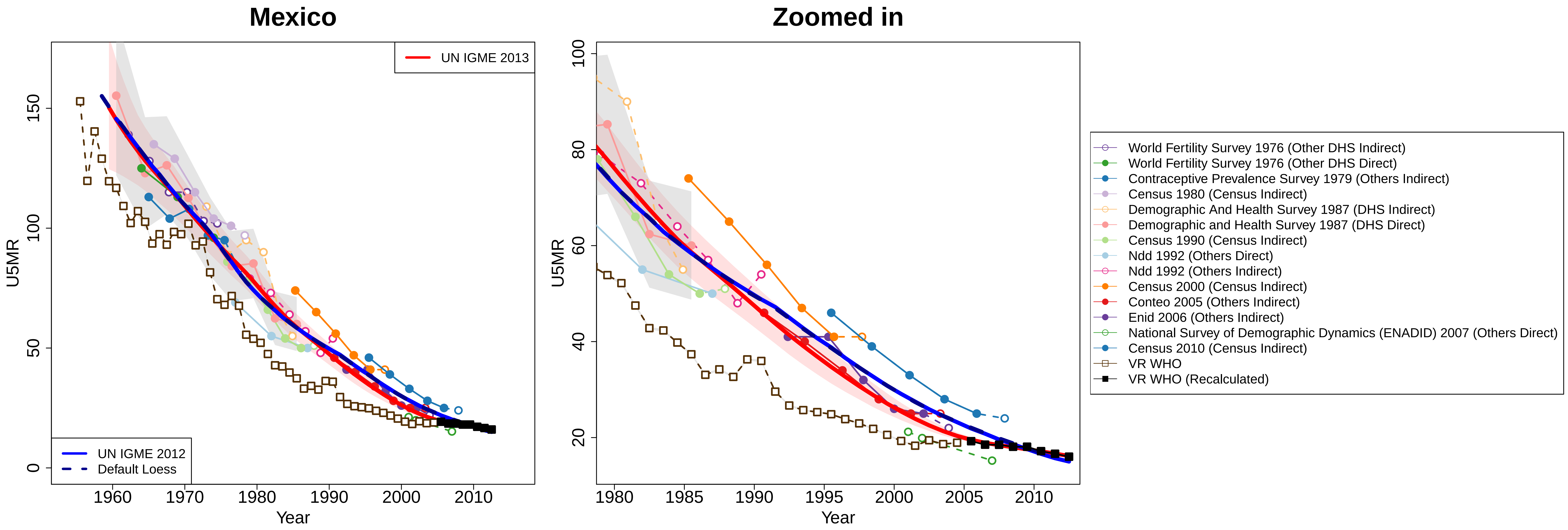

Mexico
Zoomed in
UN IGME 2013
UN IGME 2012
Default Loess
U5MR
Year
World Fertility Survey 1976 (Other DHS Indirect)
World Fertility Survey 1976 (Other DHS Direct)
Contraceptive Prevalence Survey 1979 (Others Indirect)
Census 1980 (Census Indirect)
Demographic And Health Survey 1987 (DHS Indirect)
Demographic and Health Survey 1987 (DHS Direct)
Census 1990 (Census Indirect)
Ndd 1992 (Others Direct)
Ndd 1992 (Others Indirect)
Census 2000 (Census Indirect)
Conteo 2005 (Others Indirect)
Enid 2006 (Others Indirect)
National Survey of Demographic Dynamics (ENADID) 2007 (Others Direct)
Census 2010 (Census Indirect)
VR WHO
VR WHO (Recalculated)

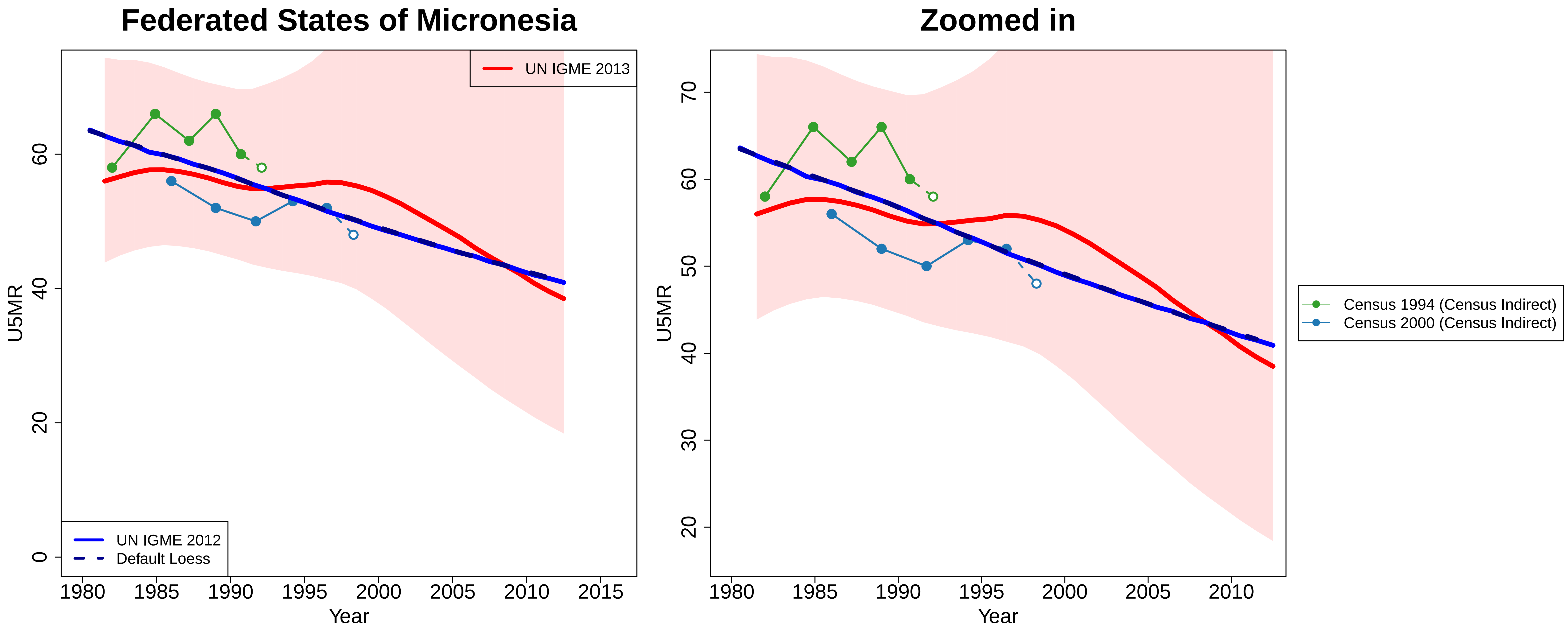
Federated States of Micronesia
Zoomed in
UN IGME 2013
UN IGME 2012
Default Loess
Census 1994 (Census Indirect)
Census 2000 (Census Indirect)
U5MR
Year
0
20
40
60
30
50
70
1980
1985
1990
1995
2000
2005
2010
2015

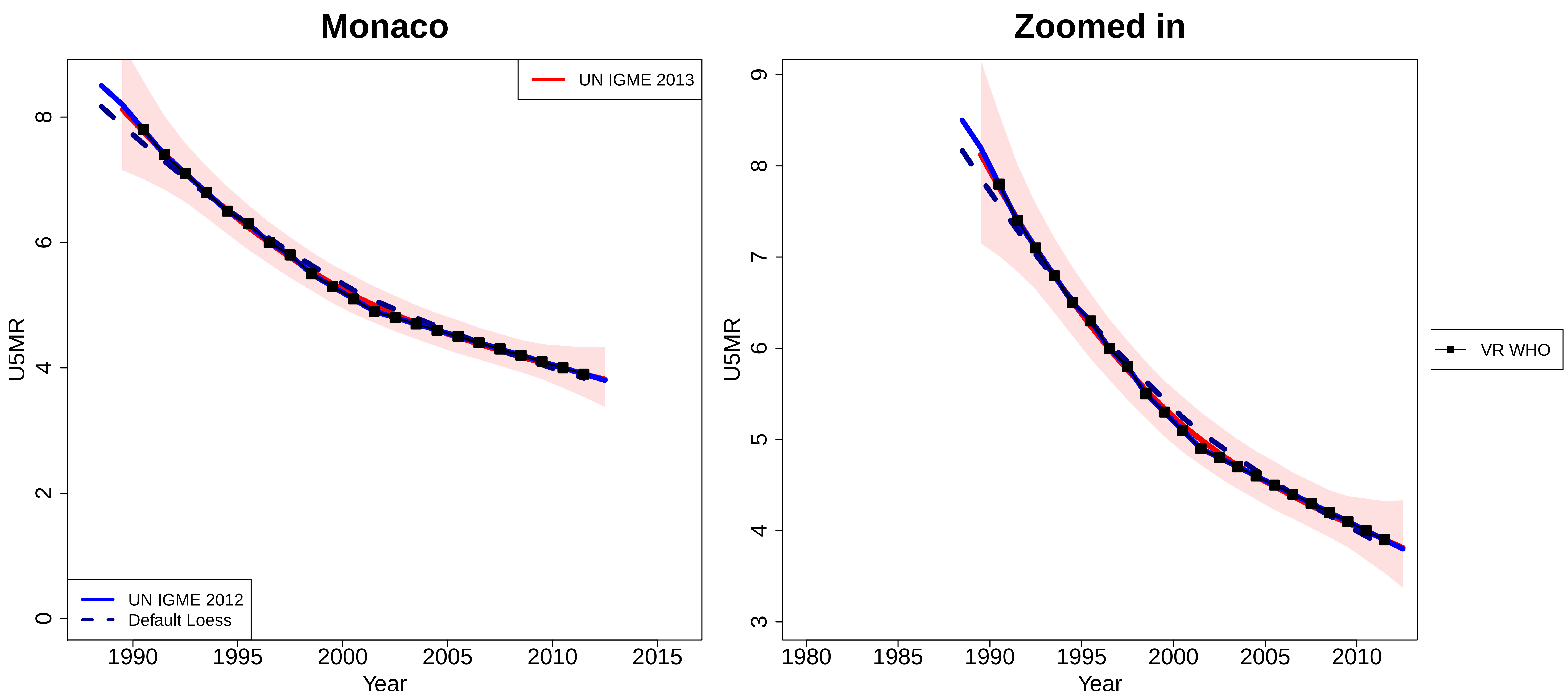

Monaco
Zoomed in
UN IGME 2013
UN IGME 2012
Default Loess
VR WHO
U5MR
Year

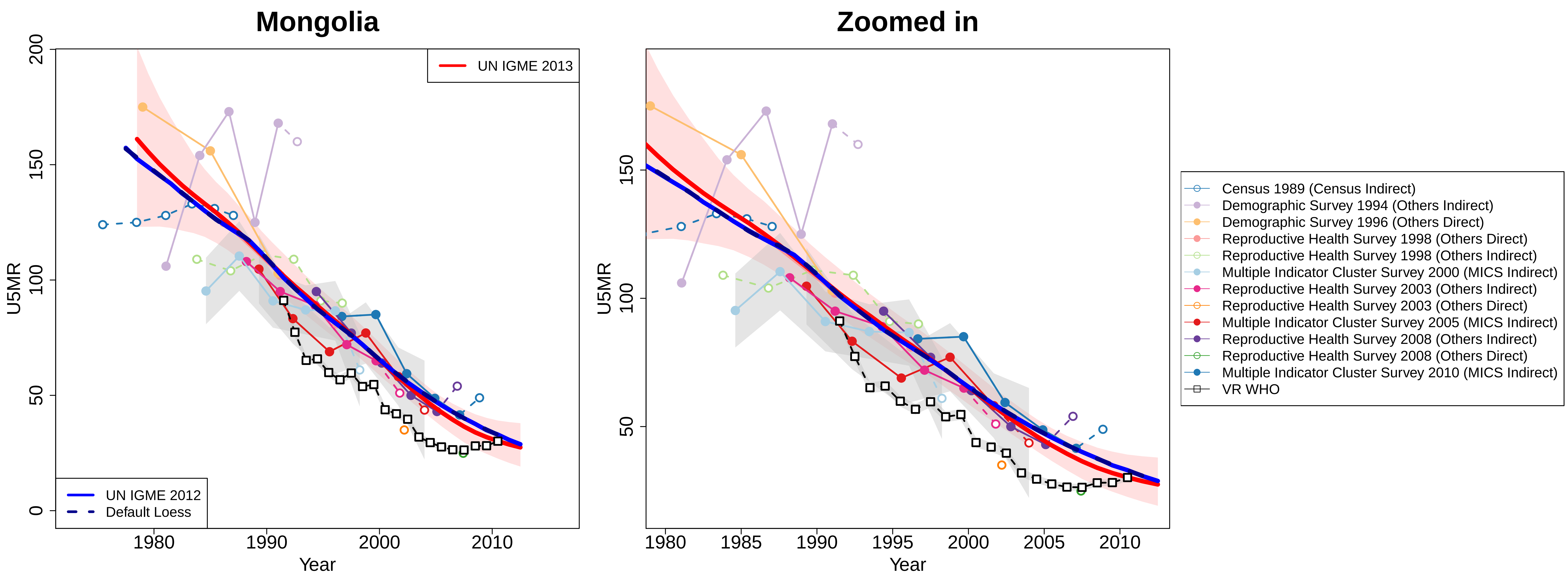

Mongolia
Zoomed in
UN IGME 2013
UN IGME 2012
Default Loess
U5MR
Year
0
50
100
150
200
1980
1985
1990
1995
2000
2005
2010
Census 1989 (Census Indirect)
Demographic Survey 1994 (Others Indirect)
Demographic Survey 1996 (Others Direct)
Reproductive Health Survey 1998 (Others Direct)
Reproductive Health Survey 1998 (Others Indirect)
Multiple Indicator Cluster Survey 2000 (MICS Indirect)
Reproductive Health Survey 2003 (Others Indirect)
Reproductive Health Survey 2003 (Others Direct)
Multiple Indicator Cluster Survey 2005 (MICS Indirect)
Reproductive Health Survey 2008 (Others Indirect)
Reproductive Health Survey 2008 (Others Direct)
Multiple Indicator Cluster Survey 2010 (MICS Indirect)
VR WHO

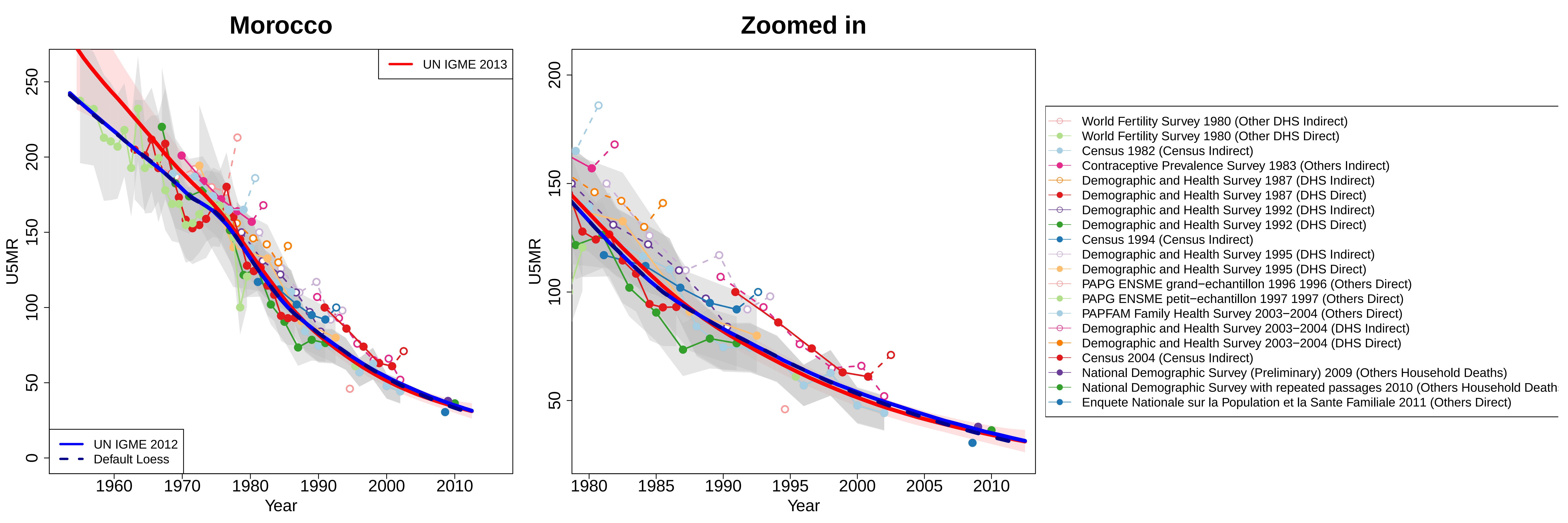

Morocco
Zoomed in
UN IGME 2013
UN IGME 2012
Default Loess
U5MR
Year
World Fertility Survey 1980 (Other DHS Indirect)
World Fertility Survey 1980 (Other DHS Direct)
Census 1982 (Census Indirect)
Contraceptive Prevalence Survey 1983 (Others Indirect)
Demographic and Health Survey 1987 (DHS Indirect)
Demographic and Health Survey 1987 (DHS Direct)
Demographic and Health Survey 1992 (DHS Indirect)
Demographic and Health Survey 1992 (DHS Direct)
Census 1994 (Census Indirect)
Demographic and Health Survey 1995 (DHS Indirect)
Demographic and Health Survey 1995 (DHS Direct)
PAPG ENSME grand−echantillon 1996 1996 (Others Direct)
PAPG ENSME petit−echantillon 1997 1997 (Others Direct)
PAPFAM Family Health Survey 2003−2004 (Others Direct)
Demographic and Health Survey 2003−2004 (DHS Indirect)
Demographic and Health Survey 2003−2004 (DHS Direct)
Census 2004 (Census Indirect)
National Demographic Survey (Preliminary) 2009 (Others Household Deaths)
National Demographic Survey with repeated passages 2010 (Others Household Death
Enquete Nationale sur la Population et la Sante Familiale 2011 (Others Direct)

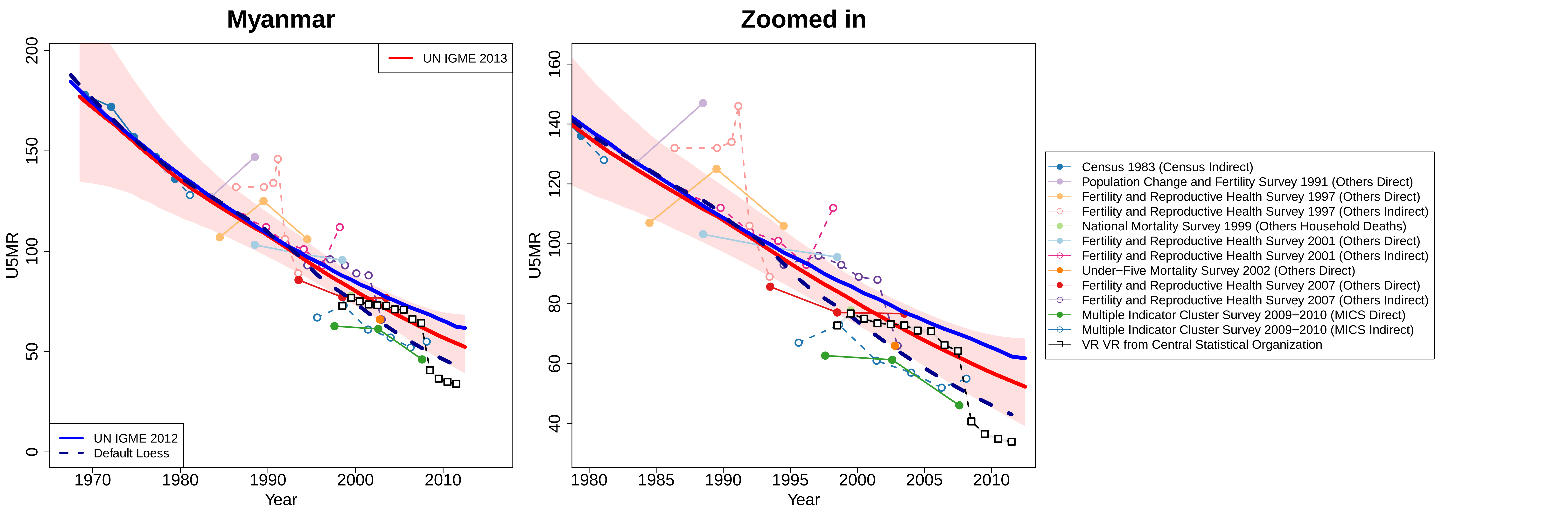

Myanmar
Zoomed in
UN IGME 2013
UN IGME 2012
Default Loess
U5MR
Year
Census 1983 (Census Indirect)
Population Change and Fertility Survey 1991 (Others Direct)
Fertility and Reproductive Health Survey 1997 (Others Direct)
Fertility and Reproductive Health Survey 1997 (Others Indirect)
National Mortality Survey 1999 (Others Household Deaths)
Fertility and Reproductive Health Survey 2001 (Others Direct)
Fertility and Reproductive Health Survey 2001 (Others Indirect)
Under−Five Mortality Survey 2002 (Others Direct)
Fertility and Reproductive Health Survey 2007 (Others Direct)
Fertility and Reproductive Health Survey 2007 (Others Indirect)
Multiple Indicator Cluster Survey 2009−2010 (MICS Direct)
Multiple Indicator Cluster Survey 2009−2010 (MICS Indirect)
VR VR from Central Statistical Organization

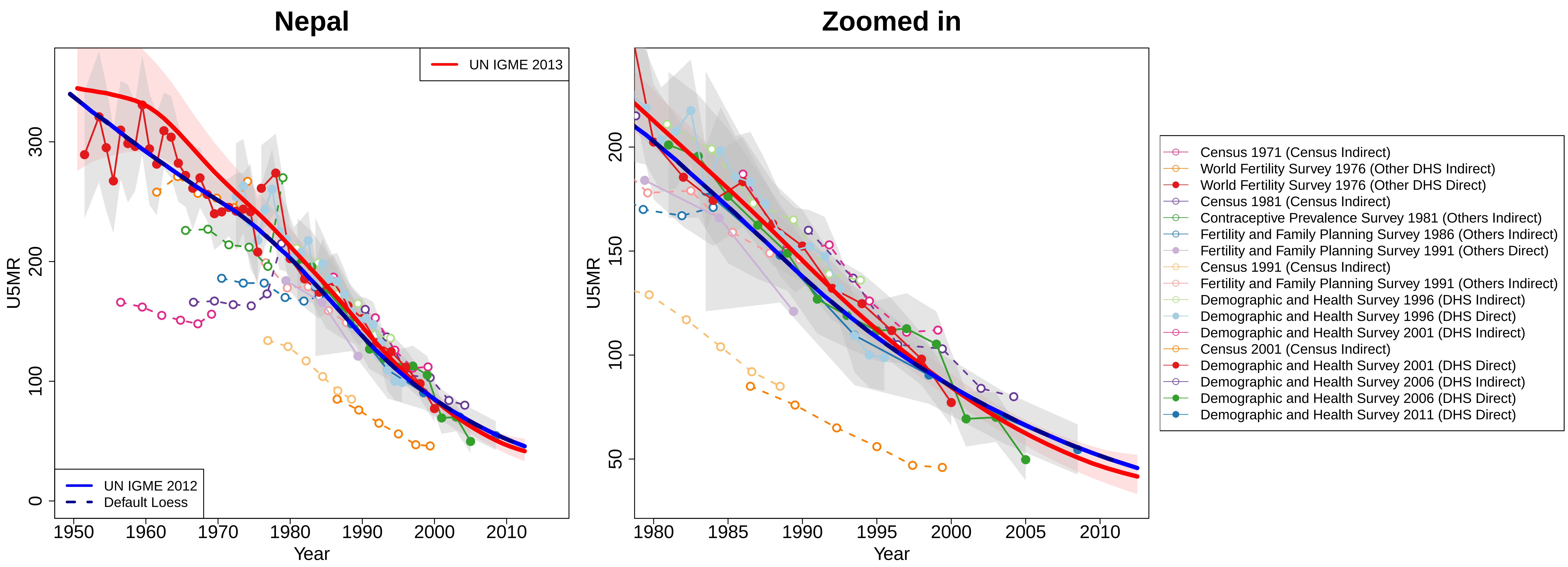
Nepal
Zoomed in
U5MR
Year
UN IGME 2013
UN IGME 2012
Default Loess
Census 1971 (Census Indirect)
World Fertility Survey 1976 (Other DHS Indirect)
World Fertility Survey 1976 (Other DHS Direct)
Census 1981 (Census Indirect)
Contraceptive Prevalence Survey 1981 (Others Indirect)
Fertility and Family Planning Survey 1986 (Others Indirect)
Fertility and Family Planning Survey 1991 (Others Direct)
Census 1991 (Census Indirect)
Fertility and Family Planning Survey 1991 (Others Indirect)
Demographic and Health Survey 1996 (DHS Indirect)
Demographic and Health Survey 1996 (DHS Direct)
Demographic and Health Survey 2001 (DHS Indirect)
Census 2001 (Census Indirect)
Demographic and Health Survey 2001 (DHS Direct)
Demographic and Health Survey 2006 (DHS Indirect)
Demographic and Health Survey 2006 (DHS Direct)
Demographic and Health Survey 2011 (DHS Direct)

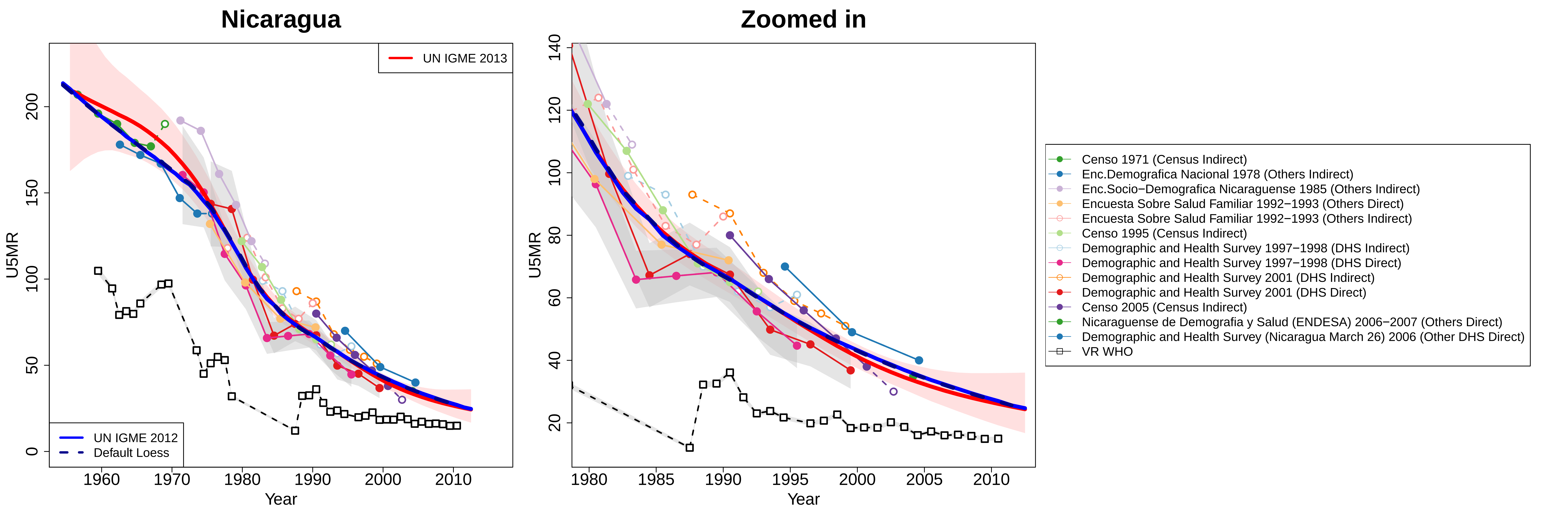
Nicaragua
Zoomed in
UN IGME 2013
UN IGME 2012
Default Loess
U5MR
Year
Censo 1971 (Census Indirect)
Enc.Demografica Nacional 1978 (Others Indirect)
Enc.Socio−Demografica Nicaraguense 1985 (Others Indirect)
Encuesta Sobre Salud Familiar 1992−1993 (Others Direct)
Encuesta Sobre Salud Familiar 1992−1993 (Others Indirect)
Censo 1995 (Census Indirect)
Demographic and Health Survey 1997−1998 (DHS Indirect)
Demographic and Health Survey 1997−1998 (DHS Direct)
Demographic and Health Survey 2001 (DHS Indirect)
Demographic and Health Survey 2001 (DHS Direct)
Censo 2005 (Census Indirect)
Nicaraguense de Demografia y Salud (ENDESA) 2006−2007 (Others Direct)
Demographic and Health Survey (Nicaragua March 26) 2006 (Other DHS Direct)
VR WHO

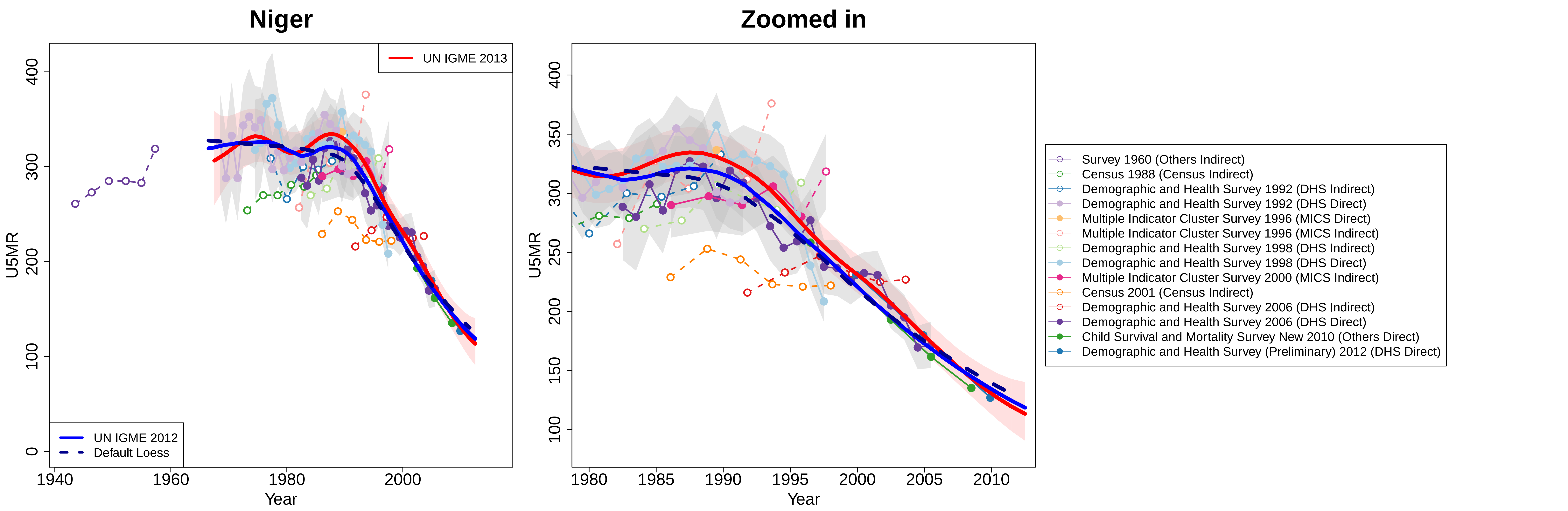
Niger
Zoomed in
U5MR
Year
UN IGME 2013
UN IGME 2012
Default Loess
Survey 1960 (Others Indirect)
Census 1988 (Census Indirect)
Demographic and Health Survey 1992 (DHS Indirect)
Demographic and Health Survey 1992 (DHS Direct)
Multiple Indicator Cluster Survey 1996 (MICS Direct)
Multiple Indicator Cluster Survey 1996 (MICS Indirect)
Demographic and Health Survey 1998 (DHS Indirect)
Demographic and Health Survey 1998 (DHS Direct)
Multiple Indicator Cluster Survey 2000 (MICS Indirect)
Census 2001 (Census Indirect)
Demographic and Health Survey 2006 (DHS Indirect)
Demographic and Health Survey 2006 (DHS Direct)
Child Survival and Mortality Survey New 2010 (Others Direct)
Demographic and Health Survey (Preliminary) 2012 (DHS Direct)

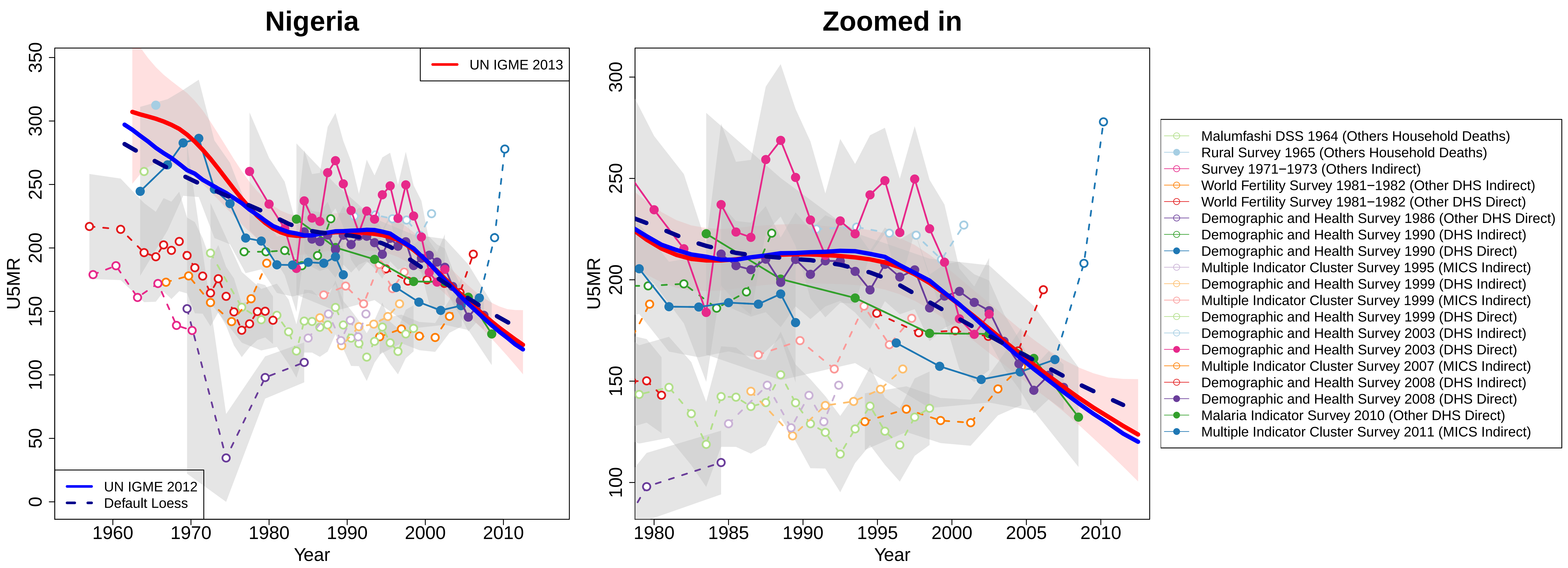

Nigeria
Zoomed in
U5MR
Year
UN IGME 2013
UN IGME 2012
Default Loess
Malumfashi DSS 1964 (Others Household Deaths)
Rural Survey 1965 (Others Household Deaths)
Survey 1971−1973 (Others Indirect)
World Fertility Survey 1981−1982 (Other DHS Indirect)
World Fertility Survey 1981−1982 (Other DHS Direct)
Demographic and Health Survey 1986 (Other DHS Direct)
Demographic and Health Survey 1990 (DHS Indirect)
Demographic and Health Survey 1990 (DHS Direct)
Multiple Indicator Cluster Survey 1995 (MICS Indirect)
Demographic and Health Survey 1999 (DHS Indirect)
Multiple Indicator Cluster Survey 1999 (MICS Indirect)
Demographic and Health Survey 1999 (DHS Direct)
Demographic and Health Survey 2003 (DHS Indirect)
Demographic and Health Survey 2003 (DHS Direct)
Multiple Indicator Cluster Survey 2007 (MICS Indirect)
Demographic and Health Survey 2008 (DHS Indirect)
Demographic and Health Survey 2008 (DHS Direct)
Malaria Indicator Survey 2010 (Other DHS Direct)
Multiple Indicator Cluster Survey 2011 (MICS Indirect)

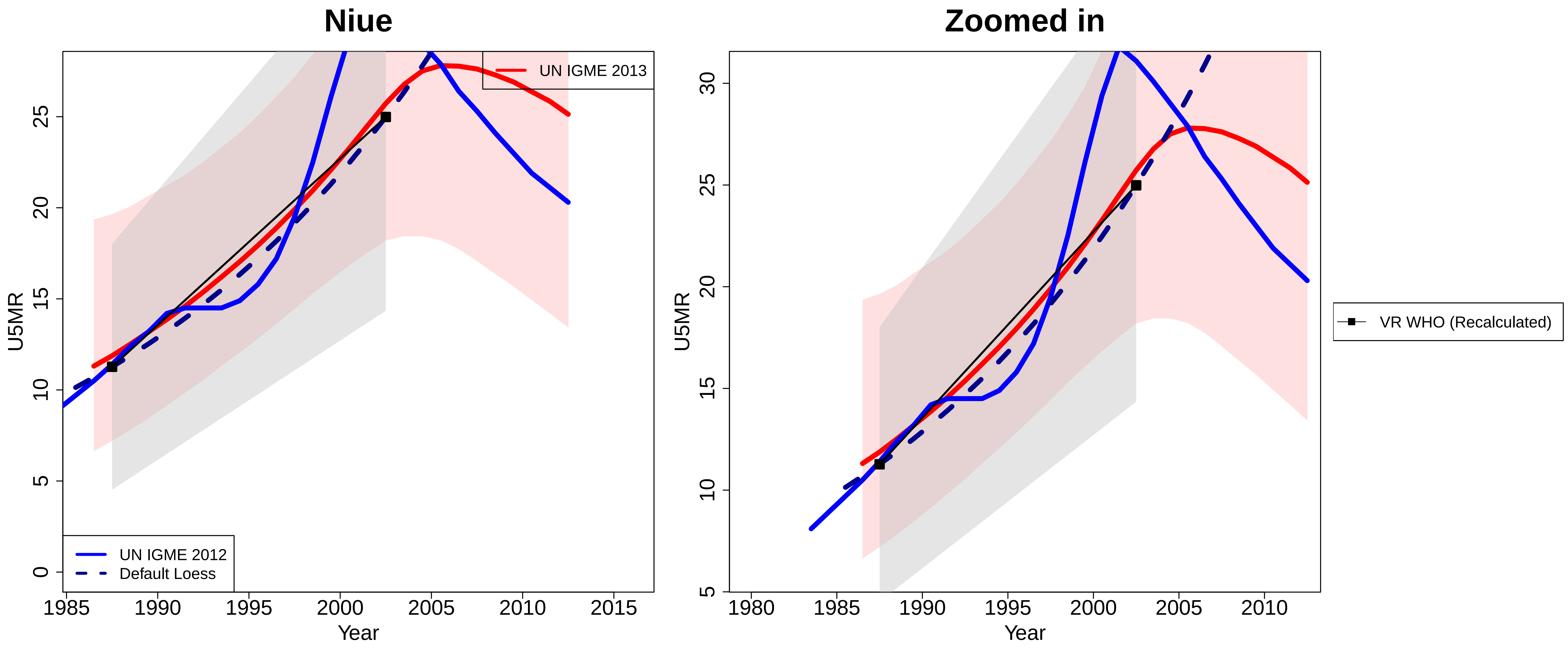

Niue
Zoomed in
UN IGME 2013
UN IGME 2012
Default Loess
VR WHO (Recalculated)
U5MR
Year
0
5
10
15
20
25
30
1980
1985
1990
1995
2000
2005
2010
2015

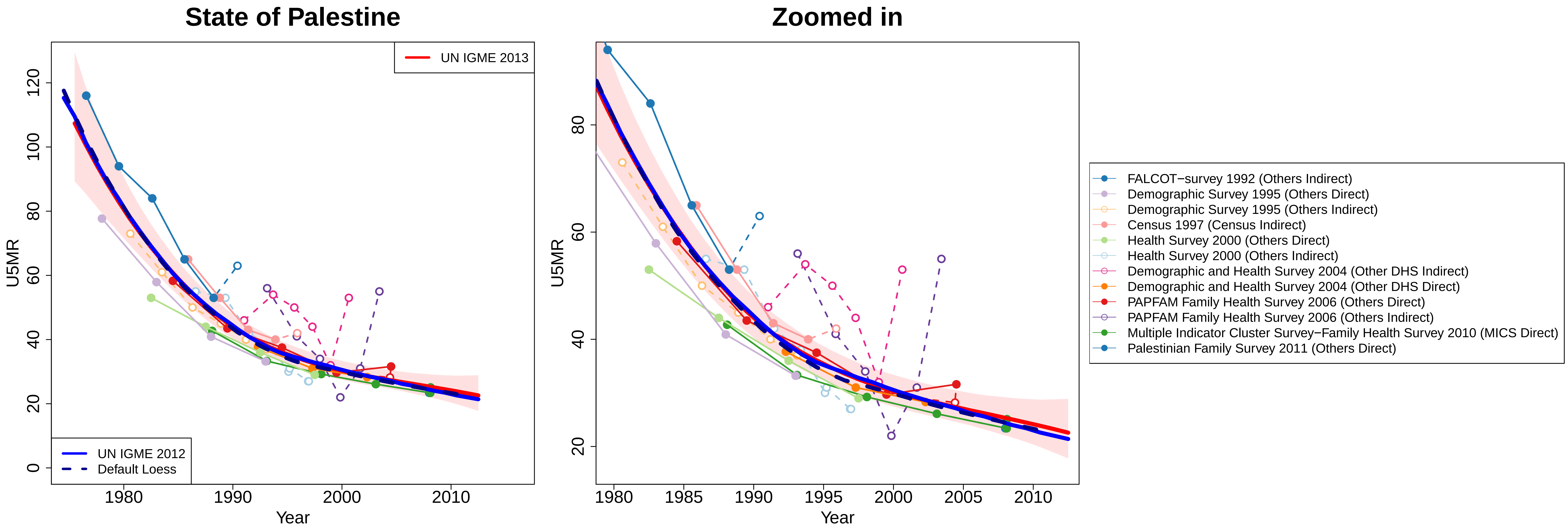

State of Palestine
Zoomed in
UN IGME 2013
UN IGME 2012
Default Loess
U5MR
Year
0
20
40
60
80
100
120
1980
1985
1990
1995
2000
2005
2010
FALCOT−survey 1992 (Others Indirect)
Demographic Survey 1995 (Others Direct)
Demographic Survey 1995 (Others Indirect)
Census 1997 (Census Indirect)
Health Survey 2000 (Others Direct)
Health Survey 2000 (Others Indirect)
Demographic and Health Survey 2004 (Other DHS Indirect)
Demographic and Health Survey 2004 (Other DHS Direct)
PAPFAM Family Health Survey 2006 (Others Direct)
PAPFAM Family Health Survey 2006 (Others Indirect)
Multiple Indicator Cluster Survey−Family Health Survey 2010 (MICS Direct)
Palestinian Family Survey 2011 (Others Direct)

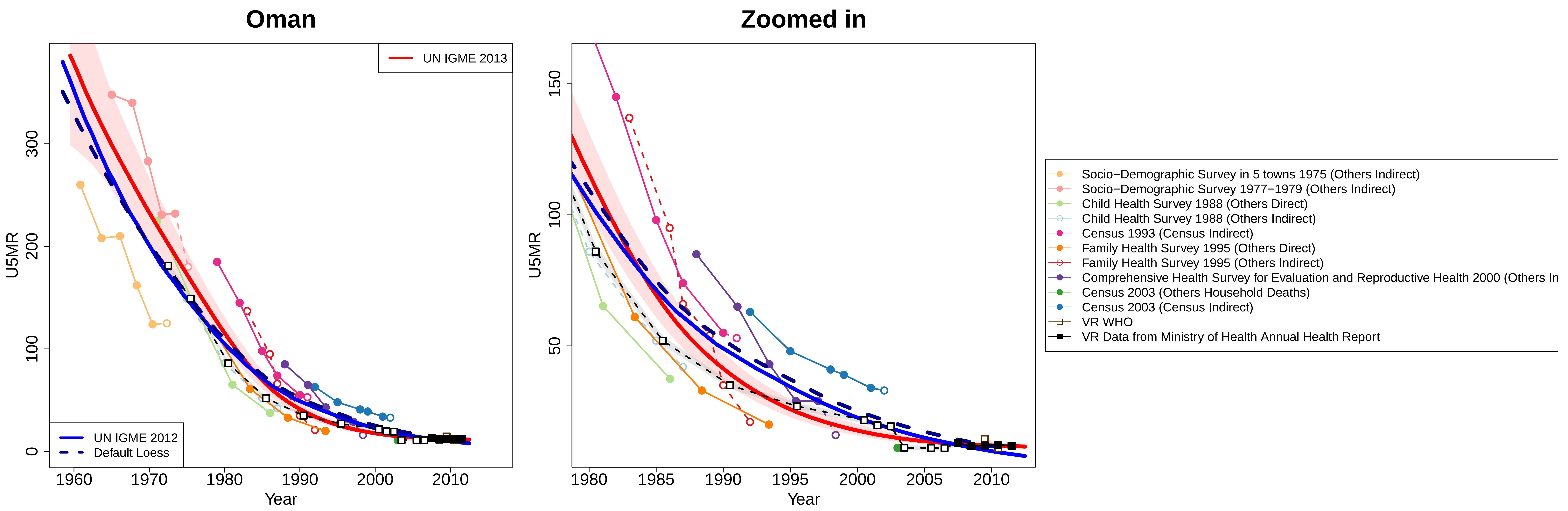

Oman
UN IGME 2013
UN IGME 2012
Default Loess
U5MR
Year
0
100
200
300
1960
1970
1980
1990
2000
2010
Zoomed in
U5MR
Year
50
100
150
1980
1985
1990
1995
2000
2005
2010
Socio−Demographic Survey in 5 towns 1975 (Others Indirect)
Socio−Demographic Survey 1977−1979 (Others Indirect)
Child Health Survey 1988 (Others Direct)
Child Health Survey 1988 (Others Indirect)
Census 1993 (Census Indirect)
Family Health Survey 1995 (Others Direct)
Family Health Survey 1995 (Others Indirect)
Comprehensive Health Survey for Evaluation and Reproductive Health 2000 (Others In
Census 2003 (Others Household Deaths)
Census 2003 (Census Indirect)
VR WHO
VR Data from Ministry of Health Annual Health Report

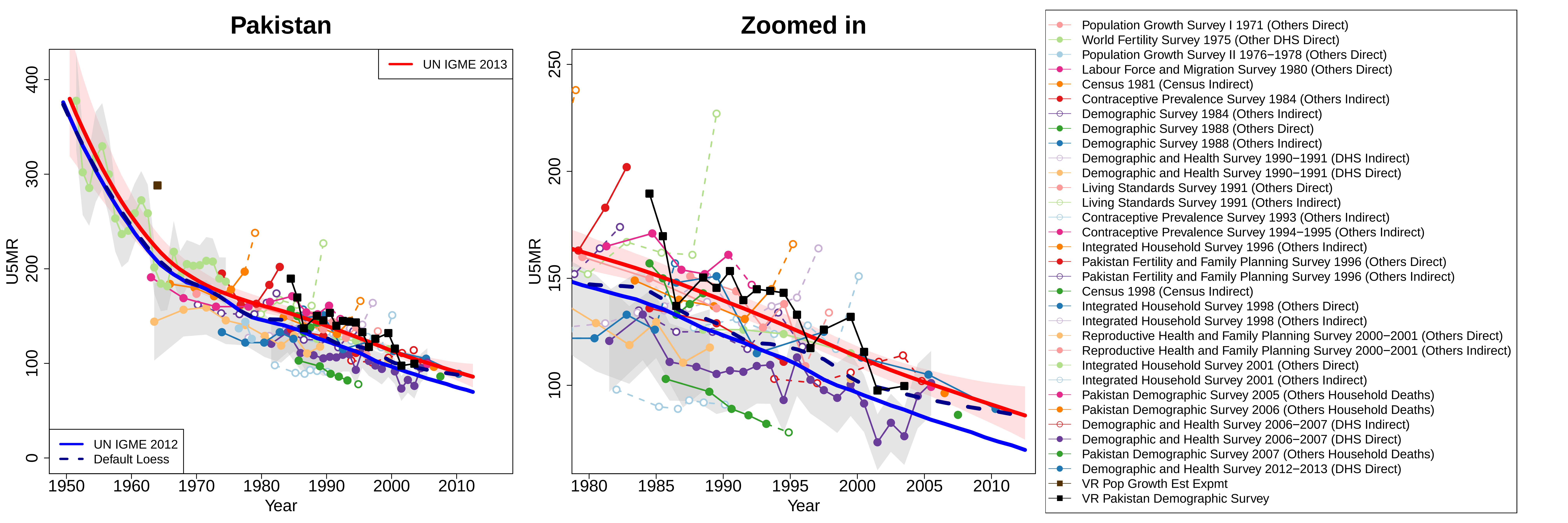
Pakistan
Zoomed in
UN IGME 2013
UN IGME 2012
Default Loess
U5MR
Year
Population Growth Survey I 1971 (Others Direct)
World Fertility Survey 1975 (Other DHS Direct)
Population Growth Survey II 1976−1978 (Others Direct)
Labour Force and Migration Survey 1980 (Others Direct)
Census 1981 (Census Indirect)
Contraceptive Prevalence Survey 1984 (Others Indirect)
Demographic Survey 1984 (Others Indirect)
Demographic Survey 1988 (Others Direct)
Demographic Survey 1988 (Others Indirect)
Demographic and Health Survey 1990−1991 (DHS Indirect)
Demographic and Health Survey 1990−1991 (DHS Direct)
Living Standards Survey 1991 (Others Direct)
Living Standards Survey 1991 (Others Indirect)
Contraceptive Prevalence Survey 1993 (Others Indirect)
Contraceptive Prevalence Survey 1994−1995 (Others Indirect)
Integrated Household Survey 1996 (Others Indirect)
Pakistan Fertility and Family Planning Survey 1996 (Others Direct)
Pakistan Fertility and Family Planning Survey 1996 (Others Indirect)
Census 1998 (Census Indirect)
Integrated Household Survey 1998 (Others Direct)
Integrated Household Survey 1998 (Others Indirect)
Reproductive Health and Family Planning Survey 2000−2001 (Others Direct)
Reproductive Health and Family Planning Survey 2000−2001 (Others Indirect)
Integrated Household Survey 2001 (Others Direct)
Integrated Household Survey 2001 (Others Indirect)
Pakistan Demographic Survey 2005 (Others Household Deaths)
Pakistan Demographic Survey 2006 (Others Household Deaths)
Demographic and Health Survey 2006−2007 (DHS Indirect)
Demographic and Health Survey 2006−2007 (DHS Direct)
Pakistan Demographic Survey 2007 (Others Household Deaths)
Demographic and Health Survey 2012−2013 (DHS Direct)
VR Pop Growth Est Expmt
VR Pakistan Demographic Survey

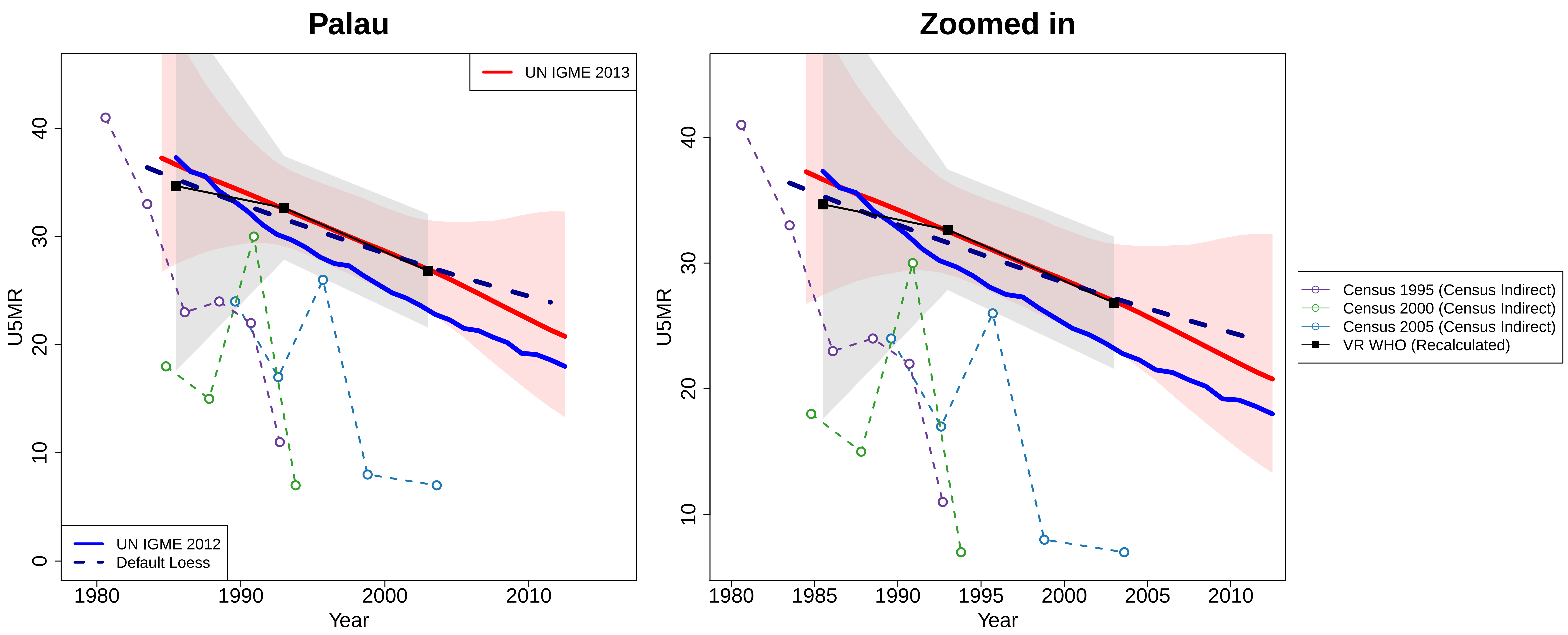

Palau
Zoomed in
UN IGME 2013
UN IGME 2012
Default Loess
Census 1995 (Census Indirect)
Census 2000 (Census Indirect)
Census 2005 (Census Indirect)
VR WHO (Recalculated)
U5MR
Year
0
10
20
30
40
1980
1985
1990
1995
2000
2005
2010

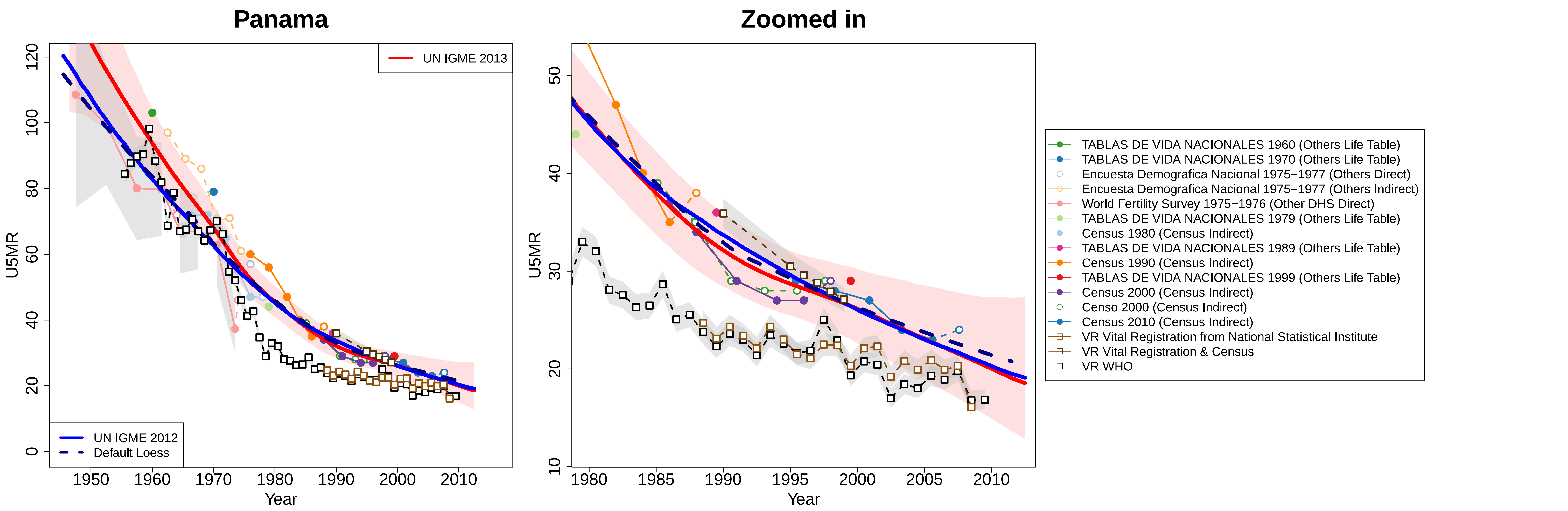

Panama
UN IGME 2013
UN IGME 2012
Default Loess
U5MR
Year
0
20
40
60
80
100
120
1950
1960
1970
1980
1990
2000
2010
Zoomed in
10
20
30
40
50
1980
1985
1990
1995
2000
2005
2010
TABLAS DE VIDA NACIONALES 1960 (Others Life Table)
TABLAS DE VIDA NACIONALES 1970 (Others Life Table)
Encuesta Demografica Nacional 1975−1977 (Others Direct)
Encuesta Demografica Nacional 1975−1977 (Others Indirect)
World Fertility Survey 1975−1976 (Other DHS Direct)
TABLAS DE VIDA NACIONALES 1979 (Others Life Table)
Census 1980 (Census Indirect)
TABLAS DE VIDA NACIONALES 1989 (Others Life Table)
Census 1990 (Census Indirect)
TABLAS DE VIDA NACIONALES 1999 (Others Life Table)
Census 2000 (Census Indirect)
Censo 2000 (Census Indirect)
Census 2010 (Census Indirect)
VR Vital Registration from National Statistical Institute
VR Vital Registration & Census
VR WHO

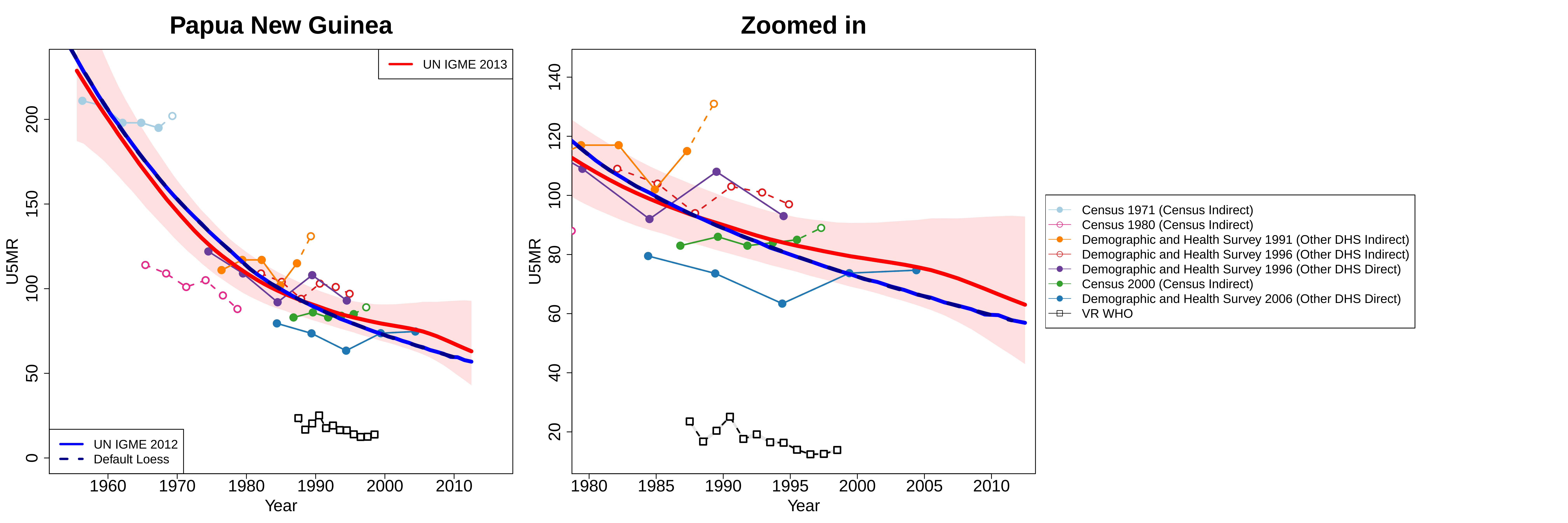

Papua New Guinea
UN IGME 2013
UN IGME 2012
Default Loess
U5MR
Year
Zoomed in
Census 1971 (Census Indirect)
Census 1980 (Census Indirect)
Demographic and Health Survey 1991 (Other DHS Indirect)
Demographic and Health Survey 1996 (Other DHS Indirect)
Demographic and Health Survey 1996 (Other DHS Direct)
Census 2000 (Census Indirect)
Demographic and Health Survey 2006 (Other DHS Direct)
VR WHO

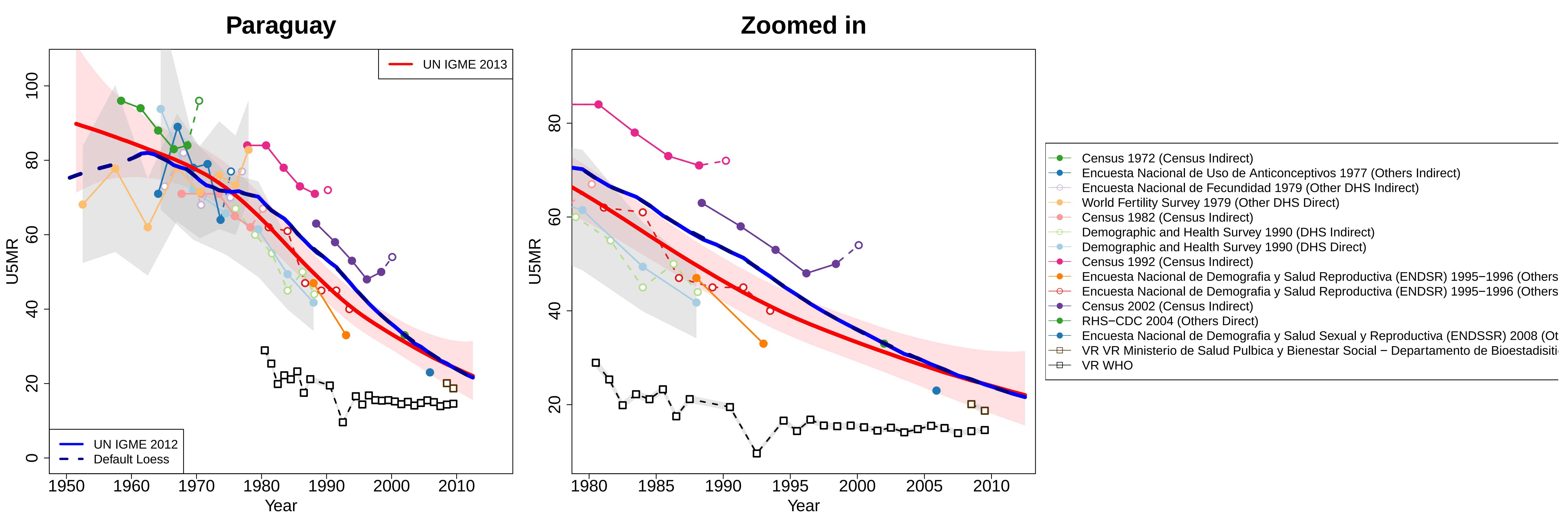

Paraguay
Zoomed in
UN IGME 2013
UN IGME 2012
Default Loess
U5MR
Year
Census 1972 (Census Indirect)
Encuesta Nacional de Uso de Anticonceptivos 1977 (Others Indirect)
Encuesta Nacional de Fecundidad 1979 (Other DHS Indirect)
World Fertility Survey 1979 (Other DHS Direct)
Census 1982 (Census Indirect)
Demographic and Health Survey 1990 (DHS Indirect)
Demographic and Health Survey 1990 (DHS Direct)
Census 1992 (Census Indirect)
Encuesta Nacional de Demografia y Salud Reproductiva (ENDSR) 1995−1996 (Others
Encuesta Nacional de Demografia y Salud Reproductiva (ENDSR) 1995−1996 (Others
Census 2002 (Census Indirect)
RHS−CDC 2004 (Others Direct)
Encuesta Nacional de Demografia y Salud Sexual y Reproductiva (ENDSSR) 2008 (Ot
VR VR Ministerio de Salud Pulbica y Bienestar Social − Departamento de Bioestadisiti
VR WHO

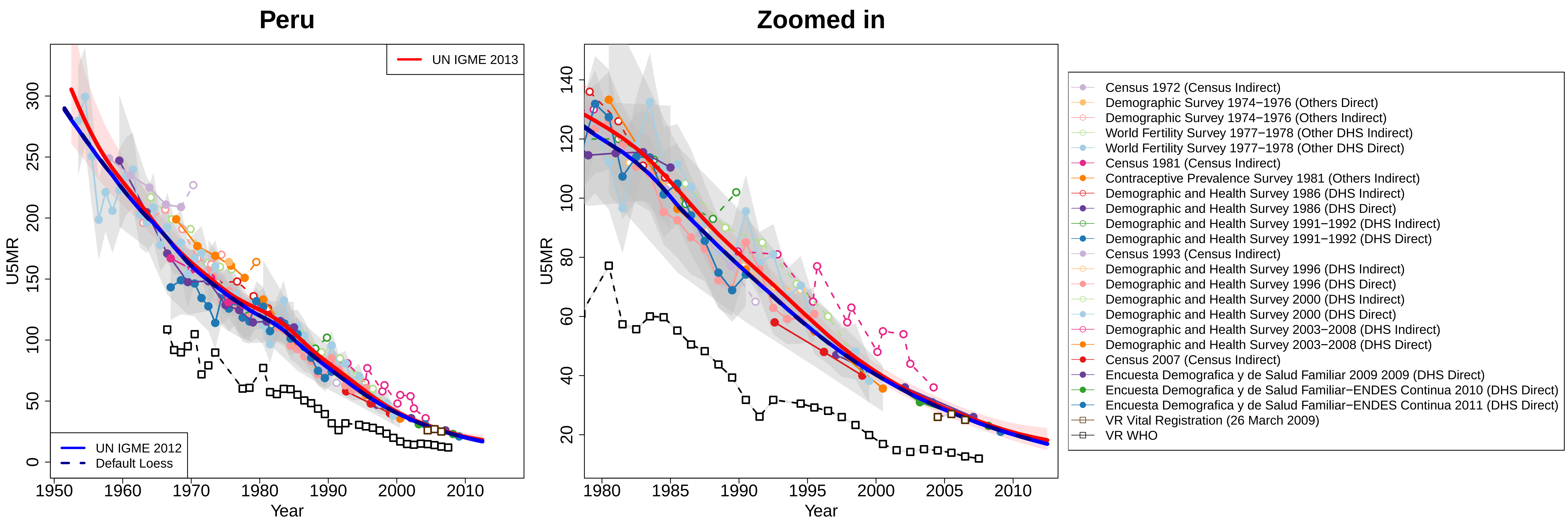

Peru
Zoomed in
UN IGME 2013
UN IGME 2012
Default Loess
U5MR
Year
Census 1972 (Census Indirect)
Demographic Survey 1974−1976 (Others Direct)
Demographic Survey 1974−1976 (Others Indirect)
World Fertility Survey 1977−1978 (Other DHS Indirect)
World Fertility Survey 1977−1978 (Other DHS Direct)
Census 1981 (Census Indirect)
Contraceptive Prevalence Survey 1981 (Others Indirect)
Demographic and Health Survey 1986 (DHS Indirect)
Demographic and Health Survey 1986 (DHS Direct)
Demographic and Health Survey 1991−1992 (DHS Indirect)
Demographic and Health Survey 1991−1992 (DHS Direct)
Census 1993 (Census Indirect)
Demographic and Health Survey 1996 (DHS Indirect)
Demographic and Health Survey 1996 (DHS Direct)
Demographic and Health Survey 2000 (DHS Indirect)
Demographic and Health Survey 2000 (DHS Direct)
Demographic and Health Survey 2003−2008 (DHS Indirect)
Demographic and Health Survey 2003−2008 (DHS Direct)
Census 2007 (Census Indirect)
Encuesta Demografica y de Salud Familiar 2009 2009 (DHS Direct)
Encuesta Demografica y de Salud Familiar−ENDES Continua 2010 (DHS Direct)
Encuesta Demografica y de Salud Familiar−ENDES Continua 2011 (DHS Direct)
VR Vital Registration (26 March 2009)
VR WHO

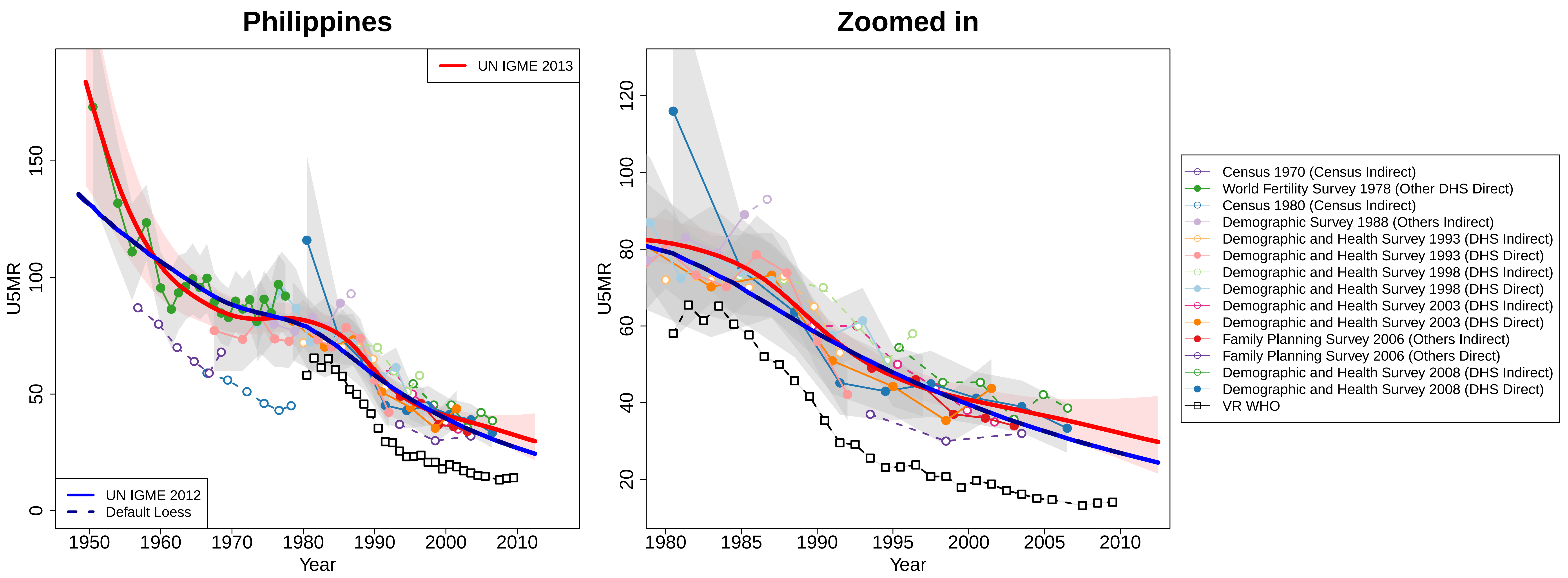

Philippines
Zoomed in
UN IGME 2013
UN IGME 2012
Default Loess
U5MR
Year
0
50
100
150
1950
1960
1970
1980
1990
2000
2010
20
40
60
80
100
120
1980
1985
1990
1995
2000
2005
2010
Census 1970 (Census Indirect)
World Fertility Survey 1978 (Other DHS Direct)
Census 1980 (Census Indirect)
Demographic Survey 1988 (Others Indirect)
Demographic and Health Survey 1993 (DHS Indirect)
Demographic and Health Survey 1993 (DHS Direct)
Demographic and Health Survey 1998 (DHS Indirect)
Demographic and Health Survey 1998 (DHS Direct)
Demographic and Health Survey 2003 (DHS Indirect)
Demographic and Health Survey 2003 (DHS Direct)
Family Planning Survey 2006 (Others Indirect)
Family Planning Survey 2006 (Others Direct)
Demographic and Health Survey 2008 (DHS Indirect)
Demographic and Health Survey 2008 (DHS Direct)
VR WHO

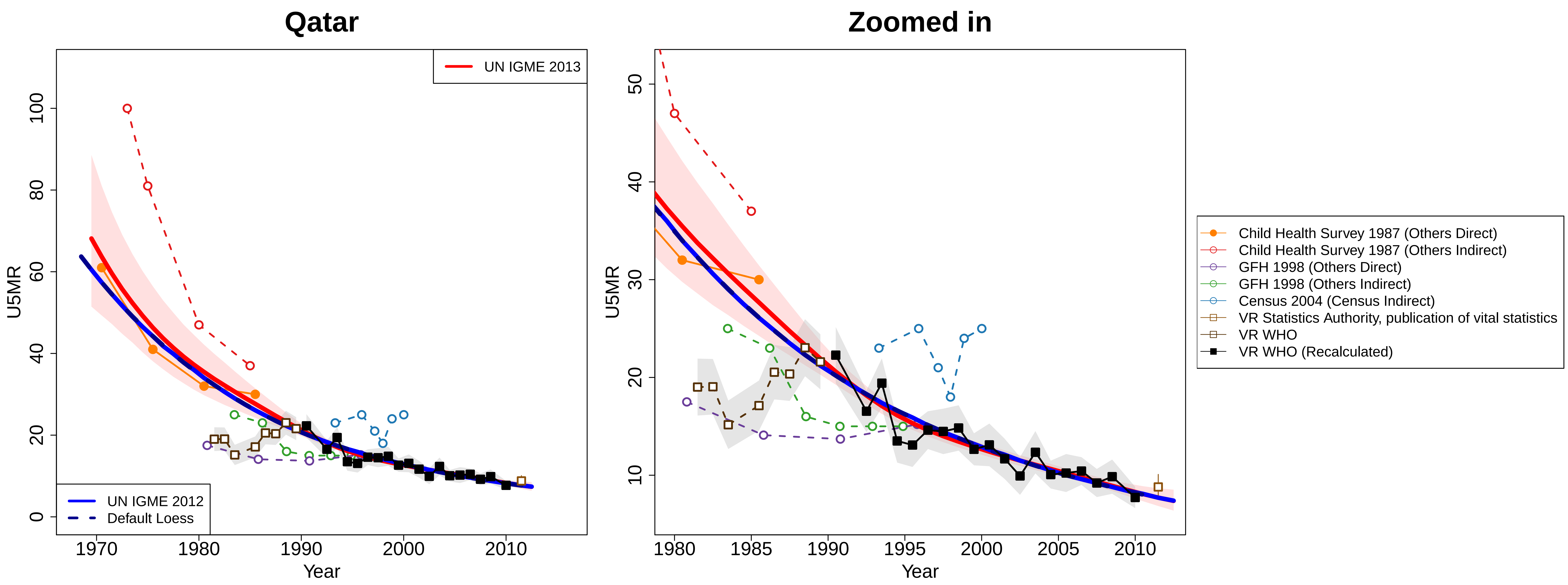

Qatar
Zoomed in
UN IGME 2013
UN IGME 2012
Default Loess
U5MR
Year
0
20
40
60
80
100
1970
1980
1990
2000
2010
10
20
30
40
50
1980
1985
1990
1995
2000
2005
2010
Child Health Survey 1987 (Others Direct)
Child Health Survey 1987 (Others Indirect)
GFH 1998 (Others Direct)
GFH 1998 (Others Indirect)
Census 2004 (Census Indirect)
VR Statistics Authority, publication of vital statistics
VR WHO
VR WHO (Recalculated)

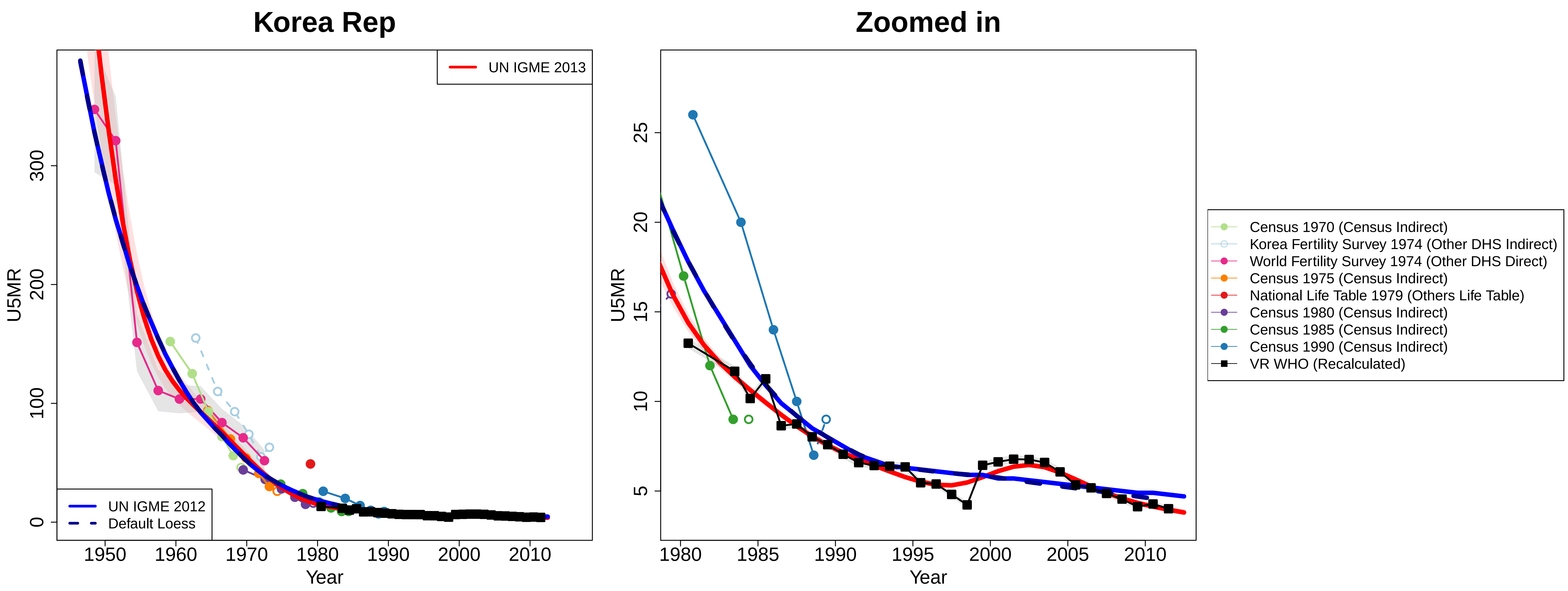

Korea Rep
Zoomed in
U5MR
Year
UN IGME 2013
UN IGME 2012
Default Loess
Census 1970 (Census Indirect)
Korea Fertility Survey 1974 (Other DHS Indirect)
World Fertility Survey 1974 (Other DHS Direct)
Census 1975 (Census Indirect)
National Life Table 1979 (Others Life Table)
Census 1980 (Census Indirect)
Census 1985 (Census Indirect)
Census 1990 (Census Indirect)
VR WHO (Recalculated)

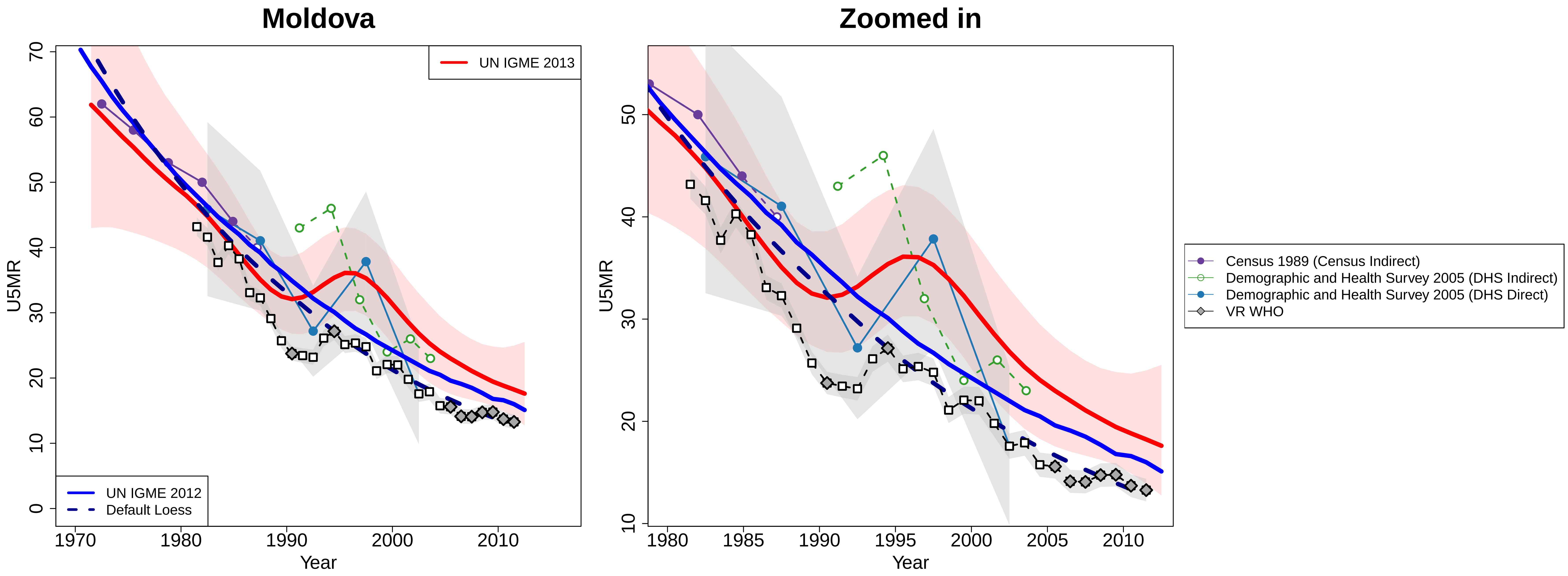

Moldova
Zoomed in
UN IGME 2013
UN IGME 2012
Default Loess
Census 1989 (Census Indirect)
Demographic and Health Survey 2005 (DHS Indirect)
Demographic and Health Survey 2005 (DHS Direct)
VR WHO
U5MR
Year

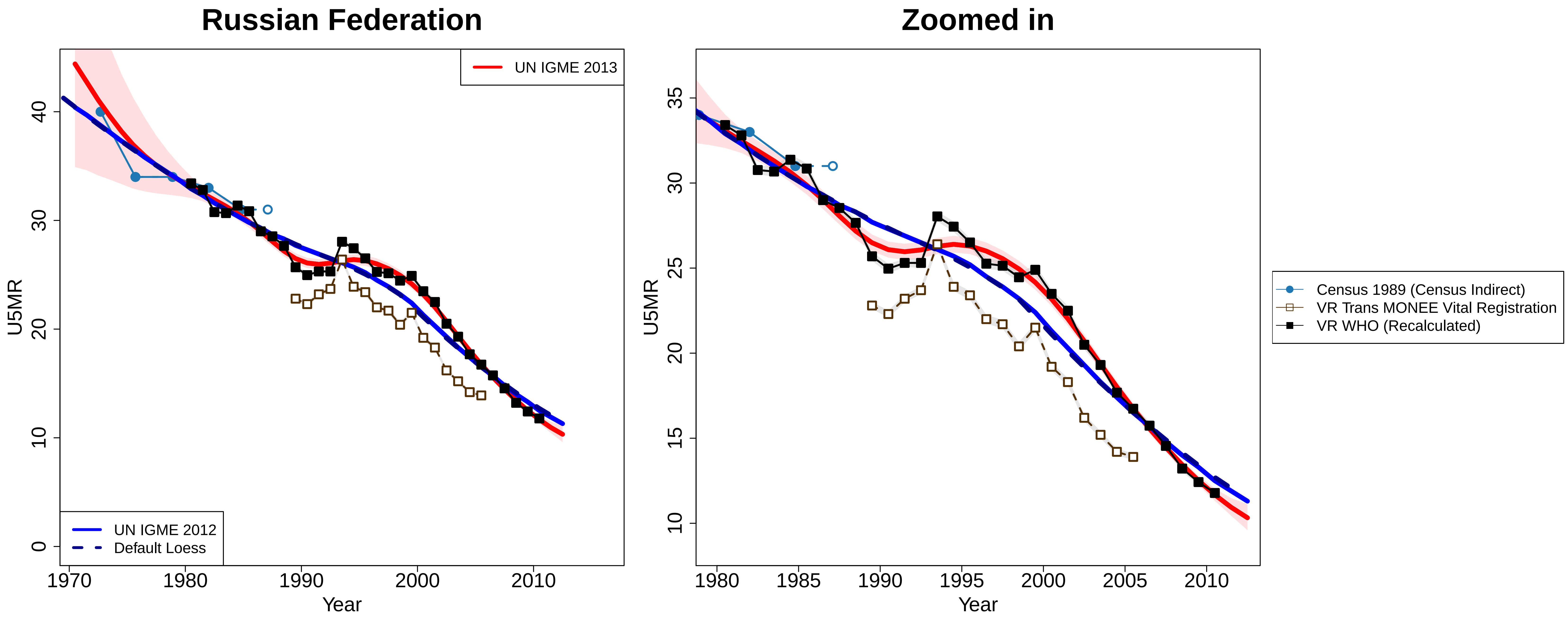

Russian Federation
Zoomed in
U5MR
Year
UN IGME 2013
UN IGME 2012
Default Loess
Census 1989 (Census Indirect)
VR Trans MONEE Vital Registration
VR WHO (Recalculated)

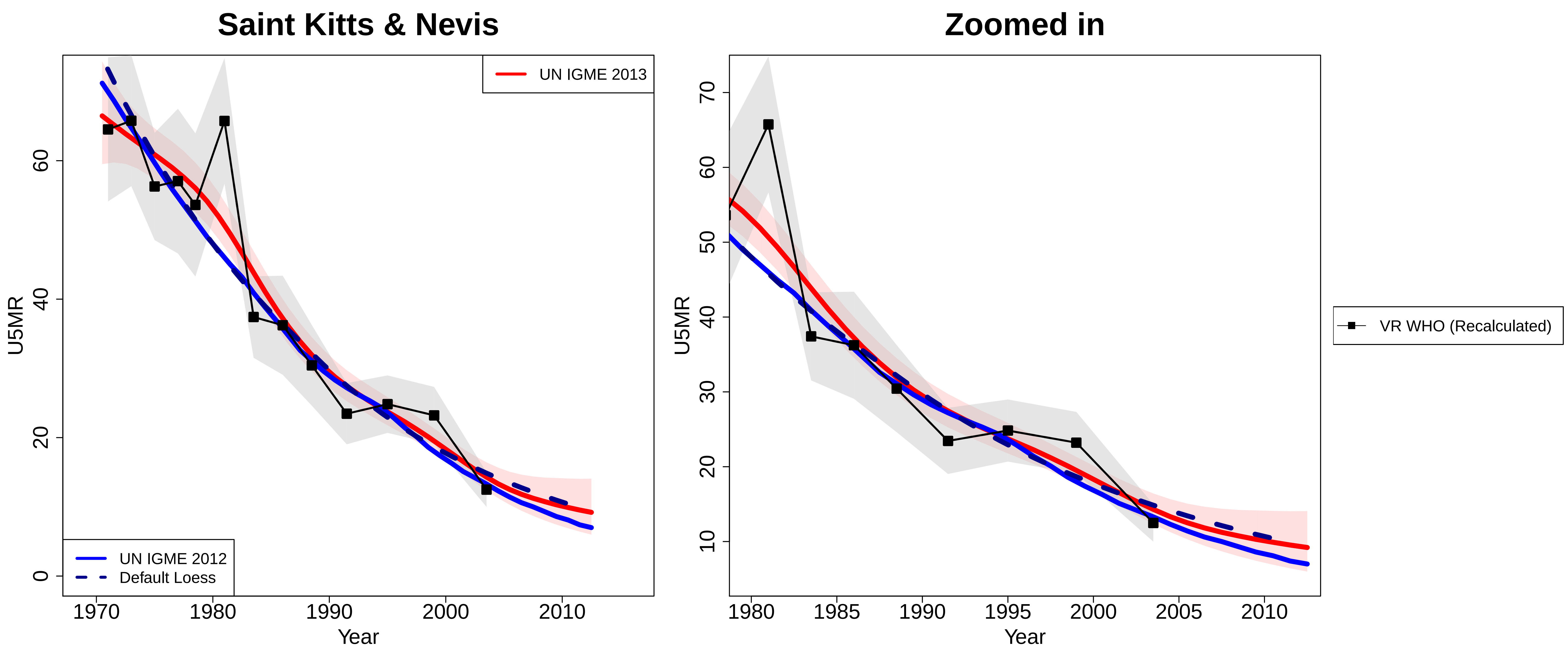

Saint Kitts & Nevis
Zoomed in
UN IGME 2013
UN IGME 2012
Default Loess
VR WHO (Recalculated)
U5MR
Year
0
20
40
60
10
30
50
70
1970
1980
1990
2000
2010
1985
1995
2005

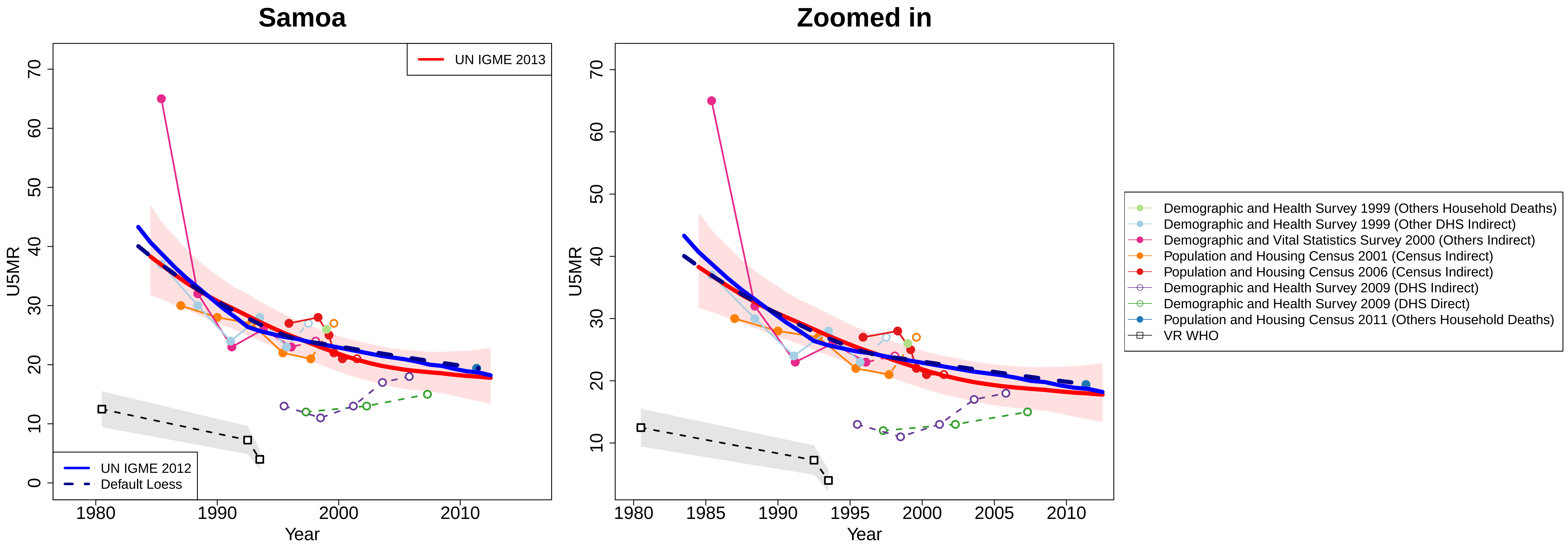
Samoa
Zoomed in
UN IGME 2013
UN IGME 2012
Default Loess
U5MR
Year
0
10
20
30
40
50
60
70
1980
1985
1990
1995
2000
2005
2010
Demographic and Health Survey 1999 (Others Household Deaths)
Demographic and Health Survey 1999 (Other DHS Indirect)
Demographic and Vital Statistics Survey 2000 (Others Indirect)
Population and Housing Census 2001 (Census Indirect)
Population and Housing Census 2006 (Census Indirect)
Demographic and Health Survey 2009 (DHS Indirect)
Demographic and Health Survey 2009 (DHS Direct)
Population and Housing Census 2011 (Others Household Deaths)
VR WHO

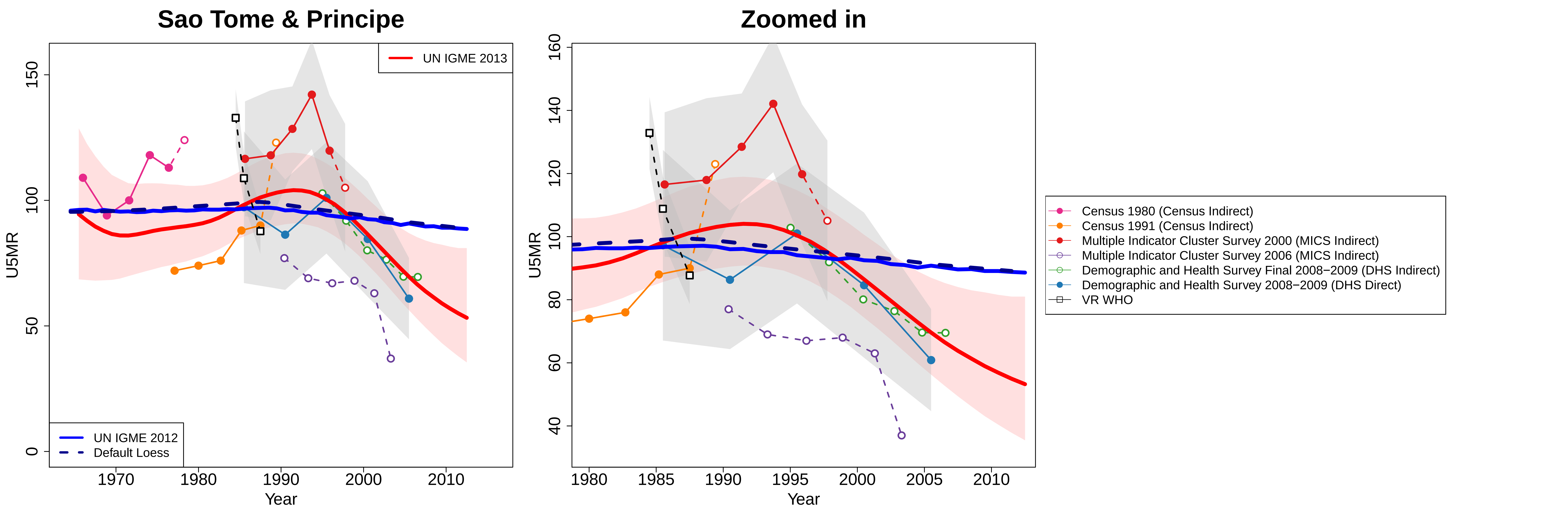

Sao Tome & Principe
Zoomed in
UN IGME 2013
UN IGME 2012
Default Loess
U5MR
Year
Census 1980 (Census Indirect)
Census 1991 (Census Indirect)
Multiple Indicator Cluster Survey 2000 (MICS Indirect)
Multiple Indicator Cluster Survey 2006 (MICS Indirect)
Demographic and Health Survey Final 2008−2009 (DHS Indirect)
Demographic and Health Survey 2008−2009 (DHS Direct)
VR WHO

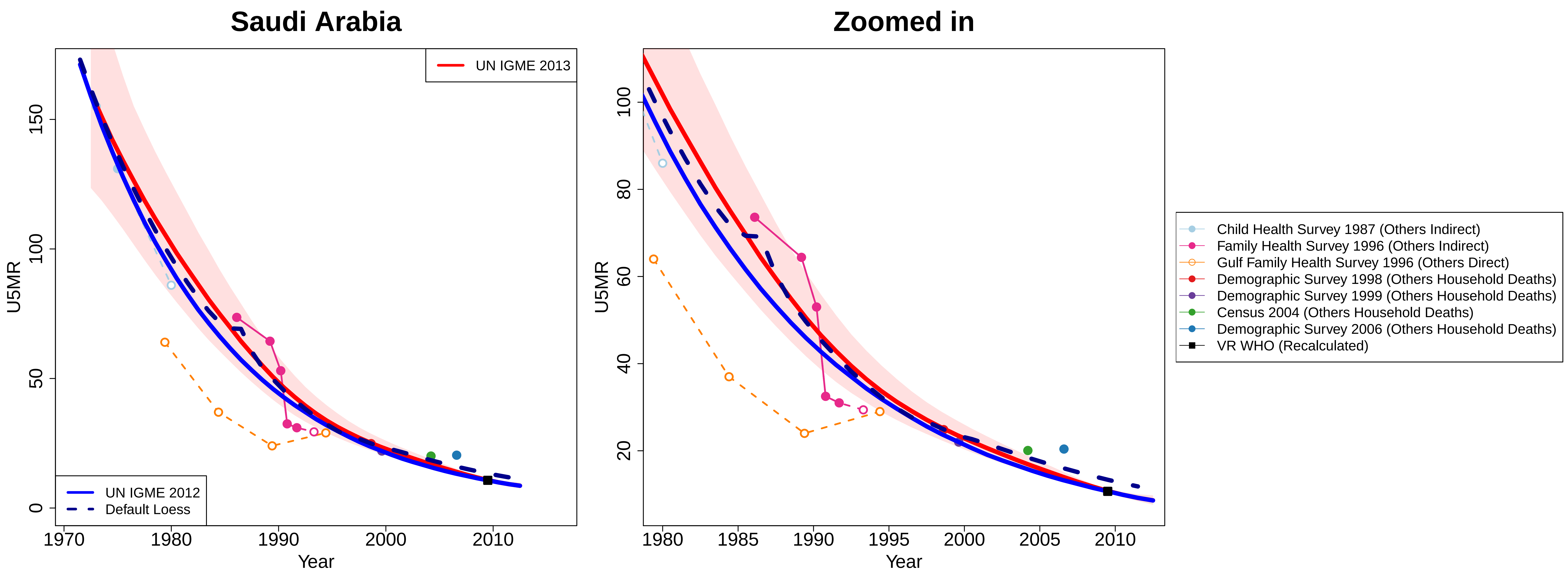

Saudi Arabia
Zoomed in
U5MR
Year
UN IGME 2013
UN IGME 2012
Default Loess
Child Health Survey 1987 (Others Indirect)
Family Health Survey 1996 (Others Indirect)
Gulf Family Health Survey 1996 (Others Direct)
Demographic Survey 1998 (Others Household Deaths)
Demographic Survey 1999 (Others Household Deaths)
Census 2004 (Others Household Deaths)
Demographic Survey 2006 (Others Household Deaths)
VR WHO (Recalculated)

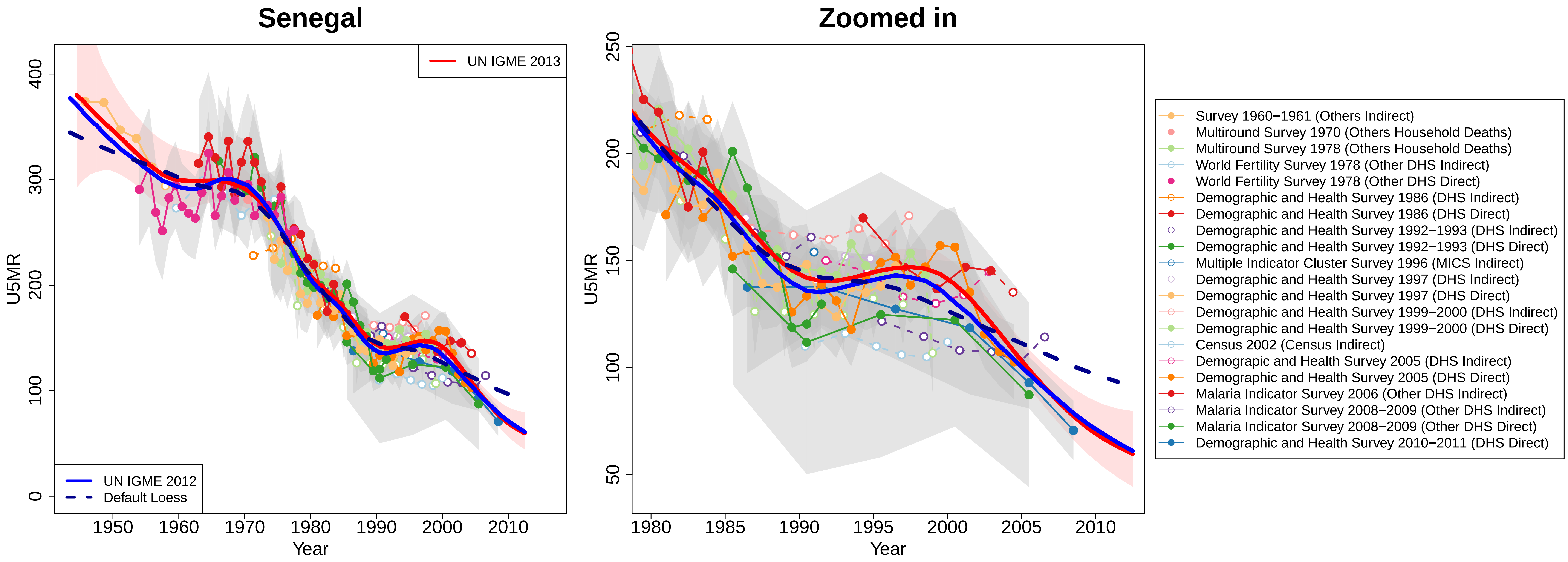

Senegal
Zoomed in
UN IGME 2013
UN IGME 2012
Default Loess
U5MR
Year
Survey 1960−1961 (Others Indirect)
Multiround Survey 1970 (Others Household Deaths)
Multiround Survey 1978 (Others Household Deaths)
World Fertility Survey 1978 (Other DHS Indirect)
World Fertility Survey 1978 (Other DHS Direct)
Demographic and Health Survey 1986 (DHS Indirect)
Demographic and Health Survey 1986 (DHS Direct)
Demographic and Health Survey 1992−1993 (DHS Indirect)
Demographic and Health Survey 1992−1993 (DHS Direct)
Multiple Indicator Cluster Survey 1996 (MICS Indirect)
Demographic and Health Survey 1997 (DHS Indirect)
Demographic and Health Survey 1997 (DHS Direct)
Demographic and Health Survey 1999−2000 (DHS Indirect)
Demographic and Health Survey 1999−2000 (DHS Direct)
Census 2002 (Census Indirect)
Demograpic and Health Survey 2005 (DHS Indirect)
Demographic and Health Survey 2005 (DHS Direct)
Malaria Indicator Survey 2006 (Other DHS Indirect)
Malaria Indicator Survey 2008−2009 (Other DHS Indirect)
Malaria Indicator Survey 2008−2009 (Other DHS Direct)
Demographic and Health Survey 2010−2011 (DHS Direct)

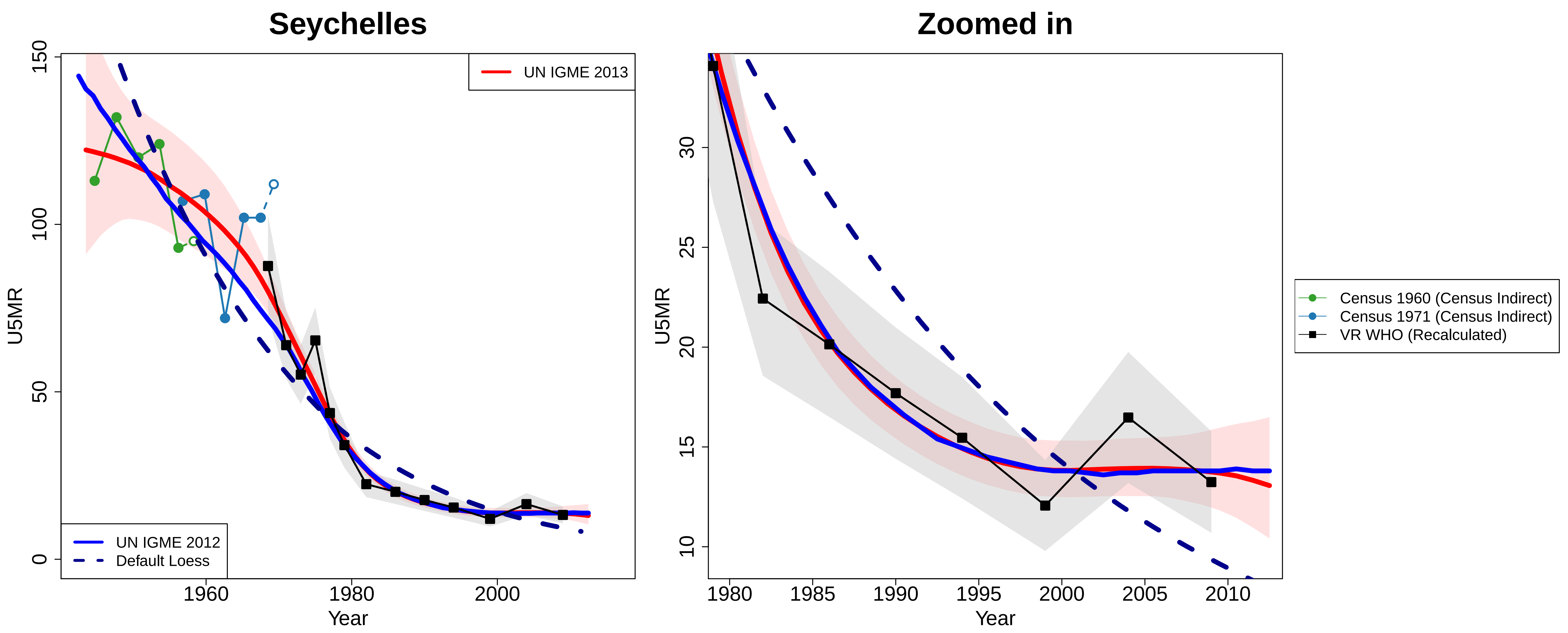

Seychelles
Zoomed in
UN IGME 2013
UN IGME 2012
Default Loess
Census 1960 (Census Indirect)
Census 1971 (Census Indirect)
VR WHO (Recalculated)
U5MR
Year
0
50
100
150
1960
1980
2000
10
15
20
25
30
1980
1985
1990
1995
2000
2005
2010

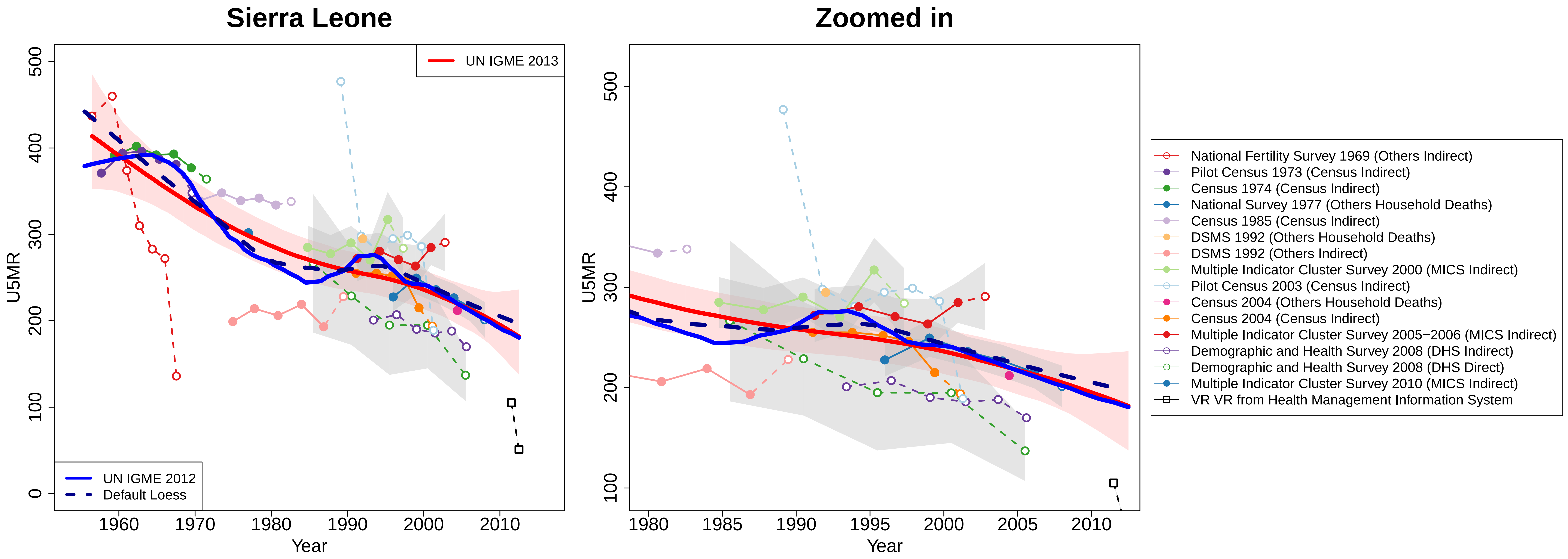
Sierra Leone
Zoomed in
U5MR
Year
UN IGME 2013
UN IGME 2012
Default Loess
National Fertility Survey 1969 (Others Indirect)
Pilot Census 1973 (Census Indirect)
Census 1974 (Census Indirect)
National Survey 1977 (Others Household Deaths)
Census 1985 (Census Indirect)
DSMS 1992 (Others Household Deaths)
DSMS 1992 (Others Indirect)
Multiple Indicator Cluster Survey 2000 (MICS Indirect)
Pilot Census 2003 (Census Indirect)
Census 2004 (Others Household Deaths)
Census 2004 (Census Indirect)
Multiple Indicator Cluster Survey 2005−2006 (MICS Indirect)
Demographic and Health Survey 2008 (DHS Indirect)
Demographic and Health Survey 2008 (DHS Direct)
Multiple Indicator Cluster Survey 2010 (MICS Indirect)
VR VR from Health Management Information System

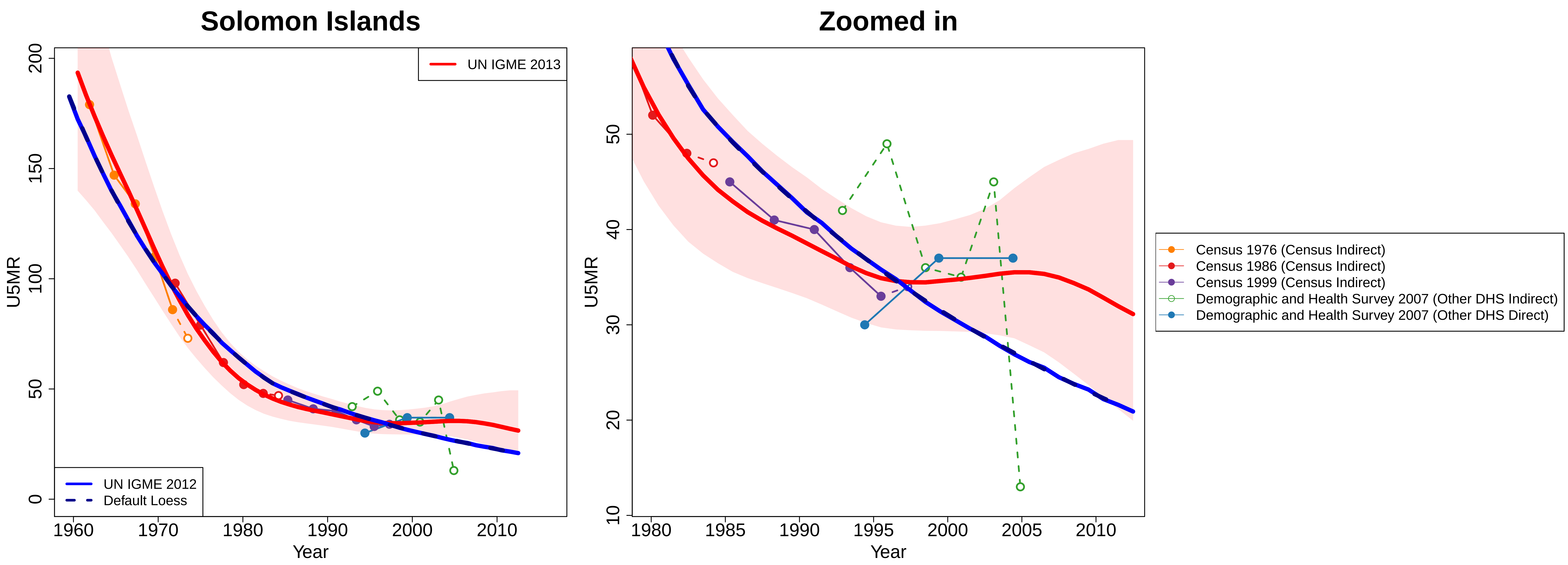

Solomon Islands
Zoomed in
U5MR
Year
UN IGME 2013
UN IGME 2012
Default Loess
Census 1976 (Census Indirect)
Census 1986 (Census Indirect)
Census 1999 (Census Indirect)
Demographic and Health Survey 2007 (Other DHS Indirect)
Demographic and Health Survey 2007 (Other DHS Direct)

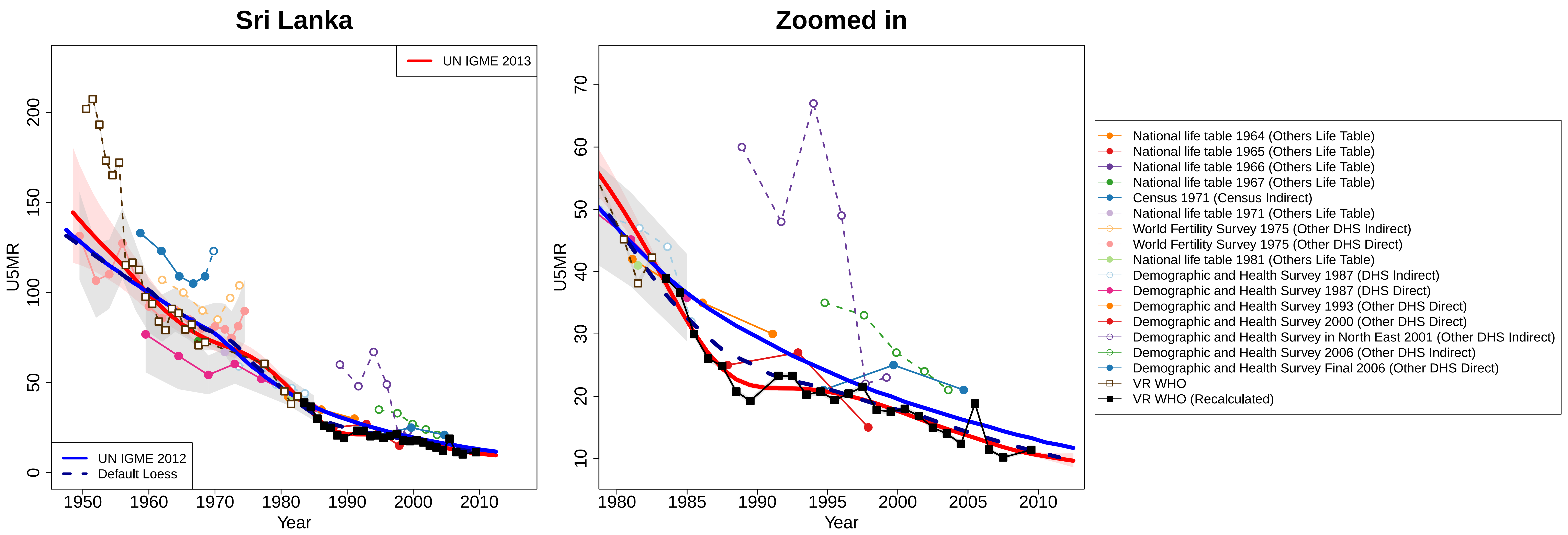

Sri Lanka
Zoomed in
UN IGME 2013
UN IGME 2012
Default Loess
U5MR
Year
National life table 1964 (Others Life Table)
National life table 1965 (Others Life Table)
National life table 1966 (Others Life Table)
National life table 1967 (Others Life Table)
Census 1971 (Census Indirect)
National life table 1971 (Others Life Table)
World Fertility Survey 1975 (Other DHS Indirect)
World Fertility Survey 1975 (Other DHS Direct)
National life table 1981 (Others Life Table)
Demographic and Health Survey 1987 (DHS Indirect)
Demographic and Health Survey 1987 (DHS Direct)
Demographic and Health Survey 1993 (Other DHS Direct)
Demographic and Health Survey 2000 (Other DHS Direct)
Demographic and Health Survey in North East 2001 (Other DHS Indirect)
Demographic and Health Survey 2006 (Other DHS Indirect)
Demographic and Health Survey Final 2006 (Other DHS Direct)
VR WHO
VR WHO (Recalculated)

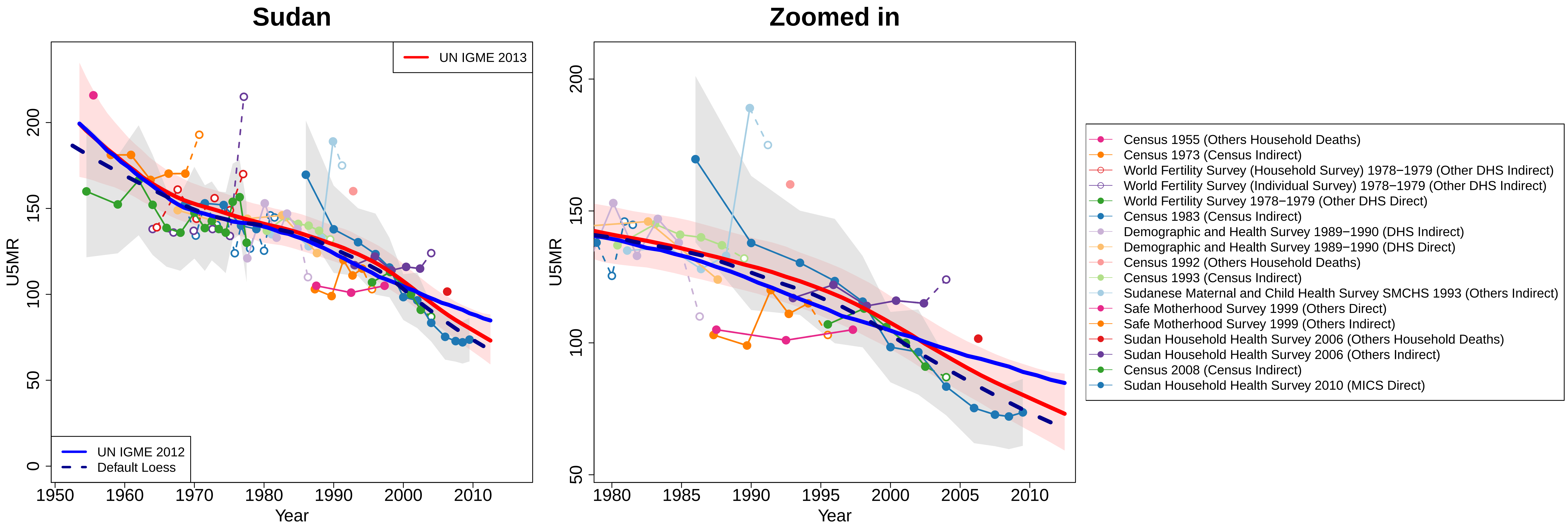

Sudan
Zoomed in
UN IGME 2013
UN IGME 2012
Default Loess
U5MR
Year
Census 1955 (Others Household Deaths)
Census 1973 (Census Indirect)
World Fertility Survey (Household Survey) 1978−1979 (Other DHS Indirect)
World Fertility Survey (Individual Survey) 1978−1979 (Other DHS Indirect)
World Fertility Survey 1978−1979 (Other DHS Direct)
Census 1983 (Census Indirect)
Demographic and Health Survey 1989−1990 (DHS Indirect)
Demographic and Health Survey 1989−1990 (DHS Direct)
Census 1992 (Others Household Deaths)
Census 1993 (Census Indirect)
Sudanese Maternal and Child Health Survey SMCHS 1993 (Others Indirect)
Safe Motherhood Survey 1999 (Others Direct)
Safe Motherhood Survey 1999 (Others Indirect)
Sudan Household Health Survey 2006 (Others Household Deaths)
Sudan Household Health Survey 2006 (Others Indirect)
Census 2008 (Census Indirect)
Sudan Household Health Survey 2010 (MICS Direct)

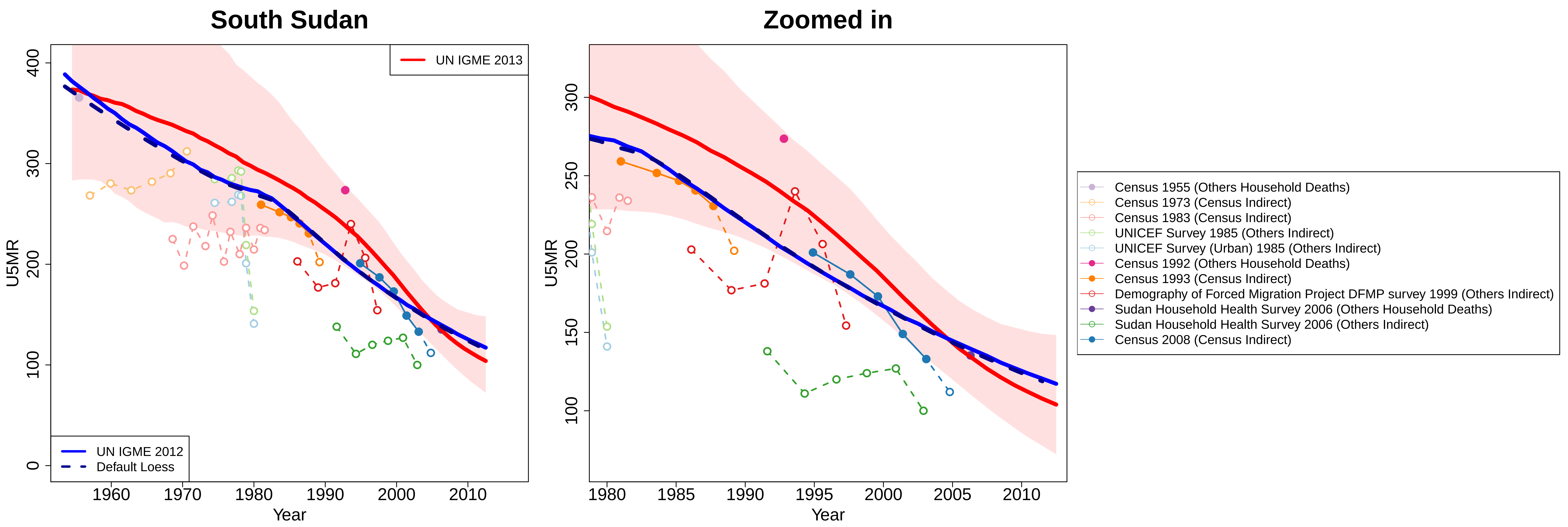

South Sudan
Zoomed in
U5MR
Year
UN IGME 2013
UN IGME 2012
Default Loess
Census 1955 (Others Household Deaths)
Census 1973 (Census Indirect)
Census 1983 (Census Indirect)
UNICEF Survey 1985 (Others Indirect)
UNICEF Survey (Urban) 1985 (Others Indirect)
Census 1992 (Others Household Deaths)
Census 1993 (Census Indirect)
Demography of Forced Migration Project DFMP survey 1999 (Others Indirect)
Sudan Household Health Survey 2006 (Others Household Deaths)
Sudan Household Health Survey 2006 (Others Indirect)
Census 2008 (Census Indirect)

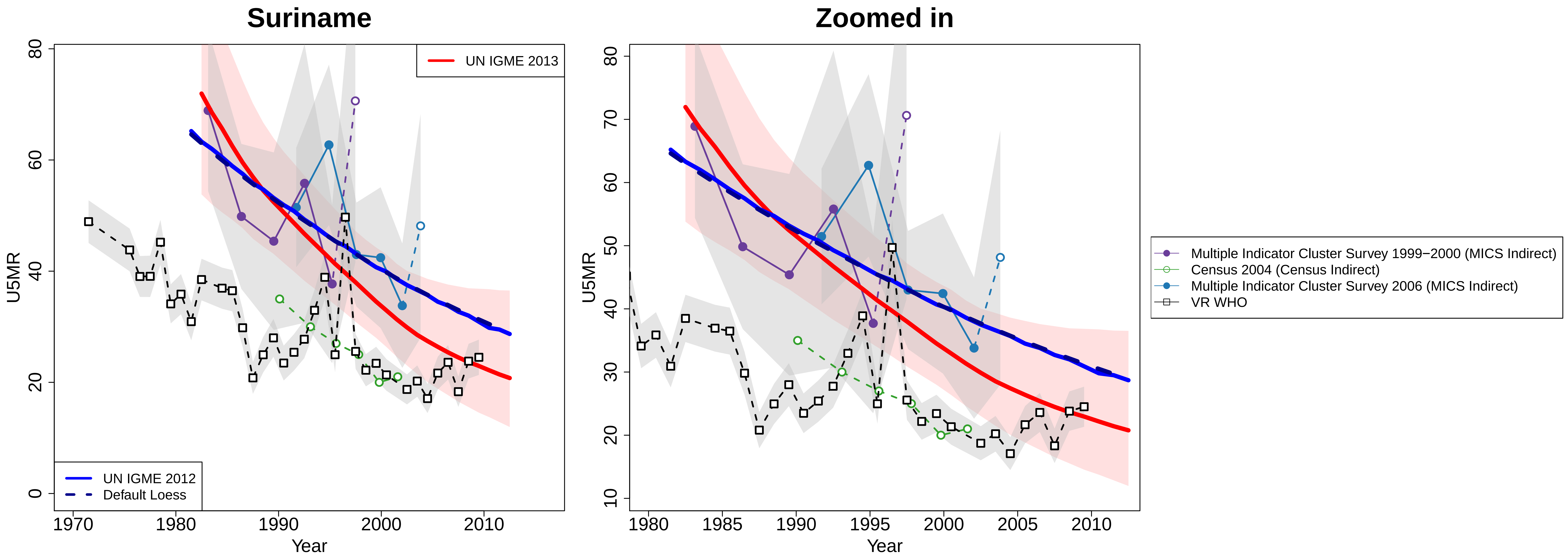

Suriname
Zoomed in
U5MR
Year
UN IGME 2013
UN IGME 2012
Default Loess
Multiple Indicator Cluster Survey 1999−2000 (MICS Indirect)
Census 2004 (Census Indirect)
Multiple Indicator Cluster Survey 2006 (MICS Indirect)
VR WHO

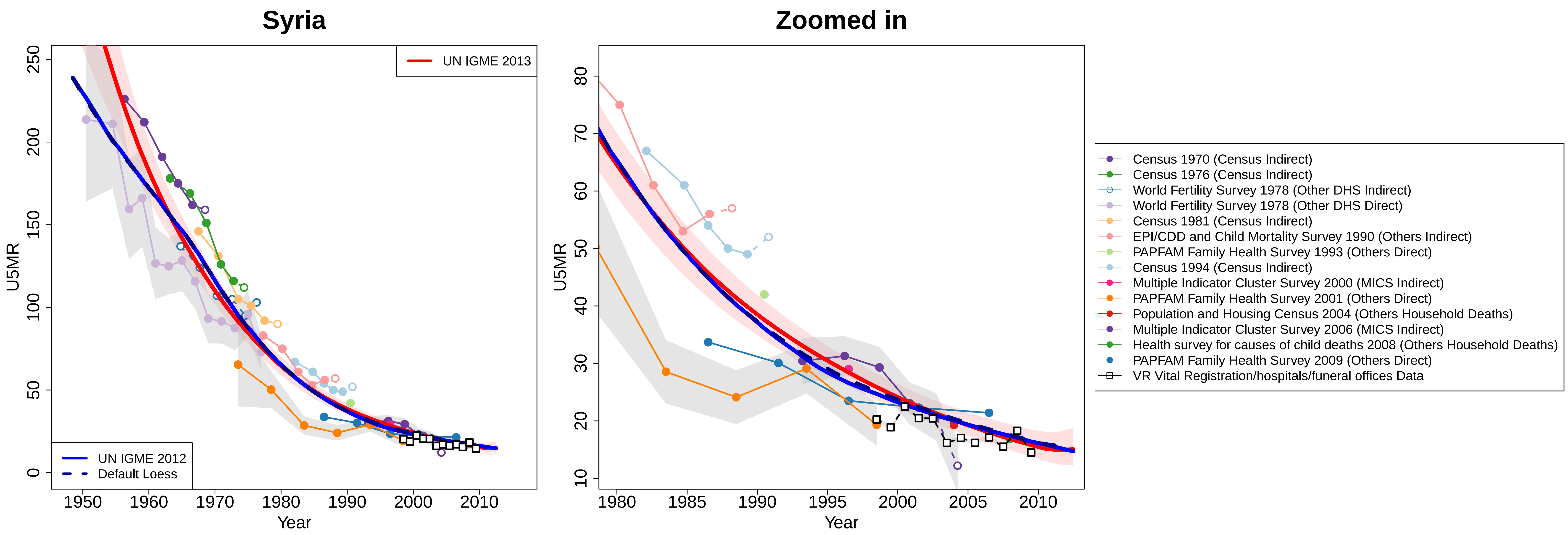
Syria
Zoomed in
U5MR
Year
UN IGME 2013
UN IGME 2012
Default Loess
Census 1970 (Census Indirect)
Census 1976 (Census Indirect)
World Fertility Survey 1978 (Other DHS Indirect)
World Fertility Survey 1978 (Other DHS Direct)
Census 1981 (Census Indirect)
EPI/CDD and Child Mortality Survey 1990 (Others Indirect)
PAPFAM Family Health Survey 1993 (Others Direct)
Census 1994 (Census Indirect)
Multiple Indicator Cluster Survey 2000 (MICS Indirect)
PAPFAM Family Health Survey 2001 (Others Direct)
Population and Housing Census 2004 (Others Household Deaths)
Multiple Indicator Cluster Survey 2006 (MICS Indirect)
Health survey for causes of child deaths 2008 (Others Household Deaths)
PAPFAM Family Health Survey 2009 (Others Direct)
VR Vital Registration/hospitals/funeral offices Data

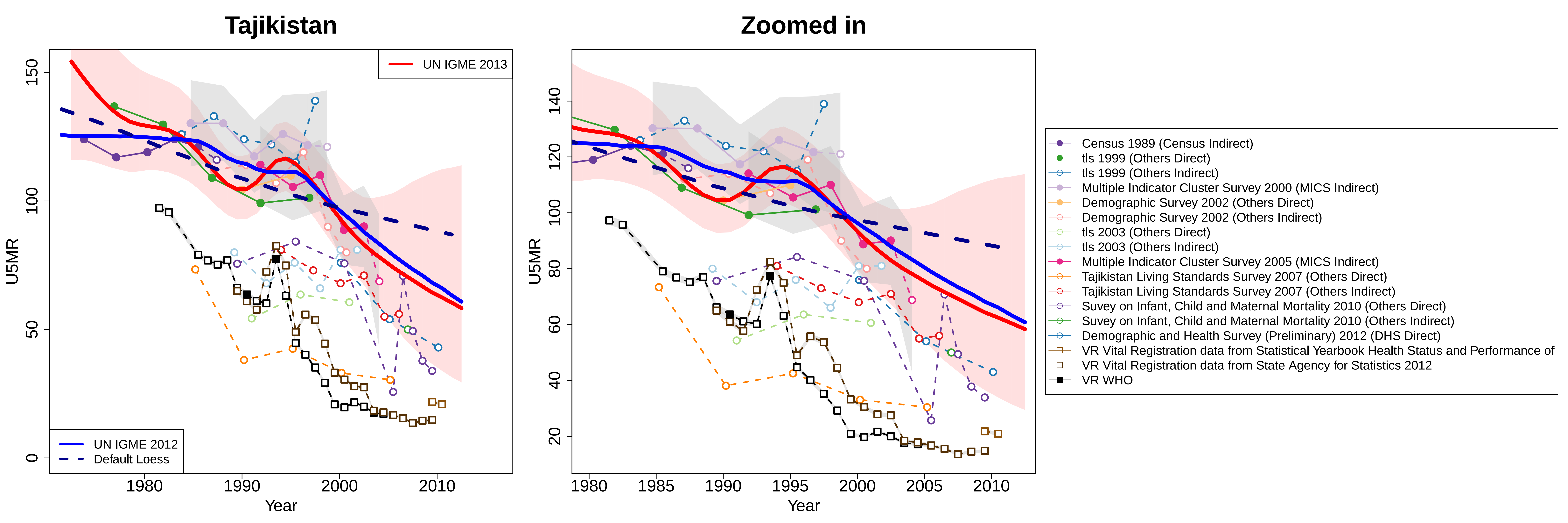
Tajikistan
Zoomed in
UN IGME 2013
UN IGME 2012
Default Loess
U5MR
Year
150
100
50
0
140
120
100
80
60
40
20
1980
1985
1990
1995
2000
2005
2010
Census 1989 (Census Indirect)
tls 1999 (Others Direct)
tls 1999 (Others Indirect)
Multiple Indicator Cluster Survey 2000 (MICS Indirect)
Demographic Survey 2002 (Others Direct)
Demographic Survey 2002 (Others Indirect)
tls 2003 (Others Direct)
tls 2003 (Others Indirect)
Multiple Indicator Cluster Survey 2005 (MICS Indirect)
Tajikistan Living Standards Survey 2007 (Others Direct)
Tajikistan Living Standards Survey 2007 (Others Indirect)
Suvey on Infant, Child and Maternal Mortality 2010 (Others Direct)
Suvey on Infant, Child and Maternal Mortality 2010 (Others Indirect)
Demographic and Health Survey (Preliminary) 2012 (DHS Direct)
VR Vital Registration data from Statistical Yearbook Health Status and Performance of
VR Vital Registration data from State Agency for Statistics 2012
VR WHO

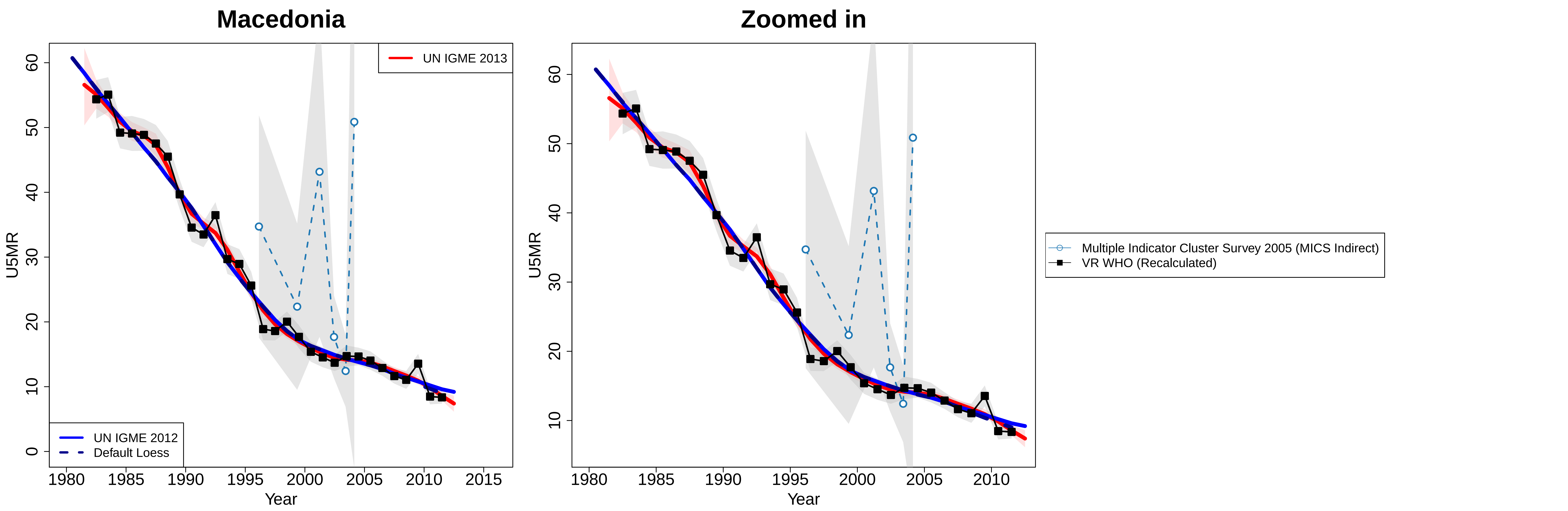

Macedonia
Zoomed in
U5MR
Year
UN IGME 2013
UN IGME 2012
Default Loess
Multiple Indicator Cluster Survey 2005 (MICS Indirect)
VR WHO (Recalculated)

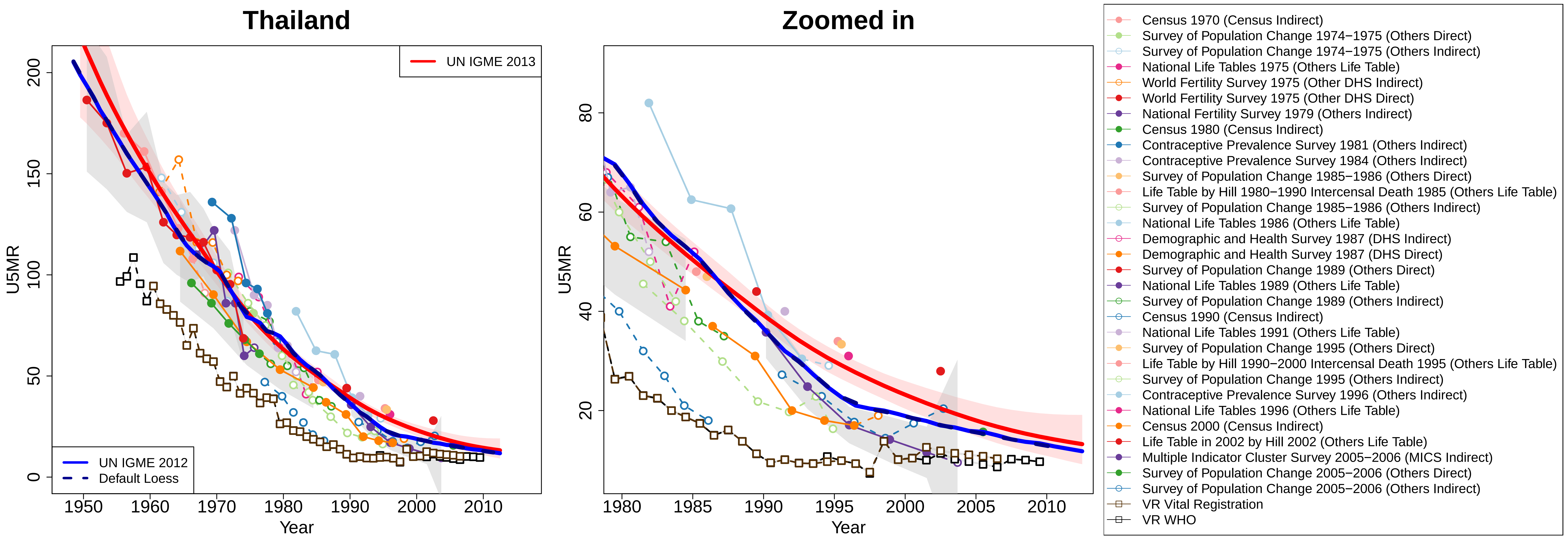
Thailand
Zoomed in
U5MR
Year
UN IGME 2013
UN IGME 2012
Default Loess
Census 1970 (Census Indirect)
Survey of Population Change 1974−1975 (Others Direct)
Survey of Population Change 1974−1975 (Others Indirect)
National Life Tables 1975 (Others Life Table)
World Fertility Survey 1975 (Other DHS Indirect)
World Fertility Survey 1975 (Other DHS Direct)
National Fertility Survey 1979 (Others Indirect)
Census 1980 (Census Indirect)
Contraceptive Prevalence Survey 1981 (Others Indirect)
Contraceptive Prevalence Survey 1984 (Others Indirect)
Survey of Population Change 1985−1986 (Others Direct)
Life Table by Hill 1980−1990 Intercensal Death 1985 (Others Life Table)
Survey of Population Change 1985−1986 (Others Indirect)
National Life Tables 1986 (Others Life Table)
Demographic and Health Survey 1987 (DHS Indirect)
Demographic and Health Survey 1987 (DHS Direct)
Survey of Population Change 1989 (Others Direct)
National Life Tables 1989 (Others Life Table)
Survey of Population Change 1989 (Others Indirect)
Census 1990 (Census Indirect)
National Life Tables 1991 (Others Life Table)
Survey of Population Change 1995 (Others Direct)
Life Table by Hill 1990−2000 Intercensal Death 1995 (Others Life Table)
Survey of Population Change 1995 (Others Indirect)
Contraceptive Prevalence Survey 1996 (Others Indirect)
National Life Tables 1996 (Others Life Table)
Census 2000 (Census Indirect)
Life Table in 2002 by Hill 2002 (Others Life Table)
Multiple Indicator Cluster Survey 2005−2006 (MICS Indirect)
Survey of Population Change 2005−2006 (Others Direct)
Survey of Population Change 2005−2006 (Others Indirect)
VR Vital Registration
VR WHO

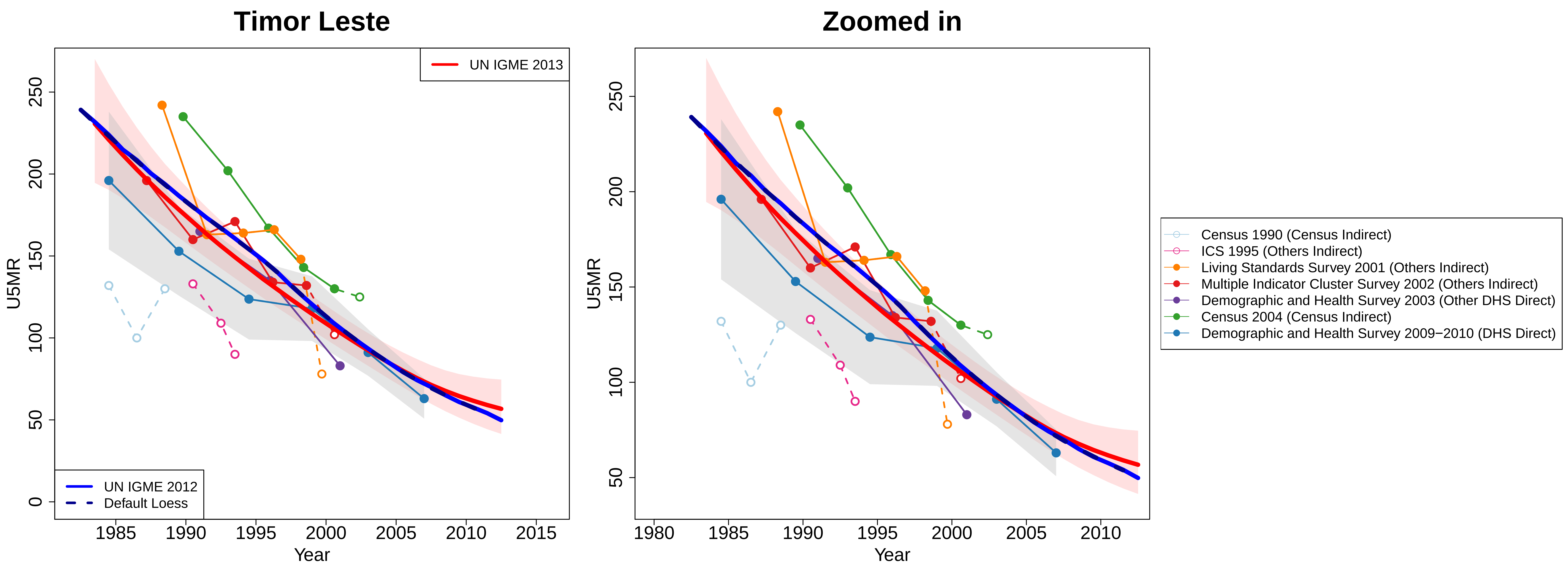

Timor Leste
Zoomed in
U5MR
Year
UN IGME 2013
UN IGME 2012
Default Loess
Census 1990 (Census Indirect)
ICS 1995 (Others Indirect)
Living Standards Survey 2001 (Others Indirect)
Multiple Indicator Cluster Survey 2002 (Others Indirect)
Demographic and Health Survey 2003 (Other DHS Direct)
Census 2004 (Census Indirect)
Demographic and Health Survey 2009−2010 (DHS Direct)

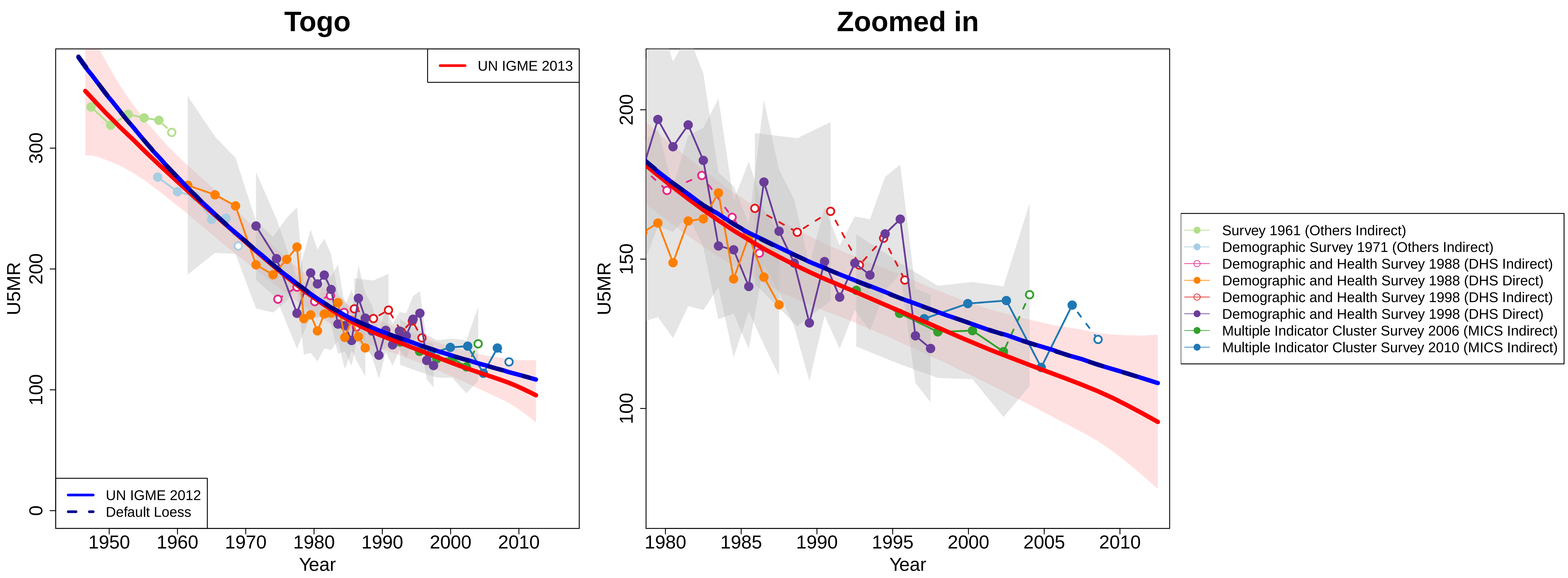
Togo
Zoomed in
UN IGME 2013
UN IGME 2012
Default Loess
U5MR
Year
Survey 1961 (Others Indirect)
Demographic Survey 1971 (Others Indirect)
Demographic and Health Survey 1988 (DHS Indirect)
Demographic and Health Survey 1988 (DHS Direct)
Demographic and Health Survey 1998 (DHS Indirect)
Demographic and Health Survey 1998 (DHS Direct)
Multiple Indicator Cluster Survey 2006 (MICS Indirect)
Multiple Indicator Cluster Survey 2010 (MICS Indirect)

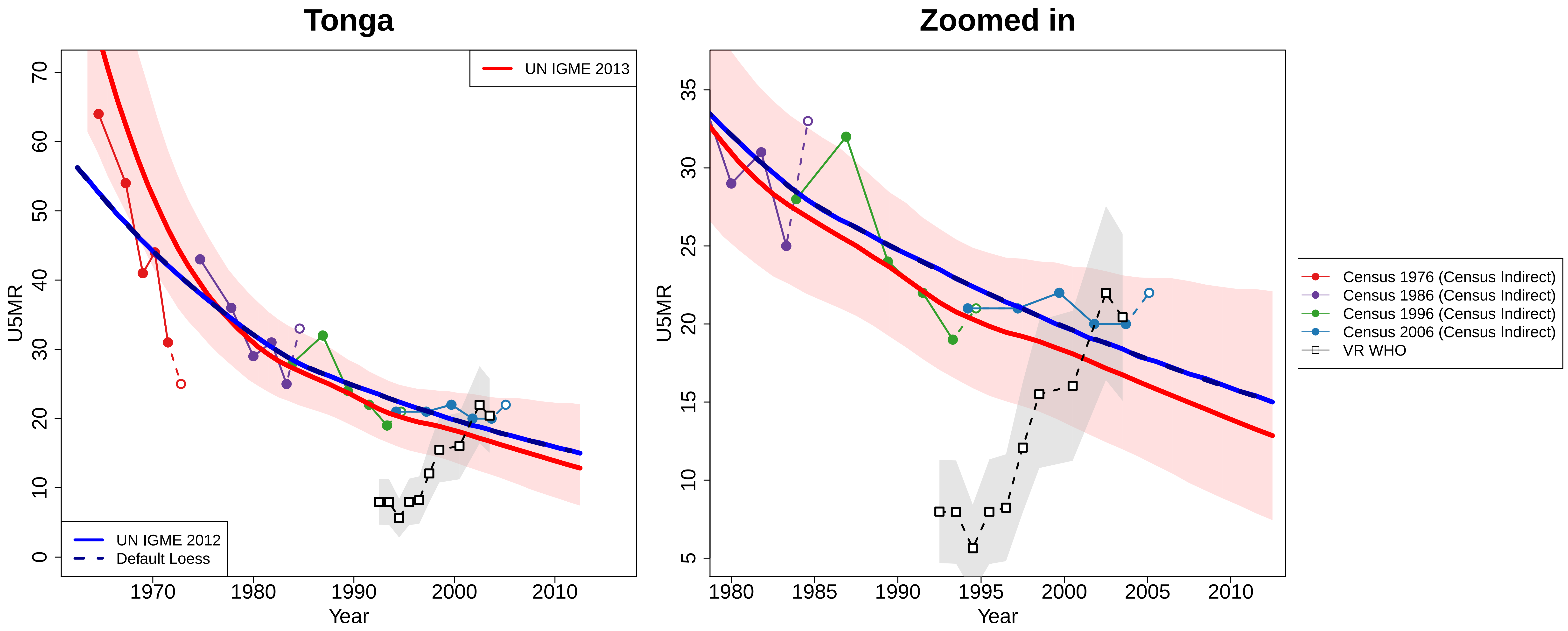

Tonga
Zoomed in
UN IGME 2013
UN IGME 2012
Default Loess
U5MR
Year
Census 1976 (Census Indirect)
Census 1986 (Census Indirect)
Census 1996 (Census Indirect)
Census 2006 (Census Indirect)
VR WHO

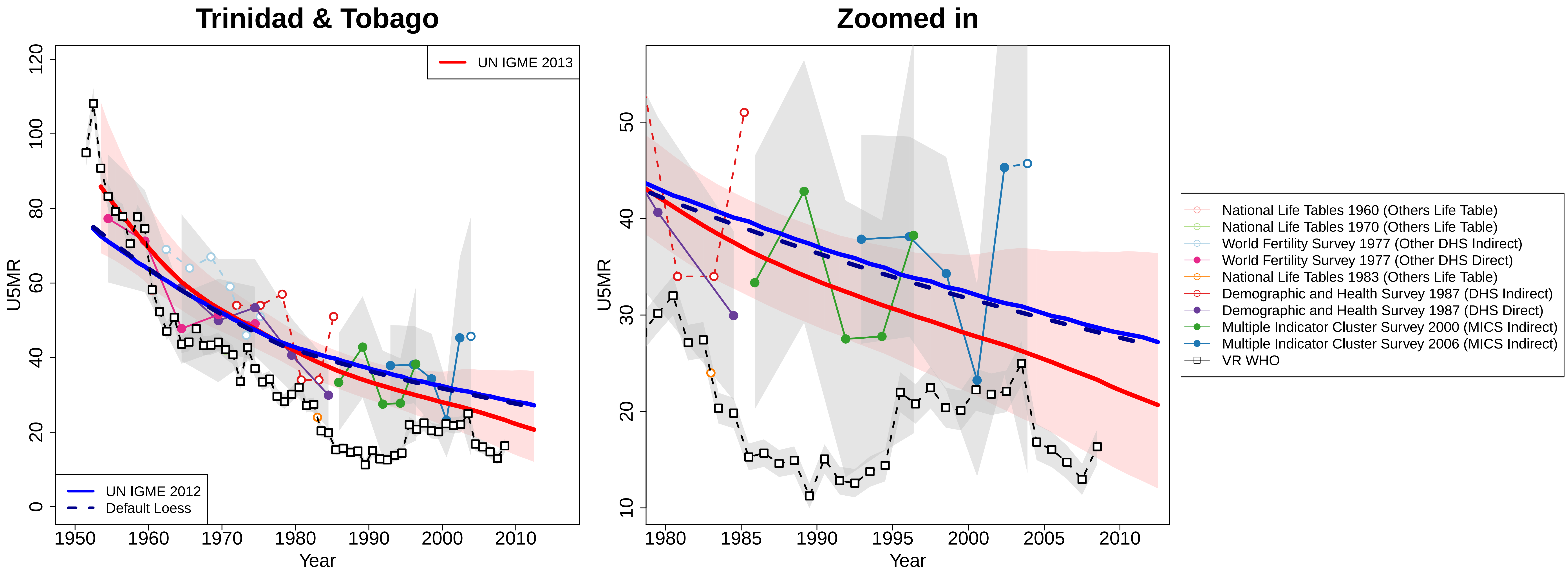
Trinidad & Tobago
Zoomed in
U5MR
Year
UN IGME 2013
UN IGME 2012
Default Loess
0
20
40
60
80
100
120
1950
1960
1970
1980
1990
2000
2010
10
20
30
40
50
1980
1985
1990
1995
2000
2005
2010
National Life Tables 1960 (Others Life Table)
National Life Tables 1970 (Others Life Table)
World Fertility Survey 1977 (Other DHS Indirect)
World Fertility Survey 1977 (Other DHS Direct)
National Life Tables 1983 (Others Life Table)
Demographic and Health Survey 1987 (DHS Indirect)
Demographic and Health Survey 1987 (DHS Direct)
Multiple Indicator Cluster Survey 2000 (MICS Indirect)
Multiple Indicator Cluster Survey 2006 (MICS Indirect)
VR WHO

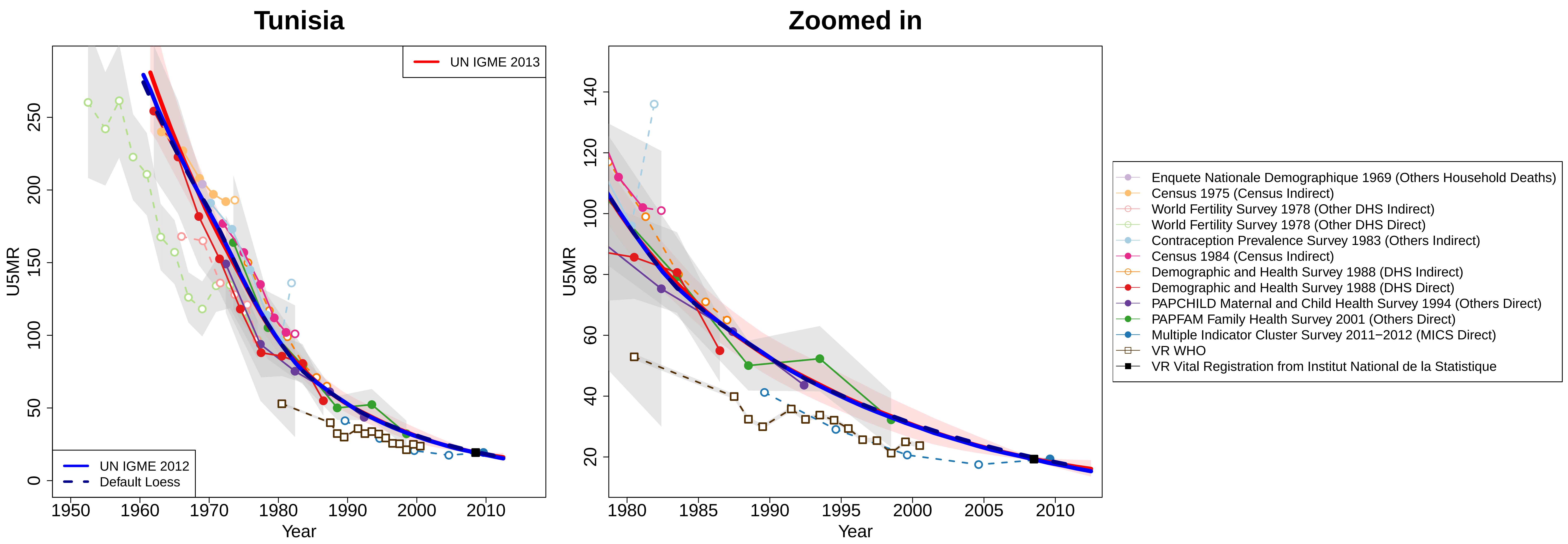

Tunisia
Zoomed in
UN IGME 2013
UN IGME 2012
Default Loess
U5MR
Year
Enquete Nationale Demographique 1969 (Others Household Deaths)
Census 1975 (Census Indirect)
World Fertility Survey 1978 (Other DHS Indirect)
World Fertility Survey 1978 (Other DHS Direct)
Contraception Prevalence Survey 1983 (Others Indirect)
Census 1984 (Census Indirect)
Demographic and Health Survey 1988 (DHS Indirect)
Demographic and Health Survey 1988 (DHS Direct)
PAPCHILD Maternal and Child Health Survey 1994 (Others Direct)
PAPFAM Family Health Survey 2001 (Others Direct)
Multiple Indicator Cluster Survey 2011–2012 (MICS Direct)
VR WHO
VR Vital Registration from Institut National de la Statistique

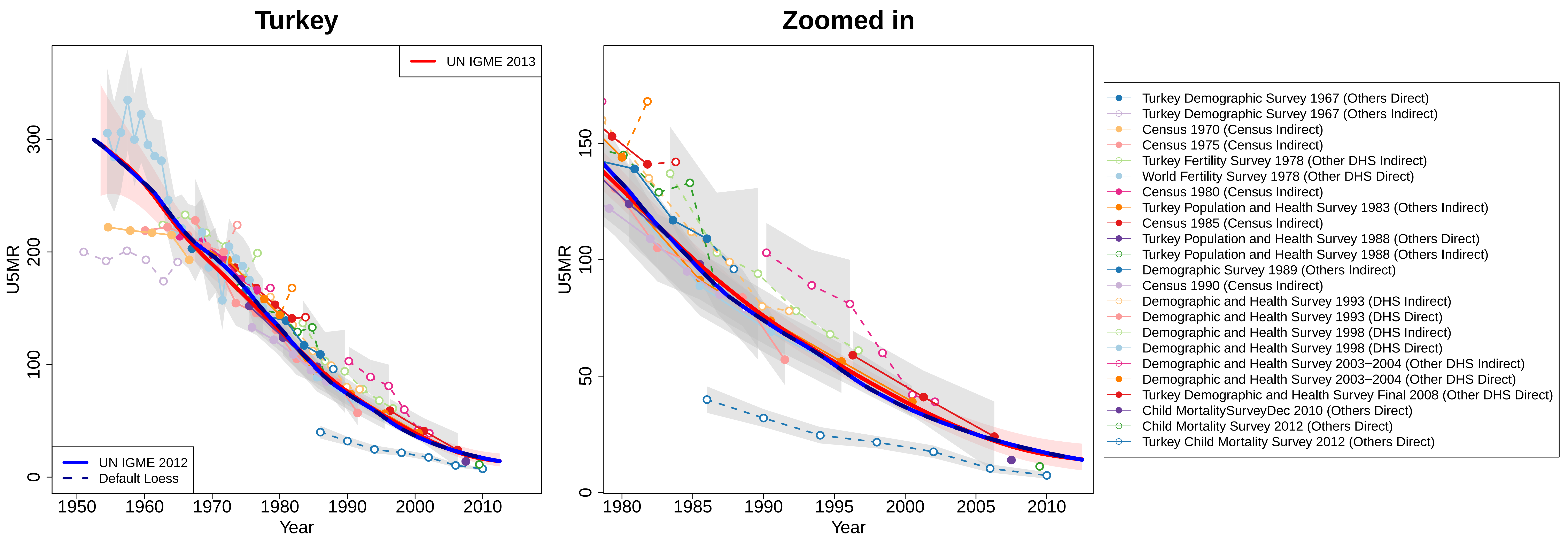
Turkey
Zoomed in
U5MR
Year
UN IGME 2013
UN IGME 2012
Default Loess
Turkey Demographic Survey 1967 (Others Direct)
Turkey Demographic Survey 1967 (Others Indirect)
Census 1970 (Census Indirect)
Census 1975 (Census Indirect)
Turkey Fertility Survey 1978 (Other DHS Indirect)
World Fertility Survey 1978 (Other DHS Direct)
Census 1980 (Census Indirect)
Turkey Population and Health Survey 1983 (Others Indirect)
Census 1985 (Census Indirect)
Turkey Population and Health Survey 1988 (Others Direct)
Turkey Population and Health Survey 1988 (Others Indirect)
Demographic Survey 1989 (Others Indirect)
Census 1990 (Census Indirect)
Demographic and Health Survey 1993 (DHS Indirect)
Demographic and Health Survey 1993 (DHS Direct)
Demographic and Health Survey 1998 (DHS Indirect)
Demographic and Health Survey 1998 (DHS Direct)
Demographic and Health Survey 2003−2004 (Other DHS Indirect)
Demographic and Health Survey 2003−2004 (Other DHS Direct)
Turkey Demographic and Health Survey Final 2008 (Other DHS Direct)
Child MortalitySurveyDec 2010 (Others Direct)
Child Mortality Survey 2012 (Others Direct)
Turkey Child Mortality Survey 2012 (Others Direct)

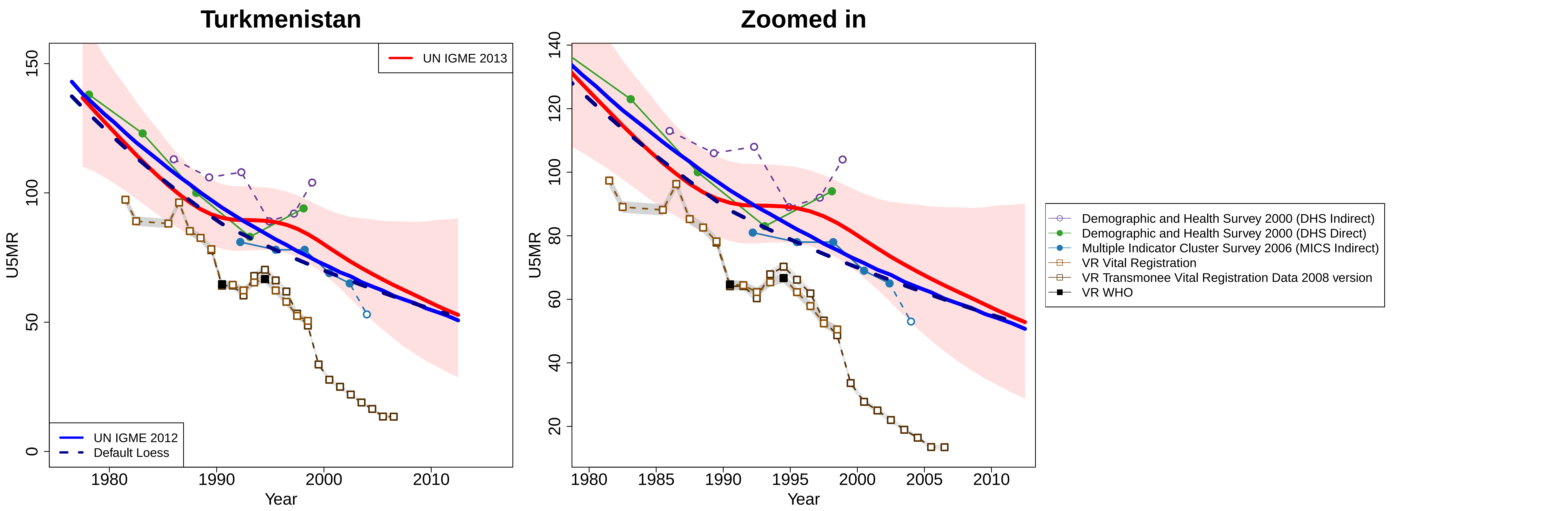

Turkmenistan
Zoomed in
U5MR
Year
UN IGME 2013
UN IGME 2012
Default Loess
Demographic and Health Survey 2000 (DHS Indirect)
Demographic and Health Survey 2000 (DHS Direct)
Multiple Indicator Cluster Survey 2006 (MICS Indirect)
VR Vital Registration
VR Transmonee Vital Registration Data 2008 version
VR WHO
0
50
100
150
1980
1990
2000
2010
20
40
60
80
100
120
140
1985
1995
2005

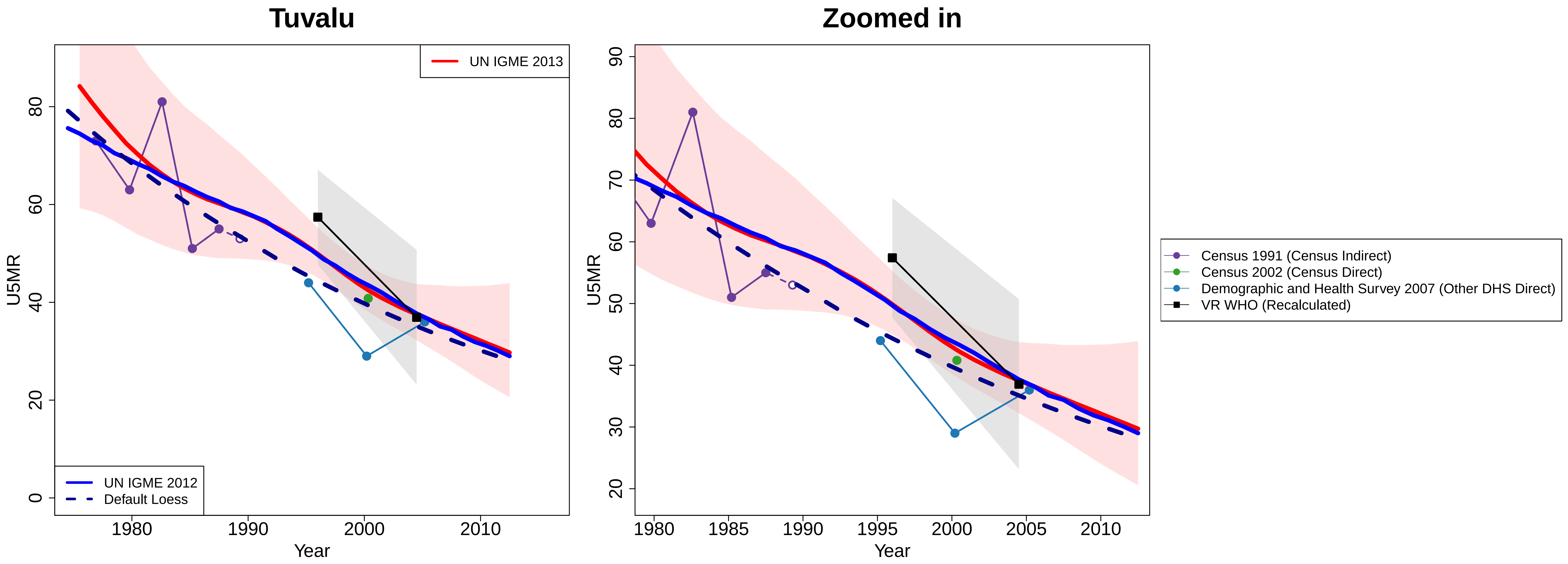

Tuvalu
Zoomed in
UN IGME 2013
UN IGME 2012
Default Loess
U5MR
Year
Census 1991 (Census Indirect)
Census 2002 (Census Direct)
Demographic and Health Survey 2007 (Other DHS Direct)
VR WHO (Recalculated)

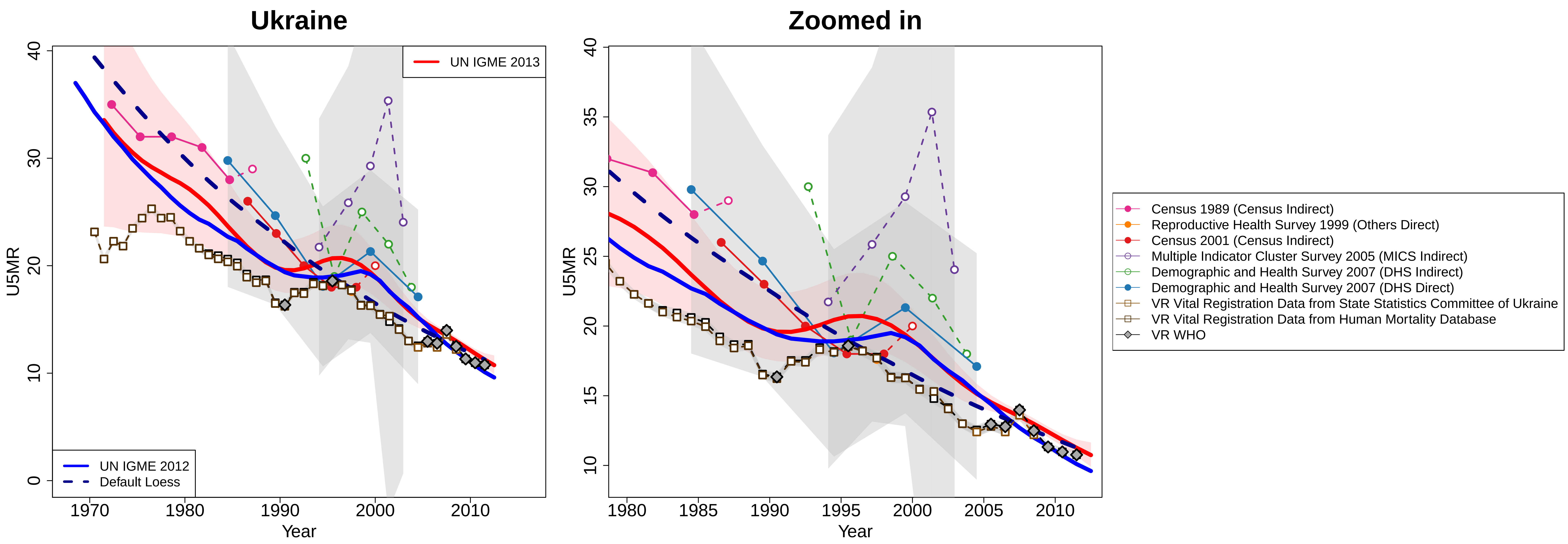

Ukraine
Zoomed in
U5MR
Year
UN IGME 2013
UN IGME 2012
Default Loess
Census 1989 (Census Indirect)
Reproductive Health Survey 1999 (Others Direct)
Census 2001 (Census Indirect)
Multiple Indicator Cluster Survey 2005 (MICS Indirect)
Demographic and Health Survey 2007 (DHS Indirect)
Demographic and Health Survey 2007 (DHS Direct)
VR Vital Registration Data from State Statistics Committee of Ukraine
VR Vital Registration Data from Human Mortality Database
VR WHO

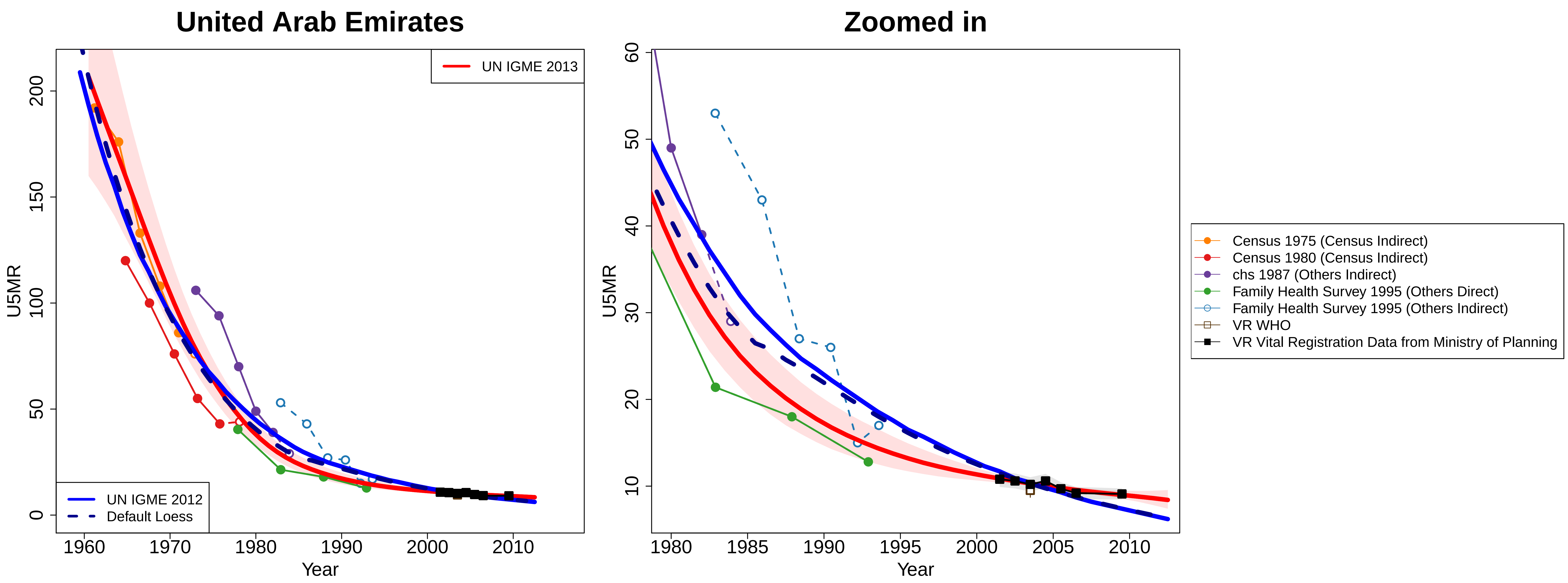

United Arab Emirates
Zoomed in
U5MR
Year
UN IGME 2013
UN IGME 2012
Default Loess
Census 1975 (Census Indirect)
Census 1980 (Census Indirect)
chs 1987 (Others Indirect)
Family Health Survey 1995 (Others Direct)
Family Health Survey 1995 (Others Indirect)
VR WHO
VR Vital Registration Data from Ministry of Planning

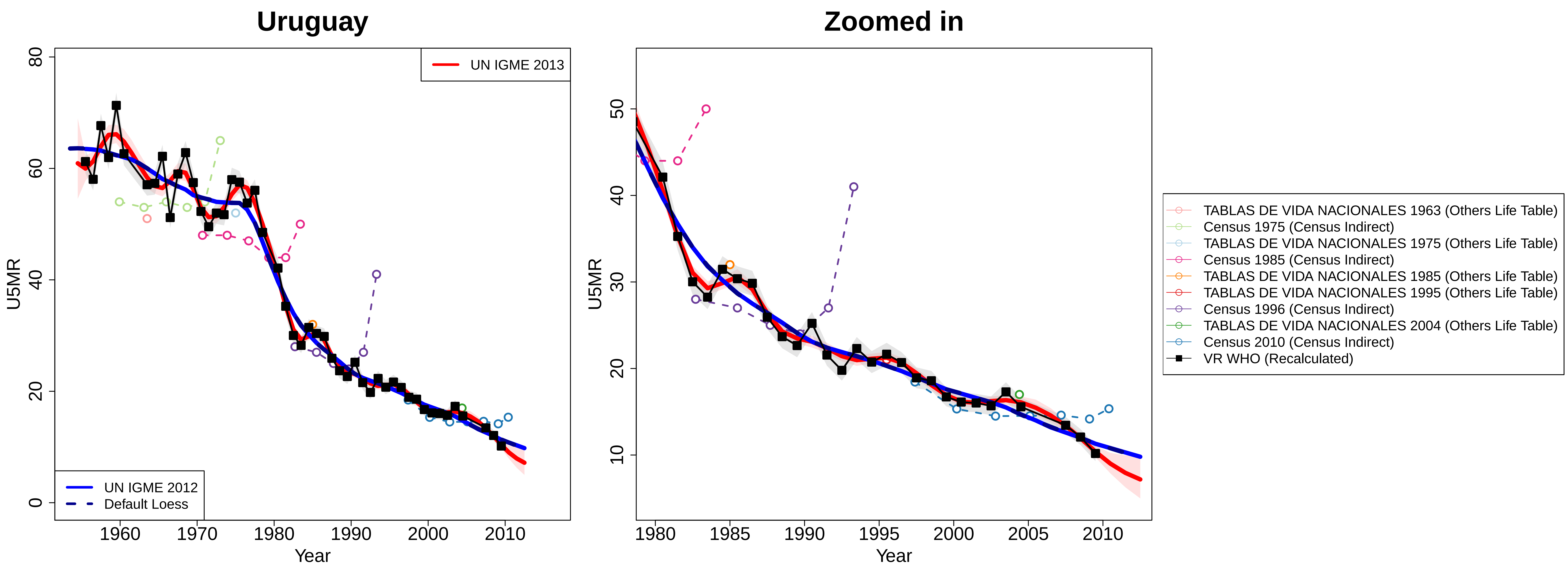
Uruguay
Zoomed in
UN IGME 2013
UN IGME 2012
Default Loess
U5MR
Year
TABLAS DE VIDA NACIONALES 1963 (Others Life Table)
Census 1975 (Census Indirect)
TABLAS DE VIDA NACIONALES 1975 (Others Life Table)
Census 1985 (Census Indirect)
TABLAS DE VIDA NACIONALES 1985 (Others Life Table)
TABLAS DE VIDA NACIONALES 1995 (Others Life Table)
Census 1996 (Census Indirect)
TABLAS DE VIDA NACIONALES 2004 (Others Life Table)
Census 2010 (Census Indirect)
VR WHO (Recalculated)

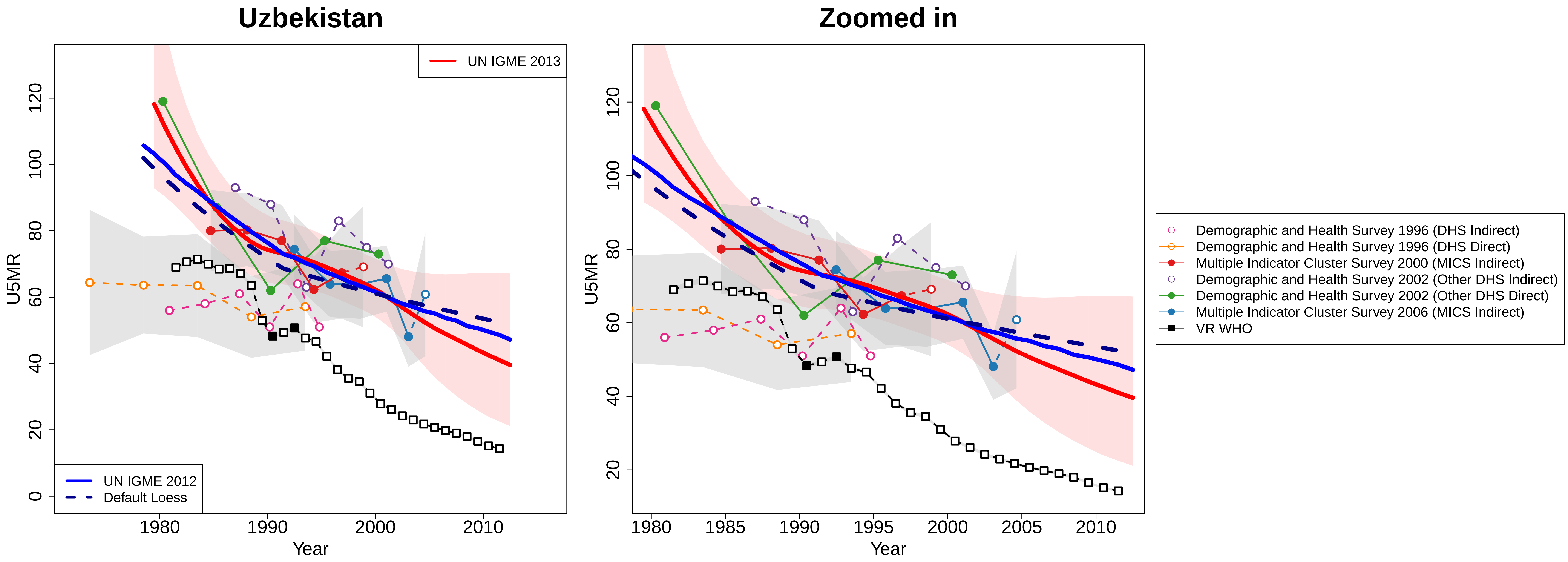

Uzbekistan
Zoomed in
U5MR
Year
UN IGME 2013
UN IGME 2012
Default Loess
Demographic and Health Survey 1996 (DHS Indirect)
Demographic and Health Survey 1996 (DHS Direct)
Multiple Indicator Cluster Survey 2000 (MICS Indirect)
Demographic and Health Survey 2002 (Other DHS Indirect)
Demographic and Health Survey 2002 (Other DHS Direct)
Multiple Indicator Cluster Survey 2006 (MICS Indirect)
VR WHO

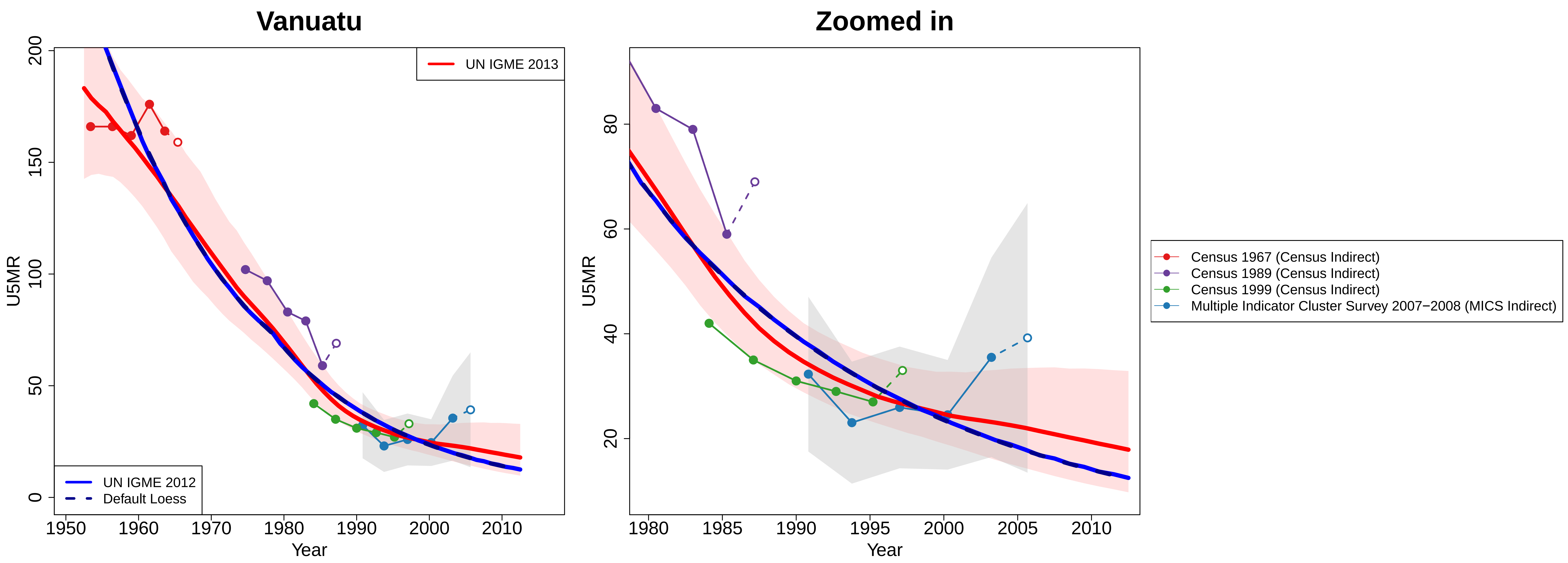
Vanuatu
Zoomed in
U5MR
Year
UN IGME 2013
UN IGME 2012
Default Loess
Census 1967 (Census Indirect)
Census 1989 (Census Indirect)
Census 1999 (Census Indirect)
Multiple Indicator Cluster Survey 2007−2008 (MICS Indirect)
0
50
100
150
200
1950
1960
1970
1980
1990
2000
2010
20
40
60
80
1985
1995
2005

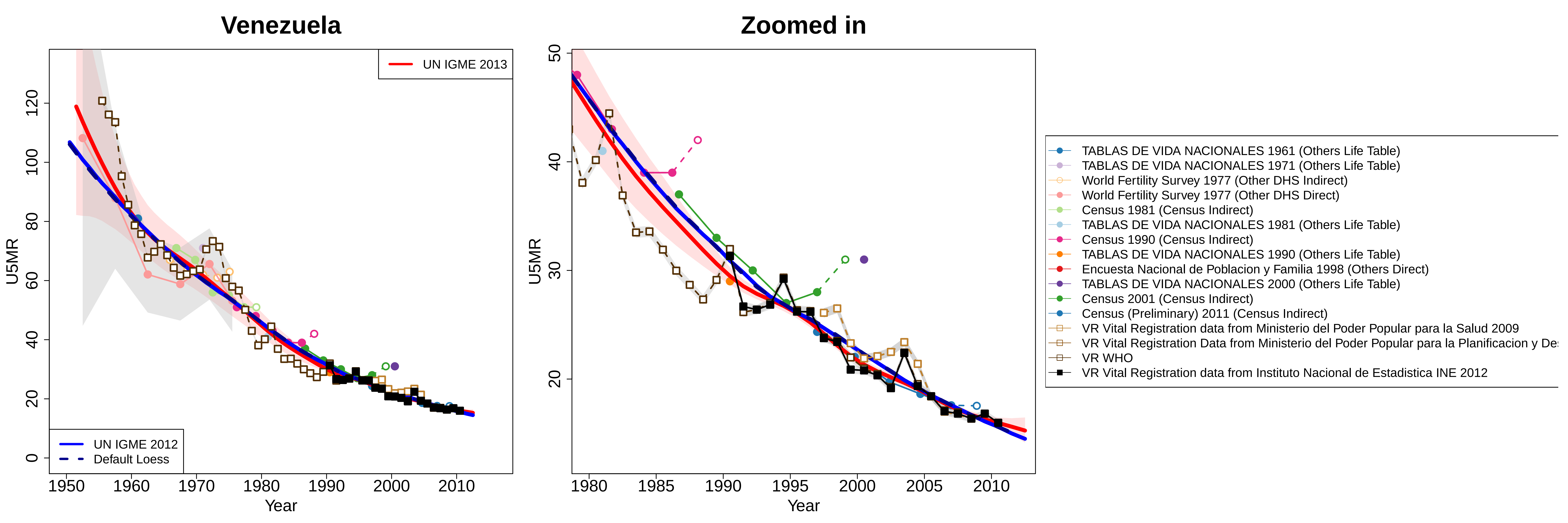
Venezuela
Zoomed in
UN IGME 2013
UN IGME 2012
Default Loess
U5MR
Year
0
20
40
60
80
100
120
1950
1960
1970
1980
1990
2000
2010
20
30
40
50
1980
1985
1990
1995
2000
2005
2010
TABLAS DE VIDA NACIONALES 1961 (Others Life Table)
TABLAS DE VIDA NACIONALES 1971 (Others Life Table)
World Fertility Survey 1977 (Other DHS Indirect)
World Fertility Survey 1977 (Other DHS Direct)
Census 1981 (Census Indirect)
TABLAS DE VIDA NACIONALES 1981 (Others Life Table)
Census 1990 (Census Indirect)
TABLAS DE VIDA NACIONALES 1990 (Others Life Table)
Encuesta Nacional de Poblacion y Familia 1998 (Others Direct)
TABLAS DE VIDA NACIONALES 2000 (Others Life Table)
Census 2001 (Census Indirect)
Census (Preliminary) 2011 (Census Indirect)
VR Vital Registration data from Ministerio del Poder Popular para la Salud 2009
VR Vital Registration Data from Ministerio del Poder Popular para la Planificacion y De
VR WHO
VR Vital Registration data from Instituto Nacional de Estadistica INE 2012

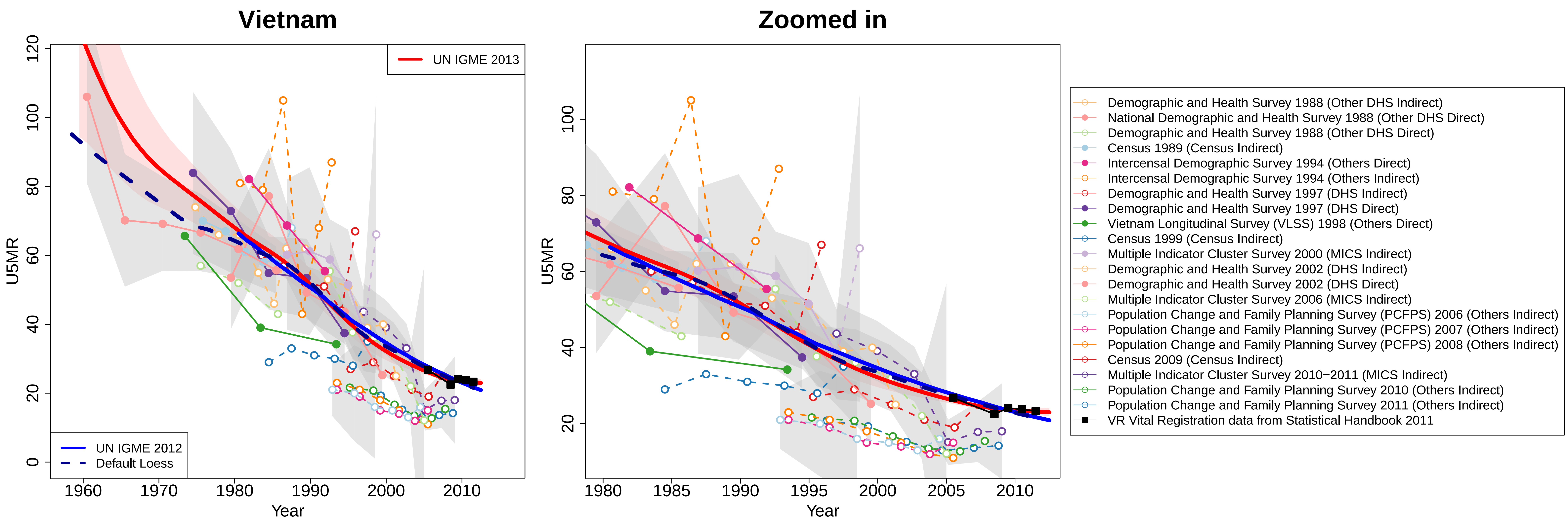

Vietnam
Zoomed in
U5MR
Year
UN IGME 2013
UN IGME 2012
Default Loess
Demographic and Health Survey 1988 (Other DHS Indirect)
National Demographic and Health Survey 1988 (Other DHS Direct)
Demographic and Health Survey 1988 (Other DHS Direct)
Census 1989 (Census Indirect)
Intercensal Demographic Survey 1994 (Others Direct)
Intercensal Demographic Survey 1994 (Others Indirect)
Demographic and Health Survey 1997 (DHS Indirect)
Demographic and Health Survey 1997 (DHS Direct)
Vietnam Longitudinal Survey (VLSS) 1998 (Others Direct)
Census 1999 (Census Indirect)
Multiple Indicator Cluster Survey 2000 (MICS Indirect)
Demographic and Health Survey 2002 (DHS Indirect)
Demographic and Health Survey 2002 (DHS Direct)
Multiple Indicator Cluster Survey 2006 (MICS Indirect)
Population Change and Family Planning Survey (PCFPS) 2006 (Others Indirect)
Population Change and Family Planning Survey (PCFPS) 2007 (Others Indirect)
Population Change and Family Planning Survey (PCFPS) 2008 (Others Indirect)
Census 2009 (Census Indirect)
Multiple Indicator Cluster Survey 2010−2011 (MICS Indirect)
Population Change and Family Planning Survey 2010 (Others Indirect)
Population Change and Family Planning Survey 2011 (Others Indirect)
VR Vital Registration data from Statistical Handbook 2011

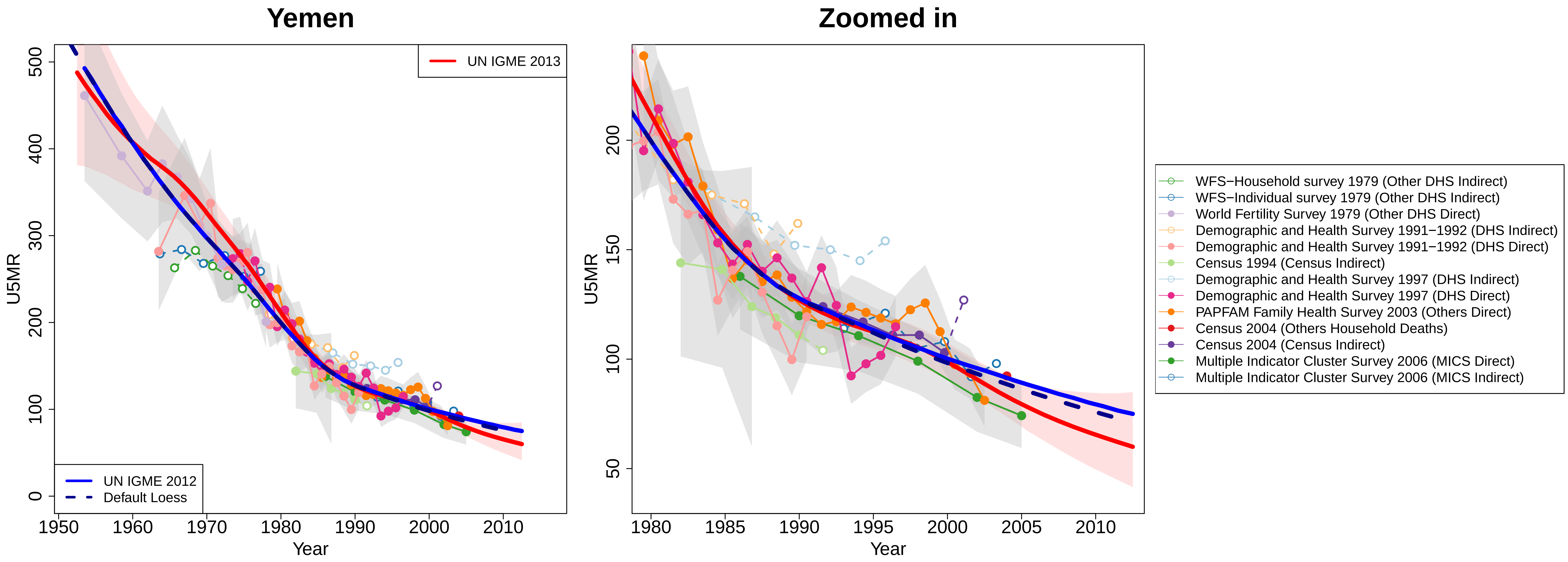
Yemen
Zoomed in
U5MR
Year
UN IGME 2013
UN IGME 2012
Default Loess
WFS−Household survey 1979 (Other DHS Indirect)
WFS−Individual survey 1979 (Other DHS Indirect)
World Fertility Survey 1979 (Other DHS Direct)
Demographic and Health Survey 1991−1992 (DHS Indirect)
Demographic and Health Survey 1991−1992 (DHS Direct)
Census 1994 (Census Indirect)
Demographic and Health Survey 1997 (DHS Indirect)
Demographic and Health Survey 1997 (DHS Direct)
PAPFAM Family Health Survey 2003 (Others Direct)
Census 2004 (Others Household Deaths)
Census 2004 (Census Indirect)
Multiple Indicator Cluster Survey 2006 (MICS Direct)
Multiple Indicator Cluster Survey 2006 (MICS Indirect)

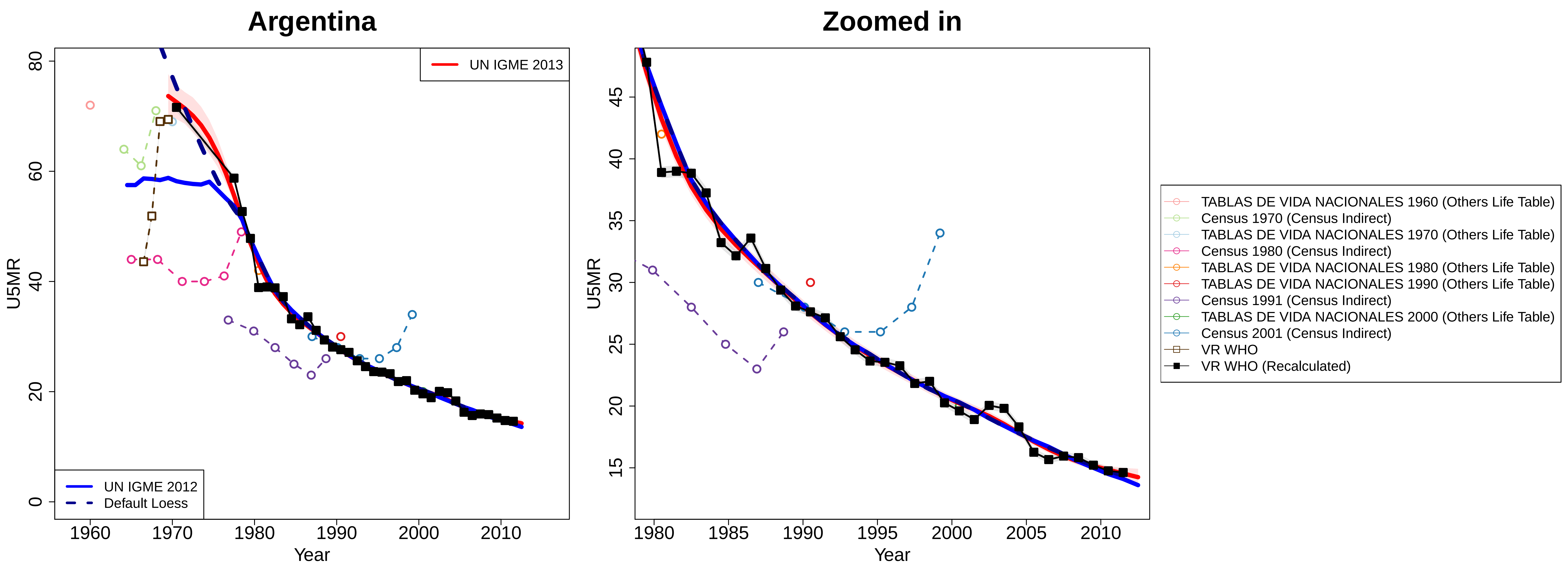

Argentina
Zoomed in
UN IGME 2013
UN IGME 2012
Default Loess
U5MR
Year
0
20
40
60
80
1960
1970
1980
1990
2000
2010
15
20
25
30
35
40
45
1985
1995
2005
TABLAS DE VIDA NACIONALES 1960 (Others Life Table)
Census 1970 (Census Indirect)
TABLAS DE VIDA NACIONALES 1970 (Others Life Table)
Census 1980 (Census Indirect)
TABLAS DE VIDA NACIONALES 1980 (Others Life Table)
TABLAS DE VIDA NACIONALES 1990 (Others Life Table)
Census 1991 (Census Indirect)
TABLAS DE VIDA NACIONALES 2000 (Others Life Table)
Census 2001 (Census Indirect)
VR WHO
VR WHO (Recalculated)

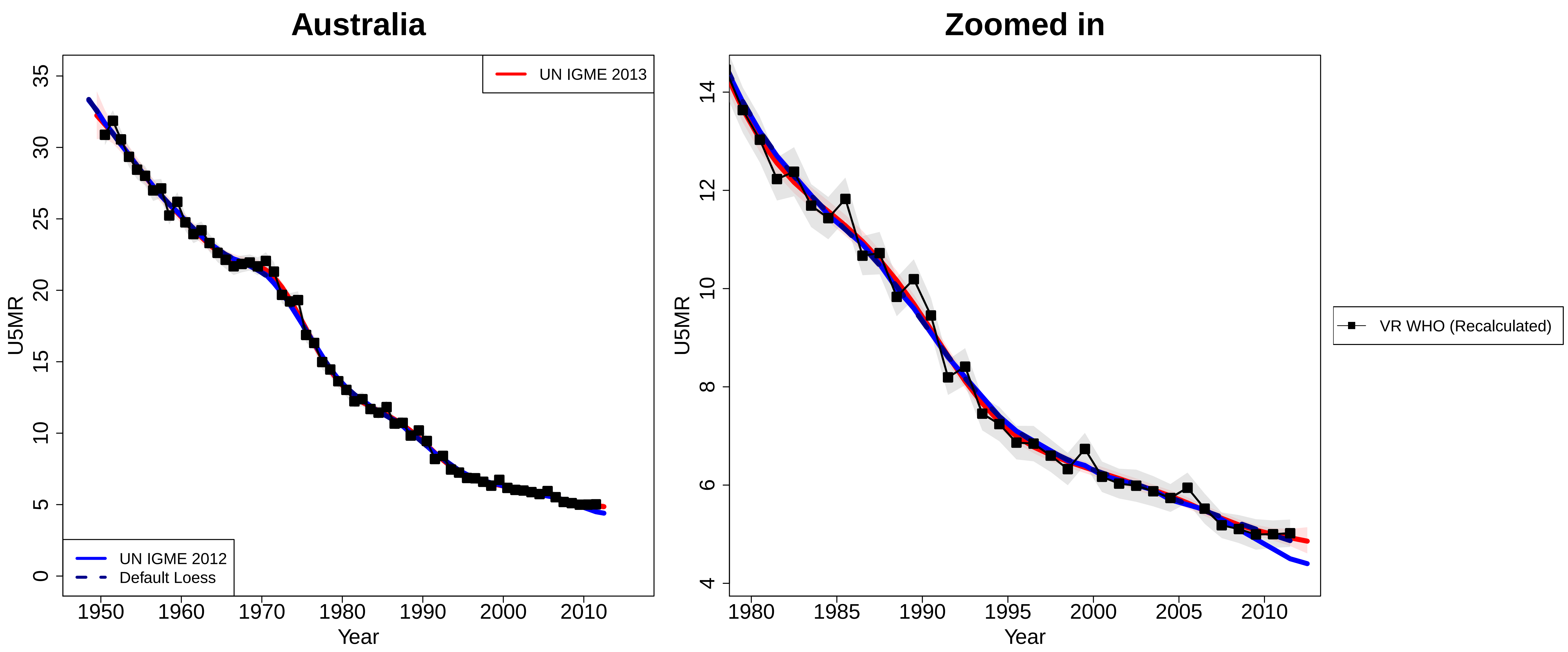

Australia
Zoomed in
UN IGME 2013
UN IGME 2012
Default Loess
VR WHO (Recalculated)
U5MR
Year
0
5
10
15
20
25
30
35
1950
1960
1970
1980
1990
2000
2010
4
6
8
10
12
14
1980
1985
1990
1995
2000
2005
2010

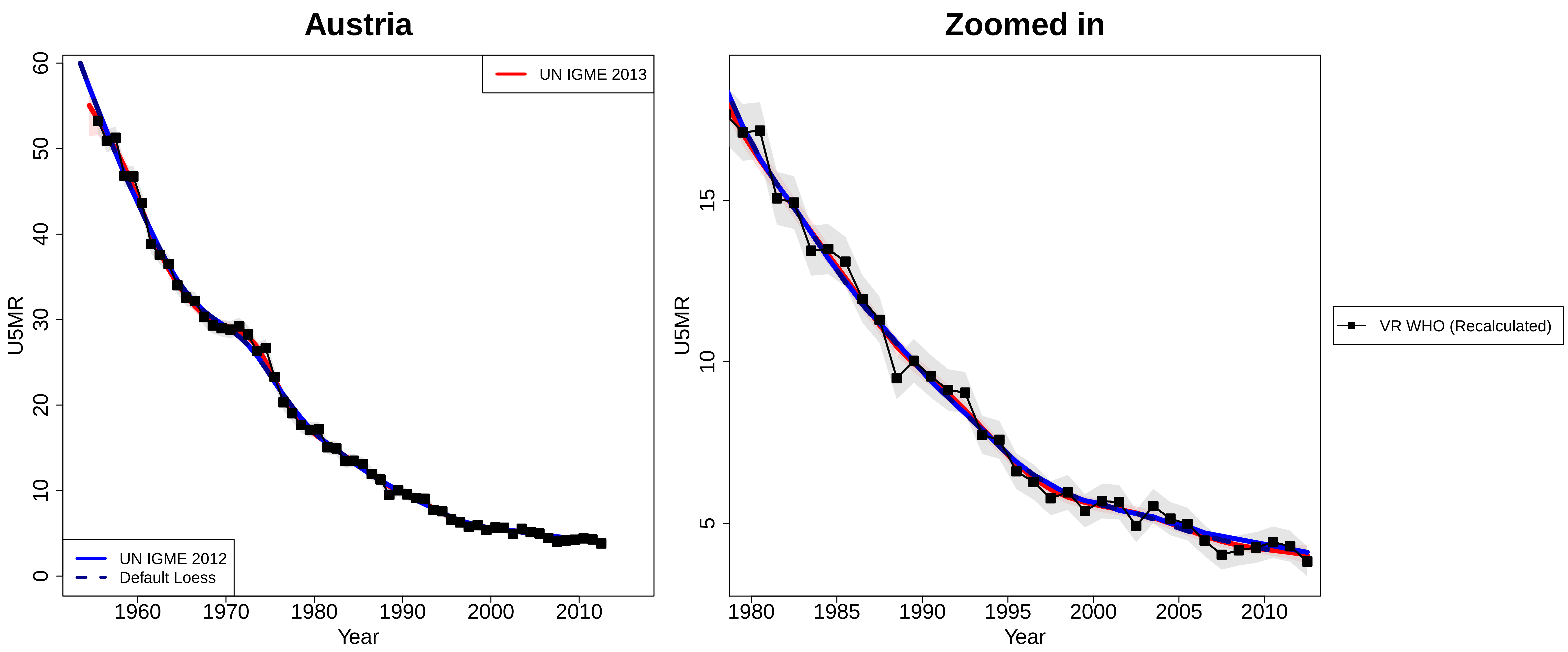

Austria
UN IGME 2013
UN IGME 2012
Default Loess
U5MR
Year
Zoomed in
VR WHO (Recalculated)

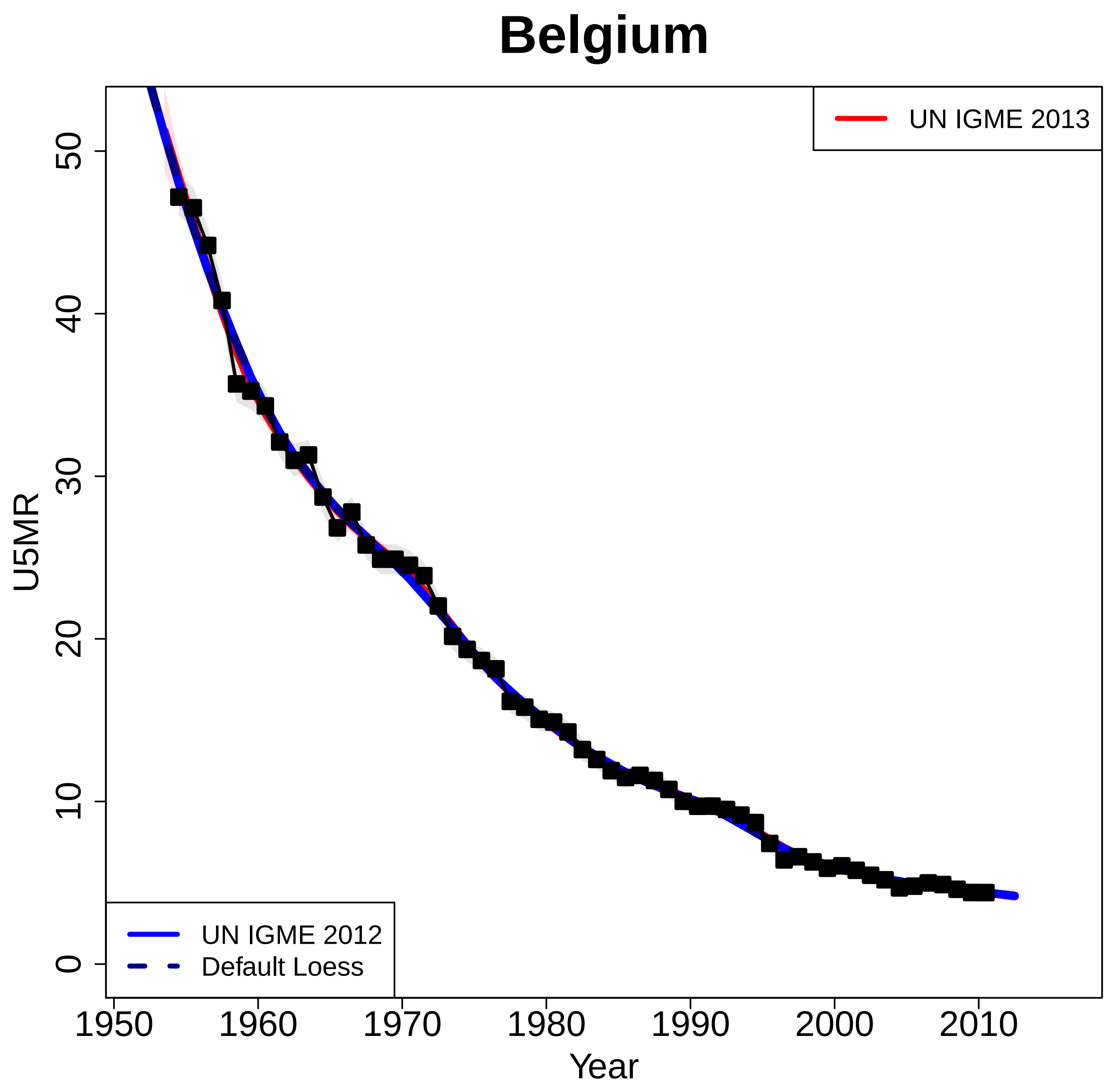

Belgium
UN IGME 2013
UN IGME 2012
Default Loess
U5MR
Year
0
10
20
30
40
50
1950
1960
1970
1980
1990
2000
2010

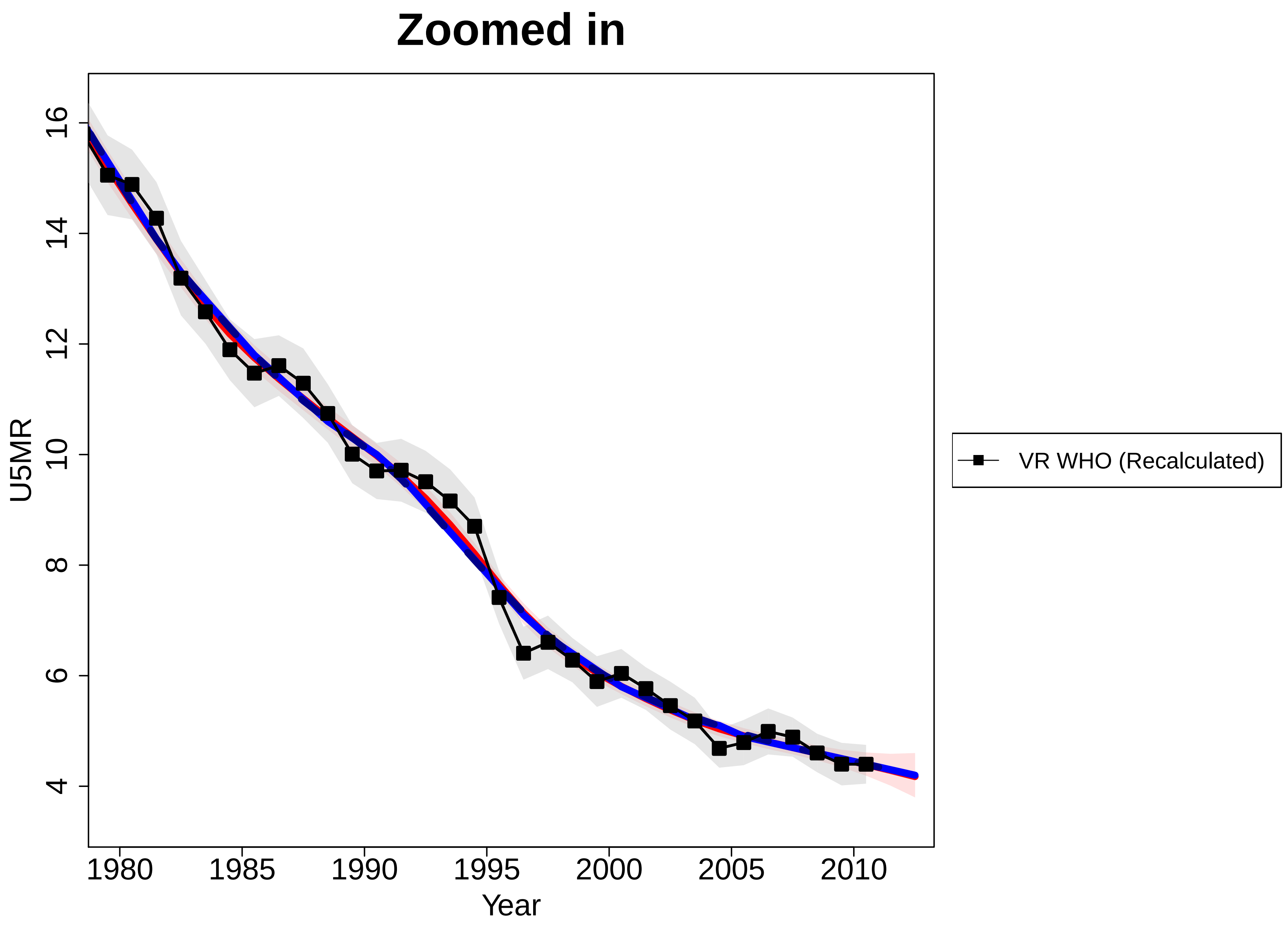

Zoomed in
VR WHO (Recalculated)
U5MR
Year
4
6
8
10
12
14
16
1980
1985
1990
1995
2000
2005
2010

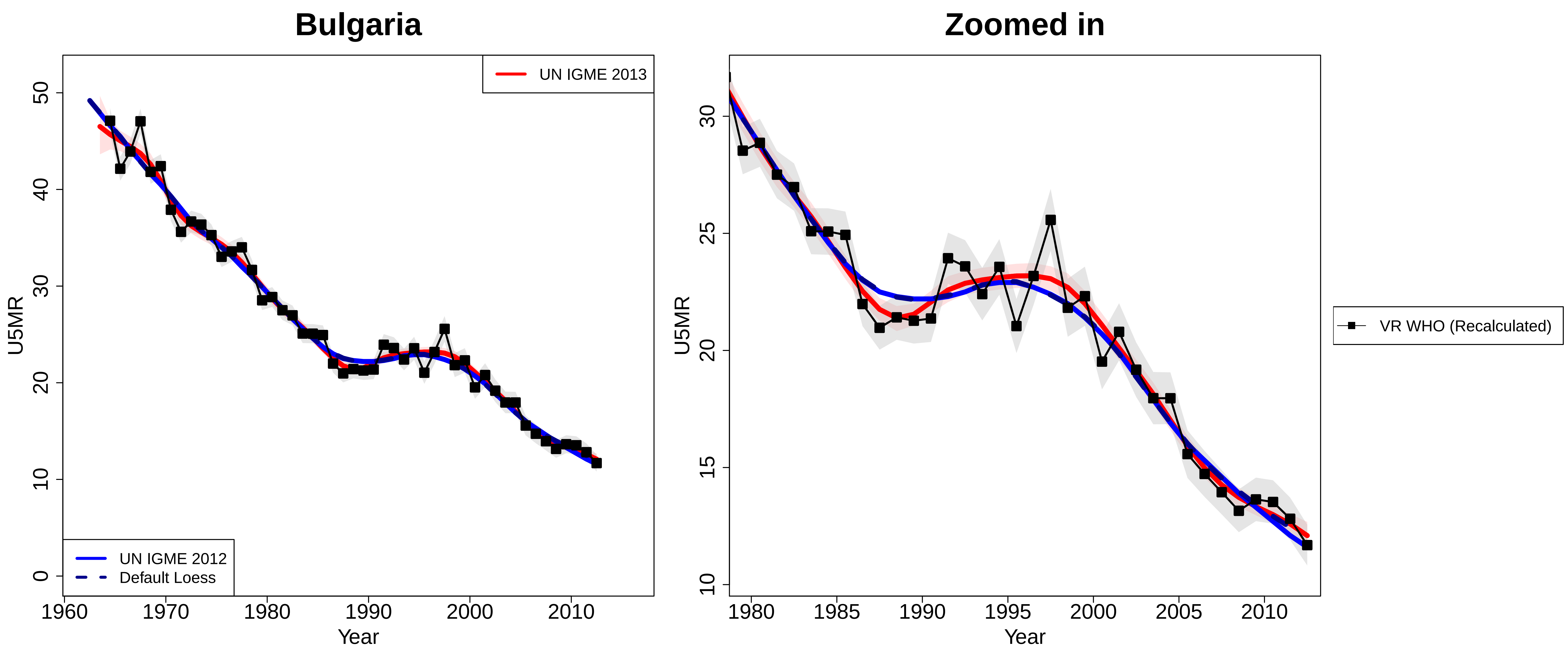
Bulgaria
Zoomed in
UN IGME 2013
UN IGME 2012
Default Loess
VR WHO (Recalculated)
U5MR
Year
0
10
20
30
40
50
1960
1970
1980
1990
2000
2010
15
25
1985
1995
2005

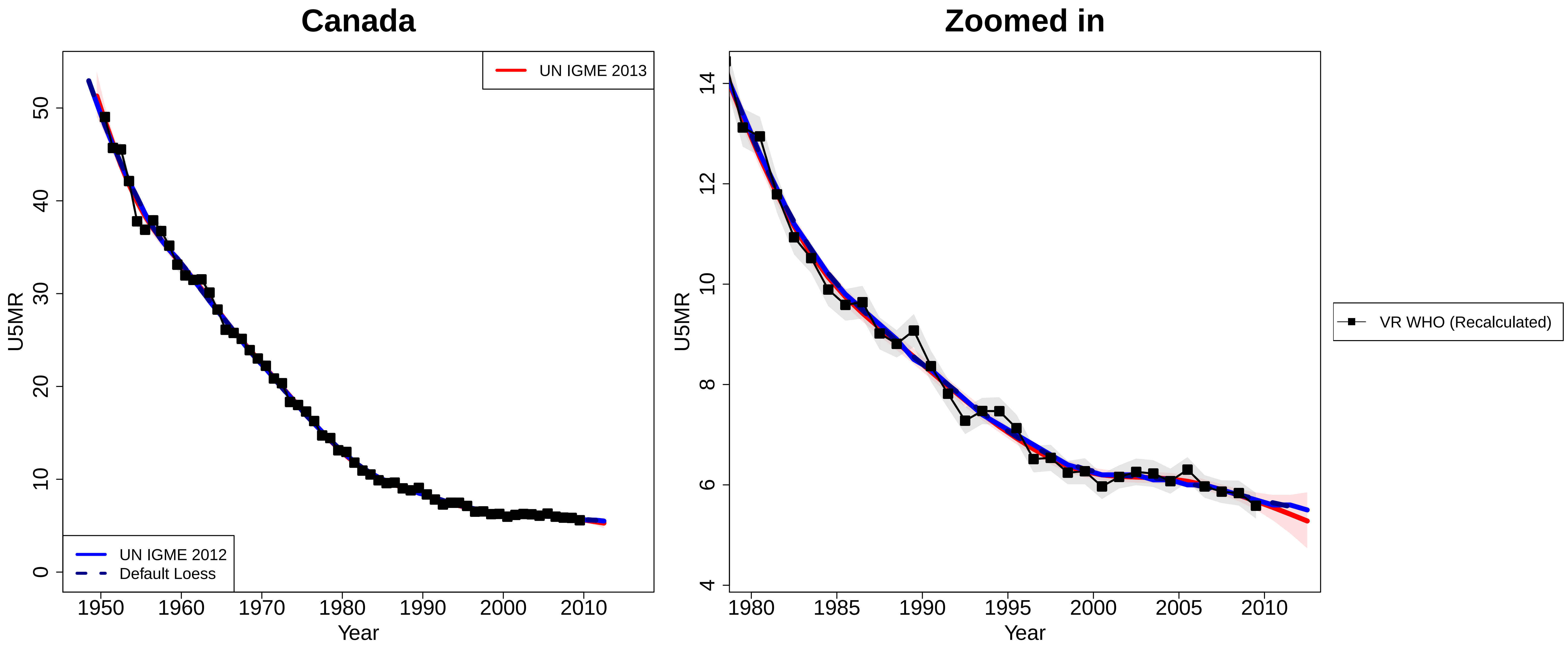
Canada
UN IGME 2013
UN IGME 2012
Default Loess
U5MR
Year
0
10
20
30
40
50
1950
1960
1970
1980
1990
2000
2010
Zoomed in
U5MR
Year
4
6
8
10
12
14
1980
1985
1990
1995
2000
2005
2010
VR WHO (Recalculated)

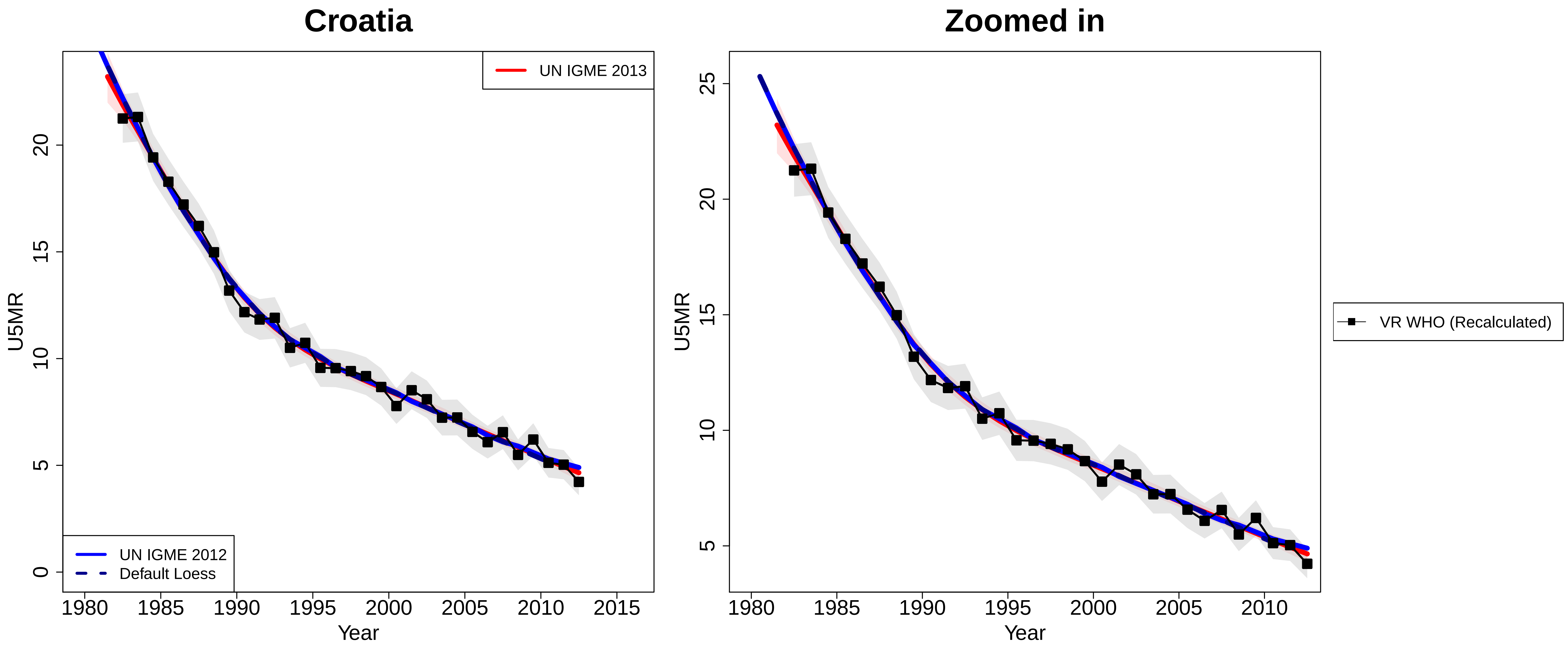

Croatia
Zoomed in
UN IGME 2013
UN IGME 2012
Default Loess
VR WHO (Recalculated)
U5MR
Year
0
5
10
15
20
25
1980
1985
1990
1995
2000
2005
2010
2015

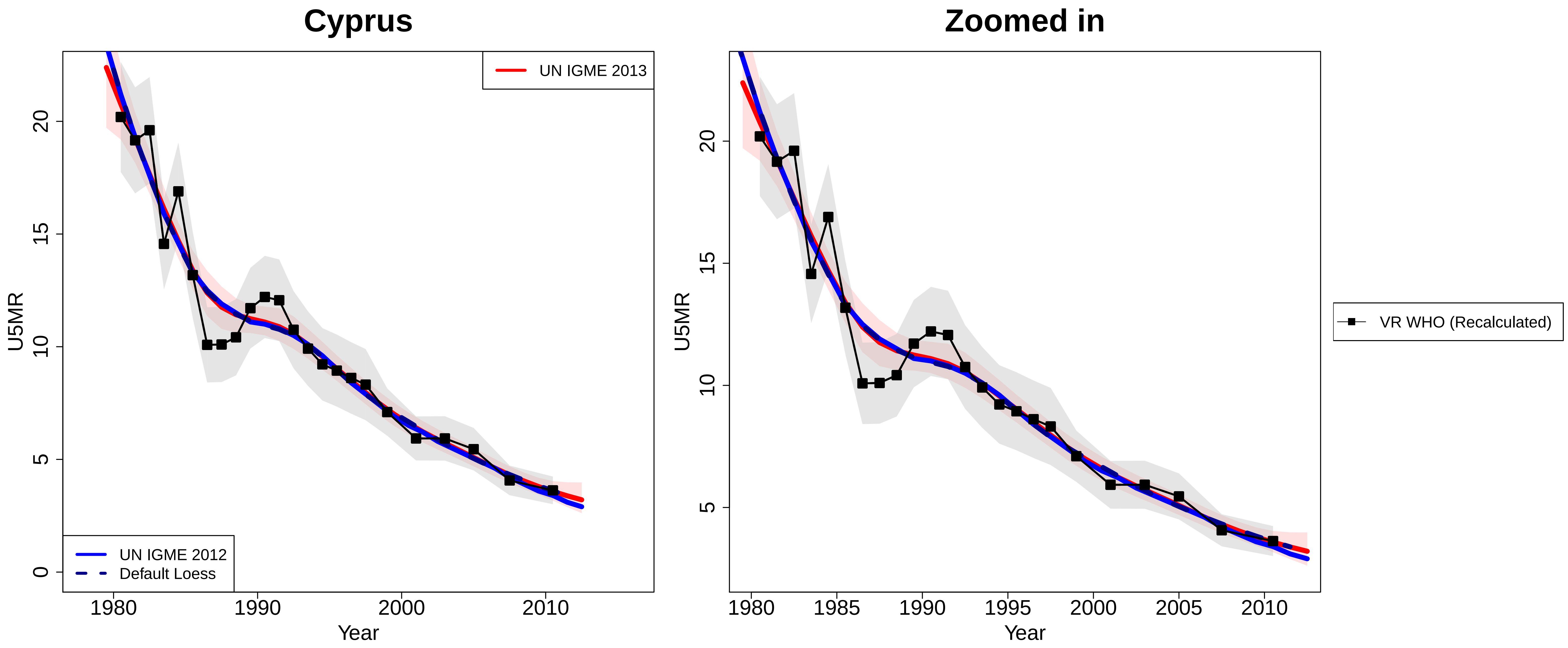

Cyprus
Zoomed in
UN IGME 2013
UN IGME 2012
Default Loess
VR WHO (Recalculated)
U5MR
Year
0
5
10
15
20
1980
1985
1990
1995
2000
2005
2010

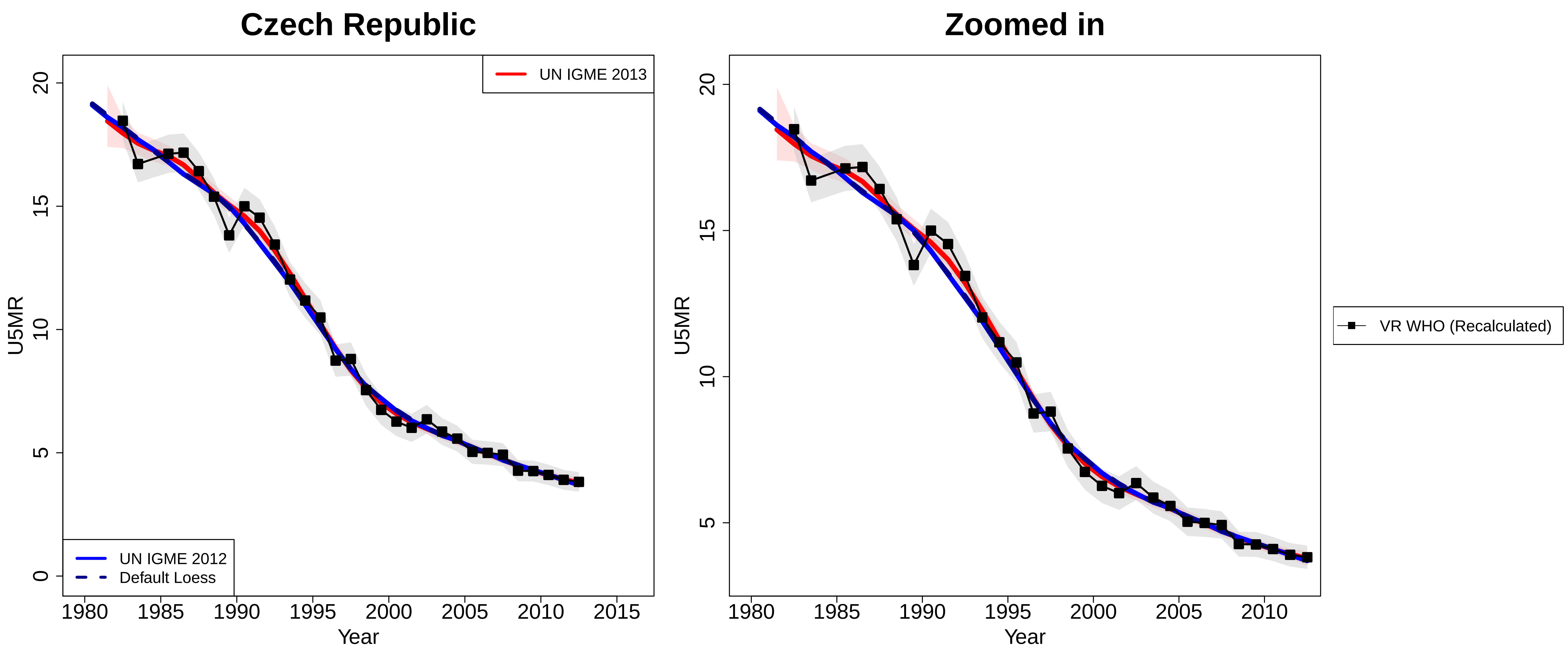
Czech Republic
Zoomed in
UN IGME 2013
UN IGME 2012
Default Loess
VR WHO (Recalculated)
U5MR
Year
0
5
10
15
20
1980
1985
1990
1995
2000
2005
2010
2015

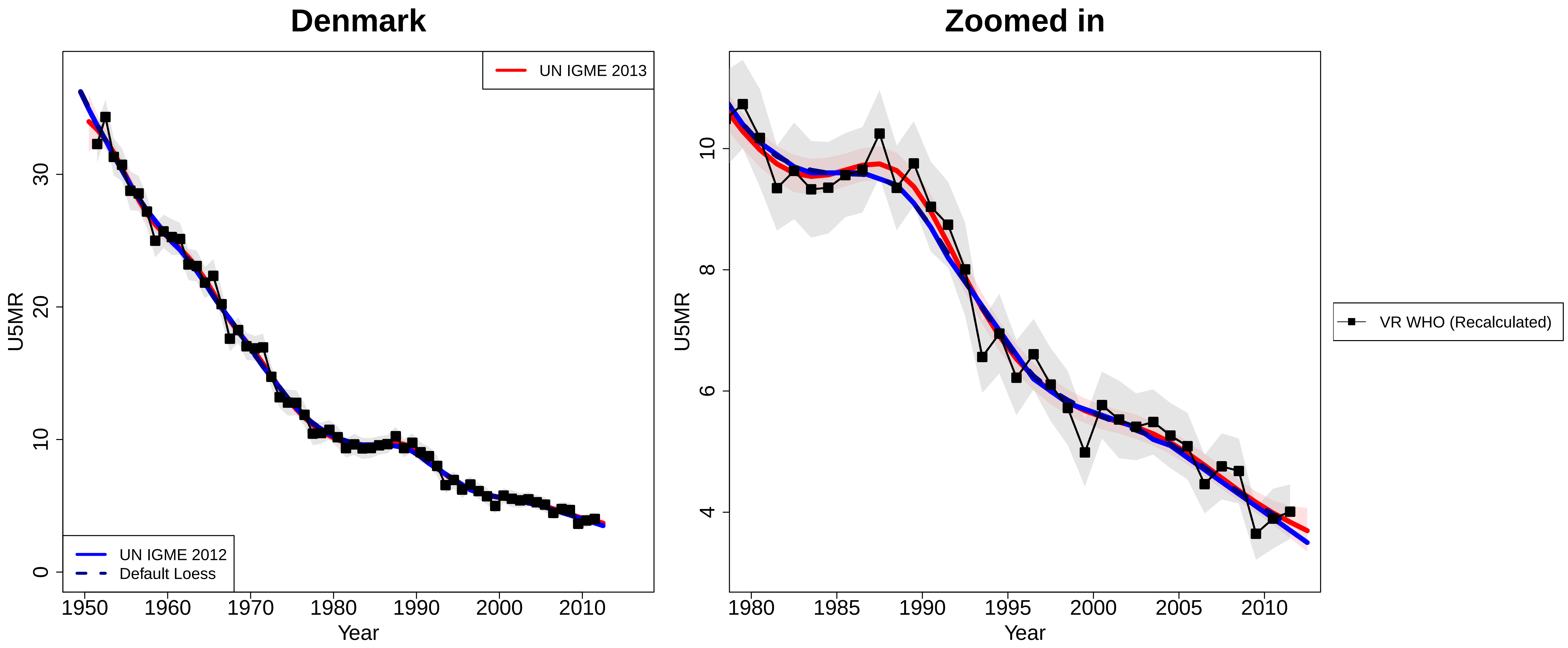

Denmark
Zoomed in
UN IGME 2013
UN IGME 2012
Default Loess
VR WHO (Recalculated)
U5MR
Year
0
10
20
30
4
6
8
10
1950
1960
1970
1980
1990
2000
2010
1985
1995
2005

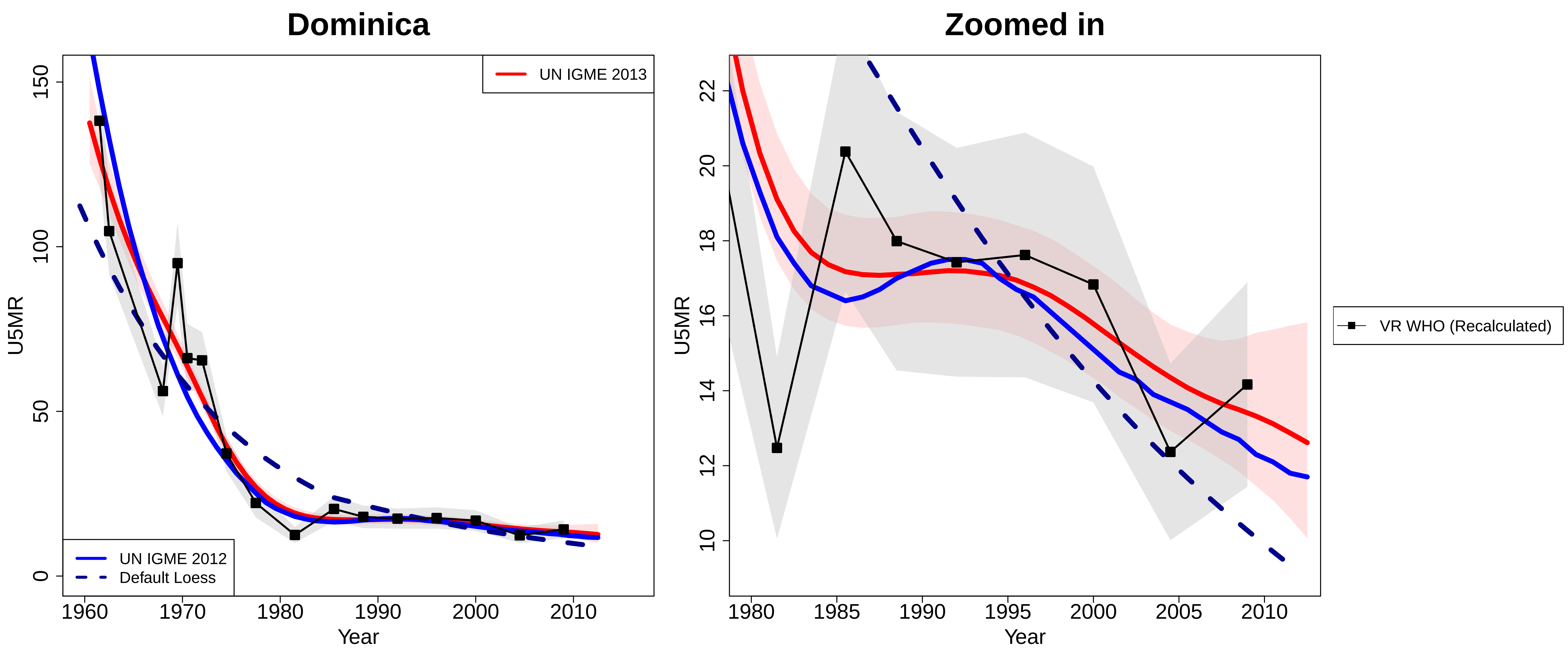
Dominica
Zoomed in
UN IGME 2013
UN IGME 2012
Default Loess
VR WHO (Recalculated)
U5MR
Year
0
50
100
150
10
12
14
16
18
20
22
1960
1970
1980
1990
2000
2010
1985
1995
2005

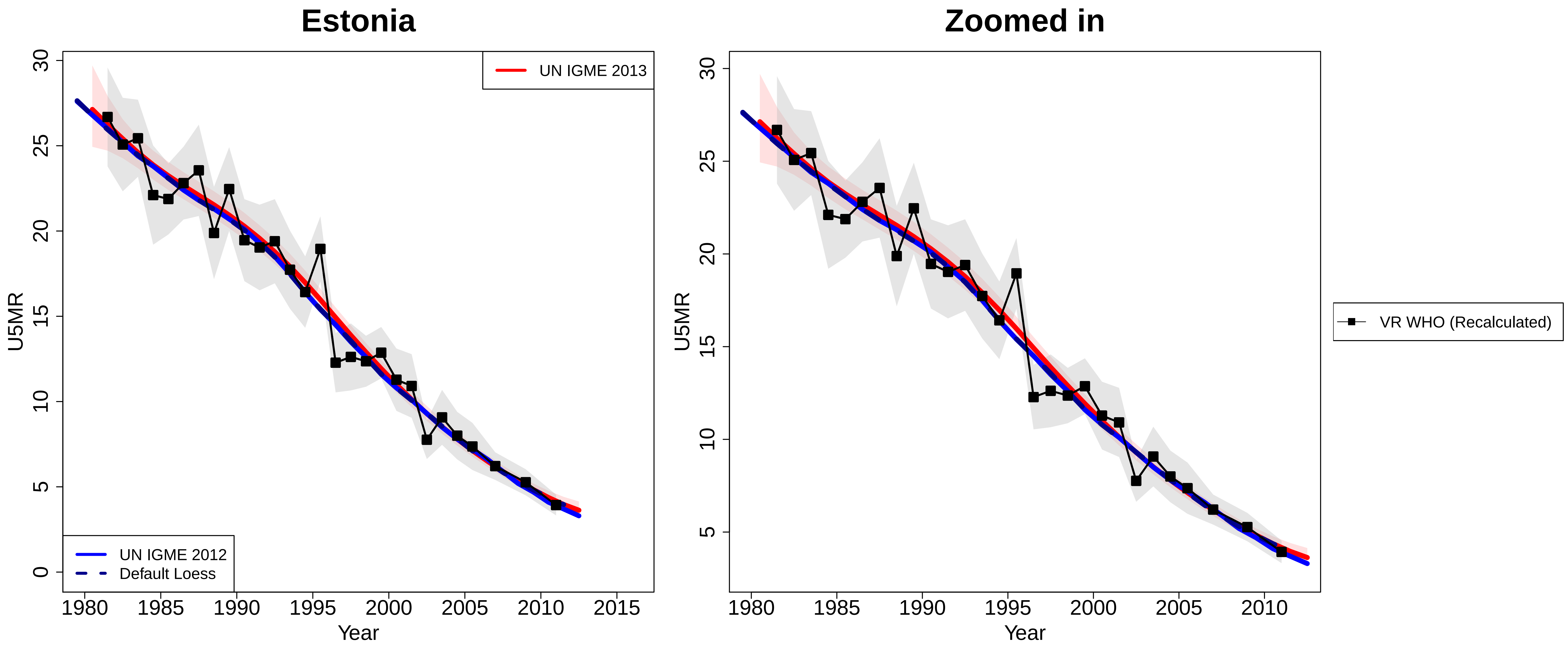

Estonia
Zoomed in
U5MR
Year
UN IGME 2013
UN IGME 2012
Default Loess
VR WHO (Recalculated)

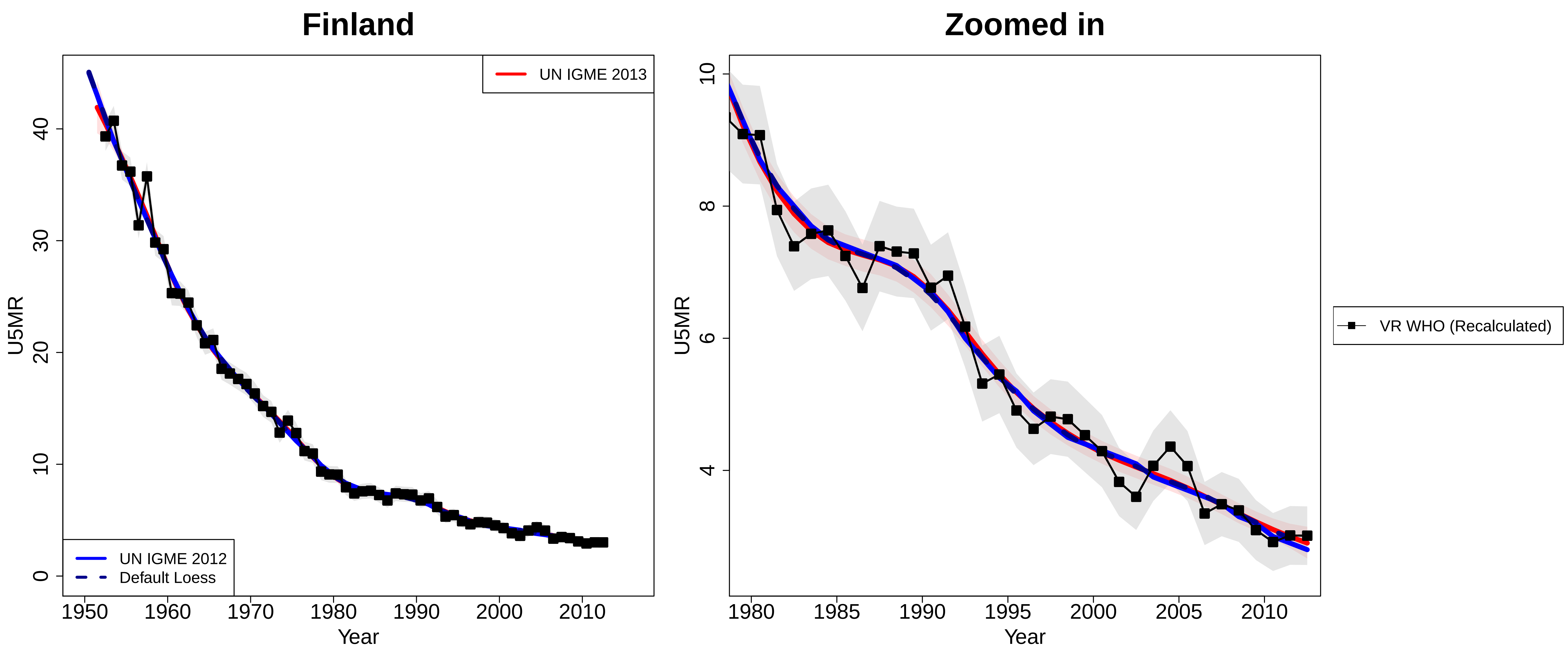

Finland
Zoomed in
UN IGME 2013
UN IGME 2012
Default Loess
VR WHO (Recalculated)
U5MR
Year
0
10
20
30
40
1950
1960
1970
1980
1990
2000
2010
4
6
8
10
1985
1995
2005

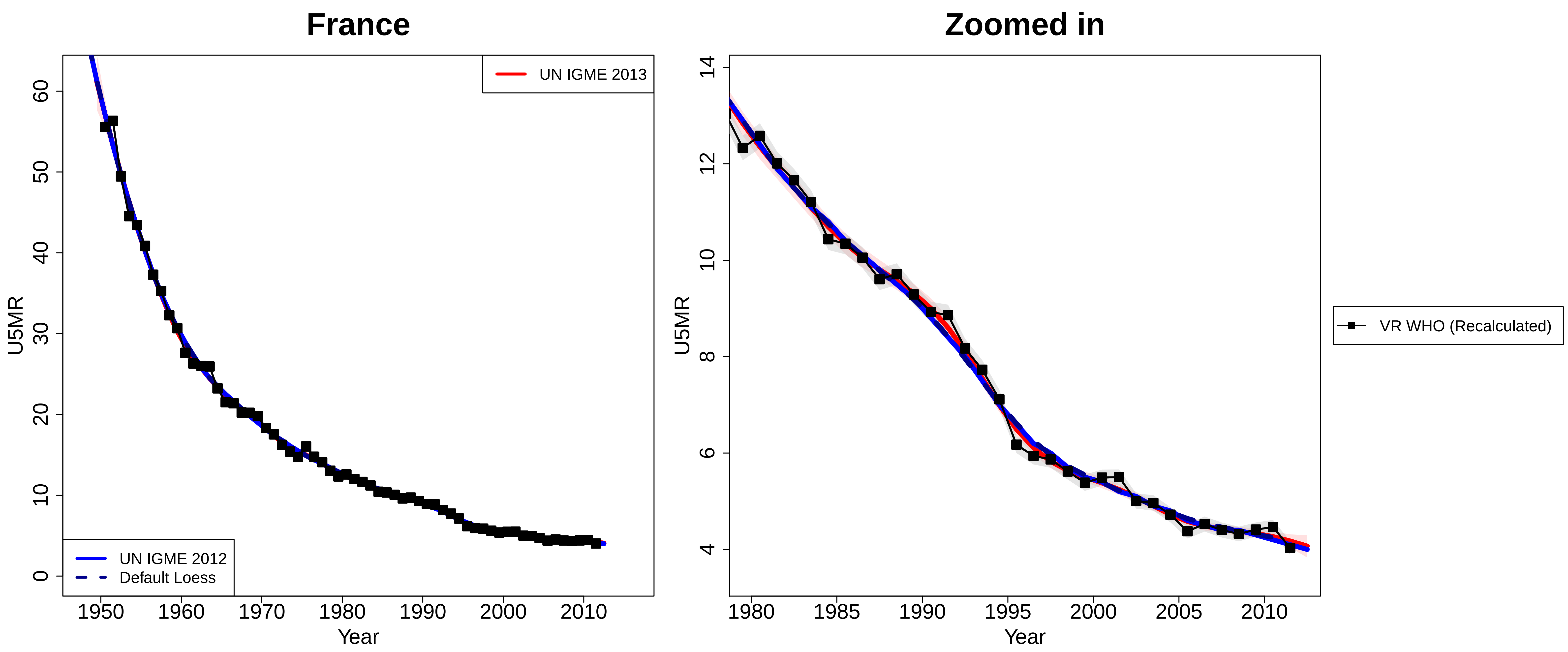
France
Zoomed in
UN IGME 2013
UN IGME 2012
Default Loess
VR WHO (Recalculated)
U5MR
Year
0
10
20
30
40
50
60
1950
1960
1970
1980
1990
2000
2010
4
6
8
10
12
14
1980
1985
1990
1995
2000
2005
2010

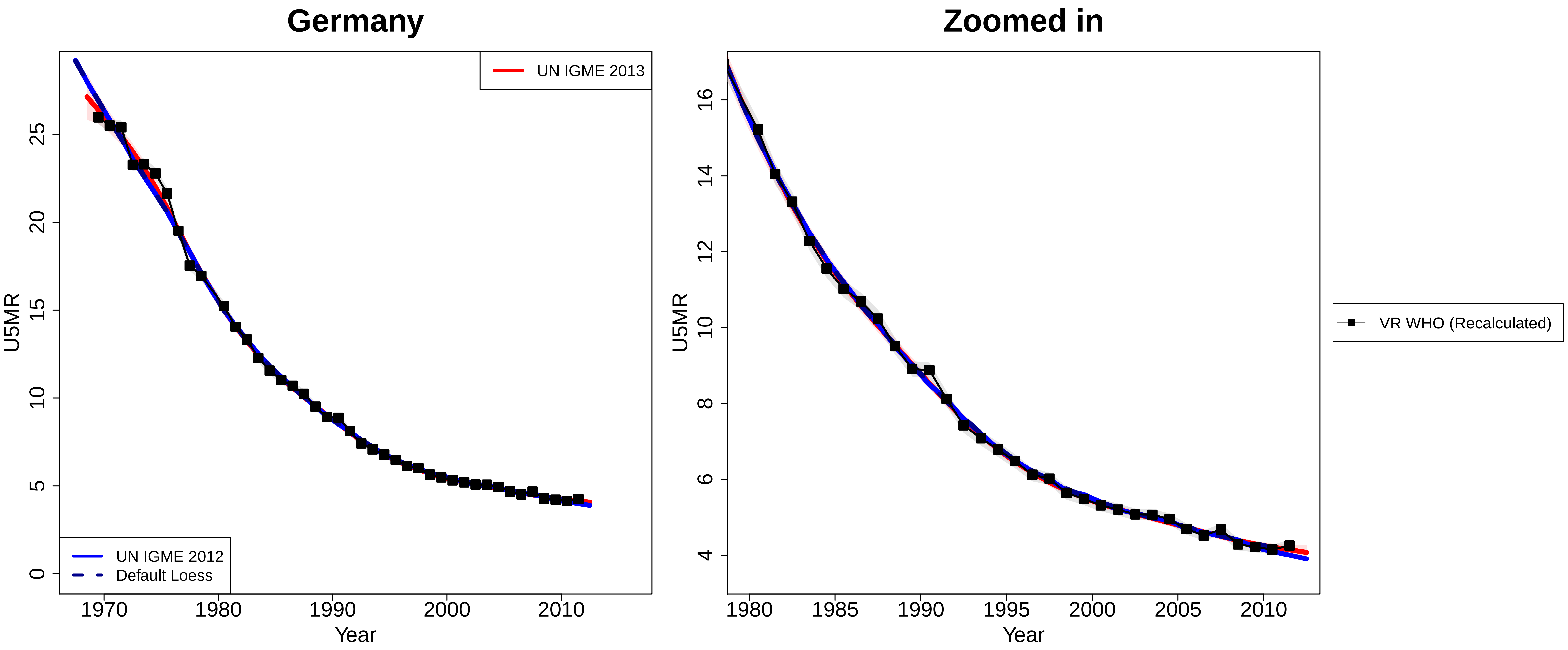
Germany
Zoomed in
UN IGME 2013
UN IGME 2012
Default Loess
VR WHO (Recalculated)
U5MR
Year
0
5
10
15
20
25
1970
1980
1990
2000
2010
4
6
8
10
12
14
16
1980
1985
1990
1995
2000
2005
2010

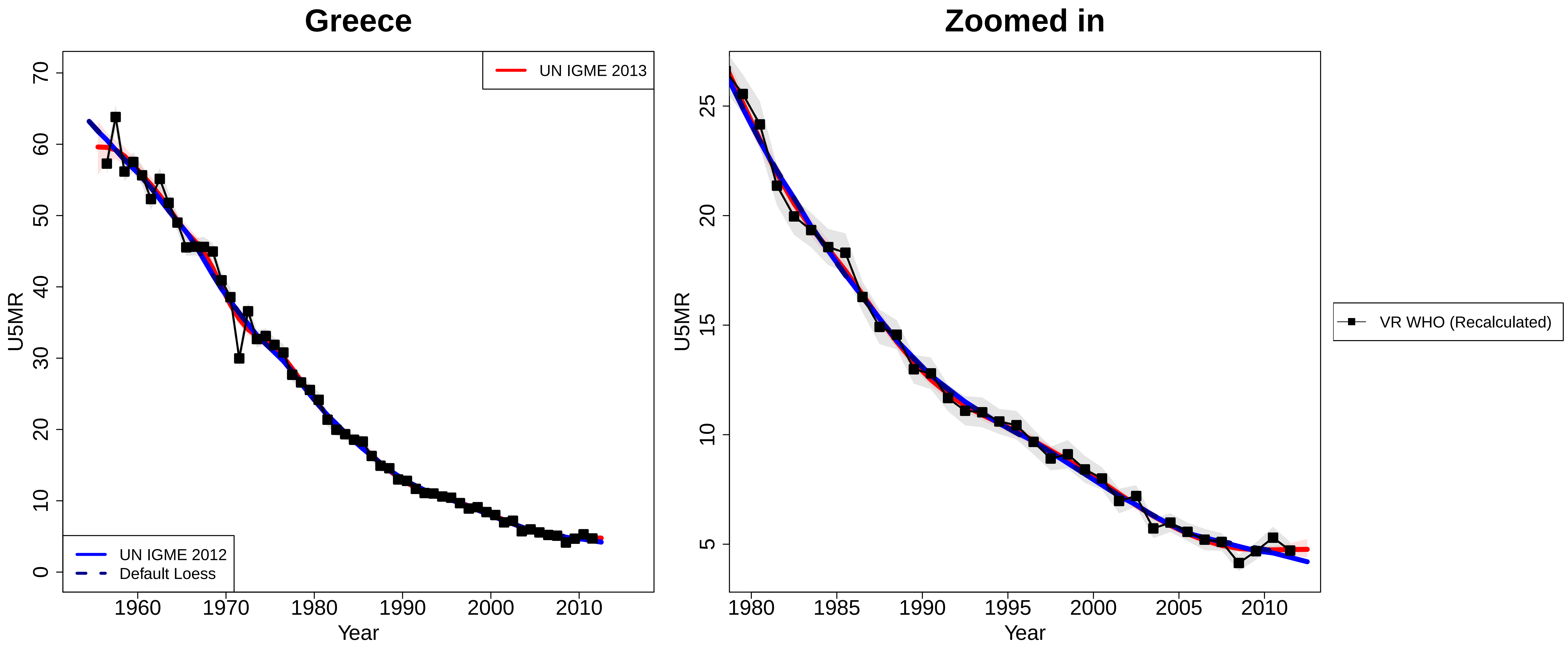
Greece
Zoomed in
UN IGME 2013
UN IGME 2012
Default Loess
VR WHO (Recalculated)
U5MR
Year
0
10
20
30
40
50
60
70
1960
1970
1980
1990
2000
2010
5
10
15
20
25
1980
1985
1990
1995
2000
2005
2010

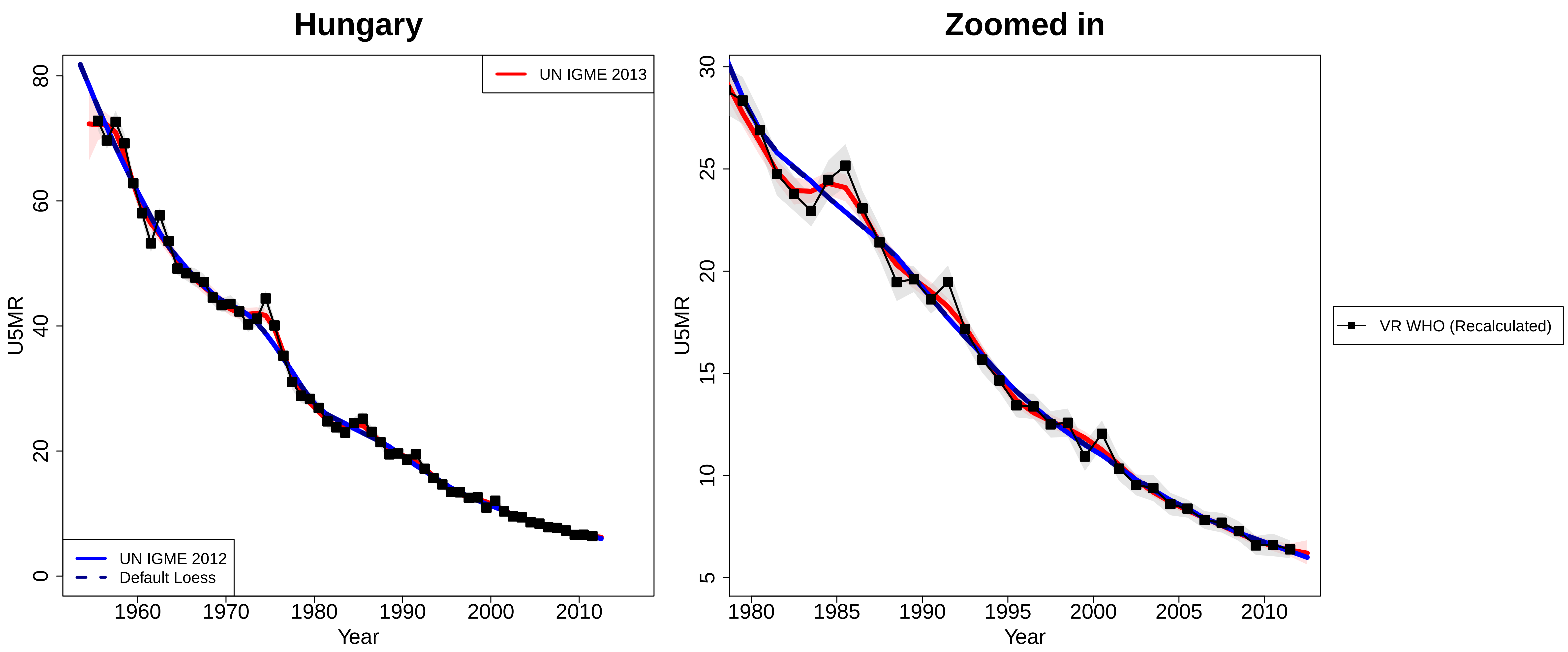

Hungary
UN IGME 2013
UN IGME 2012
Default Loess
U5MR
Year
Zoomed in
VR WHO (Recalculated)

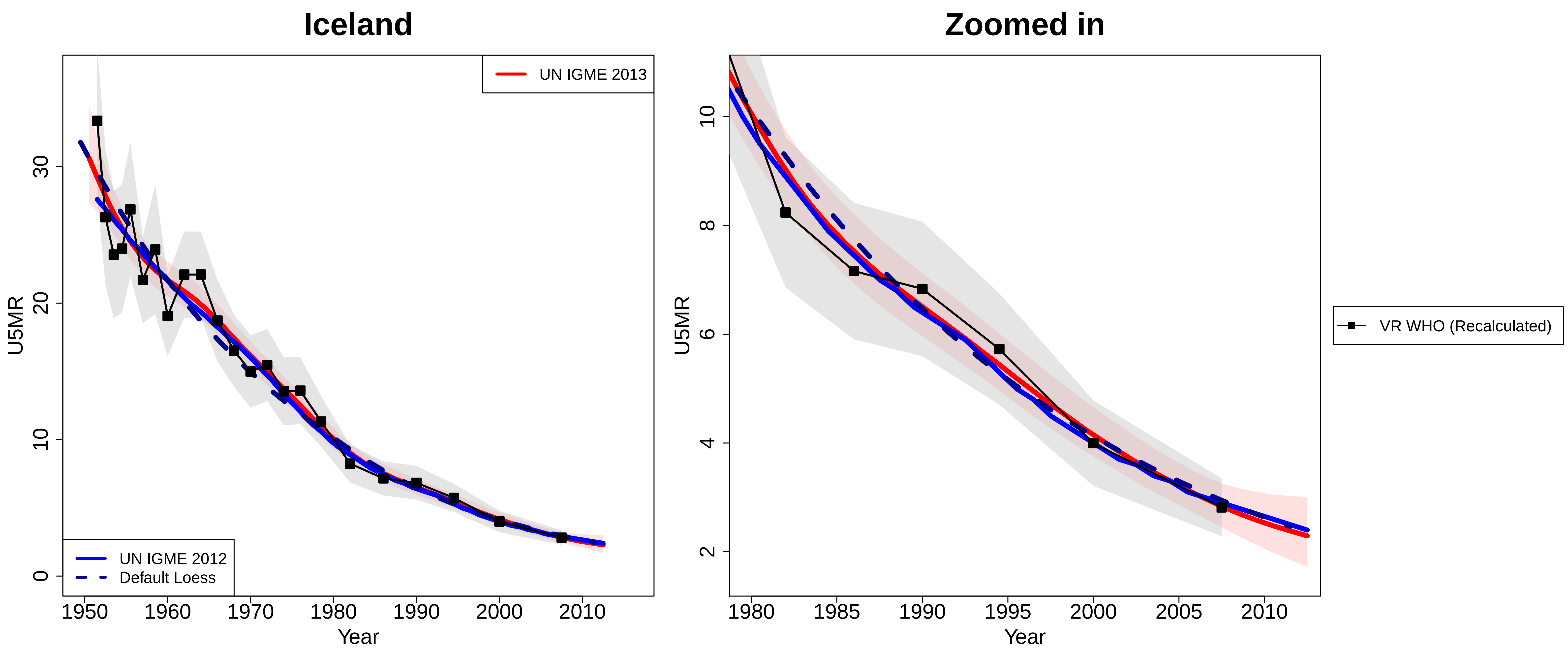
Iceland
Zoomed in
UN IGME 2013
UN IGME 2012
Default Loess
VR WHO (Recalculated)
U5MR
Year
0
10
20
30
1950
1960
1970
1980
1990
2000
2010
2
4
6
8
10
1985
1995
2005

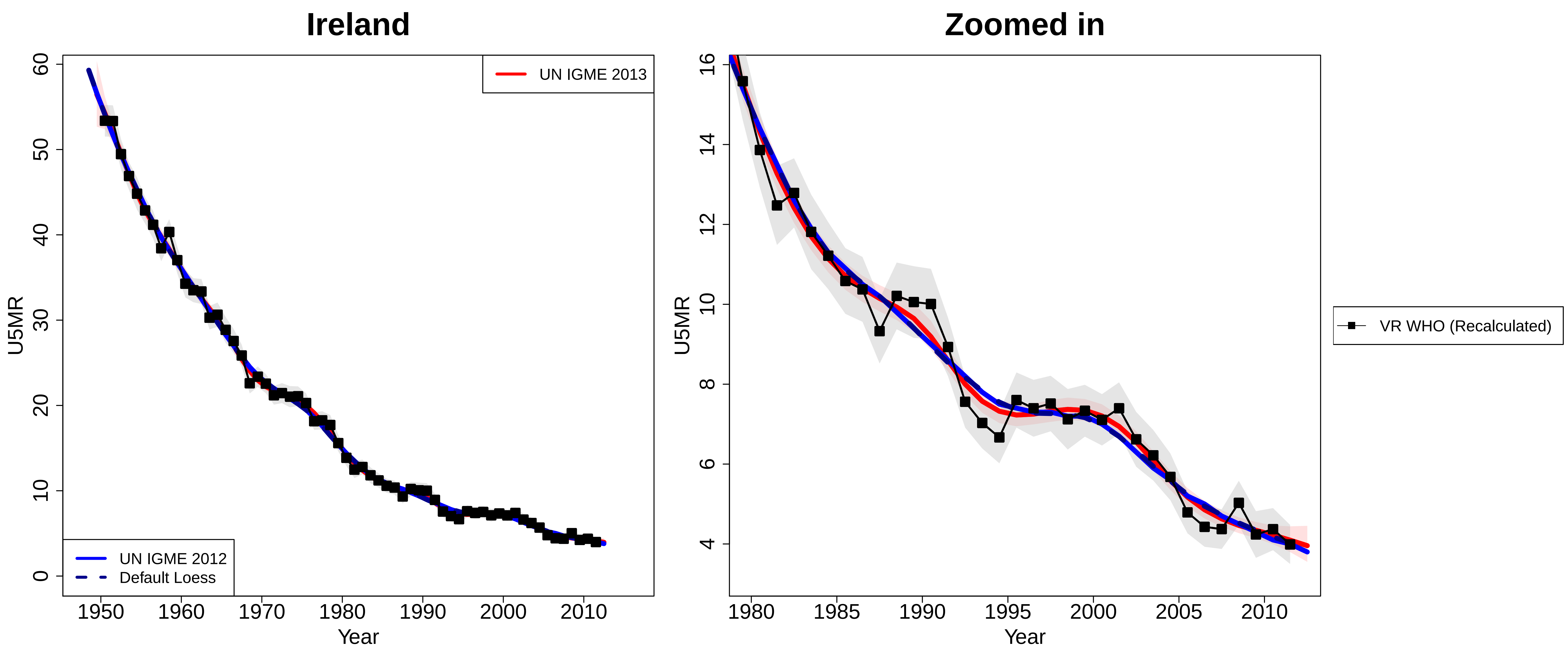
Ireland
Zoomed in
UN IGME 2013
UN IGME 2012
Default Loess
VR WHO (Recalculated)
U5MR
Year
0
10
20
30
40
50
60
1950
1960
1970
1980
1990
2000
2010
4
6
8
10
12
14
16
1985
1995
2005

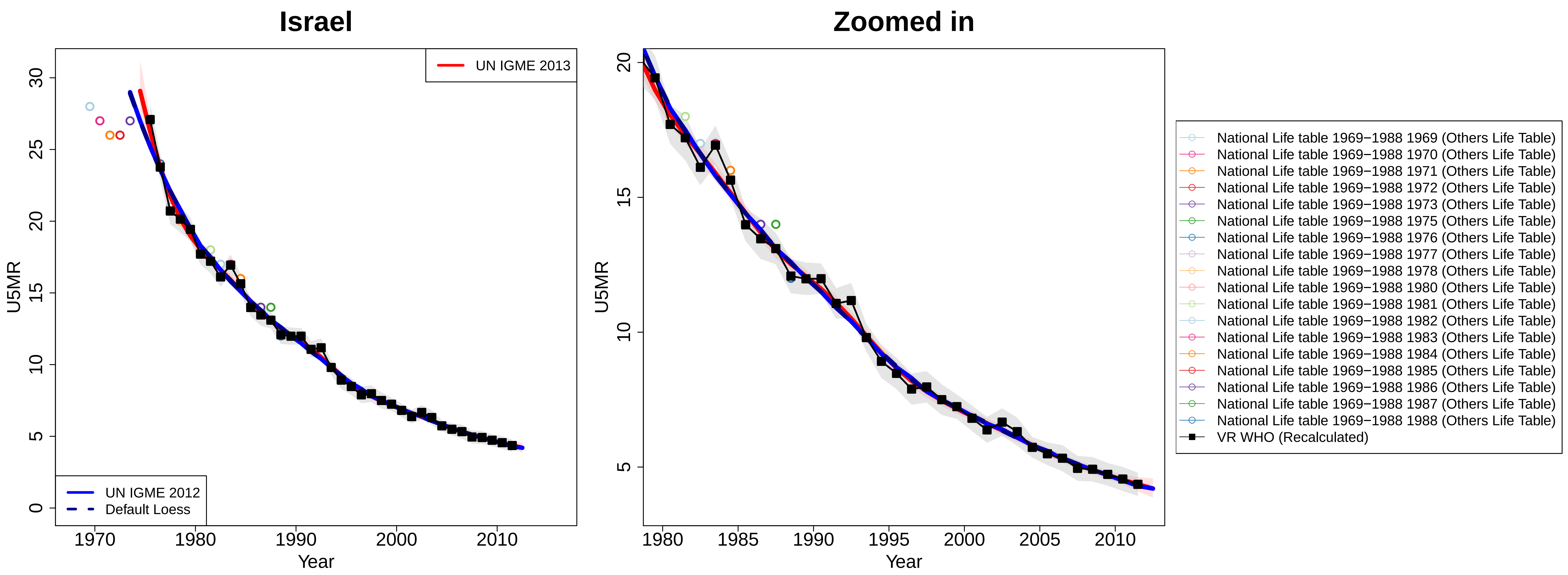

Israel
UN IGME 2013
UN IGME 2012
Default Loess
U5MR
Year
Zoomed in
National Life table 1969−1988 1969 (Others Life Table)
National Life table 1969−1988 1970 (Others Life Table)
National Life table 1969−1988 1971 (Others Life Table)
National Life table 1969−1988 1972 (Others Life Table)
National Life table 1969−1988 1973 (Others Life Table)
National Life table 1969−1988 1975 (Others Life Table)
National Life table 1969−1988 1976 (Others Life Table)
National Life table 1969−1988 1977 (Others Life Table)
National Life table 1969−1988 1978 (Others Life Table)
National Life table 1969−1988 1980 (Others Life Table)
National Life table 1969−1988 1981 (Others Life Table)
National Life table 1969−1988 1982 (Others Life Table)
National Life table 1969−1988 1983 (Others Life Table)
National Life table 1969−1988 1984 (Others Life Table)
National Life table 1969−1988 1985 (Others Life Table)
National Life table 1969−1988 1986 (Others Life Table)
National Life table 1969−1988 1987 (Others Life Table)
National Life table 1969−1988 1988 (Others Life Table)
VR WHO (Recalculated)

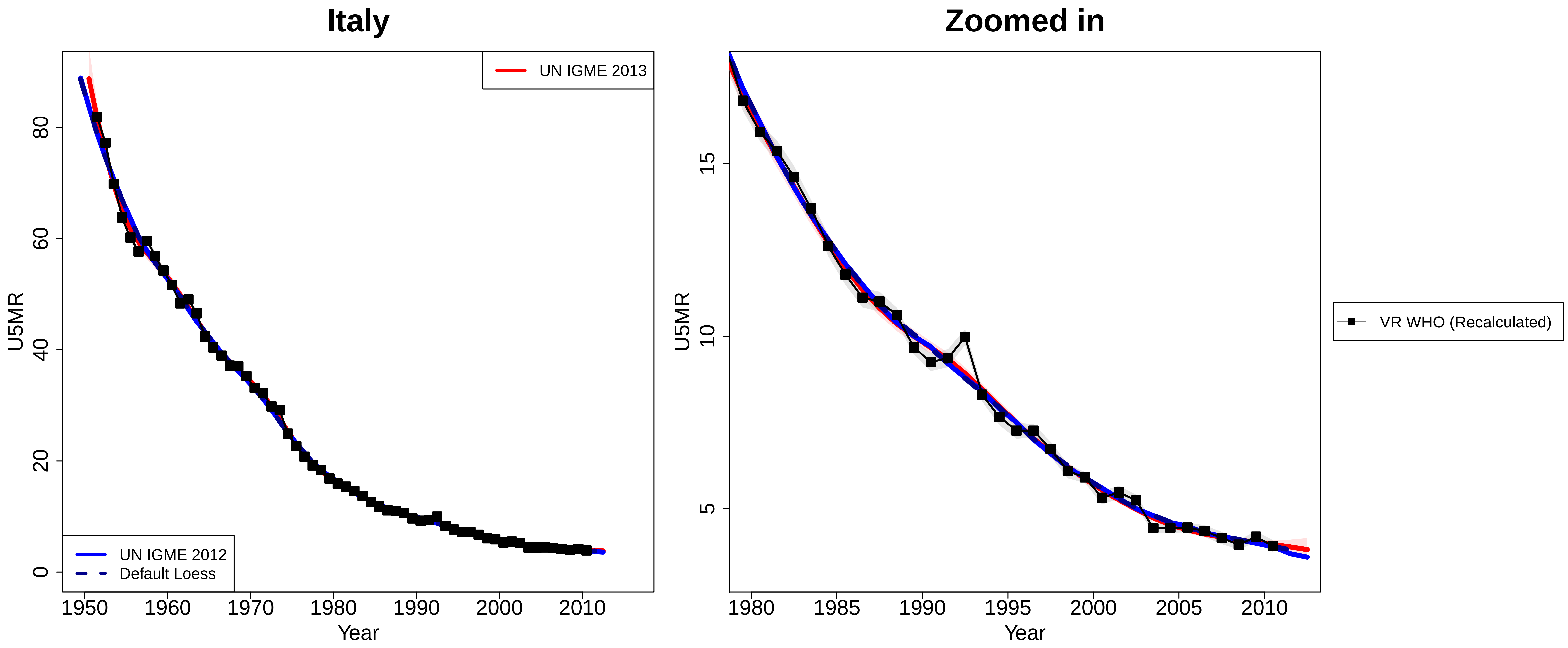

Italy
Zoomed in
UN IGME 2013
UN IGME 2012
Default Loess
VR WHO (Recalculated)
U5MR
Year
0
20
40
60
80
1950
1960
1970
1980
1990
2000
2010
5
10
15
1980
1985
1990
1995
2000
2005
2010

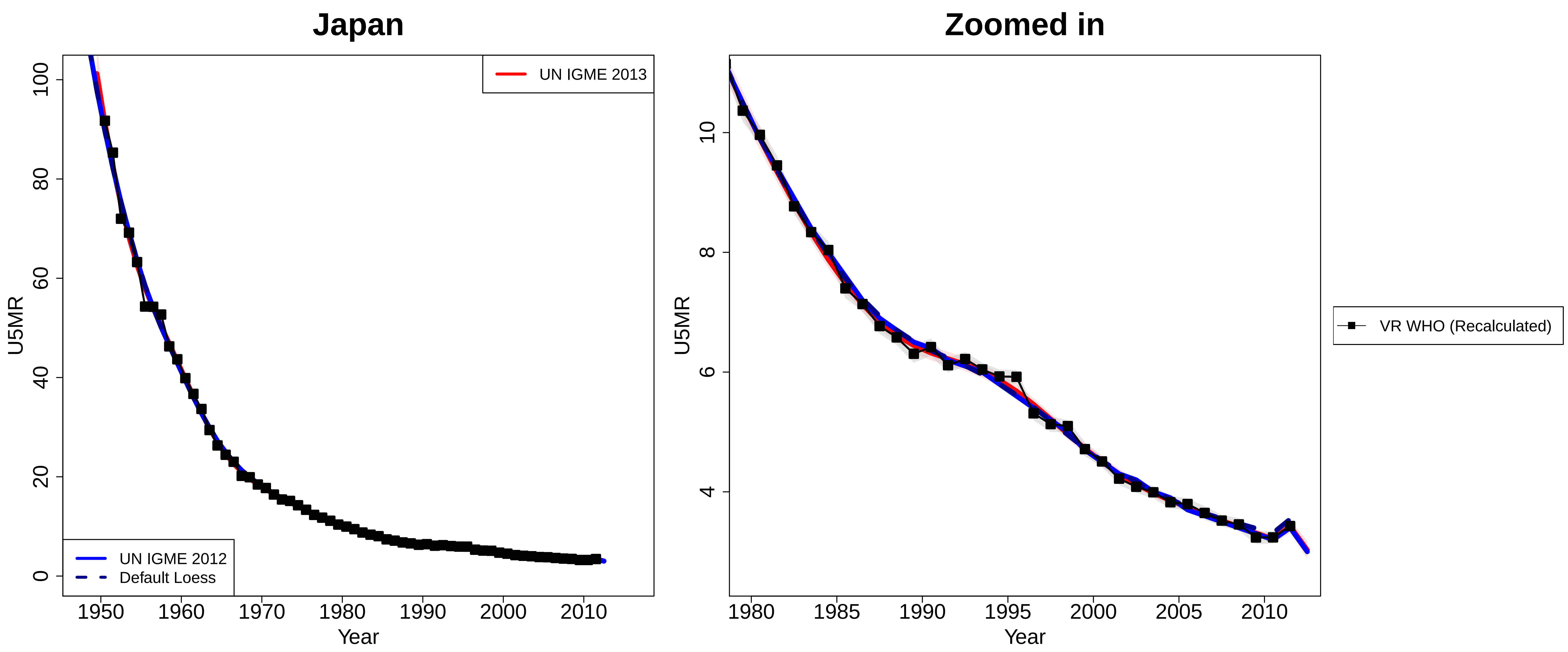
Japan
Zoomed in
UN IGME 2013
UN IGME 2012
Default Loess
VR WHO (Recalculated)
U5MR
Year
0
20
40
60
80
100
1950
1960
1970
1980
1990
2000
2010
4
6
8
10
1985
1995
2005

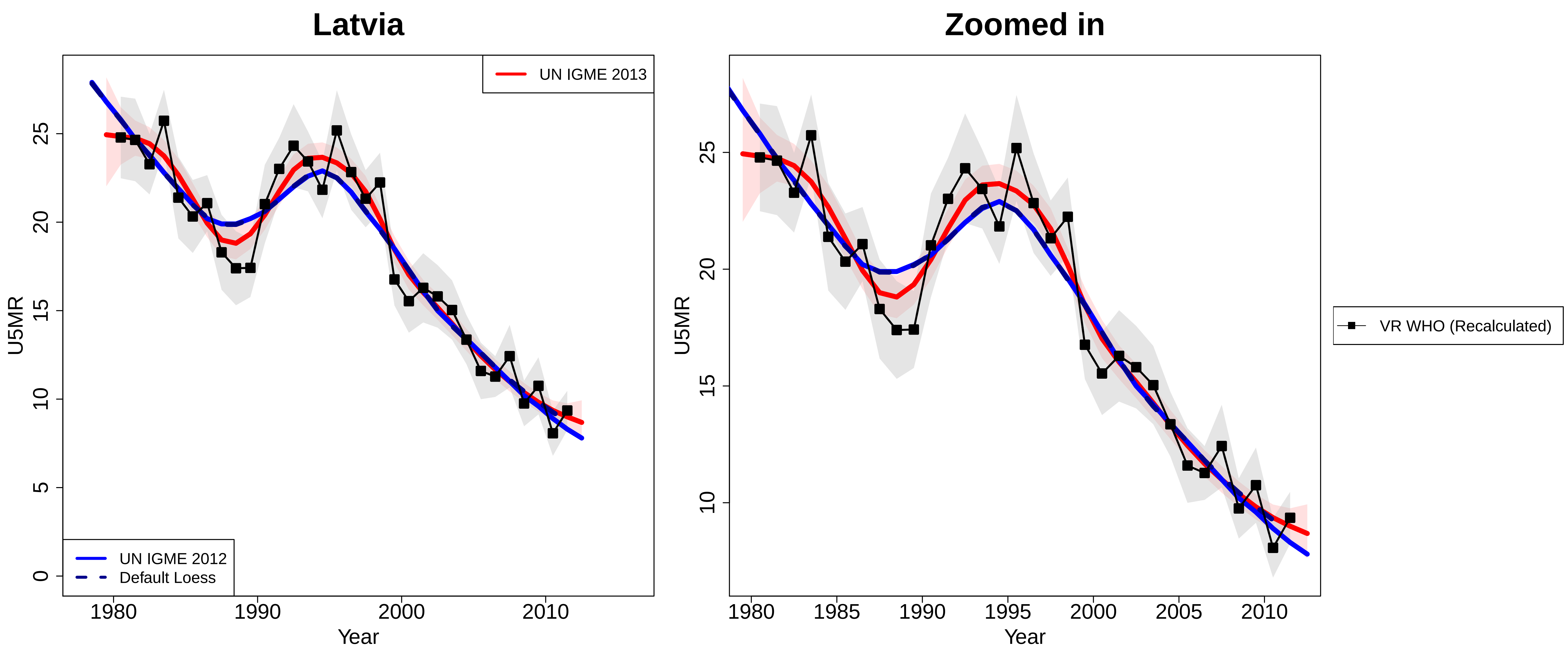
Latvia
Zoomed in
UN IGME 2013
UN IGME 2012
Default Loess
VR WHO (Recalculated)
U5MR
Year
0
5
10
15
20
25
1980
1985
1990
1995
2000
2005
2010

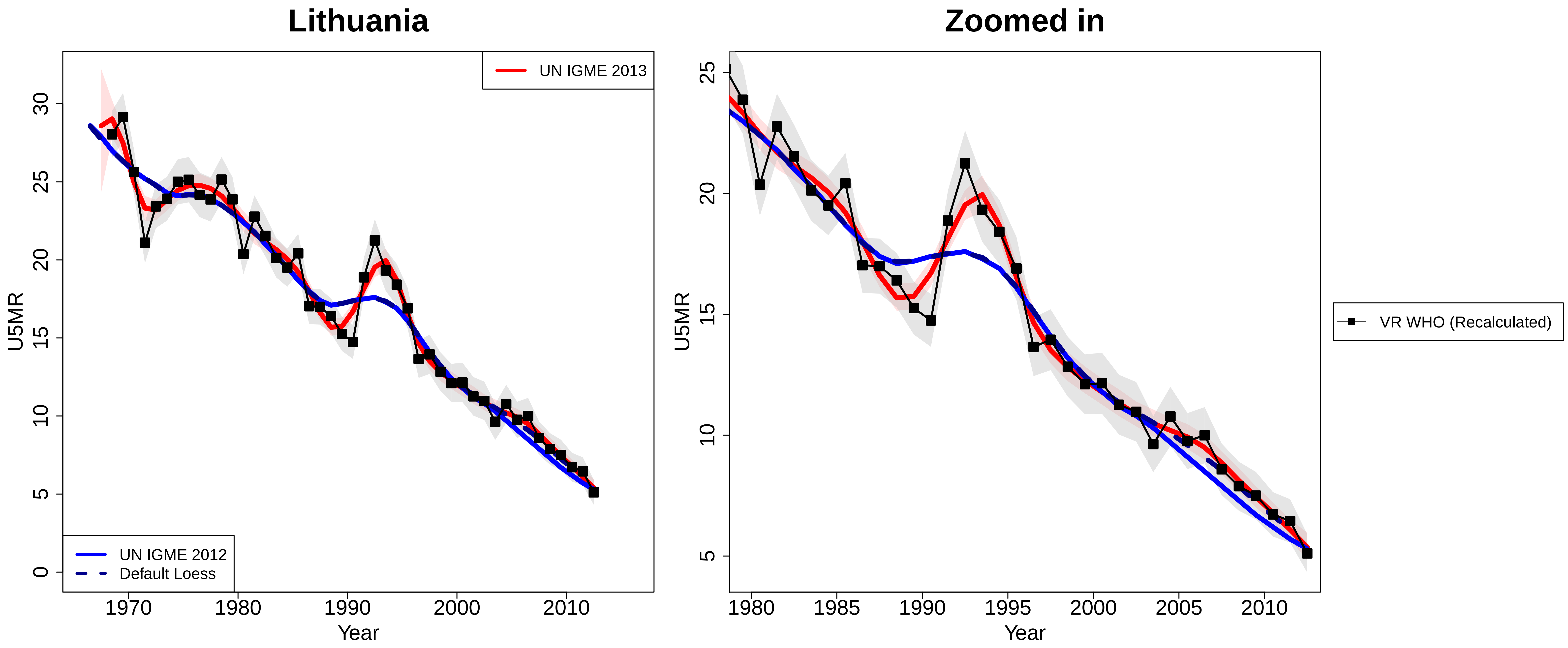
Lithuania
Zoomed in
UN IGME 2013
UN IGME 2012
Default Loess
VR WHO (Recalculated)
U5MR
Year
0
5
10
15
20
25
30
1970
1980
1990
2000
2010
1985
1995
2005

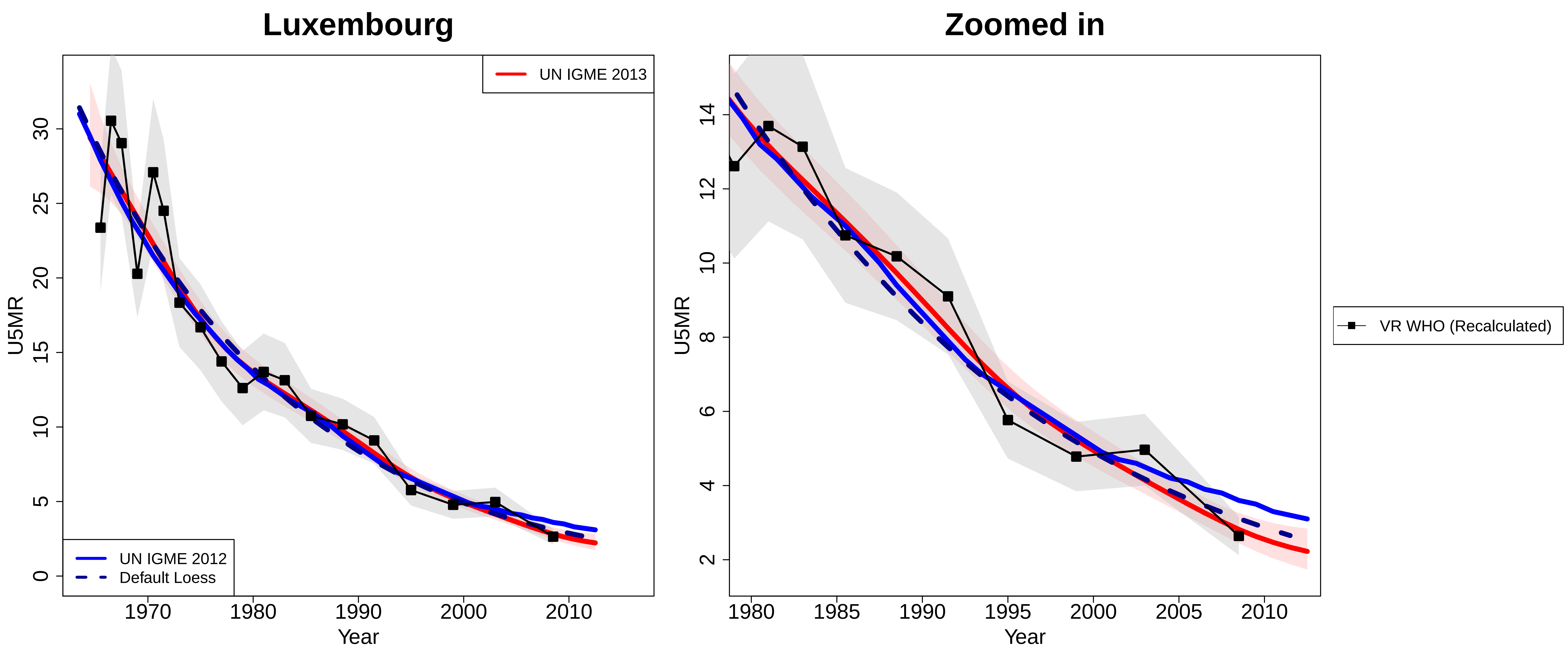

Luxembourg
Zoomed in
UN IGME 2013
UN IGME 2012
Default Loess
VR WHO (Recalculated)
U5MR
Year
0
5
10
15
20
25
30
1970
1980
1990
2000
2010
2
4
6
8
10
12
14
1980
1985
1990
1995
2000
2005
2010

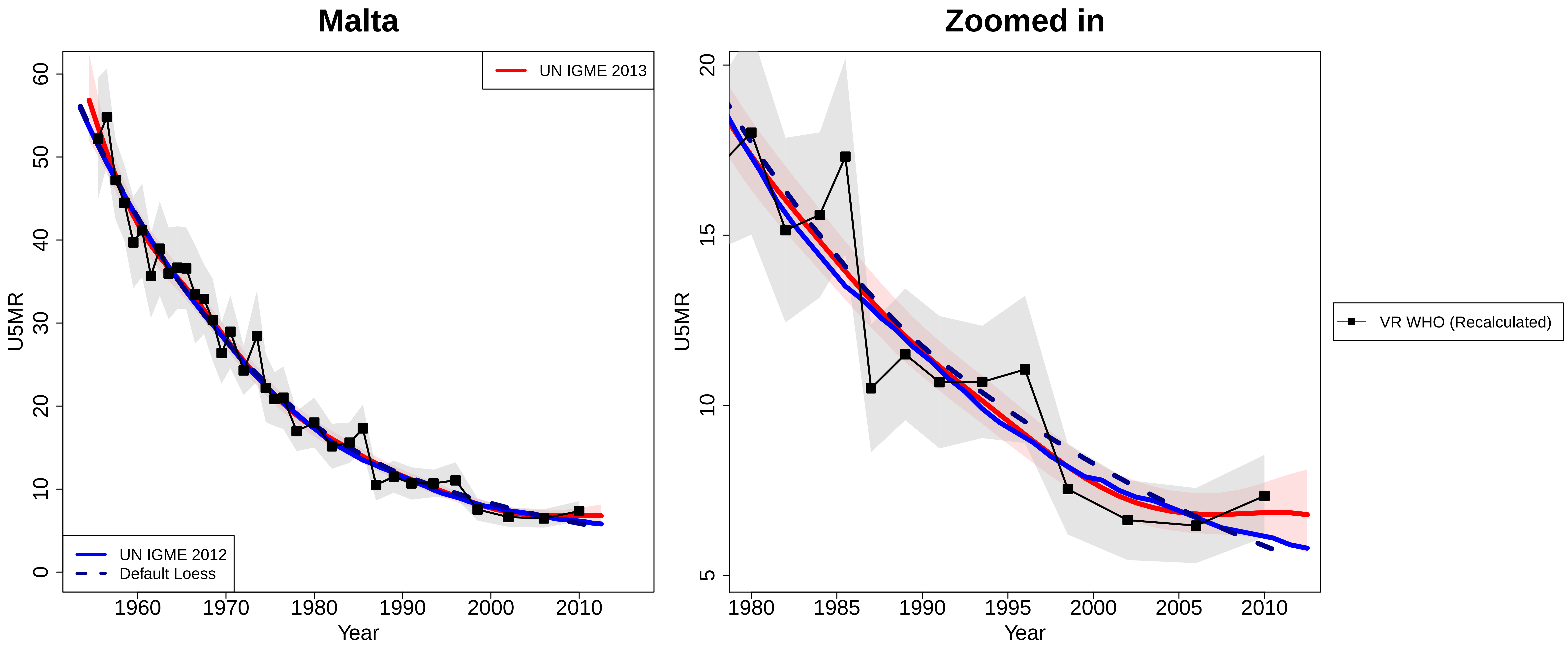

Malta
UN IGME 2013
UN IGME 2012
Default Loess
U5MR
Year
0
10
20
30
40
50
60
1960
1970
1980
1990
2000
2010
Zoomed in
VR WHO (Recalculated)
5
10
15
20
1980
1985
1990
1995
2000
2005
2010

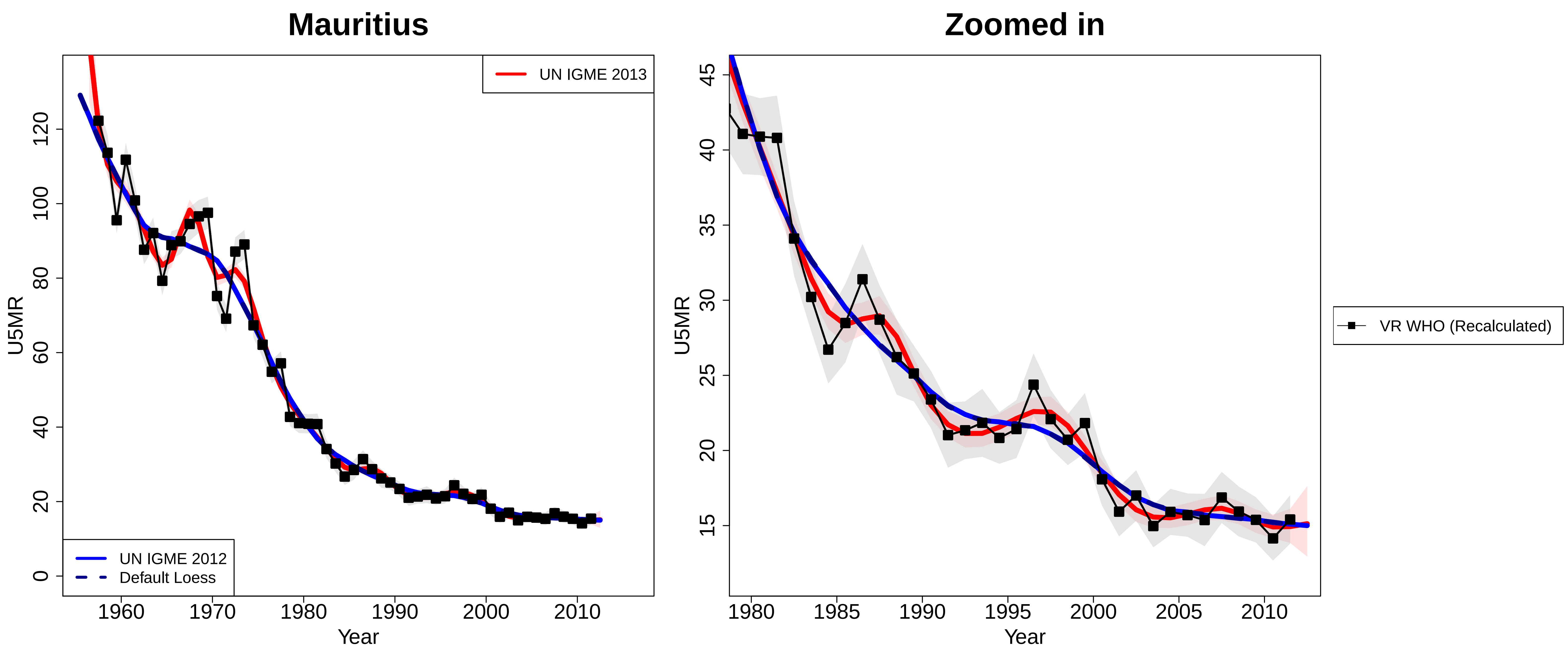
Mauritius
Zoomed in
U5MR
Year
UN IGME 2013
UN IGME 2012
Default Loess
VR WHO (Recalculated)
0
20
40
60
80
100
120
1960
1970
1980
1990
2000
2010
15
20
25
30
35
40
45
1980
1985
1990
1995
2000
2005
2010

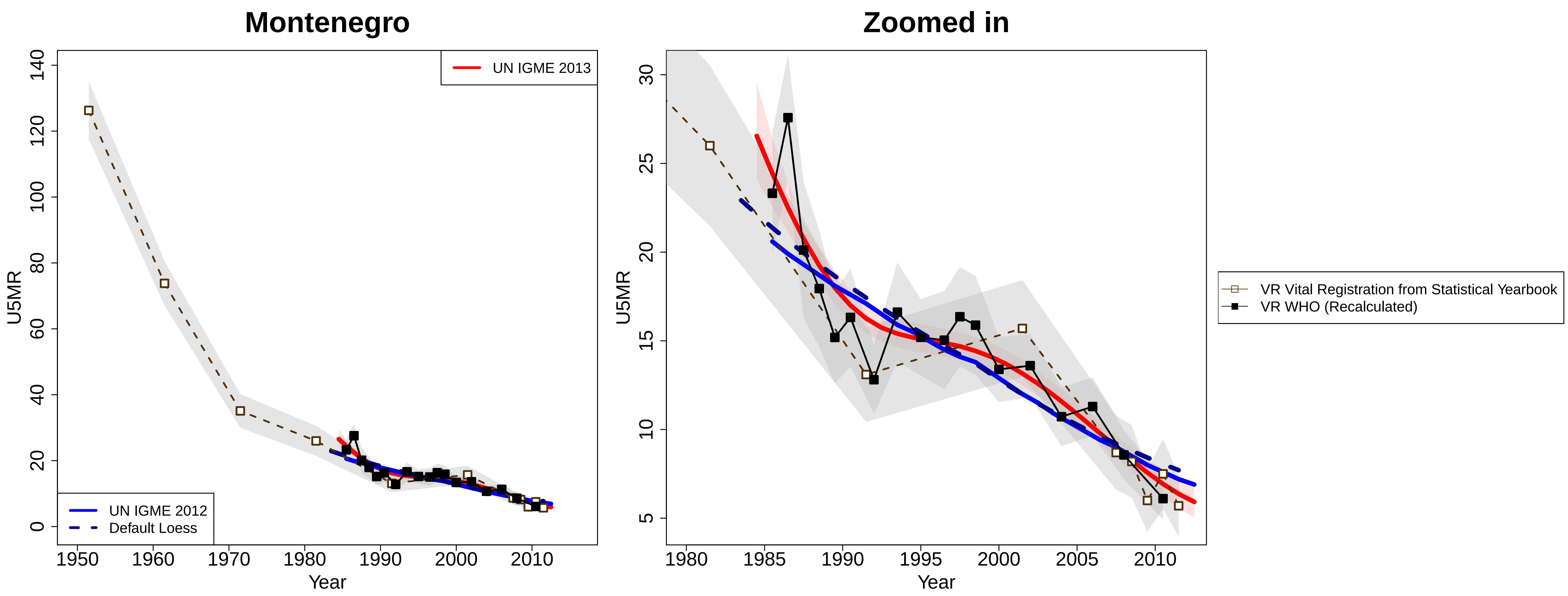

Montenegro
Zoomed in
U5MR
Year
UN IGME 2013
UN IGME 2012
Default Loess
VR Vital Registration from Statistical Yearbook
VR WHO (Recalculated)
0
20
40
60
80
100
120
140
1950
1960
1970
1980
1990
2000
2010
5
10
15
20
25
30
1980
1985
1990
1995
2000
2005
2010

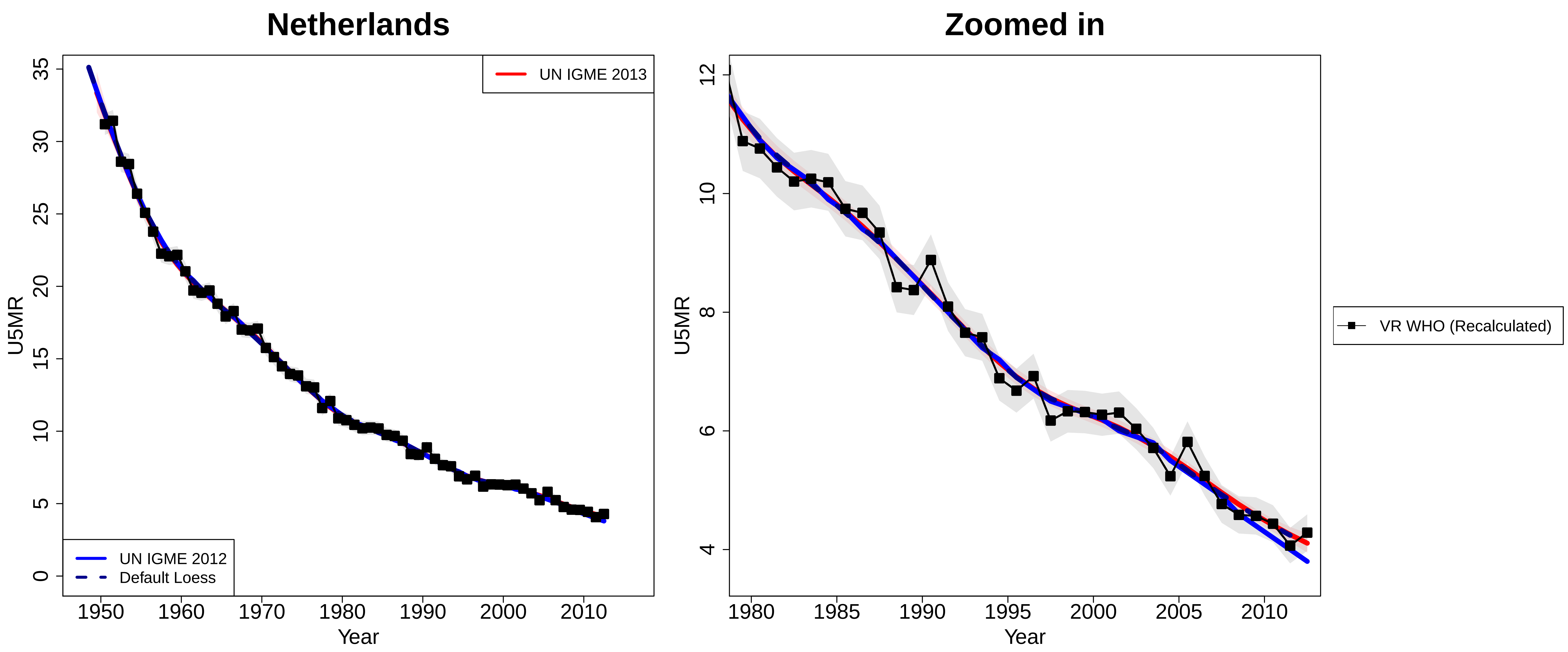
Netherlands
Zoomed in
UN IGME 2013
UN IGME 2012
Default Loess
VR WHO (Recalculated)
U5MR
Year
0
5
10
15
20
25
30
35
1950
1960
1970
1980
1990
2000
2010
4
6
8
10
12
1980
1985
1990
1995
2000
2005
2010

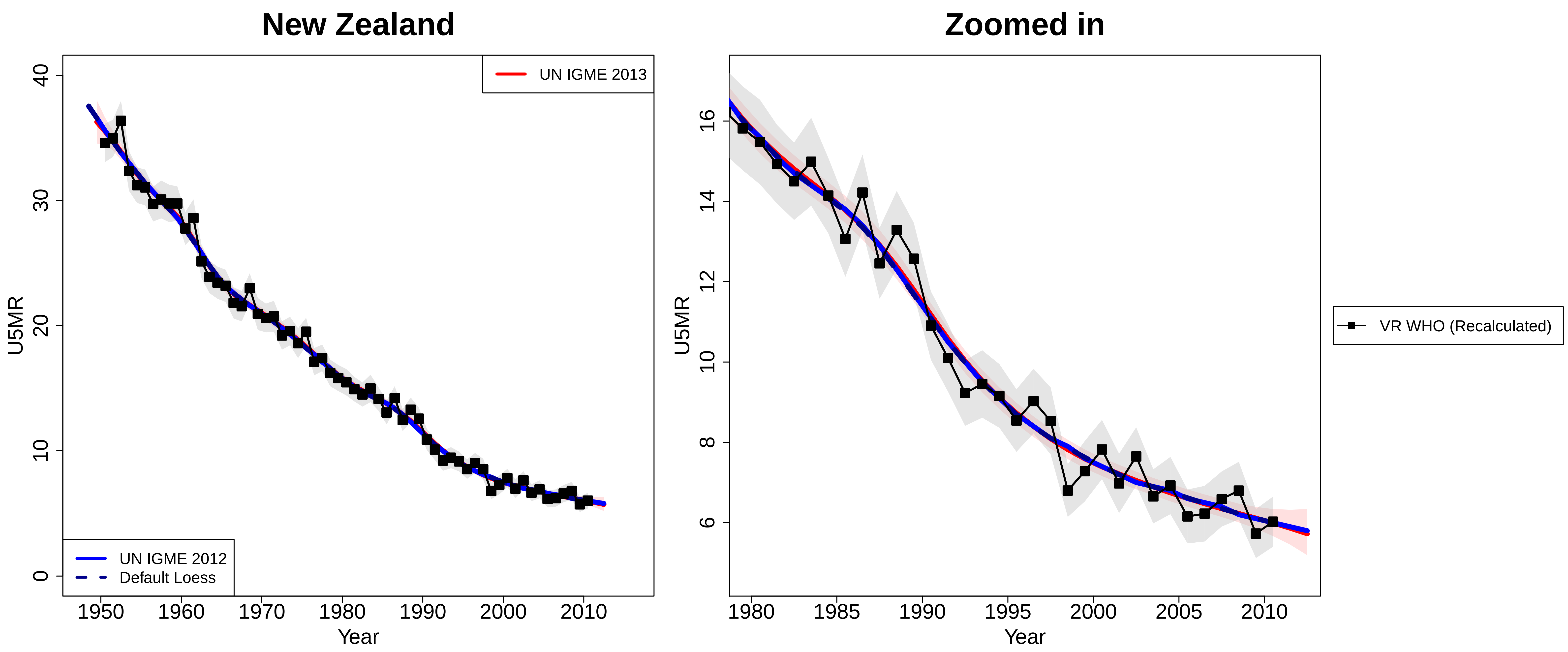

New Zealand
UN IGME 2013
UN IGME 2012
Default Loess
U5MR
0
10
20
30
40
1950
1960
1970
1980
1990
2000
2010
Year
Zoomed in
U5MR
6
8
10
12
14
16
1980
1985
1990
1995
2000
2005
2010
Year
VR WHO (Recalculated)

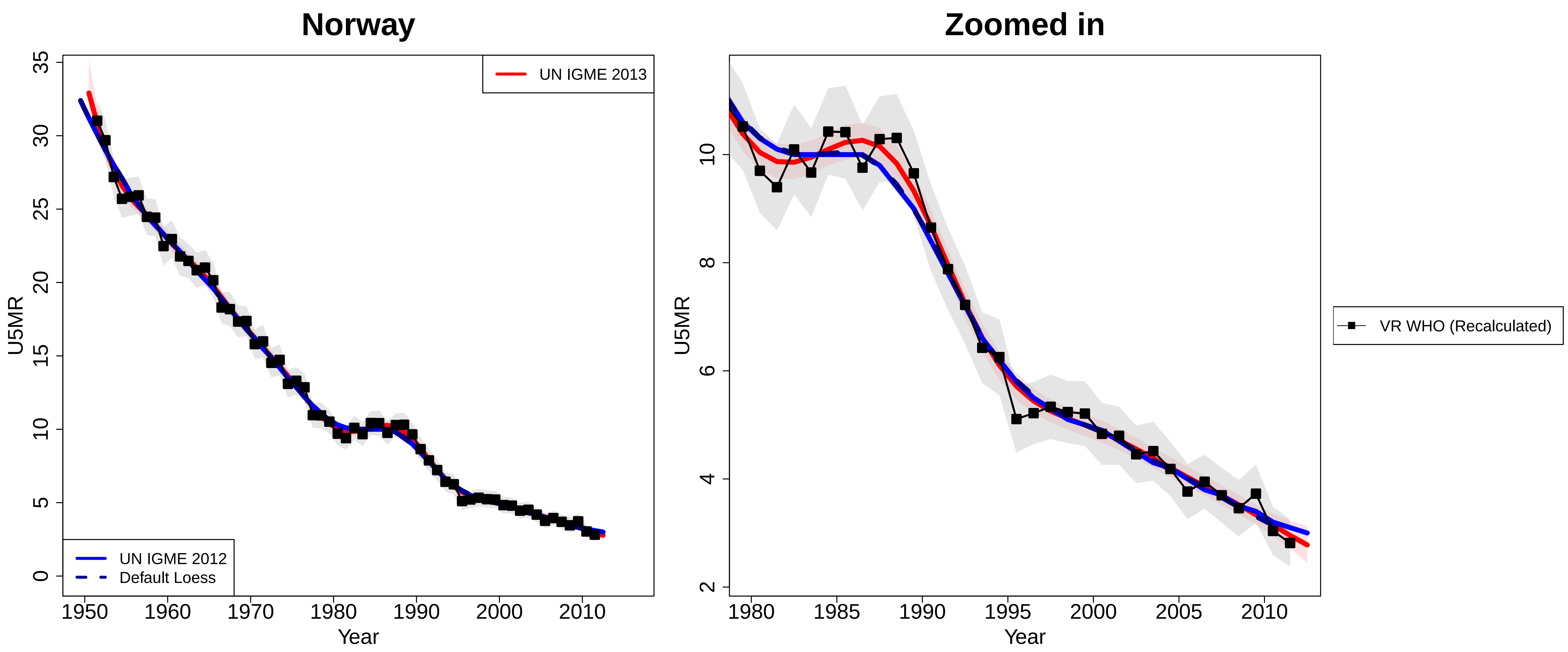

Norway
Zoomed in
U5MR
Year
UN IGME 2013
UN IGME 2012
Default Loess
VR WHO (Recalculated)
0
5
10
15
20
25
30
35
1950
1960
1970
1980
1990
2000
2010
2
4
6
8
10
1980
1985
1990
1995
2000
2005
2010

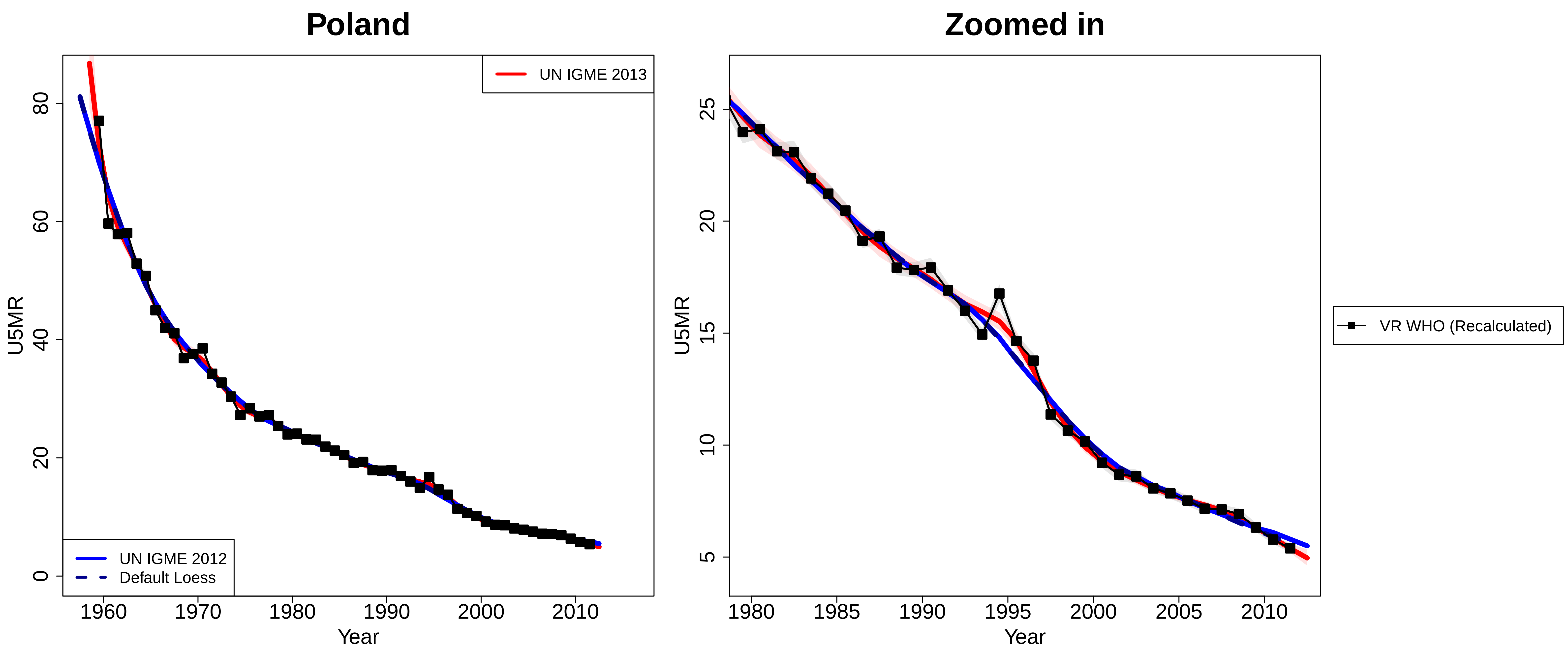

Poland
UN IGME 2013
UN IGME 2012
Default Loess
U5MR
Year
0
20
40
60
80
1960
1970
1980
1990
2000
2010
Zoomed in
U5MR
Year
5
10
15
20
25
1980
1985
1990
1995
2000
2005
2010
VR WHO (Recalculated)

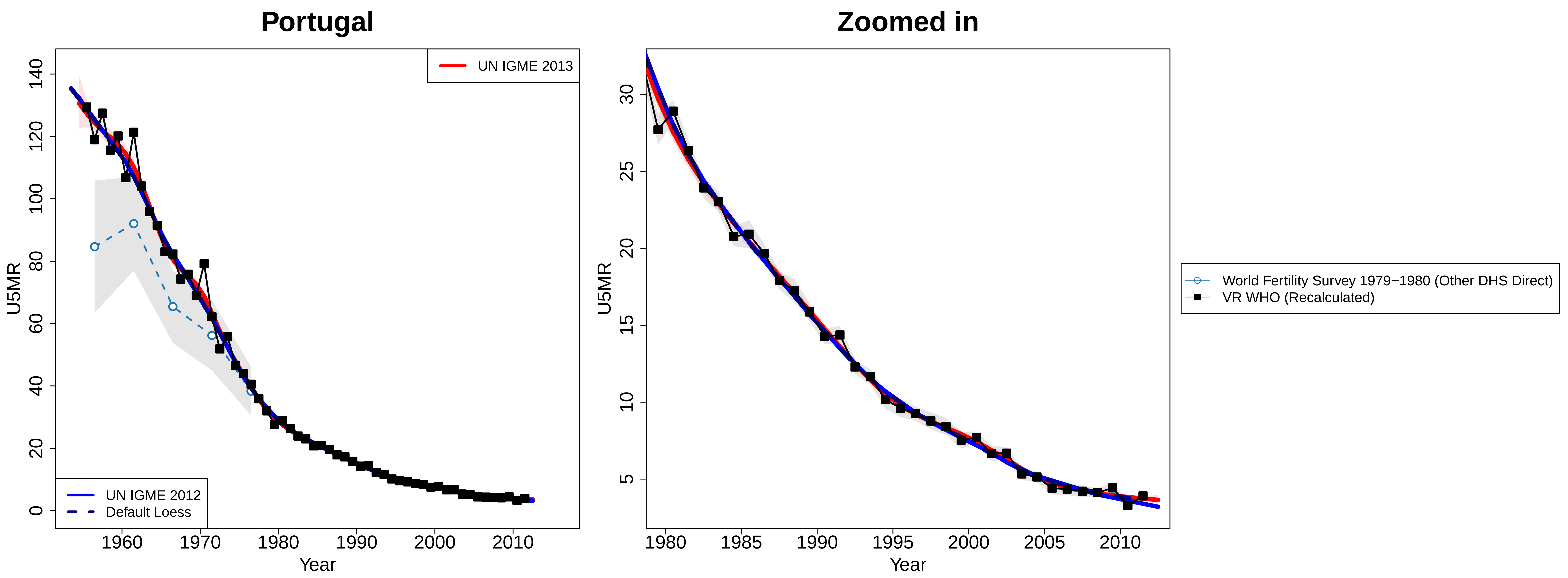

Portugal
Zoomed in
UN IGME 2013
UN IGME 2012
Default Loess
World Fertility Survey 1979−1980 (Other DHS Direct)
VR WHO (Recalculated)
U5MR
Year
0
20
40
60
80
100
120
140
1960
1970
1980
1990
2000
2010
5
10
15
20
25
30
1980
1985
1990
1995
2000
2005
2010

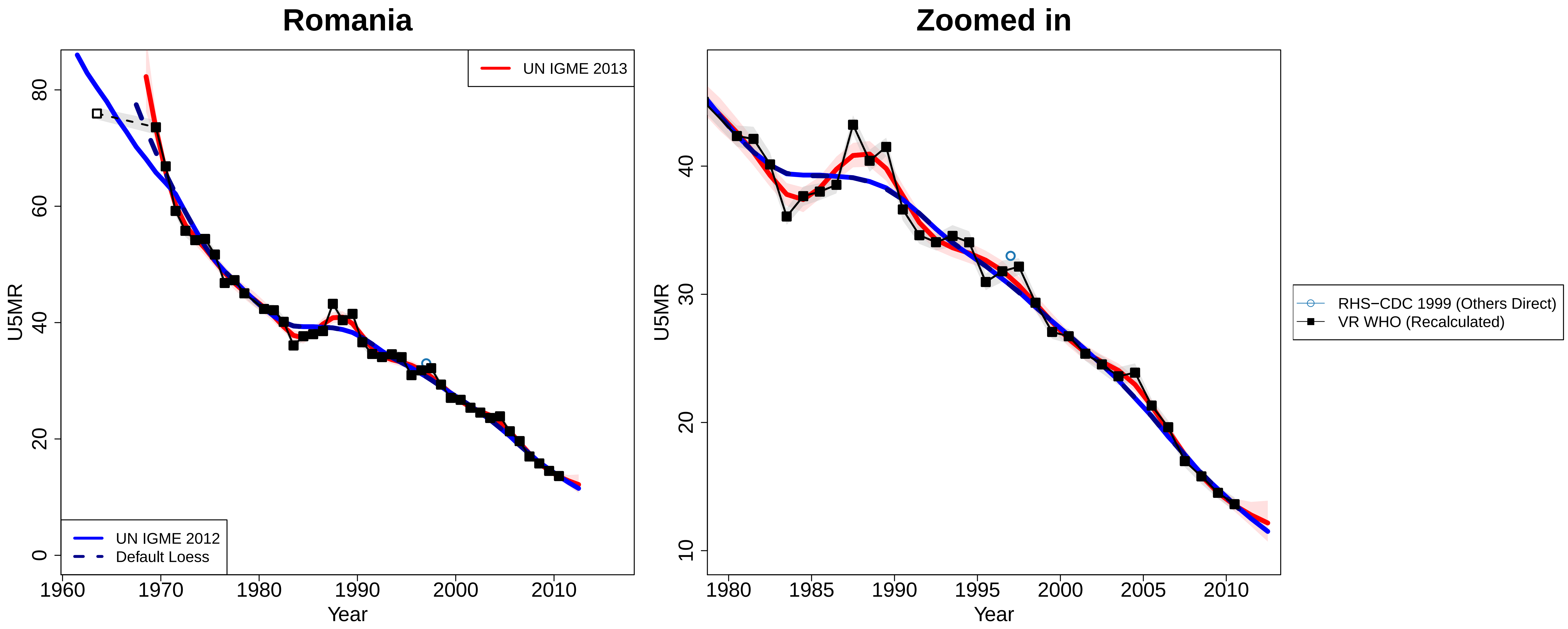

Romania
Zoomed in
UN IGME 2013
UN IGME 2012
Default Loess
RHS−CDC 1999 (Others Direct)
VR WHO (Recalculated)
U5MR
Year
0
20
40
60
80
10
30
1960
1970
1980
1990
2000
2010
1985
1995
2005

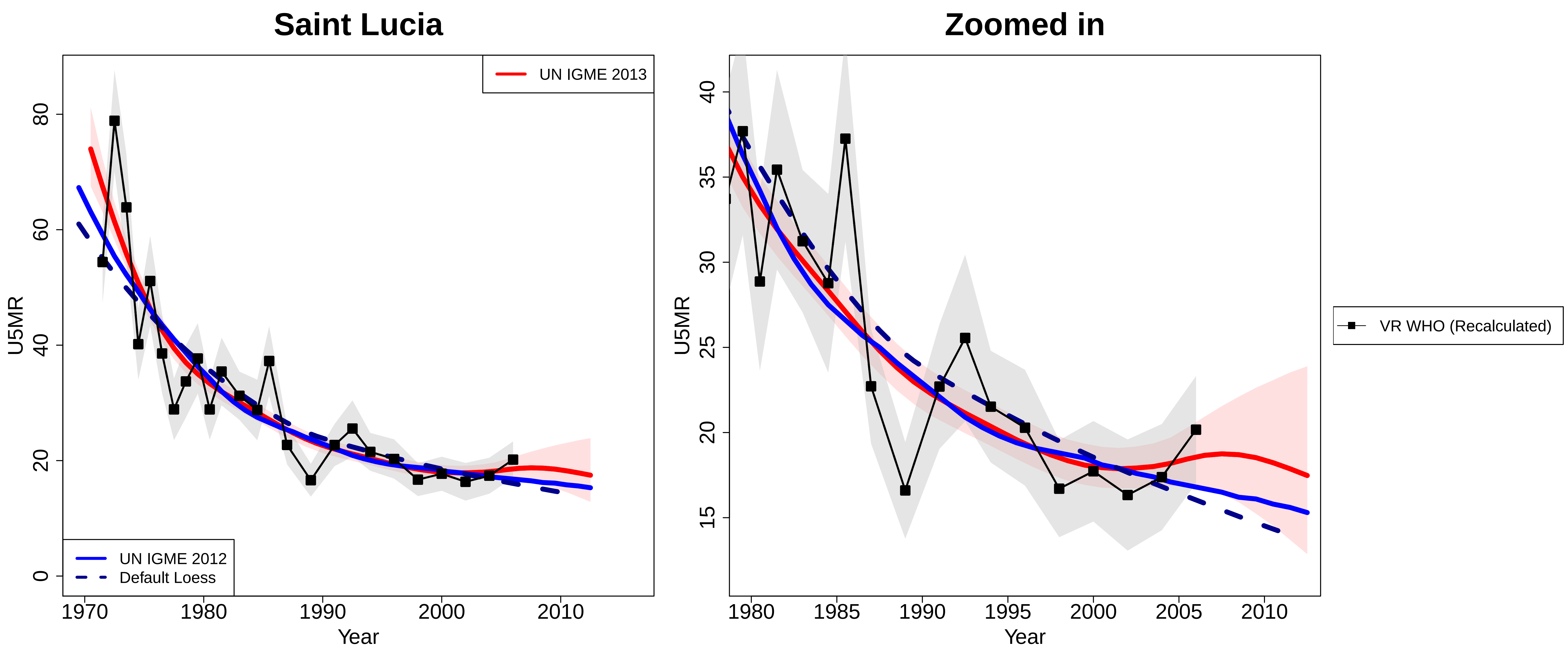
Saint Lucia
Zoomed in
U5MR
Year
UN IGME 2013
UN IGME 2012
Default Loess
VR WHO (Recalculated)
0
20
40
60
80
1970
1980
1990
2000
2010
15
20
25
30
35
40
1980
1985
1990
1995
2000
2005
2010

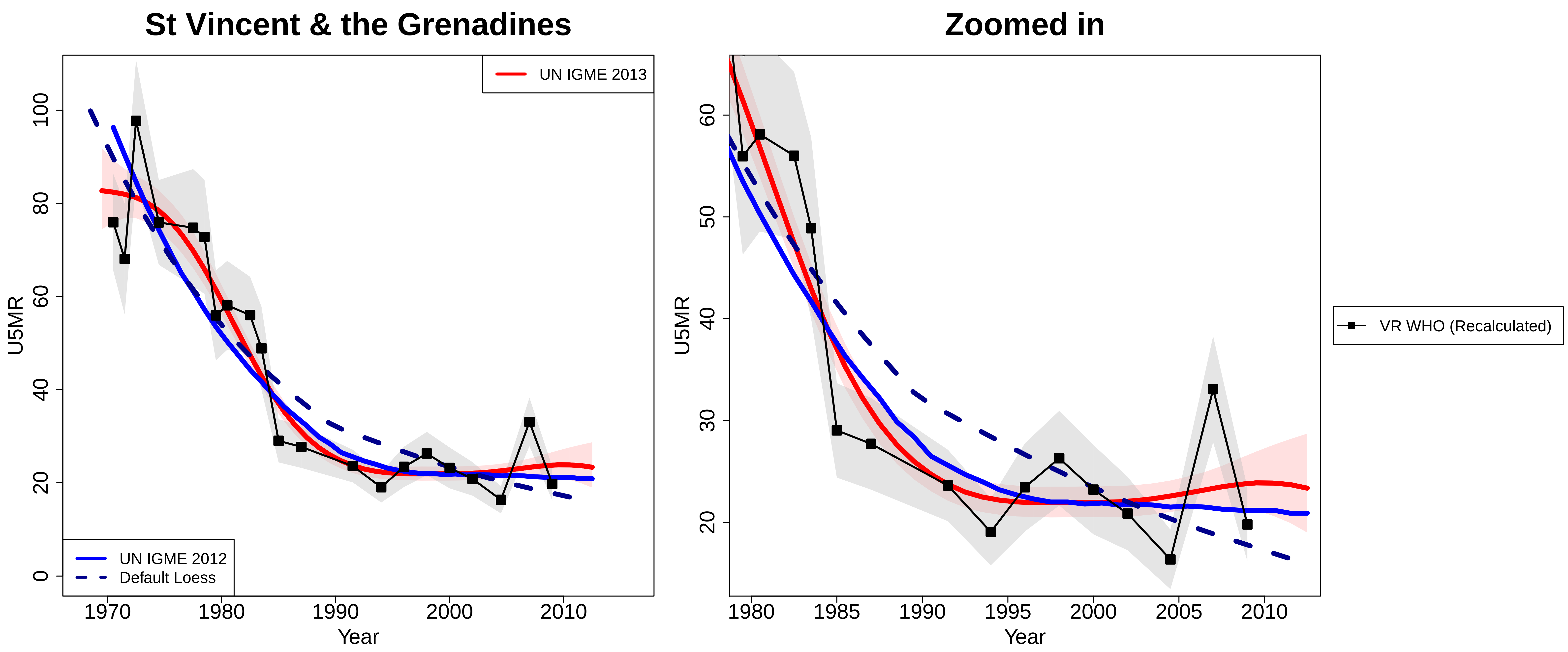

St Vincent & the Grenadines
Zoomed in
UN IGME 2013
UN IGME 2012
Default Loess
VR WHO (Recalculated)
U5MR
Year
0
20
40
60
80
100
1970
1980
1990
2000
2010
30
50
1985
1995
2005

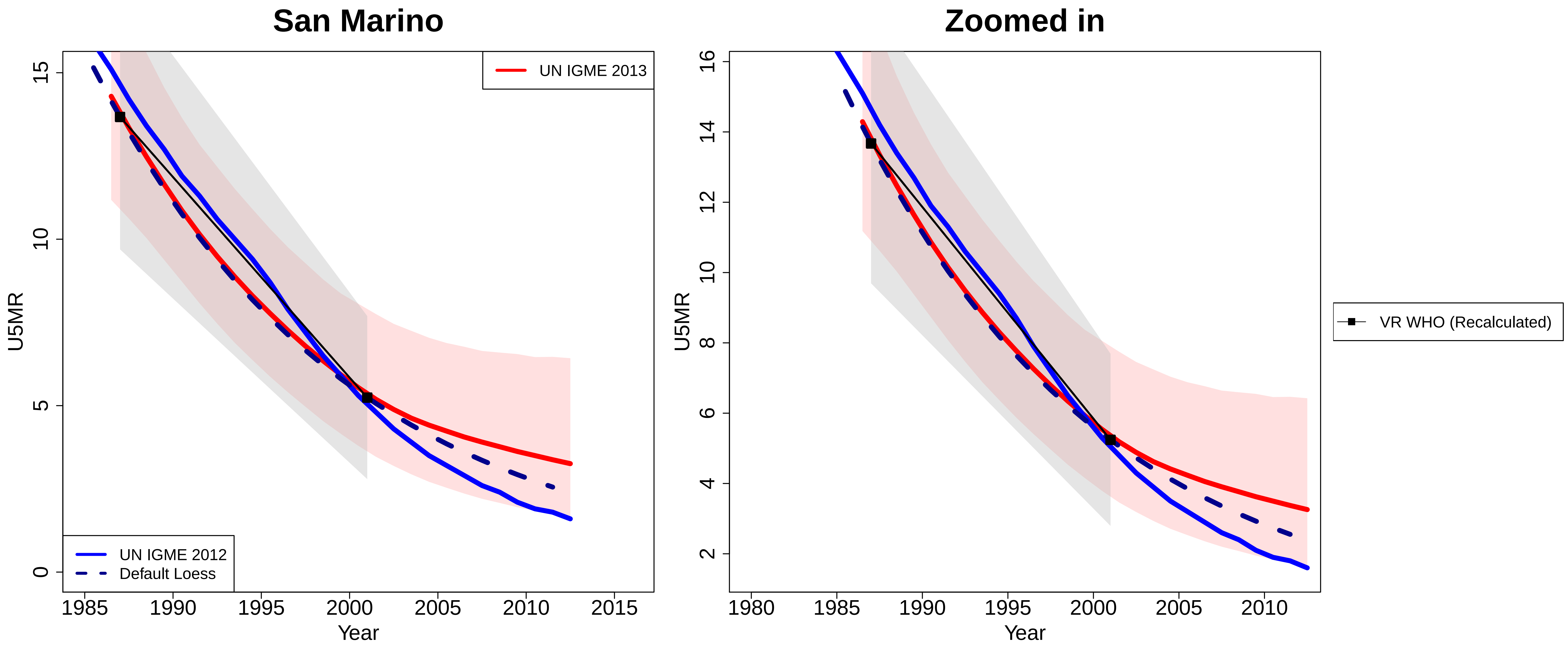
San Marino
Zoomed in
UN IGME 2013
UN IGME 2012
Default Loess
VR WHO (Recalculated)
U5MR
Year
0
5
10
15
2
4
6
8
10
12
14
16
1980
1985
1990
1995
2000
2005
2010
2015

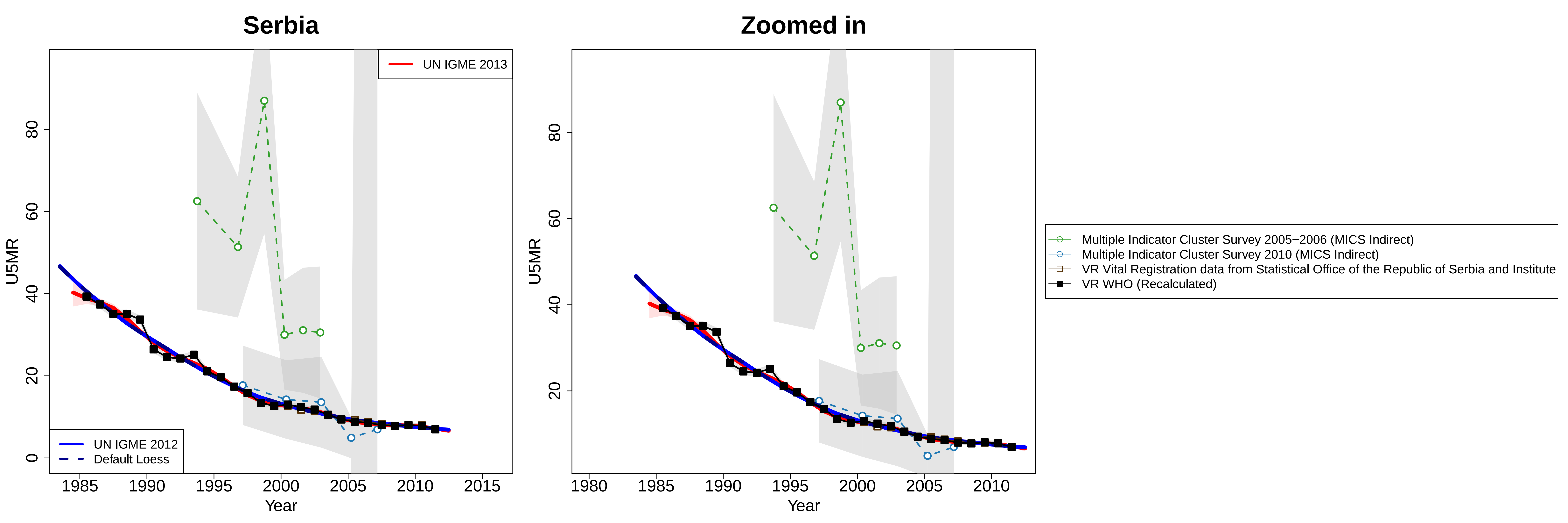

Serbia
Zoomed in
UN IGME 2013
UN IGME 2012
Default Loess
U5MR
Year
Multiple Indicator Cluster Survey 2005−2006 (MICS Indirect)
Multiple Indicator Cluster Survey 2010 (MICS Indirect)
VR Vital Registration data from Statistical Office of the Republic of Serbia and Institute
VR WHO (Recalculated)

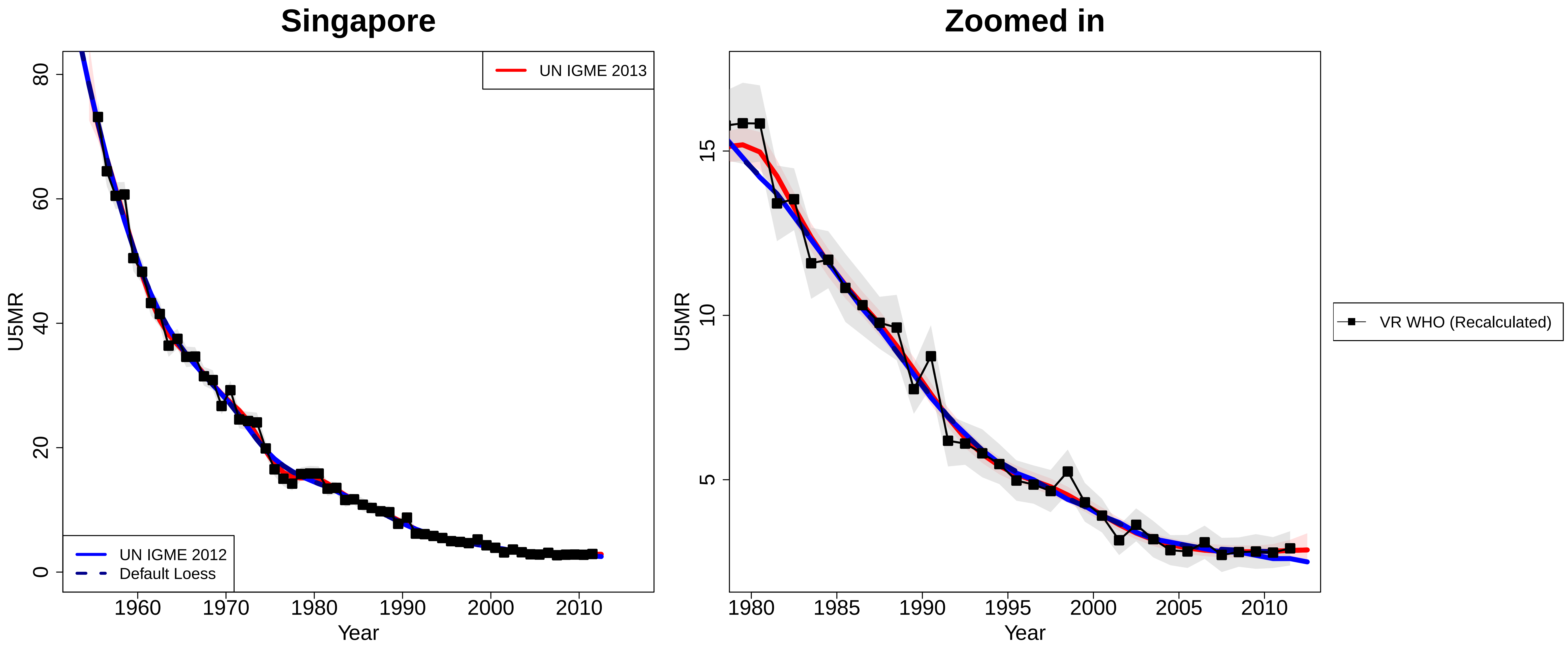
Singapore
Zoomed in
UN IGME 2013
UN IGME 2012
Default Loess
VR WHO (Recalculated)
U5MR
Year
0
20
40
60
80
1960
1970
1980
1990
2000
2010
5
10
15
1985
1995
2005

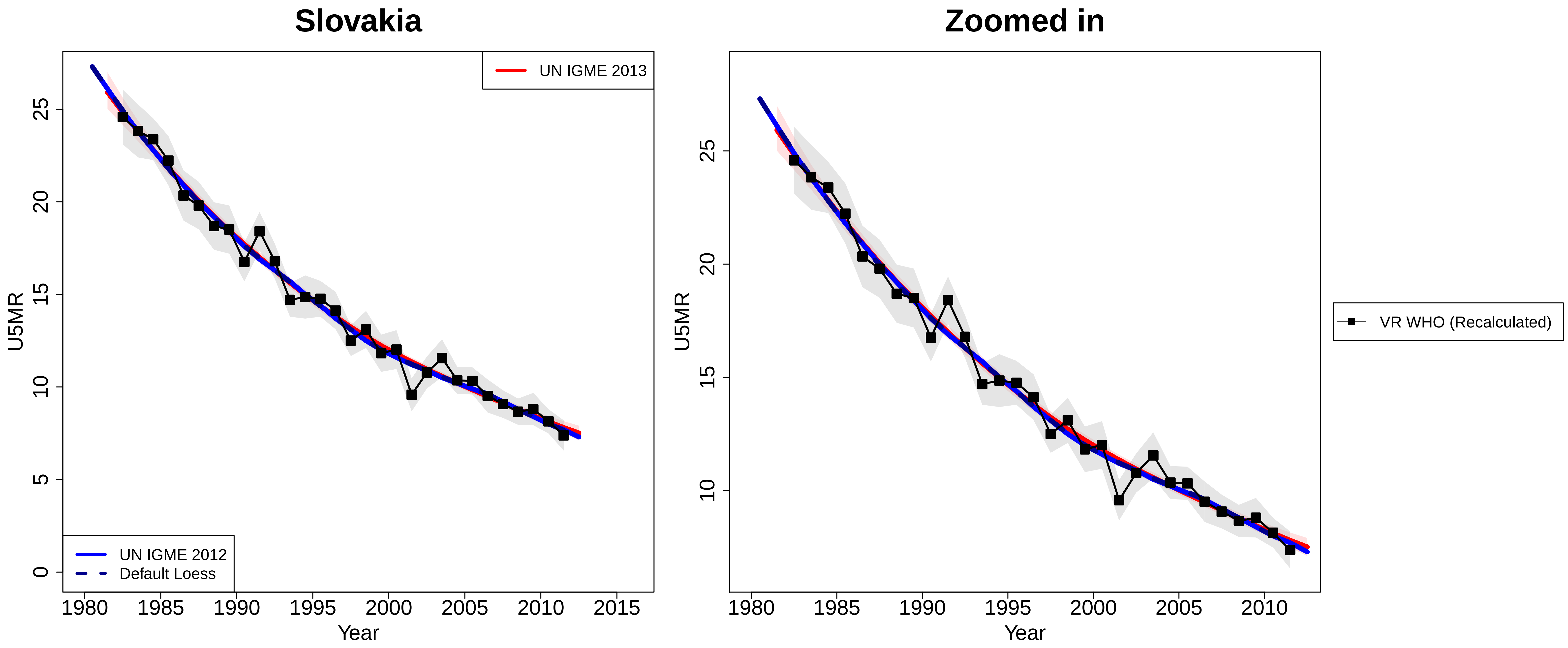

Slovakia
Zoomed in
UN IGME 2013
UN IGME 2012
Default Loess
VR WHO (Recalculated)
U5MR
Year

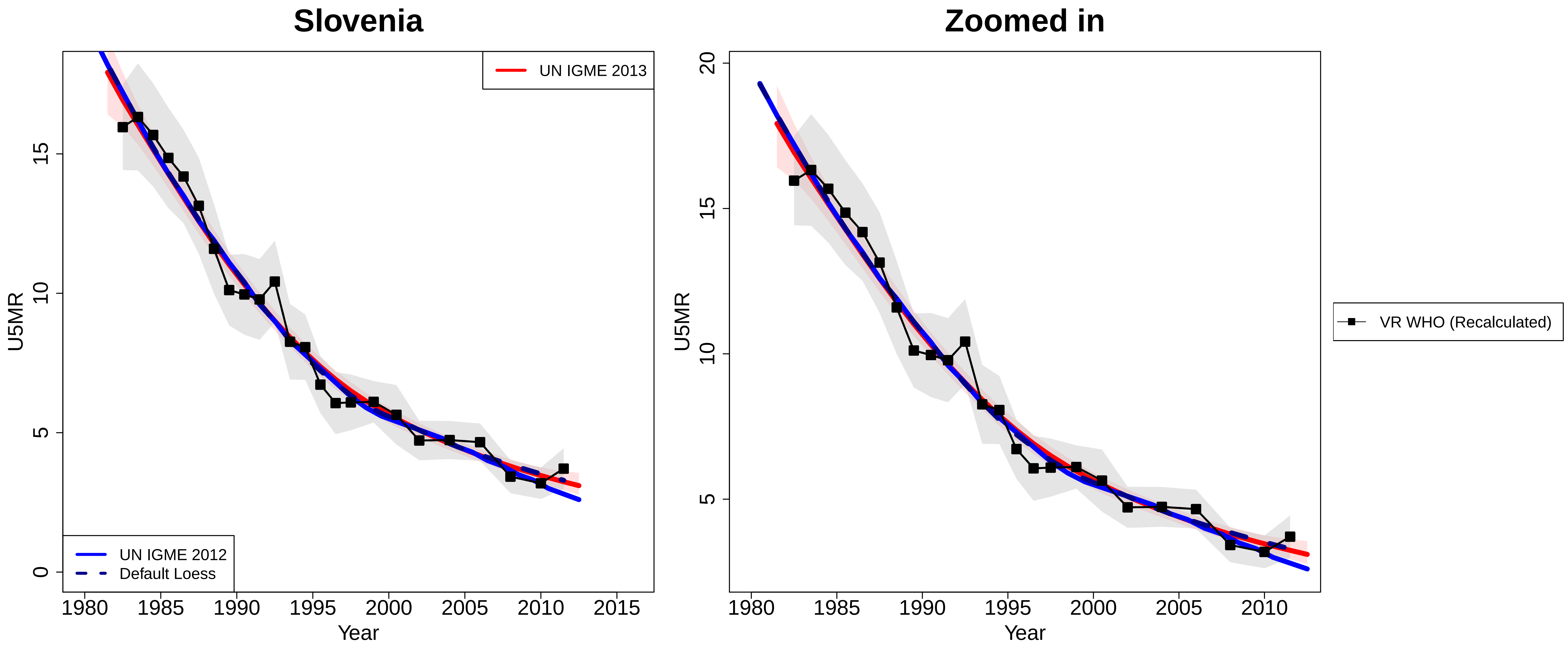

Slovenia
Zoomed in
UN IGME 2013
UN IGME 2012
Default Loess
VR WHO (Recalculated)
U5MR
Year
0
5
10
15
20
1980
1985
1990
1995
2000
2005
2010
2015

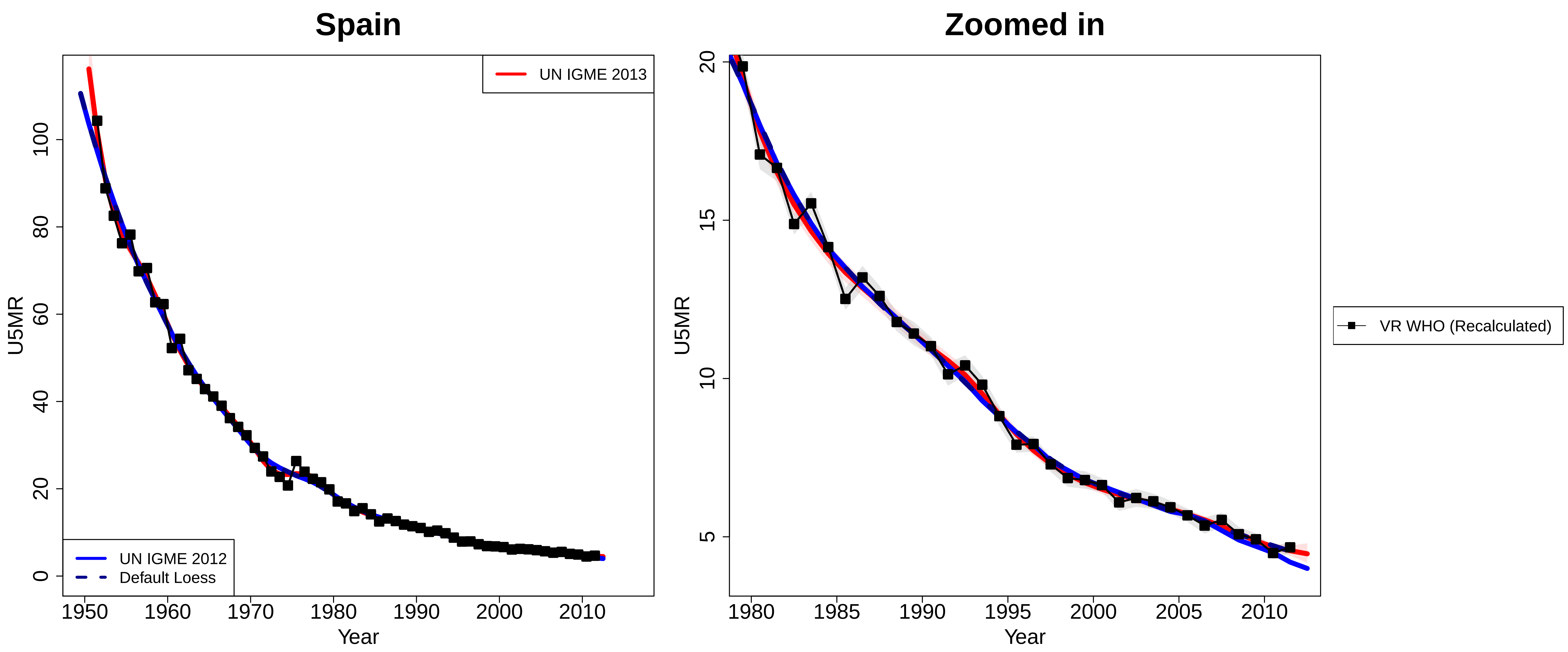

Spain
Zoomed in
UN IGME 2013
UN IGME 2012
Default Loess
VR WHO (Recalculated)
U5MR
Year
0
20
40
60
80
100
1950
1960
1970
1980
1990
2000
2010
5
10
15
20
1980
1985
1990
1995
2000
2005
2010

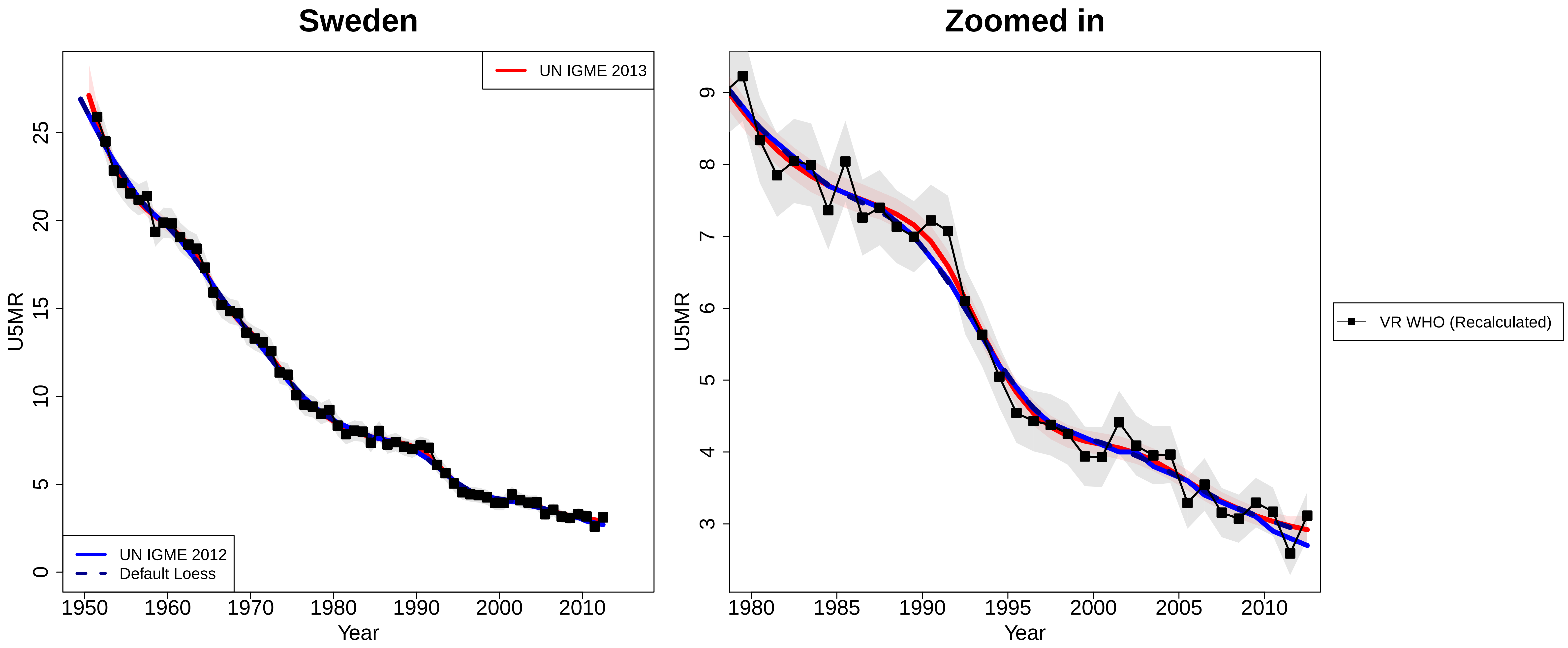

Sweden
UN IGME 2013
UN IGME 2012
Default Loess
U5MR
Year
Zoomed in
VR WHO (Recalculated)
U5MR
Year

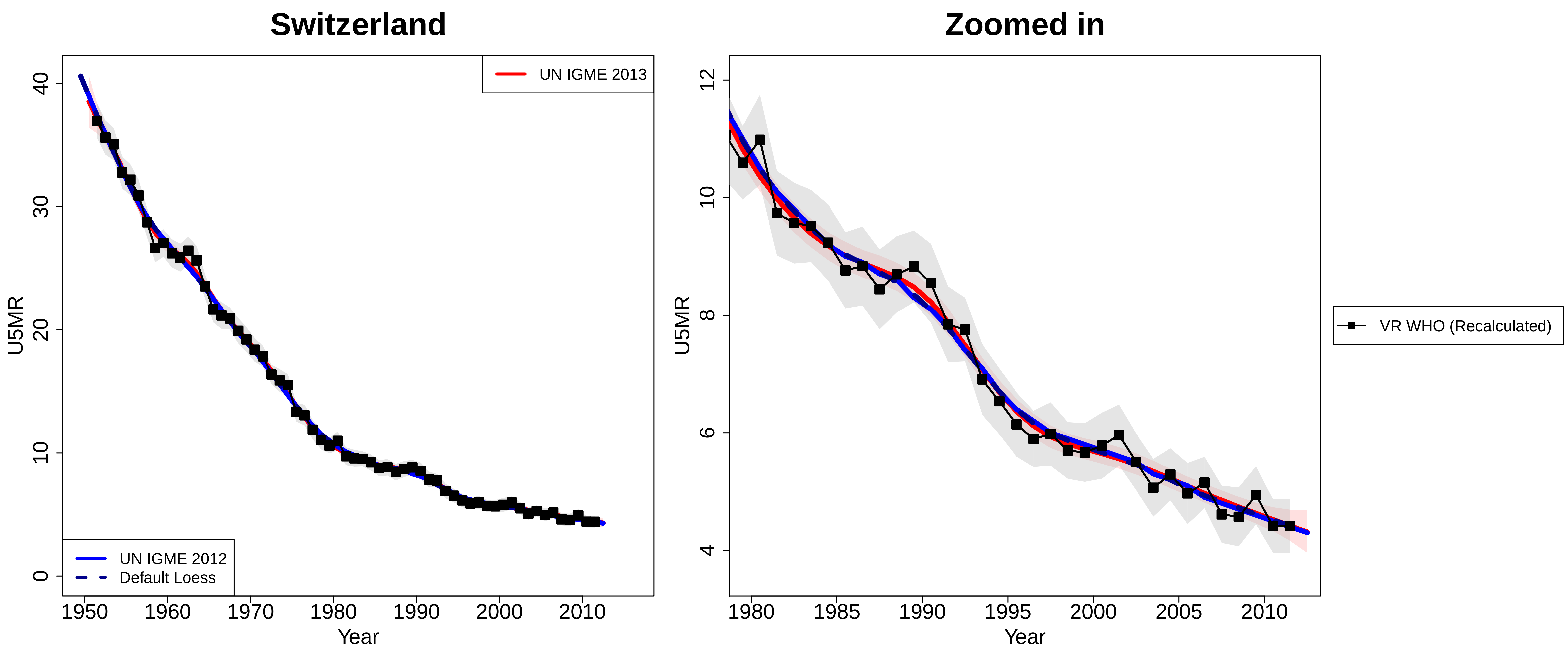

Switzerland
Zoomed in
UN IGME 2013
UN IGME 2012
Default Loess
VR WHO (Recalculated)
U5MR
Year
0
10
20
30
40
4
6
8
10
12
1950
1960
1970
1980
1990
2000
2010
1985
1995
2005

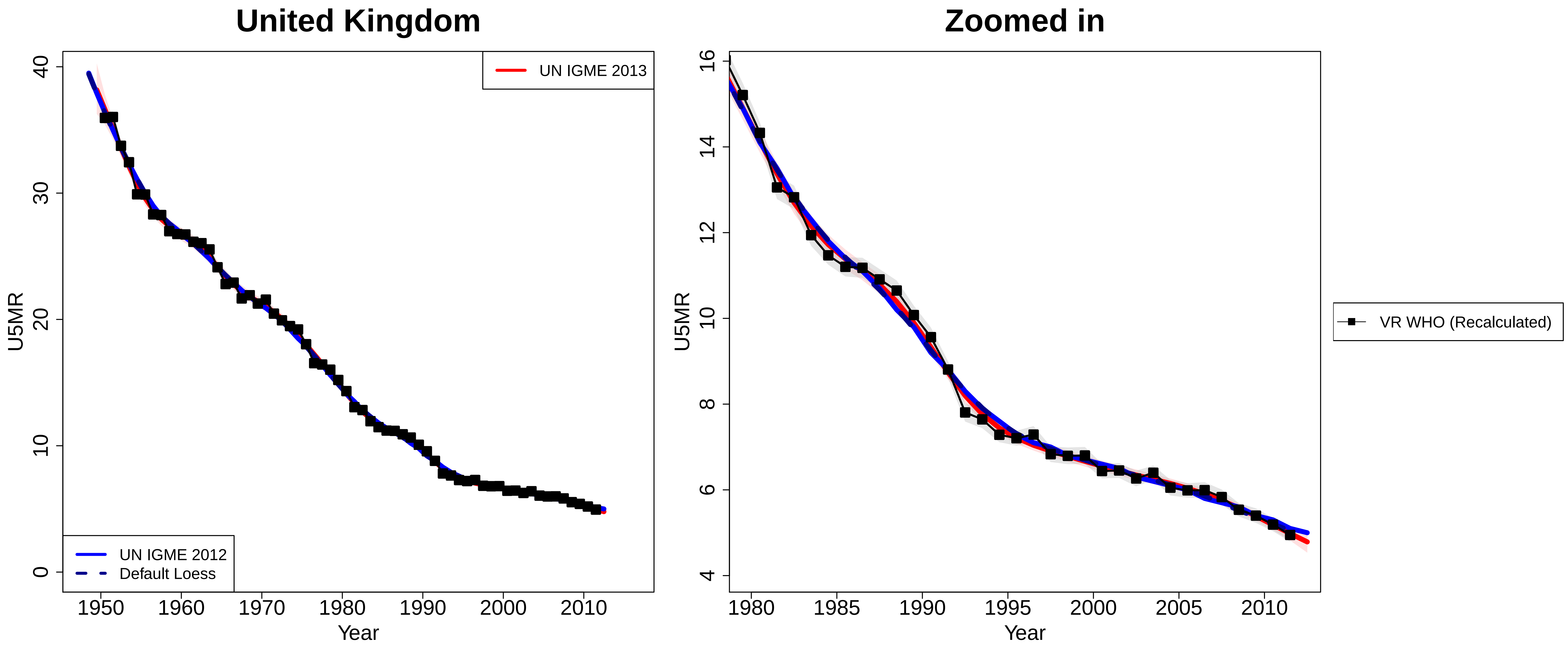
United Kingdom
UN IGME 2013
UN IGME 2012
Default Loess
U5MR
0
10
20
30
40
1950
1960
1970
1980
1990
2000
2010
Year
Zoomed in
U5MR
4
6
8
10
12
14
16
1980
1985
1990
1995
2000
2005
2010
Year
VR WHO (Recalculated)

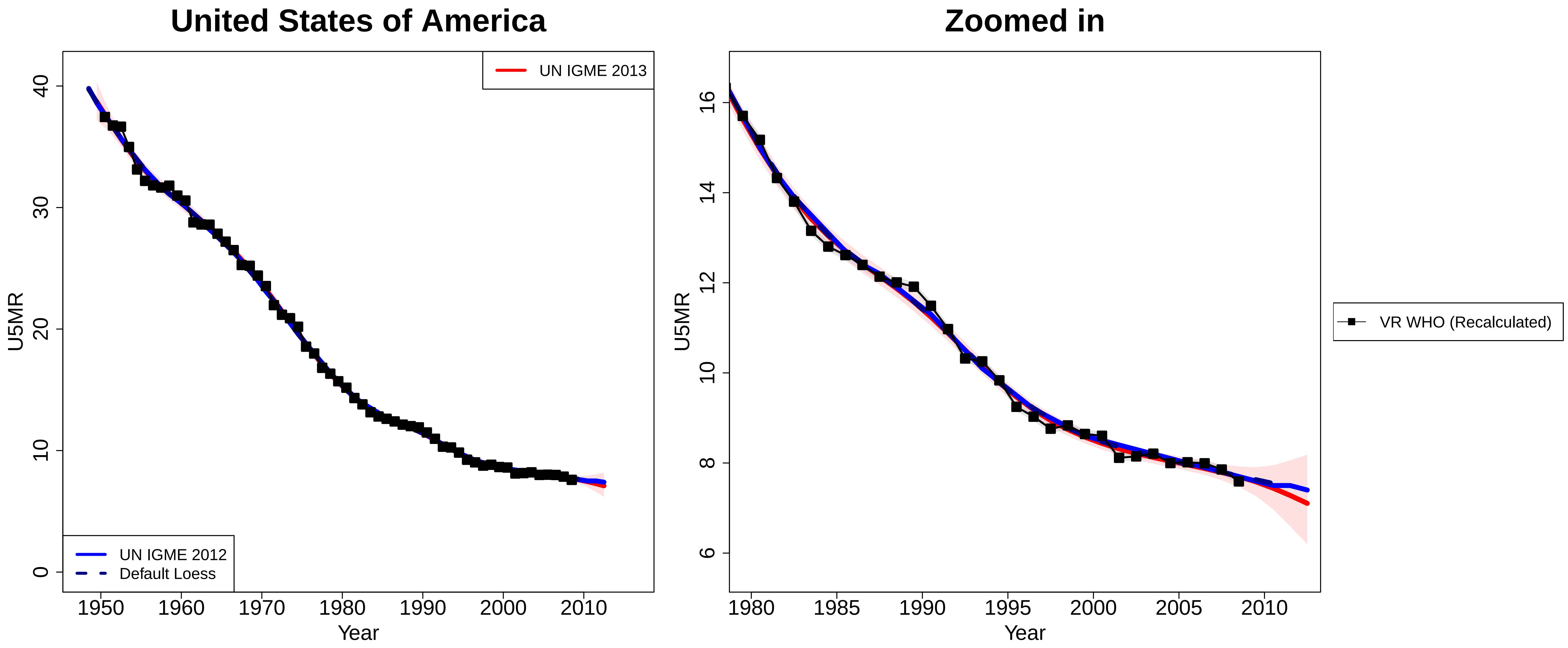
United States of America
Zoomed in
UN IGME 2013
UN IGME 2012
Default Loess
VR WHO (Recalculated)
U5MR
Year
0
10
20
30
40
6
8
10
12
14
16
1950
1960
1970
1980
1990
2000
2010
1985
1995
2005

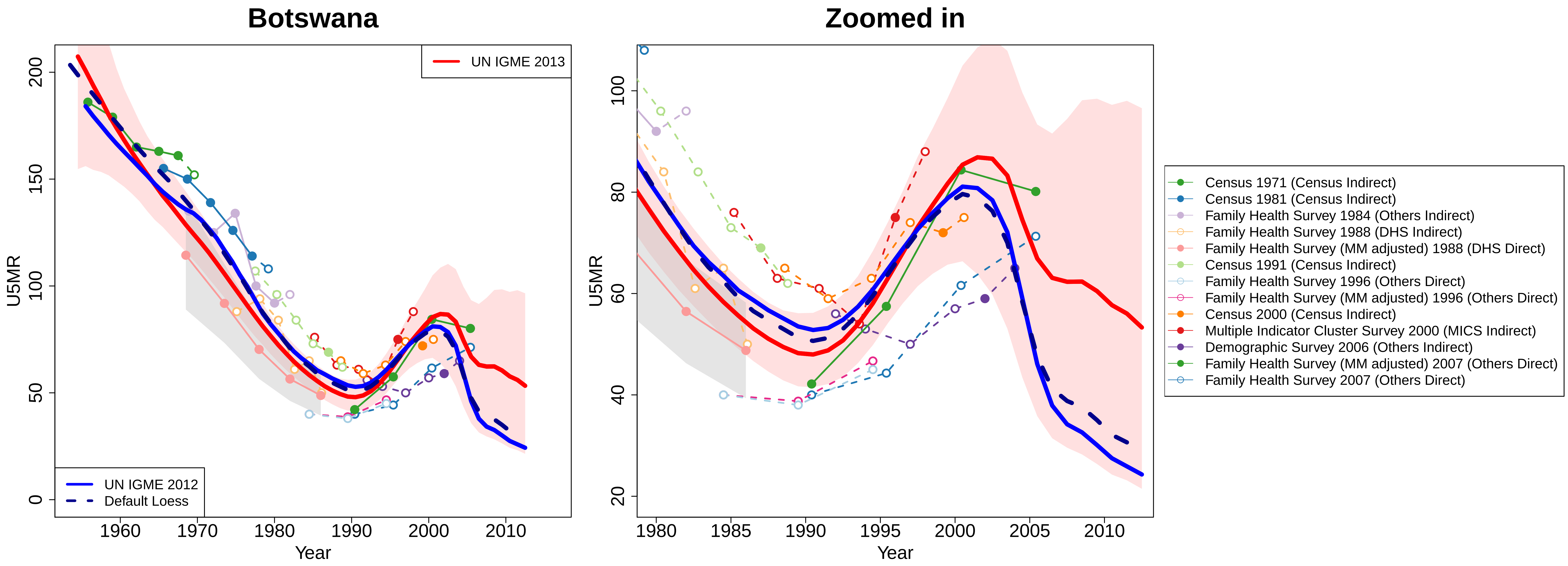

Botswana
Zoomed in
UN IGME 2013
UN IGME 2012
Default Loess
U5MR
Year
Census 1971 (Census Indirect)
Census 1981 (Census Indirect)
Family Health Survey 1984 (Others Indirect)
Family Health Survey 1988 (DHS Indirect)
Family Health Survey (MM adjusted) 1988 (DHS Direct)
Census 1991 (Census Indirect)
Family Health Survey 1996 (Others Direct)
Family Health Survey (MM adjusted) 1996 (Others Direct)
Census 2000 (Census Indirect)
Multiple Indicator Cluster Survey 2000 (MICS Indirect)
Demographic Survey 2006 (Others Indirect)
Family Health Survey (MM adjusted) 2007 (Others Direct)
Family Health Survey 2007 (Others Direct)

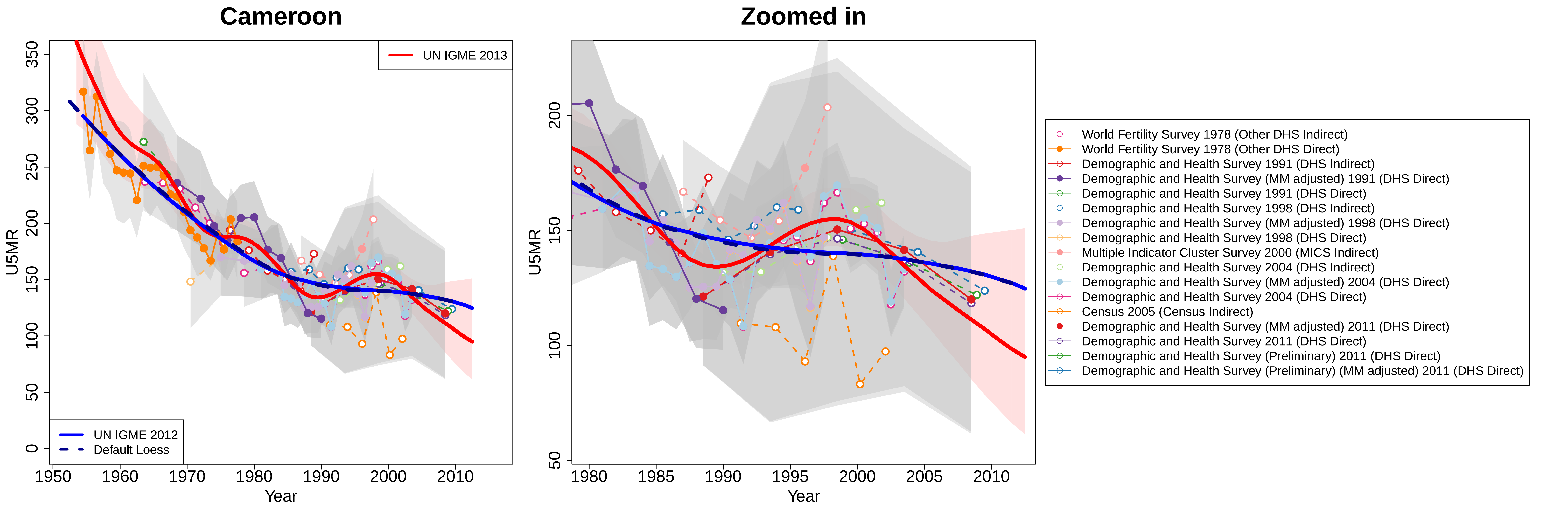
Cameroon
Zoomed in
UN IGME 2013
UN IGME 2012
Default Loess
U5MR
Year
World Fertility Survey 1978 (Other DHS Indirect)
World Fertility Survey 1978 (Other DHS Direct)
Demographic and Health Survey 1991 (DHS Indirect)
Demographic and Health Survey (MM adjusted) 1991 (DHS Direct)
Demographic and Health Survey 1991 (DHS Direct)
Demographic and Health Survey 1998 (DHS Indirect)
Demographic and Health Survey (MM adjusted) 1998 (DHS Direct)
Demographic and Health Survey 1998 (DHS Direct)
Multiple Indicator Cluster Survey 2000 (MICS Indirect)
Demographic and Health Survey 2004 (DHS Indirect)
Demographic and Health Survey (MM adjusted) 2004 (DHS Direct)
Demographic and Health Survey 2004 (DHS Direct)
Census 2005 (Census Indirect)
Demographic and Health Survey (MM adjusted) 2011 (DHS Direct)
Demographic and Health Survey 2011 (DHS Direct)
Demographic and Health Survey (Preliminary) 2011 (DHS Direct)
Demographic and Health Survey (Preliminary) (MM adjusted) 2011 (DHS Direct)

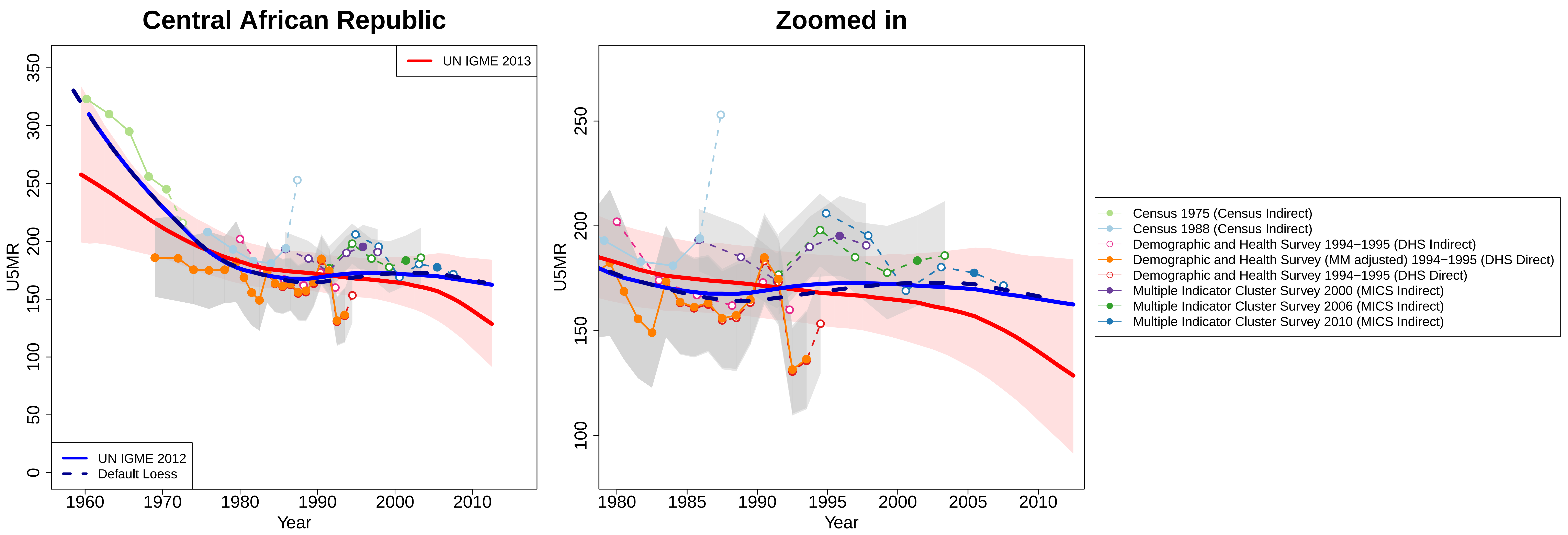
Central African Republic
Zoomed in
UN IGME 2013
UN IGME 2012
Default Loess
U5MR
Year
Census 1975 (Census Indirect)
Census 1988 (Census Indirect)
Demographic and Health Survey 1994−1995 (DHS Indirect)
Demographic and Health Survey (MM adjusted) 1994−1995 (DHS Direct)
Demographic and Health Survey 1994−1995 (DHS Direct)
Multiple Indicator Cluster Survey 2000 (MICS Indirect)
Multiple Indicator Cluster Survey 2006 (MICS Indirect)
Multiple Indicator Cluster Survey 2010 (MICS Indirect)

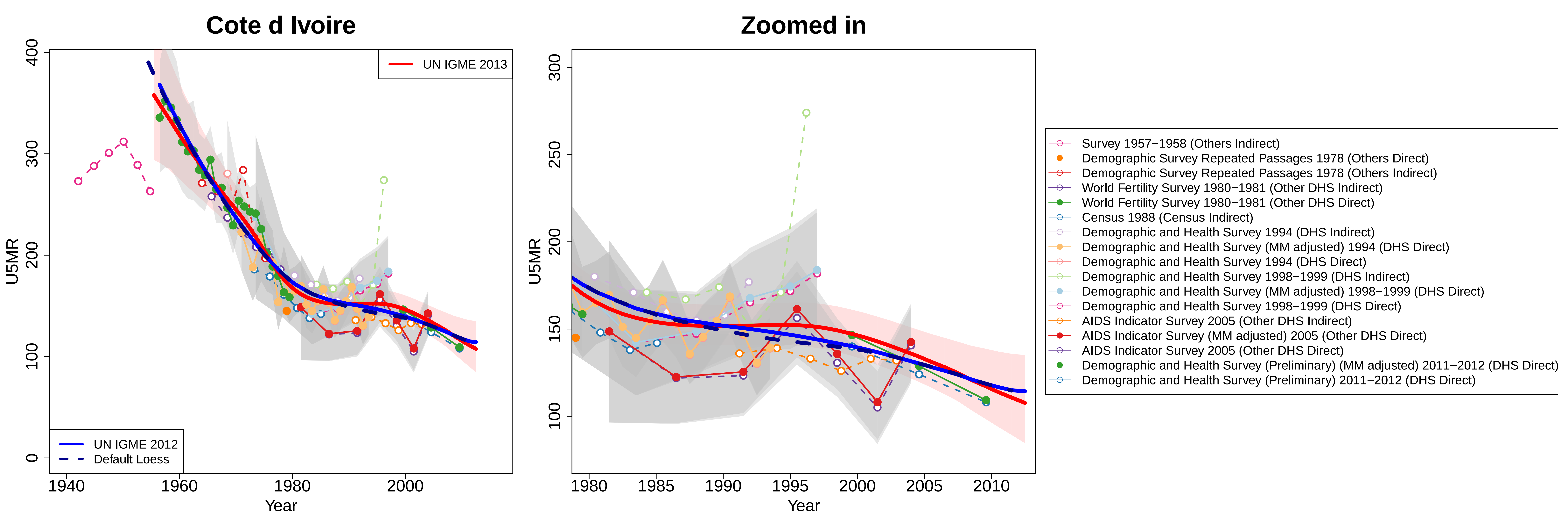
Cote d Ivoire
Zoomed in
U5MR
Year
UN IGME 2013
UN IGME 2012
Default Loess
0
100
200
300
400
1940
1960
1980
2000
150
250
1985
1990
1995
2005
2010
Survey 1957−1958 (Others Indirect)
Demographic Survey Repeated Passages 1978 (Others Direct)
Demographic Survey Repeated Passages 1978 (Others Indirect)
World Fertility Survey 1980−1981 (Other DHS Indirect)
World Fertility Survey 1980−1981 (Other DHS Direct)
Census 1988 (Census Indirect)
Demographic and Health Survey 1994 (DHS Indirect)
Demographic and Health Survey (MM adjusted) 1994 (DHS Direct)
Demographic and Health Survey 1994 (DHS Direct)
Demographic and Health Survey 1998−1999 (DHS Indirect)
Demographic and Health Survey (MM adjusted) 1998−1999 (DHS Direct)
Demographic and Health Survey 1998−1999 (DHS Direct)
AIDS Indicator Survey 2005 (Other DHS Indirect)
AIDS Indicator Survey (MM adjusted) 2005 (Other DHS Direct)
AIDS Indicator Survey 2005 (Other DHS Direct)
Demographic and Health Survey (Preliminary) (MM adjusted) 2011−2012 (DHS Direct)
Demographic and Health Survey (Preliminary) 2011−2012 (DHS Direct)

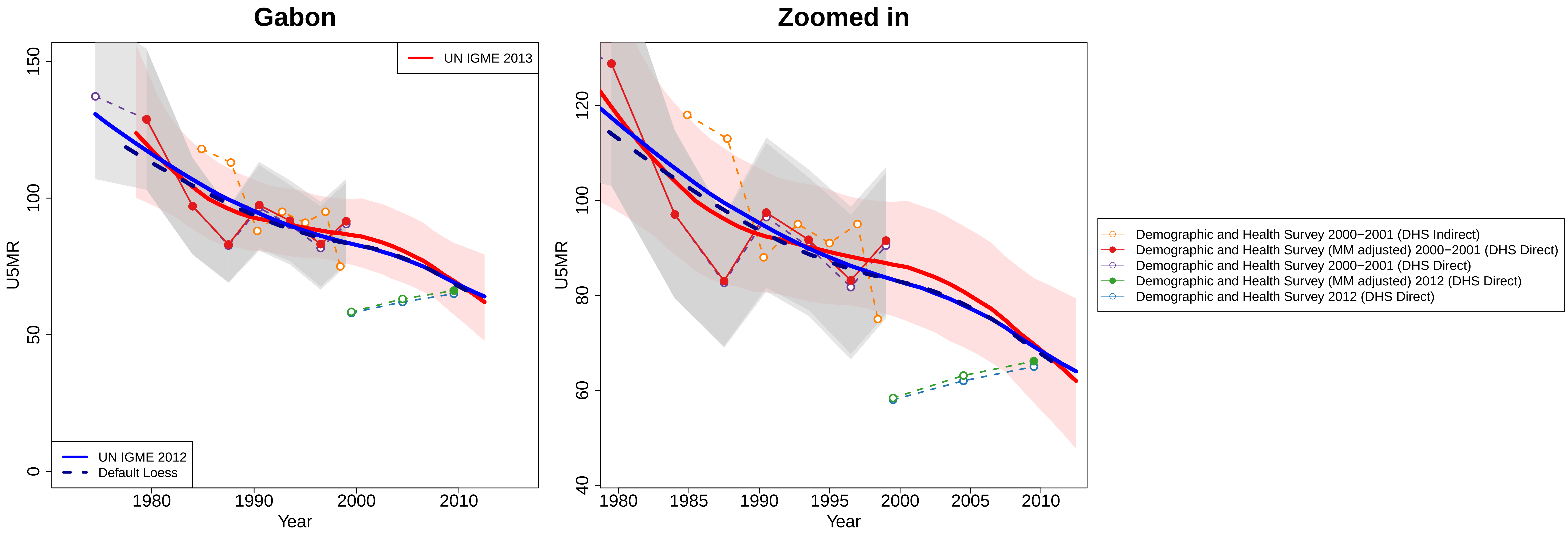

Gabon
Zoomed in
UN IGME 2013
UN IGME 2012
Default Loess
U5MR
Year
Demographic and Health Survey 2000−2001 (DHS Indirect)
Demographic and Health Survey (MM adjusted) 2000−2001 (DHS Direct)
Demographic and Health Survey 2000−2001 (DHS Direct)
Demographic and Health Survey (MM adjusted) 2012 (DHS Direct)
Demographic and Health Survey 2012 (DHS Direct)

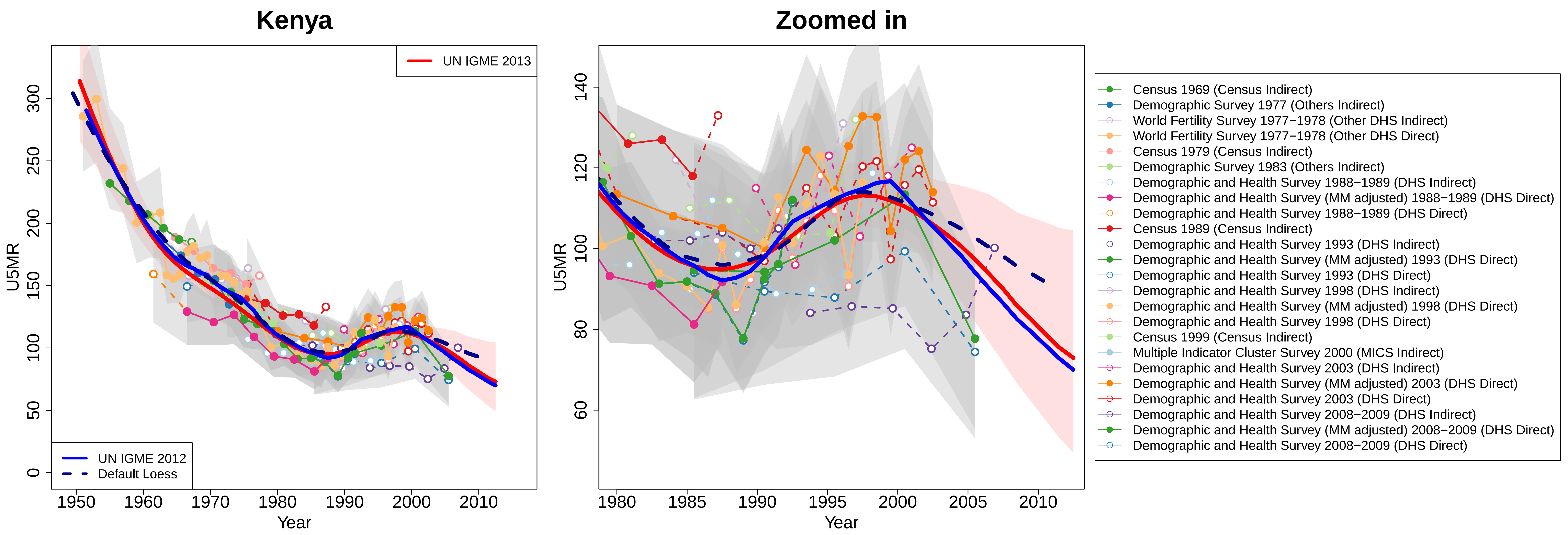
Kenya
Zoomed in
UN IGME 2013
UN IGME 2012
Default Loess
U5MR
Year
Census 1969 (Census Indirect)
Demographic Survey 1977 (Others Indirect)
World Fertility Survey 1977−1978 (Other DHS Indirect)
World Fertility Survey 1977−1978 (Other DHS Direct)
Census 1979 (Census Indirect)
Demographic Survey 1983 (Others Indirect)
Demographic and Health Survey 1988−1989 (DHS Indirect)
Demographic and Health Survey (MM adjusted) 1988−1989 (DHS Direct)
Demographic and Health Survey 1988−1989 (DHS Direct)
Census 1989 (Census Indirect)
Demographic and Health Survey 1993 (DHS Indirect)
Demographic and Health Survey (MM adjusted) 1993 (DHS Direct)
Demographic and Health Survey 1993 (DHS Direct)
Demographic and Health Survey 1998 (DHS Indirect)
Demographic and Health Survey (MM adjusted) 1998 (DHS Direct)
Demographic and Health Survey 1998 (DHS Direct)
Census 1999 (Census Indirect)
Multiple Indicator Cluster Survey 2000 (MICS Indirect)
Demographic and Health Survey 2003 (DHS Indirect)
Demographic and Health Survey (MM adjusted) 2003 (DHS Direct)
Demographic and Health Survey 2003 (DHS Direct)
Demographic and Health Survey 2008−2009 (DHS Indirect)
Demographic and Health Survey (MM adjusted) 2008−2009 (DHS Direct)
Demographic and Health Survey 2008−2009 (DHS Direct)

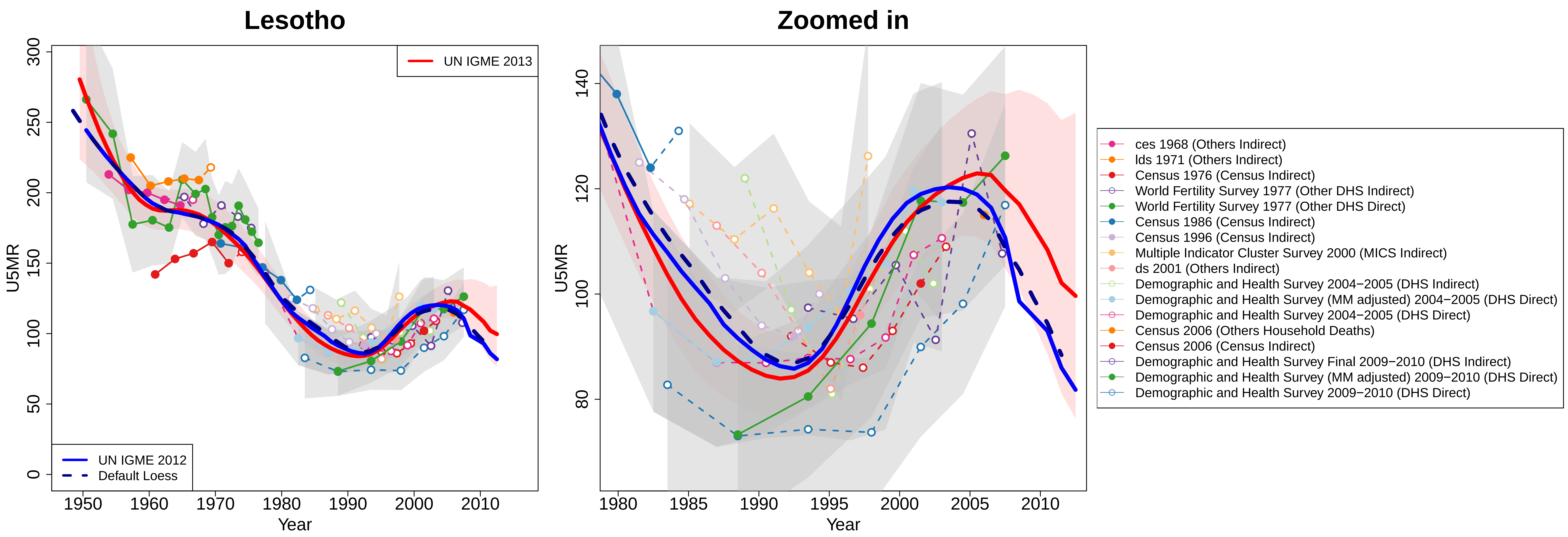

Lesotho
Zoomed in
U5MR
Year
UN IGME 2013
UN IGME 2012
Default Loess
0
50
100
150
200
250
300
1950
1960
1970
1980
1990
2000
2010
80
100
120
140
1980
1985
1990
1995
2000
2005
2010
ces 1968 (Others Indirect)
lds 1971 (Others Indirect)
Census 1976 (Census Indirect)
World Fertility Survey 1977 (Other DHS Indirect)
World Fertility Survey 1977 (Other DHS Direct)
Census 1986 (Census Indirect)
Census 1996 (Census Indirect)
Multiple Indicator Cluster Survey 2000 (MICS Indirect)
ds 2001 (Others Indirect)
Demographic and Health Survey 2004−2005 (DHS Indirect)
Demographic and Health Survey (MM adjusted) 2004−2005 (DHS Direct)
Demographic and Health Survey 2004−2005 (DHS Direct)
Census 2006 (Others Household Deaths)
Census 2006 (Census Indirect)
Demographic and Health Survey Final 2009−2010 (DHS Indirect)
Demographic and Health Survey (MM adjusted) 2009−2010 (DHS Direct)
Demographic and Health Survey 2009−2010 (DHS Direct)

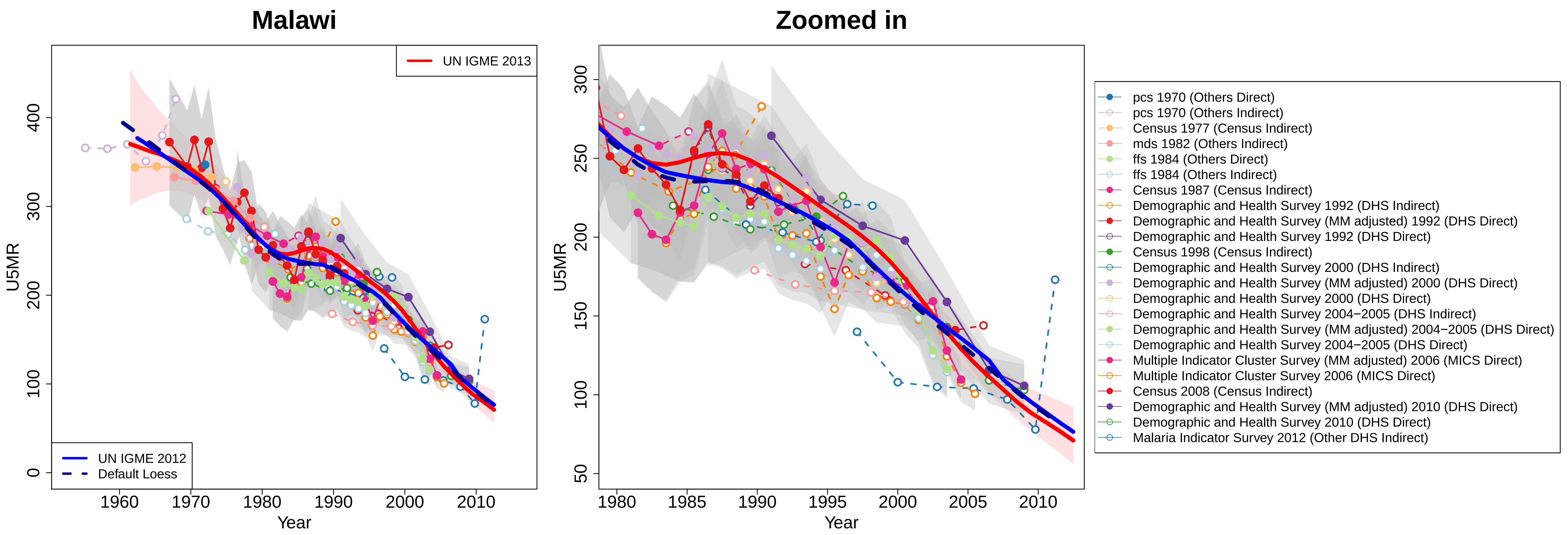

Malawi
Zoomed in
UN IGME 2013
UN IGME 2012
Default Loess
U5MR
Year
0
100
200
300
400
1960
1970
1980
1990
2000
2010
50
100
150
200
250
300
1980
1985
1990
1995
2000
2005
2010
pcs 1970 (Others Direct)
pcs 1970 (Others Indirect)
Census 1977 (Census Indirect)
mds 1982 (Others Indirect)
ffs 1984 (Others Direct)
ffs 1984 (Others Indirect)
Census 1987 (Census Indirect)
Demographic and Health Survey 1992 (DHS Indirect)
Demographic and Health Survey (MM adjusted) 1992 (DHS Direct)
Demographic and Health Survey 1992 (DHS Direct)
Census 1998 (Census Indirect)
Demographic and Health Survey 2000 (DHS Indirect)
Demographic and Health Survey (MM adjusted) 2000 (DHS Direct)
Demographic and Health Survey 2000 (DHS Direct)
Demographic and Health Survey 2004−2005 (DHS Indirect)
Demographic and Health Survey (MM adjusted) 2004−2005 (DHS Direct)
Demographic and Health Survey 2004−2005 (DHS Direct)
Multiple Indicator Cluster Survey (MM adjusted) 2006 (MICS Direct)
Multiple Indicator Cluster Survey 2006 (MICS Direct)
Census 2008 (Census Indirect)
Demographic and Health Survey (MM adjusted) 2010 (DHS Direct)
Demographic and Health Survey 2010 (DHS Direct)
Malaria Indicator Survey 2012 (Other DHS Indirect)

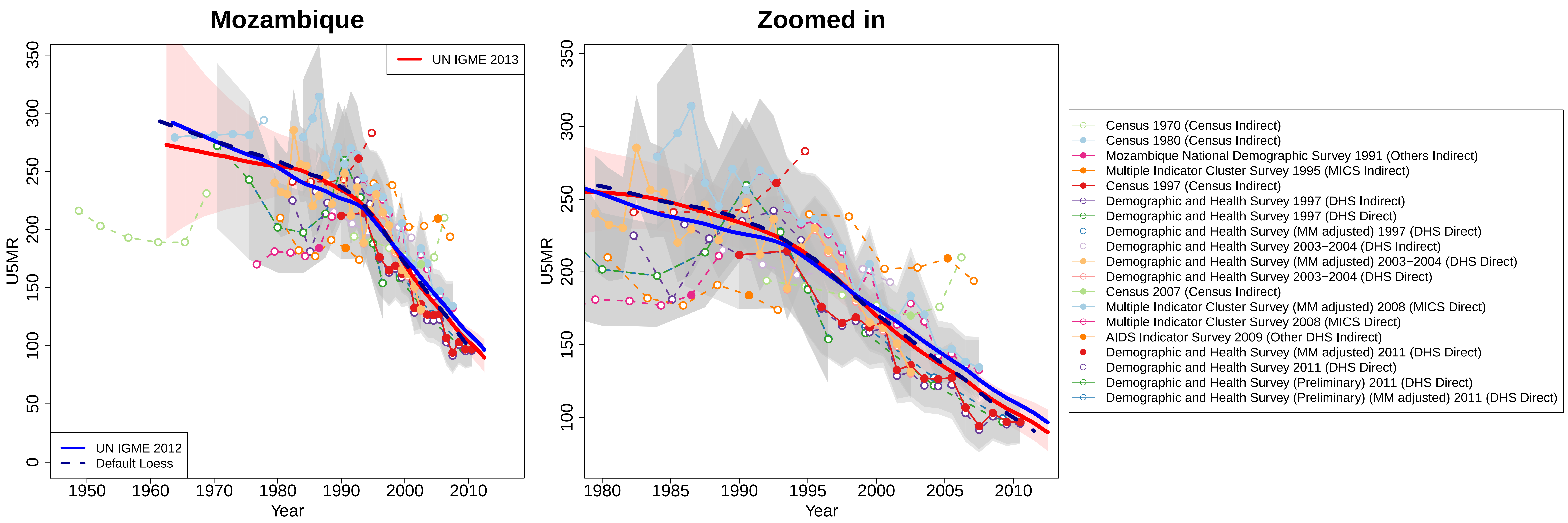

Mozambique
Zoomed in
UN IGME 2013
UN IGME 2012
Default Loess
U5MR
Year
Census 1970 (Census Indirect)
Census 1980 (Census Indirect)
Mozambique National Demographic Survey 1991 (Others Indirect)
Multiple Indicator Cluster Survey 1995 (MICS Indirect)
Census 1997 (Census Indirect)
Demographic and Health Survey 1997 (DHS Indirect)
Demographic and Health Survey 1997 (DHS Direct)
Demographic and Health Survey (MM adjusted) 1997 (DHS Direct)
Demographic and Health Survey 2003−2004 (DHS Indirect)
Demographic and Health Survey (MM adjusted) 2003−2004 (DHS Direct)
Demographic and Health Survey 2003−2004 (DHS Direct)
Census 2007 (Census Indirect)
Multiple Indicator Cluster Survey (MM adjusted) 2008 (MICS Direct)
Multiple Indicator Cluster Survey 2008 (MICS Direct)
AIDS Indicator Survey 2009 (Other DHS Indirect)
Demographic and Health Survey (MM adjusted) 2011 (DHS Direct)
Demographic and Health Survey 2011 (DHS Direct)
Demographic and Health Survey (Preliminary) 2011 (DHS Direct)
Demographic and Health Survey (Preliminary) (MM adjusted) 2011 (DHS Direct)

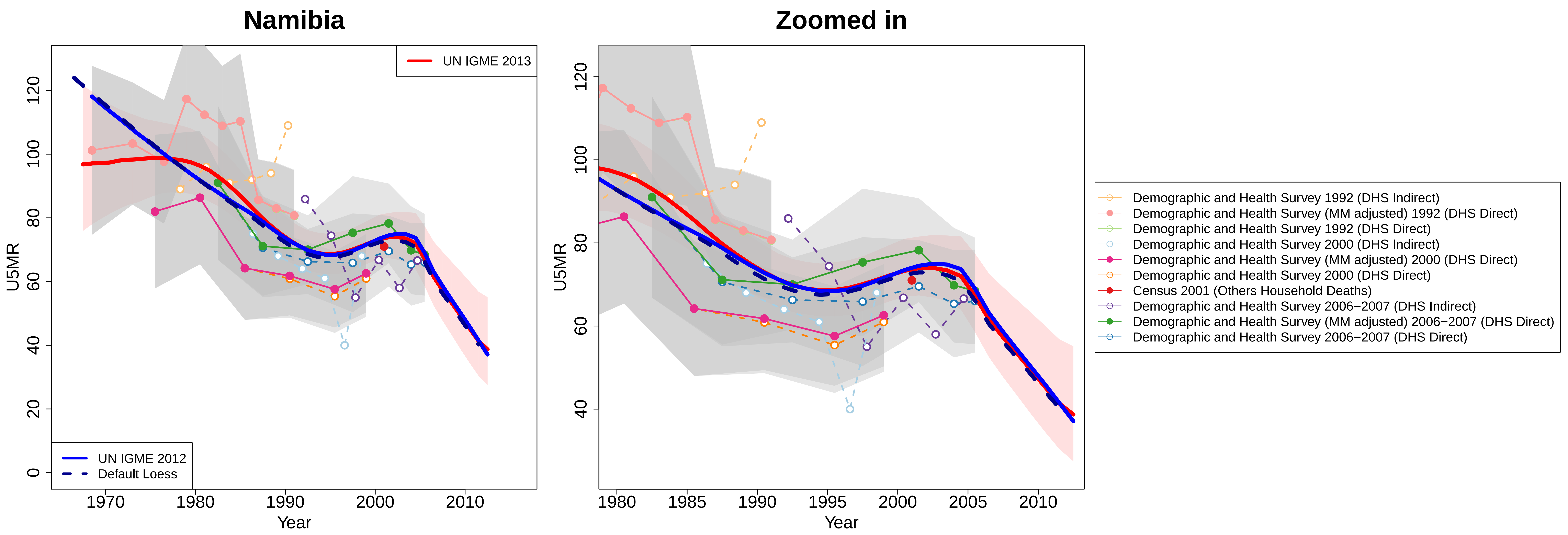
Namibia
Zoomed in
UN IGME 2013
UN IGME 2012
Default Loess
U5MR
Year
Demographic and Health Survey 1992 (DHS Indirect)
Demographic and Health Survey (MM adjusted) 1992 (DHS Direct)
Demographic and Health Survey 1992 (DHS Direct)
Demographic and Health Survey 2000 (DHS Indirect)
Demographic and Health Survey (MM adjusted) 2000 (DHS Direct)
Demographic and Health Survey 2000 (DHS Direct)
Census 2001 (Others Household Deaths)
Demographic and Health Survey 2006−2007 (DHS Indirect)
Demographic and Health Survey (MM adjusted) 2006−2007 (DHS Direct)
Demographic and Health Survey 2006−2007 (DHS Direct)

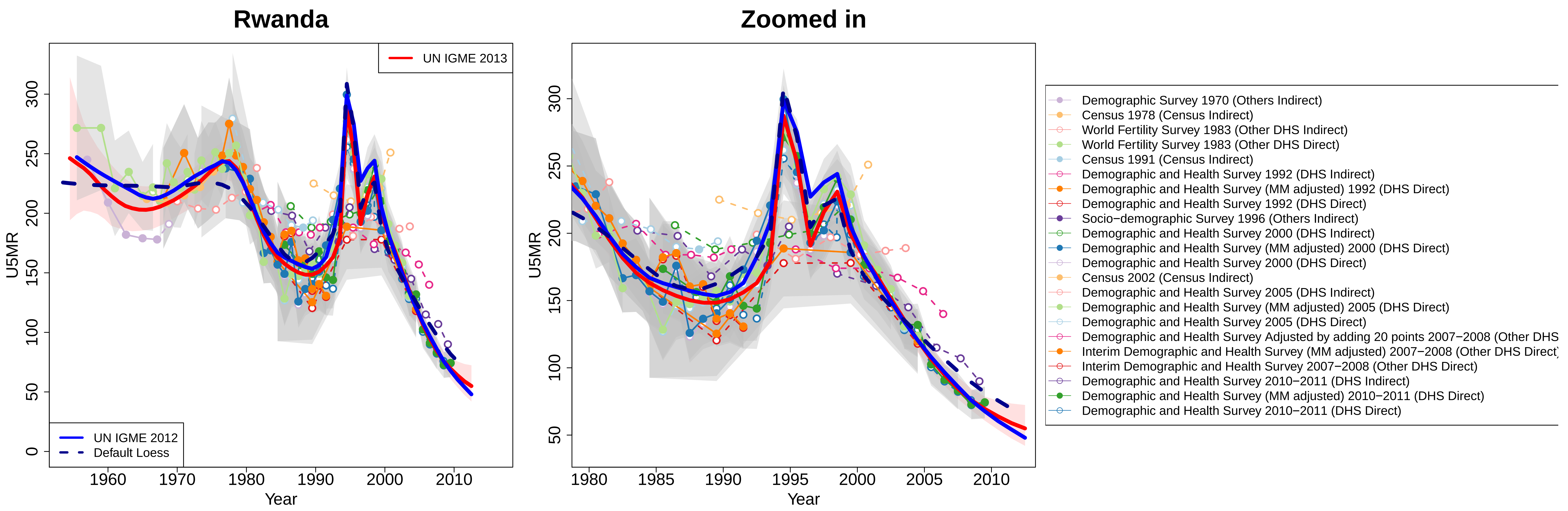

Rwanda
Zoomed in
UN IGME 2013
UN IGME 2012
Default Loess
U5MR
Year
Demographic Survey 1970 (Others Indirect)
Census 1978 (Census Indirect)
World Fertility Survey 1983 (Other DHS Indirect)
World Fertility Survey 1983 (Other DHS Direct)
Census 1991 (Census Indirect)
Demographic and Health Survey 1992 (DHS Indirect)
Demographic and Health Survey (MM adjusted) 1992 (DHS Direct)
Demographic and Health Survey 1992 (DHS Direct)
Socio−demographic Survey 1996 (Others Indirect)
Demographic and Health Survey 2000 (DHS Indirect)
Demographic and Health Survey (MM adjusted) 2000 (DHS Direct)
Demographic and Health Survey 2000 (DHS Direct)
Census 2002 (Census Indirect)
Demographic and Health Survey 2005 (DHS Indirect)
Demographic and Health Survey (MM adjusted) 2005 (DHS Direct)
Demographic and Health Survey 2005 (DHS Direct)
Demographic and Health Survey Adjusted by adding 20 points 2007−2008 (Other DHS
Interim Demographic and Health Survey (MM adjusted) 2007−2008 (Other DHS Direct)
Interim Demographic and Health Survey 2007−2008 (Other DHS Direct)
Demographic and Health Survey 2010−2011 (DHS Indirect)
Demographic and Health Survey (MM adjusted) 2010−2011 (DHS Direct)
Demographic and Health Survey 2010−2011 (DHS Direct)

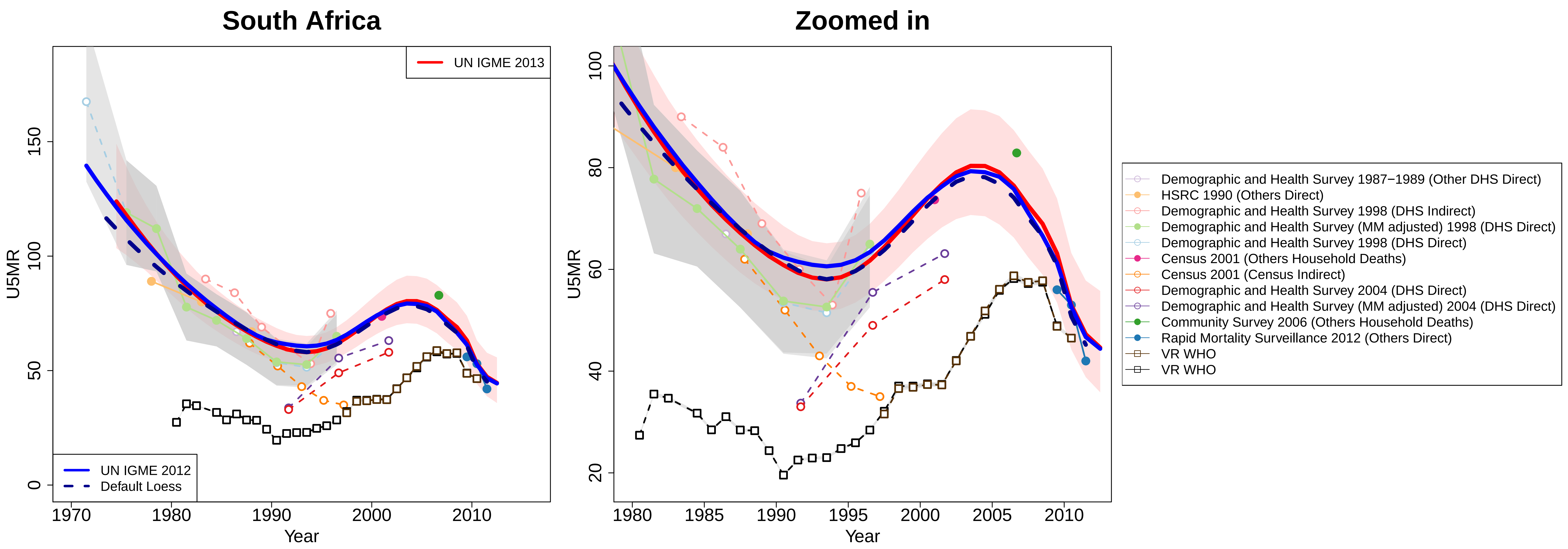

South Africa
Zoomed in
UN IGME 2013
UN IGME 2012
Default Loess
U5MR
Year
0
50
100
150
1970
1980
1990
2000
2010
20
40
60
80
100
1985
1995
2005
Demographic and Health Survey 1987−1989 (Other DHS Direct)
HSRC 1990 (Others Direct)
Demographic and Health Survey 1998 (DHS Indirect)
Demographic and Health Survey (MM adjusted) 1998 (DHS Direct)
Demographic and Health Survey 1998 (DHS Direct)
Census 2001 (Others Household Deaths)
Census 2001 (Census Indirect)
Demographic and Health Survey 2004 (DHS Direct)
Demographic and Health Survey (MM adjusted) 2004 (DHS Direct)
Community Survey 2006 (Others Household Deaths)
Rapid Mortality Surveillance 2012 (Others Direct)
VR WHO
VR WHO

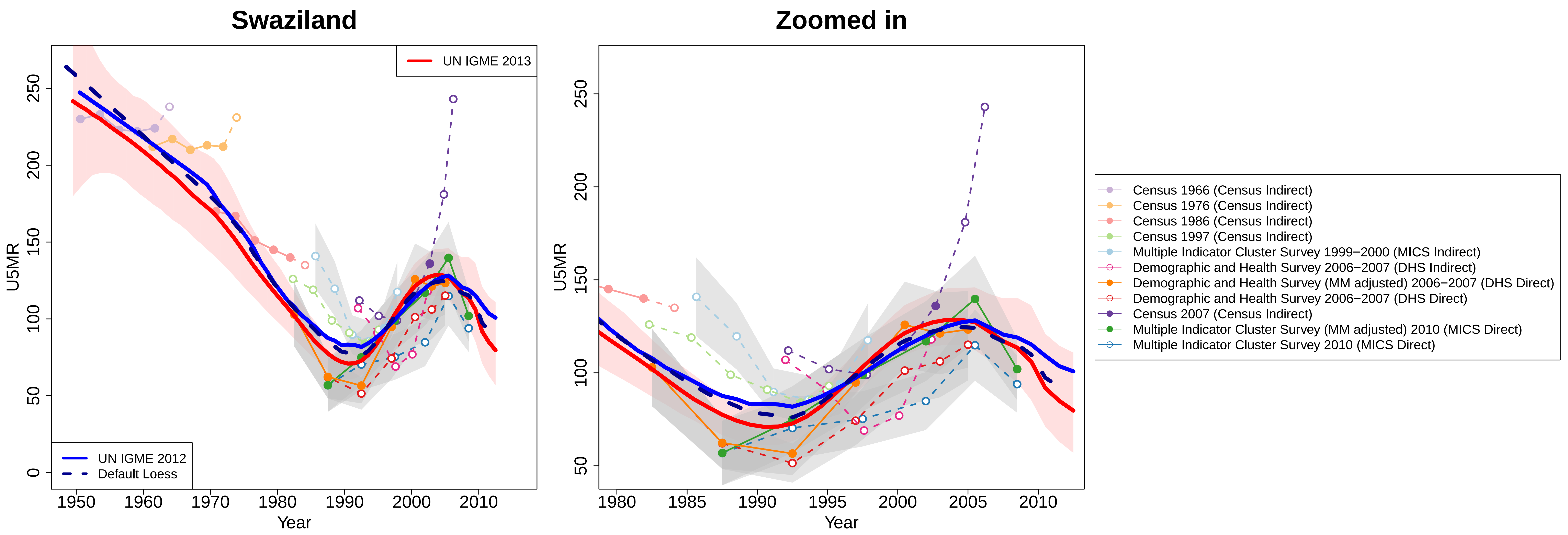
Swaziland
Zoomed in
UN IGME 2013
UN IGME 2012
Default Loess
U5MR
Year
Census 1966 (Census Indirect)
Census 1976 (Census Indirect)
Census 1986 (Census Indirect)
Census 1997 (Census Indirect)
Multiple Indicator Cluster Survey 1999−2000 (MICS Indirect)
Demographic and Health Survey 2006−2007 (DHS Indirect)
Demographic and Health Survey (MM adjusted) 2006−2007 (DHS Direct)
Demographic and Health Survey 2006−2007 (DHS Direct)
Census 2007 (Census Indirect)
Multiple Indicator Cluster Survey (MM adjusted) 2010 (MICS Direct)
Multiple Indicator Cluster Survey 2010 (MICS Direct)

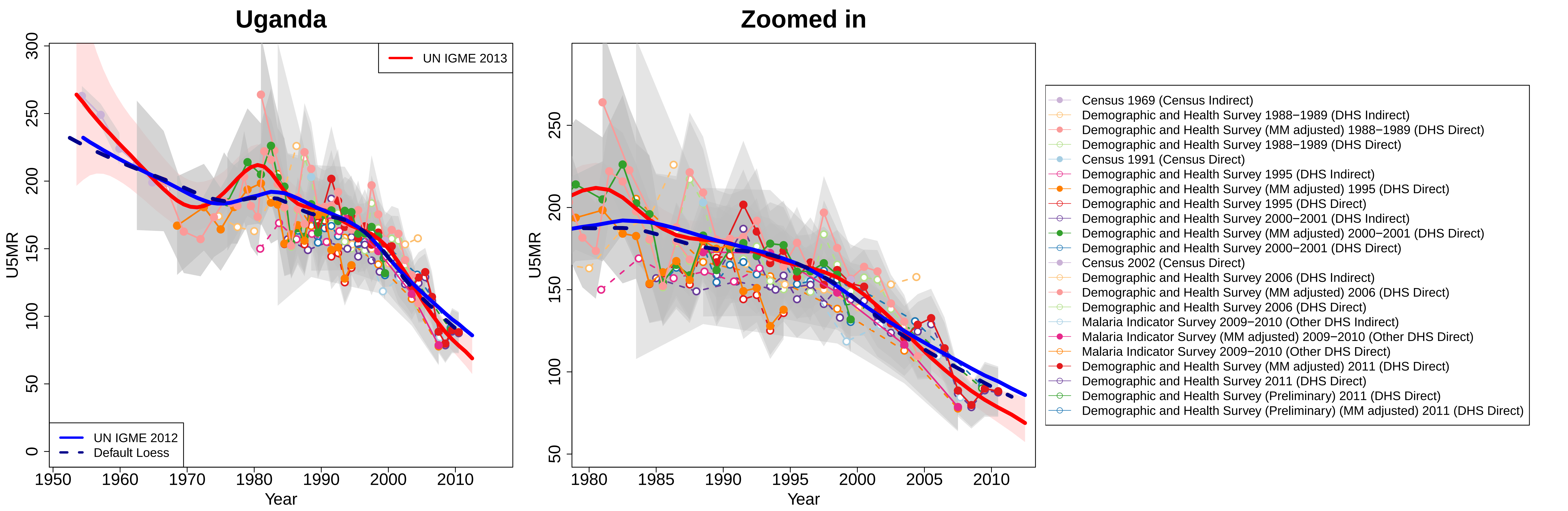
Uganda
Zoomed in
UN IGME 2013
UN IGME 2012
Default Loess
U5MR
Year
Census 1969 (Census Indirect)
Demographic and Health Survey 1988−1989 (DHS Indirect)
Demographic and Health Survey (MM adjusted) 1988−1989 (DHS Direct)
Demographic and Health Survey 1988−1989 (DHS Direct)
Census 1991 (Census Direct)
Demographic and Health Survey 1995 (DHS Indirect)
Demographic and Health Survey (MM adjusted) 1995 (DHS Direct)
Demographic and Health Survey 1995 (DHS Direct)
Demographic and Health Survey 2000−2001 (DHS Indirect)
Demographic and Health Survey (MM adjusted) 2000−2001 (DHS Direct)
Demographic and Health Survey 2000−2001 (DHS Direct)
Census 2002 (Census Direct)
Demographic and Health Survey 2006 (DHS Indirect)
Demographic and Health Survey (MM adjusted) 2006 (DHS Direct)
Demographic and Health Survey 2006 (DHS Direct)
Malaria Indicator Survey 2009−2010 (Other DHS Indirect)
Malaria Indicator Survey (MM adjusted) 2009−2010 (Other DHS Direct)
Malaria Indicator Survey 2009−2010 (Other DHS Direct)
Demographic and Health Survey (MM adjusted) 2011 (DHS Direct)
Demographic and Health Survey 2011 (DHS Direct)
Demographic and Health Survey (Preliminary) 2011 (DHS Direct)
Demographic and Health Survey (Preliminary) (MM adjusted) 2011 (DHS Direct)

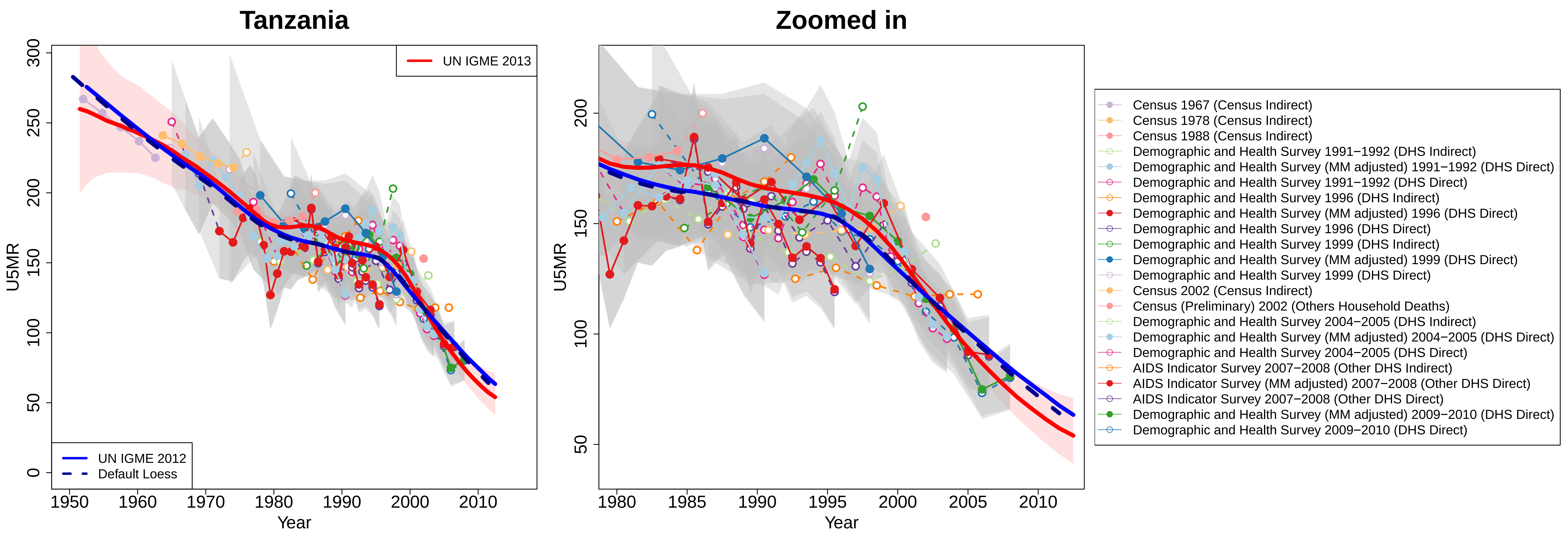

Tanzania
Zoomed in
U5MR
Year
UN IGME 2013
UN IGME 2012
Default Loess
0
50
100
150
200
250
300
1950
1960
1970
1980
1990
2000
2010
1980
1985
1990
1995
2000
2005
2010
Census 1967 (Census Indirect)
Census 1978 (Census Indirect)
Census 1988 (Census Indirect)
Demographic and Health Survey 1991−1992 (DHS Indirect)
Demographic and Health Survey (MM adjusted) 1991−1992 (DHS Direct)
Demographic and Health Survey 1991−1992 (DHS Direct)
Demographic and Health Survey 1996 (DHS Indirect)
Demographic and Health Survey (MM adjusted) 1996 (DHS Direct)
Demographic and Health Survey 1996 (DHS Direct)
Demographic and Health Survey 1999 (DHS Indirect)
Demographic and Health Survey (MM adjusted) 1999 (DHS Direct)
Demographic and Health Survey 1999 (DHS Direct)
Census 2002 (Census Indirect)
Census (Preliminary) 2002 (Others Household Deaths)
Demographic and Health Survey 2004−2005 (DHS Indirect)
Demographic and Health Survey (MM adjusted) 2004−2005 (DHS Direct)
Demographic and Health Survey 2004−2005 (DHS Direct)
AIDS Indicator Survey 2007−2008 (Other DHS Indirect)
AIDS Indicator Survey (MM adjusted) 2007−2008 (Other DHS Direct)
AIDS Indicator Survey 2007−2008 (Other DHS Direct)
Demographic and Health Survey (MM adjusted) 2009−2010 (DHS Direct)
Demographic and Health Survey 2009−2010 (DHS Direct)

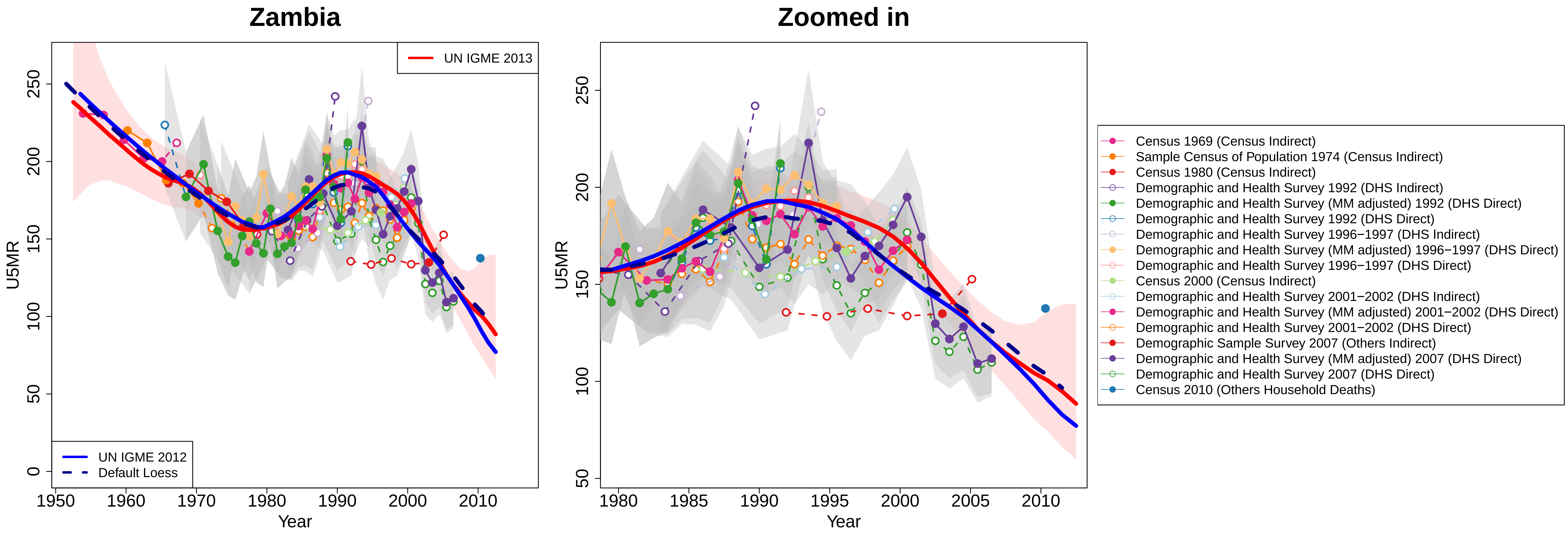

Zambia
Zoomed in
U5MR
Year
UN IGME 2013
UN IGME 2012
Default Loess
Census 1969 (Census Indirect)
Sample Census of Population 1974 (Census Indirect)
Census 1980 (Census Indirect)
Demographic and Health Survey 1992 (DHS Indirect)
Demographic and Health Survey (MM adjusted) 1992 (DHS Direct)
Demographic and Health Survey 1992 (DHS Direct)
Demographic and Health Survey 1996−1997 (DHS Indirect)
Demographic and Health Survey (MM adjusted) 1996−1997 (DHS Direct)
Demographic and Health Survey 1996−1997 (DHS Direct)
Census 2000 (Census Indirect)
Demographic and Health Survey 2001−2002 (DHS Indirect)
Demographic and Health Survey (MM adjusted) 2001−2002 (DHS Direct)
Demographic and Health Survey 2001−2002 (DHS Direct)
Demographic Sample Survey 2007 (Others Indirect)
Demographic and Health Survey (MM adjusted) 2007 (DHS Direct)
Demographic and Health Survey 2007 (DHS Direct)
Census 2010 (Others Household Deaths)

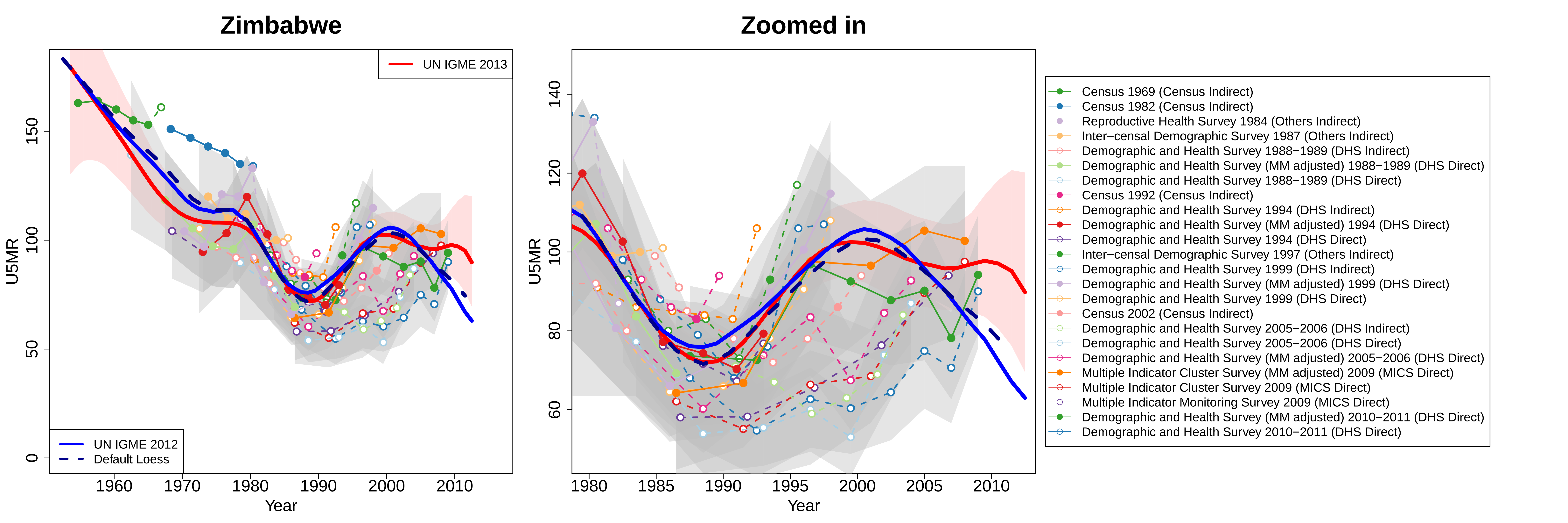
Zimbabwe
Zoomed in
UN IGME 2013
UN IGME 2012
Default Loess
U5MR
Year
Census 1969 (Census Indirect)
Census 1982 (Census Indirect)
Reproductive Health Survey 1984 (Others Indirect)
Inter−censal Demographic Survey 1987 (Others Indirect)
Demographic and Health Survey 1988−1989 (DHS Indirect)
Demographic and Health Survey (MM adjusted) 1988−1989 (DHS Direct)
Demographic and Health Survey 1988−1989 (DHS Direct)
Census 1992 (Census Indirect)
Demographic and Health Survey 1994 (DHS Indirect)
Demographic and Health Survey (MM adjusted) 1994 (DHS Direct)
Demographic and Health Survey 1994 (DHS Direct)
Inter−censal Demographic Survey 1997 (Others Indirect)
Demographic and Health Survey 1999 (DHS Indirect)
Demographic and Health Survey (MM adjusted) 1999 (DHS Direct)
Demographic and Health Survey 1999 (DHS Direct)
Census 2002 (Census Indirect)
Demographic and Health Survey 2005−2006 (DHS Indirect)
Demographic and Health Survey 2005−2006 (DHS Direct)
Demographic and Health Survey (MM adjusted) 2005−2006 (DHS Direct)
Multiple Indicator Cluster Survey (MM adjusted) 2009 (MICS Direct)
Multiple Indicator Cluster Survey 2009 (MICS Direct)
Multiple Indicator Monitoring Survey 2009 (MICS Direct)
Demographic and Health Survey (MM adjusted) 2010−2011 (DHS Direct)
Demographic and Health Survey 2010−2011 (DHS Direct)

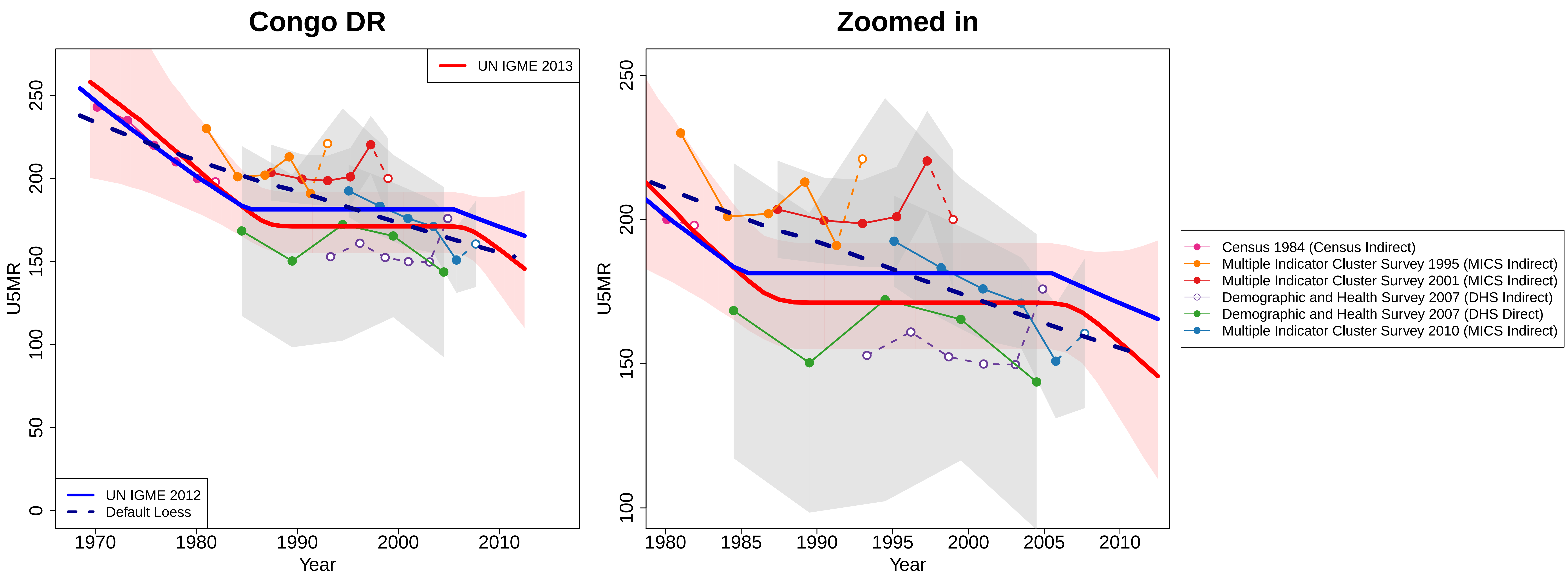

Congo DR
Zoomed in
UN IGME 2013
UN IGME 2012
Default Loess
U5MR
Year
0
50
100
150
200
250
1970
1980
1990
2000
2010
1985
1995
2005
Census 1984 (Census Indirect)
Multiple Indicator Cluster Survey 1995 (MICS Indirect)
Multiple Indicator Cluster Survey 2001 (MICS Indirect)
Demographic and Health Survey 2007 (DHS Indirect)
Demographic and Health Survey 2007 (DHS Direct)
Multiple Indicator Cluster Survey 2010 (MICS Indirect)

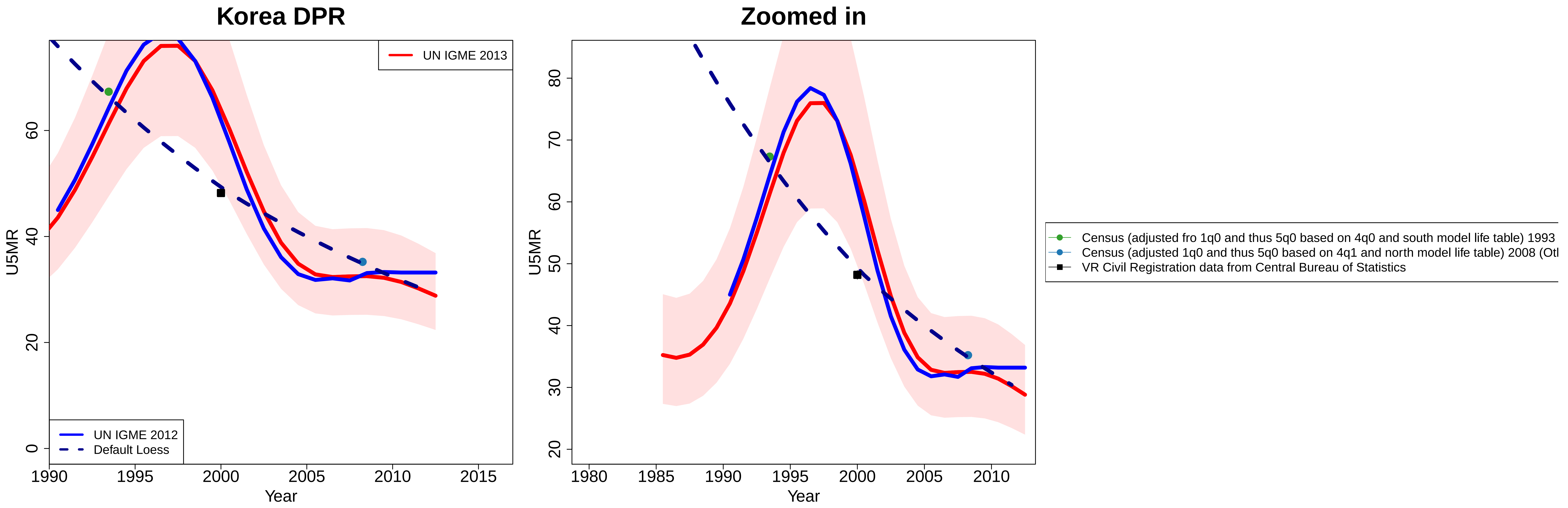
Korea DPR
UN IGME 2013
UN IGME 2012
Default Loess
U5MR
Year
0
20
40
60
1990
1995
2000
2005
2010
2015
Zoomed in
U5MR
Year
20
30
40
50
60
70
80
1980
1985
1990
1995
2000
2005
2010
Census (adjusted fro 1q0 and thus 5q0 based on 4q0 and south model life table) 1993
Census (adjusted 1q0 and thus 5q0 based on 4q1 and north model life table) 2008 (Otl
VR Civil Registration data from Central Bureau of Statistics

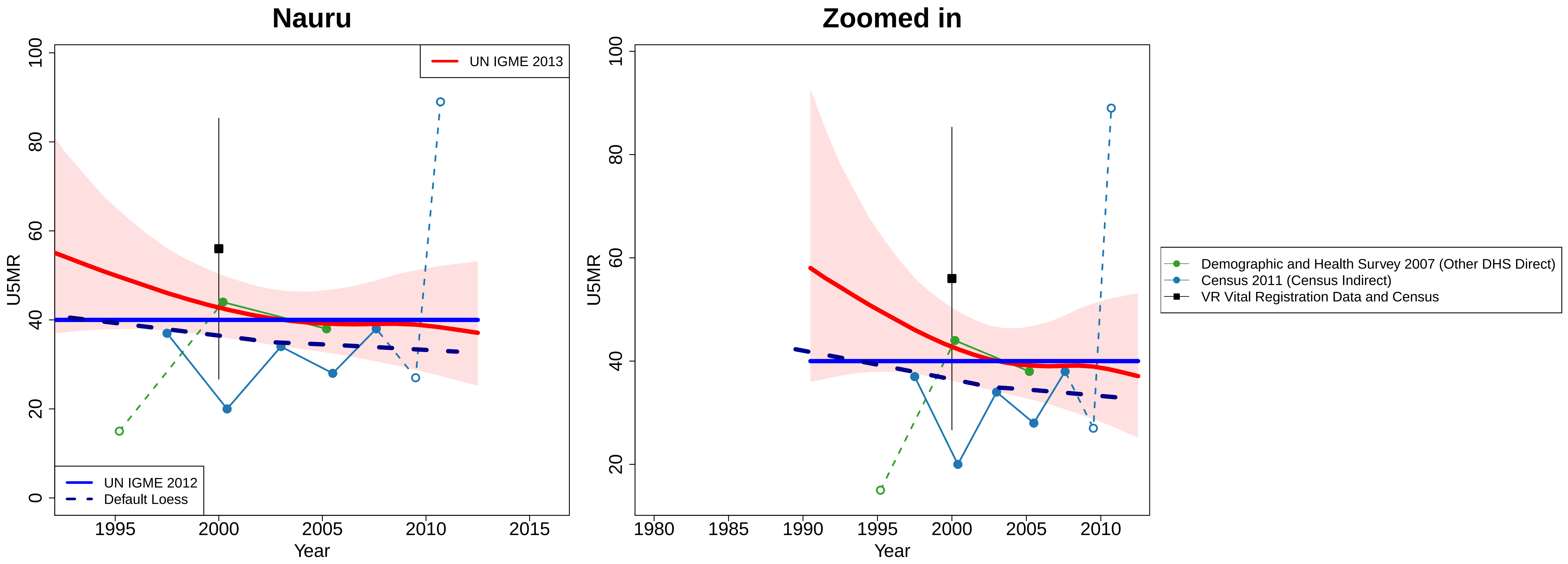

Nauru
Zoomed in
U5MR
Year
UN IGME 2013
UN IGME 2012
Default Loess
Demographic and Health Survey 2007 (Other DHS Direct)
Census 2011 (Census Indirect)
VR Vital Registration Data and Census
0
20
40
60
80
100
1980
1985
1990
1995
2000
2005
2010
2015

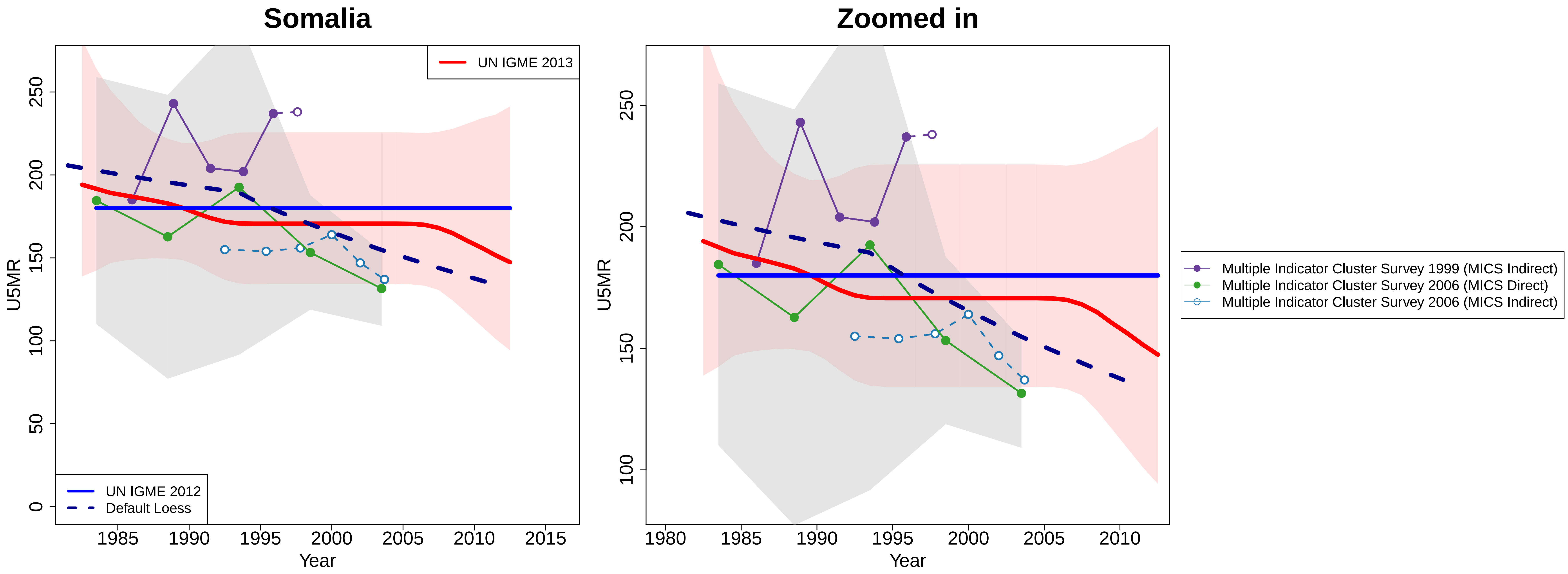
Somalia
Zoomed in
U5MR
Year
UN IGME 2013
UN IGME 2012
Default Loess
Multiple Indicator Cluster Survey 1999 (MICS Indirect)
Multiple Indicator Cluster Survey 2006 (MICS Direct)
Multiple Indicator Cluster Survey 2006 (MICS Indirect)
0
50
100
150
200
250
1980
1985
1990
1995
2000
2005
2010
2015
