# Supplementary material for: Child Mortality Estimation 2013: An Overview of Updates in Estimation Methods by the United Nations Inter-Agency Group for Child Mortality Estimation
Source: PLoS One. 2014 Jul 11;9(7):e101112. doi: 10.1371/journal.pone.0101112 (PMC4094389; doi:10.1371/journal.pone.0101112)
Supplement: Figure S2 — U5MR estimates for 33 countries where the difference in estimation method between UN IGME 2012 and UN IGME 2013 led to an absolute difference in U5MR estimate for the year 1990 or 2011 of more than 10 deaths per 1,000 births. The default loess fit to the 2013 UN IGME database is given by the dashed dark blue line, the B2 method (B3 method for curve fitting but treating all observations equally) fit is shown by the solid light green line (with 90% credible intervals given by the shaded regions) and UN IGME 2013 estimates are given in red by the solid red line (with 90% uncertainty intervals denoted by the shaded regions). Connected dots denote data from the UN IGME 2013 database and gray shaded areas around series of observations represent the sampling variability in the series (quantified by two times of the sampling standard errors). (PDF) [file pone.0101112.s002.pdf]

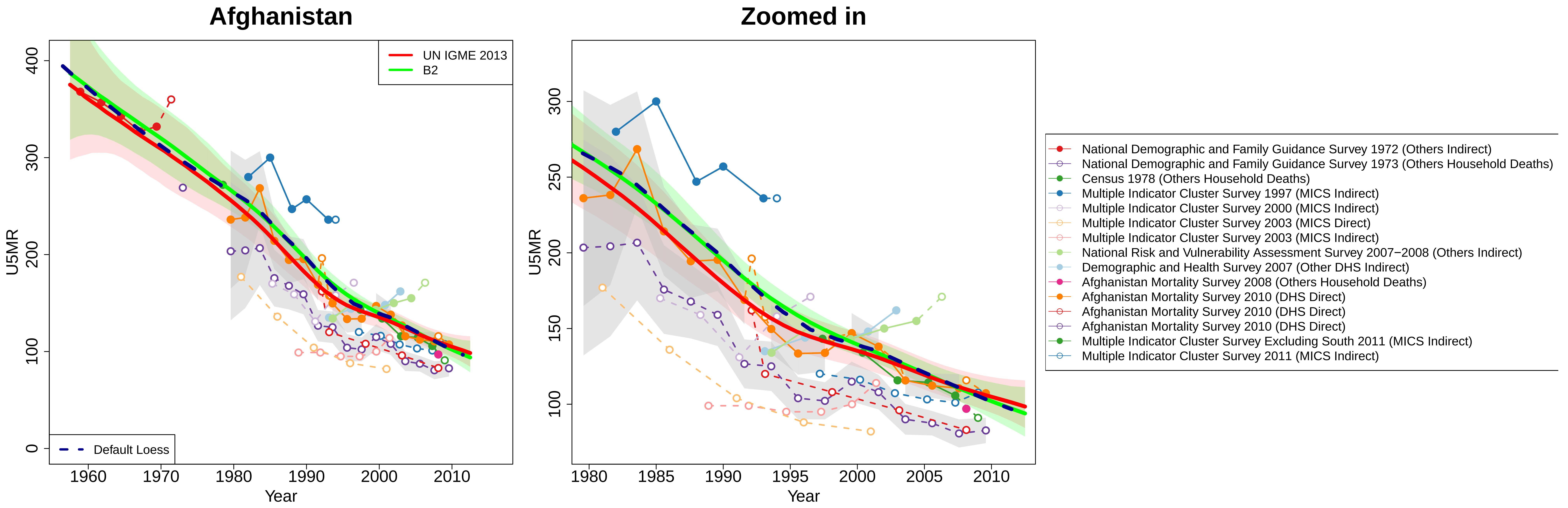

Afghanistan
Zoomed in
UN IGME 2013
B2
Default Loess
U5MR
Year
National Demographic and Family Guidance Survey 1972 (Others Indirect)
National Demographic and Family Guidance Survey 1973 (Others Household Deaths)
Census 1978 (Others Household Deaths)
Multiple Indicator Cluster Survey 1997 (MICS Indirect)
Multiple Indicator Cluster Survey 2000 (MICS Indirect)
Multiple Indicator Cluster Survey 2003 (MICS Direct)
Multiple Indicator Cluster Survey 2003 (MICS Indirect)
National Risk and Vulnerability Assessment Survey 2007−2008 (Others Indirect)
Demographic and Health Survey 2007 (Other DHS Indirect)
Afghanistan Mortality Survey 2008 (Others Household Deaths)
Afghanistan Mortality Survey 2010 (DHS Direct)
Afghanistan Mortality Survey 2010 (DHS Direct)
Afghanistan Mortality Survey 2010 (DHS Direct)
Multiple Indicator Cluster Survey Excluding South 2011 (MICS Indirect)
Multiple Indicator Cluster Survey 2011 (MICS Indirect)

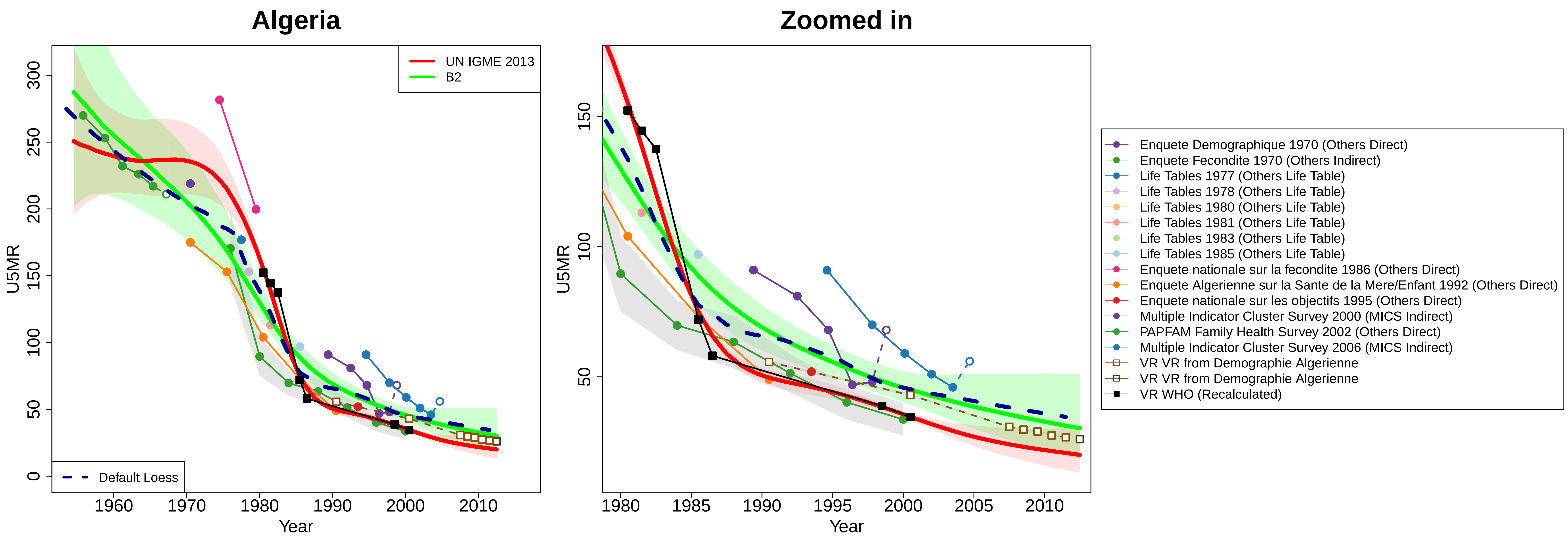
Algeria
Zoomed in
UN IGME 2013
B2
Default Loess
U5MR
Year
Enquete Demographique 1970 (Others Direct)
Enquete Fecondite 1970 (Others Indirect)
Life Tables 1977 (Others Life Table)
Life Tables 1978 (Others Life Table)
Life Tables 1980 (Others Life Table)
Life Tables 1981 (Others Life Table)
Life Tables 1983 (Others Life Table)
Life Tables 1985 (Others Life Table)
Enquete nationale sur la fecondite 1986 (Others Direct)
Enquete Algerienne sur la Sante de la Mere/Enfant 1992 (Others Direct)
Enquete nationale sur les objectifs 1995 (Others Direct)
Multiple Indicator Cluster Survey 2000 (MICS Indirect)
PAPFAM Family Health Survey 2002 (Others Direct)
Multiple Indicator Cluster Survey 2006 (MICS Indirect)
VR VR from Demographie Algerienne
VR VR from Demographie Algerienne
VR WHO (Recalculated)

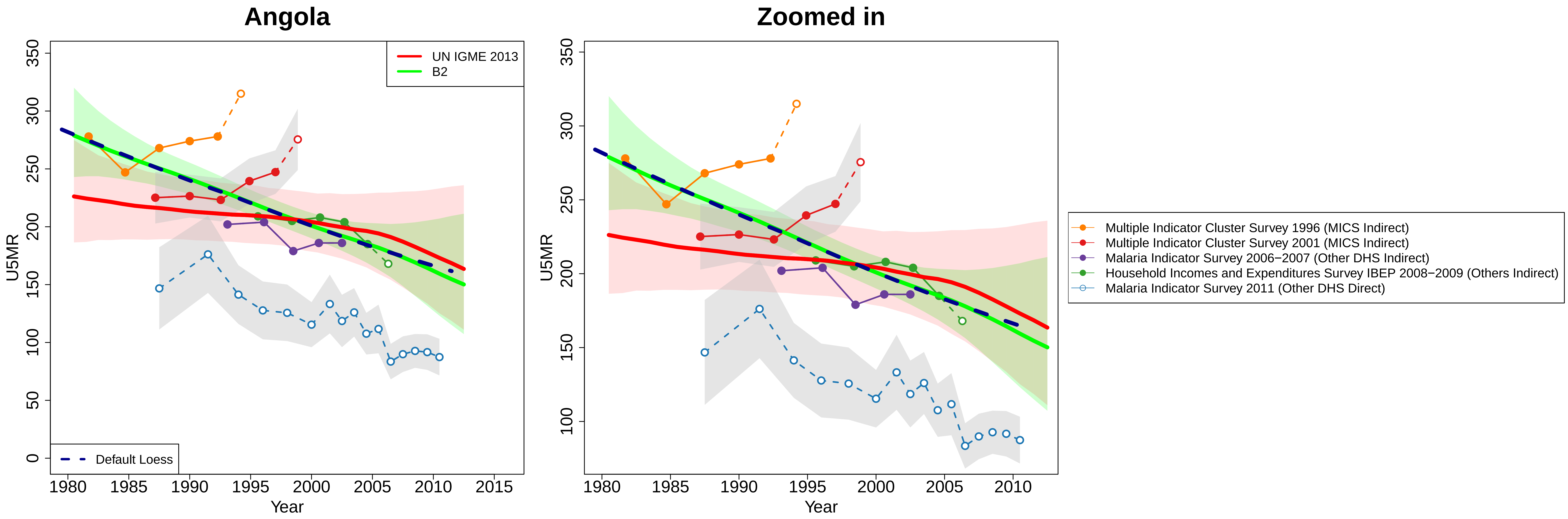
Angola
Zoomed in
UN IGME 2013
B2
Default Loess
U5MR
Year
Multiple Indicator Cluster Survey 1996 (MICS Indirect)
Multiple Indicator Cluster Survey 2001 (MICS Indirect)
Malaria Indicator Survey 2006−2007 (Other DHS Indirect)
Household Incomes and Expenditures Survey IBEP 2008−2009 (Others Indirect)
Malaria Indicator Survey 2011 (Other DHS Direct)

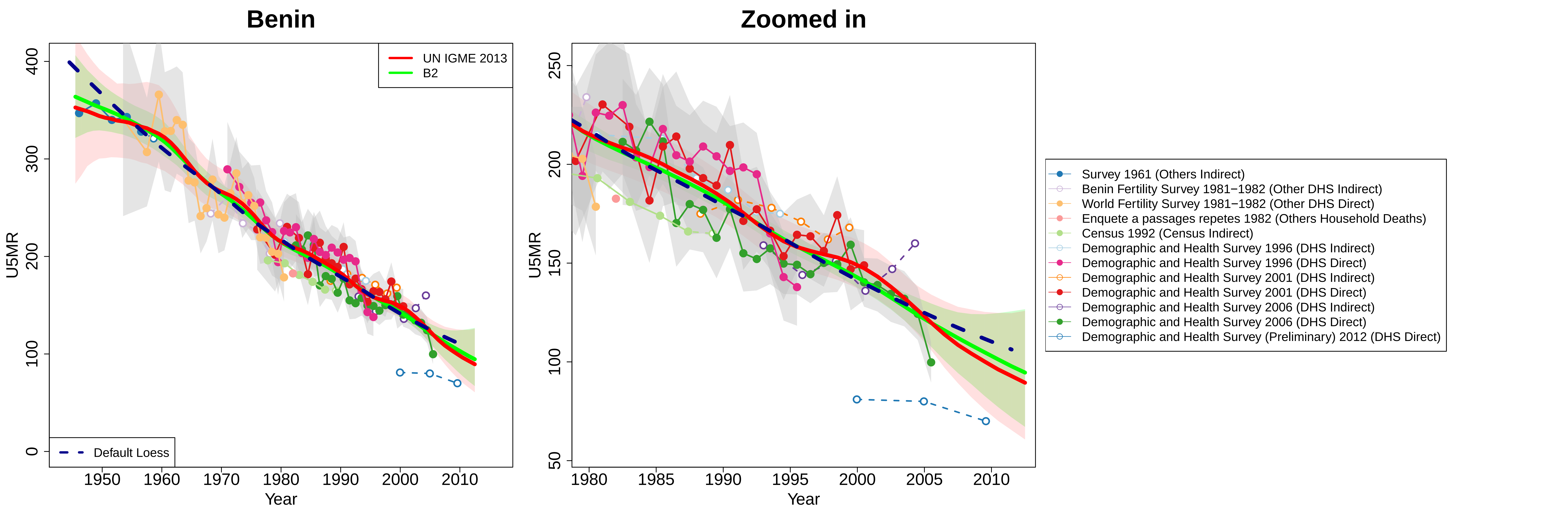
Benin
Zoomed in
U5MR
Year
UN IGME 2013
B2
Default Loess
Survey 1961 (Others Indirect)
Benin Fertility Survey 1981−1982 (Other DHS Indirect)
World Fertility Survey 1981−1982 (Other DHS Direct)
Enquete a passages repetes 1982 (Others Household Deaths)
Census 1992 (Census Indirect)
Demographic and Health Survey 1996 (DHS Indirect)
Demographic and Health Survey 1996 (DHS Direct)
Demographic and Health Survey 2001 (DHS Indirect)
Demographic and Health Survey 2001 (DHS Direct)
Demographic and Health Survey 2006 (DHS Indirect)
Demographic and Health Survey 2006 (DHS Direct)
Demographic and Health Survey (Preliminary) 2012 (DHS Direct)

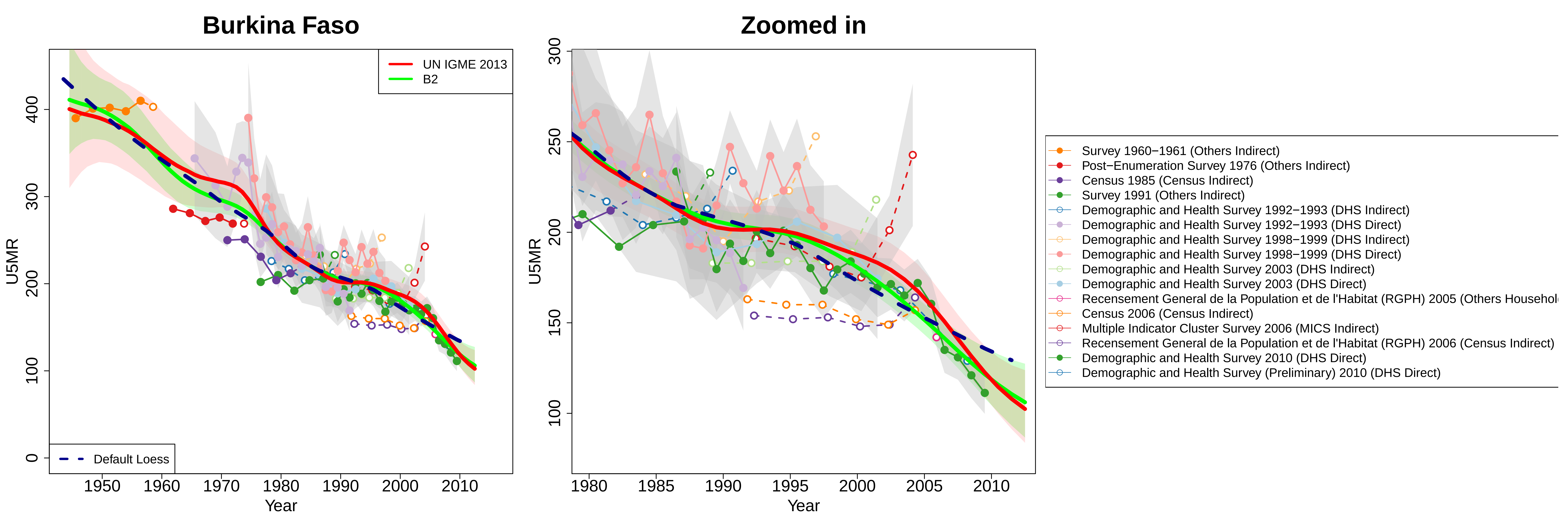

Burkina Faso
Zoomed in
UN IGME 2013
B2
Default Loess
U5MR
Year
0
100
200
300
400
1950
1960
1970
1980
1990
2000
2010
100
150
200
250
300
1980
1985
1990
1995
2000
2005
2010
Survey 1960−1961 (Others Indirect)
Post−Enumeration Survey 1976 (Others Indirect)
Census 1985 (Census Indirect)
Survey 1991 (Others Indirect)
Demographic and Health Survey 1992−1993 (DHS Indirect)
Demographic and Health Survey 1992−1993 (DHS Direct)
Demographic and Health Survey 1998−1999 (DHS Indirect)
Demographic and Health Survey 1998−1999 (DHS Direct)
Demographic and Health Survey 2003 (DHS Indirect)
Demographic and Health Survey 2003 (DHS Direct)
Recensement General de la Population et de l'Habitat (RGPH) 2005 (Others Househol
Census 2006 (Census Indirect)
Multiple Indicator Cluster Survey 2006 (MICS Indirect)
Recensement General de la Population et de l'Habitat (RGPH) 2006 (Census Indirect)
Demographic and Health Survey 2010 (DHS Direct)
Demographic and Health Survey (Preliminary) 2010 (DHS Direct)

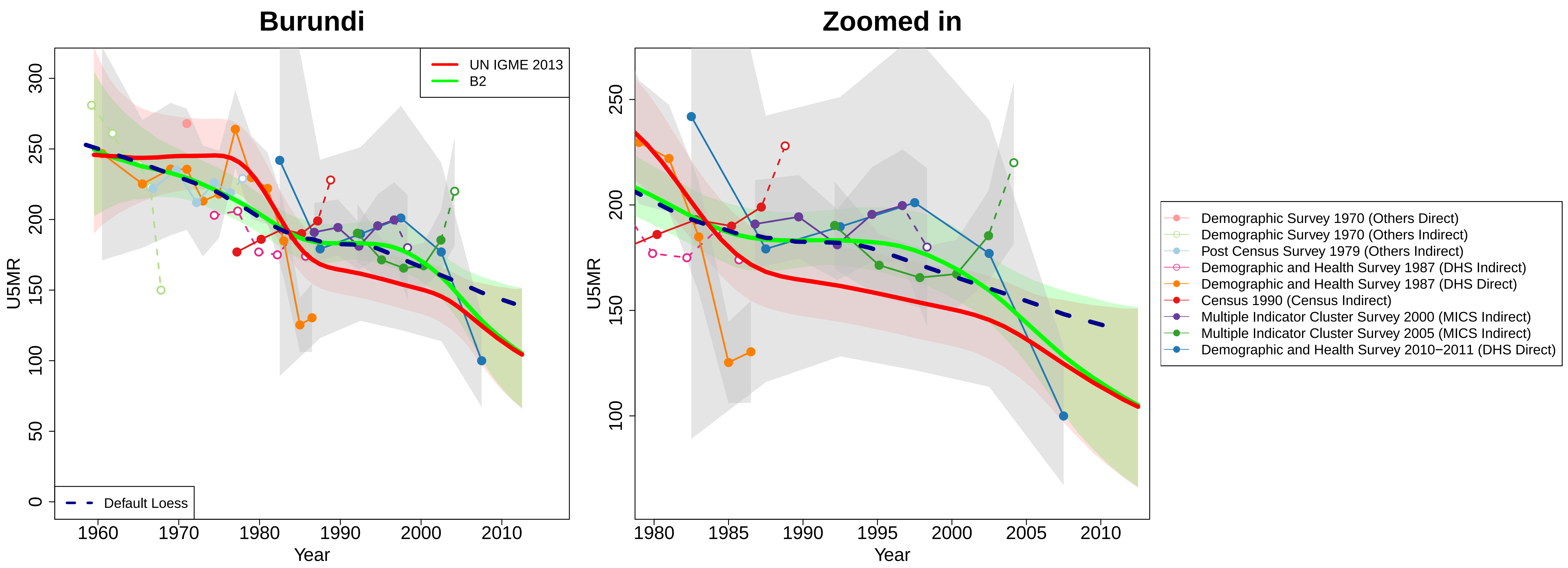
Burundi
Zoomed in
U5MR
Year
UN IGME 2013
B2
Default Loess
Demographic Survey 1970 (Others Direct)
Demographic Survey 1970 (Others Indirect)
Post Census Survey 1979 (Others Indirect)
Demographic and Health Survey 1987 (DHS Indirect)
Demographic and Health Survey 1987 (DHS Direct)
Census 1990 (Census Indirect)
Multiple Indicator Cluster Survey 2000 (MICS Indirect)
Multiple Indicator Cluster Survey 2005 (MICS Indirect)
Demographic and Health Survey 2010–2011 (DHS Direct)

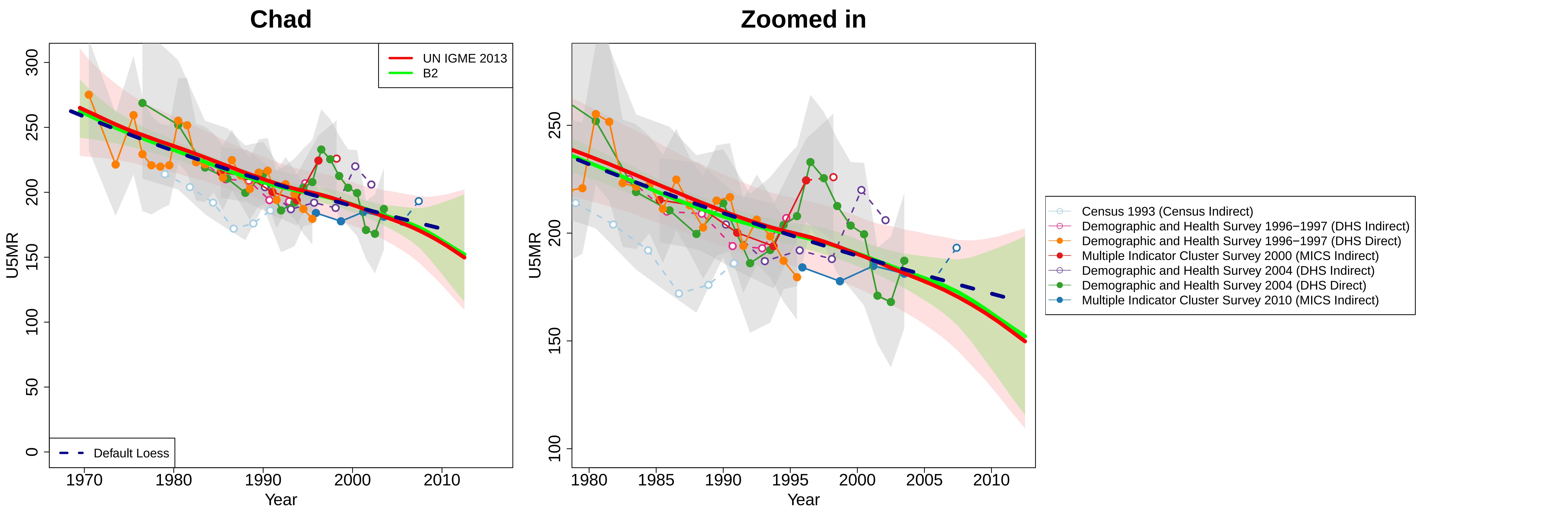
Chad
Zoomed in
UN IGME 2013
B2
Default Loess
U5MR
Year
Census 1993 (Census Indirect)
Demographic and Health Survey 1996−1997 (DHS Indirect)
Demographic and Health Survey 1996−1997 (DHS Direct)
Multiple Indicator Cluster Survey 2000 (MICS Indirect)
Demographic and Health Survey 2004 (DHS Indirect)
Demographic and Health Survey 2004 (DHS Direct)
Multiple Indicator Cluster Survey 2010 (MICS Indirect)

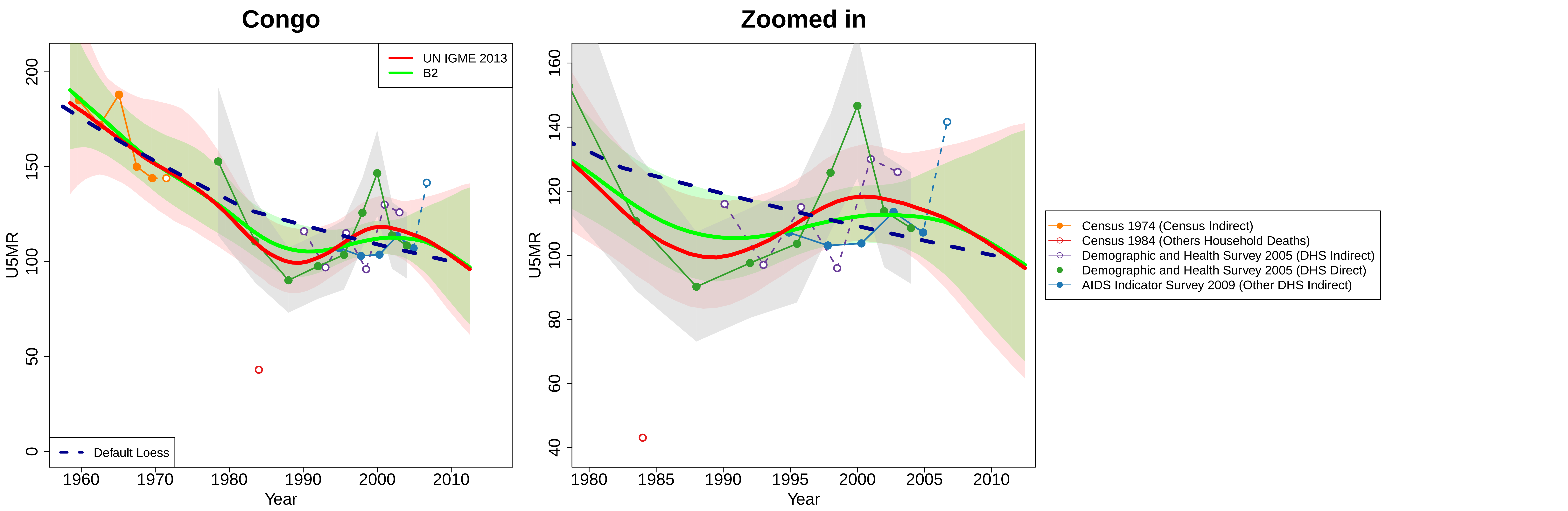

Congo
Zoomed in
UN IGME 2013
B2
Default Loess
U5MR
Year
Census 1974 (Census Indirect)
Census 1984 (Others Household Deaths)
Demographic and Health Survey 2005 (DHS Indirect)
Demographic and Health Survey 2005 (DHS Direct)
AIDS Indicator Survey 2009 (Other DHS Indirect)

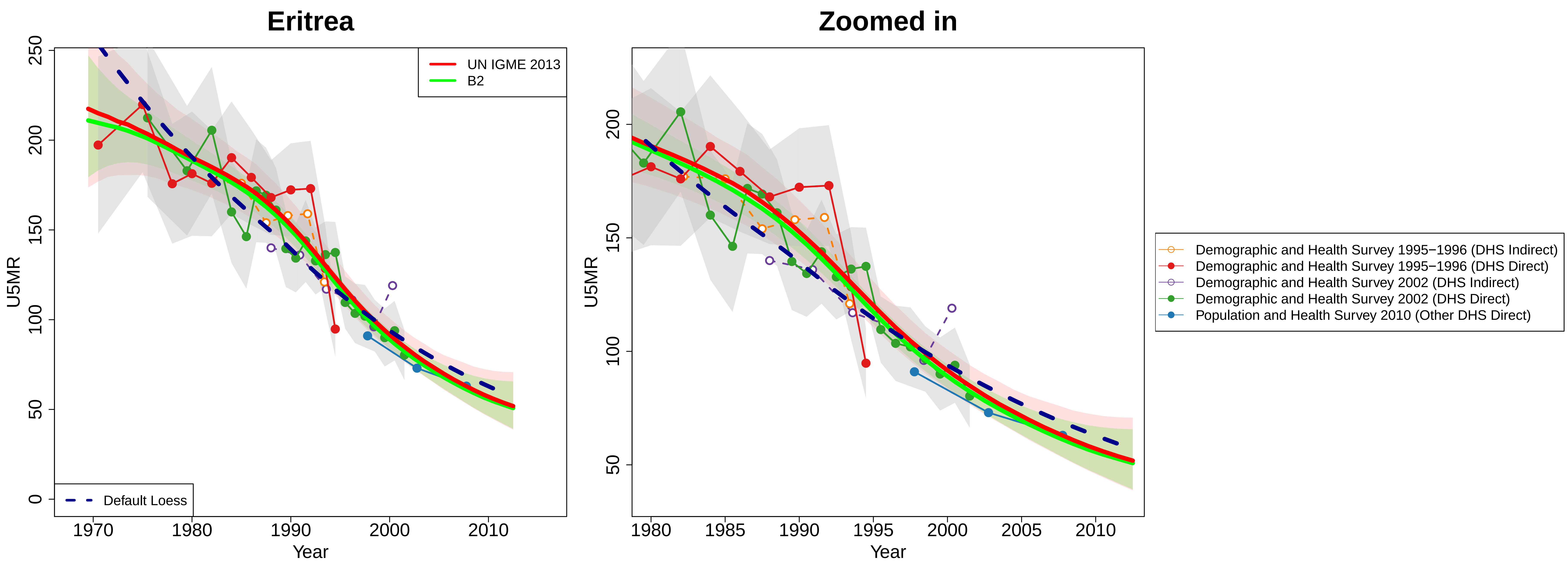

Eritrea
Zoomed in
UN IGME 2013
B2
Default Loess
U5MR
Year
Demographic and Health Survey 1995−1996 (DHS Indirect)
Demographic and Health Survey 1995−1996 (DHS Direct)
Demographic and Health Survey 2002 (DHS Indirect)
Demographic and Health Survey 2002 (DHS Direct)
Population and Health Survey 2010 (Other DHS Direct)

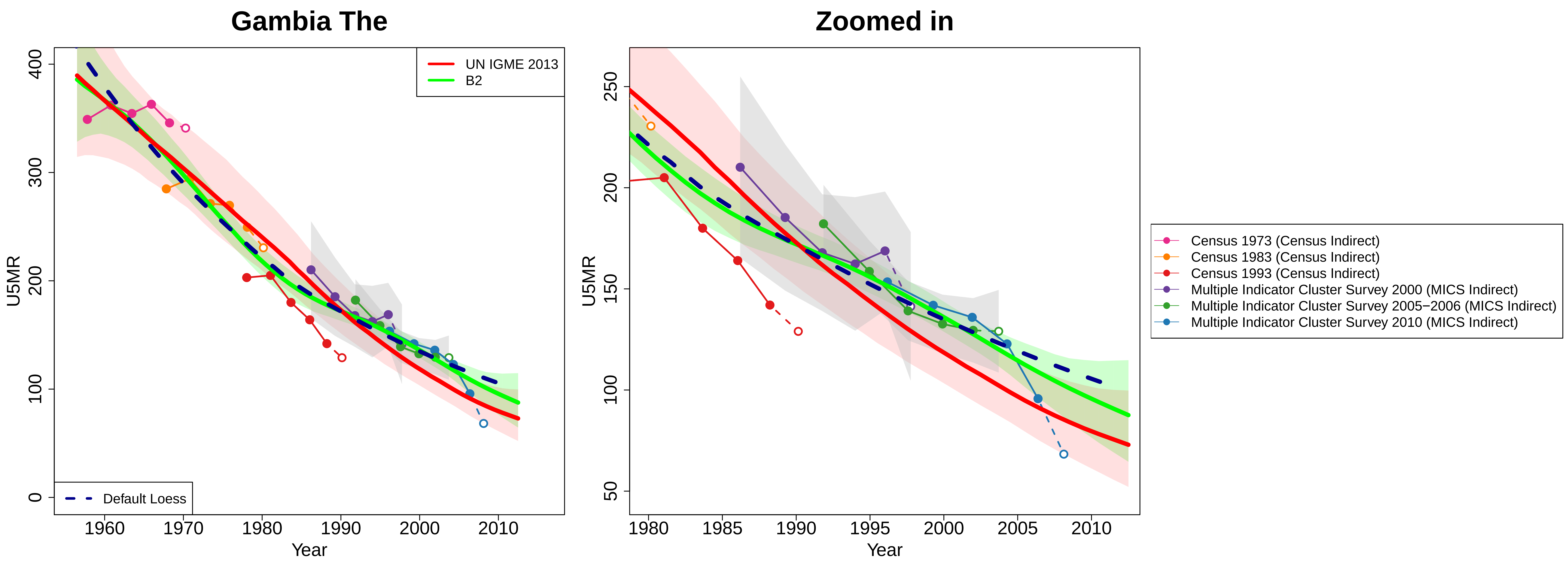

Gambia The
Zoomed in
UN IGME 2013
B2
Default Loess
U5MR
Year
Census 1973 (Census Indirect)
Census 1983 (Census Indirect)
Census 1993 (Census Indirect)
Multiple Indicator Cluster Survey 2000 (MICS Indirect)
Multiple Indicator Cluster Survey 2005−2006 (MICS Indirect)
Multiple Indicator Cluster Survey 2010 (MICS Indirect)

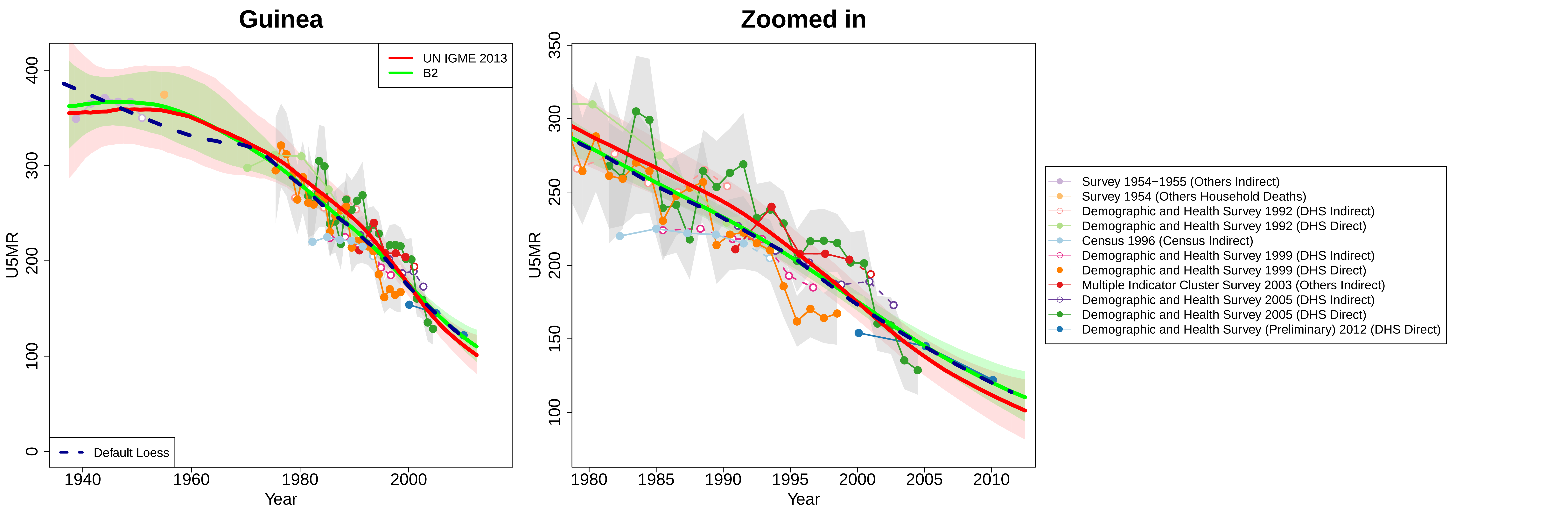

Guinea
Zoomed in
U5MR
Year
UN IGME 2013
B2
Default Loess
1940
1960
1980
2000
0
100
200
300
400
1980
1985
1990
1995
2000
2005
2010
100
150
200
250
300
350
Survey 1954−1955 (Others Indirect)
Survey 1954 (Others Household Deaths)
Demographic and Health Survey 1992 (DHS Indirect)
Demographic and Health Survey 1992 (DHS Direct)
Census 1996 (Census Indirect)
Demographic and Health Survey 1999 (DHS Indirect)
Demographic and Health Survey 1999 (DHS Direct)
Multiple Indicator Cluster Survey 2003 (Others Indirect)
Demographic and Health Survey 2005 (DHS Indirect)
Demographic and Health Survey 2005 (DHS Direct)
Demographic and Health Survey (Preliminary) 2012 (DHS Direct)

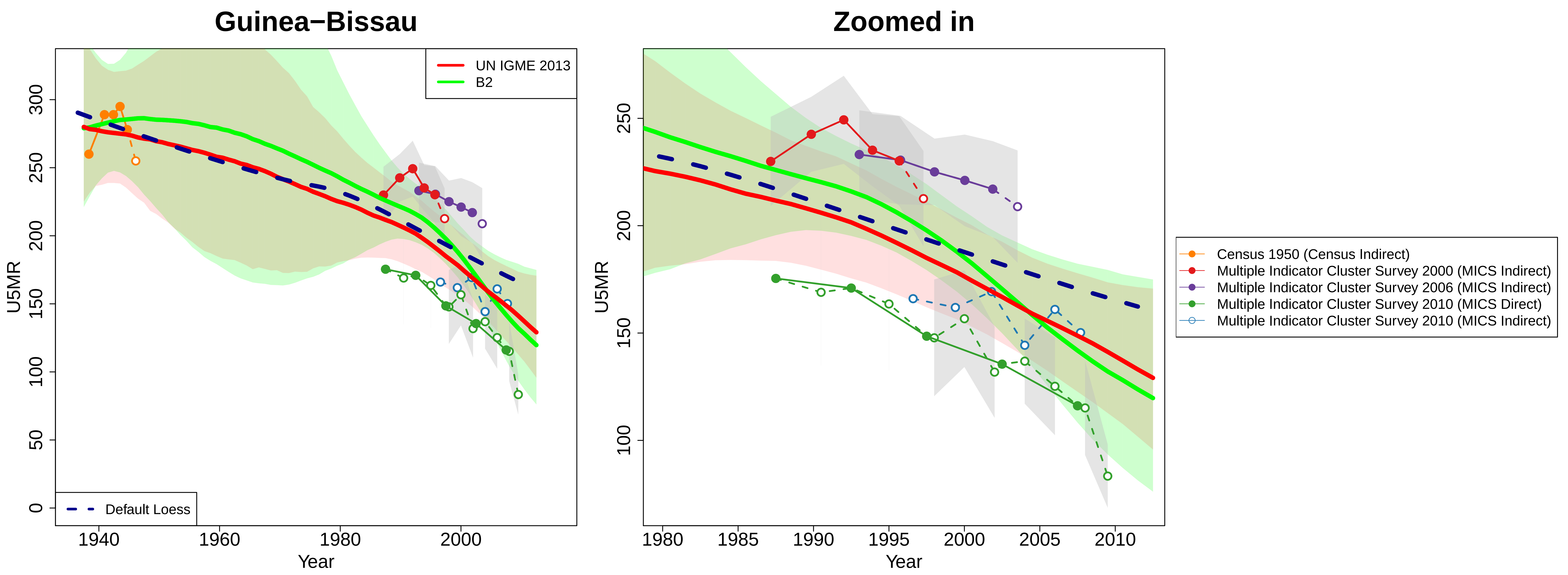
Guinea−Bissau
Zoomed in
UN IGME 2013
B2
Default Loess
U5MR
Year
Census 1950 (Census Indirect)
Multiple Indicator Cluster Survey 2000 (MICS Indirect)
Multiple Indicator Cluster Survey 2006 (MICS Indirect)
Multiple Indicator Cluster Survey 2010 (MICS Direct)
Multiple Indicator Cluster Survey 2010 (MICS Indirect)

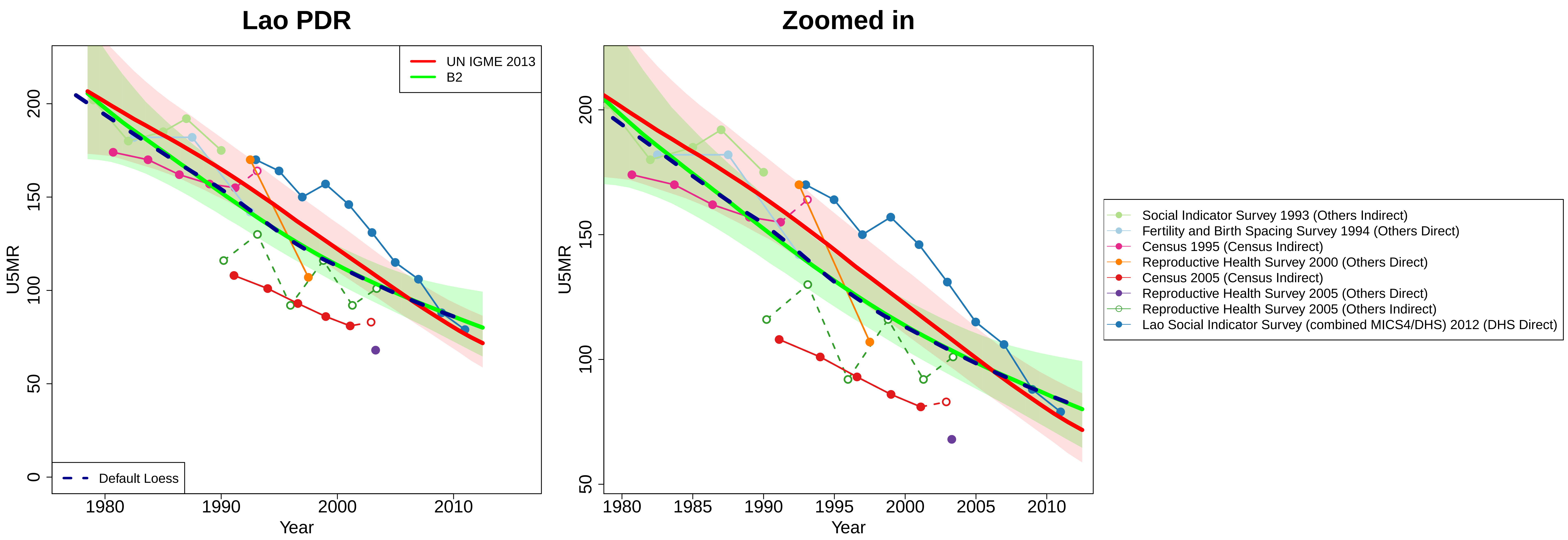

Lao PDR
Zoomed in
UN IGME 2013
B2
Default Loess
U5MR
Year
0
50
100
150
200
1980
1985
1990
1995
2000
2005
2010
Social Indicator Survey 1993 (Others Indirect)
Fertility and Birth Spacing Survey 1994 (Others Direct)
Census 1995 (Census Indirect)
Reproductive Health Survey 2000 (Others Direct)
Census 2005 (Census Indirect)
Reproductive Health Survey 2005 (Others Direct)
Reproductive Health Survey 2005 (Others Indirect)
Lao Social Indicator Survey (combined MICS4/DHS) 2012 (DHS Direct)

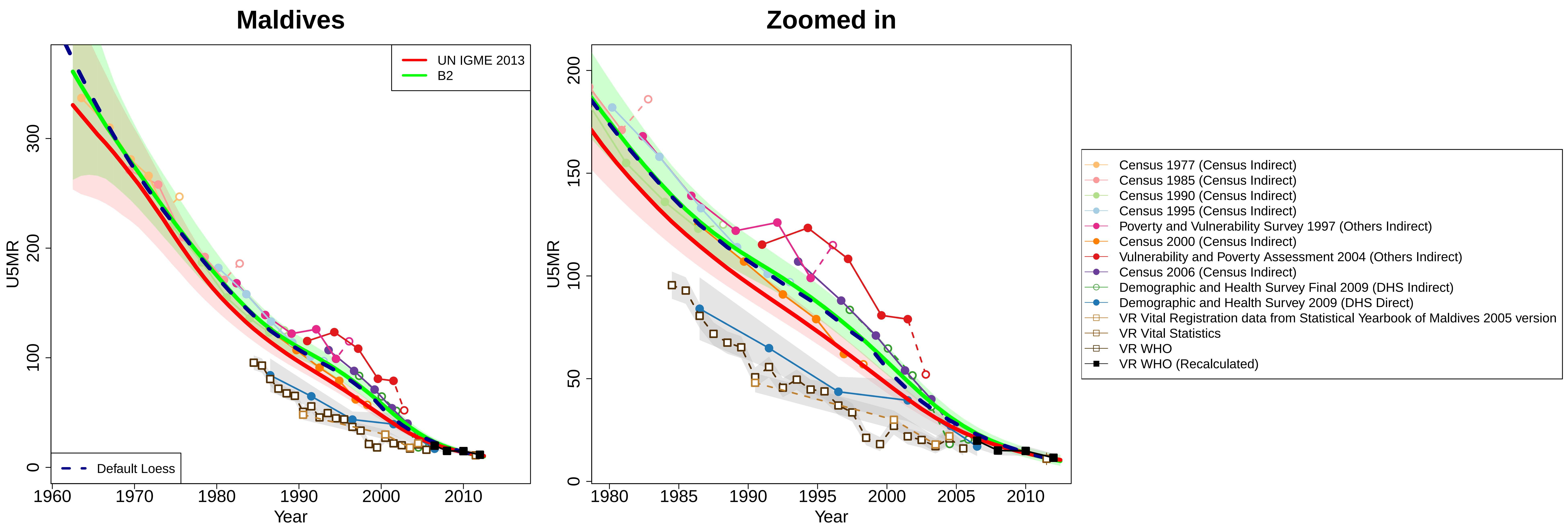
Maldives
Zoomed in
UN IGME 2013
B2
Default Loess
U5MR
Year
Census 1977 (Census Indirect)
Census 1985 (Census Indirect)
Census 1990 (Census Indirect)
Census 1995 (Census Indirect)
Poverty and Vulnerability Survey 1997 (Others Indirect)
Census 2000 (Census Indirect)
Vulnerability and Poverty Assessment 2004 (Others Indirect)
Census 2006 (Census Indirect)
Demographic and Health Survey Final 2009 (DHS Indirect)
Demographic and Health Survey 2009 (DHS Direct)
VR Vital Registration data from Statistical Yearbook of Maldives 2005 version
VR Vital Statistics
VR WHO
VR WHO (Recalculated)

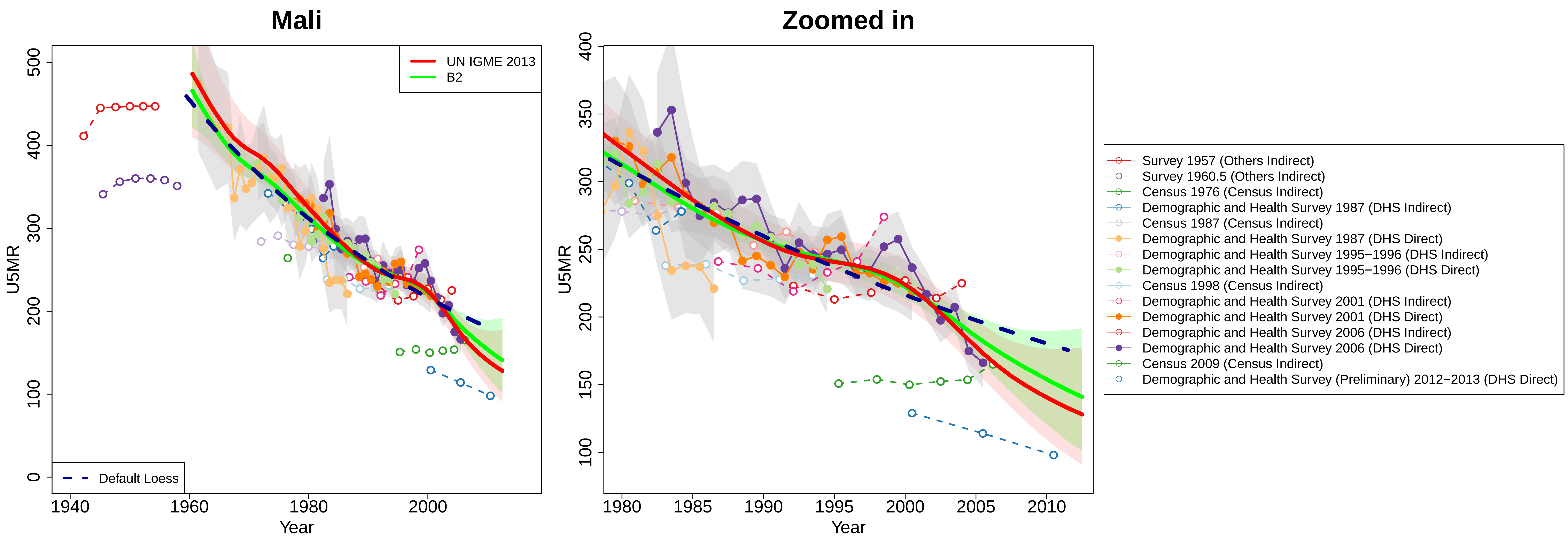
Mali
Zoomed in
UN IGME 2013
B2
Default Loess
U5MR
Year
Survey 1957 (Others Indirect)
Survey 1960.5 (Others Indirect)
Census 1976 (Census Indirect)
Demographic and Health Survey 1987 (DHS Indirect)
Census 1987 (Census Indirect)
Demographic and Health Survey 1987 (DHS Direct)
Demographic and Health Survey 1995−1996 (DHS Indirect)
Demographic and Health Survey 1995−1996 (DHS Direct)
Census 1998 (Census Indirect)
Demographic and Health Survey 2001 (DHS Indirect)
Demographic and Health Survey 2001 (DHS Direct)
Demographic and Health Survey 2006 (DHS Indirect)
Demographic and Health Survey 2006 (DHS Direct)
Census 2009 (Census Indirect)
Demographic and Health Survey (Preliminary) 2012−2013 (DHS Direct)

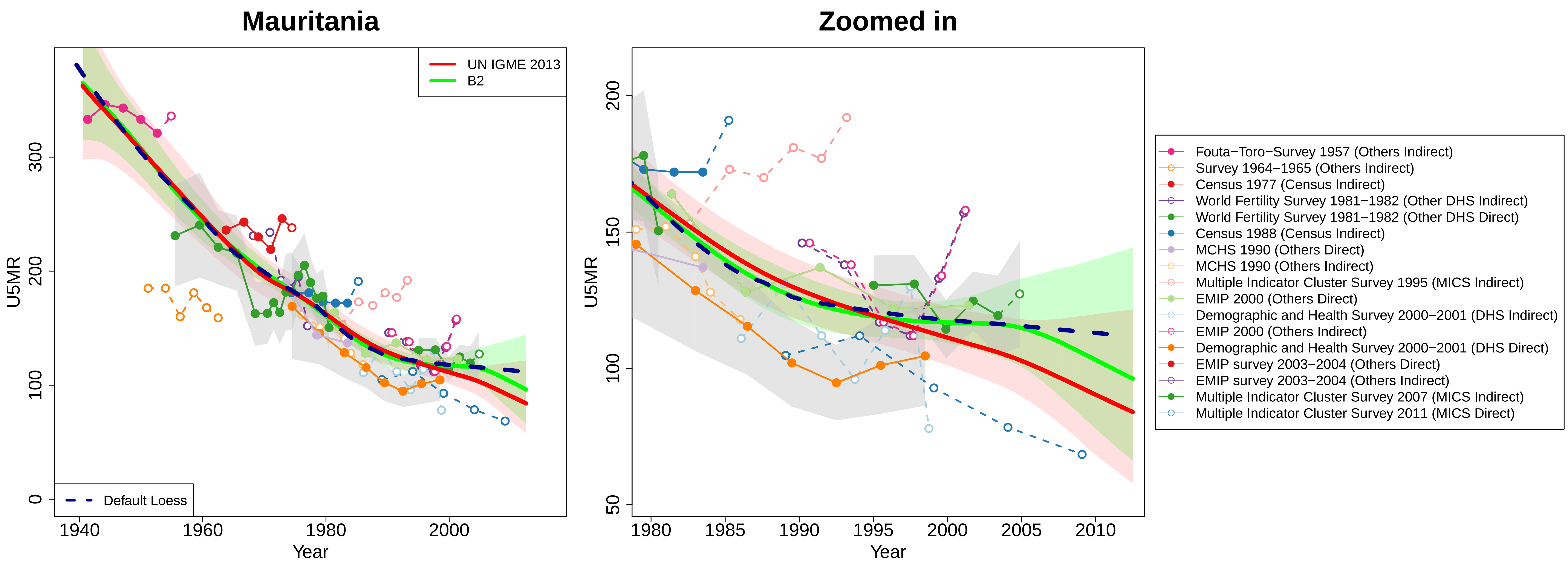

Mauritania
Zoomed in
UN IGME 2013
B2
Default Loess
U5MR
Year
0
100
200
300
1940
1960
1980
2000
50
100
150
200
1980
1985
1990
1995
2000
2005
2010
Fouta−Toro−Survey 1957 (Others Indirect)
Survey 1964−1965 (Others Indirect)
Census 1977 (Census Indirect)
World Fertility Survey 1981−1982 (Other DHS Indirect)
World Fertility Survey 1981−1982 (Other DHS Direct)
Census 1988 (Census Indirect)
MCHS 1990 (Others Direct)
MCHS 1990 (Others Indirect)
Multiple Indicator Cluster Survey 1995 (MICS Indirect)
EMIP 2000 (Others Direct)
Demographic and Health Survey 2000−2001 (DHS Indirect)
EMIP 2000 (Others Indirect)
Demographic and Health Survey 2000−2001 (DHS Direct)
EMIP survey 2003−2004 (Others Direct)
EMIP survey 2003−2004 (Others Indirect)
Multiple Indicator Cluster Survey 2007 (MICS Indirect)
Multiple Indicator Cluster Survey 2011 (MICS Direct)

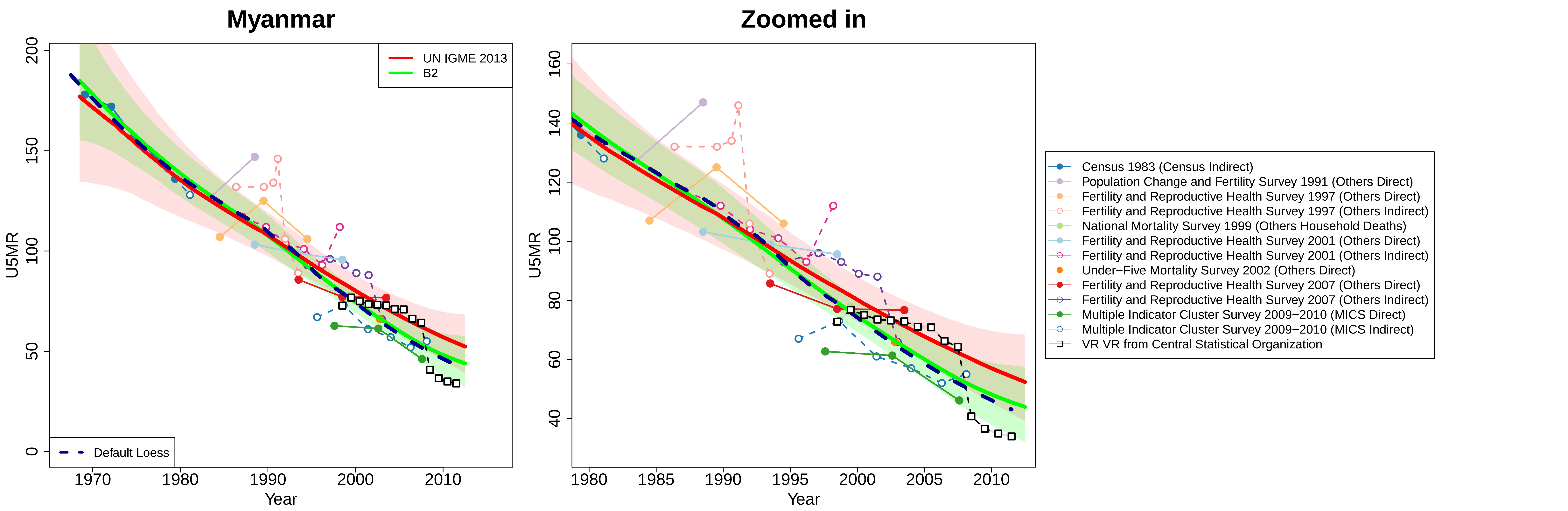

Myanmar
Zoomed in
UN IGME 2013
B2
Default Loess
U5MR
Year
Census 1983 (Census Indirect)
Population Change and Fertility Survey 1991 (Others Direct)
Fertility and Reproductive Health Survey 1997 (Others Direct)
Fertility and Reproductive Health Survey 1997 (Others Indirect)
National Mortality Survey 1999 (Others Household Deaths)
Fertility and Reproductive Health Survey 2001 (Others Direct)
Fertility and Reproductive Health Survey 2001 (Others Indirect)
Under−Five Mortality Survey 2002 (Others Direct)
Fertility and Reproductive Health Survey 2007 (Others Direct)
Fertility and Reproductive Health Survey 2007 (Others Indirect)
Multiple Indicator Cluster Survey 2009−2010 (MICS Direct)
Multiple Indicator Cluster Survey 2009−2010 (MICS Indirect)
VR VR from Central Statistical Organization

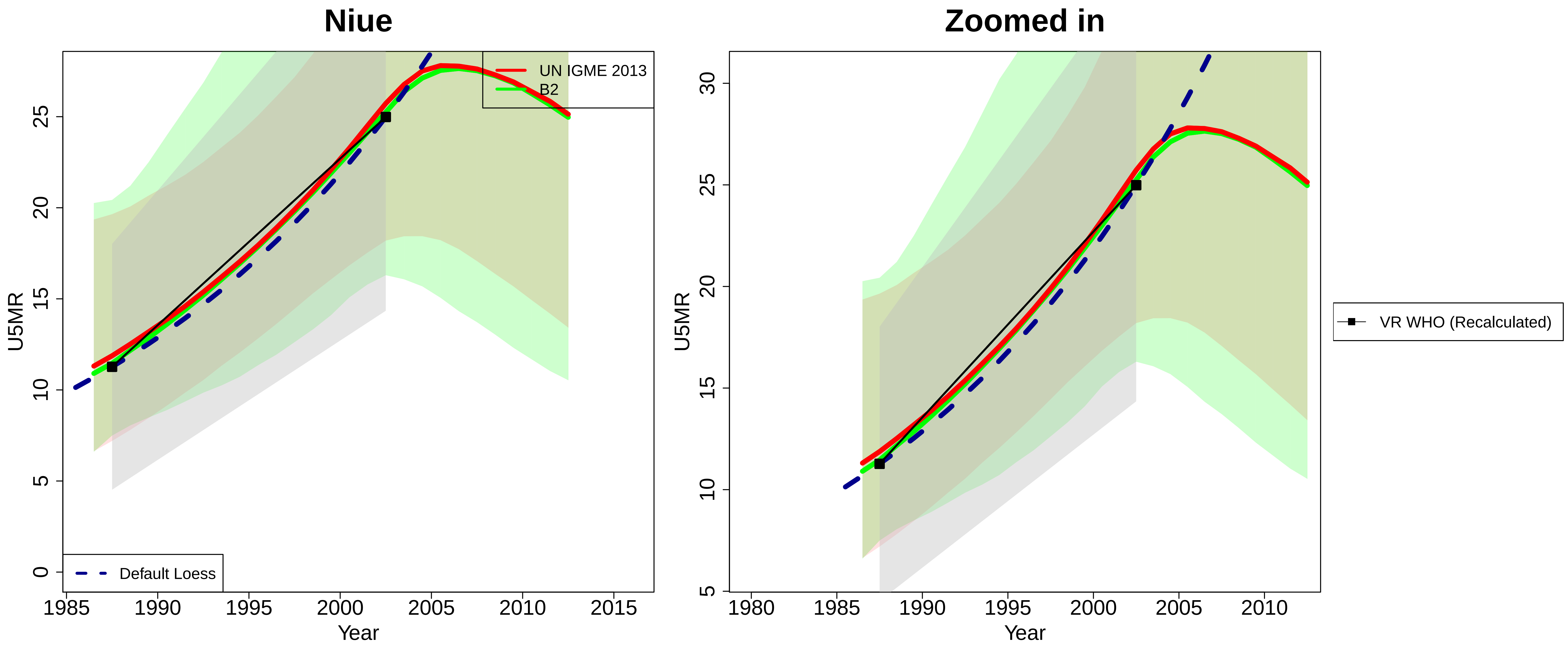
Niue
Zoomed in
UN IGME 2013
B2
Default Loess
VR WHO (Recalculated)
U5MR
Year
0
5
10
15
20
25
30
1980
1985
1990
1995
2000
2005
2010
2015

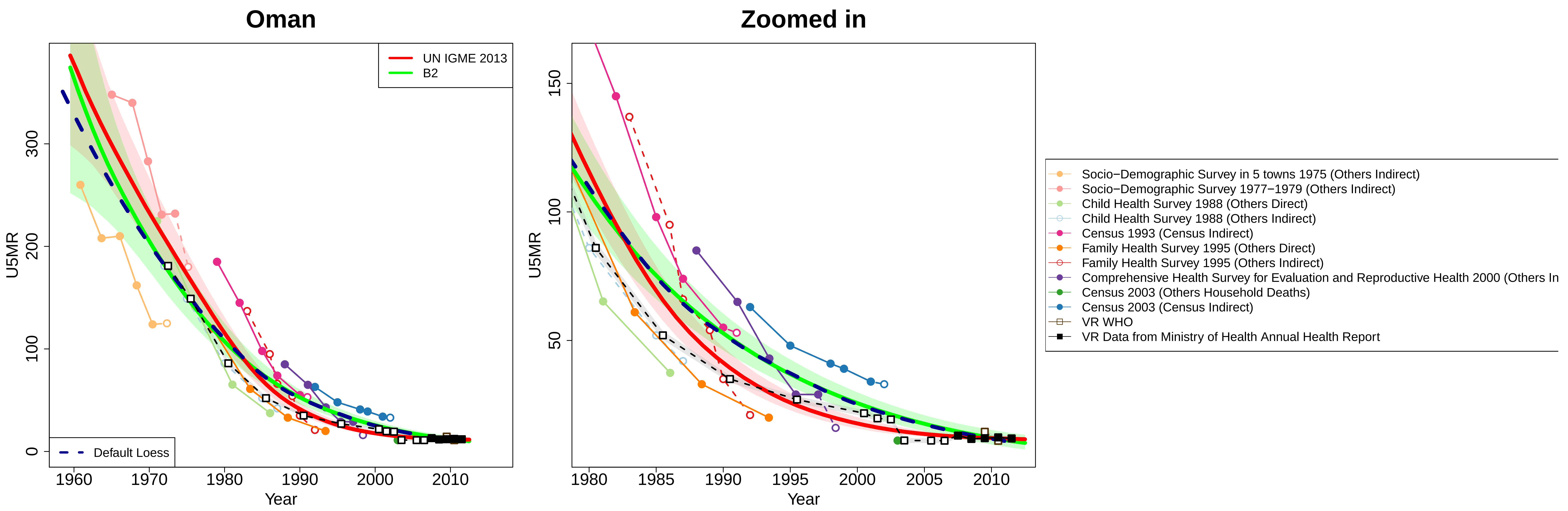
Oman
Zoomed in
UN IGME 2013
B2
Default Loess
U5MR
Year
Socio−Demographic Survey in 5 towns 1975 (Others Indirect)
Socio−Demographic Survey 1977−1979 (Others Indirect)
Child Health Survey 1988 (Others Direct)
Child Health Survey 1988 (Others Indirect)
Census 1993 (Census Indirect)
Family Health Survey 1995 (Others Direct)
Family Health Survey 1995 (Others Indirect)
Comprehensive Health Survey for Evaluation and Reproductive Health 2000 (Others In
Census 2003 (Others Household Deaths)
Census 2003 (Census Indirect)
VR WHO
VR Data from Ministry of Health Annual Health Report

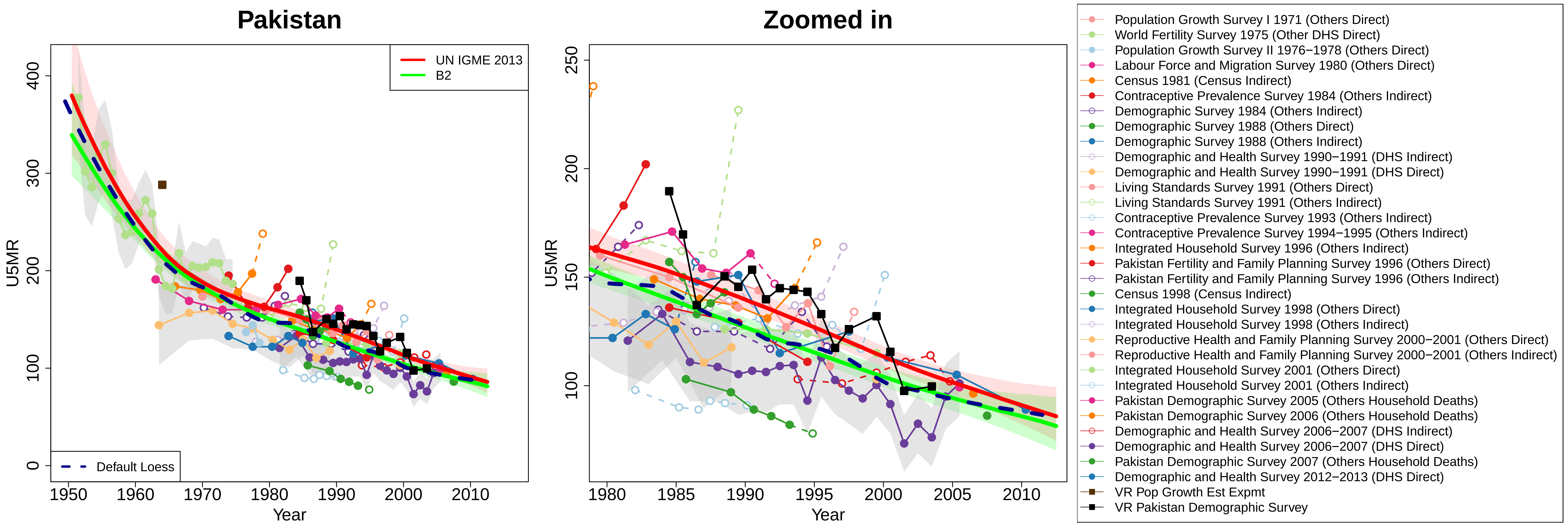

Pakistan
Zoomed in
U5MR
Year
0
100
200
300
400
1950
1960
1970
1980
1990
2000
2010
UN IGME 2013
B2
Default Loess
100
150
200
250
1980
1985
1990
1995
2000
2005
2010
Population Growth Survey I 1971 (Others Direct)
World Fertility Survey 1975 (Other DHS Direct)
Population Growth Survey II 1976−1978 (Others Direct)
Labour Force and Migration Survey 1980 (Others Direct)
Census 1981 (Census Indirect)
Contraceptive Prevalence Survey 1984 (Others Indirect)
Demographic Survey 1984 (Others Indirect)
Demographic Survey 1988 (Others Direct)
Demographic Survey 1988 (Others Indirect)
Demographic and Health Survey 1990−1991 (DHS Indirect)
Demographic and Health Survey 1990−1991 (DHS Direct)
Living Standards Survey 1991 (Others Direct)
Living Standards Survey 1991 (Others Indirect)
Contraceptive Prevalence Survey 1993 (Others Indirect)
Contraceptive Prevalence Survey 1994−1995 (Others Indirect)
Integrated Household Survey 1996 (Others Indirect)
Pakistan Fertility and Family Planning Survey 1996 (Others Direct)
Pakistan Fertility and Family Planning Survey 1996 (Others Indirect)
Census 1998 (Census Indirect)
Integrated Household Survey 1998 (Others Direct)
Integrated Household Survey 1998 (Others Indirect)
Reproductive Health and Family Planning Survey 2000−2001 (Others Direct)
Reproductive Health and Family Planning Survey 2000−2001 (Others Indirect)
Integrated Household Survey 2001 (Others Direct)
Integrated Household Survey 2001 (Others Indirect)
Pakistan Demographic Survey 2005 (Others Household Deaths)
Pakistan Demographic Survey 2006 (Others Household Deaths)
Demographic and Health Survey 2006−2007 (DHS Indirect)
Demographic and Health Survey 2006−2007 (DHS Direct)
Pakistan Demographic Survey 2007 (Others Household Deaths)
Demographic and Health Survey 2012−2013 (DHS Direct)
VR Pop Growth Est Expmt
VR Pakistan Demographic Survey

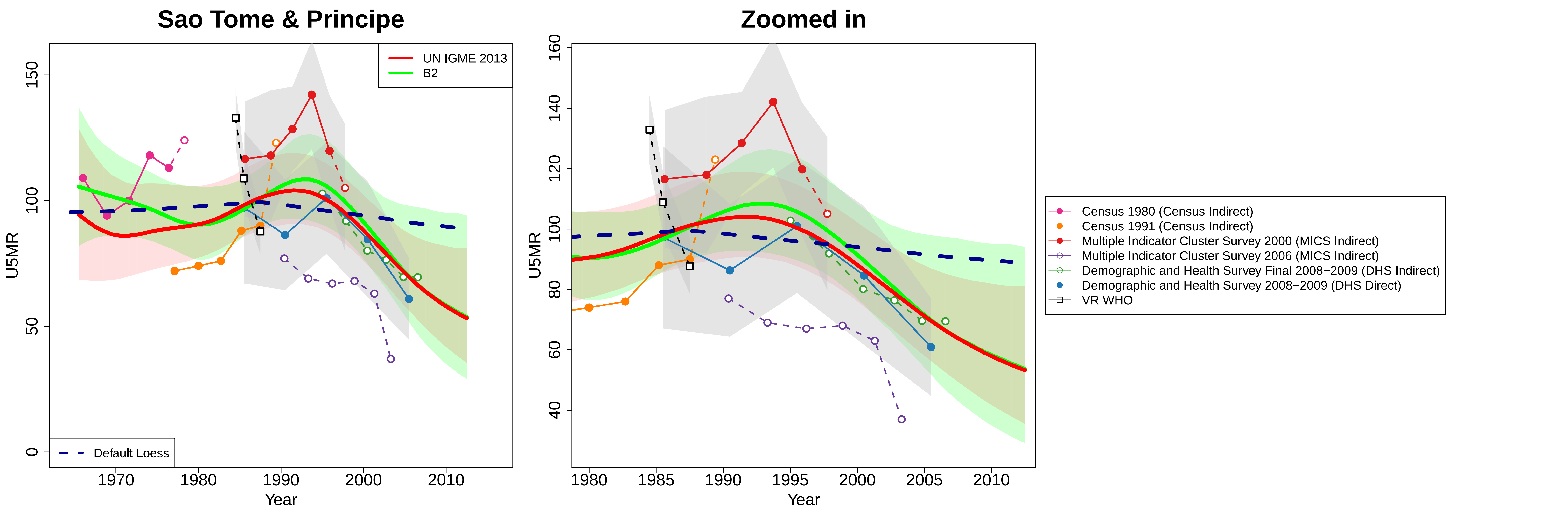
Sao Tome & Principe
Zoomed in
UN IGME 2013
B2
Default Loess
U5MR
Year
Census 1980 (Census Indirect)
Census 1991 (Census Indirect)
Multiple Indicator Cluster Survey 2000 (MICS Indirect)
Multiple Indicator Cluster Survey 2006 (MICS Indirect)
Demographic and Health Survey Final 2008−2009 (DHS Indirect)
Demographic and Health Survey 2008−2009 (DHS Direct)
VR WHO

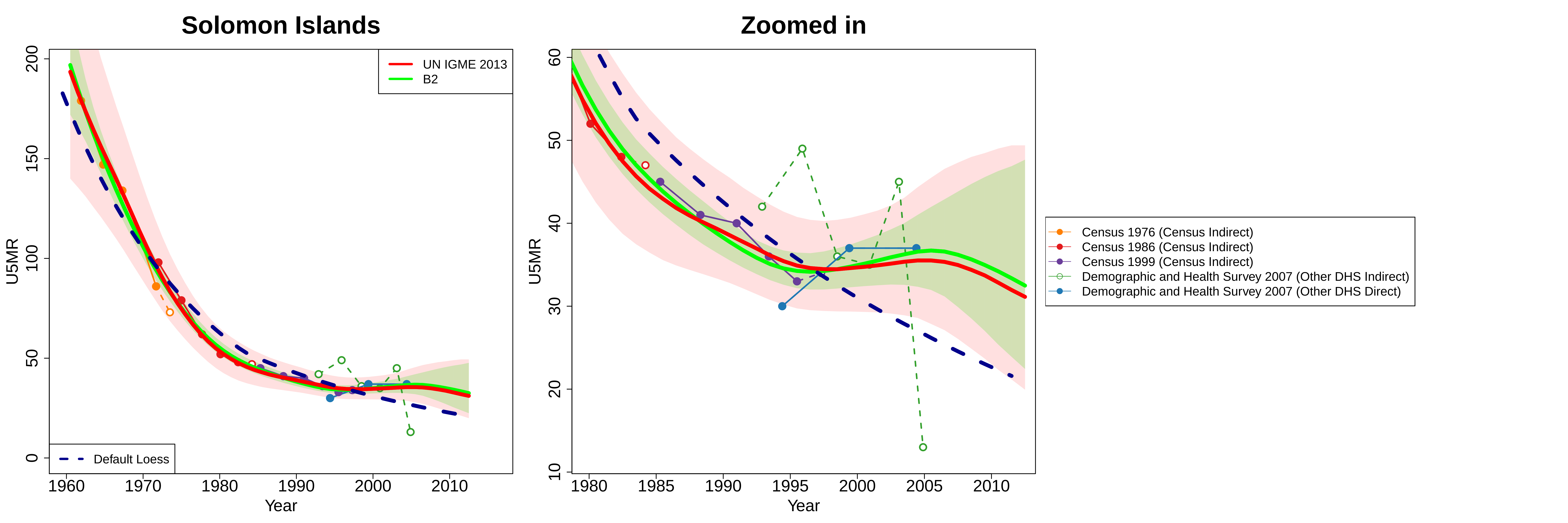
Solomon Islands
Zoomed in
UN IGME 2013
B2
Default Loess
U5MR
Year
Census 1976 (Census Indirect)
Census 1986 (Census Indirect)
Census 1999 (Census Indirect)
Demographic and Health Survey 2007 (Other DHS Indirect)
Demographic and Health Survey 2007 (Other DHS Direct)

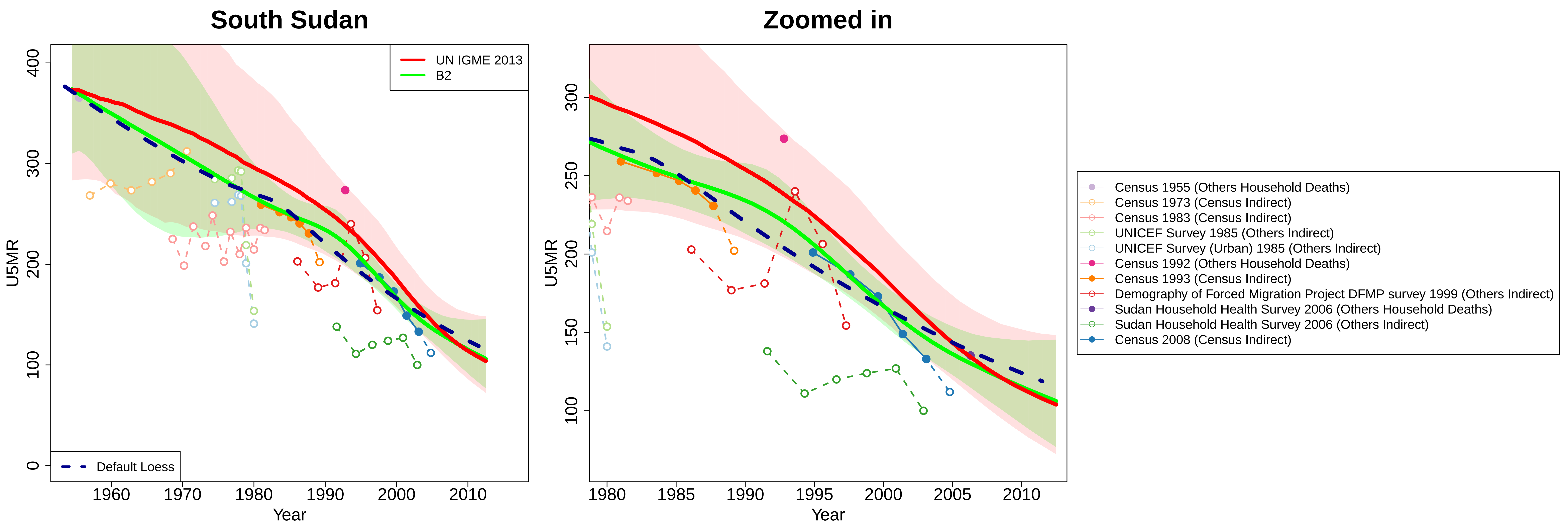

South Sudan
Zoomed in
UN IGME 2013
B2
Default Loess
U5MR
Year
Census 1955 (Others Household Deaths)
Census 1973 (Census Indirect)
Census 1983 (Census Indirect)
UNICEF Survey 1985 (Others Indirect)
UNICEF Survey (Urban) 1985 (Others Indirect)
Census 1992 (Others Household Deaths)
Census 1993 (Census Indirect)
Demography of Forced Migration Project DFMP survey 1999 (Others Indirect)
Sudan Household Health Survey 2006 (Others Household Deaths)
Sudan Household Health Survey 2006 (Others Indirect)
Census 2008 (Census Indirect)

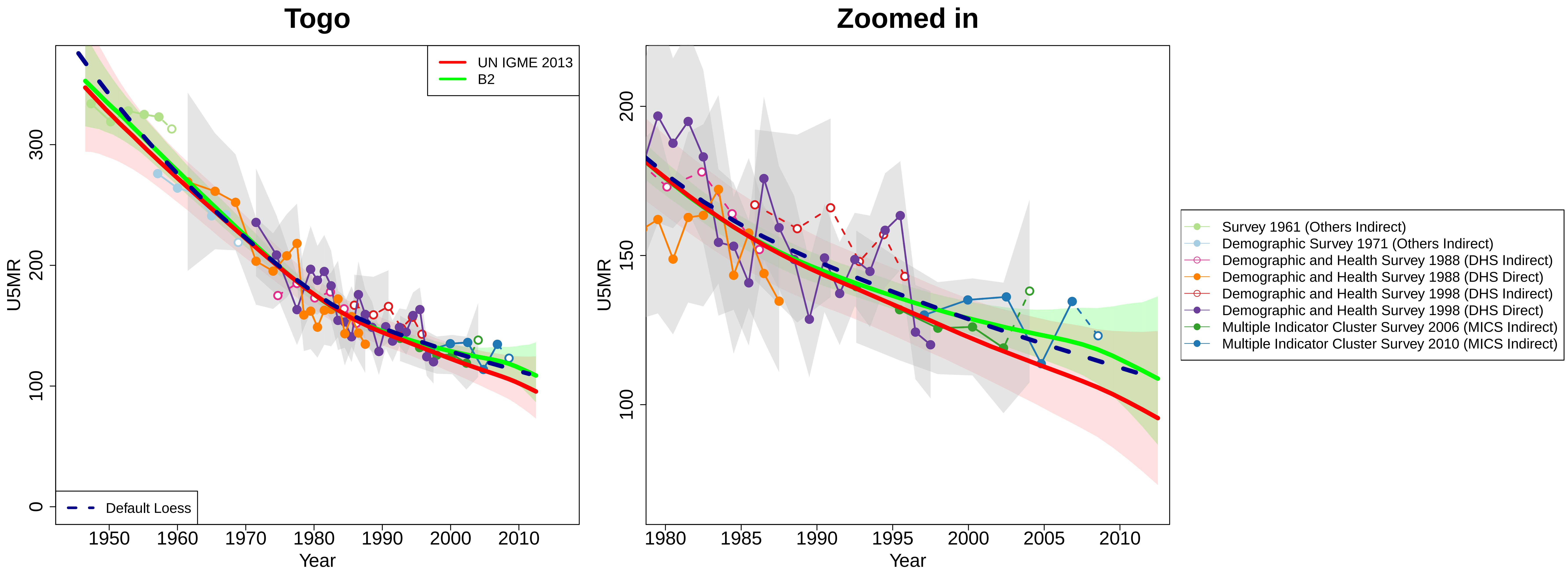
Togo
Zoomed in
U5MR
Year
UN IGME 2013
B2
Default Loess
Survey 1961 (Others Indirect)
Demographic Survey 1971 (Others Indirect)
Demographic and Health Survey 1988 (DHS Indirect)
Demographic and Health Survey 1988 (DHS Direct)
Demographic and Health Survey 1998 (DHS Indirect)
Demographic and Health Survey 1998 (DHS Direct)
Multiple Indicator Cluster Survey 2006 (MICS Indirect)
Multiple Indicator Cluster Survey 2010 (MICS Indirect)

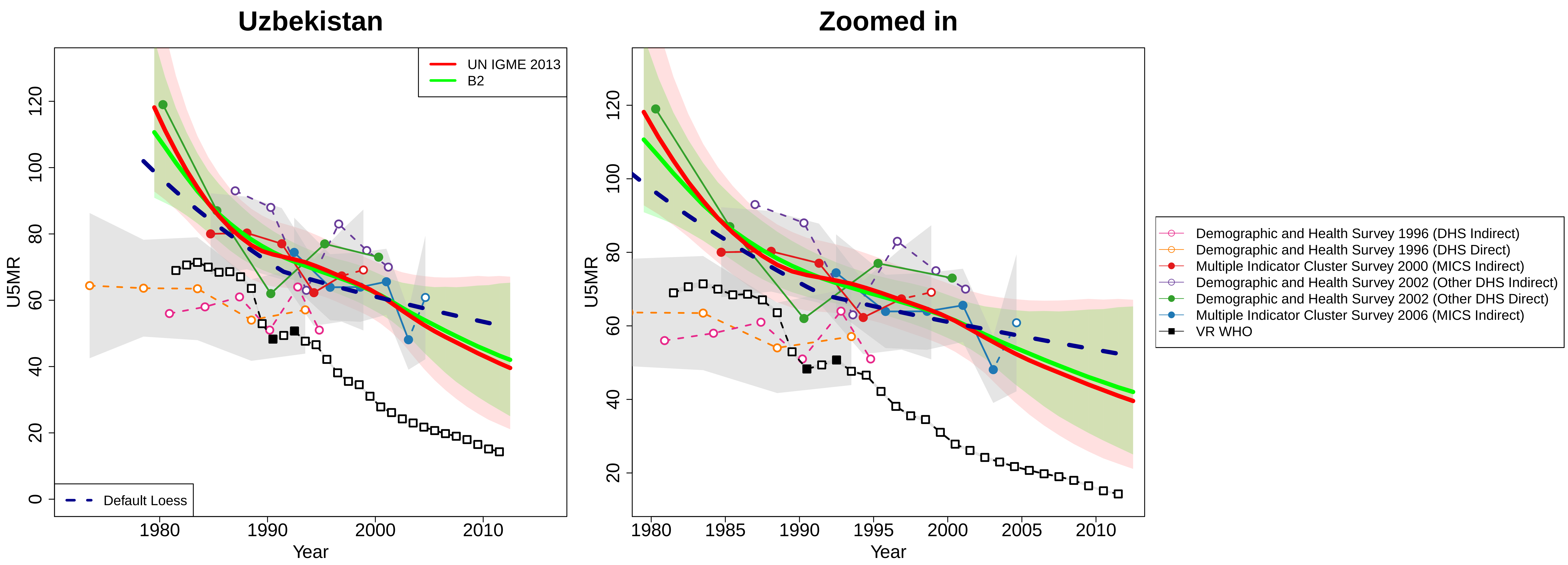
Uzbekistan
Zoomed in
UN IGME 2013
B2
Default Loess
U5MR
Year
Demographic and Health Survey 1996 (DHS Indirect)
Demographic and Health Survey 1996 (DHS Direct)
Multiple Indicator Cluster Survey 2000 (MICS Indirect)
Demographic and Health Survey 2002 (Other DHS Indirect)
Demographic and Health Survey 2002 (Other DHS Direct)
Multiple Indicator Cluster Survey 2006 (MICS Indirect)
VR WHO

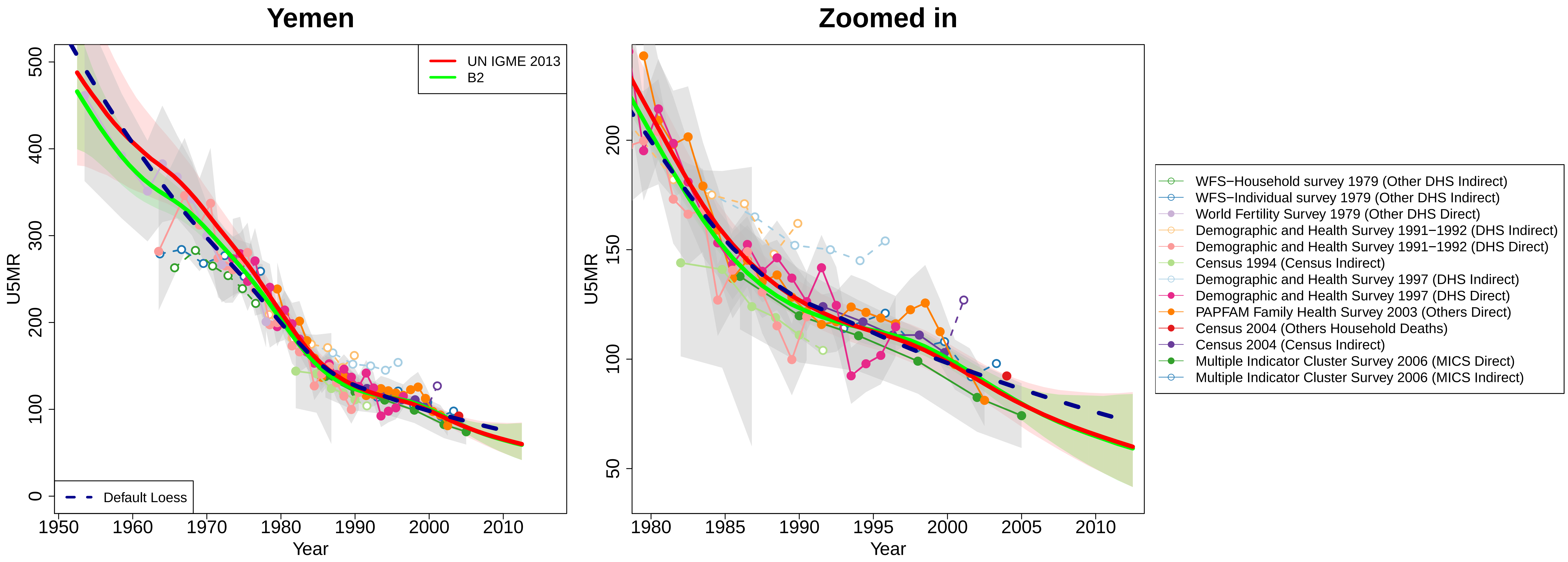

Yemen
Zoomed in
UN IGME 2013
B2
Default Loess
U5MR
Year
WFS−Household survey 1979 (Other DHS Indirect)
WFS−Individual survey 1979 (Other DHS Indirect)
World Fertility Survey 1979 (Other DHS Direct)
Demographic and Health Survey 1991−1992 (DHS Indirect)
Demographic and Health Survey 1991−1992 (DHS Direct)
Census 1994 (Census Indirect)
Demographic and Health Survey 1997 (DHS Indirect)
Demographic and Health Survey 1997 (DHS Direct)
PAPFAM Family Health Survey 2003 (Others Direct)
Census 2004 (Others Household Deaths)
Census 2004 (Census Indirect)
Multiple Indicator Cluster Survey 2006 (MICS Direct)
Multiple Indicator Cluster Survey 2006 (MICS Indirect)

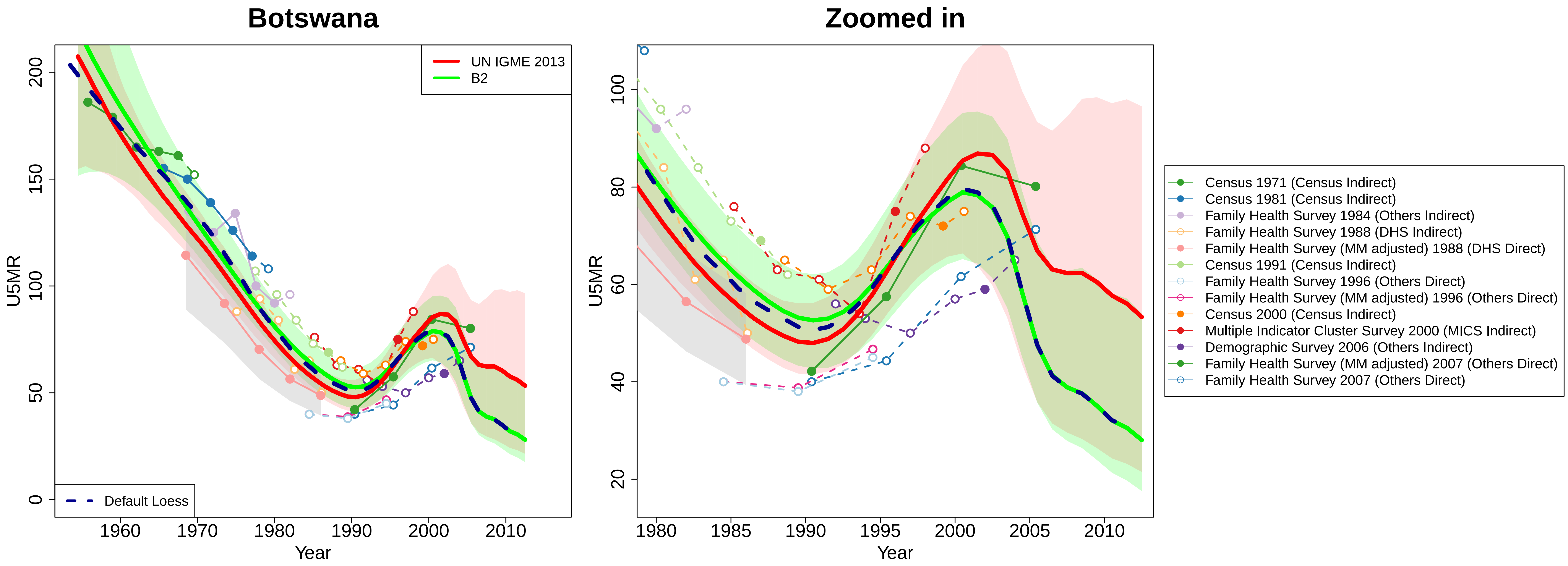

Botswana
Zoomed in
UN IGME 2013
B2
Default Loess
U5MR
Year
Census 1971 (Census Indirect)
Census 1981 (Census Indirect)
Family Health Survey 1984 (Others Indirect)
Family Health Survey 1988 (DHS Indirect)
Family Health Survey (MM adjusted) 1988 (DHS Direct)
Census 1991 (Census Indirect)
Family Health Survey 1996 (Others Direct)
Family Health Survey (MM adjusted) 1996 (Others Direct)
Census 2000 (Census Indirect)
Multiple Indicator Cluster Survey 2000 (MICS Indirect)
Demographic Survey 2006 (Others Indirect)
Family Health Survey (MM adjusted) 2007 (Others Direct)
Family Health Survey 2007 (Others Direct)

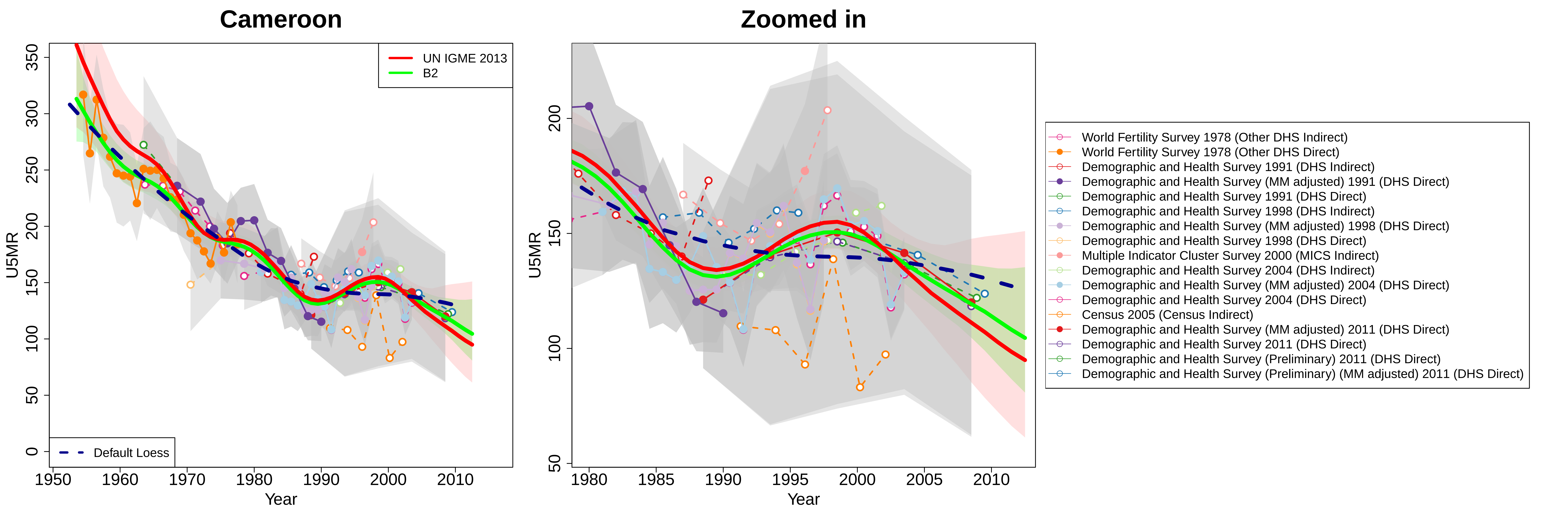
Cameroon
Zoomed in
UN IGME 2013
B2
Default Loess
U5MR
Year
World Fertility Survey 1978 (Other DHS Indirect)
World Fertility Survey 1978 (Other DHS Direct)
Demographic and Health Survey 1991 (DHS Indirect)
Demographic and Health Survey (MM adjusted) 1991 (DHS Direct)
Demographic and Health Survey 1991 (DHS Direct)
Demographic and Health Survey 1998 (DHS Indirect)
Demographic and Health Survey (MM adjusted) 1998 (DHS Direct)
Demographic and Health Survey 1998 (DHS Direct)
Multiple Indicator Cluster Survey 2000 (MICS Indirect)
Demographic and Health Survey 2004 (DHS Indirect)
Demographic and Health Survey (MM adjusted) 2004 (DHS Direct)
Demographic and Health Survey 2004 (DHS Direct)
Census 2005 (Census Indirect)
Demographic and Health Survey (MM adjusted) 2011 (DHS Direct)
Demographic and Health Survey 2011 (DHS Direct)
Demographic and Health Survey (Preliminary) 2011 (DHS Direct)
Demographic and Health Survey (Preliminary) (MM adjusted) 2011 (DHS Direct)

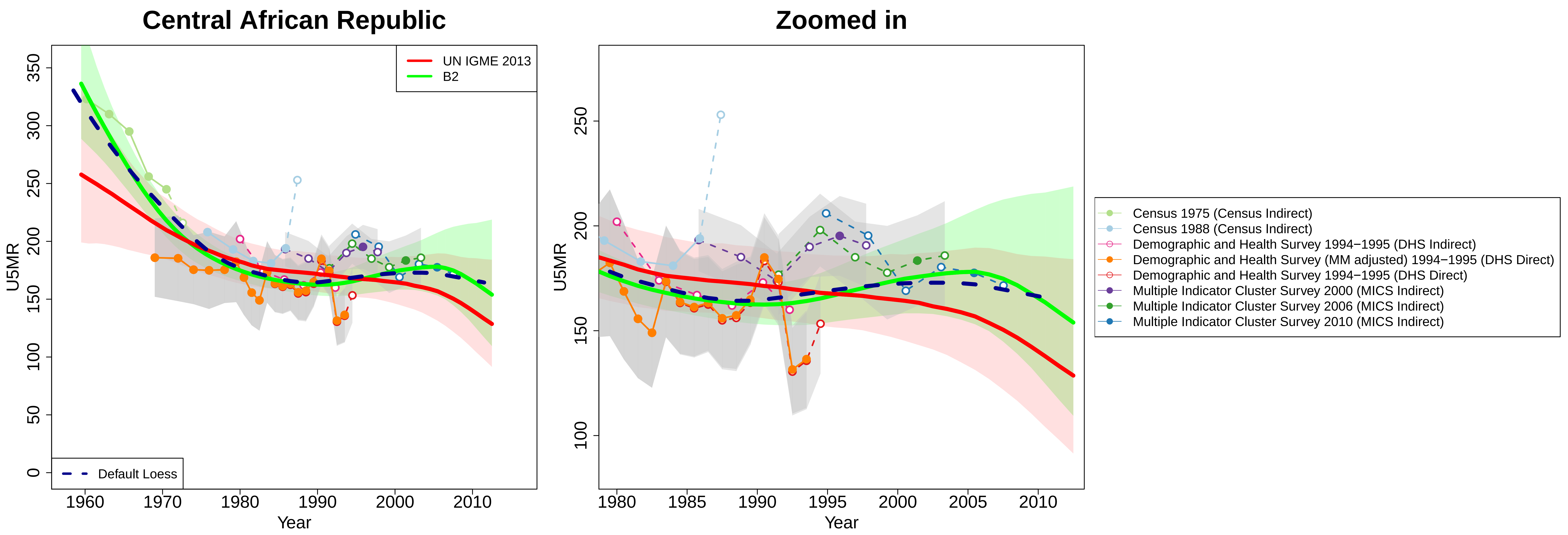
Central African Republic
Zoomed in
U5MR
Year
UN IGME 2013
B2
Default Loess
Census 1975 (Census Indirect)
Census 1988 (Census Indirect)
Demographic and Health Survey 1994−1995 (DHS Indirect)
Demographic and Health Survey (MM adjusted) 1994−1995 (DHS Direct)
Demographic and Health Survey 1994−1995 (DHS Direct)
Multiple Indicator Cluster Survey 2000 (MICS Indirect)
Multiple Indicator Cluster Survey 2006 (MICS Indirect)
Multiple Indicator Cluster Survey 2010 (MICS Indirect)

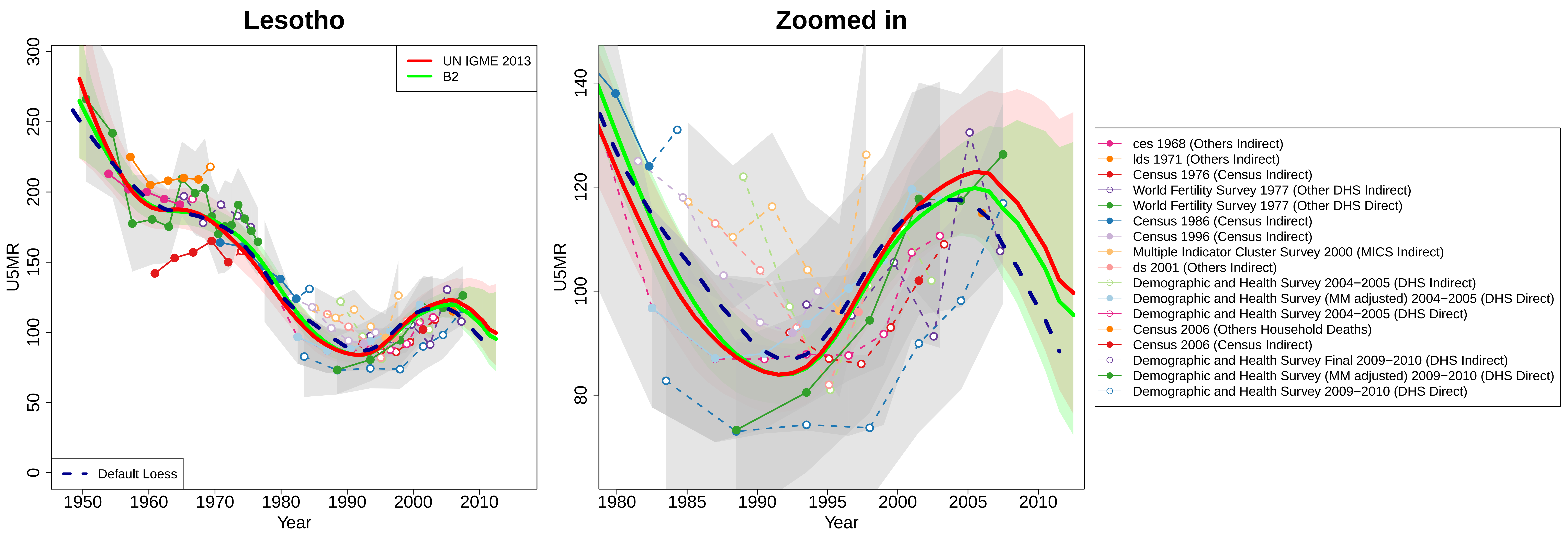
Lesotho
Zoomed in
UN IGME 2013
B2
Default Loess
U5MR
Year
ces 1968 (Others Indirect)
lds 1971 (Others Indirect)
Census 1976 (Census Indirect)
World Fertility Survey 1977 (Other DHS Indirect)
World Fertility Survey 1977 (Other DHS Direct)
Census 1986 (Census Indirect)
Census 1996 (Census Indirect)
Multiple Indicator Cluster Survey 2000 (MICS Indirect)
ds 2001 (Others Indirect)
Demographic and Health Survey 2004−2005 (DHS Indirect)
Demographic and Health Survey (MM adjusted) 2004−2005 (DHS Direct)
Demographic and Health Survey 2004−2005 (DHS Direct)
Census 2006 (Others Household Deaths)
Census 2006 (Census Indirect)
Demographic and Health Survey Final 2009−2010 (DHS Indirect)
Demographic and Health Survey (MM adjusted) 2009−2010 (DHS Direct)
Demographic and Health Survey 2009−2010 (DHS Direct)

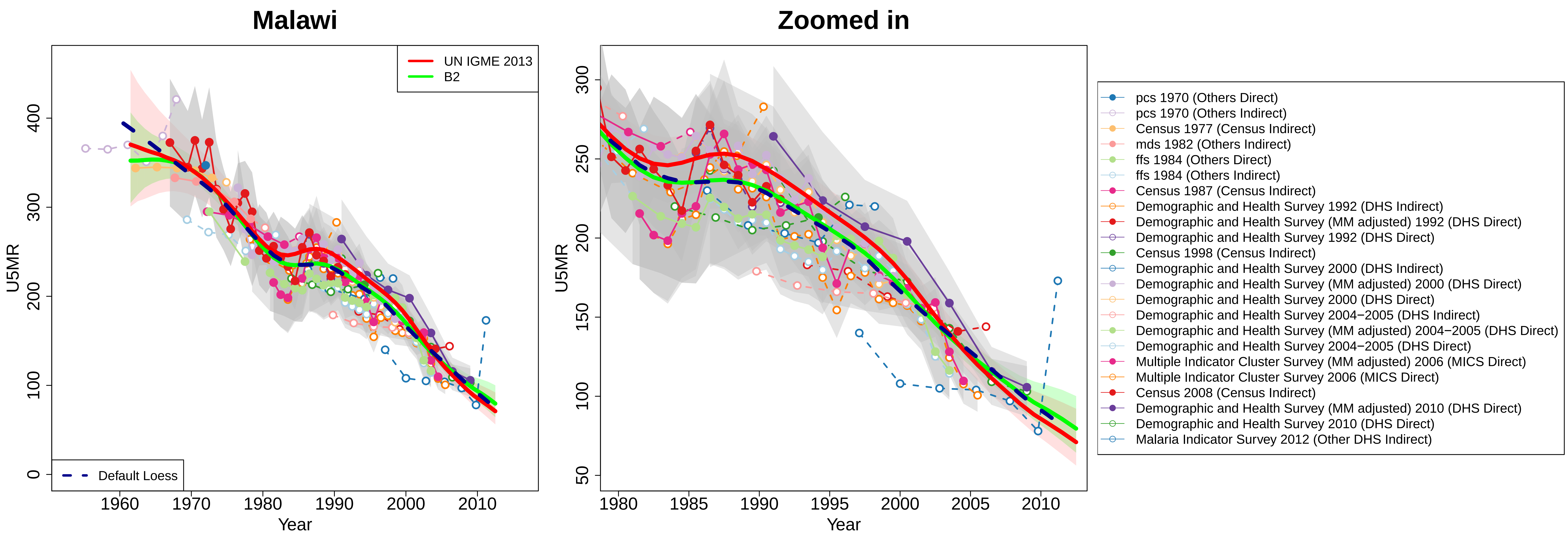

Malawi
Zoomed in
U5MR
Year
UN IGME 2013
B2
Default Loess
pcs 1970 (Others Direct)
pcs 1970 (Others Indirect)
Census 1977 (Census Indirect)
mds 1982 (Others Indirect)
ffs 1984 (Others Direct)
ffs 1984 (Others Indirect)
Census 1987 (Census Indirect)
Demographic and Health Survey 1992 (DHS Indirect)
Demographic and Health Survey (MM adjusted) 1992 (DHS Direct)
Demographic and Health Survey 1992 (DHS Direct)
Census 1998 (Census Indirect)
Demographic and Health Survey 2000 (DHS Indirect)
Demographic and Health Survey (MM adjusted) 2000 (DHS Direct)
Demographic and Health Survey 2000 (DHS Direct)
Demographic and Health Survey 2004−2005 (DHS Indirect)
Demographic and Health Survey (MM adjusted) 2004−2005 (DHS Direct)
Demographic and Health Survey 2004−2005 (DHS Direct)
Multiple Indicator Cluster Survey (MM adjusted) 2006 (MICS Direct)
Multiple Indicator Cluster Survey 2006 (MICS Direct)
Census 2008 (Census Indirect)
Demographic and Health Survey (MM adjusted) 2010 (DHS Direct)
Demographic and Health Survey 2010 (DHS Direct)
Malaria Indicator Survey 2012 (Other DHS Indirect)

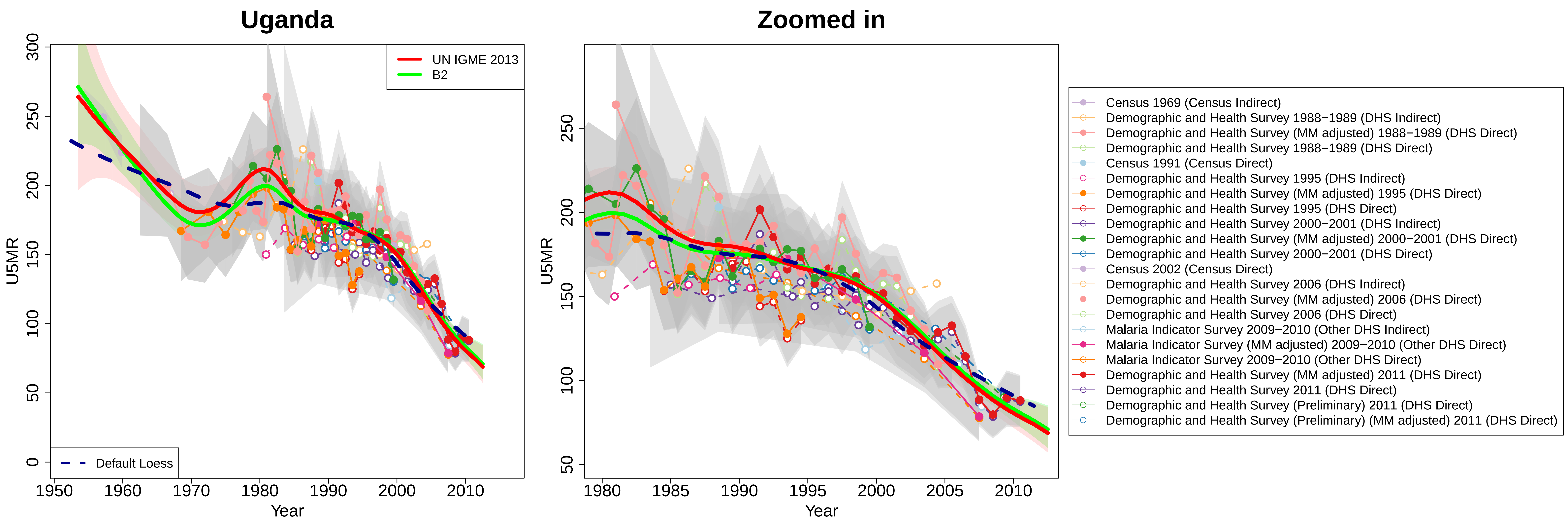

Uganda
Zoomed in
UN IGME 2013
B2
Default Loess
U5MR
Year
Census 1969 (Census Indirect)
Demographic and Health Survey 1988−1989 (DHS Indirect)
Demographic and Health Survey (MM adjusted) 1988−1989 (DHS Direct)
Demographic and Health Survey 1988−1989 (DHS Direct)
Census 1991 (Census Direct)
Demographic and Health Survey 1995 (DHS Indirect)
Demographic and Health Survey (MM adjusted) 1995 (DHS Direct)
Demographic and Health Survey 1995 (DHS Direct)
Demographic and Health Survey 2000−2001 (DHS Indirect)
Demographic and Health Survey (MM adjusted) 2000−2001 (DHS Direct)
Demographic and Health Survey 2000−2001 (DHS Direct)
Census 2002 (Census Direct)
Demographic and Health Survey 2006 (DHS Indirect)
Demographic and Health Survey (MM adjusted) 2006 (DHS Direct)
Demographic and Health Survey 2006 (DHS Direct)
Malaria Indicator Survey 2009−2010 (Other DHS Indirect)
Malaria Indicator Survey (MM adjusted) 2009−2010 (Other DHS Direct)
Malaria Indicator Survey 2009−2010 (Other DHS Direct)
Demographic and Health Survey (MM adjusted) 2011 (DHS Direct)
Demographic and Health Survey 2011 (DHS Direct)
Demographic and Health Survey (Preliminary) 2011 (DHS Direct)
Demographic and Health Survey (Preliminary) (MM adjusted) 2011 (DHS Direct)

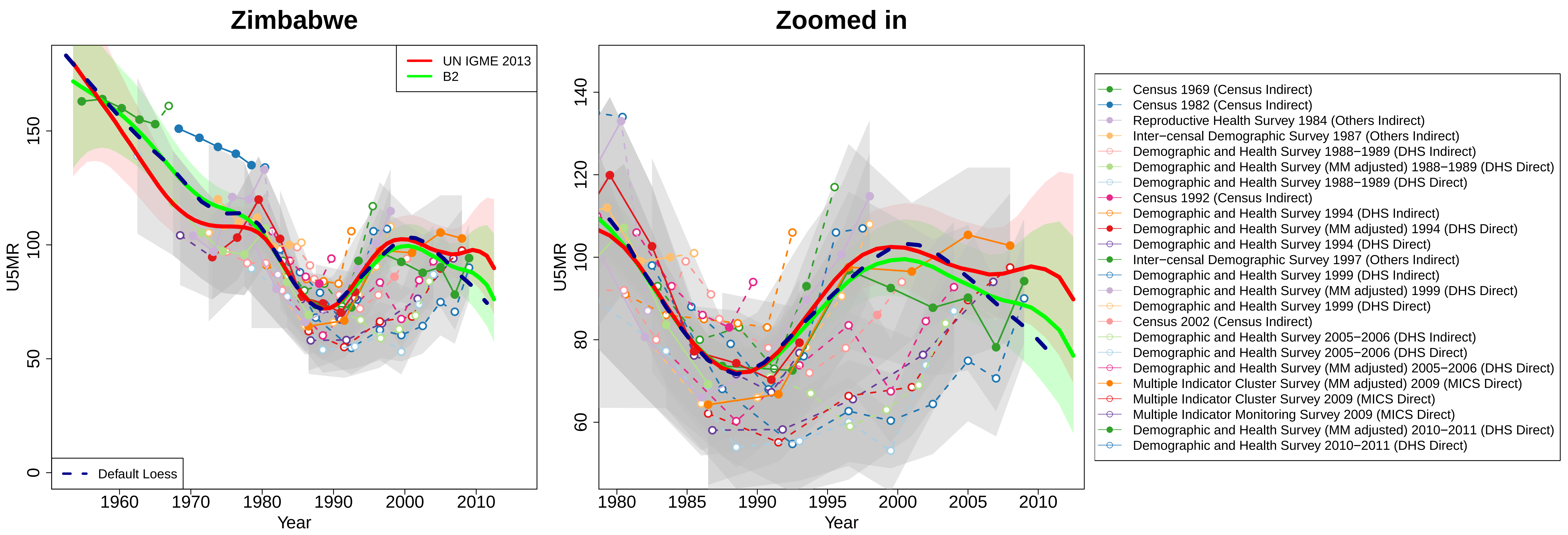
Zimbabwe
Zoomed in
U5MR
Year
UN IGME 2013
B2
Default Loess
Census 1969 (Census Indirect)
Census 1982 (Census Indirect)
Reproductive Health Survey 1984 (Others Indirect)
Inter−censal Demographic Survey 1987 (Others Indirect)
Demographic and Health Survey 1988−1989 (DHS Indirect)
Demographic and Health Survey (MM adjusted) 1988−1989 (DHS Direct)
Demographic and Health Survey 1988−1989 (DHS Direct)
Census 1992 (Census Indirect)
Demographic and Health Survey 1994 (DHS Indirect)
Demographic and Health Survey (MM adjusted) 1994 (DHS Direct)
Demographic and Health Survey 1994 (DHS Direct)
Inter−censal Demographic Survey 1997 (Others Indirect)
Demographic and Health Survey 1999 (DHS Indirect)
Demographic and Health Survey (MM adjusted) 1999 (DHS Direct)
Demographic and Health Survey 1999 (DHS Direct)
Census 2002 (Census Indirect)
Demographic and Health Survey 2005−2006 (DHS Indirect)
Demographic and Health Survey 2005−2006 (DHS Direct)
Demographic and Health Survey (MM adjusted) 2005−2006 (DHS Direct)
Multiple Indicator Cluster Survey (MM adjusted) 2009 (MICS Direct)
Multiple Indicator Cluster Survey 2009 (MICS Direct)
Multiple Indicator Monitoring Survey 2009 (MICS Direct)
Demographic and Health Survey (MM adjusted) 2010−2011 (DHS Direct)
Demographic and Health Survey 2010−2011 (DHS Direct)
